# Exploration of Biomarkers of Psoriasis through Combined Multiomics Analysis

**DOI:** 10.1155/2022/7731082

**Published:** 2022-09-23

**Authors:** Lu Xing, Tao Wu, Li Yu, Nian Zhou, Zhao Zhang, Yunjing Pu, Jinnan Wu, Hong Shu

**Affiliations:** ^1^Department of Dermatology, Kunming Children's Hospital, Kunming, China; ^2^Department of Colorectal Surgery, Third Affiliated Hospital of Kunming Medical University, Tumor Hospital of Yunnan Province, Kunming, China

## Abstract

**Background:**

Aberrant DNA methylation patterns are of increasing interest in the study of psoriasis mechanisms. This study aims to screen potential diagnostic indicators affected by DNA methylation for psoriasis based on bioinformatics using multiple machine learning algorithms and to preliminarily explore its molecular mechanisms.

**Methods:**

GSE13355, GSE14905, and GSE73894 were collected from the gene expression omnibus (GEO) database. Differentially expressed genes (DEGs) and differentially methylated region- (DMR-) genes between psoriasis and control samples were combined to obtain differentially expressed methylated genes. Subsequently, a protein-protein interaction (PPI) network was established to analyze the interaction between differentially expressed methylated genes. Moreover, the hub genes of psoriasis were screened by the least absolute shrinkage and selection operator (LASSO), Random Forest (RF), and Support Vector Machine (SVM), which were further performed single-gene gene set enrichment analysis (GSEA) to clarify the pathogenesis of psoriasis. The druggable genes were predicted using DGIdb. Finally, the expressions of hub genes in psoriasis lesions and healthy controls were detected by immunohistochemistry (IHC) and quantitative real-time PCR (RT-qPCR).

**Results:**

In this study, a total of 767 DEGs and 896 DMR-genes were obtained. Functional enrichment showed that they were significantly associated with skin development, skin barrier function, immune/inflammatory response, and cell cycle. The combined transcriptomic and DNA methylation data resulted in 33 differentially expressed methylated genes, of which GJB2 was the final identified hub gene for psoriasis, with robust diagnostic power. IHC and RT-qPCR showed that GJB2 was significantly higher in psoriasis samples than those in healthy controls. Additionally, GJB2 may be involved in the development and progression of psoriasis by disrupting the body's immune system, mediating the cell cycle, and destroying the skin barrier, in addition to possibly inducing diseases related to the skeletal aspects of psoriasis. Moreover, OCTANOL and CARBENOXOLONE were identified as promising compounds through the DGIdb database.

**Conclusion:**

The abnormal expression of GJB2 might play a critical role in psoriasis development and progression. The genes identified in our study might serve as a diagnostic indicator and therapeutic target in psoriasis.

## 1. Introduction

Psoriasis is a chronic recurrent inflammatory skin disease induced by the interaction of heredity and environment; with the typical clinical manifestation of scaly erythema or plaque, which is localized or widely distributed [1]. The incidence of psoriasis in developed countries is higher than that in developing countries, and it is more common in adults compared with children [2, 3]. In addition, the prevalence of psoriasis in gender is basically the same between men and women, with an average age of about 33 years [4]. The treatment and management of psoriasis have been dramatically changed thanks for the emergence of biologics [5], but there are still some defects in biologics. It has been mentioned that biologics is unsuitable for patients with hepatitis B, tuberculosis, and allergies [6–8]. At present, psoriasis remains incurable, which shows a high recurrence rate after drug withdrawal [9, 10]. The pathogenesis of psoriasis is not completely clear, but an increasing amount of evidence shows that the abnormal DNA methylation pattern is one of the most critical pathogenic factors, including differential methylation sites and differential methylation regions [11–13].

DNA methylation, an epigenetic regulatory mechanism, plays an essential role in gene expression, differentiation, cell proliferation, development, and genomic imprinting [14, 15]. It is mediated by DNA methyltransferase, which occurs on cytosine phosphate guanine (CPG) island and transfers the methyl of S-adenosylmethionine to the 5th carbon atom of cytosine ring [16]. DNA methylation is generally negatively correlated with gene expression [17]. Several studies have demonstrated that DNA methylation was closely related to the pathogenesis, severity, and treatment of psoriasis. The down-regulation of secreted frizzled-related protein 4 (SFRP4) caused by hypermethylation [18] or the up-regulation of B-cell receptor-associated protein 31 (BACP31) caused by hypomethylation [19] might lead to excessive proliferation and abnormal apoptosis of psoriasis keratinocytes. The results of genome-wide methylated DNA immunoprecipitation sequencing (MeDIP-Seq) on lesions and healthy skins of psoriasis patients showed that differential methylated regions (DMR) covered almost all genomes, and the methylation levels of tissue inhibitor of metalloproteinase 2(TIMP2) and programmed cell death 5(PDCD5) were positively correlated with the score of psoriatic area and severity index (PASI) [20]. Human leukocyte antigen (HLA) gene region also acts a significant role in the pathogenesis and development of psoriasis. Additionally, the methylation level of HLA-C promoter region in psoriasis patients was significantly higher than that in healthy controls. Further studies suggested that the hypermethylation of HLA-C could be involved in the pathogenesis of psoriasis by not affecting the expression of HLA-C [21]. The methylation degree of HLA-DRB1 in psoriasis lesions was remarkably lower than that of nonlesion tissues, which was negatively correlated with PASI score [22]. Furthermore, a DNA methylation spectrum analysis was performed on the genomes of 12 lesions of psoriasis patients pre and postultraviolet radiation B (UVB) treatment. The results demonstrated that the methylation status of 3665 methylation variable positions (MVP) in psoriasis samples had changed and the patient's condition had improved. It indicated that DNA methylation could be dynamically reversed in the treatment of psoriasis [23]. Therefore, the explorations of key genes related to DNA methylation and their biological function were extremely crucial to reveal the molecular mechanism of psoriasis and develop new therapeutic targets.

In this study, bioinformatics methods were utilized to analyze the transcriptome data and methylation data of psoriatic lesions and healthy controls from GEO database. Next, multiple machine learning algorithms were performed to screen the key genes related to DNA methylation. Finally, the accuracy and expression of key gene models were verified by ROC curves and molecular biological experiments. In conclusion, this study identified key diagnostic indicators and therapeutic targets of psoriasis by combined multiomics analysis.

## 2. Materials and Methods

### 2.1. Data Source

The psoriasis-related data utilized for this study were obtained from the GEO database. Gene expression profiling array GSE13355 (https://www.ncbi.nlm.nih.gov/geo/query/acc.cgi?acc=GSE13355) [24–26] provided mRNA expression data from 122 skin biopsy samples with 58 psoriasis lesions and 64 healthy control skin. Additionally, the gene expression profiling of 33 psoriasis lesions and 21 healthy control skin were obtained from GSE14905 (https://www.ncbi.nlm.nih.gov/geo/query/acc.cgi?acc=GSE14905) [27]. Both datasets were generated by the platform GPL570 (Affymetrix Human Genome U133 Plus 2.0 Array).

The DNA methylation MBD-seq data were obtained under accession number GEO: GSE73894 (GPL13534 platform; https://www.ncbi.nlm.nih.gov/geo/query/acc.cgi?acc=GSE73894) [28, 29], which includes 114 psoriasis lesions and 64 healthy control skin samples.

### 2.2. Differentially Expressed Genes Analysis

R package limma [30] was used in the screening of differentially expressed gene (DEG) between psoriasis lesions and control skin samples, with the screening criteria of absolute value of log_2_ fold change (FC) > 1 and *P* < 0.05.

### 2.3. Analysis of Differentially Methylated Regions

The Bumphunter function of the R package ChAMP was used for the differentially methylated region (DMR) analysis. The parameter setting was as follows: a minimum number of probes in the methylation region (minProbes) ≥7, adjusted (adj.) *P* < 0.05. The methylation level of the gene was represented by the average beta value of CpG in different regions of the gene. The beta value matrix was analyzed by the R package limma to screen differentially methylated genes (DMR-genes), and the |Δβ| > 0.1 was set as the threshold. The distribution of genes in different gene regions was visualized by the R package named “Upset” [31]. Finally, DMR-genes were overlapped with DEGs, and the intersected genes represented differentially expressed methylated genes.

### 2.4. Functional Enrichment Analysis

Gene ontology (GO) [32] and Kyoto Encyclopedia of Genes and Genomes (KEGG) [33] pathway enrichment analyses were performed to the differentially expressed methylated genes by the R package clusterProfiler [34]. The results satisfied *P* < 0.05 and count > 2 were considered as statistically significant.

### 2.5. Protein-Protein Interaction (PPI) Network Analysis

To explore the interaction between differentially expressed methylated genes, these genes were uploaded to the STRING database (https://string-db.org) with the cutoff value set as 0.15 to receive the interaction relationship information between genes. Then, the interactive information was visualized into a imported into a PPI network by Cytoscape [35]. The top 10 genes with the highest connectivity (degree) in the PPI network were identified as key genes for subsequent analysis.

### 2.6. Integrating Multiple Machine Learning Algorithms to Identify Hub Genes

Three machine learning algorithms were implemented to filter feature genes, including least absolute shrinkage and selection operator (LASSO), Random Forest classifier (RF), and support vector machine recursive feature elimination (SVM-RFE), using a 10-fold cross-validation approach.

LASSO [36] was employed by R package glmnet with the parameters set as famil = binomial and type.measure = class. In the RF [37], the importance and importance ranking of each gene were obtained using the RFE method, and the error rate and accuracy rate of the combination in each iteration were obtained, which was employed by R package Random Forest. The characteristic genes were the corresponding genes in the best combination with the lowest error rate. Meanwhile, SVM-RFE [38] was performed by R package e1071, and the 10-fold cross-validation algorithm was applied as the resampling method for SVM-RFE. The final importance of features was based on the average importance of each feature in each iteration.

Afterward, the genes within the intersection of three subsets were selected as hub genes for subsequent analyses.

### 2.7. The Receiver Operating Characteristic (ROC) Curve Analysis

The receiver operating characteristic (ROC) curve analysis was used to evaluate the discrimination ability of the hub gene in the GSE13355 dataset; the discrimination ability of each model was quantified by the area under the ROC curve (AUC). The reliability of these gene predictions would be verified in the independent GSE14905 dataset. The ROC analysis was achieved through the R package pROC [39].

### 2.8. Single-Gene Gene Set Enrichment Analysis (GSEA) Analysis

Single-gene GSEA was conducted based on the gene list sorted by Spearman correlation coefficient between every gene and the specified hub gene to predict the significant biological processes and pathways associated with the hub gene. The background gene sets for GO and KEGG were obtained from the MSigDB database (https://www.gsea-msigdb.org/gsea/msigdb/). The Normalized Enrichment Score (NES)|>1, *P* < 0.05, and *q* < 0.2 were considered significant thresholds.

### 2.9. Drug and Hub Gene Interaction Analysis

The drug-gene interaction database (DGIdb; https://www.dgidb.org/) was used to investigate potential diagnosis-related gene therapy targets for hub genes [40].

### 2.10. Immunohistochemical Staining

8 psoriasis lesions and 11 healthy skin tissues were made into paraffin blocks, which were then cut into sections at a thickness of 5 *μ*m by a slicer (Leica Co., Ltd., Shanghai, China), followed by baking at 50°C. The sections were dewaxed twice using xylene (5 min each), and then dehydrated by graded ethanol with certain concentration separately (3 min each). The endogenous peroxidase in the tissue sections was blocked with methanol containing 0.3% H_2_O_2_. The sections were then incubated with the anti-connexin 26 (Cx26) antibody (1 : 100,Ab65969,Abcam, Cambridge, UK) as primary antibodies at 4°C by the streptavidinbiotin peroxidase (SP) coupling two-step method and standard SP kit. Pathological changes were observed under an optical microscope (DMM-300D, Shanghai Caikon Optical Instrument Co., Ltd., Shanghai, China) (×200) and photographed.

### 2.11. RNA Isolation and Quantification

The total RNA from 8 psoriasis lesions and 11 healthy skin tissues were extracted based on the Trizol method (Cat:9109, Takara, Dalian, China). Total RNA was reversely transcribed into cDNA using a reverse transcription kit (Cat:KR118-02, TianGen, Beijing, China), after which quantitative PCR (qPCR) amplification analysis was conducted. Primers of GJB2 and GAPDH were designed and then synthesized by Sangon company (Sangon Biotech, Shanghai, China) **(**Table 1**)**. The qPCR was subsequently conducted using the dNTP mixture (Cat:FP205-02, TianGen, Beijing, China) on the 7500 Real-Time PCR Systems (Applied Biosystems, Thermo Fisher Scientific, Foster city, California). GAPDH was regarded as the internal reference. 2^−ΔCt^ was employed to determine the expression ratio of the target gene in the psoriasis group to that of the healthy group using the following formula: ΔCt = Ct (GJB2)–Ct (GAPDH). The experiment was independently repeated three times.

### 2.12. Statistical Analysis

The R software packages were used in the statistical analysis. All network plots were visualized by Cytoscape software. The R package (ggplot2, Pheatmap, GOplot, UpSetR, VennDiagram, ggpubr) was used for visualization (volcano plot, heat map, GO/KEGG enrichment plot, upset plot, Venn diagram, box line plot). The difference in hub genes between normal and psoriasis samples was detected by the Wilcoxon rank-sum test. *P* < 0.05 was set as the threshold of significance if not otherwise stated.

## 3. Results

### 3.1. Analysis of the Psoriasis-Related DEGs in the GSE13355 Dataset

**Figure 1:**
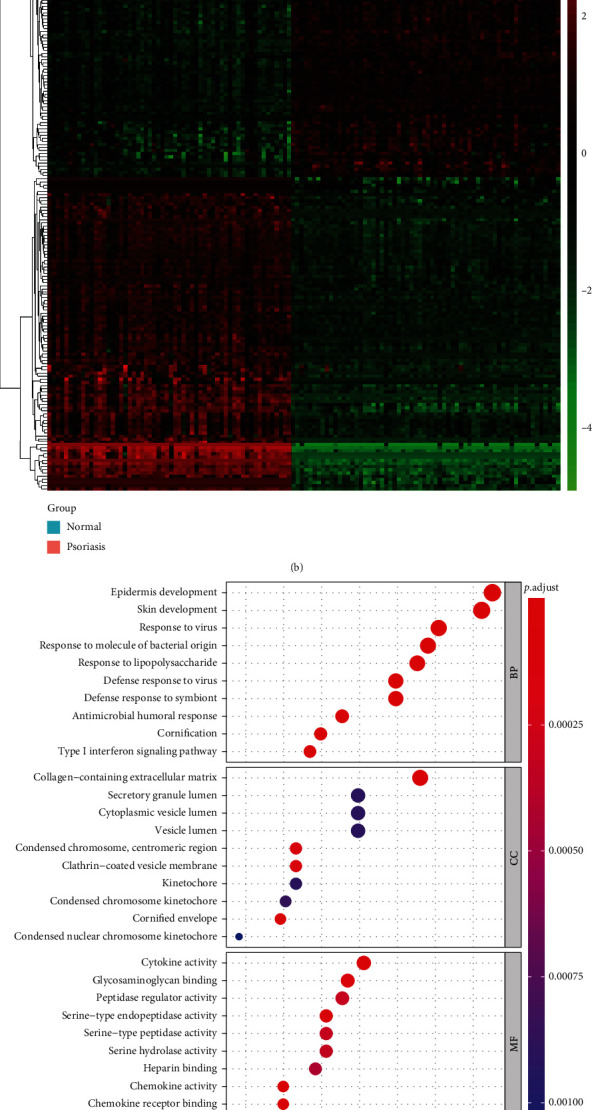
Analysis of DEGs between psoriasis lesions and healthy controls in GSE13355. (a) Volcano map of DEGs in GSE13355. (b) Heat map of top 100 DEGs between psoriasis and healthy samples in GSE13355. (c) Top 10 enriched GO terms by 767 DEGs in each category. (d) Top 10 enriched KEGG pathways by 767 DEGs.

After background correction and Robust Multichip Average (RAM) normalization of gene expression profiles from skin samples of 58 psoriasis patients and 64 healthy subjects in the GSE13355 dataset using the R package affy (V 1.70.0), PCA showed that the same type of samples in this dataset had aggregation properties (Supplementary Figure 1). Then, a total of 767 DEGs were identified, of which 448 genes were expressed upregulated and 319 genes were downregulated in psoriasis samples (Figure 1(a); Supplementary Table 1). The heat map demonstrated the differential expression patterns of top 100 upregulated and downregulated genes between the two groups (Figure 1(b)).

Furthermore, GO enrichment analysis of DEGs (Figure 1(c); Supplementary Table 2) revealed that in the BP category, DEGs were significantly associated with skin development, viral/bacterial response, immune/inflammatory response, and cell cycle, such as epidermis development, positive regulation of epidermal growth factor-activated receptor activity, response to virus, defense response to fungus, type 2 immune response, response to chemokine, and organelle fission. Furthermore, these genes were inextricably linked to positive regulation of wound healing, establishment of skin barrier, regulation of response to wounding, and several physiological processes such as differentiation, migration, and chemotaxis of various immune cells (e.g., leukocytes, neutrophils, T cells, macrophages). In the CC category, cornified envelope, collagen-containing extracellular matrix, condensed chromosome, centromeric region, clathrin-coated vesicle membrane, and condensed chromosome kinetochore were the five most significantly enriched terms (Supplementary Table 2). Moreover, the MF category indicated that these genes were remarkably relevant to chemokines, cytokines, and growth factors such as chemokine activity, CCR chemokine receptor binding, cytokine activity, and epidermal growth factor receptor binding (Supplementary Table 2). In addition, KEGG analysis illustrated that these genes were involved in a total of 9 pathways, including those associated with viral infection (‘Influenza A' and ‘Hepatitis C'), inflammatory responses (‘Viral protein interaction with cytokine and cytokine receptor,' ‘IL-17 signaling pathway,' ‘Cytokine-cytokine receptor interaction,' and ‘Chemokine signaling pathway'), and cancer (‘PPAR signaling pathway' and ‘Prostate cancer'). Moreover, the Pyrimidine metabolism pathway was also significantly enriched **(**Figure 1(d); Supplementary Table 3).

### 3.2. DNA Methylation Profiling of Human Psoriasis in the GSE73894 Dataset

**Figure 2:**
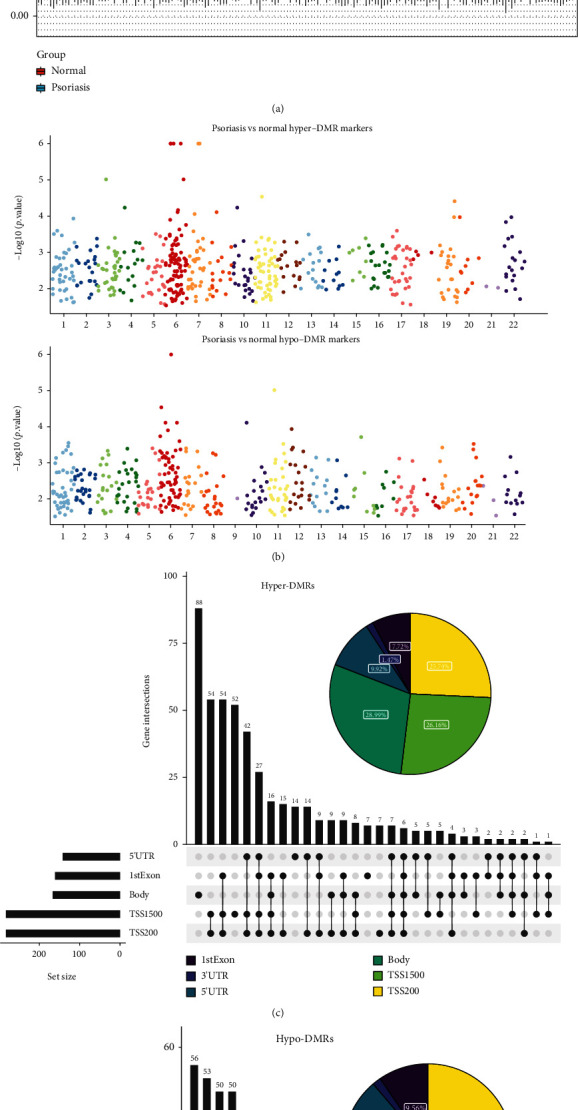
DNA methylation profiling of healthy controls and psoriasis lesions in GSE73894. (a) DNA methylation levels in healthy controls and psoriasis lesions. (b) Chromosome distribution of DMRs. The number of dots represents the distribution of DMR across different chromosomes. (c and d) Genomic distribution of the hyper-DMRs and hypo-DMRs. Pie charts represent the proportion of DMRs in different genomic contexts. Upset graphs represent the number of DMRs distributed in single or combined genomic regions.

DNA methylation is a highly stable epigenetic mark associated with disease pathogenesis [13]. DNA methylation has been reported to be one of the important factors in the differentiation of keratin-forming cells [16, 41], which prompted us to speculate that DNA methylation is essential for the development of psoriasis. To characterize aberrant DNA methylation in psoriasis, the overall DNA methylation levels in 64 healthy control skins and 114 lesioned skins of psoriasis patients from the GSE73894 dataset were evaluated, and the result indicated methylation levels in lesioned skins of psoriasis were relatively high compared to healthy control skins **(**Figure 2(a)**)**. Subsequently, the methylation data were quality-controlled and normalized by the R package ChAMP. A total of 73107 low-quality probes were filtered out from 485577 probes. PCA (Supplementary Figure 2A) and methylation distribution density (Supplementary Figure 2B) analyses based on the beta value of the methylation sites demonstrated the reliability of the data. Further, we performed DMR analysis using the Bumphunter method of the R package ChAMP and identified a total of 961 DMRs, which were classified into hyper-MRs and hypo-MRs based on|value| > 0.1**(**Figure 2(b)**)**. Subsequently, 896 corresponding genes in the DMRs were identified. The hyper-MRs contained a total of 480 genes (hyper-MR-genes; Supplementary Table 4) and the hypo-MRs included 436 genes (hypo-MR-genes; Supplementary Table 5), of which 20 genes were hypermethylated and hypomethylated in different regions. We then mapped DMRs to the entire genome by creating an Upset map and found that both hyper- **(**Figure 2(c)**)** and hypo- **(**Figure 2(d)**)** MR-genes were mainly concentrated in TSS200, TSS1500, and body.

**Figure 3:**
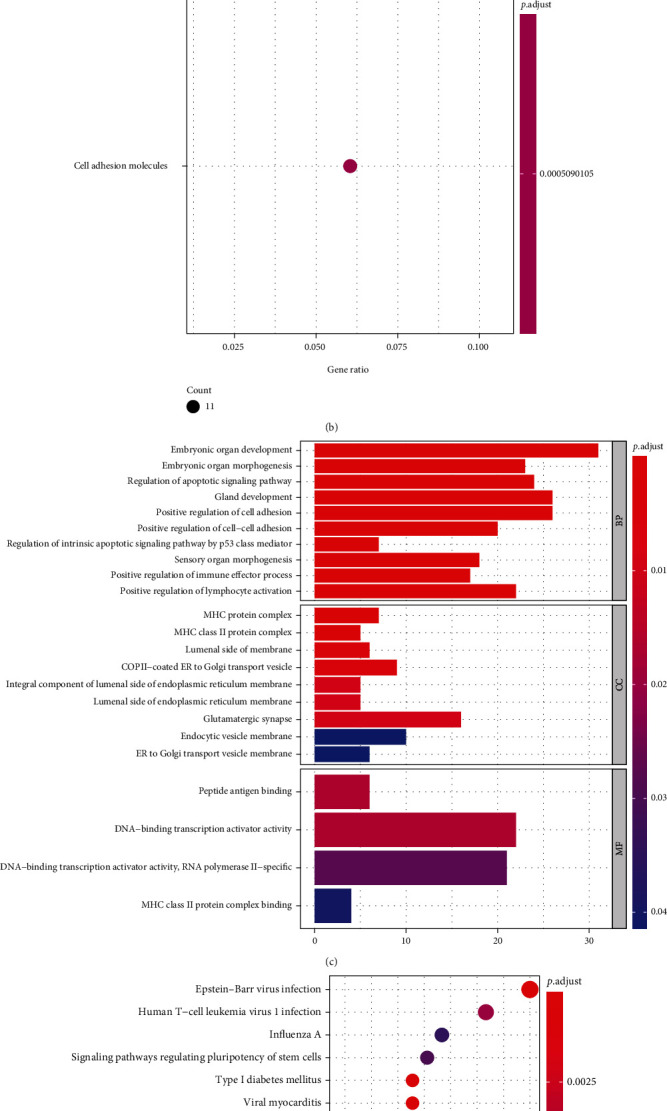
Functional enrichment analysis of the hyper-MR- and hypo-MR-genes. (a) GO enrichment result of hyper-MR-genes. (b) Enriched KEGG pathway of hyper-MR-genes. (c) GO enrichment result of hypo-MR genes. (d) Enriched KEGG pathways of hypo-MR genes.

To investigate the potential regulatory mechanisms of aberrant DNA methylation in psoriasis more closely, a functional enrichment analysis was performed on the hyper-MR- and hypo-MR-genes, respectively. GO analysis showed that the hyper-MR-genes were mainly enriched in the skeletal system (‘embryonic skeletal system development', ‘embryonic skeletal system morphogenesis', and ‘skeletal system morphogenesis'; BP), ‘pancreatic juice secretion' (BP), ‘body fluid secretion' (BP), and MHC-related terms [‘MHC class II protein complex' (CC), ‘MHC protein complex' (CC), and ‘MHC class II receptor activity' (MF)] **(**Figure 3(a); Supplementary Table 6). GO analysis of hypo-MR-genes (Figure 3(c); Supplementary Table 7) indicated that in the BP category, these genes were tightly correlated with tissue/organ development, immune response, immune cell biological processes, cell cycle, and apoptosis, including embryonic organ development, positive regulation of immune effector process, T cell activation, cell cycle G1/S phase transition, regulation of apoptotic signaling pathway, etc. In the CC category, MHC protein complex, MHC class II protein complex, and lumenal side of the membrane were the three most enriched terms. Moreover, a total of four MF terms, peptide antigen binding, DNA-binding transcription activator activity, DNA-binding transcription activator activity, RNA polymerase II-specific, and MHC class II protein complex-binding were enriched. KEGG analysis demonstrated that hyper-MR-genes enriched only 1 pathway, which was Cell adhesion molecules (Figure 3(b); Supplementary Table 8); whereas hypo-MR-genes enriched a total of 35 KEGG pathways, including multiple diseases, viral infections, inflammatory responses, immune cells, and apoptosis, which contained Type I diabetes mellitus, Epstein-Barr virus infection, Allograft rejection, Th1 and Th2 cell differentiation, Apoptosis, etc. (Figure 3(d); Supplementary Table 9).

### 3.3. Identification and Analysis of Key Differentially Expressed Methylation Genes

**Figure 4:**
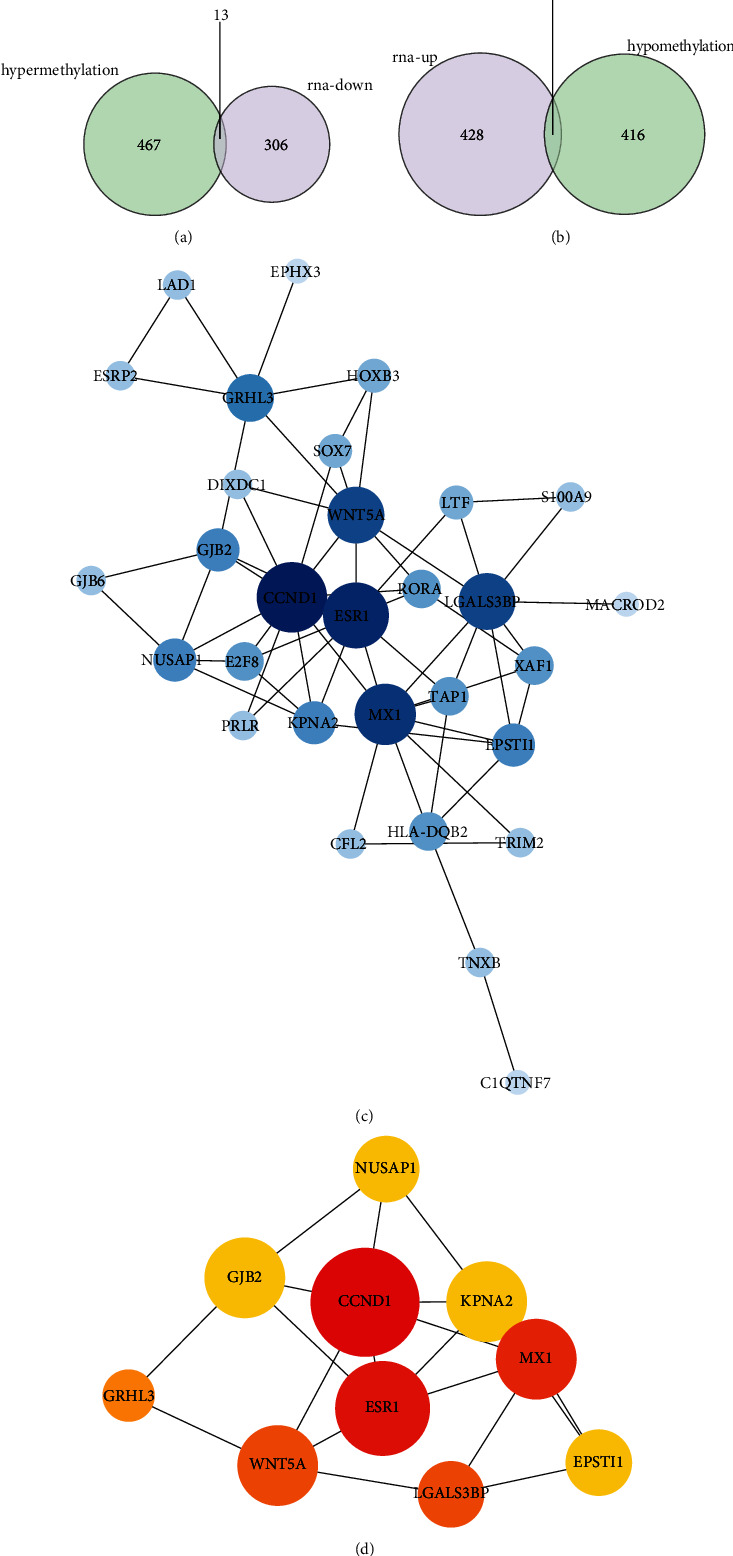
Identification and analysis of key differentially expressed methylation genes. (a) Venn diagram of hyperdownregulated genes identification. (b) Venn diagram of hypoupregulated genes identification. (c) A PPI network for 30 differentially expressed methylated genes. (C) Identification of 10 key genes.

To obtain differentially expressed methylated genes, we performed an overlap analysis of 767 psoriasis-related DEGs and 896 DMR-genes obtained above **(**Figures 4(a) and 4(b)**)**. Thirteen common genes were identified in the list of downregulated DEGs and hyper-DMR-genes **(**Figure 4(a)**)**, namely, TRIM2, HOXB3, TNXB, C1QTNF7, ESR1, CFL2, CCND1, DIXDC1, HLA-DQB2, PRLR, MACROD2, RORA, and ZSCAN18, which were termed as hyperdownregulated genes; while there were 20 common genes in the list of upregulated DEGs and hypo-DMR-genes **(**Figure 4(b)**)**, namely, TAP1, S100A9, EPSTI1, GJB2, GRHL3, TTC22, SOX7, WNT5A, XAF1, GJB6, LAD1, POLR3G, KPNA2, E2F8, MX1, LTF, EPHX3, LGALS3BP, NUSAP1, and ESRP2, defined as hypoupregulated genes. The acquired genes above were collectively labeled as differentially expressed methylation genes.

Subsequently, a PPI network was constructed for the 33 differentially expressed methylated genes by STRING online analysis tool. After removing discrete nodes (confidence = 0.15), a PPI network was obtained, which contained 30 genes **(**Figure 4(c)**)**, and 61 edges. Furthermore, the top 10 genes with the highest degree were identified as key genes, namely, *CCND1* (degree = 11), *ESR1* (degree = 10), *MX1* (degree = 9), *WNT5A* (degree = 8), *LGALS3BP* (degree = 8), *GRHL3* (degree = 6), *NUSAP1* (degree = 5), *GJB2* (degree = 5), *KPNA2* (degree = 5), and *EPSTI* (degree = 5), and the complex interactions between them were displayed in Figure 4(d).

### 3.4. GJB2 Was a Diagnostic Indicator for Psoriasis

**Figure 5:**
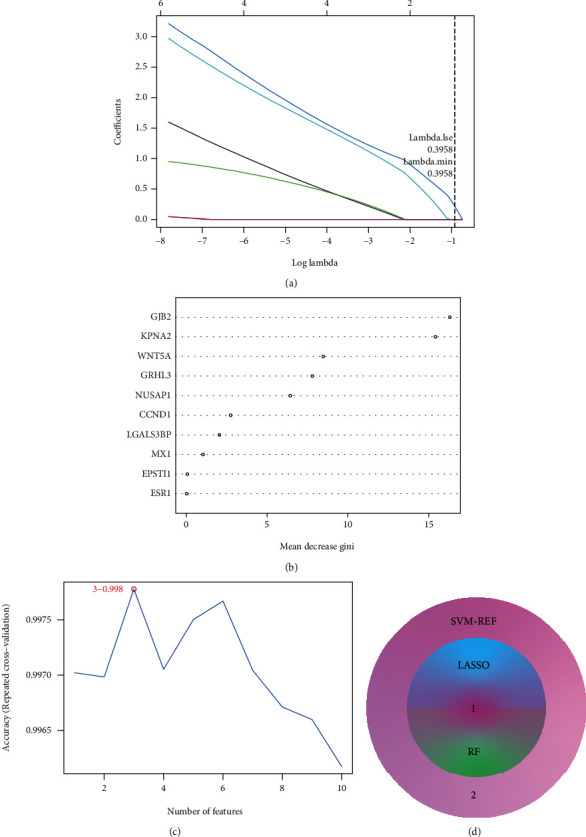
Identification of the hub gene. (a) Identification of feature gene by Least absolute shrinkage and selection operator (LASSO) regression analysis. The horizontal axis represents the lambda value and the vertical axis represents the independent variable coefficient. (b) Screen diagnostic markers by Random Forest (RF) model algorithm. The importance of features ranked by mean decrease Gini. (c) Selection genes by the support vector machine recursive feature elimination (SVM-RFE) algorithm. (d) Intersection analysis of feature genes identified by three algorithms.

To obtain reliable and robust biomarkers and to reduce the possibility of overfitting, we employed three machine learning methods in the GSE13355 dataset, including LASSO regression, RF, and SVM-RFE for three-pass authentication. The optimal value of *λ* was set at 0.3958 based on the minimum criterion for LASSO regression, at which point one predictive feature (GJB2) with a non-zero coefficient was identified among the 10 key genes **(**Figure 5(a)**)**. The importance of each feature in the RF model was illustrated in Figure 5(b), and after calculating the accuracy of the model under different features using 10-fold cross-validation, it was determined that the model was the most accurate when GJB2 was selected. In SVM-RFE, the accuracy of each combination of iterations was calculated by 10-fold cross-validation, which revealed that the SVM model appeared to have the best prediction performance when the first three genetic features (GJB2, WNT5A, and KPNA2) were included **(**Figure 5(c)**)**. Then, GJB2 was the only overlapped gene among the three machine learning methods, which was identified as the hub gene for psoriasis **(**Figure 5(d)**)**.

**Figure 6:**
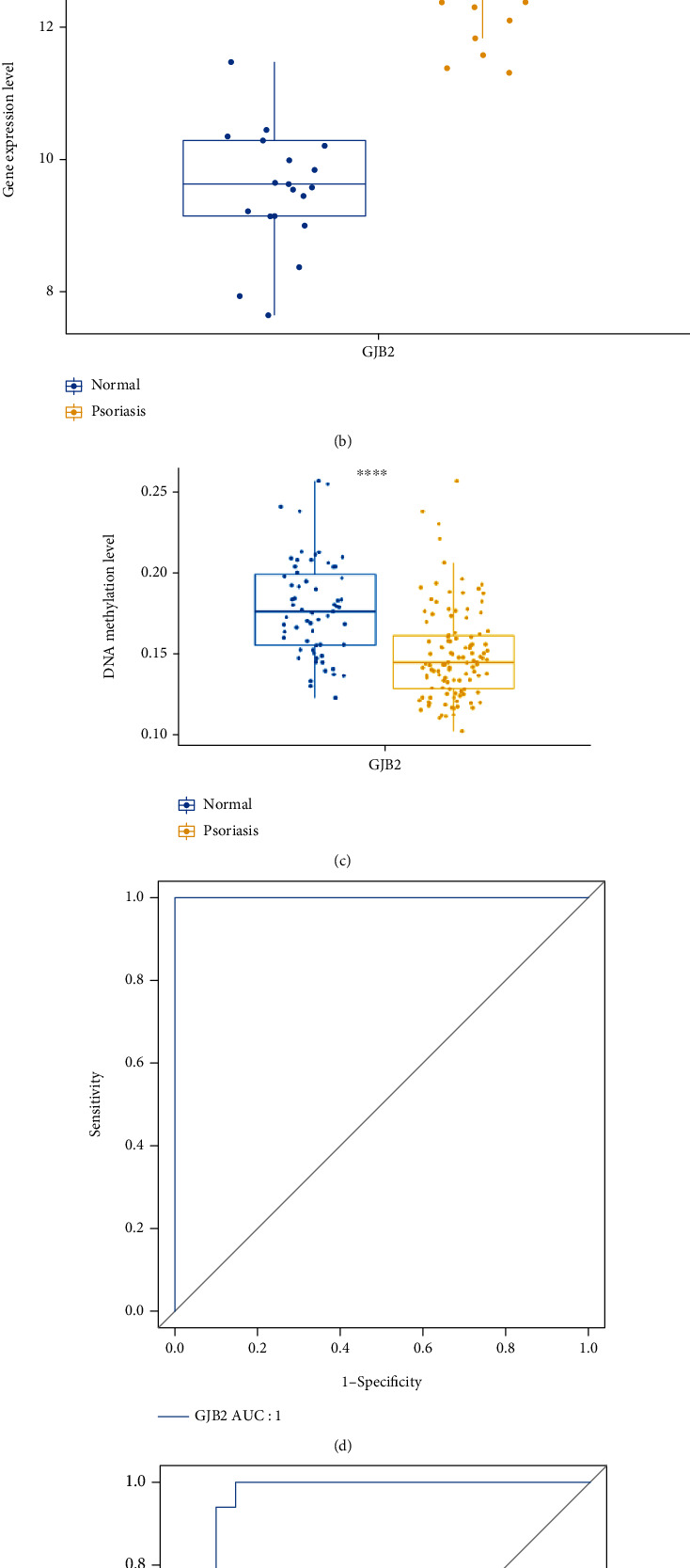
Evaluation of diagnostic value of GJB2 by Wilcoxon rank-sum test and ROC curves. (a) The expression of GJB2 between healthy controls and psoriasis lesions in GSE13355. (b) The expression of GJB2 between healthy controls and psoriasis lesions in GSE14905. (c) The DNA methylation levels of GJB2 between healthy controls and psoriasis lesions in GSE73894. (d) Evaluation of GJB2 AUC in GSE13355. (e) Evaluation of GJB2 AUC in GSE14905.

The Wilcoxon rank-sum test results of GJB2 illustrated that GJB2 was significantly overexpressed in psoriatic lesioned skin samples compared to healthy control skin samples (all *P* < 0.0001) (Figures 6(a) and (b)**)**. Moreover, we assessed the DNA methylation levels of GJB2 between the normal and psoriasis groups in GSE73894, and the results showed that the DNA methylation levels of GJB2 were significantly lower in the psoriasis group **(**Figure 6(c)**)**. Further, ROC curves indicated that GJB2 was able to effectively differentiate between psoriatic samples and healthy control samples both in the GSE13355 dataset and in the GSE14905 dataset, with an AUC of 1 in the GSE13355 dataset **(**Figure 6(d)**)** and an AUC of 0.965 in the GSE14905 dataset **(**Figure 6(e)**)**. This evidence suggested that GJB2 was a potential diagnostic marker for psoriasis.

### 3.5. Single-Gene GSEA of GJB2

**Figure 7:**
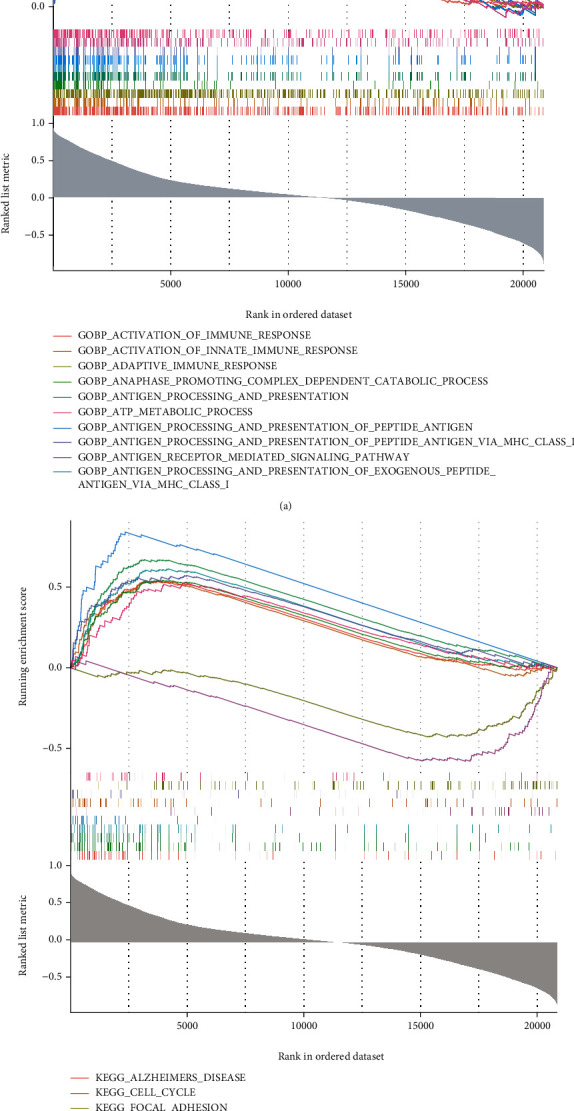
Single-gene GSEA of GJB2. (a) The top 10 enriched GO terms. (b) The top 10 enriched KEGG pathways.

To further explore the potential molecular mechanisms of hub gene involvement in the psoriasis process, we performed a single-gene GSEA of GJB2. The top 10 enriched terms from GO analysis were demonstrated in Figure 7(a), all of which were in the BP category and closely associated with immune response, antigen processing, such as activation of immune response, antigen processing and presentation, antigen receptor mediated signaling pathway, and anaphase promoting complex dependent catabolic process. Besides, GJB2 was also notably linked to cell cycle, tissue/organ growth and development, and inflammatory response, including cell cycle g2 m phase transition, DNA replication, kidney epithelium development, skeletal system morphogenesis, and positive regulation of cell-cell adhesion. More importantly, some terms related to skin development were also significantly enriched, such as regulation of cysteine type endopeptidase activity, keratinocyte differentiation, keratinization, epidermis development, and regulation of morphogenesis of an epithelium (Supplementary Table 10). KEGG analysis showed that GJB2 was significantly associated with a variety of diseases (Alzheimers disease, huntingtons disease, parkinsons disease, etc.), cell biological processes (cell cycle, DNA replication, p53 signaling pathway, TGF beta signaling pathway, JAK stat signaling pathway, etc.), and immune/inflammatory response (antigen processing and presentation, allograft rejection, primary immunodeficiency, cytokine cytokine receptor interaction, etc.) related pathways (Figure 7(b); Supplementary Table 11). This evidence suggested that GJB2 may be involved in the development and progression of psoriasis by disrupting the body's immune system, mediating the cell cycle, and destroying the skin barrier, in addition to possibly inducing diseases related to the skeletal aspects of psoriasis, such as arthritic psoriasis, or being critical in the process of skeletal aberrations in patients with psoriasis treated with hormonal therapy.

### 3.6. Prediction of Potential Drugs Targeting GJB2

GO-BP enrichment analysis revealed that GJB2 was inextricably linked to pteridine-containing compound metabolic process, pteridine-containing compound biosynthetic process, and tetrahydrofolate metabolic process (Supplementary Table 10). Drugs commonly used in psoriasis, such as methotrexate, have been reported to restore the normal methylation state by interfering with the methyl transfer function of folic acid [42]. Inspired by this, we believed that GJB2 was most likely a hopeful therapeutic target to be developed for psoriasis. Through the DGIdb database, the interaction of GJB2 with molecular drugs was predicted, and a total of 2 compounds were put forward as inhibitors of GJB2, namely OCTANOL and CARBENOXOLONE **(**Table 2**)**. These drugs could be the potential effective antipsoriasis drugs in the future.

### 3.7. Relatively High Expression of GJB2 in Psoriasis

**Figure 8:**
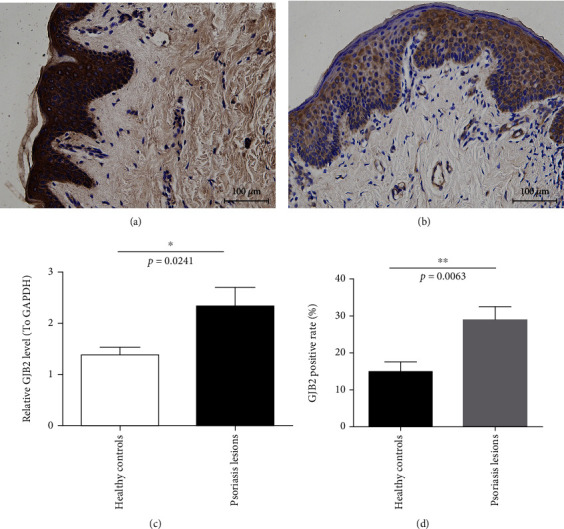
GJB2 expression in healthy controls and psoriasis lesions. (a) Immunohistochemical staining of GJB2 protein in psoriasis lesions. (b) Immunohistochemical staining of GJB2 protein in healthy controls. (c) Quantitative analysis of GJB2 mRNA in healthy controls and psoriasis lesions by qPCR.

To further investigate the expression of GJB2 in psoriasis, we performed IHC staining and real-time qPCR using 8 psoriasis lesions and 11 healthy skin tissues. Just as we expected, IHC staining suggested that the protein level of connexin 26 (Cx26) was markedly higher in psoriasis lesions than in healthy controls **(**Figures 8(a), 8(b) and 8(d)**)**. Moreover, it can be found from the real-time qPCR result that the mRNA expression of IGF2BP3 in psoriasis lesions are upregulated compared to healthy controls **(**Figure 8(c)**)**.

## 4. Discussion

Identifying molecular targets and regulatory mechanisms related to DNA methylation would contribute to the diagnosis and treatment of psoriasis. In this study, we identified 767 psoriasis-related DEGs and 896 DMR-genes in various GEO databases. 30 hyperdownregulated genes and 20 hypoupregulated genes were screened by overlap analysis. Through the construction of the PPI network, 10 key genes were selected, and GJB2 was finally identified as the hub gene filtered by multiple machine learning algorithms. At the same time, we conducted a single-gene GSEA on GJB2, and the result suggested that it might be involved in the development and progression of psoriasis by disrupting the body's immune system, mediating the cell cycle, and destroying the skin barrier. Finally, the expression and methylation level of GJB2 in psoriasis were verified by the external dataset and qRCR [43].

Gap junction beta 2 (GJB2) gene is located in 13q11-12 region, with a total length of 5.5 kb, encoding gap junction protein connexin 26 (Cx26) [44]. Gap junction channels allow the exchange of ions, second messengers, and metabolites less than 1 kDa between adjacent cells, which acts a significant role in regulating homeostasis and tissue differentiation [45]. Screening gene mutation of GJB2 could contribute to gene diagnosis and genetic counseling in families with Non-Syndromic Hearing Loss (NSHL) [46]. Connexin 26 missense mutation can cause palmoplantar keratoderma associated with sensorineural hearing loss [47] and temporal bones with cochlear otosclerosis [48]. The above diseases are largely caused by the thickening of the skin epidermis, which reveals a critical pattern for Cx26 in maintaining the balance between proliferation and differentiation of the epidermis.

Previous studies have found that the polymorphism of GJB2 gene and the high expression of Cx26 are strongly correlated with the pathogenesis of psoriasis. Consistent with the experimental results of our study, the expression of Cx26 in psoriatic plaque was significantly upregulated. Moreover, the high expression of Cx26 would destroy the skin barrier [44] and activate the skin inflammatory response [49]. Our single-gene GSEA also suggested that GJB2 may be involved in the development and progression of psoriasis by regulating immune microenvironment of skin and destroying the skin barrier, which is consistent with the previous finding. Rs72474224 [50] and Rs3751385 [51] in GJB2 were preferentially associated with psoriasis susceptibility of the Chinese Han population.

Octanol and carbenone were predicted to be used as two new GJB2 inhibitors through DGIdb database. Octanol is a kind of saturated fatty alcohol, which also act as a T-type calcium channels (T-channels) inhibitor [52]. At present, there is no report on the application of octanol in the field of biomedicine, but the therapeutic value of carbenoxolone has been proved in various diseases. Carbenoxolone could restrain the growth of colon cancer by blocking the gap junction channel and reducing the transport of glucose [53]. In angiotensin II dependent hypertension, the combination of carbenoxolone and ramipril could significantly inhibit the proliferation and migration of VSMCs [54]. In addition, carbenoxolone would also exert an antiepileptic effect in vivo and vitro by regulating gap junctions between astrocytes [55]. However, the application of carbenoxolone in psoriasis needs to be further studied.

In conclusion, this study indicated that GJB2 could be a key target for the diagnosis and treatment of psoriasis through combined multiomics, and single-gene GSEA revealed that GJB2 might induce psoriasis by regulating body immunity and destroying skin barrier. In addition, reversing the hypomethylation of GJB2 might be a new strategy for the treatment of psoriasis in the future. However, there are still some limitations in this study. For example, samples in GEO database only generally divided into psoriasis or health, which lacks subdivision of disease subtypes and severity, and there is a certain heterogeneity between patients.

## Figures and Tables

**Table 1:** Primer sequence of GJB2 and GAPDH.

Gene name	Primer sequence
*GJB2*	F: ATCCTGGGGGGTGTGAAC R: GCATGGAGAAGCCGTCGT
*GAPDH*	F: CCCATCACCATCTTCCAGG R: CATCACGCCACAGTTTCCC

**Table 2:** The inhibitory drugs of GJB2.

Gene	Drug	Interaction types
*GJB2*	OCTANOL	Inhibitor (inhibitory)
*GJB2*	CARBENOXOLONE	Inhibitor (inhibitory)

## Data Availability

The data underlying this study are freely available from the GSE13355 dataset (https://www.ncbi.nlm.nih.gov/geo/query/acc.cgi?acc=GSE13355), the GSE14905 dataset (https://www.ncbi.nlm.nih.gov/geo/query/acc.cgi?acc=GSE14905), and the GSE73894 dataset (https://www.ncbi.nlm.nih.gov/geo/query/acc.cgi?acc=GSE73894).

## Supplementary Materials

Supplementary Materials Supplementary Figure 1 The PCA of gene expression in psoriasis lesions and healthy controls in GSE13355 database. Supplementary Figure 2 The PCA and methylation distribution density in psoriasis lesions and healthy controls from the GSE73894 dataset. (A) PCA in GSE73894. (B) Methylation distribution density in GSE73894. Supplementary Table 1 Identification of DEGs in the psoriatic lesions and healthy control group in GSE13355. Supplementary Table 2 GO analysis on 767 DEGs in GSE13355. Supplementary Table 3 KEGG analysis on 767 DEGs in GSE13355. Supplementary Table 4 Identification of hyper-MR-genes. Supplementary Table 5 Identification of hypo-MR-genes. Supplementary Table 6 GO analysis of hyper-MR-genes. Supplementary Table 7 GO analysis of hypo-MR-genes. Supplementary Table 8 KEGG analysis of hyper-MR-genes. Supplementary Table 9 KEGG analysis of hypo-MR-genes. Supplementary Table 10 GO analysis through single-gene GSEA of GJB2. Supplementary Table 11 KEGG analysis through single-gene GSEA of GJB2.
