# Supplementary material for: Exploration of Biomarkers of Psoriasis through Combined Multiomics Analysis
Source: Mediators Inflamm. 2022 Sep 23;2022:7731082. doi: 10.1155/2022/7731082 (PMC9525798; doi:10.1155/2022/7731082)
Supplement: Supplementary Materials — Supplementary Figure 1 The PCA of gene expression in psoriasis lesions and healthy controls in GSE13355 database. Supplementary Figure 2 The PCA and methylation distribution density in psoriasis lesions and healthy controls from the GSE73894 dataset. (A) PCA in GSE73894. (B) Methylation distribution density in GSE73894. Supplementary Table 1 Identification of DEGs in the psoriatic lesions and healthy control group in GSE13355. Supplementary Table 2 GO analysis on 767 DEGs in GSE13355. Supplementary Table 3 KEGG analysis on 767 DEGs in GSE13355. Supplementary Table 4 Identification of hyper-MR-genes. Supplementary Table 5 Identification of hypo-MR-genes. Supplementary Table 6 GO analysis of hyper-MR-genes. Supplementary Table 7 GO analysis of hypo-MR-genes. Supplementary Table 8 KEGG analysis of hyper-MR-genes. Supplementary Table 9 KEGG analysis of hypo-MR-genes. Supplementary Table 10 GO analysis through single-gene GSEA of GJB2. Supplementary Table 11 KEGG analysis through single-gene GSEA of GJB2. [file 7731082.f1.zip › Supplementary Table 1 (1).docx]

| Identification of DEGs in psoriatic lesions and healthy control group in GSE13355 | | | | | | |
| --- | --- | --- | --- | --- | --- | --- |
|  | logFC | AveExpr | t | PValue | adj.P.Val | B |
| WIF1 | -3.80422307 | 7.794006386 | -20.25917187 | 5.09E-41 | 5.06E-39 | 82.86460965 |
| BTC | -3.790678485 | 6.594479975 | -26.859932 | 2.54E-53 | 1.15E-50 | 111.1146476 |
| THRSP | -3.249820583 | 9.547593861 | -10.67851641 | 3.06E-19 | 3.06E-18 | 32.78371226 |
| CCL27 | -2.934702844 | 8.514926129 | -18.99262887 | 2.13E-38 | 1.61E-36 | 76.83519391 |
| KRT77 | -2.929341699 | 10.21988455 | -23.82444927 | 6.17E-48 | 1.33E-45 | 98.75958 |
| PM20D1 | -2.830195527 | 7.729666615 | -7.989715049 | 8.22E-13 | 4.36E-12 | 18.065245 |
| ELOVL3 | -2.735325093 | 7.595222311 | -8.965676734 | 4.14E-15 | 2.74E-14 | 23.31515005 |
| GAL | -2.728196934 | 8.101170789 | -8.545291661 | 4.12E-14 | 2.48E-13 | 21.03464488 |
| ACSBG1 | -2.559244885 | 7.974564535 | -9.250038195 | 8.66E-16 | 6.12E-15 | 24.87079135 |
| C5orf46 | -2.4937686 | 9.915738554 | -23.49674914 | 2.50E-47 | 5.17E-45 | 97.36301058 |
| FABP7 | -2.415915195 | 9.659164202 | -9.191963145 | 1.19E-15 | 8.31E-15 | 24.55233588 |
| MSMB | -2.38265122 | 6.589766715 | -14.88704895 | 2.64E-29 | 7.35E-28 | 55.91310271 |
| HSD11B1 | -2.31890591 | 8.2962621 | -16.18491377 | 2.83E-32 | 1.10E-30 | 62.74571503 |
| FAR2 | -2.208160809 | 8.163864945 | -8.51123084 | 4.96E-14 | 2.96E-13 | 20.8510288 |
| IL37 | -2.201010915 | 9.436840013 | -21.60466008 | 1.04E-43 | 1.38E-41 | 89.04722204 |
| WDR72 | -2.071880551 | 7.274300388 | -14.54461233 | 1.65E-28 | 4.20E-27 | 54.079961 |
| HS3ST6 | -2.070703235 | 7.609577705 | -17.22543063 | 1.36E-34 | 6.71E-33 | 68.08150685 |
| HSD3B1 | -2.0258013 | 5.925484305 | -7.972955827 | 8.99E-13 | 4.76E-12 | 17.97659573 |
| SERPINA12 | -2.012768937 | 10.2682184 | -13.08480105 | 4.68E-25 | 8.47E-24 | 46.14284111 |
| TLCD4 | -2.009256864 | 6.981446508 | -9.905723652 | 2.28E-17 | 1.88E-16 | 28.48769973 |
| PIP | -2.004878168 | 11.95682707 | -8.904150825 | 5.81E-15 | 3.79E-14 | 22.97984974 |
| TPPP | -1.989706016 | 7.825638705 | -16.5655973 | 3.96E-33 | 1.68E-31 | 64.71305699 |
| STXBP6 | -1.984615042 | 6.789356082 | -18.23898255 | 8.52E-37 | 5.27E-35 | 73.1503824 |
| CHP2 | -1.976188689 | 9.516379387 | -19.10935654 | 1.21E-38 | 9.43E-37 | 77.39943782 |
| TSPAN8 | -1.972414975 | 10.04229477 | -12.24164858 | 4.96E-23 | 7.24E-22 | 41.48604776 |
| KRT79 | -1.918371321 | 7.648609942 | -7.689638932 | 4.05E-12 | 2.00E-11 | 16.48727002 |
| MUC7 | -1.912706553 | 5.705564883 | -10.56822724 | 5.66E-19 | 5.50E-18 | 32.16935872 |
| EMX2 | -1.911455881 | 7.299208524 | -19.04286623 | 1.67E-38 | 1.28E-36 | 77.07824685 |
| SCGB2A1 | -1.90612606 | 6.558382918 | -10.21416342 | 4.09E-18 | 3.63E-17 | 30.1994257 |
| MACROD2 | -1.882937854 | 7.172787402 | -22.94136818 | 2.76E-46 | 5.15E-44 | 94.96703178 |
| CLDN8 | -1.812442127 | 7.642881615 | -13.92081574 | 4.82E-27 | 1.04E-25 | 50.7110865 |
| FADS1 | -1.803885603 | 8.661813458 | -8.981789571 | 3.79E-15 | 2.52E-14 | 23.40304067 |
| F3 | -1.801988519 | 8.52905684 | -20.04676548 | 1.38E-40 | 1.30E-38 | 81.86770127 |
| ZBTB16 | -1.79586883 | 7.44363256 | -11.50153414 | 3.07E-21 | 3.74E-20 | 37.37093657 |
| WNT2B | -1.757999294 | 7.032180672 | -18.39152136 | 4.01E-37 | 2.58E-35 | 73.90202512 |
| GAN | -1.730503675 | 9.927028844 | -19.9113648 | 2.62E-40 | 2.35E-38 | 81.22921845 |
| LEPR | -1.730398716 | 9.950282327 | -11.203404 | 1.62E-20 | 1.85E-19 | 35.70948542 |
| GREM1 | -1.728303903 | 8.159419293 | -11.12196346 | 2.56E-20 | 2.85E-19 | 35.25547734 |
| CLDN23 | -1.693807365 | 6.443502787 | -18.09692352 | 1.72E-36 | 1.03E-34 | 72.44772568 |
| AQP9 | -1.693760277 | 7.512201512 | -16.20700448 | 2.53E-32 | 9.87E-31 | 62.86034859 |
| LRRC17 | -1.68149958 | 7.301337133 | -15.64826075 | 4.68E-31 | 1.56E-29 | 59.9434458 |
| CDHR1 | -1.668454642 | 8.630668316 | -17.15896029 | 1.90E-34 | 9.22E-33 | 67.74459599 |
| FA2H | -1.668287577 | 7.187244202 | -9.623515008 | 1.10E-16 | 8.47E-16 | 26.92665805 |
| C1QTNF7 | -1.632347973 | 6.336561984 | -14.26247087 | 7.57E-28 | 1.77E-26 | 52.5607932 |
| FADS2 | -1.620125736 | 7.894144473 | -8.975522133 | 3.93E-15 | 2.60E-14 | 23.36884982 |
| AGTR1 | -1.619802314 | 9.22058802 | -10.9540535 | 6.55E-20 | 7.01E-19 | 34.31939556 |
| SOX5 | -1.60117674 | 7.540268844 | -18.58289862 | 1.57E-37 | 1.06E-35 | 74.84086442 |
| CMAHP | -1.575463302 | 6.642531873 | -15.02447046 | 1.27E-29 | 3.65E-28 | 56.64533748 |
| IGFL2 | -1.571564931 | 8.458711574 | -13.31301007 | 1.33E-25 | 2.52E-24 | 47.39531619 |
| LGR5 | -1.570591964 | 7.554761635 | -7.361295741 | 2.26E-11 | 1.04E-10 | 14.78526151 |
| SLC46A2 | -1.567578675 | 7.381873007 | -12.13356578 | 9.05E-23 | 1.28E-21 | 40.88633804 |
| ZNF273 | -1.565177051 | 8.346284998 | -18.89637168 | 3.40E-38 | 2.51E-36 | 76.36859326 |
| WAKMAR2 | -1.56344687 | 6.967218902 | -10.40666743 | 1.40E-18 | 1.30E-17 | 31.26995736 |
| OSR2 | -1.560761993 | 8.277651774 | -13.79466151 | 9.58E-27 | 2.02E-25 | 50.02542867 |
| SLC1A6 | -1.56054885 | 7.110666967 | -8.41958561 | 8.14E-14 | 4.74E-13 | 20.35793235 |
| TMEM255A | -1.552155195 | 5.766022439 | -13.44491776 | 6.47E-26 | 1.26E-24 | 48.11747405 |
| CST6 | -1.55135667 | 12.40474132 | -13.02867243 | 6.37E-25 | 1.14E-23 | 45.8342174 |
| GREM2 | -1.546440861 | 6.309657672 | -14.16887997 | 1.26E-27 | 2.88E-26 | 52.05517047 |
| CRAT | -1.54462665 | 9.056831663 | -8.819267767 | 9.24E-15 | 5.93E-14 | 22.51807796 |
| PTPN21 | -1.529943939 | 8.628882003 | -19.39513026 | 3.06E-39 | 2.50E-37 | 78.77348494 |
| NR3C2 | -1.525405039 | 7.27121406 | -17.1938098 | 1.60E-34 | 7.80E-33 | 67.92130198 |
| FLG2 | -1.520966359 | 12.8709811 | -10.82780974 | 1.33E-19 | 1.38E-18 | 33.61567893 |
| DES | -1.515371531 | 8.308353519 | -11.75844905 | 7.31E-22 | 9.54E-21 | 38.80138491 |
| CD207 | -1.512739323 | 8.584755742 | -11.3590122 | 6.80E-21 | 8.02E-20 | 36.57681883 |
| SCD5 | -1.512128973 | 7.150620998 | -18.77010638 | 6.28E-38 | 4.40E-36 | 75.75473847 |
| SORBS1 | -1.503448043 | 9.396540405 | -12.9034742 | 1.27E-24 | 2.19E-23 | 45.1450363 |
| RGMB | -1.50172529 | 8.9035425 | -12.41888176 | 1.86E-23 | 2.83E-22 | 42.46826043 |
| C14orf132 | -1.498960293 | 9.113795441 | -17.17544678 | 1.75E-34 | 8.52E-33 | 67.82821016 |
| UST | -1.492906014 | 7.923836277 | -13.74384907 | 1.26E-26 | 2.63E-25 | 49.74886804 |
| SCGB1D2 | -1.48926957 | 10.48299348 | -6.676523025 | 7.47E-10 | 3.00E-09 | 11.33391567 |
| ZSCAN18 | -1.474094318 | 8.472359627 | -16.70482927 | 1.93E-33 | 8.50E-32 | 65.42825628 |
| CILP | -1.464722927 | 9.94369878 | -9.243137846 | 9.00E-16 | 6.34E-15 | 24.83293428 |
| MFSD4A | -1.463759158 | 7.621268967 | -12.45720571 | 1.50E-23 | 2.31E-22 | 42.68044083 |
| CA6 | -1.463696733 | 9.564738683 | -8.68130968 | 1.96E-14 | 1.22E-13 | 21.76971309 |
| GPRASP1 | -1.462918145 | 6.816945484 | -18.86322775 | 3.99E-38 | 2.88E-36 | 76.20765661 |
| SPRR4 | -1.44280286 | 9.631126968 | -8.40499327 | 8.81E-14 | 5.12E-13 | 20.27954893 |
| OGN | -1.438697674 | 7.791980853 | -7.109456379 | 8.31E-11 | 3.64E-10 | 13.49939976 |
| PDK4 | -1.433489092 | 8.357573268 | -8.048641497 | 6.00E-13 | 3.22E-12 | 18.37740418 |
| MAMDC2 | -1.432911605 | 9.075031713 | -10.70730657 | 2.60E-19 | 2.63E-18 | 32.94412434 |
| ID4 | -1.430334608 | 11.41964373 | -22.18213684 | 7.82E-45 | 1.24E-42 | 91.63149905 |
| ELMOD1 | -1.425281752 | 9.234673598 | -9.322188781 | 5.81E-16 | 4.21E-15 | 25.26692393 |
| BHLHE41 | -1.420079 | 8.531961782 | -13.26280124 | 1.76E-25 | 3.27E-24 | 47.12008614 |
| CAB39L | -1.414761996 | 8.620255776 | -12.07583948 | 1.25E-22 | 1.73E-21 | 40.56583074 |
| ENPP5 | -1.409751332 | 5.517596803 | -13.6895579 | 1.70E-26 | 3.49E-25 | 49.45312969 |
| SCEL | -1.398448425 | 11.08155575 | -14.36234959 | 4.42E-28 | 1.07E-26 | 53.09946665 |
| POSTN | -1.390445302 | 12.06402308 | -14.16697463 | 1.27E-27 | 2.90E-26 | 52.04486835 |
| ACTG2 | -1.381759711 | 10.4873551 | -11.54347375 | 2.43E-21 | 2.99E-20 | 37.60455135 |
| ANKRD33B | -1.375361231 | 7.66100068 | -20.87164945 | 2.96E-42 | 3.24E-40 | 85.70721304 |
| ABHD12B | -1.375078994 | 7.554576984 | -10.06048115 | 9.64E-18 | 8.26E-17 | 29.34592249 |
| SLITRK6 | -1.369624656 | 7.691471623 | -11.13982114 | 2.32E-20 | 2.60E-19 | 35.35503133 |
| RBP4 | -1.36234647 | 7.006106674 | -7.666418212 | 4.57E-12 | 2.25E-11 | 16.36601995 |
| ZNF91 | -1.35750986 | 9.189913785 | -13.48812523 | 5.11E-26 | 1.01E-24 | 48.3537262 |
| SNTB1 | -1.356126892 | 6.736470142 | -21.7958433 | 4.40E-44 | 6.12E-42 | 89.90735043 |
| RHOBTB3 | -1.352466735 | 8.984747392 | -15.60876974 | 5.76E-31 | 1.90E-29 | 59.73592829 |
| HAO2 | -1.350838034 | 6.424465201 | -7.713216905 | 3.57E-12 | 1.78E-11 | 16.61051655 |
| PTN | -1.350179091 | 8.590362279 | -9.853840764 | 3.05E-17 | 2.47E-16 | 28.20029798 |
| ARFGEF3 | -1.350108238 | 7.767492467 | -16.05817924 | 5.48E-32 | 2.03E-30 | 62.08695294 |
| TMEM116 | -1.345679201 | 7.252253297 | -17.44341258 | 4.52E-35 | 2.32E-33 | 69.18252146 |
| AFF3 | -1.343388779 | 6.394105902 | -12.19583768 | 6.40E-23 | 9.22E-22 | 41.2319242 |
| FAM189A2 | -1.341637048 | 7.262395215 | -16.31720913 | 1.43E-32 | 5.76E-31 | 63.43136301 |
| COBL | -1.34125255 | 8.622603838 | -21.27862211 | 4.57E-43 | 5.45E-41 | 87.56990496 |
| RAI2 | -1.337619279 | 8.336550687 | -13.71038261 | 1.52E-26 | 3.13E-25 | 49.566597 |
| PAMR1 | -1.332177221 | 8.709602921 | -12.00257082 | 1.88E-22 | 2.57E-21 | 40.15883542 |
| AGR3 | -1.332034608 | 5.05318427 | -8.626238455 | 2.65E-14 | 1.63E-13 | 21.47175401 |
| PCP4 | -1.32949286 | 8.105557225 | -10.22123983 | 3.93E-18 | 3.50E-17 | 30.23875218 |
| FGFBP2 | -1.326594583 | 8.101320994 | -8.745353812 | 1.38E-14 | 8.74E-14 | 22.11678625 |
| SEPTIN7P13 | -1.325136245 | 6.142612664 | -11.11323346 | 2.69E-20 | 2.99E-19 | 35.20680853 |
| CLMP | -1.324461628 | 8.930274458 | -11.26302584 | 1.16E-20 | 1.34E-19 | 36.04183468 |
| GLDC | -1.323479519 | 6.733440047 | -6.634074257 | 9.24E-10 | 3.68E-09 | 11.1249253 |
| PLLP | -1.317056222 | 8.901137038 | -16.29321898 | 1.62E-32 | 6.43E-31 | 63.30718295 |
| PPARGC1A | -1.307258096 | 7.484311759 | -12.6547508 | 5.02E-24 | 8.25E-23 | 43.77287604 |
| CGNL1 | -1.305151495 | 9.659148763 | -14.98344708 | 1.58E-29 | 4.48E-28 | 56.42695775 |
| NTM | -1.304949447 | 7.501139041 | -14.93745973 | 2.02E-29 | 5.68E-28 | 56.18194172 |
| SCN7A | -1.304913844 | 5.960433295 | -10.9445663 | 6.90E-20 | 7.37E-19 | 34.26650743 |
| ZNF677 | -1.302689651 | 7.402738402 | -15.3677962 | 2.05E-30 | 6.30E-29 | 58.46587017 |
| PEG3 | -1.298938269 | 6.199459297 | -14.14405859 | 1.44E-27 | 3.24E-26 | 51.92093552 |
| GATA3 | -1.296490215 | 11.79167418 | -18.06299111 | 2.04E-36 | 1.21E-34 | 72.27951006 |
| CNKSR2 | -1.295847722 | 5.564262934 | -11.5801749 | 1.98E-21 | 2.46E-20 | 37.80895671 |
| KRT15 | -1.293961405 | 11.51413981 | -10.68626539 | 2.93E-19 | 2.93E-18 | 32.82688633 |
| LYVE1 | -1.291190002 | 9.088466548 | -7.504580307 | 1.07E-11 | 5.09E-11 | 15.52462081 |
| ARHGEF26 | -1.287954919 | 8.222884516 | -19.43961859 | 2.47E-39 | 2.05E-37 | 78.98645461 |
| GALNT16 | -1.28762345 | 8.373815631 | -10.05713468 | 9.82E-18 | 8.40E-17 | 29.32735024 |
| CHL1 | -1.283721903 | 8.101691635 | -10.22285172 | 3.90E-18 | 3.47E-17 | 30.24771044 |
| PAPLN | -1.275799369 | 6.959174823 | -10.93784233 | 7.17E-20 | 7.63E-19 | 34.22902374 |
| NOVA1 | -1.27516367 | 7.743231587 | -10.11598653 | 7.07E-18 | 6.13E-17 | 29.65405037 |
| ADH1B | -1.272667527 | 10.69850038 | -9.045859152 | 2.67E-15 | 1.79E-14 | 23.7528393 |
| PLCB4 | -1.272462689 | 7.042091173 | -10.86684971 | 1.07E-19 | 1.12E-18 | 33.83328401 |
| EEF2K | -1.270909298 | 10.32353701 | -18.35340803 | 4.84E-37 | 3.05E-35 | 73.71449661 |
| SOX6 | -1.270485633 | 7.197812352 | -14.07844888 | 2.05E-27 | 4.57E-26 | 51.56584054 |
| CD34 | -1.270029899 | 8.910617692 | -10.69924295 | 2.72E-19 | 2.74E-18 | 32.89919426 |
| MAML2 | -1.267745736 | 9.153993926 | -16.59170623 | 3.46E-33 | 1.48E-31 | 64.84735032 |
| CLDN1 | -1.267328862 | 11.29013516 | -22.7130095 | 7.50E-46 | 1.30E-43 | 93.97113619 |
| GXYLT2 | -1.266507295 | 9.259153287 | -9.188640741 | 1.22E-15 | 8.45E-15 | 24.53412847 |
| LAMB4 | -1.264218403 | 6.591601133 | -12.87327129 | 1.50E-24 | 2.56E-23 | 44.97862131 |
| SERTM1 | -1.260061001 | 5.070588434 | -8.422803374 | 8.00E-14 | 4.66E-13 | 20.37522161 |
| CYP4B1 | -1.25668907 | 7.152155844 | -7.102666146 | 8.61E-11 | 3.76E-10 | 13.46498285 |
| CORO2B | -1.255626072 | 6.255329607 | -14.52996601 | 1.79E-28 | 4.53E-27 | 54.00129119 |
| IGDCC4 | -1.255146859 | 7.038569369 | -9.538646557 | 1.75E-16 | 1.33E-15 | 26.45838264 |
| HOXC10 | -1.253780005 | 7.960150943 | -11.48610013 | 3.35E-21 | 4.06E-20 | 37.28495639 |
| GUCY1A2 | -1.250438908 | 7.206974934 | -10.1002667 | 7.72E-18 | 6.67E-17 | 29.56676884 |
| RAI14 | -1.247085486 | 8.767003099 | -21.52420188 | 1.50E-43 | 1.93E-41 | 88.68388654 |
| DDAH1 | -1.240118537 | 9.301837376 | -18.35500786 | 4.80E-37 | 3.04E-35 | 73.72237197 |
| APOC1 | -1.238105548 | 8.389104376 | -7.740197806 | 3.10E-12 | 1.56E-11 | 16.75171116 |
| CYP39A1 | -1.236854476 | 7.535711249 | -10.91341008 | 8.22E-20 | 8.70E-19 | 34.09282502 |
| GSTA3 | -1.235373825 | 7.66389735 | -15.40326936 | 1.70E-30 | 5.30E-29 | 58.65323817 |
| AR | -1.232012385 | 8.537380871 | -13.44128264 | 6.60E-26 | 1.29E-24 | 48.09759098 |
| PGM5 | -1.226403901 | 8.54886952 | -14.32487161 | 5.41E-28 | 1.29E-26 | 52.89744982 |
| APOD | -1.223837742 | 11.41084625 | -7.430623006 | 1.58E-11 | 7.37E-11 | 15.14232063 |
| NRN1 | -1.22063498 | 8.988068398 | -15.23075645 | 4.24E-30 | 1.28E-28 | 57.74073128 |
| FIBIN | -1.219343344 | 8.661080658 | -11.13313399 | 2.41E-20 | 2.69E-19 | 35.31775154 |
| NAP1L3 | -1.218054621 | 7.110606711 | -9.629169692 | 1.06E-16 | 8.22E-16 | 26.95787953 |
| HINT3 | -1.216190324 | 6.860242689 | -11.86472851 | 4.04E-22 | 5.38E-21 | 39.39260189 |
| MGST1 | -1.21418638 | 11.08068618 | -8.239881858 | 2.15E-13 | 1.20E-12 | 19.39525933 |
| ADRB2 | -1.213990366 | 7.428067194 | -15.46701483 | 1.22E-30 | 3.85E-29 | 58.98958919 |
| CACNA2D1 | -1.213216615 | 7.999187607 | -13.4745743 | 5.50E-26 | 1.08E-24 | 48.27964772 |
| ADGRL3 | -1.212176268 | 7.634106608 | -12.5921107 | 7.10E-24 | 1.14E-22 | 43.42671094 |
| SRGAP2B | -1.211359269 | 8.945896623 | -15.48072465 | 1.13E-30 | 3.60E-29 | 59.06186958 |
| TNXB | -1.210124223 | 9.944564518 | -8.953453107 | 4.43E-15 | 2.92E-14 | 23.24849576 |
| RORA | -1.209927268 | 11.72510702 | -16.96185651 | 5.19E-34 | 2.42E-32 | 66.74235298 |
| SYT8 | -1.208002668 | 7.286811025 | -16.16175469 | 3.20E-32 | 1.23E-30 | 62.62547571 |
| SCARA5 | -1.207577188 | 10.82492311 | -9.850871268 | 3.10E-17 | 2.51E-16 | 28.18385387 |
| PRLR | -1.204260117 | 6.165922098 | -15.57118964 | 7.02E-31 | 2.28E-29 | 59.53828889 |
| MYH11 | -1.202886202 | 11.23457141 | -13.29005078 | 1.51E-25 | 2.84E-24 | 47.26948369 |
| MYOCD | -1.202863488 | 7.435194648 | -7.847102351 | 1.76E-12 | 9.05E-12 | 17.31280274 |
| LMOD1 | -1.202758451 | 8.32830239 | -12.37032588 | 2.43E-23 | 3.66E-22 | 42.19932194 |
| TPM2 | -1.199087875 | 9.893345355 | -11.08808888 | 3.09E-20 | 3.42E-19 | 35.06662921 |
| ANG | -1.198605318 | 7.219984238 | -18.13497324 | 1.42E-36 | 8.57E-35 | 72.63617899 |
| SSPN | -1.198573283 | 8.551348701 | -8.068642825 | 5.39E-13 | 2.90E-12 | 18.4835215 |
| OMD | -1.196741684 | 7.912072304 | -7.784230896 | 2.45E-12 | 1.25E-11 | 16.9825049 |
| DKK2 | -1.196350821 | 8.078146851 | -9.213015471 | 1.06E-15 | 7.43E-15 | 24.66773468 |
| PPP1R1B | -1.195643958 | 7.969283182 | -9.394250474 | 3.90E-16 | 2.87E-15 | 25.66308958 |
| ADIPOQ | -1.194448524 | 8.412067105 | -5.811530977 | 4.97E-08 | 1.66E-07 | 7.210611603 |
| MIR100HG | -1.193959146 | 8.272760174 | -10.52941523 | 7.03E-19 | 6.79E-18 | 31.95322694 |
| PRRG3 | -1.193084428 | 6.837409287 | -9.767847106 | 4.92E-17 | 3.94E-16 | 27.72433136 |
| TMPRSS11E | -1.192577975 | 6.166038729 | -6.997034599 | 1.48E-10 | 6.33E-10 | 12.93136903 |
| ITM2A | -1.192554214 | 10.60123434 | -12.08864964 | 1.16E-22 | 1.62E-21 | 40.63696709 |
| PLEKHH1 | -1.191828714 | 7.465679998 | -12.67304062 | 4.54E-24 | 7.50E-23 | 43.87390698 |
| EPCAM | -1.189971785 | 8.470809461 | -9.406353836 | 3.65E-16 | 2.69E-15 | 25.72967812 |
| MTURN | -1.188506622 | 8.343736707 | -15.69195257 | 3.72E-31 | 1.26E-29 | 60.17283191 |
| ZNF667-AS1 | -1.186859091 | 7.496711139 | -12.71859037 | 3.53E-24 | 5.88E-23 | 44.12543197 |
| MCOLN3 | -1.183929883 | 7.638040057 | -9.952589783 | 1.76E-17 | 1.46E-16 | 28.74745464 |
| MYL9 | -1.183838762 | 9.834023365 | -7.731125562 | 3.25E-12 | 1.63E-11 | 16.70421593 |
| DMD | -1.179120522 | 8.527410991 | -13.73077907 | 1.36E-26 | 2.81E-25 | 49.67769522 |
| PDGFRL | -1.178445099 | 8.850857256 | -8.797186027 | 1.04E-14 | 6.65E-14 | 22.39811205 |
| CCDC18-AS1 | -1.175031748 | 7.938295601 | -15.18192474 | 5.50E-30 | 1.63E-28 | 57.48184558 |
| HMGCS2 | -1.173744319 | 7.598111468 | -11.05265041 | 3.77E-20 | 4.14E-19 | 34.86906101 |
| PSAPL1 | -1.169868461 | 9.626048767 | -8.426981141 | 7.82E-14 | 4.56E-13 | 20.39767167 |
| PRUNE2 | -1.169683046 | 7.48955429 | -13.09052979 | 4.53E-25 | 8.22E-24 | 46.17432821 |
| LOC101927164 | -1.168597599 | 8.478110214 | -12.01778952 | 1.72E-22 | 2.36E-21 | 40.24339005 |
| CRIP1 | -1.160546614 | 10.95688496 | -13.77864088 | 1.05E-26 | 2.19E-25 | 49.93825598 |
| MEOX2 | -1.159744188 | 7.277922313 | -10.77341714 | 1.80E-19 | 1.85E-18 | 33.31252831 |
| CRYAB | -1.158785215 | 10.63574229 | -15.84056589 | 1.71E-31 | 6.00E-30 | 60.95143729 |
| ESR1 | -1.158185505 | 7.050216953 | -9.720215495 | 6.41E-17 | 5.07E-16 | 27.46091643 |
| RETREG1 | -1.156968395 | 8.439832862 | -15.29802518 | 2.97E-30 | 9.03E-29 | 58.09693746 |
| HLA-DQB2 | -1.15629662 | 9.333783576 | -8.390586348 | 9.53E-14 | 5.51E-13 | 20.20219738 |
| IGFBP6 | -1.154667728 | 10.02575025 | -8.892853564 | 6.18E-15 | 4.02E-14 | 22.91833602 |
| CCN5 | -1.152527295 | 11.13560526 | -9.100205419 | 1.98E-15 | 1.35E-14 | 24.04994006 |
| PKIB | -1.150890093 | 8.059593408 | -11.03107276 | 4.26E-20 | 4.64E-19 | 34.74876647 |
| SYNPO2 | -1.147773065 | 10.13407931 | -12.43058078 | 1.74E-23 | 2.67E-22 | 42.53304016 |
| RNASE4 | -1.145230422 | 10.11254577 | -13.74072799 | 1.29E-26 | 2.67E-25 | 49.7318735 |
| DNM1 | -1.144494427 | 8.054957135 | -10.00485002 | 1.31E-17 | 1.11E-16 | 29.03725855 |
| HPGDS | -1.143087074 | 7.847668323 | -10.92556658 | 7.68E-20 | 8.15E-19 | 34.1605915 |
| FNDC1 | -1.140396329 | 6.0327873 | -9.018515405 | 3.10E-15 | 2.07E-14 | 23.60348992 |
| DCLK1 | -1.13783951 | 9.838645811 | -9.140957996 | 1.58E-15 | 1.08E-14 | 24.27295278 |
| SYBU | -1.136247382 | 7.842343132 | -15.51197967 | 9.59E-31 | 3.06E-29 | 59.22657271 |
| SYNM | -1.12876798 | 9.782249338 | -9.686670154 | 7.72E-17 | 6.07E-16 | 27.27550175 |
| NCALD | -1.128583559 | 8.648286796 | -9.452710732 | 2.82E-16 | 2.11E-15 | 25.98484471 |
| ACADL | -1.127822442 | 5.880044353 | -18.52203585 | 2.11E-37 | 1.38E-35 | 74.54279516 |
| ZNF423 | -1.127524665 | 9.34391732 | -13.05284546 | 5.58E-25 | 1.01E-23 | 45.96715996 |
| FAM171B | -1.126820245 | 7.472398041 | -11.73713293 | 8.24E-22 | 1.07E-20 | 38.68276541 |
| PLIN1 | -1.12678947 | 8.822824714 | -7.103131467 | 8.59E-11 | 3.75E-10 | 13.46734093 |
| CAMK2N1 | -1.125401773 | 9.23635728 | -10.76976984 | 1.83E-19 | 1.89E-18 | 33.2922019 |
| LOC100506990 | -1.124586589 | 9.681042615 | -13.69908679 | 1.61E-26 | 3.32E-25 | 49.50505419 |
| EFNB2 | -1.121114488 | 10.12564331 | -18.45573959 | 2.93E-37 | 1.90E-35 | 74.21757971 |
| TSPYL5 | -1.120109559 | 8.107605403 | -14.21468531 | 9.80E-28 | 2.28E-26 | 52.30273596 |
| PCDH7 | -1.120108108 | 8.706739213 | -12.24350685 | 4.91E-23 | 7.18E-22 | 41.49635398 |
| FOS | -1.119832746 | 8.560254073 | -7.296503098 | 3.17E-11 | 1.44E-10 | 14.45273199 |
| PELI2 | -1.113645381 | 8.069853067 | -14.63110477 | 1.04E-28 | 2.70E-27 | 54.54410076 |
| HOXC6 | -1.112757027 | 9.865778454 | -15.08242202 | 9.33E-30 | 2.72E-28 | 56.95352602 |
| ACKR4 | -1.109189691 | 7.832571596 | -8.395472867 | 9.28E-14 | 5.38E-13 | 20.22842935 |
| RHOB | -1.108167681 | 10.7101175 | -10.02108626 | 1.20E-17 | 1.02E-16 | 29.12732643 |
| EDIL3 | -1.107899449 | 9.346870453 | -14.80305972 | 4.14E-29 | 1.12E-27 | 55.46460198 |
| MAP1B | -1.107120482 | 10.29218623 | -14.04861738 | 2.41E-27 | 5.35E-26 | 51.4042544 |
| RHPN2 | -1.105604135 | 7.343755779 | -7.517248724 | 1.00E-11 | 4.78E-11 | 15.59024794 |
| SCGB2A2 | -1.103251856 | 12.53787286 | -7.794663866 | 2.32E-12 | 1.18E-11 | 17.03725313 |
| BEX5 | -1.101318997 | 7.331075148 | -13.25771594 | 1.81E-25 | 3.35E-24 | 47.09219939 |
| PDZK1 | -1.098938634 | 5.566586928 | -6.327740904 | 4.20E-09 | 1.57E-08 | 9.635974008 |
| CNN1 | -1.098434581 | 9.001522776 | -11.40871162 | 5.15E-21 | 6.16E-20 | 36.85377546 |
| ADCY2 | -1.098147653 | 7.928496245 | -16.1428702 | 3.53E-32 | 1.35E-30 | 62.52738288 |
| PDGFD | -1.096536553 | 8.628243828 | -8.20731684 | 2.56E-13 | 1.42E-12 | 19.22143734 |
| NEGR1 | -1.095884731 | 8.546144885 | -8.877775015 | 6.71E-15 | 4.36E-14 | 22.83625942 |
| ACOX2 | -1.095368718 | 7.38191481 | -11.41454845 | 4.99E-21 | 5.97E-20 | 36.88629962 |
| SMAD9 | -1.09295688 | 7.534243705 | -10.2824148 | 2.80E-18 | 2.52E-17 | 30.5788141 |
| ELN | -1.092357415 | 8.865399987 | -6.45578375 | 2.24E-09 | 8.60E-09 | 10.25412583 |
| ZNF573 | -1.090450707 | 6.667057416 | -10.06646137 | 9.32E-18 | 7.99E-17 | 29.37911303 |
| ATP1A2 | -1.088656959 | 7.310539103 | -11.77488661 | 6.67E-22 | 8.73E-21 | 38.89284731 |
| SLC29A1 | -1.087042174 | 8.34964645 | -9.530225656 | 1.84E-16 | 1.39E-15 | 26.41195183 |
| C8orf88 | -1.084661618 | 6.57200285 | -13.23300963 | 2.07E-25 | 3.84E-24 | 46.95668779 |
| PLA2R1 | -1.084168843 | 6.755430156 | -11.5503824 | 2.34E-21 | 2.89E-20 | 37.64303097 |
| BAG2 | -1.083364321 | 6.805047259 | -7.910295292 | 1.26E-12 | 6.56E-12 | 17.64567496 |
| TPM1 | -1.082406525 | 11.09175821 | -14.66542859 | 8.65E-29 | 2.27E-27 | 54.7280821 |
| ZNF559 | -1.082078183 | 7.609284567 | -14.2867478 | 6.64E-28 | 1.57E-26 | 52.69181324 |
| EN1 | -1.081988906 | 8.12845597 | -8.933439211 | 4.94E-15 | 3.25E-14 | 23.13940304 |
| PNPLA3 | -1.08196877 | 7.365739295 | -10.37625145 | 1.66E-18 | 1.53E-17 | 31.10072066 |
| RBMS3 | -1.078383122 | 9.075672041 | -11.07073207 | 3.41E-20 | 3.75E-19 | 34.9698657 |
| SLC18A2 | -1.076649329 | 6.885508658 | -13.43152353 | 6.97E-26 | 1.35E-24 | 48.04420631 |
| FASN | -1.076283842 | 8.650213385 | -6.685955209 | 7.13E-10 | 2.87E-09 | 11.3804384 |
| PDGFC | -1.073578651 | 10.07401625 | -15.85723195 | 1.56E-31 | 5.52E-30 | 61.0385952 |
| ISM1 | -1.072464411 | 9.11079723 | -9.561064113 | 1.55E-16 | 1.18E-15 | 26.58201704 |
| HOXB3 | -1.071984805 | 8.416895426 | -10.27982898 | 2.84E-18 | 2.56E-17 | 30.56443688 |
| PLN | -1.071825288 | 8.992081566 | -8.31196314 | 1.46E-13 | 8.27E-13 | 19.78070493 |
| RCAN2 | -1.06894425 | 9.553896964 | -12.72553423 | 3.39E-24 | 5.67E-23 | 44.16376484 |
| PCDH20 | -1.068369981 | 5.23021309 | -14.31913038 | 5.58E-28 | 1.33E-26 | 52.86649105 |
| SLC2A13 | -1.068092614 | 6.8443495 | -12.75819503 | 2.83E-24 | 4.77E-23 | 44.34402591 |
| FBLN1 | -1.066384894 | 11.3934324 | -8.753524244 | 1.32E-14 | 8.37E-14 | 22.16110694 |
| FCGBP | -1.066142264 | 8.642615652 | -10.04993445 | 1.02E-17 | 8.72E-17 | 29.2873924 |
| IL17D | -1.063906936 | 7.007884631 | -9.040096706 | 2.75E-15 | 1.85E-14 | 23.72135774 |
| OLFML3 | -1.063441455 | 10.38448927 | -9.707719236 | 6.87E-17 | 5.42E-16 | 27.39183625 |
| SUSD2 | -1.062394943 | 7.370631541 | -12.14710902 | 8.39E-23 | 1.19E-21 | 40.96151239 |
| SPRY2 | -1.060887386 | 8.106644302 | -16.29691642 | 1.58E-32 | 6.32E-31 | 63.32632648 |
| CDON | -1.06024997 | 8.536376361 | -12.50444543 | 1.15E-23 | 1.80E-22 | 42.94187603 |
| LPL | -1.059526761 | 8.593664952 | -7.09768447 | 8.83E-11 | 3.85E-10 | 13.4397415 |
| HOTAIR | -1.058055414 | 5.863384877 | -9.259145774 | 8.24E-16 | 5.84E-15 | 24.92076551 |
| FRZB | -1.05700167 | 8.619813127 | -10.98816955 | 5.41E-20 | 5.84E-19 | 34.50958515 |
| CREB5 | -1.056376311 | 7.178922967 | -8.565730781 | 3.69E-14 | 2.23E-13 | 21.14491802 |
| SDC2 | -1.052482548 | 9.798310961 | -12.57818093 | 7.67E-24 | 1.23E-22 | 43.3497008 |
| TNS1 | -1.050279498 | 10.84117314 | -13.50886834 | 4.56E-26 | 9.04E-25 | 48.46709354 |
| TNMD | -1.047377037 | 6.482497923 | -6.91904986 | 2.20E-10 | 9.28E-10 | 12.53962414 |
| UBE2QL1 | -1.047232269 | 7.527573917 | -7.727341066 | 3.32E-12 | 1.66E-11 | 16.68440891 |
| DPYSL3 | -1.046857574 | 9.697368575 | -11.76157268 | 7.19E-22 | 9.39E-21 | 38.81876611 |
| FAM13A | -1.046620384 | 9.580469945 | -13.01142782 | 7.00E-25 | 1.25E-23 | 45.73935381 |
| APOE | -1.045155284 | 10.10812234 | -8.70711371 | 1.71E-14 | 1.07E-13 | 21.90948015 |
| LORICRIN | -1.044842909 | 13.03689533 | -8.215844119 | 2.45E-13 | 1.36E-12 | 19.26693406 |
| LINC00312 | -1.044145732 | 6.962931526 | -12.15215369 | 8.16E-23 | 1.16E-21 | 40.98951176 |
| TGFBR3 | -1.043526676 | 10.72769913 | -15.04744931 | 1.12E-29 | 3.25E-28 | 56.76758267 |
| IL17RD | -1.042140323 | 8.368939287 | -12.13444003 | 9.01E-23 | 1.27E-21 | 40.89119099 |
| CRISPLD1 | -1.041566308 | 6.464256356 | -8.473554479 | 6.08E-14 | 3.59E-13 | 20.64814217 |
| TCF7L1 | -1.041409317 | 7.759585982 | -12.09718887 | 1.11E-22 | 1.55E-21 | 40.68438269 |
| RNF128 | -1.03711378 | 8.297110297 | -10.47603521 | 9.48E-19 | 8.99E-18 | 31.65603548 |
| DBN1 | -1.035848501 | 8.922210718 | -12.80456695 | 2.19E-24 | 3.70E-23 | 44.59984774 |
| PCDHB16 | -1.034481106 | 6.41522332 | -11.34504911 | 7.36E-21 | 8.65E-20 | 36.49900162 |
| SGCG | -1.032195426 | 7.134722081 | -11.61305322 | 1.65E-21 | 2.07E-20 | 37.99204559 |
| ALCAM | -1.031756813 | 9.809449357 | -16.60250229 | 3.27E-33 | 1.41E-31 | 64.90285666 |
| KRT10-AS1 | -1.031327702 | 8.713220591 | -13.30744724 | 1.38E-25 | 2.60E-24 | 47.36483176 |
| PCOLCE2 | -1.030636066 | 8.60276539 | -5.556547828 | 1.62E-07 | 5.14E-07 | 6.055677436 |
| BCL2 | -1.030044415 | 8.10642375 | -12.58584491 | 7.35E-24 | 1.18E-22 | 43.39207216 |
| TIMP3 | -1.029490542 | 11.57916385 | -12.72307454 | 3.44E-24 | 5.74E-23 | 44.15018669 |
| PIK3C2G | -1.02945507 | 6.994449433 | -13.91367121 | 5.01E-27 | 1.08E-25 | 50.67229289 |
| KRT19 | -1.028162834 | 9.038478059 | -6.387030317 | 3.14E-09 | 1.19E-08 | 9.921434135 |
| PWAR6 | -1.027639184 | 6.129682863 | -12.22696691 | 5.38E-23 | 7.80E-22 | 41.40461558 |
| EMX2OS | -1.026028348 | 7.984955 | -16.22896733 | 2.25E-32 | 8.87E-31 | 62.97426159 |
| KCNMB4 | -1.024997301 | 7.415242256 | -16.54987802 | 4.29E-33 | 1.82E-31 | 64.63216404 |
| ZNF529 | -1.024026184 | 7.593862598 | -12.63819466 | 5.50E-24 | 9.00E-23 | 43.6814048 |
| LRRN4CL | -1.023969122 | 9.384329244 | -10.57697639 | 5.39E-19 | 5.25E-18 | 32.21808506 |
| BCAR3 | -1.022670644 | 6.026841704 | -17.63305143 | 1.74E-35 | 9.36E-34 | 70.13555817 |
| ZNF107 | -1.022568823 | 6.189723199 | -14.31139763 | 5.81E-28 | 1.38E-26 | 52.82478824 |
| SLITRK4 | -1.02250839 | 6.332944189 | -6.524838395 | 1.59E-09 | 6.20E-09 | 10.59003836 |
| ADGRD1 | -1.022450002 | 7.002553697 | -9.331348723 | 5.53E-16 | 4.01E-15 | 25.31725319 |
| C1orf68 | -1.021491722 | 10.1239937 | -7.182341591 | 5.71E-11 | 2.54E-10 | 13.86968076 |
| ADRB1 | -1.019657463 | 5.513387 | -10.02711756 | 1.16E-17 | 9.86E-17 | 29.16078773 |
| CCND1 | -1.019567643 | 9.338577781 | -17.17899712 | 1.72E-34 | 8.39E-33 | 67.846212 |
| MYLK | -1.018308668 | 11.56039099 | -12.00812224 | 1.82E-22 | 2.49E-21 | 40.18967995 |
| MYADM | -1.017562669 | 11.0525754 | -11.64791805 | 1.36E-21 | 1.72E-20 | 38.18616897 |
| FBXO32 | -1.014123741 | 7.851371291 | -9.682567151 | 7.90E-17 | 6.20E-16 | 27.25282907 |
| DIXDC1 | -1.013983158 | 8.549524553 | -13.59227912 | 2.89E-26 | 5.80E-25 | 48.92260666 |
| TFAP2B | -1.012795802 | 7.635853764 | -12.15672288 | 7.96E-23 | 1.13E-21 | 41.01487115 |
| GLDN | -1.012322557 | 9.186874352 | -11.15778391 | 2.10E-20 | 2.36E-19 | 35.45516999 |
| MSRB3 | -1.012314275 | 9.093196517 | -10.28800589 | 2.71E-18 | 2.45E-17 | 30.60990168 |
| AHNAK2 | -1.011698952 | 11.59079864 | -18.42686332 | 3.37E-37 | 2.18E-35 | 74.07575279 |
| CFL2 | -1.010793124 | 8.416757393 | -10.12542374 | 6.71E-18 | 5.83E-17 | 29.70645461 |
| FAM117A | -1.010256981 | 8.623421814 | -17.09951095 | 2.58E-34 | 1.24E-32 | 67.44280912 |
| MYOC | -1.009814409 | 6.58085219 | -11.48477045 | 3.37E-21 | 4.09E-20 | 37.27754878 |
| LINC02754 | -1.009297401 | 5.355308467 | -9.757688481 | 5.20E-17 | 4.15E-16 | 27.66813795 |
| TRIM2 | -1.005078338 | 8.323677219 | -10.67330076 | 3.15E-19 | 3.14E-18 | 32.75465344 |
| C14orf28 | -1.004891071 | 7.285248713 | -14.03088224 | 2.65E-27 | 5.86E-26 | 51.30815117 |
| ZNF83 | -1.004660299 | 7.583833424 | -11.3385508 | 7.63E-21 | 8.96E-20 | 36.46278522 |
| CCDC146 | -1.003399838 | 7.194361506 | -12.08637356 | 1.18E-22 | 1.64E-21 | 40.62432821 |
| FHL1 | -1.00176175 | 12.25792815 | -14.49565137 | 2.15E-28 | 5.41E-27 | 53.8168935 |
| IGFBP5 | -1.000606716 | 11.3196311 | -10.12525025 | 6.72E-18 | 5.84E-17 | 29.70549119 |
| CASP7 | 1.003468853 | 9.042005786 | 17.35172931 | 7.17E-35 | 3.64E-33 | 68.72015418 |
| PSPH | 1.003995086 | 6.305831028 | 11.91831923 | 3.00E-22 | 4.03E-21 | 39.69058021 |
| CD48 | 1.006817641 | 8.58742028 | 12.75275569 | 2.92E-24 | 4.90E-23 | 44.31400975 |
| KIF2A | 1.00691384 | 7.825116896 | 14.95025754 | 1.89E-29 | 5.32E-28 | 56.2501496 |
| TRIM16 | 1.007236047 | 8.9207494 | 13.59768684 | 2.81E-26 | 5.65E-25 | 48.95211903 |
| LY6D | 1.008448217 | 12.18547516 | 16.58769102 | 3.53E-33 | 1.51E-31 | 64.8267031 |
| SLC27A4 | 1.008448771 | 8.568944303 | 13.48993652 | 5.06E-26 | 9.98E-25 | 48.36362684 |
| IFI16 | 1.012012546 | 11.03772136 | 21.63447825 | 9.10E-44 | 1.22E-41 | 89.18167152 |
| SERPINB8 | 1.014189817 | 9.121155717 | 13.69354493 | 1.66E-26 | 3.42E-25 | 49.47485659 |
| PGLYRP4 | 1.016897364 | 7.656009267 | 12.58597555 | 7.35E-24 | 1.18E-22 | 43.39279435 |
| SQLE | 1.01692648 | 10.38616063 | 14.43072499 | 3.05E-28 | 7.59E-27 | 53.46767848 |
| ABHD17C | 1.017998813 | 9.075777071 | 17.03547185 | 3.57E-34 | 1.68E-32 | 67.11723538 |
| CYB5R2 | 1.020967409 | 8.564908419 | 11.56231443 | 2.19E-21 | 2.71E-20 | 37.70948728 |
| TAP1 | 1.023138372 | 9.443380686 | 17.446476 | 4.45E-35 | 2.30E-33 | 69.19795248 |
| CTSC | 1.023302276 | 10.44755895 | 21.805606 | 4.21E-44 | 5.90E-42 | 89.95115109 |
| NUF2 | 1.023775807 | 5.498091013 | 13.25688076 | 1.82E-25 | 3.37E-24 | 47.08761926 |
| NFE2L3 | 1.024436906 | 7.022337778 | 13.81097536 | 8.77E-27 | 1.85E-25 | 50.11417404 |
| CRCT1 | 1.026089845 | 10.43578465 | 9.953523207 | 1.75E-17 | 1.46E-16 | 28.75262946 |
| TUBB6 | 1.026529444 | 11.39969163 | 20.24544854 | 5.43E-41 | 5.37E-39 | 82.80037367 |
| PPARD | 1.027172873 | 9.607016025 | 17.4112833 | 5.31E-35 | 2.72E-33 | 69.0206097 |
| DDX60L | 1.033183557 | 7.489795672 | 10.07333995 | 8.97E-18 | 7.70E-17 | 29.41729176 |
| CCL19 | 1.034430862 | 9.711470981 | 8.80687922 | 9.89E-15 | 6.32E-14 | 22.450765 |
| ESRP2 | 1.034711687 | 9.346726113 | 19.60282604 | 1.13E-39 | 9.65E-38 | 79.76558117 |
| PLA2G2F | 1.035083238 | 5.393620695 | 11.62733858 | 1.52E-21 | 1.92E-20 | 38.0715884 |
| SLC23A2 | 1.035359127 | 7.92955137 | 12.75608417 | 2.87E-24 | 4.82E-23 | 44.33237766 |
| RIPOR3 | 1.038350363 | 6.46358432 | 12.90443721 | 1.26E-24 | 2.18E-23 | 45.1503414 |
| RAET1E | 1.038748423 | 7.425056613 | 11.6478781 | 1.36E-21 | 1.72E-20 | 38.18594653 |
| SPC25 | 1.038812833 | 4.973896279 | 17.82957546 | 6.51E-36 | 3.64E-34 | 71.11844018 |
| MFHAS1 | 1.039603225 | 9.638307796 | 12.36276833 | 2.53E-23 | 3.80E-22 | 42.15745189 |
| IFI30 | 1.039823984 | 10.17855924 | 21.81228853 | 4.09E-44 | 5.76E-42 | 89.98112567 |
| LTB | 1.039971373 | 7.861584925 | 14.72664452 | 6.23E-29 | 1.66E-27 | 55.05591271 |
| RABIF | 1.040915669 | 8.493159044 | 19.952304 | 2.16E-40 | 1.96E-38 | 81.42251351 |
| ODF3B | 1.0411027 | 7.76603023 | 17.27994968 | 1.03E-34 | 5.15E-33 | 68.35743305 |
| CXCL11 | 1.042581432 | 4.942965928 | 6.612751772 | 1.03E-09 | 4.08E-09 | 11.02018446 |
| ABCG1 | 1.049280799 | 7.524911382 | 11.91607779 | 3.04E-22 | 4.08E-21 | 39.67811922 |
| SERPINB9 | 1.049902563 | 8.091609848 | 8.033466197 | 6.51E-13 | 3.48E-12 | 18.29694568 |
| CTPS1 | 1.051089919 | 7.48675676 | 17.53380809 | 2.87E-35 | 1.51E-33 | 69.63736811 |
| IL4I1 | 1.052122851 | 6.424518115 | 18.9419427 | 2.72E-38 | 2.04E-36 | 76.58964324 |
| CD36 | 1.05230835 | 10.43396808 | 9.354396412 | 4.87E-16 | 3.54E-15 | 25.44392549 |
| PMEL | 1.052960704 | 9.211202281 | 10.17112854 | 5.20E-18 | 4.57E-17 | 29.960311 |
| EPSTI1 | 1.053901473 | 7.241741361 | 10.25425966 | 3.27E-18 | 2.93E-17 | 30.42228497 |
| HCAR3 | 1.054052093 | 9.849191292 | 14.08738818 | 1.95E-27 | 4.37E-26 | 51.61424549 |
| GSPT1 | 1.055687694 | 10.28602294 | 21.31968809 | 3.79E-43 | 4.60E-41 | 87.75670944 |
| GLRX3 | 1.055774206 | 10.38126769 | 23.18714549 | 9.50E-47 | 1.89E-44 | 96.03188578 |
| RDH12 | 1.055965724 | 9.573018992 | 14.48015575 | 2.34E-28 | 5.86E-27 | 53.73358601 |
| KCNK6 | 1.056048088 | 7.778048218 | 20.00055701 | 1.72E-40 | 1.58E-38 | 81.65006705 |
| CDCA5 | 1.056826104 | 6.565241442 | 19.98263614 | 1.87E-40 | 1.70E-38 | 81.56558968 |
| GINS1 | 1.057506436 | 7.127469965 | 14.47154549 | 2.45E-28 | 6.13E-27 | 53.6872853 |
| ST6GALNAC1 | 1.059513341 | 5.448096189 | 12.37743668 | 2.33E-23 | 3.52E-22 | 42.23871432 |
| EZH2 | 1.059694177 | 7.214719685 | 15.37618724 | 1.96E-30 | 6.03E-29 | 58.51020391 |
| BLNK | 1.06152635 | 8.595295205 | 15.6365683 | 4.98E-31 | 1.65E-29 | 59.88202262 |
| DDX39A | 1.061709758 | 9.804415717 | 23.90291894 | 4.42E-48 | 9.71E-46 | 99.09212354 |
| FANCI | 1.061953287 | 7.549274438 | 14.01391536 | 2.91E-27 | 6.41E-26 | 51.21618421 |
| NIPAL4 | 1.064570802 | 9.41315118 | 16.34883264 | 1.21E-32 | 4.93E-31 | 63.59495113 |
| AURKB | 1.066700359 | 6.332683379 | 19.5007093 | 1.85E-39 | 1.53E-37 | 79.27848991 |
| IRF8 | 1.067321803 | 8.9350923 | 14.2248004 | 9.28E-28 | 2.16E-26 | 52.35737868 |
| CENPF | 1.077757305 | 8.130164004 | 16.94674975 | 5.61E-34 | 2.59E-32 | 66.6653403 |
| HSPA4 | 1.077867287 | 9.493673664 | 21.64385827 | 8.72E-44 | 1.17E-41 | 89.223943 |
| CENPW | 1.078846839 | 8.703813154 | 16.98972005 | 4.50E-34 | 2.11E-32 | 66.88432494 |
| RALGPS2 | 1.079189805 | 9.01216673 | 16.14131045 | 3.55E-32 | 1.35E-30 | 62.51927912 |
| EHF | 1.07951054 | 11.67961074 | 17.45530482 | 4.25E-35 | 2.20E-33 | 69.24241839 |
| ANGPTL4 | 1.081156789 | 7.700665018 | 15.27538504 | 3.35E-30 | 1.01E-28 | 57.97710691 |
| C1QB | 1.082597867 | 8.400227493 | 8.525808153 | 4.58E-14 | 2.74E-13 | 20.92958967 |
| IRF9 | 1.084342791 | 9.832465128 | 20.90566483 | 2.53E-42 | 2.79E-40 | 85.86369598 |
| CYP7B1 | 1.085236607 | 5.168345284 | 16.78082329 | 1.31E-33 | 5.88E-32 | 65.81762748 |
| MAP3K9 | 1.085259297 | 7.235938886 | 12.30454252 | 3.50E-23 | 5.18E-22 | 41.83477642 |
| SLC28A3 | 1.085698042 | 7.528899934 | 10.63343484 | 3.93E-19 | 3.88E-18 | 32.53255937 |
| KIF18B | 1.08613929 | 7.201059978 | 18.9312954 | 2.87E-38 | 2.14E-36 | 76.53802043 |
| LRRC8B | 1.08753564 | 9.17874326 | 15.59935146 | 6.05E-31 | 1.98E-29 | 59.68641108 |
| HMOX1 | 1.087943617 | 9.263830415 | 9.726633849 | 6.18E-17 | 4.91E-16 | 27.496402 |
| FOSL1 | 1.088237691 | 5.795227435 | 10.94835509 | 6.76E-20 | 7.22E-19 | 34.2876287 |
| PCCA-DT | 1.089267629 | 6.785792795 | 13.90485305 | 5.26E-27 | 1.13E-25 | 50.62440546 |
| LYPD5 | 1.089986411 | 7.866562902 | 12.18264294 | 6.89E-23 | 9.87E-22 | 41.15871239 |
| ETHE1 | 1.090807013 | 9.148216558 | 19.01767918 | 1.89E-38 | 1.44E-36 | 76.95642985 |
| CYP4F22 | 1.091827415 | 8.878872508 | 9.964666721 | 1.64E-17 | 1.37E-16 | 28.81441212 |
| ELOVL7 | 1.093388061 | 10.55672903 | 16.46274466 | 6.72E-33 | 2.79E-31 | 64.18322861 |
| RASGRP1 | 1.093575552 | 8.958390288 | 11.03374215 | 4.19E-20 | 4.57E-19 | 34.76364823 |
| TTC22 | 1.09384653 | 9.332501025 | 14.32810343 | 5.31E-28 | 1.27E-26 | 52.91487554 |
| VSIG10L | 1.096235313 | 8.927674738 | 10.16525955 | 5.37E-18 | 4.71E-17 | 29.92770759 |
| GSDMC | 1.096979921 | 8.965559279 | 13.53576899 | 3.94E-26 | 7.83E-25 | 48.61406202 |
| POLE2 | 1.098293559 | 7.036241996 | 18.3466068 | 5.01E-37 | 3.15E-35 | 73.68101321 |
| TMEM79 | 1.101110843 | 10.18233567 | 16.58120832 | 3.65E-33 | 1.56E-31 | 64.79336335 |
| H2AX | 1.10210215 | 8.846189524 | 18.53232203 | 2.01E-37 | 1.33E-35 | 74.59320377 |
| FZD5 | 1.10262831 | 7.245368332 | 15.27634921 | 3.33E-30 | 1.01E-28 | 57.98221128 |
| AURKA | 1.105226561 | 7.041668011 | 17.49783925 | 3.43E-35 | 1.80E-33 | 69.45650452 |
| CH25H | 1.105999793 | 7.230183131 | 12.97221686 | 8.69E-25 | 1.53E-23 | 45.52357637 |
| BDH1 | 1.106211003 | 8.381853529 | 15.46837589 | 1.21E-30 | 3.83E-29 | 58.99676587 |
| SLPI | 1.106689032 | 11.76882129 | 19.08432383 | 1.37E-38 | 1.05E-36 | 77.27857994 |
| IL20 | 1.10908131 | 5.36673605 | 14.15294964 | 1.37E-27 | 3.11E-26 | 51.96902526 |
| CYP2C18 | 1.109310126 | 7.407920438 | 9.577182222 | 1.42E-16 | 1.08E-15 | 26.67093564 |
| IL4R | 1.115875892 | 8.716663087 | 19.37830863 | 3.32E-39 | 2.69E-37 | 78.69289245 |
| LAD1 | 1.119770986 | 9.617997211 | 12.04649404 | 1.47E-22 | 2.03E-21 | 40.40284707 |
| PRSS3P2 | 1.121731761 | 8.274640909 | 19.60986249 | 1.10E-39 | 9.37E-38 | 79.79909565 |
| MCM10 | 1.122593018 | 5.432080413 | 17.45799327 | 4.20E-35 | 2.18E-33 | 69.25595671 |
| HSPA4L | 1.123358141 | 8.115366999 | 16.22353559 | 2.32E-32 | 9.09E-31 | 62.94609451 |
| SHCBP1 | 1.125653939 | 5.552854165 | 14.15556752 | 1.35E-27 | 3.06E-26 | 51.98318341 |
| SERPINA3 | 1.126415685 | 8.395941361 | 7.188921411 | 5.52E-11 | 2.46E-10 | 13.90318466 |
| RAB38 | 1.126780898 | 10.44098267 | 20.4799545 | 1.81E-41 | 1.87E-39 | 83.89476393 |
| UCK2 | 1.128330967 | 8.202944694 | 24.40693982 | 5.28E-49 | 1.33E-46 | 101.2110002 |
| OTUB2 | 1.129926916 | 8.124713471 | 10.88120656 | 9.84E-20 | 1.03E-18 | 33.91331139 |
| FCGR1B | 1.131193025 | 5.698734309 | 11.27373676 | 1.10E-20 | 1.26E-19 | 36.10153734 |
| AMMECR1 | 1.132771575 | 8.743538793 | 8.782003637 | 1.13E-14 | 7.20E-14 | 22.31566842 |
| FAM83A | 1.136298449 | 8.143584803 | 15.76410778 | 2.55E-31 | 8.76E-30 | 60.55117823 |
| SLC7A1 | 1.136614359 | 11.18202419 | 24.88200826 | 7.31E-50 | 2.01E-47 | 103.1813808 |
| BUB1 | 1.137273326 | 5.379140731 | 16.47748021 | 6.23E-33 | 2.61E-31 | 64.25921429 |
| AMPD3 | 1.139966358 | 8.579530563 | 11.85892709 | 4.18E-22 | 5.54E-21 | 39.36033869 |
| MX2 | 1.142632574 | 8.34920389 | 9.3365467 | 5.37E-16 | 3.89E-15 | 25.34581718 |
| SPIN4 | 1.143653538 | 7.384921172 | 14.35378452 | 4.62E-28 | 1.12E-26 | 53.05331046 |
| TGFA | 1.14431144 | 9.130087381 | 13.57327896 | 3.21E-26 | 6.42E-25 | 48.81889515 |
| SOST | 1.148790119 | 5.078210989 | 9.721076044 | 6.38E-17 | 5.05E-16 | 27.46567403 |
| CENPN | 1.15240463 | 7.588290113 | 18.54611036 | 1.88E-37 | 1.25E-35 | 74.66075401 |
| CLDN17 | 1.153274263 | 6.06783548 | 9.778908129 | 4.62E-17 | 3.71E-16 | 27.78552474 |
| FOXM1 | 1.154725968 | 6.076989214 | 18.87800061 | 3.72E-38 | 2.71E-36 | 76.27940635 |
| FBXO6 | 1.155538741 | 8.010890484 | 13.58177186 | 3.06E-26 | 6.13E-25 | 48.86525692 |
| CCL22 | 1.155613846 | 7.072298261 | 15.96718945 | 8.80E-32 | 3.18E-30 | 61.6128326 |
| PLSCR1 | 1.156163129 | 9.926348911 | 14.33984525 | 4.99E-28 | 1.20E-26 | 52.9781782 |
| CENPA | 1.158622106 | 6.717639946 | 13.65045328 | 2.10E-26 | 4.27E-25 | 49.23996222 |
| PLCXD1 | 1.159592622 | 9.494484381 | 15.108916 | 8.10E-30 | 2.38E-28 | 57.0943024 |
| DEPDC1B | 1.160598339 | 4.851079247 | 14.3497807 | 4.73E-28 | 1.14E-26 | 53.03173191 |
| ECT2 | 1.161743526 | 7.029557439 | 12.37041366 | 2.43E-23 | 3.66E-22 | 42.19980827 |
| C12orf75 | 1.164160522 | 8.595625065 | 13.85009032 | 7.09E-27 | 1.51E-25 | 50.32686037 |
| KRT6B | 1.164897662 | 13.39886954 | 12.78163596 | 2.49E-24 | 4.20E-23 | 44.47336022 |
| GINS2 | 1.166752937 | 7.10223824 | 15.97986771 | 8.24E-32 | 2.99E-30 | 61.67895293 |
| ANLN | 1.167253975 | 7.356127687 | 10.40526425 | 1.41E-18 | 1.31E-17 | 31.26214929 |
| E2F8 | 1.173130062 | 6.492090993 | 12.10539087 | 1.06E-22 | 1.48E-21 | 40.72992295 |
| SMPD3 | 1.176300321 | 7.971917437 | 14.38154817 | 3.98E-28 | 9.73E-27 | 53.20289976 |
| LTB4R | 1.18960251 | 8.249699681 | 11.32264306 | 8.34E-21 | 9.74E-20 | 36.37412609 |
| FERMT1 | 1.191286331 | 10.28266345 | 13.61115743 | 2.61E-26 | 5.26E-25 | 49.02562361 |
| LINC01806 | 1.191706391 | 6.341935049 | 20.71408434 | 6.12E-42 | 6.55E-40 | 84.98045622 |
| AIM2 | 1.193071366 | 6.091439802 | 11.99435905 | 1.96E-22 | 2.69E-21 | 40.1132074 |
| SLC16A6 | 1.197510203 | 8.087432754 | 12.97320109 | 8.65E-25 | 1.52E-23 | 45.52899386 |
| LRP8 | 1.198580087 | 6.751068235 | 16.05427707 | 5.59E-32 | 2.07E-30 | 62.06663973 |
| JPT1 | 1.20059968 | 9.267449218 | 24.91374954 | 6.41E-50 | 1.81E-47 | 103.3121139 |
| TRIM22 | 1.200978245 | 10.42931429 | 14.98660256 | 1.55E-29 | 4.42E-28 | 56.44376162 |
| SMOX | 1.201822126 | 7.763581322 | 15.53775327 | 8.37E-31 | 2.69E-29 | 59.36230845 |
| NMI | 1.203178191 | 9.776980508 | 25.38538716 | 9.26E-51 | 3.07E-48 | 105.2412769 |
| KIF11 | 1.20663921 | 6.640006482 | 15.02383956 | 1.27E-29 | 3.66E-28 | 56.64198035 |
| SOX7 | 1.207278082 | 9.897696 | 19.4110141 | 2.84E-39 | 2.33E-37 | 78.84955133 |
| GK | 1.210239245 | 7.25084045 | 15.54772762 | 7.94E-31 | 2.57E-29 | 59.414818 |
| INA | 1.211124443 | 6.224556536 | 19.0880921 | 1.34E-38 | 1.04E-36 | 77.29677829 |
| UBE2L6 | 1.212544143 | 9.468659293 | 20.14565994 | 8.67E-41 | 8.30E-39 | 82.3325656 |
| EIF4EBP1 | 1.213509652 | 8.090405984 | 22.99559615 | 2.18E-46 | 4.10E-44 | 95.20260259 |
| TNIP3 | 1.215233858 | 4.723684655 | 9.716796398 | 6.53E-17 | 5.17E-16 | 27.44201428 |
| TMEM54 | 1.217924617 | 9.51316588 | 17.62518498 | 1.81E-35 | 9.71E-34 | 70.09611452 |
| PRC1 | 1.217990166 | 8.424240054 | 18.99652959 | 2.09E-38 | 1.59E-36 | 76.85407747 |
| PSME2 | 1.219298513 | 10.87690779 | 30.67366778 | 1.67E-59 | 1.59E-56 | 125.2697885 |
| TNFRSF21 | 1.219977858 | 9.117697169 | 20.3727277 | 2.99E-41 | 3.03E-39 | 83.39522366 |
| IDO1 | 1.220687665 | 6.449262161 | 15.35588464 | 2.19E-30 | 6.69E-29 | 58.40292257 |
| HBEGF | 1.222464368 | 8.583383414 | 11.34722779 | 7.27E-21 | 8.55E-20 | 36.51114369 |
| GINS3 | 1.222638661 | 6.960497057 | 22.83724724 | 4.35E-46 | 7.90E-44 | 94.51372985 |
| AREG | 1.226057077 | 6.78331025 | 10.9660936 | 6.12E-20 | 6.57E-19 | 34.38651596 |
| KIF4A | 1.227457675 | 6.225099429 | 18.53121091 | 2.02E-37 | 1.33E-35 | 74.58775929 |
| SCO2 | 1.229229864 | 9.193397147 | 16.9606876 | 5.22E-34 | 2.43E-32 | 66.73639501 |
| GCH1 | 1.231424668 | 9.350892653 | 23.17598335 | 9.97E-47 | 1.96E-44 | 95.98368151 |
| PRSS2 | 1.236041826 | 8.112824546 | 18.81508055 | 5.05E-38 | 3.59E-36 | 75.9736192 |
| VSNL1 | 1.240816733 | 9.971626913 | 16.0026802 | 7.32E-32 | 2.67E-30 | 61.79787884 |
| S100P | 1.241578165 | 9.492857187 | 8.085619354 | 4.92E-13 | 2.66E-12 | 18.57365404 |
| C12orf56 | 1.242776252 | 5.63381382 | 18.96860239 | 2.39E-38 | 1.80E-36 | 76.71883777 |
| DUOXA1 | 1.244291164 | 8.393445091 | 17.32843717 | 8.07E-35 | 4.07E-33 | 68.6025231 |
| ID1 | 1.245534797 | 11.33116185 | 17.58507025 | 2.21E-35 | 1.18E-33 | 69.89485219 |
| HSD17B2 | 1.246999537 | 6.901877296 | 7.923204985 | 1.17E-12 | 6.14E-12 | 17.71378449 |
| SLC16A1 | 1.249027584 | 8.675411048 | 19.53161019 | 1.59E-39 | 1.34E-37 | 79.42602583 |
| CCNE2 | 1.250180786 | 6.198518535 | 14.15176827 | 1.38E-27 | 3.12E-26 | 51.96263591 |
| LDLR | 1.250707429 | 9.689161462 | 15.18489627 | 5.41E-30 | 1.61E-28 | 57.49760683 |
| MREG | 1.252063966 | 10.11528329 | 24.10450349 | 1.88E-48 | 4.41E-46 | 99.94311351 |
| GBAP1 | 1.253878716 | 9.563851277 | 18.80148309 | 5.39E-38 | 3.81E-36 | 75.90747015 |
| DDX60 | 1.254876043 | 9.438608885 | 12.07744553 | 1.24E-22 | 1.72E-21 | 40.57474967 |
| CALML3 | 1.256580546 | 11.3626668 | 10.6430589 | 3.73E-19 | 3.69E-18 | 32.58617241 |
| NCAPG | 1.262493968 | 5.843442553 | 18.53127414 | 2.02E-37 | 1.33E-35 | 74.58806908 |
| PDSS1 | 1.2713787 | 6.857115647 | 19.6884904 | 7.54E-40 | 6.50E-38 | 80.17316979 |
| DHRS9 | 1.27168665 | 8.18555498 | 12.5217063 | 1.05E-23 | 1.65E-22 | 43.03737141 |
| CCL2 | 1.274165157 | 8.882294374 | 10.2242492 | 3.87E-18 | 3.44E-17 | 30.25547713 |
| FLVCR2 | 1.27420172 | 7.302736986 | 19.28585951 | 5.17E-39 | 4.15E-37 | 78.24932396 |
| AASS | 1.277167447 | 5.910325823 | 13.99795181 | 3.17E-27 | 6.95E-26 | 51.12963184 |
| PYCARD | 1.277620548 | 10.60653256 | 26.13857954 | 4.42E-52 | 1.77E-49 | 108.2708653 |
| PRDM1 | 1.278181642 | 9.3121015 | 14.81365327 | 3.91E-29 | 1.06E-27 | 55.52121162 |
| FPR1 | 1.283975715 | 7.029940131 | 13.32453113 | 1.25E-25 | 2.38E-24 | 47.45844432 |
| NEK2 | 1.287187357 | 6.810361599 | 18.5546371 | 1.80E-37 | 1.21E-35 | 74.70251511 |
| LGALS3BP | 1.292564278 | 9.57008884 | 14.30480366 | 6.02E-28 | 1.43E-26 | 52.78922236 |
| PHLDA2 | 1.293511524 | 8.557064277 | 14.26602256 | 7.43E-28 | 1.74E-26 | 52.57996481 |
| IL7R | 1.294271391 | 8.510938418 | 11.42418248 | 4.73E-21 | 5.67E-20 | 36.93998146 |
| TXNDC17 | 1.297902466 | 11.21697402 | 24.63239967 | 2.06E-49 | 5.30E-47 | 102.1493261 |
| UBE2F | 1.298424144 | 9.820162225 | 23.23479897 | 7.73E-47 | 1.55E-44 | 96.23751244 |
| NABP1 | 1.299221306 | 8.196958215 | 17.66455011 | 1.49E-35 | 8.03E-34 | 70.29341992 |
| FUT3 | 1.300007299 | 6.379049538 | 20.76695339 | 4.79E-42 | 5.18E-40 | 85.2246594 |
| MOXD1 | 1.302668376 | 8.4729778 | 20.32903562 | 3.67E-41 | 3.66E-39 | 83.19125654 |
| GK3P | 1.304815801 | 6.060623118 | 15.46164536 | 1.25E-30 | 3.95E-29 | 58.96127476 |
| APOL1 | 1.311455183 | 7.829984437 | 18.69541413 | 9.04E-38 | 6.21E-36 | 75.39065583 |
| C15orf48 | 1.319091892 | 10.64281921 | 11.08155363 | 3.21E-20 | 3.54E-19 | 35.0301955 |
| KPNA2 | 1.32015598 | 10.4004667 | 27.96278683 | 3.59E-55 | 2.14E-52 | 115.356587 |
| TMEM165 | 1.321886303 | 9.899119618 | 26.21631732 | 3.24E-52 | 1.33E-49 | 108.5800193 |
| ZBED2 | 1.322465657 | 6.356222508 | 12.22898648 | 5.32E-23 | 7.73E-22 | 41.41581774 |
| CYP2E1 | 1.323080526 | 7.746719523 | 10.35273732 | 1.89E-18 | 1.74E-17 | 30.96990811 |
| ULBP2 | 1.323351828 | 6.665758648 | 16.75120311 | 1.52E-33 | 6.74E-32 | 65.66594581 |
| TMEM40 | 1.324131673 | 10.21188558 | 17.7239702 | 1.10E-35 | 6.06E-34 | 70.59087632 |
| NT5C3A | 1.327323383 | 10.56198332 | 19.51231587 | 1.75E-39 | 1.46E-37 | 79.3339196 |
| IDH3A | 1.332610203 | 8.900442585 | 24.68638606 | 1.65E-49 | 4.40E-47 | 102.3731447 |
| TPBG | 1.334089344 | 10.07445399 | 26.88442886 | 2.31E-53 | 1.07E-50 | 111.2102474 |
| AFAP1L2 | 1.334251142 | 6.839735418 | 16.33430302 | 1.31E-32 | 5.28E-31 | 63.51980429 |
| CALHM6 | 1.337642134 | 7.479460352 | 12.46379038 | 1.45E-23 | 2.23E-22 | 42.71688902 |
| KLRB1 | 1.338261979 | 6.0941426 | 14.75641967 | 5.31E-29 | 1.43E-27 | 55.21522969 |
| MPHOSPH6 | 1.343228874 | 10.24465558 | 25.00955448 | 4.32E-50 | 1.25E-47 | 103.7060152 |
| HR | 1.346460427 | 9.531761328 | 17.96764087 | 3.27E-36 | 1.90E-34 | 71.80604416 |
| ZWINT | 1.348565943 | 8.73989821 | 17.61717047 | 1.88E-35 | 1.01E-33 | 70.05592052 |
| KLK8 | 1.349562305 | 10.75404887 | 17.71886649 | 1.13E-35 | 6.18E-34 | 70.56534463 |
| LIPG | 1.349663724 | 5.606160847 | 11.69891125 | 1.02E-21 | 1.31E-20 | 38.47003822 |
| EPHA2 | 1.352291515 | 8.360446122 | 21.32894124 | 3.64E-43 | 4.44E-41 | 87.79877188 |
| GPT2 | 1.352604965 | 8.538842013 | 14.7508111 | 5.47E-29 | 1.47E-27 | 55.18522701 |
| CD2 | 1.363906369 | 7.787682347 | 16.13614365 | 3.65E-32 | 1.38E-30 | 62.49243264 |
| CCNE1 | 1.367805396 | 7.52213917 | 19.2809402 | 5.30E-39 | 4.23E-37 | 78.22569062 |
| WDR4 | 1.369614884 | 7.720078934 | 16.36223947 | 1.13E-32 | 4.62E-31 | 63.66426855 |
| EREG | 1.370699353 | 8.70698629 | 10.51911831 | 7.45E-19 | 7.16E-18 | 31.89589314 |
| FBXO45 | 1.372434208 | 10.18806604 | 18.51306868 | 2.21E-37 | 1.44E-35 | 74.49883952 |
| MICALL1 | 1.375250969 | 9.910272369 | 24.90307491 | 6.70E-50 | 1.86E-47 | 103.268161 |
| UBE2T | 1.376964249 | 6.946219257 | 20.3863138 | 2.81E-41 | 2.86E-39 | 83.45859814 |
| TPX2 | 1.377029492 | 7.370207016 | 17.56001506 | 2.51E-35 | 1.33E-33 | 69.76904385 |
| TYMS | 1.37992252 | 8.923539961 | 18.54407608 | 1.90E-37 | 1.26E-35 | 74.65078941 |
| SERPINA1 | 1.380082952 | 7.193350042 | 14.39858608 | 3.63E-28 | 8.94E-27 | 53.29466228 |
| CCNA2 | 1.382129122 | 7.31245422 | 15.38210529 | 1.90E-30 | 5.86E-29 | 58.54146704 |
| GNA15 | 1.382343925 | 9.124616848 | 19.59337198 | 1.19E-39 | 1.00E-37 | 79.72054169 |
| LCK | 1.382680273 | 7.269719684 | 16.53884493 | 4.54E-33 | 1.91E-31 | 64.57536891 |
| BUB1B | 1.387114194 | 7.126260435 | 17.91492038 | 4.25E-36 | 2.44E-34 | 71.54376524 |
| PGM2 | 1.389020705 | 10.2529447 | 23.71774424 | 9.72E-48 | 2.05E-45 | 98.30622033 |
| EPN3 | 1.389095802 | 8.659668602 | 16.30211495 | 1.54E-32 | 6.19E-31 | 63.35323911 |
| COMP | 1.391870154 | 8.182423675 | 7.361317942 | 2.26E-11 | 1.04E-10 | 14.78537565 |
| CD24 | 1.392435058 | 12.15313826 | 25.52598057 | 5.22E-51 | 1.76E-48 | 105.8115392 |
| TMEM86A | 1.39267933 | 7.918331639 | 18.65908009 | 1.08E-37 | 7.39E-36 | 75.21329087 |
| KIF2C | 1.393118201 | 7.572387169 | 20.69412051 | 6.71E-42 | 7.11E-40 | 84.88815121 |
| GZMA | 1.393900269 | 6.7050869 | 14.27685873 | 7.01E-28 | 1.65E-26 | 52.63844977 |
| HMMR | 1.402033536 | 6.791986403 | 13.43746827 | 6.74E-26 | 1.31E-24 | 48.07672636 |
| F12 | 1.404410595 | 8.242362967 | 22.58716309 | 1.30E-45 | 2.16E-43 | 93.41961278 |
| PCP4L1 | 1.407949938 | 6.591009984 | 13.1170148 | 3.92E-25 | 7.13E-24 | 46.31986861 |
| GPR68 | 1.409652235 | 8.466633148 | 26.97326394 | 1.63E-53 | 7.74E-51 | 111.556398 |
| PRKCQ | 1.411603264 | 6.336145448 | 19.70688253 | 6.91E-40 | 5.98E-38 | 80.26055703 |
| PRSS53 | 1.41645408 | 7.292400082 | 22.61923524 | 1.13E-45 | 1.89E-43 | 93.56035164 |
| NUSAP1 | 1.421479336 | 8.6350089 | 20.81073578 | 3.91E-42 | 4.25E-40 | 85.42662438 |
| FAM83D | 1.430782261 | 7.850657602 | 14.60122915 | 1.22E-28 | 3.15E-27 | 54.38386568 |
| TK1 | 1.438826907 | 7.977625703 | 23.80969548 | 6.57E-48 | 1.40E-45 | 98.69697494 |
| HK2 | 1.444139346 | 9.363068603 | 17.35098044 | 7.20E-35 | 3.65E-33 | 68.71637327 |
| FCGR3B | 1.444832668 | 6.562048698 | 12.33303924 | 2.99E-23 | 4.46E-22 | 41.99272019 |
| IRAK2 | 1.446151355 | 6.993544844 | 17.86925027 | 5.34E-36 | 3.02E-34 | 71.31627778 |
| UBE2C | 1.446822519 | 8.205255427 | 25.05068951 | 3.65E-50 | 1.07E-47 | 103.8748235 |
| UNC93A | 1.447482454 | 8.329963907 | 10.73814143 | 2.19E-19 | 2.23E-18 | 33.11594457 |
| NETO2 | 1.44858695 | 6.340074623 | 14.28976408 | 6.53E-28 | 1.55E-26 | 52.7080879 |
| PLA2G3 | 1.452250416 | 8.729545457 | 21.06345569 | 1.22E-42 | 1.40E-40 | 86.58768837 |
| FABP5 | 1.468451157 | 13.11081374 | 30.69093206 | 1.57E-59 | 1.56E-56 | 125.3306941 |
| SPTSSA | 1.475800553 | 10.30982133 | 21.17639874 | 7.29E-43 | 8.50E-41 | 87.10398675 |
| SELL | 1.477187021 | 5.650123486 | 13.33544863 | 1.18E-25 | 2.25E-24 | 47.51825597 |
| IFIH1 | 1.477890057 | 7.746298044 | 15.72406087 | 3.14E-31 | 1.07E-29 | 60.34126529 |
| DNASE1L3 | 1.481009434 | 8.68535279 | 16.6375827 | 2.73E-33 | 1.19E-31 | 65.0831203 |
| PPIF | 1.484514107 | 9.835643805 | 18.95526147 | 2.55E-38 | 1.92E-36 | 76.65419817 |
| PLAC8 | 1.492219564 | 5.509128629 | 12.23608025 | 5.12E-23 | 7.45E-22 | 41.45516407 |
| CARD6 | 1.494825565 | 8.136051245 | 21.93984755 | 2.31E-44 | 3.36E-42 | 90.55223575 |
| EPHX3 | 1.507933915 | 10.13495742 | 21.8608657 | 3.29E-44 | 4.66E-42 | 90.19885334 |
| GPX2 | 1.510702518 | 7.916629348 | 15.6018555 | 5.97E-31 | 1.96E-29 | 59.69957725 |
| CDK1 | 1.51266136 | 8.736757768 | 20.96859666 | 1.89E-42 | 2.13E-40 | 86.15282133 |
| IVL | 1.517259347 | 11.24692914 | 22.52445577 | 1.72E-45 | 2.82E-43 | 93.14407993 |
| BCL2A1 | 1.526303953 | 5.465291911 | 17.76975258 | 8.77E-36 | 4.86E-34 | 70.81975997 |
| SELE | 1.530676863 | 6.551419388 | 9.646191847 | 9.66E-17 | 7.51E-16 | 27.05188008 |
| STAT1 | 1.532869763 | 10.81002041 | 23.34893326 | 4.72E-47 | 9.66E-45 | 96.7289058 |
| ISG20 | 1.540133439 | 7.270096324 | 19.35341867 | 3.74E-39 | 3.02E-37 | 78.57357839 |
| MMP9 | 1.543298433 | 8.461254463 | 18.54601618 | 1.88E-37 | 1.25E-35 | 74.6602927 |
| FAM43A | 1.54791343 | 9.524433426 | 18.76779155 | 6.35E-38 | 4.43E-36 | 75.74346563 |
| TRIM14 | 1.554631566 | 8.765620711 | 24.91502379 | 6.38E-50 | 1.81E-47 | 103.3173598 |
| SLAMF7 | 1.557755989 | 6.135920455 | 18.69847297 | 8.91E-38 | 6.14E-36 | 75.40557993 |
| APOBEC3B | 1.55789633 | 6.184109011 | 15.46905321 | 1.20E-30 | 3.82E-29 | 59.00033716 |
| MAD2L1 | 1.561190243 | 7.898828529 | 16.04115837 | 5.99E-32 | 2.21E-30 | 61.99833569 |
| NFKBIZ | 1.563578405 | 10.93318109 | 24.17511912 | 1.40E-48 | 3.39E-46 | 100.240099 |
| IFIT1 | 1.575501012 | 9.582256179 | 11.512494 | 2.89E-21 | 3.53E-20 | 37.43198928 |
| CHAC2 | 1.576942635 | 6.07081218 | 15.79553856 | 2.16E-31 | 7.48E-30 | 60.71579985 |
| GRHL3 | 1.585852317 | 9.039580525 | 20.96468406 | 1.93E-42 | 2.16E-40 | 86.13486034 |
| TMPRSS4 | 1.587069607 | 7.121427523 | 22.16879761 | 8.30E-45 | 1.30E-42 | 91.57226785 |
| NOD2 | 1.588698709 | 8.226784772 | 25.69242354 | 2.66E-51 | 9.91E-49 | 106.4838176 |
| ADAP2 | 1.593770988 | 9.084617144 | 27.39037299 | 3.23E-54 | 1.68E-51 | 113.1706231 |
| ALDH1A3 | 1.599522112 | 8.321918417 | 12.9969268 | 7.59E-25 | 1.35E-23 | 45.65956712 |
| SOCS3 | 1.606926759 | 6.515903959 | 15.00771197 | 1.39E-29 | 3.98E-28 | 56.55614858 |
| PI15 | 1.610382734 | 6.835549885 | 10.86807047 | 1.06E-19 | 1.11E-18 | 33.84008865 |
| NDC80 | 1.611422309 | 6.426010136 | 22.1146337 | 1.06E-44 | 1.62E-42 | 91.33153538 |
| PTTG1 | 1.619044717 | 9.76449996 | 26.78524112 | 3.41E-53 | 1.51E-50 | 110.8227721 |
| GBP1 | 1.619914748 | 9.864533639 | 14.37528445 | 4.12E-28 | 1.00E-26 | 53.16915768 |
| IFIT3 | 1.621371037 | 9.382720426 | 13.18934294 | 2.63E-25 | 4.85E-24 | 46.71706992 |
| UHRF1 | 1.622758111 | 7.238738481 | 19.19042301 | 8.19E-39 | 6.45E-37 | 77.79027777 |
| CRABP2 | 1.623733036 | 11.05763264 | 15.8595846 | 1.54E-31 | 5.46E-30 | 61.05089621 |
| TTK | 1.628479418 | 6.097939569 | 16.27915298 | 1.74E-32 | 6.89E-31 | 63.23434148 |
| TOP2A | 1.633792186 | 8.305299645 | 15.20725486 | 4.81E-30 | 1.44E-28 | 57.61616777 |
| SLC16A10 | 1.638868517 | 9.081311136 | 18.36078834 | 4.67E-37 | 2.97E-35 | 73.75082425 |
| RNASE7 | 1.639079611 | 9.191946975 | 9.922694323 | 2.08E-17 | 1.72E-16 | 28.58174411 |
| POLR3G | 1.647041034 | 6.072542436 | 21.89194156 | 2.86E-44 | 4.08E-42 | 90.33798552 |
| S100A2 | 1.64954092 | 12.52885315 | 23.00504417 | 2.09E-46 | 3.97E-44 | 95.24360946 |
| ATP10B | 1.650193938 | 8.499327472 | 18.59992295 | 1.44E-37 | 9.77E-36 | 74.92415496 |
| SAMSN1 | 1.658746803 | 6.629247006 | 13.03538685 | 6.14E-25 | 1.10E-23 | 45.87114825 |
| KLRK1-AS1 | 1.665353706 | 6.561864861 | 15.97006889 | 8.67E-32 | 3.14E-30 | 61.62785124 |
| GM2A | 1.674366708 | 11.58397095 | 27.41137816 | 2.97E-54 | 1.59E-51 | 113.2514344 |
| ASPM | 1.679320035 | 7.439264863 | 19.10517355 | 1.24E-38 | 9.58E-37 | 77.37924795 |
| IFI44 | 1.686879821 | 8.0640688 | 12.88439603 | 1.41E-24 | 2.42E-23 | 45.03992447 |
| GBP6 | 1.689607833 | 5.67628693 | 14.40081966 | 3.59E-28 | 8.84E-27 | 53.30668975 |
| SLC7A5 | 1.69109897 | 8.719102665 | 11.37893482 | 6.09E-21 | 7.22E-20 | 36.68784448 |
| MKI67 | 1.692263117 | 7.114397668 | 25.22587456 | 1.78E-50 | 5.55E-48 | 104.5916085 |
| CERS3 | 1.696315121 | 9.905566019 | 20.04463454 | 1.40E-40 | 1.31E-38 | 81.85767085 |
| MMP1 | 1.702291319 | 5.142518624 | 8.390713284 | 9.52E-14 | 5.51E-13 | 20.20287875 |
| PRSS3 | 1.709528676 | 10.23208729 | 21.46326129 | 1.98E-43 | 2.49E-41 | 88.40815338 |
| HRH2 | 1.7138283 | 6.075042393 | 17.83519676 | 6.33E-36 | 3.55E-34 | 71.14648277 |
| CCR7 | 1.715326734 | 6.189726736 | 21.64913899 | 8.52E-44 | 1.15E-41 | 89.247736 |
| XAF1 | 1.717243669 | 8.7068925 | 13.01444679 | 6.89E-25 | 1.23E-23 | 45.75596283 |
| CHAC1 | 1.7189532 | 6.619216413 | 17.09424298 | 2.65E-34 | 1.26E-32 | 67.41604589 |
| VNN1 | 1.7251669 | 5.748844699 | 12.70167517 | 3.87E-24 | 6.43E-23 | 44.03204106 |
| FAM110C | 1.727223898 | 9.330850656 | 23.62464484 | 1.45E-47 | 3.02E-45 | 97.90957341 |
| NAMPT | 1.730936183 | 10.41860317 | 23.08094732 | 1.51E-46 | 2.94E-44 | 95.57266012 |
| IFI44L | 1.740281539 | 7.384105144 | 7.381100336 | 2.04E-11 | 9.42E-11 | 14.88713079 |
| KIF20A | 1.74346166 | 6.47150698 | 22.34019022 | 3.88E-45 | 6.22E-43 | 92.33165682 |
| FUT2 | 1.745846826 | 7.677373363 | 15.06534192 | 1.02E-29 | 2.98E-28 | 56.86273063 |
| BIRC5 | 1.746180319 | 7.368756335 | 25.55593136 | 4.62E-51 | 1.63E-48 | 105.9327394 |
| DSG3 | 1.751509627 | 10.46123595 | 21.58531463 | 1.14E-43 | 1.49E-41 | 88.95993474 |
| CDKN3 | 1.753540381 | 7.085500499 | 20.94184391 | 2.14E-42 | 2.39E-40 | 86.02997303 |
| GGH | 1.75776574 | 9.875410886 | 22.45446287 | 2.34E-45 | 3.81E-43 | 92.83596992 |
| SERPINB1 | 1.760231082 | 9.558002995 | 20.9338465 | 2.22E-42 | 2.46E-40 | 85.99323155 |
| DSC2 | 1.768264995 | 11.20527307 | 24.00344453 | 2.89E-48 | 6.58E-46 | 99.51708494 |
| ACP3 | 1.770147862 | 9.861844303 | 17.71508015 | 1.15E-35 | 6.28E-34 | 70.54640101 |
| ABCA12 | 1.784329795 | 9.180155438 | 19.13659521 | 1.06E-38 | 8.33E-37 | 77.53085524 |
| CYSRT1 | 1.78787088 | 9.066569066 | 20.2196867 | 6.13E-41 | 5.98E-39 | 82.67972347 |
| ZDHHC21 | 1.789150531 | 7.650081557 | 22.02914962 | 1.55E-44 | 2.30E-42 | 90.95086668 |
| CEP55 | 1.802915067 | 6.626069985 | 19.98932471 | 1.81E-40 | 1.66E-38 | 81.59712376 |
| HYAL4 | 1.805436113 | 6.099203316 | 18.401079 | 3.83E-37 | 2.47E-35 | 73.94902252 |
| PLBD1 | 1.824817002 | 10.70722435 | 33.32205485 | 1.85E-63 | 2.15E-60 | 134.3059323 |
| GJB6 | 1.825501705 | 11.98442028 | 26.47373168 | 1.17E-52 | 5.07E-50 | 109.5990675 |
| GALNT6 | 1.831688739 | 7.995571282 | 16.52242843 | 4.94E-33 | 2.08E-31 | 64.49083447 |
| CCNB2 | 1.840112343 | 8.179898901 | 23.84993 | 5.53E-48 | 1.20E-45 | 98.86764285 |
| PARP9 | 1.840202806 | 8.890733571 | 22.93383009 | 2.86E-46 | 5.27E-44 | 94.9342577 |
| FGFBP1 | 1.84491878 | 10.22960924 | 19.94153015 | 2.27E-40 | 2.05E-38 | 81.37166529 |
| CDH3 | 1.846592274 | 9.692285395 | 20.70308767 | 6.44E-42 | 6.85E-40 | 84.92961812 |
| TIGAR | 1.855587352 | 7.813941137 | 19.74654368 | 5.72E-40 | 5.02E-38 | 80.44885392 |
| SLC26A9 | 1.855633247 | 6.714996016 | 19.26990984 | 5.58E-39 | 4.43E-37 | 78.17268749 |
| MELK | 1.871084861 | 7.598600723 | 27.88424527 | 4.84E-55 | 2.80E-52 | 115.0586317 |
| PBK | 1.874685258 | 7.371753112 | 20.17136605 | 7.69E-41 | 7.41E-39 | 82.45319653 |
| RTP4 | 1.880050468 | 6.780072007 | 19.59573259 | 1.17E-39 | 9.94E-38 | 79.73178878 |
| ARG1 | 1.887622457 | 11.72597663 | 17.21991879 | 1.40E-34 | 6.86E-33 | 68.05359037 |
| CDC20 | 1.907879083 | 7.542793837 | 25.68621493 | 2.73E-51 | 9.98E-49 | 106.4587954 |
| IL19 | 1.909684066 | 5.880039328 | 13.26346342 | 1.75E-25 | 3.26E-24 | 47.12371725 |
| SPRR1A | 1.918868466 | 12.34987039 | 14.78917606 | 4.46E-29 | 1.21E-27 | 55.39039308 |
| SDR9C7 | 1.924943151 | 9.093882657 | 18.61793103 | 1.32E-37 | 8.98E-36 | 75.01221824 |
| CLEC7A | 1.930402934 | 8.504282861 | 25.53183216 | 5.10E-51 | 1.74E-48 | 105.8352263 |
| PNP | 1.938688016 | 8.833763603 | 23.07191413 | 1.57E-46 | 3.03E-44 | 95.5335362 |
| CXCL2 | 1.950507546 | 5.753029307 | 15.89879864 | 1.26E-31 | 4.49E-30 | 61.25583563 |
| IL36A | 1.954145892 | 6.166253444 | 15.09846443 | 8.56E-30 | 2.51E-28 | 57.03877672 |
| LRG1 | 1.954313797 | 8.073430205 | 17.40148123 | 5.58E-35 | 2.85E-33 | 68.97118765 |
| CXCR4 | 1.966248327 | 7.744443258 | 22.77473887 | 5.72E-46 | 1.02E-43 | 94.24096496 |
| PLAT | 1.970416009 | 7.634926037 | 12.23101114 | 5.27E-23 | 7.65E-22 | 41.42704795 |
| CHRNA9 | 1.984826369 | 6.175157481 | 14.91937356 | 2.22E-29 | 6.24E-28 | 56.08551943 |
| CARHSP1 | 2.007443596 | 9.963688307 | 26.44923329 | 1.28E-52 | 5.47E-50 | 109.5023906 |
| CFB | 2.007953181 | 7.384639437 | 23.96273941 | 3.43E-48 | 7.69E-46 | 99.34515046 |
| KLK10 | 2.009968584 | 10.89338145 | 18.06022111 | 2.06E-36 | 1.22E-34 | 72.26577174 |
| DLGAP5 | 2.02281559 | 6.506791869 | 24.6528838 | 1.89E-49 | 4.99E-47 | 102.2342891 |
| CKS2 | 2.027322816 | 8.90642192 | 22.34494457 | 3.80E-45 | 6.14E-43 | 92.3526707 |
| KLHDC7B-DT | 2.048982466 | 6.978659918 | 25.9445007 | 9.63E-52 | 3.65E-49 | 107.4961705 |
| TMEM45B | 2.069138489 | 9.463551461 | 29.80065381 | 3.85E-58 | 3.21E-55 | 122.1540731 |
| SH3PXD2A-AS1 | 2.077865495 | 6.720055739 | 24.00232549 | 2.90E-48 | 6.58E-46 | 99.51236081 |
| PCLAF | 2.080449584 | 9.643034634 | 26.08234858 | 5.54E-52 | 2.14E-49 | 108.0468328 |
| OAS1 | 2.106468613 | 7.911535483 | 15.18409333 | 5.43E-30 | 1.62E-28 | 57.49334806 |
| WFDC12 | 2.106608891 | 9.928996253 | 14.9514191 | 1.87E-29 | 5.29E-28 | 56.25633945 |
| MPZL2 | 2.107454606 | 9.913752339 | 27.33636681 | 3.97E-54 | 2.02E-51 | 112.9626396 |
| ALOX12B | 2.115002892 | 9.854329489 | 17.10078739 | 2.56E-34 | 1.23E-32 | 67.44929335 |
| SLC5A1 | 2.118680144 | 7.794422269 | 25.1887897 | 2.07E-50 | 6.16E-48 | 104.4401601 |
| SLC7A11 | 2.125315597 | 7.384119148 | 22.70097602 | 7.90E-46 | 1.35E-43 | 93.91848226 |
| FCHSD1 | 2.129378039 | 7.867267026 | 17.91396342 | 4.27E-36 | 2.44E-34 | 71.53900123 |
| ARNTL2 | 2.137176108 | 7.717055621 | 31.93174223 | 2.05E-61 | 2.25E-58 | 129.6380498 |
| CMPK2 | 2.155641921 | 8.850920479 | 14.19067085 | 1.12E-27 | 2.58E-26 | 52.17296883 |
| MX1 | 2.155884629 | 10.09789945 | 15.9997948 | 7.43E-32 | 2.71E-30 | 61.78284006 |
| CXCR2 | 2.164052094 | 6.847335878 | 24.48733263 | 3.77E-49 | 9.59E-47 | 101.5462506 |
| IRF7 | 2.175516051 | 8.089311645 | 24.16703762 | 1.45E-48 | 3.47E-46 | 100.2061403 |
| VNN3 | 2.185140355 | 6.304453463 | 22.0022734 | 1.74E-44 | 2.56E-42 | 90.83099861 |
| GDPD3 | 2.192825789 | 8.190073696 | 24.12824499 | 1.70E-48 | 4.04E-46 | 100.0430269 |
| LAMP3 | 2.202362621 | 9.22675839 | 22.75164004 | 6.33E-46 | 1.12E-43 | 94.14005029 |
| OASL | 2.248898412 | 7.520742353 | 20.96958739 | 1.88E-42 | 2.13E-40 | 86.15736906 |
| HAL | 2.258962476 | 10.81410485 | 16.74461918 | 1.58E-33 | 6.96E-32 | 65.63221569 |
| ARSF | 2.267682406 | 7.315364402 | 21.92784477 | 2.43E-44 | 3.53E-42 | 90.49858221 |
| UPP1 | 2.278515018 | 7.253794344 | 21.66578684 | 7.90E-44 | 1.08E-41 | 89.32272253 |
| SPRR1B | 2.278916518 | 12.47718599 | 25.55323751 | 4.67E-51 | 1.63E-48 | 105.9218424 |
| KRT6A | 2.285553954 | 12.59516544 | 20.48123402 | 1.80E-41 | 1.86E-39 | 83.90071607 |
| IFI6 | 2.306304083 | 9.38602597 | 16.05743092 | 5.50E-32 | 2.04E-30 | 62.0830576 |
| RRM2 | 2.310694869 | 8.70563706 | 25.19288858 | 2.03E-50 | 6.15E-48 | 104.4569069 |
| SAMD9 | 2.324980452 | 7.379785779 | 21.46294353 | 1.98E-43 | 2.49E-41 | 88.40671443 |
| WNT5A | 2.335206084 | 8.694591487 | 22.70913047 | 7.63E-46 | 1.31E-43 | 93.95416491 |
| TGM3 | 2.340129756 | 9.819222163 | 18.85648256 | 4.13E-38 | 2.96E-36 | 76.17488692 |
| TREX2 | 2.383529048 | 7.039483202 | 22.25065335 | 5.77E-45 | 9.18E-43 | 91.93539452 |
| RSAD2 | 2.396140055 | 7.455376406 | 13.10766519 | 4.12E-25 | 7.50E-24 | 46.26849634 |
| PDZK1IP1 | 2.417832842 | 10.33691126 | 35.3904652 | 2.24E-66 | 2.92E-63 | 140.9592846 |
| ISG15 | 2.442721447 | 9.270825354 | 15.60264037 | 5.95E-31 | 1.95E-29 | 59.70370388 |
| CFAP251 | 2.446525558 | 7.359148377 | 13.59007436 | 2.93E-26 | 5.87E-25 | 48.91057358 |
| CNFN | 2.457849668 | 11.3425119 | 29.50989387 | 1.11E-57 | 8.28E-55 | 121.1005176 |
| MXD1 | 2.467124069 | 8.550627549 | 25.27608467 | 1.45E-50 | 4.64E-48 | 104.7964127 |
| CD274 | 2.479888421 | 6.891146254 | 25.21356648 | 1.87E-50 | 5.73E-48 | 104.5413615 |
| TTC39A | 2.487370328 | 7.281038315 | 29.90594227 | 2.63E-58 | 2.29E-55 | 122.5336073 |
| HAS3 | 2.499477386 | 7.964950954 | 20.02557175 | 1.53E-40 | 1.42E-38 | 81.76791602 |
| TGM1 | 2.500211756 | 9.747889726 | 29.14507805 | 4.25E-57 | 2.84E-54 | 119.7671896 |
| GZMB | 2.501864354 | 6.248106946 | 20.33089857 | 3.64E-41 | 3.65E-39 | 83.19995828 |
| CXCL1 | 2.514139903 | 5.655670419 | 14.51456371 | 1.95E-28 | 4.90E-27 | 53.9185378 |
| SERPINB13 | 2.545892676 | 10.78920931 | 28.32910246 | 8.94E-56 | 5.65E-53 | 116.7380085 |
| A2ML1 | 2.546718353 | 8.818185131 | 21.2512724 | 5.18E-43 | 6.14E-41 | 87.44537729 |
| PRSS27 | 2.555014335 | 6.05103418 | 20.00907691 | 1.65E-40 | 1.52E-38 | 81.69021478 |
| CXCL9 | 2.56423713 | 7.863031543 | 13.97983204 | 3.50E-27 | 7.62E-26 | 51.0313608 |
| TMC5 | 2.570462678 | 8.116376131 | 17.81061725 | 7.15E-36 | 3.98E-34 | 71.02383548 |
| RGS20 | 2.57084866 | 6.888737038 | 25.28089958 | 1.42E-50 | 4.62E-48 | 104.8160376 |
| CXCL13 | 2.578251107 | 5.375295561 | 15.80319377 | 2.08E-31 | 7.19E-30 | 60.75587757 |
| CCNB1 | 2.584748954 | 7.922505974 | 28.10057379 | 2.12E-55 | 1.30E-52 | 115.8777853 |
| TYMP | 2.589747351 | 9.203164817 | 27.30363537 | 4.51E-54 | 2.24E-51 | 112.8364401 |
| CXCL8 | 2.620659746 | 6.085863154 | 11.42703196 | 4.65E-21 | 5.58E-20 | 36.95585881 |
| CCL18 | 2.635244899 | 8.788772533 | 15.65914257 | 4.42E-31 | 1.48E-29 | 60.00059661 |
| SPRR3 | 2.737482955 | 9.539959557 | 26.08939405 | 5.38E-52 | 2.12E-49 | 108.0749219 |
| IL36RN | 2.740364851 | 9.437390796 | 24.80196283 | 1.02E-49 | 2.76E-47 | 102.8511897 |
| RGS1 | 2.781896222 | 6.150302315 | 29.2492488 | 2.90E-57 | 2.01E-54 | 120.1492201 |
| CXCL10 | 2.79730586 | 7.358874093 | 15.41205668 | 1.62E-30 | 5.07E-29 | 58.69963103 |
| HERC6 | 2.802161143 | 8.43684525 | 19.74641622 | 5.72E-40 | 5.02E-38 | 80.44824908 |
| GJB2 | 2.860798669 | 11.76649338 | 35.43282682 | 1.96E-66 | 2.72E-63 | 141.0920731 |
| ZC3H12A | 2.927326571 | 8.531550861 | 29.25753946 | 2.81E-57 | 2.01E-54 | 120.1795798 |
| CCL20 | 2.964483692 | 6.01319055 | 22.15408784 | 8.86E-45 | 1.38E-42 | 91.50692563 |
| IFI27 | 2.989544536 | 10.80626898 | 29.65944329 | 6.44E-58 | 5.17E-55 | 121.643406 |
| ATP12A | 2.995101794 | 7.951345527 | 22.00454282 | 1.73E-44 | 2.55E-42 | 90.84112367 |
| SPRR2G | 3.024034114 | 12.339238 | 21.55357668 | 1.31E-43 | 1.70E-41 | 88.81663136 |
| OAS2 | 3.167226049 | 7.147280649 | 19.73469235 | 6.05E-40 | 5.26E-38 | 80.39260908 |
| MMP12 | 3.17725532 | 6.035651222 | 16.47111339 | 6.44E-33 | 2.69E-31 | 64.22638623 |
| KLK6 | 3.191681515 | 8.871449485 | 16.28124957 | 1.72E-32 | 6.83E-31 | 63.24520025 |
| KLK13 | 3.278525593 | 8.107532405 | 23.95726967 | 3.51E-48 | 7.79E-46 | 99.32203211 |
| SLC6A14 | 3.399294707 | 8.310330261 | 21.61028277 | 1.02E-43 | 1.35E-41 | 89.07258308 |
| IGFL1 | 3.420663726 | 6.818647869 | 22.68918064 | 8.32E-46 | 1.41E-43 | 93.86685319 |
| S100A8 | 3.58818795 | 12.72327174 | 18.88371521 | 3.61E-38 | 2.65E-36 | 76.30715389 |
| TMPRSS11D | 3.615500247 | 7.999853166 | 26.24893965 | 2.85E-52 | 1.19E-49 | 108.7095595 |
| GDA | 3.827545127 | 6.577871084 | 29.5852525 | 8.44E-58 | 6.52E-55 | 121.3743478 |
| CHI3L2 | 3.890697946 | 8.072097832 | 29.95065865 | 2.24E-58 | 2.03E-55 | 122.6944811 |
| RHCG | 3.966321155 | 9.116438636 | 29.13856989 | 4.36E-57 | 2.84E-54 | 119.7432871 |
| ADAMDEC1 | 3.970247236 | 6.543360696 | 31.28412945 | 1.94E-60 | 2.03E-57 | 127.407017 |
| LCN2 | 4.070775222 | 9.230828063 | 25.64034897 | 3.28E-51 | 1.18E-48 | 106.2738128 |
| KRT16 | 4.084055962 | 11.30533005 | 34.2505983 | 8.71E-65 | 1.07E-61 | 137.3344984 |
| HPSE | 4.115816592 | 8.383293116 | 40.08293488 | 1.66E-72 | 3.15E-69 | 154.8919412 |
| LTF | 4.123392639 | 8.970549088 | 24.64449275 | 1.96E-49 | 5.11E-47 | 102.199491 |
| S100A7 | 4.202867798 | 11.84279458 | 21.90689076 | 2.67E-44 | 3.85E-42 | 90.40487328 |
| LCE3D | 4.611936028 | 11.09316554 | 39.8301419 | 3.43E-72 | 5.96E-69 | 154.1790964 |
| KYNU | 4.665010329 | 8.29381359 | 47.32063068 | 7.64E-81 | 2.66E-77 | 173.7293212 |
| C10orf99 | 5.007760644 | 9.22832623 | 47.65020045 | 3.40E-81 | 1.42E-77 | 174.5212685 |
| IL36G | 5.190572101 | 9.114585558 | 50.19918737 | 7.59E-84 | 3.96E-80 | 180.4745752 |
| SERPINB3 | 5.367419073 | 11.01641212 | 35.43854833 | 1.92E-66 | 2.72E-63 | 141.1099976 |
| AKR1B10 | 5.82662834 | 8.708583531 | 66.44295799 | 2.66E-98 | 2.78E-94 | 212.5329528 |
| S100A12 | 5.842720257 | 7.491346577 | 44.68066928 | 6.10E-78 | 1.59E-74 | 167.1899837 |
| S100A9 | 5.849416407 | 11.46109745 | 41.51346565 | 2.98E-74 | 6.90E-71 | 158.8510152 |
| SPRR2C | 5.860379707 | 7.190119726 | 36.98158984 | 1.58E-68 | 2.54E-65 | 145.8556588 |
| TCN1 | 6.216941899 | 8.532529611 | 40.78460515 | 2.28E-73 | 4.75E-70 | 156.8495589 |
| PI3 | 7.042829493 | 9.827143798 | 94.26097271 | 1.23E-116 | 2.56E-112 | 251.7183221 |
| S100A7A | 7.230665031 | 8.380011574 | 66.21883544 | 3.99E-98 | 2.78E-94 | 212.1480147 |
| SERPINB4 | 8.565018504 | 9.015851687 | 47.12870343 | 1.23E-80 | 3.66E-77 | 173.2656985 |
