# Supplementary material for: Exploration of Biomarkers of Psoriasis through Combined Multiomics Analysis
Source: Mediators Inflamm. 2022 Sep 23;2022:7731082. doi: 10.1155/2022/7731082 (PMC9525798; doi:10.1155/2022/7731082)
Supplement: Supplementary Materials — Supplementary Figure 1 The PCA of gene expression in psoriasis lesions and healthy controls in GSE13355 database. Supplementary Figure 2 The PCA and methylation distribution density in psoriasis lesions and healthy controls from the GSE73894 dataset. (A) PCA in GSE73894. (B) Methylation distribution density in GSE73894. Supplementary Table 1 Identification of DEGs in the psoriatic lesions and healthy control group in GSE13355. Supplementary Table 2 GO analysis on 767 DEGs in GSE13355. Supplementary Table 3 KEGG analysis on 767 DEGs in GSE13355. Supplementary Table 4 Identification of hyper-MR-genes. Supplementary Table 5 Identification of hypo-MR-genes. Supplementary Table 6 GO analysis of hyper-MR-genes. Supplementary Table 7 GO analysis of hypo-MR-genes. Supplementary Table 8 KEGG analysis of hyper-MR-genes. Supplementary Table 9 KEGG analysis of hypo-MR-genes. Supplementary Table 10 GO analysis through single-gene GSEA of GJB2. Supplementary Table 11 KEGG analysis through single-gene GSEA of GJB2. [file 7731082.f1.zip › Supplementary Table 10 (1).docx]

| GO analysis through single gene GSEA of GJB2 | | | | | | | | | | |
| --- | --- | --- | --- | --- | --- | --- | --- | --- | --- | --- |
| ID | Description | setSize | enrichmentScore | NES | pvalue | p.adjust | qvalues | rank | leading edge | core enrichment |
| GOBP | CYCLIC NUCLEOTIDE BIOSYNTHETIC PROCESS | 22 | -0.528470324 | -1.645693711 | 0.011485999 | 0.049975999 | 0.034909043 | 4263 | tags=36%, list=20%, signal=29% | GUCY1B1/NPR1/ADCY6/NPR2/ADCY9/GUCY1A2/ADCY4/ADCY2 |
| GOBP | POSITIVE REGULATION OF B CELL MEDIATED IMMUNITY | 37 | 0.488978484 | 1.627575082 | 0.011484067 | 0.049975999 | 0.034909043 | 5845 | tags=59%, list=28%, signal=43% | NOD2/EXOSC3/NSD2/MAD2L2/TFRC/HMCES/HLA-E/TNF/XCL1/NECTIN2/TNFSF13/PTPRC/CD28/CD40/TBX21/MLH1/KMT5C/CLCF1/LTA/TP53BP1/CD226/PAXIP1 |
| GOBP | ALPHA AMINO ACID BIOSYNTHETIC PROCESS | 63 | 0.411648157 | 1.525086511 | 0.011430029 | 0.049796975 | 0.034783992 | 5787 | tags=54%, list=28%, signal=39% | GOT2/GOT1/PSPH/AASS/SHMT2/MTHFD1/ASL/MTRR/ADI1/NAGS/GLUL/DHFR/PHGDH/PARK7/SERINC5/PSAT1/ILVBL/PLOD2/ASNS/MTHFR/CAD/AASDHPPT/ENOPH1/SLC25A12/ALDH18A1/PYCR1/MTHFD2L/PYCR3/CLN3/AGXT2/SDSL/SDS/APIP/SEPHS2 |
| GOMF | PHOSPHATASE INHIBITOR ACTIVITY | 45 | -0.43388252 | -1.613074225 | 0.011380812 | 0.049614726 | 0.034656688 | 2818 | tags=29%, list=14%, signal=25% | TESC/ANP32E/SET/PPP1R1B/PPP1R14A/PTN/PPP1R1A/SH3RF2/PHACTR3/PPP1R36/PABIR1/PPP1R14C/LGALS3 |
| GOBP | METANEPHROS MORPHOGENESIS | 31 | -0.482317803 | -1.641426575 | 0.01136229 | 0.049566147 | 0.034622755 | 3438 | tags=39%, list=16%, signal=32% | LGR4/FRAS1/BMP4/WNT7B/HES1/PDGFRB/SOX8/FMN1/LIF/GREM1/PKD2/PKD1 |
| GOBP | SEMI LUNAR VALVE DEVELOPMENT | 41 | -0.449690494 | -1.62925065 | 0.011318451 | 0.049406987 | 0.034511579 | 4512 | tags=39%, list=22%, signal=31% | HEY1/TGFB2/BMP2/ELN/BMP4/ROBO2/JAG1/SLIT2/EFNA1/TWIST1/SLIT3/ROCK2/RBPJ/NFATC1/NOTCH2/GATA3 |
| GOBP | NON CANONICAL WNT SIGNALING PATHWAY | 149 | 0.342411771 | 1.44501946 | 0.011305172 | 0.049381109 | 0.034493503 | 2155 | tags=26%, list=10%, signal=23% | PSME2/PSMB2/WNT5A/PSMA5/PSMB5/FZD5/AP2S1/PSMA3/PSMD12/PSME4/PSMA2/PSME1/GRHL3/PSMB6/PSMB10/PSMB3/PSMD6/PSMB8/PSMC4/PSMA4/PSMD2/PSMD11/PSMD1/PSMD14/PSMC3/PSMB9/PSMA7/PSMD9/PSMD8/PSMA1/PSMD13/PSMF1/PSMC5/PSMC2/PSMB1/PSMC6/PSME3/PSMD7 |
| GOBP | BLOOD VESSEL ENDOTHELIAL CELL MIGRATION | 113 | -0.328030745 | -1.433020807 | 0.011259128 | 0.049211986 | 0.034375368 | 4235 | tags=40%, list=20%, signal=32% | KDR/MIA3/HDAC9/HDAC5/PRKD2/ANGPT1/SCARB1/MAP2K5/FOXC2/NRP1/VEGFC/EGR3/SIRT1/ABL1/SRPX2/JCAD/PRKD1/RGCC/TMSB4X/AMOT/STAT5A/CLEC14A/SLIT2/SPRED1/PRKCA/MMRN2/EFNA1/PPARG/ACVRL1/APOE/GREM1/MECP2/KLF4/FGFR1/MEF2C/AKT3/RHOJ/STARD13/MEOX2/ATP2B4/FBXW7/SP1/GATA2/MAP3K3/EFNB2 |
| GOBP | POSITIVE REGULATION OF CELL GROWTH | 152 | 0.325619266 | 1.375458428 | 0.011204647 | 0.049005741 | 0.034231302 | 3427 | tags=26%, list=16%, signal=22% | S100A9/S100A8/CXCR4/CRABP2/EXOSC4/RAB11A/KRT17/SLC23A2/PRSS2/CXCL16/HBEGF/NCBP1/SFN/ADAM17/DBNL/RASAL1/SEMA7A/DNPH1/SLC25A33/RPS6KA1/CSNK2A1/CIB1/DERL2/DDX49/PLAA/ITSN2/EXOSC2/ADAM10/CEP43/KDM2B/NDEL1/EIF4G2/CYFIP1/FXN/EIF4G1/PSMD10/LPAR3/SEMA4D/CD38 |
| GOBP | REGULATION OF CELL CYCLE CHECKPOINT | 30 | 0.511937315 | 1.615183012 | 0.011146515 | 0.048783249 | 0.034075888 | 3136 | tags=40%, list=15%, signal=34% | CCNB1/NDC80/MAD2L1/CDT1/GEN1/MAD2L1BP/WDR76/CHEK2/LCMT1/THOC5/FBXO4/RFWD3 |
| GOMF | CYCLIC NUCLEOTIDE GATED ION CHANNEL ACTIVITY | 10 | -0.676936927 | -1.695418733 | 0.011106111 | 0.048638104 | 0.033974502 | 838 | tags=30%, list=4%, signal=29% | CNGA4/CNGA1/AQP1 |
| GOBP | ACTIN FILAMENT REORGANIZATION | 10 | -0.676056925 | -1.69321473 | 0.011106111 | 0.048638104 | 0.033974502 | 3727 | tags=80%, list=18%, signal=66% | ARAP1/WHAMM/DMTN/NEDD9/MCU/EFS/GDPD2/GSN |
| GOBP | METENCEPHALON DEVELOPMENT | 99 | -0.334166429 | -1.440648618 | 0.010979431 | 0.048146095 | 0.033630825 | 3432 | tags=38%, list=16%, signal=32% | PTPN11/AGTPBP1/PAK1/FCGR2B/OPHN1/DLL1/ATP7A/SEMA4C/NCOA1/TTLL1/KAT2A/TRNP1/PTBP2/CNTN1/ABL1/ND4/CBLN1/COX1/ZNF365/EN1/GNPAT/LRP6/PTPRS/FOXC1/MECP2/SEC24B/PTN/PPARGC1A/HERC1/TTBK2/RERE/FZD4/CKB/LPAR1/SPTBN2/GLI2/BCL2/RORA |
| GOBP | BASEMENT MEMBRANE ASSEMBLY | 15 | -0.605869729 | -1.705686035 | 0.010936555 | 0.047989404 | 0.033521374 | 4535 | tags=67%, list=22%, signal=52% | NTNG2/PLOD3/PHLDB2/RAMP2/NTN4/CLASP1/LAMB2/PHLDB1/DAG1/LAMB4 |
| GOMF | CYCLIN DEPENDENT PROTEIN SERINE THREONINE KINASE REGULATOR ACTIVITY | 47 | 0.450155992 | 1.573716939 | 0.010908725 | 0.047898573 | 0.033457927 | 4080 | tags=45%, list=20%, signal=36% | CCNB1/CCNB2/CKS2/CCNE1/CCNA2/CCNE2/CCNC/CCNF/CDKN1A/CKS1B/CCND2/CCNO/CCNQ/CDKN2A/CCNH/CASP3/CCND3/CDKN2D/CDK4/CCNYL1/CCNK |
| GOBP | POSITIVE REGULATION OF LEUKOCYTE APOPTOTIC PROCESS | 24 | 0.545166435 | 1.642715829 | 0.010882092 | 0.047812882 | 0.033398071 | 3143 | tags=54%, list=15%, signal=46% | CD274/WNT5A/PRELID1/IDO1/LYN/ZC3H8/LGALS9/ADAM8/PIK3CD/CCL5/CDKN2A/BAX/ANXA1 |
| GOMF | CARBON NITROGEN LYASE ACTIVITY | 13 | 0.643473443 | 1.667168493 | 0.010868989 | 0.047786564 | 0.033379687 | 1143 | tags=46%, list=5%, signal=44% | CHAC1/CHAC2/GGCT/ASL/HAL/ADSL |
| GOBP | MIRNA METABOLIC PROCESS | 29 | 0.523506916 | 1.642781444 | 0.010839596 | 0.047688542 | 0.033311217 | 2357 | tags=31%, list=11%, signal=28% | ZC3H12A/RAN/PNPT1/TARBP2/NFKB1/TUT7/HRAS/SND1/XPO5 |
| GOBP | POSITIVE REGULATION OF INTRACELLULAR PROTEIN TRANSPORT | 176 | 0.329160252 | 1.408781605 | 0.010776019 | 0.047439904 | 0.03313754 | 3991 | tags=34%, list=19%, signal=28% | ZC3H12A/RAN/TFDP1/GZMB/FZD5/NMT1/UBE2L3/YWHAQ/ZPR1/UBE2D3/SFN/ECT2/TMEM30B/IFNG/BCAP31/IL1B/UBL5/ATG13/BAG3/PDCD10/BID/CDK5/MTCL1/PDCD5/TCAF2/TMEM30A/EDEM1/RAC2/KIF20B/PLK3/ITGB2/CIB1/SAE1/HRAS/CASP8/TP63/TARDBP/YWHAZ/ARIH2/PIK3R2/EDEM2/UBE2J2/HCLS1/SREBF2/OAZ1/MFF/HTRA2/MIEF1/PRKCD/RIOK2/NUTF2/MAPK8/CEMIP/HDAC3/GSK3A/CACNB3/EMD/CDH1/JUP/MAPK14 |
| GOBP | HISTONE PHOSPHORYLATION | 39 | 0.46582586 | 1.562530497 | 0.010762561 | 0.04741173 | 0.03311786 | 3348 | tags=38%, list=16%, signal=32% | CCNB1/CDK1/AURKB/MACROH2A1/AURKA/CCNA2/ZMPSTE24/IL1B/CDK5/RPS6KA4/HASPIN/VRK1/CDK2/CDK9/PRKCD |
| GOBP | LYMPH NODE DEVELOPMENT | 17 | 0.59739754 | 1.666897385 | 0.010753312 | 0.047402065 | 0.033111109 | 3543 | tags=47%, list=17%, signal=39% | POLB/LTB/SPNS2/IL7R/RIPK3/FADD/PDPN/IL15 |
| GOBP | VASCULAR ASSOCIATED SMOOTH MUSCLE CELL DIFFERENTIATION | 26 | -0.499618337 | -1.628604383 | 0.010698004 | 0.047189224 | 0.032962435 | 3852 | tags=46%, list=18%, signal=38% | RAMP2/GPER1/EFEMP2/HES1/FGF9/NFATC2/MYOCD/PDCD4/SGCB/NFATC1/EPC1/KIT |
| GOBP | MRNA 3 END PROCESSING | 94 | 0.380888885 | 1.499861896 | 0.010657627 | 0.04704201 | 0.032859604 | 3669 | tags=36%, list=18%, signal=30% | CCNB1/EIF4A3/DDX39A/NCBP1/SRSF9/PNPT1/CPSF6/CPSF3/ALYREF/MAGOHB/CSTF2/SARNP/LEO1/SRSF2/POLR2D/SRSF7/SRSF1/GRSF1/THOC5/CLP1/PAF1/THOC6/THOC7/SNRPA/NELFE/CPSF4/SSU72/CSTF1/MAGOH/CDK9/WDR33/CTR9/CHTOP/CSTF3 |
| GOBP | NEGATIVE REGULATION OF ANOIKIS | 17 | -0.580446566 | -1.682401728 | 0.010621246 | 0.046912248 | 0.032768964 | 3677 | tags=59%, list=18%, signal=48% | PIK3CA/ITGA5/ZNF304/PTK2/ITGB1/NTRK2/PDK4/BCL2/CAV1/TLE1 |
| GOBP | OSTEOBLAST DEVELOPMENT | 16 | -0.607359752 | -1.724588688 | 0.010546624 | 0.046613301 | 0.032560144 | 4526 | tags=56%, list=22%, signal=44% | HDAC4/LRP5L/LRP5/TNN/PTH1R/JUND/SATB2/SMAD3/GLI2 |
| GOBP | REGULATION OF REACTIVE OXYGEN SPECIES METABOLIC PROCESS | 177 | 0.316949366 | 1.357870853 | 0.010537307 | 0.046602785 | 0.032552798 | 4330 | tags=35%, list=21%, signal=28% | ZC3H12A/GCH1/HK2/CLEC7A/FOXM1/TIGAR/STK17A/GSTP1/HIF1A/EIF6/RAB27A/DUOXA1/CFLAR/TUSC2/IFNG/ACE2/CLCN3/TRAP1/IL1B/ATG5/XDH/ROMO1/SLC25A33/CDKN1A/CD177/SOD2/SYK/DYNLL1/RAC2/RIPK3/CD36/ITGB2/GNAI3/CTNS/DHFR/VDAC1/BNIP3/GLA/PARK7/CYBA/BRCA1/BCO2/TNF/GADD45A/G6PD/TSPO/ICAM1/TYROBP/PRKCD/BECN1/P2RX4/PON3/HP/MTOR/IL10/MAPK14/PTX3/MPV17/MMP3/ARF4/PLIN5/DUOXA2 |
| GOBP | CHAPERONE COFACTOR DEPENDENT PROTEIN REFOLDING | 30 | 0.514949779 | 1.62468746 | 0.010516775 | 0.046542619 | 0.032510772 | 4203 | tags=57%, list=20%, signal=45% | HSPA5/ERO1A/HSPA14/HSPA9/HSPE1/HSPA8/HSPH1/TOR1A/DNAJB1/SDF2L1/DNAJC7/TOR2A/PTGES3/TOR1B/DNAJB5/DNAJB12/SDF2 |
| GOBP | POSITIVE REGULATION OF NATURAL KILLER CELL MEDIATED IMMUNITY | 27 | 0.534742493 | 1.661626593 | 0.010516775 | 0.046542619 | 0.032510772 | 3933 | tags=52%, list=19%, signal=42% | RAET1E/IL12B/RASGRP1/IL18RAP/SH2D1A/LAG3/HLA-E/NCR3/CRTAM/KLRD1/HLA-F/NECTIN2/HLA-G/VAV1 |
| GOBP | POSITIVE REGULATION OF TELOMERE MAINTENANCE VIA TELOMERE LENGTHENING | 35 | 0.481370606 | 1.594142977 | 0.010488172 | 0.046477271 | 0.032465125 | 2945 | tags=43%, list=14%, signal=37% | PRKCQ/AURKB/CCT5/NEK2/CCT2/HNRNPA2B1/CCT3/CCT7/PNKP/XRCC5/TCP1/DKC1/FBXO4/CCT6A/CCT8 |
| GOBP | METANEPHRIC NEPHRON MORPHOGENESIS | 25 | -0.518135289 | -1.673038617 | 0.010473042 | 0.046440857 | 0.03243969 | 3438 | tags=40%, list=16%, signal=33% | LGR4/BMP4/HES1/PDGFRB/SOX8/FMN1/LIF/GREM1/PKD2/PKD1 |
| GOCC | POLYSOMAL RIBOSOME | 31 | -0.48504009 | -1.650691079 | 0.010467641 | 0.046440857 | 0.03243969 | 4610 | tags=48%, list=22%, signal=38% | RPL19/NUFIP2/RPL24/RPL41/RPL32/RPL7A/RPL31/RPL18/RPL10A/RPL11/RPL8/RPS23/EIF3H/RPL38/RPL30 |
| GOBP | REGULATION OF ATP METABOLIC PROCESS | 114 | 0.351594262 | 1.429161914 | 0.010452009 | 0.046408854 | 0.032417334 | 4881 | tags=40%, list=23%, signal=31% | ENO1/PGAM1/PPIF/TIGAR/VCP/HIF1A/EIF6/SHMT2/NUP37/NUP210/NUP88/IFNG/COX7A2P2/SLC25A33/PDE12/UQCC2/NUP50/COX7A2/DNAJC15/RAE1/SEC13/ANTKMT/PFKFB2/NUP155/TREM2/NDC1/PARP1/PFKFB4/TSPO/NUP188/PRKAG1/BEND3/NUP85/NUP205/NUP58/NUP107/NUP93/DNAJC30/ATP5IF1/POM121/SLC25A12/NUP160/ZBTB7A/NUP35/NUP42/ENTPD5 |
| GOBP | RESOLUTION OF MEIOTIC RECOMBINATION INTERMEDIATES | 18 | 0.596911783 | 1.692746637 | 0.010443842 | 0.04640326 | 0.032413427 | 6026 | tags=56%, list=29%, signal=40% | CENPX/TOP2A/SHOC1/CENPS/EME1/EME2/MLH1/TEX11/CORT/RMI1 |
| GOBP | REGULATION OF NEUROTROPHIN TRK RECEPTOR SIGNALING PATHWAY | 12 | -0.645357285 | -1.709933333 | 0.010404189 | 0.046257671 | 0.032311731 | 2962 | tags=50%, list=14%, signal=43% | AGT/NTF3/ZDHHC17/TMEM108/SPRY1/SPRY2 |
| GOBP | REGULATION OF B CELL MEDIATED IMMUNITY | 53 | 0.434200074 | 1.563237303 | 0.010398315 | 0.046257671 | 0.032311731 | 6065 | tags=57%, list=29%, signal=40% | NOD2/SUSD4/EXOSC3/NSD2/MAD2L2/TFRC/HMCES/HLA-E/TNF/XCL1/NECTIN2/IL10/FOXP3/PARP3/CR1/TNFSF13/PTPRC/CD28/CD40/TBX21/SLC15A4/C4BPA/MLH1/KMT5C/CLCF1/LTA/TP53BP1/CD226/PAXIP1/FOXJ1 |
| GOBP | REGULATION OF LYMPHOCYTE APOPTOTIC PROCESS | 53 | 0.434526303 | 1.564411817 | 0.010398315 | 0.046257671 | 0.032311731 | 3006 | tags=40%, list=14%, signal=34% | CD274/WNT5A/PRELID1/PRKCQ/AURKB/HIF1A/IDO1/JAK3/LYN/ZC3H8/IL7R/RIPK3/LGALS9/CD27/DOCK8/NOC2L/CD3G/ADAM8/FADD/CCL5/BAX |
| GOCC | CELL CORTEX | 293 | -0.264249754 | -1.311619035 | 0.01038599 | 0.046257671 | 0.032311731 | 3272 | tags=26%, list=16%, signal=22% | SPRR4/FGF1/MYRIP/CTTNBP2/SEPTIN2/PXN/WASHC1/KRT19/NDFIP1/CCN2/EXOC6B/LASP1/TRAK1/SEPTIN6/CLASP1/FRYL/PARD6G/ASPH/CLIC5/RHOBTB1/RAPGEF3/NEDD9/EEF1A1/CLDN5/FER/CRIP2/PLEKHH2/SEPTIN10/DSTN/EPB41L2/FNBP1L/PTK2/DST/FGFR2/AKAP12/SPTAN1/NUMA1/RHOB/SEPTIN7/SEPTIN11/PKD2/EPB41/PHLDB1/CALD1/SNCA/MLPH/AKAP13/PRKCB/SCIN/FERMT2/LAMC2/WDPCP/MKLN1/PAFAH1B1/TSC1/PARD3B/PARD3/EPS8/SPTBN2/MYH10/GNAI1/MYADM/ADD3/DLC1/DBN1/FLNB/ARHGEF7/TMOD1/GSN/CAV1/RHOBTB3/STXBP6/SPTBN1/UTRN/COBL/RAI14 |
| GOMF | NUCLEOSOMAL DNA BINDING | 38 | 0.47446008 | 1.589196676 | 0.010385032 | 0.046257671 | 0.032311731 | 6389 | tags=58%, list=31%, signal=40% | HDAC1/MACROH2A1/H2AZ1/HNRNPC/RCC1/HMGN1/HDAC2/MBD2/SMARCA4/ACTL6A/H1-2/MBD3/H1-5/RBBP4/H1-3/H1-1/MTA2/HMGN2/ACTB/H1-9P/H1-4/H1-6 |
| GOCC | AMINOACYL TRNA SYNTHETASE MULTIENZYME COMPLEX | 11 | 0.673588839 | 1.671615087 | 0.010381883 | 0.046257671 | 0.032311731 | 1248 | tags=55%, list=6%, signal=51% | MARS1/IARS1/AIMP1/EEF1E1/AIMP2/RARS1 |
| GOCC | CILIARY TIP | 47 | -0.422540647 | -1.593458555 | 0.010327093 | 0.046097948 | 0.032200161 | 5948 | tags=60%, list=29%, signal=43% | KIF7/TTC30B/GLI1/KIF3C/IFT88/IFT81/CYLD/IFT140/KIF3A/IFT74/DYNC2LI1/IFT27/IFT172/IFT52/TTC21B/DYNC2H1/DYNC2I1/TRAF3IP1/DYNLL2/IFT22/CLUAP1/DYNLRB2/IFT122/WDR35/WDR19/GLI2/GLI3/DYNLRB1 |
| GOMF | WNT ACTIVATED RECEPTOR ACTIVITY | 15 | -0.608120481 | -1.712022508 | 0.010258333 | 0.045821464 | 0.032007033 | 2825 | tags=73%, list=14%, signal=63% | LRP5/FZD1/FZD3/ROR1/FZD10/FZD8/FZD7/RYK/LRP6/FZD4/PKD1 |
| GOBP | POSITIVE REGULATION OF MYELOID LEUKOCYTE MEDIATED IMMUNITY | 17 | 0.598899017 | 1.671086904 | 0.010238189 | 0.045761913 | 0.031965436 | 3330 | tags=53%, list=16%, signal=45% | PTAFR/ARG1/CD177/ITGB2/HLA-E/DDX21/DDX58/TYROBP/STX4 |
| GOBP | POSITIVE REGULATION OF PHAGOCYTOSIS | 61 | 0.423994901 | 1.556039577 | 0.010181747 | 0.045539931 | 0.031810378 | 4673 | tags=44%, list=22%, signal=34% | NOD2/CLEC7A/PYCARD/RAB27A/LMAN2/IFNG/RAB31/IL1B/CCL2/LYAR/CD36/IL2RG/C2/SIRPG/CYBA/SFTPD/TNF/TREM2/IL15/DOCK2/PTX3/IL15RA/CD300LF/ABCA7/PTPRC/DNM2/FCER1G |
| GOBP | MEMBRANE LIPID METABOLIC PROCESS | 204 | 0.314357406 | 1.371738579 | 0.010168572 | 0.045511305 | 0.031790382 | 4131 | tags=33%, list=20%, signal=27% | GM2A/SPTSSA/CERS3/FUT3/SPTLC2/FUT2/ORMDL2/PPP2CA/SMPD3/ALOX12B/VAPA/CWH43/B3GNT5/PIGA/ELOVL7/CYP4F22/GBA2/GLTP/PIGW/SPNS2/PIGX/PIGO/NAGA/ALDH3B2/SMPD1/CSNK1G2/HACD1/CTSA/SERINC2/SMPD2/GLA/ARV1/ST8SIA4/SAMD8/TNFRSF1A/ARSA/SGPP2/DPM2/PIGB/MGST2/TNF/NEU2/STS/UGCG/CPTP/BAX/SERINC5/HTRA2/ELOVL4/ESYT3/AGK/PIGL/CLN6/DPM1/PRKCD/PPP2R1A/SPTLC1/SPTLC3/ST3GAL4/ELOVL1/PIGU/SPHK1/ACER3/PIGF/NEU1/PIGN/PLPP2 |
| GOMF | 2 IRON 2 SULFUR CLUSTER BINDING | 21 | 0.569822258 | 1.660708233 | 0.010144511 | 0.045433883 | 0.031736301 | 4677 | tags=62%, list=22%, signal=48% | CIAPIN1/UQCRFS1/SDHB/NDUFV2/XDH/CISD3/GLRX2/FECH/CISD1/FXN/NDUFS1/CISD2/FDX2 |
| GOBP | GLOMERULAR EPITHELIAL CELL DEVELOPMENT | 13 | -0.653968584 | -1.780843444 | 0.010134951 | 0.045421347 | 0.031727545 | 3069 | tags=69%, list=15%, signal=59% | BMP4/JAG1/ASXL1/ADIPOQ/PODXL/LAMB2/IQGAP1/MAGI2/NOTCH2 |
| GOBP | REGULATION OF DENDRITIC CELL DIFFERENTIATION | 12 | 0.658721723 | 1.669805833 | 0.010132327 | 0.045421347 | 0.031727545 | 4776 | tags=75%, list=23%, signal=58% | CEBPB/LGALS9/LILRB2/TMEM176B/HLA-B/LILRB1/HLA-G/AGER/TMEM176A |
| GOCC | UBIQUITIN LIGASE COMPLEX | 283 | 0.299077764 | 1.349044187 | 0.010121724 | 0.045421347 | 0.031727545 | 4277 | tags=34%, list=20%, signal=27% | CDC20/DCUN1D5/UBE2C/UBE2L6/UBE2N/BUB1B/CKS2/KCTD5/TNFAIP1/UBE2L3/FBXO6/FBXO45/MAEA/ELOC/DCAF13/DTL/TSPAN17/ELOB/UBE2S/ANAPC7/FBXO9/FBXW11/CCNF/CBX2/RNF11/SKP2/TRIM21/UBE2D1/UBXN8/KBTBD8/RBX1/UBE2A/GLMN/DCAF11/PDCD6/CKS1B/DCAF12/COMMD1/MAD2L2/TRPC4AP/DERL2/RNF168/TRAF7/UBE3D/ATG3/IKBKG/AUP1/CUL1/BRCC3/FBXL19/SUGT1/FBXW5/BABAM2/FBXO25/ANAPC11/RAD18/RANBP9/RNF7/ARIH2/UBE2D2/MED31/DMAC2/FBXO10/RANBP10/BRCA1/UBE2J2/WDR26/FBXO4/GPR37/KEAP1/FBXL6/PLRG1/ERCC8/RNF8/CAND1/CDC23/LMO7/UBE2E1/DCUN1D3/KLHL2/DDA1/RNF19B/ZSWIM6/OTULIN/RNF144B/DCAF1/MIB2/MED20/TRAF2/ZYG11A/MED7/FBXO38/RBCK1/CDC26/MED10 |
| GOBP | POSITIVE REGULATION OF RNA POLYMERASE II TRANSCRIPTION PREINITIATION COMPLEX ASSEMBLY | 10 | 0.692310787 | 1.67186885 | 0.01011433 | 0.045419834 | 0.031726488 | 3132 | tags=60%, list=15%, signal=51% | PSMC4/PSMC3/PSMC5/PSMC2/PSMC6/CAND1 |
| GOBP | REGULATION OF FIBROBLAST MIGRATION | 34 | -0.482395913 | -1.682484838 | 0.010005943 | 0.044963161 | 0.031407495 | 4046 | tags=44%, list=19%, signal=36% | APPL1/DMTN/PRKCE/GNA12/DDR2/MACIR/FER/PTK2/AKAP12/ITGB1/SLC8A1/SDC4/WDPCP/PAK3/ARHGEF7 |
| GOBP | MUSCLE CELL PROLIFERATION | 176 | -0.293996308 | -1.377609886 | 0.010000794 | 0.044963161 | 0.031407495 | 4184 | tags=38%, list=20%, signal=31% | S1PR1/CTNNB1/VIP/CCN4/PDE1A/TGFB2/KCNK2/MMP2/ELN/ANGPT1/GPER1/MSTN/SIX5/EFEMP2/SAV1/PTEN/FOXC2/VGLL4/NPR1/IL18/ERBB4/BMP4/JARID2/PDGFRB/OGN/YAP1/EDN1/NOG/ADIPOQ/CCN3/APOD/FGF9/PPARG/EGFR/NPY5R/ABCC4/FOXC1/HPGD/FGFR2/KLF4/BMPR1A/PDGFD/DIPK2A/MEF2C/RBP4/PPARGC1A/IGFBP5/CDKN1B/MYOCD/MEF2D/WNT2/RBPMS2/PRKG1/RBPJ/MAPK1/TGFBR2/ZFPM2/EPHB1/PDCD4/CNN1/FOXJ2/TCF7L2/CTNNBIP1/TPM1/MEIS1/TGFBR3/ANG |
| GOBP | NEUTRAL AMINO ACID TRANSPORT | 42 | 0.474513006 | 1.619698938 | 0.009952293 | 0.044781987 | 0.031280942 | 5592 | tags=62%, list=27%, signal=45% | SLC6A14/SFXN1/SLC7A5/ACE2/SLC38A5/NFKBIE/SLC36A1/SFXN2/SLC6A15/SLC3A2/CTNS/SLC38A9/SLC38A7/SLC6A20/SLC36A4/SLC43A2/SLC1A1/SLC1A5/SLC1A4/LLGL2/SLC6A19/SLC6A6/SLC38A6/SLC7A8/RGS4/SLC6A17 |
| GOBP | RECEPTOR SIGNALING PATHWAY VIA STAT | 160 | 0.33946715 | 1.446471994 | 0.00994215 | 0.04476633 | 0.031270005 | 4913 | tags=38%, list=24%, signal=29% | STAT1/NMI/PARP9/SOCS3/PPP2CA/IL20/PTPN2/BCL3/PARP14/STAT3/JAK3/IFNG/IL26/CCL2/LYN/CDK5/SOCS1/ADIPOR1/IL12B/STAP2/IL7R/CRLF3/IL6/STAMBP/IL24/IL23A/STAT2/PTK6/TSLP/TNFRSF1A/DOT1L/HCLS1/CCL5/HDAC2/TNF/OCIAD2/GADD45A/IFNAR2/CCR2/CTR9/PPP2R1A/IL15/IL10RA/PIGU/CD300A/IL10/CRLF2/EPO/PWP1/PTPRC/PRL/MGAT5/PTPRD/SH2B3/CD40/IL10RB/IFNE/NF2/IFNA2/HGS |
| GOBP | POSITIVE REGULATION OF HUMORAL IMMUNE RESPONSE | 19 | 0.59207993 | 1.700594914 | 0.009903794 | 0.044623532 | 0.031170258 | 4406 | tags=47%, list=21%, signal=37% | NOD2/CCR7/PHB/IL1B/KLK7/TNF/IL17F/CR1/PTPRC |
| GOBP | VENTRAL SPINAL CORD INTERNEURON DIFFERENTIATION | 12 | -0.646759314 | -1.713648136 | 0.009896679 | 0.044621402 | 0.03116877 | 2742 | tags=33%, list=13%, signal=29% | LMO4/GATA2/GLI2/GLI3 |
| GOBP | REGULATION OF TRANSPORTER ACTIVITY | 267 | -0.260007025 | -1.269671341 | 0.009891917 | 0.044621402 | 0.03116877 | 5098 | tags=35%, list=24%, signal=27% | FGF12/ATP1B2/STRIT1/ANO9/UBQLN1/KCNIP2/HRC/GSTM2/GRIA2/CTTNBP2NL/CAMK2D/ATP7A/FGF11/ACTN4/CASQ1/SCN1B/AKAP6/SLN/PDZK1/STAC2/CHRM3/RGN/CFTR/TRPC6/ABCB1/RASGRF2/AMIGO1/DAPK1/THADA/VAMP2/PTEN/NEDD4L/NOS1/SHANK2/PRKCE/KLHL24/PTPN3/RELN/KCTD7/TCAF1/PM20D1/CALM2/ALG10B/DLG1/APP/KCNAB1/NDFIP1/GAL/CLTRN/TMSB4X/HECW2/TESC/GOPC/CRACR2A/WNK1/ADIPOQ/NLGN1/CACNB2/TWIST1/FXYD1/FGF13/PPARG/JPH2/SHANK3/ANK2/ITGB1/PKD2/MEF2C/PPARGC1A/SNCA/PINK1/STAC/PLN/AKAP9/OSR1/SCN3B/WNK2/ATP1A2/NDFIP2/KMT2A/ADRA2A/CASQ2/GPD1L/DLG2/CACNA2D1/DMD/AHNAK/FXYD6/BCL2/CAV1/HSPA2/FHL1/ADRB2/UTRN |
| GOMF | MICROTUBULE BINDING | 265 | 0.298792807 | 1.336135852 | 0.00988907 | 0.044621402 | 0.03116877 | 2964 | tags=23%, list=14%, signal=20% | GJB6/S100A9/S100A8/DLGAP5/KIF20A/BIRC5/NUSAP1/KIF2C/KIF18B/MX1/PRC1/KATNB1/KIF4A/TPX2/SPAG5/RAB11A/POLB/KIF2A/KIF11/CRIPT/KIF14/CENPF/CENPE/VAPA/KIF23/SKA2/SKA1/FAM83D/FBXW11/RACGAP1/RGS14/GAPDH/ZNF207/MX2/KIF15/RMDN1/CKAP5/KIF18A/MTCL1/PSRC1/PLK1/RMDN2/KIF20B/NDE1/RCC2/CETN2/REEP4/HOOK1/RAE1/KIF22/MID1/EML6/NDEL1/MAP6D1/KIFC1/MAPRE1/DRG1/CAMSAP1/KIF1B/KIF21B |
| GOMF | INTRAMOLECULAR OXIDOREDUCTASE ACTIVITY | 47 | 0.453195651 | 1.584343396 | 0.009858698 | 0.044539834 | 0.031111794 | 2526 | tags=36%, list=12%, signal=32% | TPI1/ERP44/ERO1A/MIF/P4HB/EBPL/GPI/CYP2S1/EBP/CRELD2/GLRX2/MPI/PTGES2/PDIA4/PDIA6/GNPDA1/QSOX1 |
| GOBP | REGULATION OF RAS PROTEIN SIGNAL TRANSDUCTION | 188 | -0.284962491 | -1.343790571 | 0.009799469 | 0.044302043 | 0.030945693 | 3710 | tags=29%, list=18%, signal=24% | SQSTM1/LPAR4/ALS2/ARHGEF2/MADD/RDX/NRP1/PPP2CB/IQSEC1/RABL3/ABL1/DENND3/RASA4/PDGFRB/KITLG/SHOC2/ARHGAP24/RTN4R/RASA3/CYTH3/NET1/ERBIN/F2R/FBXO8/AUTS2/MET/APOE/OGT/ARHGAP35/ITPKB/PIK3CB/ARHGAP42/STMN3/SCAI/STARD13/AKAP13/ADRA1A/TIMP2/MYOC/RALGPS1/BCL6/LPAR1/EPS8/ARFGEF3/SPRY1/DLC1/NOTCH2/SPRY2/ARHGEF28/ARHGEF10/KANK1/IRS2/KANK2/DENND4C |
| GOBP | REGULATION OF HEART GROWTH | 61 | -0.386400191 | -1.510416082 | 0.00975432 | 0.044127624 | 0.030823859 | 4567 | tags=41%, list=22%, signal=32% | AKAP6/PI16/KCNK2/PARP2/SAV1/PTEN/VGLL4/ERBB4/JARID2/RGS2/YAP1/EDN1/NOG/FGFR2/BMPR1A/PPARA/MEF2C/RBP4/WNT2/RBPJ/MAPK1/TGFBR2/ZFPM2/MEIS1/TGFBR3 |
| GOBP | POLYOL METABOLIC PROCESS | 125 | 0.351797292 | 1.453696975 | 0.009725585 | 0.044027277 | 0.030753764 | 4351 | tags=38%, list=21%, signal=30% | TPI1/GCH1/PTS/GOT1/GK/PTAFR/SPTLC2/IPPK/PLCG2/PLCD1/CYP27B1/ITPKC/IMPA2/INPP1/COQ2/GALK1/PCK2/COQ3/MTMR2/DHFR/PLEK/MINPP1/BPNT1/PLCD4/P2RY1/SGPP2/SYNJ2/AGK/P2RY6/PGP/SPTLC1/SPTLC3/GALR2/PLCB3/CD244/PPIP5K1/CYP2R1/AVPR1B/SPHK1/NTSR1/ACER3/PLD4/PLPP2/SPR/GK2/HRH1/ASAH2B |
| GOBP | SPINDLE LOCALIZATION | 53 | 0.437087048 | 1.573631188 | 0.009716465 | 0.044015652 | 0.030745644 | 3542 | tags=40%, list=17%, signal=33% | NDC80/NUSAP1/MAD2L1/ASPM/DYNLT1/CENPA/FBXW11/ACTR3/ESPL1/PLK1/NDE1/WASL/KPNB1/NDEL1/NSFL1C/GPSM2/CFL1/MYH9/SAPCD2/SPDL1/HTT |
| GOBP | DNA REPLICATION DEPENDENT NUCLEOSOME ORGANIZATION | 26 | 0.543002393 | 1.663757877 | 0.009684299 | 0.043899543 | 0.03066454 | 7159 | tags=77%, list=34%, signal=51% | ASF1B/IPO4/HAT1/NASP/H3C10/CHAF1A/CHAF1B/H3C7/H3C8/H4C4/H4C9/RBBP4/H4C2/H4C5/H3C2/H4C1/H3C11/H3C1/H3C3/H3C12 |
| GOBP | POSITIVE REGULATION OF LYMPHOCYTE CHEMOTAXIS | 18 | 0.600651958 | 1.703353177 | 0.009684299 | 0.043899543 | 0.03066454 | 3616 | tags=67%, list=17%, signal=55% | WNT5A/S100A7/CXCL13/CCL4/ADAM17/CCL7/OXSR1/ADAM10/CCL5/XCL1/CCR2/TMEM102 |
| GOBP | NON MOTILE CILIUM ASSEMBLY | 53 | -0.411324023 | -1.573418545 | 0.009659018 | 0.043844111 | 0.03062582 | 5650 | tags=60%, list=27%, signal=44% | IFT80/BBS4/TMEM107/RPGRIP1L/ARL13B/CEP135/INTU/IFT88/CEP250/IFT140/TMEM216/TTC8/TBC1D32/TOGARAM1/BBS7/IFT74/C2CD3/IFT172/IFT52/CEP350/DYNC2H1/TMEM80/BBS10/CSNK1D/PIBF1/SEPTIN7/BBS1/PCM1/IFT122/CEP126/BBS2/MAP4 |
| GOBP | POSITIVE REGULATION OF TRANSMEMBRANE RECEPTOR PROTEIN SERINE THREONINE KINASE SIGNALING PATHWAY | 101 | -0.338062178 | -1.456286322 | 0.009595472 | 0.04358511 | 0.030444904 | 5601 | tags=42%, list=27%, signal=31% | SMAD2/GDF15/RNF111/ACVR2A/GDF10/FLCN/EP300/SMAD4/CDH5/GDF9/MSX1/TGFB2/ACVR2B/BMP2/BMP6/MSTN/SLC2A10/BMP4/DAB2/HES1/SNW1/ACVRL1/RBPMS/CREBBP/NUMA1/BMPR1A/GDF7/GPC3/NEO1/SDCBP/MYOCD/TGFB1I1/RBPJ/BMP7/FOXD1/ELAPOR2/CDKN1C/ACVR1B/ZNF423/INHBB/NOTCH2/TGFBR3 |
| GOBP | REGULATION OF SNARE COMPLEX ASSEMBLY | 10 | -0.682465972 | -1.709266473 | 0.009580112 | 0.043544786 | 0.030416737 | 4098 | tags=70%, list=20%, signal=56% | TRIM9/ANKRD27/CLTRN/PRRT2/VPS11/SNCA/STXBP6 |
| GOBP | SULFUR COMPOUND METABOLIC PROCESS | 352 | 0.289136224 | 1.326079441 | 0.009559248 | 0.043479369 | 0.030371042 | 4833 | tags=34%, list=23%, signal=26% | KYNU/SQOR/ETHE1/SLC7A11/GLRX3/CHAC1/HYAL4/CIAPIN1/ACOT7/AHCY/CHAC2/GSTP1/GSTO1/CNDP2/ACP3/CIAO2A/AASS/DBI/HSPA9/PDZD11/GSTA4/MTHFD1/ELOVL7/GGCT/NUBP1/SPOCK2/SLC5A6/DGAT1/SLC25A10/HS3ST3B1/TDO2/MTRR/CIAO2B/GPX1/ADI1/PPT2/HSD17B4/GCLM/GLRX2/SULT2B1/NDOR1/ACOT11/CTNS/PTGES2/TST/HACD1/GDAP1/GSTZ1/TPST2/B3GALT6/BPNT1/ACOT13/NUBP2/SULT1E1/CHST1/TXN2/PHGDH/PCCB/CHST11/PARK7/SLC25A19/EXT1/ACACA/AMD1/MGST2/PDHB/GCDH/ADO/LYRM4/OXSM/FXN/G6PD/ELOVL4/DLAT/GDAP1L1/MCCC2/B3GNT3/PAPSS2/GCLC/EXT2/DLD/SLC35D2/PC/ST3GAL4/ELOVL1/OPLAH/MTHFR/CIAO3/CLIC3/PCCA/GSTM4/BAAT/SLC1A1/MCCC1/GSTK1/NDST1/PIPOX/MICAL2/PDHA1/ENOPH1/GALNS/MCEE/ELOVL6/GSS/ACOT8/PDP2/CHST2/CHPF2/B4GALT3/MMACHC/ELOVL2/SUCLA2/SLC19A3/MTHFD2L/LPO/CHST9/TSTD1/HMGCS1 |
| GOMF | ENDONUCLEASE ACTIVITY ACTIVE WITH EITHER RIBO OR DEOXYRIBONUCLEIC ACIDS AND PRODUCING 5 PHOSPHOMONOESTERS | 38 | 0.477159179 | 1.598237266 | 0.009544635 | 0.043442313 | 0.030345158 | 4614 | tags=47%, list=22%, signal=37% | RNASEH2A/FEN1/DNASE1L3/EXO1/ELAC2/DNA2/GEN1/RPP40/POP5/POP4/POP1/MRPL44/DNASE1L1/POP7/RPP30/RPP38/DNASE1L2/ENDOV |
| GOBP | FOREBRAIN CELL MIGRATION | 52 | -0.410268872 | -1.563275983 | 0.009521816 | 0.043367839 | 0.030293136 | 2922 | tags=37%, list=14%, signal=31% | RELN/CXCL12/NRG3/LRRK2/SLIT2/SUN2/FGF13/SYNE2/BMERB1/POU3F3/ZMIZ1/RTN4/PAFAH1B1/DIXDC1/EFHC1/AXL/GLI3/SRGAP2/EMX2 |
| GOBP | POSITIVE REGULATION OF INTERFERON ALPHA PRODUCTION | 22 | 0.557953735 | 1.651665616 | 0.009495715 | 0.043278301 | 0.030230593 | 3140 | tags=41%, list=15%, signal=35% | IRF7/STAT1/IFIH1/MMP12/TBK1/HSPD1/TLR8/DDX58/CHUK |
| GOBP | TETRAPYRROLE METABOLIC PROCESS | 60 | 0.421289557 | 1.541999622 | 0.009476819 | 0.043221502 | 0.030190918 | 3160 | tags=32%, list=15%, signal=27% | TCN1/HMOX2/ABCC1/SRRD/PRSS3/ALAS1/ABCB6/HMBS/HMOX1/MTRR/FECH/MMADHC/COX10/SLC25A39/MMAB/UROS/FXN/MMAA/TSPO |
| GOCC | SPECIFIC GRANULE MEMBRANE | 89 | 0.376971521 | 1.471547983 | 0.009474717 | 0.043221502 | 0.030190918 | 3244 | tags=33%, list=16%, signal=28% | HMOX2/CKAP4/DNAJC5/CD53/RAP2B/LAMTOR2/DGAT1/CD177/TMEM30A/ATP6V1D/PLAU/CD36/ITGB2/SLC44A2/PLAUR/VAMP8/ADAM10/ITGAL/GPR84/ADAM8/TNFRSF1B/SLC2A3/SLC2A5/CYBA/CMTM6/TOM1/KCNAB2/MMP25/LAMTOR3 |
| GOBP | INTRACILIARY TRANSPORT INVOLVED IN CILIUM ASSEMBLY | 40 | -0.455060036 | -1.650807711 | 0.009456827 | 0.043188961 | 0.030168187 | 5820 | tags=68%, list=28%, signal=49% | TNPO1/TTC30B/KIF3C/IFT88/IFT81/TRIP11/IFT140/KIF3A/KIF17/IFT74/DYNC2LI1/IFT27/IFT172/IFT52/TTC21B/DYNC2H1/DYNC2I1/TRAF3IP1/DYNLL2/IFT22/CLUAP1/DYNLRB2/PCM1/IFT122/WDR35/WDR19/DYNLRB1 |
| GOBP | REGULATION OF SYNAPSE ASSEMBLY | 88 | -0.352783607 | -1.504493078 | 0.009418419 | 0.043042816 | 0.030066103 | 5104 | tags=40%, list=24%, signal=30% | AMIGO2/LRRTM2/SLITRK5/EPHB3/LRRTM1/CUX2/NRXN1/FLRT2/AMIGO1/LINGO2/SLITRK2/APP/SRPX2/ROBO2/CBLN1/SETD5/PDLIM5/SLITRK4/CLSTN1/BHLHB9/LRRN1/NLGN1/GPC4/PTPRS/GPC6/NTRK2/MEF2C/SNCA/NTN1/CLSTN2/LRFN5/DLG5/EPHB1/SLITRK6/EEF2K |
| GOMF | I SMAD BINDING | 14 | -0.623129407 | -1.722427208 | 0.00938917 | 0.042938353 | 0.029993134 | 5601 | tags=86%, list=27%, signal=63% | AXIN1/SMAD1/SMAD7/SMAD2/SMAD4/CTNNB1/SMURF1/SMAD9/SMAD5/SMAD3/TGFB1I1/AXIN2 |
| GOBP | NEGATIVE REGULATION OF CELL ADHESION MEDIATED BY INTEGRIN | 12 | -0.649468558 | -1.720826526 | 0.00938917 | 0.042938353 | 0.029993134 | 4239 | tags=67%, list=20%, signal=53% | PTPN11/ACER2/MUC1/CYP1B1/SWAP70/WNK1/NEXMIF/JAM3 |
| GOBP | ESTABLISHMENT OF PIGMENT GRANULE LOCALIZATION | 23 | 0.557459986 | 1.654560076 | 0.009352907 | 0.042830833 | 0.029918029 | 1558 | tags=39%, list=7%, signal=36% | CDH3/MREG/RAB11A/RAB27A/SHROOM2/MKKS/RAB1A/GPR143/MYO5A |
| GOBP | REGULATION OF GLUCOSE TRANSMEMBRANE TRANSPORT | 70 | -0.375893958 | -1.519196982 | 0.009337027 | 0.042787277 | 0.029887604 | 3484 | tags=34%, list=17%, signal=29% | C2CD5/RAP1A/C3/ENPP1/INSR/LEP/EDN1/ADIPOQ/RHOQ/PRKAG2/CLIP3/SLC25A27/GPC3/OSBPL8/PRKCB/BRAF/PID1/KLF15/IRS1/PIK3R1/CREBL2/SORBS1/IRS2/MEF2A |
| GOMF | G PROTEIN COUPLED CHEMOATTRACTANT RECEPTOR ACTIVITY | 26 | 0.545953885 | 1.672801241 | 0.009304528 | 0.042667455 | 0.029803907 | 6134 | tags=58%, list=29%, signal=41% | CXCR2/CXCR4/CCR7/CXCR6/CCR5/ACKR2/CCR1/CCRL2/CCR2/CXCR1/CXCR3/CCR4/CXCR5/CX3CR1/XCR1 |
| GOBP | REGULATION OF DOUBLE STRAND BREAK REPAIR | 83 | 0.393306738 | 1.515411787 | 0.009304514 | 0.042667455 | 0.029803907 | 4190 | tags=40%, list=20%, signal=32% | PARP9/FOXM1/TIMELESS/DTX3L/TRIP12/RAD51/PPP4C/PARPBP/OTUB2/PPP4R2/AUNIP/RAD51AP1/SIRT7/PNKP/FIGNL1/CGAS/NSD2/POLQ/MAD2L2/PRKDC/PML/SIRT6/PARP1/UBQLN4/CYREN/KDM1A/RMI2/WAS/OTUB1/TFIP11/PARP3/SLF1/WRAP53 |
| GOBP | ICOSANOID SECRETION | 41 | 0.47419055 | 1.614119763 | 0.009289911 | 0.042658662 | 0.029797765 | 5702 | tags=54%, list=27%, signal=39% | PLA2G3/PLA2G2F/IL1B/NOS2/SYK/BDKRB2/MAP2K6/PLA2G12A/ANXA1/PTGES/PNPLA8/P2RX4/AVPR1B/PLA2G2A/NTSR1/CYP4F2/PLA2G4F/DRD4/NMB/DRD2/TNFRSF11A/SSTR4 |
| GOBP | MULTIVESICULAR BODY ORGANIZATION | 30 | 0.520139652 | 1.641061718 | 0.009257297 | 0.042537977 | 0.029713465 | 3946 | tags=50%, list=19%, signal=41% | RAB11A/RAB27A/VPS25/STAM/VTA1/VPS37A/SNF8/PDCD6IP/VPS37B/CHMP5/CHMP1B/CHMP6/MVB12A/CHMP1A/CHMP2A |
| GOCC | NUCLEAR EXOSOME RNASE COMPLEX | 16 | 0.625479652 | 1.732014954 | 0.009255987 | 0.042537977 | 0.029713465 | 3564 | tags=56%, list=17%, signal=47% | MPHOSPH6/EXOSC4/EXOSC3/DIS3/EXOSC1/WDR74/EXOSC2/EXOSC5/EXOSC8 |
| GOBP | BASE EXCISION REPAIR | 39 | 0.471699754 | 1.582233437 | 0.009199302 | 0.042329391 | 0.029567764 | 5193 | tags=49%, list=25%, signal=37% | PCNA/POLB/RPA3/FEN1/TDG/APEX2/DNA2/PRMT6/SMUG1/POLQ/NEIL3/MPG/RPA1/POLD1/NTHL1/POLG/LIG1/APEX1/ERCC6 |
| GOBP | POSITIVE REGULATION OF CELLULAR RESPONSE TO INSULIN STIMULUS | 23 | -0.523905555 | -1.653438039 | 0.009195131 | 0.042329391 | 0.029567764 | 5586 | tags=61%, list=27%, signal=45% | PTPN11/PAK1/OPA1/MYO1C/ECHDC3/NUCKS1/SORL1/SIRT1/LEP/OSBPL8/GKAP1/IRS1/SORBS1/SERPINA12 |
| GOBP | REGULATION OF T CELL RECEPTOR SIGNALING PATHWAY | 40 | 0.472990653 | 1.596371939 | 0.009185992 | 0.042326129 | 0.029565485 | 4438 | tags=48%, list=21%, signal=37% | CCR7/GBP1/PTPN2/LCK/PVRIG/PTPN22/SH2D1A/LILRB4/LAPTM5/SLA2/PHPT1/UBASH3A/BCL10/CARD11/NECTIN2/CD300A/THY1/RAB29/MALT1 |
| GOCC | NEUROMUSCULAR JUNCTION | 69 | -0.367400942 | -1.478666065 | 0.009180099 | 0.042326129 | 0.029565485 | 3664 | tags=30%, list=18%, signal=25% | COL4A5/PDZRN3/SPOCK1/LAMA2/DLG1/APP/STXBP5/F2R/SYNJ2BP/ITGB1/SYNC/LAMB2/PTN/TBC1D24/DES/LAMA5/DLG2/LRP4/TUBA1A/POSTN/UTRN |
| GOCC | EXORIBONUCLEASE COMPLEX | 26 | 0.546297292 | 1.673853438 | 0.009177938 | 0.042326129 | 0.029565485 | 2642 | tags=42%, list=13%, signal=37% | CARHSP1/MPHOSPH6/EXOSC4/PNPT1/EXOSC3/DIS3/EXOSC1/WDR74/ZFP36/EXOSC2/EXOSC5 |
| GOMF | ATPASE COUPLED ION TRANSMEMBRANE TRANSPORTER ACTIVITY | 46 | 0.43855214 | 1.524495649 | 0.009173058 | 0.042326129 | 0.029565485 | 2676 | tags=33%, list=13%, signal=28% | ATP12A/ATP6V0D1/ATP6V1B2/ATP5F1B/ATP6V1G1/ATP6V0B/ATP2A2/ATP6V1F/ATP13A1/ATP6V1D/ATP6V1C1/ATP6V1C2/ATP6V1H/ATP6V1A/ATP6V0E1 |
| GOBP | DERMATAN SULFATE PROTEOGLYCAN METABOLIC PROCESS | 16 | -0.613980843 | -1.743389174 | 0.009152063 | 0.042285803 | 0.029537317 | 5647 | tags=62%, list=27%, signal=46% | CSGALNACT2/IDUA/VCAN/BGN/B3GAT3/CSPG4/DSEL/CHST14/DCN/UST |
| GOBP | NEGATIVE REGULATION OF AMYLOID PRECURSOR PROTEIN CATABOLIC PROCESS | 20 | -0.565867157 | -1.717742878 | 0.009149156 | 0.042285803 | 0.029537317 | 4151 | tags=55%, list=20%, signal=44% | RTN2/CLU/SORL1/GGA3/TMED10/RTN1/APOE/BIN1/NTRK2/RTN4/RTN3 |
| GOBP | NEGATIVE REGULATION OF CELLULAR RESPONSE TO GROWTH FACTOR STIMULUS | 93 | -0.355550336 | -1.524625961 | 0.009122231 | 0.042206023 | 0.029481589 | 5594 | tags=46%, list=27%, signal=34% | FGF2/SFRP1/HHEX/SFRP2/FBN1/SULF2/BAMBI/LEMD3/SHISA2/DAB2IP/HTRA1/TOB1/CRIM1/SMURF1/SORL1/CREB3L1/FSTL3/ABL1/AGT/GPC1/SLIT2/NOG/SPART/MMRN2/SEMA6A/TRIM33/PPARG/NBL1/ADGRA2/PIK3CB/GREM1/SMURF2/TNMD/CHRDL1/SKI/RBPMS2/ATP2B4/DCN/SPRY1/SPRY2/GREM2/EPN2/GATA3 |
| GOMF | PROTEIN KINASE A REGULATORY SUBUNIT BINDING | 25 | -0.522296154 | -1.686473888 | 0.009088211 | 0.042077604 | 0.029391886 | 4594 | tags=40%, list=22%, signal=31% | AKAP8/AKAP6/WASF2/PRRC1/C2orf88/WASF3/AKAP1/AKAP11/AKAP9/PJA2 |
| GOBP | NEGATIVE REGULATION OF BMP SIGNALING PATHWAY | 46 | -0.410729412 | -1.533678662 | 0.009081218 | 0.042074223 | 0.029389525 | 5538 | tags=57%, list=27%, signal=42% | CTDSPL2/SMAD7/TMPRSS6/SFRP1/SFRP2/FBN1/BAMBI/LEMD3/HTRA1/TOB1/CRIM1/SMURF1/SORL1/FSTL3/ABL1/NOG/SPART/TRIM33/PPARG/NBL1/GREM1/SMURF2/CHRDL1/SKI/RBPMS2/GREM2 |
| GOBP | ENDOCRINE PROCESS | 75 | -0.363594174 | -1.492619814 | 0.009077241 | 0.042074223 | 0.029389525 | 5339 | tags=44%, list=26%, signal=33% | TACR2/PTPN11/ACE/CPA3/CMA1/SMAD4/GDF9/C1QTNF3/PCSK5/CRY1/AVPR1A/BMP6/TRPV6/EDN3/HSD11B2/TAC1/GAL/GNAS/AGT/CTSG/TBX3/ECE1/LEP/EDN1/F2R/SELENOM/RAB11FIP5/RAB11FIP3/FOXD1/FZD4/AGTR1/INHBB/GATA3 |
| GOBP | NEGATIVE REGULATION OF CARDIOCYTE DIFFERENTIATION | 10 | -0.684925521 | -1.715426523 | 0.009071447 | 0.042074223 | 0.029389525 | 5995 | tags=90%, list=29%, signal=64% | FRS2/DLL1/SMAD4/BMP2/PRICKLE1/DKK1/EGFR/FZD7/SOX6 |
| GOBP | WHITE FAT CELL DIFFERENTIATION | 13 | -0.657402688 | -1.790194966 | 0.008942633 | 0.041518045 | 0.029001026 | 3121 | tags=54%, list=15%, signal=46% | SIRT1/PPARG/AACS/PER2/FABP4/PNPLA3/PDGFRA |
| GOBP | TRANSPORT ALONG MICROTUBULE | 152 | -0.3001989 | -1.369175572 | 0.008920447 | 0.041443682 | 0.028949082 | 5820 | tags=50%, list=28%, signal=36% | TNPO1/BICD1/TTC30B/LCA5L/KIF3C/IFT88/RPGR/LAMP1/IFT81/AP3M1/BICD2/ACTR10/TRIP11/IFT140/KIF3A/SPG11/OPA1/ARHGAP21/MAPK8IP3/BBS12/MAPT/MGARP/DYNC1H1/KIF17/ARMCX3/COPG2/IFT74/DYNC2LI1/IFT27/FEZ1/AP3M2/TTC30A/IFT172/IFT52/KIF5C/KLC3/DYNC1I1/APP/ARL8A/LCA5/TTC21B/TRAK1/DYNC2H1/SFPQ/DYNC2I1/SUN2/FYCO1/TRAF3IP1/PURA/DYNLL2/TERF2/SYNE2/IFT22/RHOT1/TUB/CLUAP1/DST/ARL3/TRAK2/DYNLRB2/MAP2/CAMSAP3/PCM1/AP3S1/IFT122/WDR35/MAP1A/PAFAH1B1/WDR19/TMEM108/KIF13A/DLG2/SYBU/SOD1/DYNLRB1/MAP1B |
| GOBP | NEURAL CREST CELL MIGRATION | 55 | -0.40143456 | -1.550229855 | 0.008919734 | 0.041443682 | 0.028949082 | 5087 | tags=47%, list=24%, signal=36% | EDNRB/TBX1/ALX1/SEMA4C/SEMA3C/SEMA3A/RADIL/PITX2/EDN3/SEMA6D/NRP1/ERBB4/BMP4/KITLG/NRTN/SOX10/SEMA3E/SOX8/SEMA4G/SEMA6A/TWIST1/SEMA3B/FN1/BMP7/SEMA3G/LAMA5 |
| GOMF | COLLAGEN BINDING | 68 | -0.378537886 | -1.518196014 | 0.008915872 | 0.041443682 | 0.028949082 | 5498 | tags=50%, list=26%, signal=37% | PCOLCE/LACRT/PAK1/VWF/COL5A3/SMAD4/DDR1/PDGFB/COL6A1/COL6A2/GP6/ITGA1/CTSK/LOX/AEBP1/DDR2/ITGA11/CHADL/ANTXR1/PDGFA/PCOLCE2/PODN/MRC2/ITGB1/FN1/ECM2/SMAD3/CCBE1/DCN/ASPN/SPARCL1/ITGA9/CRTAP/TGFBI |
| GOBP | HOMOLOGOUS RECOMBINATION | 57 | 0.43682207 | 1.585598749 | 0.00889745 | 0.041422778 | 0.02893448 | 3702 | tags=30%, list=18%, signal=25% | RAD51/TRIP13/CENPX/TOP2A/RAD51AP1/SHOC1/BRIP1/MND1/FANCD2/CENPS/RAD54L/RAD54B/MSH3/EME1/SYCE3/RAD50/REC8 |
| GOBP | REGULATION OF SIGNAL TRANSDUCTION BY P53 CLASS MEDIATOR | 173 | 0.328284379 | 1.405790137 | 0.008821668 | 0.041098451 | 0.028707932 | 4898 | tags=40%, list=23%, signal=31% | AURKB/HDAC1/AURKA/TPX2/SMYD2/HUS1/RPA3/PRMT5/ZMPSTE24/NOP2/EXO1/EEF1E1/MIF/BRIP1/CDK5/TAF10/BCL2L12/RFC2/CSNK2A1/DNA2/PLK3/BLM/RFC3/PRMT6/BOP1/ARMC10/NOC2L/TAF13/RPF2/CHEK2/BDKRB2/TP63/TRIAP1/HIPK2/BRCA1/HDAC2/CDK2/MEAF6/PML/ING2/TAF11/TOP3A/MDM2/PSMD10/RFC4/RRS1/PRKAG1/PAK1IP1/KDM1A/RMI2/SSRP1/RAD50/MAPK14/ATR/MAPKAPK5/RPA1/TAF5/HNRNPK/CSNK2B/MBD3/SETD9/PMAIP1/RFC5/TAF2/YJU2/POU4F2/RHNO1/RBBP4/PYHIN1 |
| GOBP | NLRP3 INFLAMMASOME COMPLEX ASSEMBLY | 14 | 0.645129355 | 1.704483872 | 0.00880786 | 0.0410626 | 0.02868289 | 2991 | tags=50%, list=14%, signal=43% | GBP5/STMP1/AIM2/CD36/DHX33/NLRC3/CPTP |
| GOBP | NEGATIVE REGULATION OF CELL MATRIX ADHESION | 36 | -0.458404605 | -1.614882846 | 0.008797059 | 0.041040722 | 0.028667608 | 4530 | tags=50%, list=22%, signal=39% | LRP1/PHLDB2/ACER2/DMTN/PTEN/BCAS3/JAG1/FAM107A/SEMA3E/APOD/ACVRL1/ARHGAP6/NEXMIF/MYOC/BCL6/PIK3R1/DLC1/POSTN |
| GOBP | PH REDUCTION | 57 | 0.437145755 | 1.586773675 | 0.008781145 | 0.04099495 | 0.028635635 | 3309 | tags=33%, list=16%, signal=28% | RAB38/ATP6V0D1/ATP6V1B2/ATP6V1G1/ATP6V0B/ATP6V1F/CLCN3/SLAMF8/ATP6V1D/ATP6V1C1/ATP6V1C2/GRN/ATP6V1H/ATP6V1A/SNAPIN/ATP6V0E1/FASLG/CLN6/TMEM199 |
| GOCC | PROTEIN KINASE COMPLEX | 100 | 0.380169892 | 1.513235306 | 0.008760477 | 0.040926902 | 0.028588103 | 4080 | tags=38%, list=20%, signal=31% | CCNB1/CDK1/CCNB2/CKS2/PYCARD/PCNA/CCNE1/CCNA2/CCNE2/CCNF/ATG13/CDK5/PHKA1/CDKN1A/IFIT5/BCCIP/CKS1B/SESN2/CCND2/IKBKG/PYDC1/PRKAR2A/CAB39/CCNO/CDK7/CDK2/MAP3K5/RB1/CCNH/CHUK/CDK9/PRKAG1/CCND3/CDKN2D/CDK4/PHKG2/WDR41/CCNK |
| GOMF | ANTIOXIDANT ACTIVITY | 81 | 0.393174886 | 1.512305041 | 0.008740611 | 0.040862507 | 0.028543122 | 5405 | tags=44%, list=26%, signal=33% | S100A9/TXNDC17/GPX2/SRXN1/GSTP1/GSTO1/SELENOT/TXNRD1/PRDX1/DUOX1/GPX1/SOD2/SESN2/GSTZ1/TXN/PRDX5/TXNRD3/PARK7/IPCEF1/MGST2/PRDX6/PTGES/PXDN/PRDX4/HP/ALOX5AP/PRXL2B/GSTK1/PXDNL/LPO/SELENOS/DUOX2/PTGS2/LOXHD1/NQO1/APOA4 |
| GOBP | DEOXYRIBONUCLEOSIDE MONOPHOSPHATE METABOLIC PROCESS | 16 | 0.627407851 | 1.737354326 | 0.008738178 | 0.040862507 | 0.028543122 | 1371 | tags=38%, list=7%, signal=35% | TYMP/UPP1/TK1/TYMS/XDH/DNPH1 |
| GOBP | ADHERENS JUNCTION ASSEMBLY | 12 | -0.651541414 | -1.72631875 | 0.008712492 | 0.040787818 | 0.02849095 | 5248 | tags=83%, list=25%, signal=62% | SMAD7/HIPK1/PIP5K1C/PAK2/CTNNB1/RAMP2/VCL/DLG5/JAM3/ZNF703 |
| GOBP | POSITIVE REGULATION OF PROTEIN KINASE B SIGNALING | 160 | -0.308578463 | -1.415920846 | 0.008710388 | 0.040787818 | 0.02849095 | 3370 | tags=31%, list=16%, signal=26% | FGFR3/ERBB3/KLB/FGF1/IL18/ERBB4/PDGFRB/KITLG/NRG2/INSR/LEP/STK3/IGF1R/TNFAIP8L3/MET/ESR1/PDGFA/FGF9/EGF/EGFR/HIP1/AKR1C3/PTK2/PIK3CB/FGFR2/ITGB1/GAS6/FGFR1/RICTOR/OSBPL8/IGFBP5/F10/PINK1/FERMT2/RTN4/MYOC/TSPYL5/FYN/IRS1/PIK3R1/AXL/PDGFRA/TCF7L2/SPRY2/KIT/IRS2/GATA3/F3/BTC |
| GOBP | REGULATION OF CYSTEINE TYPE ENDOPEPTIDASE ACTIVITY INVOLVED IN APOPTOTIC SIGNALING PATHWAY | 17 | 0.60461726 | 1.687042316 | 0.008692824 | 0.040752543 | 0.02846631 | 3304 | tags=59%, list=16%, signal=50% | MMP9/PIH1D1/LGALS9/CAAP1/PLAUR/CASP8/PARK7/BAX/HTRA2/NLE1 |
| GOCC | ENDOPLASMIC RETICULUM GOLGI INTERMEDIATE COMPARTMENT MEMBRANE | 72 | 0.410714214 | 1.543063657 | 0.00868614 | 0.040749645 | 0.028464286 | 3968 | tags=31%, list=19%, signal=25% | CTSC/PRRG4/SERPINA1/TGFA/AREG/TAP1/TAP2/LMAN2/BCAP31/YIF1A/YIF1B/CNIH1/YKT6/LAMP5/TMED5/SURF4/TAPBP/LMAN1/TMEM199/TMED7/TMED9/ROBO1 |
| GOCC | CYTOPLASMIC REGION | 251 | -0.270486332 | -1.310065888 | 0.008672857 | 0.040715761 | 0.028440617 | 4701 | tags=29%, list=23%, signal=23% | INPP5E/MAPT/ERC1/MGARP/DYNC1H1/KIF17/ARMCX3/PHLDB2/DNAH9/PCLO/CFAP36/CEP162/BBS7/AK8/ARL6/DYNC2LI1/EFHC2/SPATA7/DNAL1/AP3M2/TTC30A/IFT172/ZC3H14/SEPTIN2/KIF5C/DRC3/KCNAB1/ARL8A/LCA5/CFAP91/DZIP1L/LRRK2/TRAK1/DYNC2H1/SFPQ/CLASP1/DNAI7/DYNC2I1/TRAF3IP1/PURA/TERF2/TULP3/EPB41L2/DST/NUMA1/TRAK2/SEPTIN7/PKD2/EPB41/CCDC113/PHLDB1/MAP2/CAMSAP3/BBS1/GABARAPL1/AP3S1/MAPK1/WDR35/WDPCP/MAP1A/PAFAH1B1/ATG14/TMEM108/SPAG16/DLG2/GNAI1/EFHC1/GLI2/GLI3/SYBU/SOD1/CCSAP/MAP4/WLS |
| GOBP | REGULATION OF STRIATED MUSCLE CELL DIFFERENTIATION | 76 | -0.378482753 | -1.55733887 | 0.008662286 | 0.040694573 | 0.028425817 | 5433 | tags=49%, list=26%, signal=36% | MYLK3/PAK1/DLL1/IGF1/RBM24/SMAD4/TRIM32/TBX1/MAMSTR/AKAP6/HDAC4/PI16/MSX1/TMEM119/KAT2A/HDAC9/HDAC5/PLPP7/BMP2/PARP2/BMP4/RGS2/SHOX2/EDN1/CCN3/DKK1/FZD7/GREM1/PPARA/MEF2C/MYOCD/NLN/CYP26B1/BHLHE41/BCL2/SOX6/EFNB2 |
| GOCC | INTRINSIC COMPONENT OF ENDOPLASMIC RETICULUM MEMBRANE | 158 | 0.341438939 | 1.452176772 | 0.008661465 | 0.040694573 | 0.028425817 | 5241 | tags=45%, list=25%, signal=34% | SLC35B1/DHRS9/DPAGT1/HSPA5/HM13/TAP1/SPPL2A/ZMPSTE24/TAP2/ELOVL7/BCAP31/TMCO1/EMC6/UBXN8/EDEM1/EMC3/CYP2E1/EMC8/PREB/DERL2/EMC9/HACD1/AUP1/SEC61A1/SLC37A4/HLA-E/RCE1/DOLK/SLC37A2/TAPBP/SAMD8/EXT1/DPM2/CANX/SREBF2/EMC1/CPT1C/DOLPP1/ELOVL4/SLC35B4/HLA-DRA/ESYT3/EMC7/ANKLE2/B2M/HLA-F/ELOVL1/HLA-B/G6PC1/PIGU/HLA-G/ACER3/GRAMD2A/TMEM33/DNAJB12/BNIP1/ELOVL6/GPAA1/ELOVL2/EMC4/PDIA3/PORCN/HLA-DQB1/SELENOS/CLN3/HLA-A/EMC10/TM7SF2/DERL1/HLA-DPB1/HLA-C |
| GOBP | REGULATION OF INTRACELLULAR TRANSPORT | 340 | 0.292076381 | 1.337568135 | 0.008618403 | 0.04054512 | 0.028321422 | 4119 | tags=32%, list=20%, signal=26% | ZC3H12A/RAN/IFI27/TFDP1/GZMB/FZD5/PLA2G3/SPAG5/NMT1/UBE2L3/YWHAQ/FERMT1/ZPR1/PCSK9/UBE2D3/SFN/ECT2/BORCS5/NDRG4/TMEM30B/CPSF6/IFNG/BCAP31/IL1B/MX2/UBL5/ATG13/SIRT7/BAG3/PDCD10/BID/CDK5/EIPR1/HAX1/MTCL1/PDCD5/TCAF2/TMEM30A/EDEM1/NOLC1/RAC2/KIF20B/PLK3/CD36/ITGB2/CIB1/PREB/SAE1/NSUN2/MTMR2/DERL2/HRAS/SNX12/RANGAP1/CASP8/ARV1/TP63/ANTKMT/TXN/TARDBP/YWHAZ/ARIH2/NDEL1/PARK7/PIK3R2/EDEM2/IWS1/UBE2J2/NRDE2/HCLS1/SREBF2/OAZ1/MFF/SNX3/HTRA2/MIEF1/DYNLT2B/SP100/LDLRAP1/LCP1/UBAC2/PKIA/LMAN1/PRKCD/RIOK2/UHMK1/NUTF2/MAPK8/CEMIP/SCFD1/SREBF1/HDAC3/GSK3A/YOD1/CACNB3/NUP58/EMD/MAP2K1/CDH1/SLC1A1/MSN/JUP/MAPK14/NEFH/DNAJC30/ATP5IF1/UBE2J1/ARHGAP1 |
| GOBP | ORGANOPHOSPHATE ESTER TRANSPORT | 127 | 0.354312516 | 1.464699424 | 0.00861379 | 0.04054512 | 0.028321422 | 4395 | tags=33%, list=21%, signal=26% | SLC25A5/PRELID1/ABCC1/LDLR/ABCA12/PLSCR1/PRELID3B/ATP10B/DBI/TMEM30B/ABCG1/EPG5/GLTP/OSBPL2/PITPNC1/SLC25A25/SLC25A33/SLC25A51/TMEM30A/ATP10D/SLC37A4/PITPNB/SLC37A2/TRIAP1/SLC25A19/PITPNM1/SLC19A1/FASLG/CPTP/PITPNA/PRKCD/C2CD2L/G6PC1/SLC66A2/SLC25A32/PLSCR2/GJA1/ATP8B1/ABCA7/CR1/ATP11B/ABCC5 |
| GOBP | DEVELOPMENTAL CELL GROWTH | 207 | -0.290515948 | -1.369904068 | 0.008607832 | 0.04054512 | 0.028321422 | 4901 | tags=37%, list=23%, signal=28% | GDF9/GDI1/SEMA4C/SEMA3C/MAPT/DDR1/AKAP6/SHTN1/LRP1/HDAC6/USP9X/PI16/SEMA3A/DVL1/RND2/CTNNB1/RUFY3/SMURF1/PARP2/PRKN/SEMA6D/NEDD4L/NRP1/TNN/VCL/SYT1/APP/ABL1/SIN3A/KMT2D/CXCL12/RTN4R/RGS2/PDLIM5/SEMA3E/GOLGA4/EDN1/MACF1/L1CAM/SLIT2/SPART/SEMA4G/AUTS2/SEMA6A/FGF13/SEMA3B/PPP3CB/NDN/RYK/SLIT3/APOE/PTPRS/DCLK1/ULK2/LAMB2/FN1/SORBS2/GSK3B/PPARA/MAP2/SYT17/NTN1/AKAP13/IQGAP1/ADRA1A/RTN4/TGFBR2/DPYSL2/NIN/SEMA3G/TMEM108/DBN1/POSTN/ALCAM/MAP1B/COBL |
| GOBP | CELL SURFACE RECEPTOR SIGNALING PATHWAY INVOLVED IN HEART DEVELOPMENT | 27 | -0.522705826 | -1.712486987 | 0.008605482 | 0.04054512 | 0.028321422 | 4675 | tags=48%, list=22%, signal=37% | WNT11/HEY1/MSX1/CTNNB1/BMP2/BMP4/JAG1/NOG/DKK1/BMPR1A/GALNT11/RBPJ/NOTCH2 |
| GOBP | RESPONSE TO TEMPERATURE STIMULUS | 217 | 0.317504896 | 1.393501195 | 0.008603904 | 0.04054512 | 0.028321422 | 4826 | tags=37%, list=23%, signal=29% | CXCR4/EIF2B2/DNAJB6/VCP/RPA3/EIF2S1/DNAJA2/ERO1A/CXCL10/ATP2A2/NUP37/IRAK1/NUP210/PLAC8/HSBP1/NUP88/CLPB/IER5/BAG3/LYN/HMOX1/HSPA8/CDKN1A/HSPH1/SOD2/PDCD6/NUP50/GLRX2/HIKESHI/ACOT11/NR2F6/DNAJB1/CASP8/POLR2D/EIF2B1/RAE1/SEC13/DNAJC7/TFEC/HSPD1/EIF2B4/CHORDC1/NUP155/HDAC2/DNAJA3/TRPV3/MICB/NDC1/MLST8/HTRA2/DNAJC2/NUP188/NUP85/GCLC/PTGES3/FKBP4/EIF2B5/DAXX/MAPKAPK2/NUP205/EIF2B3/UCP2/NUP58/NTSR1/MTOR/NUP107/NUP93/VGF/ATR/POM121/RPA1/NUP160/AKT1S1/DNAJA1/ACADVL/NUP35/UCP3/MAPK3/SST/NUP42/ARRB2 |
| GOBP | EMBRYONIC BODY MORPHOGENESIS | 11 | -0.675456419 | -1.728554381 | 0.008530075 | 0.040270585 | 0.028129654 | 5323 | tags=82%, list=26%, signal=61% | MAB21L2/ZNF281/PHLDB2/CLASP1/PHLDB1/DAG1/IFT122/CDON/GREM2 |
| GOCC | ENDOSOME MEMBRANE | 489 | 0.279383968 | 1.312222503 | 0.008524275 | 0.040270585 | 0.028129654 | 4951 | tags=34%, list=24%, signal=27% | TMEM165/CD274/MICALL1/IRF7/FZD5/AP2S1/MREG/ABHD17C/MYD88/LAMP3/LDLR/ATP6V0D1/IRAK2/DTX3L/RAB11A/RAB27A/YIPF1/ATP6V0B/PCSK9/VPS25/IRAK1/SPPL2A/SCYL2/UBE2D3/ABCB6/SNX20/FCGR1B/RAP2B/PSENEN/AP3D1/SNX1/LAMTOR5/LAMTOR2/CLCN3/RAB31/VPS29/RAB5A/IKBKE/STOML1/GNPNAT1/SNX6/STAM/VTA1/TBK1/SPNS2/NSG1/WDR91/PLEKHB2/IFITM3/SNX10/RAB10/STEAP4/MYO1B/SLC39A4/LAMP5/EHD4/COMMD1/RCC2/AP5S1/MTMR2/CTSD/TMEM163/PMEL/ITCH/SH3GL3/NPC1/TOM1L1/ZNRF2/SNX12/TFRC/VAMP8/HLA-DMB/VPS37A/HLA-DOB/HLA-E/VAMP7/VPS33B/BOK/SLA2/SNF8/CLCN5/FURIN/ATP6V0E1/LY96/CMTM6/SORCS2/TLR8/VPS37B/CLTA/PLIN3/PML/AP1G2/CHMP5/CHMP1B/CPTP/STEAP3/SLC38A9/FGD2/CD1B/WIPI1/SNX3/UBAP1/AP5M1/ANXA1/TYRP1/SLC15A3/HLA-DRA/LAMTOR3/CHMP6/MCOLN2/RMC1/RAB8A/RHOV/B2M/CD68/ARC/VPS16/HLA-F/BACE1/BECN1/HLA-B/MVB12A/ATP6V0A2/MARCHF1/CHMP1A/SCAMP3/SNX5/SPHK1/HLA-G/SLC1A1/PLEKHM2/IRAK4/CHMP2A/ABHD17B/HPS6/ARL8B/ARHGAP1/PLPP2/ACAP1/ABCA7/CD1D/ATP11B/AP2M1/STEAP1/ABCC5/VAC14/NCDN/AMN/CLCN4/HLA-DOA/OR51E2/UBA1/PSD/CORO1C/ARHGAP32/VPS53/PDIA3/HLA-DMA/TICAM1/WDR83/SLC15A4/HLA-DQB1/STARD3NL/HGS/GGA1/SNX17/CLN3 |
| GOBP | TOLERANCE INDUCTION | 28 | 0.524572264 | 1.645818509 | 0.008487251 | 0.040124807 | 0.028027826 | 6065 | tags=68%, list=29%, signal=48% | CD274/IDO1/ICOS/LYN/LGALS9/IL2RA/LILRB4/HLA-E/CD3E/IRAK3/LILRB2/HLA-B/HLA-G/FOXP3/AIRE/CCR4/CLC/XKR8/FOXJ1 |
| GOCC | CYTOSOLIC SMALL RIBOSOMAL SUBUNIT | 43 | -0.443517903 | -1.632113213 | 0.008480951 | 0.04012326 | 0.028026745 | 5734 | tags=60%, list=27%, signal=44% | RPS3/RPS21/RPS10/RPS13/RPS12/RPS7/RPS18/EIF2D/RPS11/RPS5/RPS9/RPS25/RPS15A/RPS15/RPS27/EIF2A/RPS8/RPS6/RPS4X/RPS14/RPS17/RPS24/RPS23/RPS27L/RPS20/RPS3A |
| GOBP | NEGATIVE REGULATION OF MUSCLE CELL DIFFERENTIATION | 52 | -0.413331086 | -1.574944149 | 0.008469693 | 0.040098238 | 0.028009267 | 5351 | tags=52%, list=26%, signal=39% | PAK1/DLL1/SMAD4/PDGFB/HDAC4/PI16/MSX1/TMEM119/HDAC5/PLPP7/ANKRD17/BMP2/RGS2/CCN3/DKK1/FGF9/FZD7/NFATC2/MECP2/PPARA/MYOCD/RBPMS2/PDCD4/NFATC1/BHLHE41/PRDM6/SOX6 |
| GOBP | ESTABLISHMENT OF PROTEIN LOCALIZATION TO TELOMERE | 18 | 0.607582478 | 1.723007027 | 0.008418397 | 0.039883492 | 0.027859264 | 4190 | tags=67%, list=20%, signal=53% | CCT5/CCT2/CCT3/CCT7/BRCA2/TCP1/DKC1/CCT6A/CCT8/NABP2/ATR/WRAP53 |
| GOBP | AMINE TRANSPORT | 90 | -0.345335767 | -1.470684453 | 0.008412278 | 0.039882628 | 0.027858661 | 4134 | tags=28%, list=20%, signal=22% | AVPR1A/VIP/PRKN/SLC43A1/SLC12A2/SYT1/CLTRN/CXCL12/RGS2/LEP/SNCG/PER2/NPY5R/ITGB1/SYT17/SNCA/RAB3B/PINK1/RAB3GAP1/ARL6IP5/ATP1A2/ADRA2A/SLC18A2/SYT8/AQP9 |
| GOMF | TRANSLATION REGULATOR ACTIVITY NUCLEIC ACID BINDING | 97 | 0.375959699 | 1.488653011 | 0.008402269 | 0.039863309 | 0.027845166 | 4350 | tags=42%, list=21%, signal=34% | GSPT1/C1QBP/EIF2B2/TSFM/EIF4E2/MCTS1/EIF2S2/EIF4E/TYMS/EIF6/EIF3J/EIF2S1/SHMT2/EIF3I/MRPL58/COPS5/DENR/EIF3K/GTPBP2/TUFM/MTIF2/DHFR/EIF3B/EIF2B1/EIF4H/EIF4G2/EIF2B4/LARP1/ABCF1/EIF4G1/EIF5/EIF2B5/DAZL/EIF5A/EIF2B3/DHX29/MTRF1L/EIF3A/CPEB4/EFL1/EIF3M |
| GOBP | CARDIAC NEURAL CREST CELL DIFFERENTIATION INVOLVED IN HEART DEVELOPMENT | 11 | -0.677272217 | -1.733201174 | 0.008358617 | 0.039684235 | 0.027720079 | 4733 | tags=73%, list=23%, signal=56% | SEMA3C/PITX2/BMP4/HES1/JAG1/TWIST1/MAPK1/BMP7 |
| GOBP | TRNA METHYLATION | 39 | 0.474595195 | 1.591945682 | 0.008357548 | 0.039684235 | 0.027720079 | 3573 | tags=38%, list=17%, signal=32% | WDR4/MTO1/FTSJ1/METTL6/METTL1/HSD17B10/TRMT10C/NSUN2/TRMT6/TRMT112/METTL8/TRMT1/LCMT2/THUMPD3/TYW3 |
| GOCC | MIDBODY | 190 | 0.331031788 | 1.430175409 | 0.008321734 | 0.039565043 | 0.027636822 | 3712 | tags=29%, list=18%, signal=24% | LAP3/RAN/CDK1/KIF20A/BIRC5/CEP55/AURKB/NEK2/PRC1/KATNB1/KIF4A/AURKA/SPAG5/SHCBP1/HSPA5/KIF14/TXNDC9/CENPF/CDCA8/GNL3/BCL3/CENPE/RALB/ECT2/KIF23/RACGAP1/SLC2A1/EXOC3/ANLN/MTCL1/PSRC1/PLK1/CIAO2B/KIF20B/TTLL12/GNAI3/RCC2/USP3/CCDC124/TRAPPC14/PDCD6IP/PITPNM1/KEAP1/NAT10/VPS37B/EXOC2/CHMP1B/RNF8/KATNA1/RAB8A/UHMK1/ENTR1/MPLKIP/CAPG/AGAP2/CCDC69 |
| GOCC | LUMENAL SIDE OF MEMBRANE | 31 | 0.52287795 | 1.665964192 | 0.008320107 | 0.039565043 | 0.027636822 | 5595 | tags=61%, list=27%, signal=45% | BDH1/HM13/SPPL2A/BCAP31/HSPA8/CTSA/HLA-E/TAPBP/CANX/HLA-DRA/HLA-F/HLA-B/HLA-G/DNAJC19/HLA-DQB1/HLA-A/HLA-DPB1/HLA-C/CALR |
| GOBP | REGULATION OF SPINDLE CHECKPOINT | 15 | 0.630631366 | 1.705756052 | 0.008263371 | 0.039343251 | 0.027481897 | 2381 | tags=47%, list=11%, signal=41% | CCNB1/NDC80/MAD2L1/CDT1/GEN1/MAD2L1BP/LCMT1 |
| GOBP | DERMATAN SULFATE METABOLIC PROCESS | 13 | -0.661163173 | -1.800435266 | 0.008262981 | 0.039343251 | 0.027481897 | 5647 | tags=62%, list=27%, signal=45% | IDUA/VCAN/BGN/CSPG4/DSEL/CHST14/DCN/UST |
| GOBP | PURINE NUCLEOSIDE METABOLIC PROCESS | 59 | 0.42732034 | 1.560871443 | 0.008225954 | 0.039220697 | 0.027396291 | 2926 | tags=32%, list=14%, signal=28% | RAN/PNP/NME1/AHCY/MFN1/ENTPD7/ACP3/TJP2/APRT/IMPDH1/NT5C2/HPRT1/XDH/ADK/GNAI3/GMPS/ADA2/AMD1/CASK |
| GOBP | NUCLEOLAR LARGE RRNA TRANSCRIPTION BY RNA POLYMERASE I | 17 | 0.606874372 | 1.693340257 | 0.008177704 | 0.039018334 | 0.027254937 | 4956 | tags=76%, list=24%, signal=58% | MACROH2A1/IPPK/MARS1/SIRT7/PIH1D1/MACROH2A2/SMARCA4/NOL11/MTOR/PWP1/TAF1B/NCL/TCOF1 |
| GOBP | REGULATION OF LIPID METABOLIC PROCESS | 378 | -0.244555561 | -1.246027956 | 0.008175748 | 0.039018334 | 0.027254937 | 4981 | tags=36%, list=24%, signal=28% | STUB1/LGALS12/ABCD2/FABP3/MBTPS2/NPC2/HTR2A/CPT1A/NCOA6/FASN/H6PD/ABCA2/PDGFB/CYP1A1/DAB2IP/ELOVL5/PSAP/NCOA1/TEK/MED1/NR1D2/RGN/PLIN2/ACSL3/INSIG1/ERLIN2/AVPR1A/AADAC/RGL1/LSS/RARRES2/SORL1/BMP2/BMP6/PIP4K2A/PCK1/GPER1/NCOA2/SCARB1/PDK2/SLC27A1/PRKCE/ACSL1/FGFR3/TIAM2/PEX11A/C3/FGF1/NCOR1/FADS1/MFSD2A/WASHC1/SIRT1/SIN3A/APOC1/GPAM/ADGRF5/NPAS2/GAL/PDGFRB/AGT/ABCB4/PRKD1/GGPS1/G0S2/RORC/BCL11B/NR1H3/LEP/CHD9/ADIPOQ/FITM2/MTMR3/MBTPS1/APOD/FMO5/TNFAIP8L3/EPHX2/PRKAG2/TWIST1/PDGFA/PLPP1/PPARG/ENHO/CREBBP/AKR1C3/DKK3/APOE/PTK2/DGAT2/CD81/ORMDL1/NFYB/PIBF1/PIK3IP1/PRKAA2/PPARA/HMGCS2/PDK3/PPARGC1A/THRSP/THRA/ORMDL3/PDK4/PSAPL1/VAV3/C1QTNF2/FBXW7/SP1/PIP4K2B/STAR/CRTC3/ASAH1/ABHD6/PRKAB2/CCDC3/ABCA3/STAT5B/ATG14/ADRA2A/IRS1/NCOR2/AGTR1/PDGFRA/RXRA/CREBL2/TBL1XR1/KIT/ZBTB20/CAV1/SORBS1/SOD1/IRS2/SERPINA12/ACADL/RORA |
| GOBP | NUCLEAR ENVELOPE REASSEMBLY | 18 | 0.60903523 | 1.727126799 | 0.008165217 | 0.039014174 | 0.027252031 | 3946 | tags=50%, list=19%, signal=41% | PPP2CA/PPP2R2A/REEP4/VRK1/NSFL1C/ANKLE2/PPP2R1A/EMD/CHMP2A |
| GOBP | B CELL ACTIVATION | 261 | 0.30724782 | 1.372400746 | 0.008135359 | 0.038899175 | 0.027171702 | 4929 | tags=35%, list=24%, signal=27% | NOD2/BLNK/TNFRSF21/CTPS1/HMGB3/BAK1/EZH2/PHB/PTPN2/BCL3/ADAM17/SAMSN1/PLCG2/TNIP2/SASH3/JAK3/EXOSC3/EXO1/MIF/LYN/SLAMF8/CDKN1A/RASGRP1/SYK/IKZF3/IL7R/IL6/ITGA4/CD27/BATF/NSD2/MAD2L2/GPR183/RNF168/PRKDC/TFRC/HMCES/CASP8/LAPTM5/PIK3CD/CD86/LIG4/HSPD1/BST2/DOCK10/LAT2/LEF1/BAX/ERCC1/RNF8/PELI1/CASP3/TYROBP/PHB2/PRKCD/TCF3/CD38/TNFRSF13B/TNFRSF4/CARD11/TBC1D10C/ZAP70/MNDA/TNFSF13B/IGHV7-81/MZB1/CD300A/IL10/FOXP3/DCAF1/XBP1/PARP3/TRDC/VCAM1/CR1/TNFSF13/PTPRC/CD28/MALT1/DNAJB9/ZBTB7A/INHA/ADGRG3/PPP2R3C/CD40/TICAM1/TBX21/SLC15A4/IFNE/IFNA2/MIR17HG |
| GOBP | POSITIVE REGULATION OF SMOOTH MUSCLE CELL MIGRATION | 36 | -0.460324998 | -1.621648069 | 0.00811663 | 0.038837267 | 0.027128459 | 5576 | tags=58%, list=27%, signal=43% | NOX4/PAK1/IGF1/ATP7A/PDGFB/LRP1/HDAC4/CCN4/SEMA6D/NRP1/PDGFRB/DOCK5/DOCK4/FGF9/PDGFD/DOCK7/IGFBP5/IQGAP1/LPAR1/BCL2/POSTN |
| GOBP | NEGATIVE REGULATION OF CARTILAGE DEVELOPMENT | 25 | -0.525111697 | -1.695565165 | 0.008052985 | 0.038560196 | 0.02693492 | 5946 | tags=60%, list=28%, signal=43% | RARB/NKX3-2/WNT9A/PTPN11/WNT11/CCN4/RFLNB/BMP4/LEP/CHADL/NOG/GREM1/FRZB/EFEMP1/GLI2 |
| GOBP | CARDIAC VENTRICLE MORPHOGENESIS | 69 | -0.37248508 | -1.499128023 | 0.008026043 | 0.038458601 | 0.026863954 | 3538 | tags=33%, list=17%, signal=28% | SEMA3C/HEY1/MED1/TGFB2/FOXC2/PPP1R13L/JAG1/SOX4/NOG/CPE/NPY5R/DSP/FOXC1/FGFR2/BMPR1A/MEF2C/RBPJ/TGFBR2/ZFPM2/TNNC1/TPM1/TGFBR3/GATA3 |
| GOBP | MEMBRANE DEPOLARIZATION DURING ACTION POTENTIAL | 35 | -0.476955145 | -1.665638764 | 0.008019426 | 0.038454323 | 0.026860966 | 3906 | tags=37%, list=19%, signal=30% | SCN1B/SCN3A/SCN9A/PTPN3/CACNA1H/KCNH2/CACNB2/ANK2/SLC8A1/SCN7A/SCN3B/ATP1A2/CACNA2D1 |
| GOBP | CENTRAL NERVOUS SYSTEM NEURON AXONOGENESIS | 35 | -0.476929593 | -1.665549529 | 0.008019426 | 0.038454323 | 0.026860966 | 3554 | tags=49%, list=17%, signal=40% | EPHB3/SZT2/SCN1B/PTEN/TCTN1/SLIT2/EPHB6/TSKU/ARHGAP35/DCLK1/ADARB1/NFIB/PAFAH1B1/EPHB1/NIN/GLI2/C12orf57 |
| GOBP | REGULATION OF NEUROTRANSMITTER TRANSPORT | 94 | -0.350967098 | -1.500849042 | 0.008015088 | 0.038454323 | 0.026860966 | 3887 | tags=32%, list=19%, signal=26% | RAP1B/GPER1/PRKN/GRIN3A/NOS1/RAP1A/SYT1/PREPL/STXBP5/LRRK2/SNCG/NLGN1/SNCAIP/CACNB2/PNKD/PER2/RHOT1/GPM6B/ITGB1/GSK3B/MEF2C/P2RX1/SNCA/RAB3B/PRKCB/ADRA1A/RAB3GAP1/ATP1A2/ADRA2A/KCNMB4 |
| GOBP | REGULATION OF AXONOGENESIS | 149 | -0.307367092 | -1.398139781 | 0.007999126 | 0.038439293 | 0.026850467 | 4765 | tags=34%, list=23%, signal=27% | SEMA4C/SEMA3C/MAPT/PAK2/SHTN1/LRP1/SEMA3A/RND2/RUFY3/AMIGO1/LRRC4C/SEMA6D/PTEN/NRP1/PLXND1/EFNB3/TIAM2/ROBO2/CXCL12/RTN4R/SHOX2/SEMA3E/GOLGA4/MACF1/L1CAM/SLIT2/SPART/SEMA4G/MAP6/SEMA6A/EFNA1/FGF13/SEMA3B/RYK/PTPRS/ARHGAP35/NTRK2/FN1/TRAK2/GSK3B/MAP2/NTN1/RTN4/PAK3/NIN/PLXNA2/SEMA3G/DBN1/UST/LRP4/MAP1B |
| GOCC | T TUBULE | 51 | -0.423175171 | -1.608357132 | 0.007966264 | 0.038308776 | 0.026759299 | 2151 | tags=37%, list=10%, signal=33% | CASQ1/SCN1B/AKAP6/RTN2/RDX/CAPN3/CACNB2/FXYD1/PPP3CB/ANK2/BIN1/SLC8A1/ADRA1A/ATP2B4/STAC/ATP1A2/CACNA2D1/AHNAK/AHNAK2 |
| GOBP | MICROTUBULE BASED TRANSPORT | 182 | -0.296093086 | -1.38933779 | 0.007955253 | 0.038283229 | 0.026741454 | 5492 | tags=44%, list=26%, signal=33% | IFT88/RPGR/LAMP1/IFT81/AP3M1/BICD2/ACTR10/TRIP11/IFT140/KIF3A/SPG11/OPA1/ARHGAP21/MAPK8IP3/BBS12/MAPT/MGARP/DYNC1H1/KIF17/ARMCX3/TTLL1/COPG2/IFT74/DYNC2LI1/IFT27/NME5/FEZ1/AP3M2/TTC30A/IFT172/IFT52/KIF5C/KLC3/DYNC1I1/APP/ARL8A/LCA5/KATNIP/TTC21B/TRAK1/DYNC2H1/SFPQ/DYNC2I1/SUN2/FYCO1/TRAF3IP1/PURA/RFX3/DYNLL2/OFD1/TERF2/SYNE2/IFT22/RHOT1/SPEF2/TUB/CLUAP1/DST/ARL3/TRAK2/DYNLRB2/NPHP3/MAP2/CAMSAP3/JHY/PCM1/AP3S1/IFT122/WDR35/MAP1A/PAFAH1B1/WDR19/TMEM108/SPAG16/KIF13A/DLG2/SYBU/SOD1/DYNLRB1/MAP1B |
| GOBP | PROTEIN INSERTION INTO MITOCHONDRIAL INNER MEMBRANE | 11 | 0.685545249 | 1.701286769 | 0.007900716 | 0.038048037 | 0.026577169 | 1397 | tags=45%, list=7%, signal=42% | TIMM8B/NDUFA13/TIMM13/TIMM10/ROMO1 |
| GOBP | MODULATION BY HOST OF VIRAL GENOME REPLICATION | 17 | 0.608193923 | 1.697022153 | 0.007791364 | 0.037548337 | 0.02622812 | 1632 | tags=35%, list=8%, signal=33% | ZC3H12A/IFI27/PHB/VAPA/HSPA8/CCL8 |
| GOBP | DICARBOXYLIC ACID BIOSYNTHETIC PROCESS | 14 | 0.650021549 | 1.717409444 | 0.007748289 | 0.037367558 | 0.026101843 | 1948 | tags=36%, list=9%, signal=32% | KYNU/GOT2/GOT1/MTHFD1/MTHFD1L |
| GOMF | ENDOPEPTIDASE REGULATOR ACTIVITY | 178 | 0.326911481 | 1.400650985 | 0.007723534 | 0.037274931 | 0.026037142 | 2538 | tags=20%, list=12%, signal=18% | SERPINB3/PI3/SERPINB4/PSME2/SERPINB13/PTTG1/LTF/SERPINB1/A2ML1/BIRC5/SERPINA1/CSTB/SLPI/PSME1/CSTA/CST7/PSENEN/PSMD14/GAPDH/SPOCK2/SERPINA3/UCHL5/WFDC12/SERPINB9/RPS6KA1/SERPINB8/SPINT2/BIRC3/CD27/PSMF1/ADRM1/PSME3/CARD16/PRDX5/USP14/FURIN |
| GOBP | TRANSCRIPTION ELONGATION FROM RNA POLYMERASE I PROMOTER | 29 | 0.535126939 | 1.679245446 | 0.007706807 | 0.037220939 | 0.025999427 | 5583 | tags=55%, list=27%, signal=40% | POLR2H/POLR2F/POLR1H/POLR2L/POLR1C/TAF1D/POLR1B/CDK7/POLR2E/POLR1G/CCNH/POLR1A/TAF1B/GTF2H1/ERCC6/TAF1A |
| GOBP | RESPONSE TO X RAY | 32 | 0.49806529 | 1.607871036 | 0.007680945 | 0.037122724 | 0.025930823 | 3226 | tags=47%, list=15%, signal=40% | RAD51/CDKN1A/XRCC6/BLM/XRCC5/BRCA2/THBD/BRCC3/XRCC4/LIG4/NIPBL/ERCC8/ERCC1/ANXA1/CASP3 |
| GOBP | NEGATIVE REGULATION OF PRODUCTION OF MOLECULAR MEDIATOR OF IMMUNE RESPONSE | 39 | 0.476771437 | 1.599245499 | 0.007636047 | 0.036932297 | 0.025797806 | 5281 | tags=51%, list=25%, signal=38% | ARG1/JAK3/HMOX1/PRKDC/LILRB4/BST2/TNF/XCL1/IRAK3/CD96/HLA-F/LILRB1/IL10/FOXP3/PARP3/CR1/IL33/TBX21/IFNA2/SPINK5 |
| GOBP | NUCLEAR ENVELOPE ORGANIZATION | 52 | 0.446801736 | 1.601398646 | 0.007615412 | 0.036859031 | 0.025746629 | 4021 | tags=44%, list=19%, signal=36% | CCNB1/CDK1/CCNB2/PPP2CA/ZMPSTE24/PLK1/PPP2R2A/TOR1A/REEP4/VRK1/TARDBP/NDEL1/NSFL1C/NUP155/LEMD2/NEK6/ANKLE2/PPP2R1A/CNEP1R1/TOR1B/EMD/CHMP2A/NUP93 |
| GOBP | ENDOPLASMIC RETICULUM TO CYTOSOL TRANSPORT | 28 | 0.529630563 | 1.66168866 | 0.007607109 | 0.036845389 | 0.0257371 | 5184 | tags=61%, list=25%, signal=46% | VCP/HM13/UFD1/SEC61B/BCAP31/EDEM1/DERL2/AUP1/EDEM2/UBAC2/TMEM129/NPLOC4/YOD1/UBE2J1/HSP90B1/SELENOS/DERL1 |
| GOBP | CARDIAC VENTRICLE DEVELOPMENT | 113 | -0.333807488 | -1.458256837 | 0.007601379 | 0.036844203 | 0.025736272 | 4733 | tags=35%, list=23%, signal=27% | SEMA3C/WNT11/HEY1/NPRL3/MED1/TMEM65/TGFB2/KCNK2/SAV1/FOXC2/PPP1R13L/BMP4/ROBO2/HES1/JAG1/MATR3/TBX3/LMO4/SOX4/SLIT2/NOG/CPE/NPY5R/SLIT3/DSP/FOXC1/FGFR2/BMPR1A/MEF2C/MYOCD/PDE2A/RBPJ/MDM4/TGFBR2/ZFPM2/TNNC1/TPM1/TGFBR3/GATA3 |
| GOBP | CELL DIFFERENTIATION INVOLVED IN METANEPHROS DEVELOPMENT | 25 | -0.530994509 | -1.714560538 | 0.007536221 | 0.036554754 | 0.025534086 | 4763 | tags=52%, list=23%, signal=40% | FAT4/PDGFB/LGR4/BMP4/WWTR1/YAP1/ADIPOQ/LIF/GREM1/POU3F3/LAMB2/CD34/OSR1 |
| GOBP | REGULATION OF LEUKOCYTE MIGRATION | 194 | 0.320619527 | 1.390509518 | 0.007514225 | 0.036474394 | 0.025477953 | 4773 | tags=37%, list=23%, signal=28% | C10orf99/WNT5A/NOD2/CCL20/C1QBP/MYD88/S100A7/PYCARD/CCR7/CXCL13/PTAFR/SMPD3/CXCL10/CXCL8/CCL4/ADAM17/S100A14/RHOH/MIF/CCL2/LYN/HMOX1/SLAMF8/DEFB124/CCL8/SELE/RAC2/IL6/ITGA4/RIPK3/LGALS9/CCL7/CCL19/CXCL17/IL23A/DOCK8/OXSR1/ADAM10/ADAM8/FADD/WASL/RHOG/BDKRB1/CCL5/TNF/TREM2/XCL1/CCR1/ANXA1/ICAM1/CCR2/GCSAM/SELP/ST3GAL4/LGMN/TMEM102/P2RX4/SERPINE1/CD300A/MSN/THY1/GPSM3/C5AR2/AKIRIN1/C3AR1/CXCR3/IL33/AIF1/AGER/AIRE/MAPK3 |
| GOBP | REGULATION OF NEURON MIGRATION | 37 | -0.475049953 | -1.690434379 | 0.007494068 | 0.036402854 | 0.025427982 | 4590 | tags=51%, list=22%, signal=40% | DAB2IP/SHTN1/SEMA3A/FLRT2/ULK4/IGSF10/LRIG2/PHACTR1/ARHGEF2/TNN/ERBB4/ZNF609/NRG3/GNRH1/SEMA6A/NEXMIF/RAPGEF2/FBXO31/SRGAP2 |
| GOMF | ACTIVIN BINDING | 15 | -0.619019238 | -1.742705436 | 0.007466082 | 0.036293152 | 0.025351354 | 3980 | tags=53%, list=19%, signal=43% | SMURF1/ACVR2B/FSTL3/ACVRL1/TGFBR2/ACVR1B/FST/TGFBR3 |
| GOMF | S ADENOSYLMETHIONINE DEPENDENT METHYLTRANSFERASE ACTIVITY | 155 | 0.3429432 | 1.451492392 | 0.007454201 | 0.036261638 | 0.02532934 | 4688 | tags=36%, list=22%, signal=28% | EZH2/SMYD2/WDR4/DIMT1/COMTD1/PRMT5/DNMT1/NOP2/FTSJ1/PRMT3/METTL6/METTL1/ICMT/TRMT10C/DNMT3B/TFB2M/PRMT6/SUV39H2/EMG1/COQ3/NSD2/NSUN2/RRP8/CMTR2/TRMT6/TRMT112/TPMT/SETD4/METTL8/LCMT1/CARNMT1/ANTKMT/CSKMT/DOT1L/MEN1/MECOM/ARMT1/PRMT1/MRM2/TRMT1/METTL4/EED/MRM3/THUMPD3/DNMT3A/FDXACB1/METTL22/BUD23/NSD1/PRMT2/METTL21EP/TRMT5/METTL5/NDUFAF7/SUV39H1/SETD9 |
| GOBP | MUSCLE ORGAN MORPHOGENESIS | 69 | -0.374522419 | -1.50732763 | 0.007396559 | 0.036007307 | 0.025151686 | 4332 | tags=32%, list=21%, signal=25% | MED1/S1PR1/TGFB2/BMP2/EFEMP2/FOXC2/SHOX2/NOG/ACTC1/LIF/DSP/FOXC1/FGFR2/BMPR1A/ADARB1/WNT2/RBPJ/ZFPM2/MYLK/TNNC1/TPM1/TGFBR3 |
| GOBP | EYE MORPHOGENESIS | 140 | -0.313795968 | -1.420373165 | 0.007347336 | 0.035793619 | 0.025002422 | 3430 | tags=28%, list=16%, signal=23% | NECTIN3/IFT172/COL8A1/MFAP2/MFSD2A/BMP4/RORB/TFAP2A/JAG1/AHI1/PTPRM/DIO3/NKD1/SOX8/TSKU/SP3/TWIST1/MFAP5/LRP6/NTRK2/COL8A2/PTN/RBP4/ZHX2/SKI/WNT2/ABI2/IFT122/BMP7/TFAP2B/EFEMP1/CDON/NOTCH2/GLI3/C12orf57/BCL2/MEIS1/BCAR3/WNT2B |
| GOBP | REGULATION OF SMOOTHENED SIGNALING PATHWAY | 76 | -0.38376571 | -1.579076599 | 0.007313684 | 0.035655538 | 0.02490597 | 5948 | tags=51%, list=29%, signal=37% | KIF7/TTC23/GLI1/MGRN1/INTU/RAB34/SFRP1/PDCL/IFT81/IFT140/FBXL17/TXNDC15/FOXA1/KCTD6/PRKACB/C2CD3/PRRX1/BTRC/IFT172/TCTN1/ENPP1/DYNC2H1/SHOX2/EVC/CTNNA1/CIBAR1/GLIS2/TULP3/CREBBP/FGFR2/GPC3/MOSMO/IFT122/DLG5/PTCH1/GAS1/GLI2/GLI3/RORA |
| GOBP | ACTIVATION OF NF KAPPAB INDUCING KINASE ACTIVITY | 18 | 0.612911915 | 1.738120459 | 0.007279088 | 0.035512648 | 0.024806158 | 4438 | tags=50%, list=21%, signal=39% | CARD14/IRAK1/TNFRSF10A/TNFRSF10B/TNFSF15/CARD10/TRAF4/TRAF2/MALT1 |
| GOBP | T HELPER CELL LINEAGE COMMITMENT | 17 | 0.609939907 | 1.701893912 | 0.007276244 | 0.035512648 | 0.024806158 | 6750 | tags=82%, list=32%, signal=56% | STAT3/IL12B/IL6/IL23A/BATF/IL12RB1/MTOR/TBX21/SLAMF6/IL6R/IRF4/TNFSF18/IL23R/LY9 |
| GOBP | NEGATIVE REGULATION OF OSTEOBLAST DIFFERENTIATION | 44 | -0.438920517 | -1.614370984 | 0.007252737 | 0.035435555 | 0.024752308 | 5583 | tags=52%, list=27%, signal=38% | HDAC7/SFRP1/GDF10/BAMBI/TWIST2/CDK6/HDAC4/TOB1/CRIM1/LRP5/TNN/VEGFC/RORB/NOG/TWIST1/PPARG/GREM1/IGFBP5/SKI/SMAD3/PTCH1/NBR1/AXIN2 |
| GOMF | VINCULIN BINDING | 11 | -0.684424819 | -1.751505334 | 0.007244146 | 0.035419338 | 0.02474098 | 3136 | tags=73%, list=15%, signal=62% | PXN/CTNNA1/SORBS3/DAG1/SYNM/CORO2B/DMD/UTRN |
| GOBP | NEPHRIC DUCT DEVELOPMENT | 13 | -0.668905083 | -1.821517517 | 0.007161261 | 0.035039584 | 0.024475716 | 3069 | tags=69%, list=15%, signal=59% | EPHA7/WNT11/BMP4/AHI1/PKD2/GPC3/OSR1/PKD1/GATA3 |
| GOBP | MODULATION BY SYMBIONT OF HOST CELLULAR PROCESS | 23 | 0.566006685 | 1.679926968 | 0.007159852 | 0.035039584 | 0.024475716 | 3477 | tags=48%, list=17%, signal=40% | KPNA2/ATG5/SERPINB9/ATG7/CASP8/TNIP1/KPNB1/SCRIB/CPSF4/PHB2/KPNA3 |
| GOBP | REGULATION OF GLUCOSE IMPORT | 53 | -0.417413878 | -1.596713784 | 0.007097541 | 0.034778471 | 0.024293324 | 2867 | tags=32%, list=14%, signal=28% | ENPP1/INSR/LEP/ADIPOQ/RHOQ/PRKAG2/SLC25A27/GPC3/OSBPL8/PID1/KLF15/IRS1/PIK3R1/CREBL2/SORBS1/IRS2/MEF2A |
| GOBP | INTERSTRAND CROSS LINK REPAIR | 54 | 0.449055572 | 1.626645625 | 0.007081093 | 0.034723198 | 0.024254715 | 3338 | tags=33%, list=16%, signal=28% | UBE2T/VCP/RPA3/RAD51/FANCI/CENPX/RAD51AP1/FANCD2/CENPS/RNF168/NEIL3/FANCG/FAAP20/ERCC1/RNF8/EME1/RFWD3/MCM9 |
| GOCC | SPINDLE POLE CENTROSOME | 15 | 0.634815707 | 1.717074019 | 0.007073473 | 0.034711171 | 0.024246314 | 2583 | tags=53%, list=12%, signal=47% | DLGAP5/AURKB/AURKA/NDE1/NPM1/SPOUT1/NUBP2/NSFL1C |
| GOBP | DNA LIGATION | 15 | 0.635384856 | 1.718613474 | 0.007073473 | 0.034711171 | 0.024246314 | 5259 | tags=73%, list=25%, signal=55% | POLB/RAD51/TOP2A/XRCC6/XRCC4/LIG4/APTX/TFIP11/PARP3/LIG1/APLF |
| GOCC | MICROTUBULE ORGANIZING CENTER ATTACHMENT SITE | 11 | -0.685207603 | -1.753508548 | 0.007072689 | 0.034711171 | 0.024246314 | 2358 | tags=64%, list=11%, signal=56% | CLMN/SYNE4/SUN2/SYNE2/SYNE3/SUN1/SYNE1 |
| GOBP | NEGATIVE REGULATION OF NEURON DIFFERENTIATION | 60 | -0.397184184 | -1.544110171 | 0.007061812 | 0.034711171 | 0.024246314 | 3524 | tags=37%, list=17%, signal=31% | DLL1/IRX3/HMG20A/HEY1/MED1/MIB1/APP/HES1/DTX1/JAG1/ZNF536/SOX8/EIF4ENIF1/CNTN4/NEPRO/PBX1/GSK3B/ZHX2/BMP7/DIXDC1/GLI3/MEIS1 |
| GOMF | VASCULAR ENDOTHELIAL GROWTH FACTOR RECEPTOR BINDING | 12 | -0.661476422 | -1.752642465 | 0.007028264 | 0.034590387 | 0.024161944 | 5826 | tags=83%, list=28%, signal=60% | CCDC88A/ITGB3/CD2AP/CDH5/DAB2IP/VEGFC/ITGA5/VEGFB/PGF/GREM1 |
| GOBP | REGULATION OF MITOCHONDRIAL OUTER MEMBRANE PERMEABILIZATION INVOLVED IN APOPTOTIC SIGNALING PATHWAY | 43 | 0.4744971 | 1.627710603 | 0.006996799 | 0.034460772 | 0.024071406 | 5111 | tags=53%, list=24%, signal=40% | TFDP1/SLC25A5/GZMB/BAK1/NMT1/YWHAQ/SFN/BID/CASP8/TMEM14A/BOK/TP63/YWHAZ/BAX/HIP1R/TMEM102/MAPK8/GSK3A/ATP5IF1/CHCHD10/PMAIP1/MUL1/YWHAH |
| GOBP | PYRIMIDINE NUCLEOSIDE TRIPHOSPHATE METABOLIC PROCESS | 22 | 0.567912104 | 1.681144578 | 0.006946575 | 0.034238513 | 0.023916155 | 1837 | tags=36%, list=9%, signal=33% | UCK2/NME1/CTPS1/TYMS/ENTPD7/CMPK2/DTYMK/DCTPP1 |
| GOCC | SYNAPTIC CLEFT | 17 | -0.598759509 | -1.735481079 | 0.006944098 | 0.034238513 | 0.023916155 | 3449 | tags=41%, list=17%, signal=34% | LAMA2/LGI1/CBLN1/NLGN1/APOE/LAMB2/LAMA5 |
| GOBP | REGULATION OF MORPHOGENESIS OF AN EPITHELIUM | 59 | -0.402915283 | -1.569311068 | 0.006937597 | 0.034238513 | 0.023916155 | 3622 | tags=37%, list=17%, signal=31% | TACSTD2/WNT5B/LGR4/FGF1/ABL1/BMP4/AGT/NTN4/BTBD7/NKD1/NOG/SOX8/ESR1/PDGFA/EGF/LIF/GREM1/WNT2/BMP7/AR/WNT2B/GATA3 |
| GOBP | TOLL LIKE RECEPTOR 9 SIGNALING PATHWAY | 26 | 0.555649581 | 1.702508826 | 0.00689932 | 0.03408061 | 0.023805857 | 3931 | tags=42%, list=19%, signal=34% | MYD88/IRAK2/RSAD2/IRAK1/TNIP2/EPG5/PTPN22/LILRA4/TLR8/PIK3AP1/IRAK4 |
| GOBP | MACROPHAGE ACTIVATION INVOLVED IN IMMUNE RESPONSE | 18 | 0.614192702 | 1.741752568 | 0.00689932 | 0.03408061 | 0.023805857 | 4807 | tags=56%, list=23%, signal=43% | NMI/IFI35/IFNG/SBNO2/SYK/GRN/TREM2/TYROBP/IL33/TICAM1 |
| GOBP | BINDING OF SPERM TO ZONA PELLUCIDA | 41 | 0.483401952 | 1.645474889 | 0.006836011 | 0.033817613 | 0.023622149 | 3468 | tags=24%, list=17%, signal=20% | CCT5/CCT2/CCT3/CCT7/ALDOA/TEX101/VDAC2/TCP1/CCT8/SPA17 |
| GOMF | LIPOPOLYSACCHARIDE BINDING | 29 | 0.536853502 | 1.684663457 | 0.00682963 | 0.033810944 | 0.023617491 | 4098 | tags=45%, list=20%, signal=36% | LTF/PTAFR/TLR2/CD6/PSMA1/RNASE7/CAMP/HSPD1/LY96/TREM2/SELP/BPIFC/TRIL |
| GOBP | REGULATION OF ORGAN GROWTH | 85 | -0.367276761 | -1.534382214 | 0.006799027 | 0.033684265 | 0.023529004 | 4035 | tags=34%, list=19%, signal=28% | KCNK2/PARP2/WWC3/SAV1/PTEN/FOXC2/VGLL4/ERBB4/JARID2/RGS2/YAP1/EDN1/STK3/NOG/LATS2/FOXC1/FGFR2/BMPR1A/PPARA/MEF2C/RBP4/WNT2/RBPJ/MAPK1/TGFBR2/ZFPM2/MEIS1/SOD1/TGFBR3 |
| GOCC | INFLAMMASOME COMPLEX | 16 | 0.638025364 | 1.76675527 | 0.006796401 | 0.033684265 | 0.023529004 | 3769 | tags=56%, list=18%, signal=46% | CASP4/PYCARD/CASP1/AIM2/CASP5/GSDMD/DHX33/NLRC4/NLRP3 |
| GOBP | OLIGOPEPTIDE TRANSPORT | 13 | 0.660608472 | 1.711563455 | 0.006784949 | 0.033664172 | 0.023514968 | 4824 | tags=62%, list=23%, signal=47% | SLC7A11/ABCC1/SLC15A1/SLC9A3R1/SLC15A3/GJA1/ABCC5/SLC15A4 |
| GOBP | POSITIVE REGULATION OF NOTCH SIGNALING PATHWAY | 53 | -0.42021231 | -1.607418494 | 0.006777358 | 0.03365136 | 0.023506019 | 3048 | tags=36%, list=15%, signal=31% | ROBO2/HES1/JAG1/YAP1/SNW1/IL6ST/CCN3/JAG2/TSPAN14/NEPRO/ENHO/CREBBP/TM2D3/ZMIZ1/RBPJ/NOTCH2NLA/KIT/EPN2/MAML2 |
| GOBP | DISTAL TUBULE DEVELOPMENT | 12 | -0.665070065 | -1.76216415 | 0.006775755 | 0.03365136 | 0.023506019 | 3220 | tags=50%, list=15%, signal=42% | KLHL3/JAG1/POU3F3/PKD2/TFAP2B/PKD1 |
| GOMF | RNA HELICASE ACTIVITY | 73 | 0.411527315 | 1.552255756 | 0.006668513 | 0.033159931 | 0.023162748 | 5778 | tags=48%, list=28%, signal=35% | EIF4A3/DDX39A/IFIH1/DDX41/HELZ2/DDX28/DDX60/DDX60L/DDX56/BRIP1/DHX58/DDX52/AQR/DDX49/DHX33/SKIV2L/RAD54B/DDX21/DDX58/DDX19A/DHX32/DHX29/UPF1/DHX15/DDX46/DHX57/TDRD9/DHX34/DHX37/DDX20/SUPV3L1/DDX43/DDX23/DDX3X/DDX54 |
| GOBP | NEGATIVE REGULATION OF LEUKOCYTE MEDIATED CYTOTOXICITY | 21 | 0.58730704 | 1.711666444 | 0.006655376 | 0.033119121 | 0.023134241 | 5020 | tags=62%, list=24%, signal=47% | SERPINB4/SERPINB9/IL7R/LGALS9/HLA-E/KLRD1/HLA-F/HLA-B/LILRB1/HLA-G/PTPRC/ARRB2/HLA-A |
| GOBP | ACTIVATED T CELL PROLIFERATION | 42 | 0.48346129 | 1.65024294 | 0.00665454 | 0.033119121 | 0.023134241 | 4624 | tags=48%, list=22%, signal=37% | CD24/CD274/PYCARD/ARG1/IL12B/RIPK3/LGALS9/IL23A/IL2RA/FADD/LILRB4/TNFSF9/CRTAM/SCRIB/IL12RB1/CASP3/FOXP3/EPO/BTN3A1/AGER |
| GOBP | NEGATIVE REGULATION OF LIPID METABOLIC PROCESS | 88 | -0.35837881 | -1.528354569 | 0.006651461 | 0.033119121 | 0.023134241 | 4662 | tags=39%, list=22%, signal=30% | ABCA2/PDGFB/DAB2IP/INSIG1/ERLIN2/SORL1/BMP2/PIP4K2A/GPER1/SLC27A1/NCOR1/MFSD2A/WASHC1/SIRT1/APOC1/APOD/PDGFA/ENHO/AKR1C3/DKK3/APOE/DGAT2/ORMDL1/PIBF1/PIK3IP1/ORMDL3/FBXW7/PIP4K2B/CRTC3/CCDC3/ADRA2A/SOD1/SERPINA12/ACADL |
| GOBP | IRON SULFUR CLUSTER ASSEMBLY | 23 | 0.567102335 | 1.683178896 | 0.006643841 | 0.033119121 | 0.023134241 | 3788 | tags=48%, list=18%, signal=39% | GLRX3/CIAPIN1/CIAO2A/HSPA9/NUBP1/CIAO2B/NDOR1/NUBP2/LYRM4/FXN/CIAO3 |
| GOMF | 4 IRON 4 SULFUR CLUSTER BINDING | 40 | 0.480510179 | 1.621750791 | 0.006578399 | 0.032833347 | 0.022934623 | 5661 | tags=50%, list=27%, signal=37% | CIAPIN1/RSAD2/NDUFS7/SDHB/NUBP1/BRIP1/ACO2/NDUFS8/DNA2/NDUFV1/NUBP2/NDUFS2/CIAO3/NDUFS1/POLD1/ETFDH/NTHL1/PPAT/PRIM2/CDK5RAP1 |
| GOBP | PYRIMIDINE DEOXYRIBONUCLEOTIDE METABOLIC PROCESS | 22 | 0.568192492 | 1.681974587 | 0.006564206 | 0.032786867 | 0.022902157 | 1935 | tags=36%, list=9%, signal=33% | TYMP/UPP1/TYMS/CMPK2/DTYMK/TDG/DCTPP1/SMUG1 |
| GOBP | RESPONSE TO UV | 143 | 0.348248886 | 1.45692384 | 0.006549132 | 0.032735913 | 0.022866564 | 4890 | tags=42%, list=23%, signal=32% | PCLAF/SERPINB13/PBK/AURKB/IVL/PCNA/RUVBL2/BAK1/MAPK13/HUS1/EIF2S1/MMP9/FEN1/BCL3/CDC25A/COPS9/DTL/MYC/MMP1/IL12B/CDKN1A/GPX1/UBE2A/ST20/BRCA2/SMPD1/NOC2L/CARD16/NPM1/ATF4/RAD18/TRIAP1/MEN1/TMEM161A/ERCC8/PML/BAX/ERCC1/PARP1/CASP3/PRKCD/OPN3/POLD3/KDM1A/DCUN1D3/MAPK8/CDKN2D/ATR/HYAL3/TIPIN/POLD1/MMP3/NEDD4/TYR/CASP9/BRSK1/SPRTN/USP28/RHNO1/OPN5 |
| GOBP | T CELL DIFFERENTIATION INVOLVED IN IMMUNE RESPONSE | 71 | 0.414499345 | 1.552264383 | 0.006530542 | 0.0326673 | 0.022818637 | 3986 | tags=39%, list=19%, signal=32% | ZC3H12A/NFKBIZ/IL4R/ENTPD7/BCL3/STAT3/JAK3/IFNG/RELB/IL12B/IL6/LGALS9/CCL19/IL23A/BATF/GPR183/CD86/EOMES/LEF1/SEMA4A/NFKBID/ANXA1/HLA-DRA/IL12RB1/NLRP3/MTOR/CD80/FOXP3 |
| GOBP | POSITIVE REGULATION OF INTERLEUKIN 17 PRODUCTION | 18 | 0.617311956 | 1.750598274 | 0.006519552 | 0.032636623 | 0.022797208 | 6750 | tags=83%, list=32%, signal=56% | NOD2/MYD88/PRKCQ/SLC7A5/IL12B/IL6/IL23A/IL15/SPHK1/SLAMF6/CCL1/IL23R/IL21/TGFB1/LY9 |
| GOMF | INTERLEUKIN 1 RECEPTOR BINDING | 17 | 0.61427423 | 1.713987821 | 0.006503567 | 0.032580884 | 0.022758274 | 3931 | tags=59%, list=19%, signal=48% | IL36G/MYD88/IL36RN/IL36A/TOLLIP/IL1B/IL1RN/IL36B/TRIP6/IRAK4 |
| GOCC | ENDORIBONUCLEASE COMPLEX | 30 | 0.530399572 | 1.673432181 | 0.006486457 | 0.032519419 | 0.022715339 | 4342 | tags=50%, list=21%, signal=40% | TARBP2/HSD17B10/TRMT10C/RPP40/ZFP36/POP5/TSEN54/POP4/CLP1/TSEN34/POP1/POP7/RPP30/RPP38/TSEN2 |
| GOBP | CARBOHYDRATE DERIVATIVE TRANSPORT | 81 | 0.400166796 | 1.539198672 | 0.006485006 | 0.032519419 | 0.022715339 | 5022 | tags=37%, list=24%, signal=28% | SLC5A1/SLC25A5/SLC35B1/ALOX12B/SLC28A3/ABCG1/GLTP/P2RY2/ATG5/SLC25A25/RFT1/VAMP8/SLC37A4/SLC37A2/SLC25A19/SLC19A1/SLC35A4/SLC35B4/SLC15A3/SLC35D2/SLC50A1/VLDLR/G6PC1/GJA1/CR1/SLC35A2/PLTP/SLC15A4/CLN3/SLC29A4 |
| GOBP | ETHER BIOSYNTHETIC PROCESS | 11 | -0.688949229 | -1.76308371 | 0.006472589 | 0.032498397 | 0.022700656 | 4689 | tags=64%, list=22%, signal=49% | FASN/AGPS/PLA2G4C/LPCAT2/FAR1/GNPAT/CHPT1 |
| GOBP | EPITHELIAL CELL PROLIFERATION | 372 | -0.258127916 | -1.319375508 | 0.006441499 | 0.032366486 | 0.022608513 | 3663 | tags=30%, list=18%, signal=25% | SAV1/AGGF1/SCARB1/BTRC/MAP2K5/TNFSF12/VASH2/PTEN/NRP1/LGR4/IFT172/VEGFC/IFT52/COL8A1/FGF1/EGR3/DLG1/SIRT1/BMP4/PTPRK/JCAD/HES1/PRKD1/RGCC/CXCL12/EGFL7/BCL11B/VEGFB/YAP1/PTPRM/STAT5A/LEP/MST1/NOG/CCN3/CD109/PRKCA/SRSF6/ZNF304/PGF/WDR48/LGR5/PURA/TWIST1/ESR1/FGF9/C6orf89/IRF6/DUSP10/PPARG/EGF/EGFR/KRT2/PGR/ACVRL1/FZD7/SYNJ2BP/APOE/FABP7/MTSS1/FGFR2/KLF9/TNMD/ZFP36L1/CDC73/BMPR1A/NR2F2/COL8A2/FGFR1/LIMS2/RICTOR/GPC3/PTN/MEF2C/IGFBP5/AKT3/ALDH1A2/WNT2/NFIB/ARNT/SMAD3/EPPK1/CD34/RTN4/LAMC1/ATOH8/MAPK1/FBXW7/SP1/GATA2/OSR1/PTCH1/CDKN1C/NUPR1/AGTR1/TCF7L2/FST/MCC/ZNF703/AR/NOTCH2/CAV2/KIT/CAV1/OSR2/TGFBR3/CCND1/ANG/GATA3/F3/CLDN1 |
| GOMF | AROMATIC AMINO ACID TRANSMEMBRANE TRANSPORTER ACTIVITY | 10 | 0.712291067 | 1.720119446 | 0.006413717 | 0.032251012 | 0.022527853 | 3694 | tags=70%, list=18%, signal=58% | SLC7A1/SLC16A10/SLC7A5/SLC38A5/SLC3A2/SLC38A7/SLC36A4 |
| GOCC | NUCLEAR ENVELOPE | 445 | 0.279258002 | 1.307631396 | 0.006410561 | 0.032251012 | 0.022527853 | 4196 | tags=31%, list=20%, signal=25% | RAN/ATP5MF/IFI27/GCH1/JPT1/CLCA2/LRRC59/MX1/MAD2L1/LMNB2/VRK2/SMOX/CENPF/NUP37/APEH/VAPA/NUP210/ZMPSTE24/DTL/TXNL4A/RANBP1/DUSP2/TMPO/FBXW11/AEN/NUP88/GAPDH/NUP62CL/MRPS14/GTPBP4/FAM169A/MX2/BRIP1/MRPL19/HAX1/ALG14/RRP12/EBP/RRM1/NUP50/XPOT/RAC2/RCC1/NOC4L/HTATIP2/TOR1A/NUDT1/NXT1/CCND2/CETN2/TOR3A/MAD2L1BP/NPC1/LMNB1/CSE1L/RANGAP1/LYPLA1/ACKR2/RAE1/MVP/TOR4A/BNIP3/SEC13/PTGER3/BOK/DCTN5/KPNB1/SULT1E1/TOR2A/NDEL1/MRPS23/NUP155/MGST2/AGPAT5/SLC16A3/LEMD2/MLIP/PLRG1/LBR/MLX/INTS5/CPTP/NDC1/BAX/YBX1/ABCF1/MFSD10/PARP1/WDR3/DNAJC2/SIGMAR1/GLE1/RNF123/NUP188/ITPRIP/ROGDI/LMO7/SHISA5/CPNE1/PTGES/NUP85/CNEP1R1/KPNA3/EI24/NUP205/TMEM201/UBE2I/NUTF2/TMEM176B/EIF5A/CBX3/TOR1B/AGFG1/P2RX4/CDK4/TOR1AIP2/TRA2B/SREBF1/ENY2/ZC3HC1/ALOX5AP/NUP58/EMD/LRPPRC/MTOR/CHMP2A/TMC8/NUP107/TMEM33/IL15RA/NUP93/NAV3/TMC6/DNAJB12/POM121/MYO6/YEATS4 |
| GOBP | METANEPHRIC TUBULE DEVELOPMENT | 21 | -0.570810729 | -1.755761004 | 0.006389881 | 0.032179325 | 0.022477779 | 3438 | tags=52%, list=16%, signal=44% | LGR4/WNT7B/HES1/WWTR1/YAP1/SOX8/LIF/POU3F3/PKD2/OSR1/PKD1 |
| GOMF | EXTRACELLULAR MATRIX BINDING | 54 | -0.423465508 | -1.620549984 | 0.006387261 | 0.032179325 | 0.022477779 | 3784 | tags=39%, list=18%, signal=32% | ELN/ITGAV/ADAMTS5/BCAM/SMOC2/NTN4/GPC1/FBLN2/CLEC14A/SSC5D/SLIT2/ITGB1/LGALS1/CD248/DAG1/DCN/SPARCL1/ITGA9/LGALS3/OLFML2A/TGFBI |
| GOBP | REGULATION OF EPITHELIAL TO MESENCHYMAL TRANSITION | 92 | -0.366339211 | -1.568530453 | 0.006335002 | 0.031950856 | 0.022318189 | 5115 | tags=48%, list=25%, signal=36% | GLIPR2/SMAD4/FOXA1/ALX1/BAMBI/DAB2IP/PHLDB2/CTNNB1/TGFB2/BMP2/LDLRAD4/USF3/PTEN/GCNT2/SPRED2/BMP4/DAB2/RGCC/DACT3/JAG1/WWTR1/CLASP1/NOG/SPRED1/EFNA1/TWIST1/FOXC1/GREM1/PHLDB1/ELL3/SDCBP/DAG1/SMAD3/FERMT2/TGFB1I1/TGFBR2/BMP7/IL17RD/SPRY1/TCF7L2/ZNF703/SPRY2/AXIN2/GATA3 |
| GOBP | RIBONUCLEOSIDE CATABOLIC PROCESS | 22 | 0.569040091 | 1.684483667 | 0.006309918 | 0.031848258 | 0.022246523 | 2659 | tags=32%, list=13%, signal=28% | UPP1/PNP/AHCY/APOBEC3B/HPRT1/XDH/ADA2 |
| GOCC | ENDOPLASMIC RETICULUM GOLGI INTERMEDIATE COMPARTMENT | 124 | 0.36673141 | 1.514953549 | 0.006301793 | 0.03183116 | 0.02223458 | 3424 | tags=26%, list=16%, signal=22% | GJB2/CTSC/PRRG4/RER1/SERPINA1/ERP44/TGFA/CNIH4/AREG/HSPA5/TAP1/PTPN2/TAP2/LMAN2/BCAP31/YIF1A/YIF1B/CNIH1/P4HB/GNPNAT1/YKT6/TRAPPC5/LAMP5/TMED5/SURF4/PDIA6/TAPBP/PROM1/MYDGF/LMAN1/TMEM199/TMED7 |
| GOMF | STEROID BINDING | 99 | -0.345820701 | -1.49089218 | 0.006281817 | 0.031754137 | 0.022180778 | 4188 | tags=34%, list=20%, signal=28% | INSIG1/ERLIN2/PMP2/PAQR6/SOAT1/GPER1/HSD11B2/PGRMC1/OSBPL5/RORC/ESRRG/NR1H3/APOD/CYP3A4/ESR1/PGR/OSBP/OSBPL9/CD81/OSBPL8/SCARB2/IRX5/STAR/OSBPL1A/PTCH1/ATP1A2/NR3C1/AR/CAV1/INSIG2/NR3C2/HSD11B1/PGRMC2/RORA |
| GOBP | POSITIVE REGULATION OF CELLULAR AMIDE METABOLIC PROCESS | 147 | 0.346482327 | 1.457618738 | 0.006273334 | 0.031735131 | 0.022167502 | 3964 | tags=36%, list=19%, signal=29% | NOD2/EIF4A3/C1QBP/PTAFR/EIF6/KRT17/SMPD3/BCL3/IFNGR1/CDC123/RPS6KB2/TARBP2/FASTKD2/TRUB2/PYM1/IFNG/ABCG1/TRMT10C/COA3/UQCC2/IL6/MPV17L2/POLR2G/PRKDC/NPM1/ELAVL1/POLR2D/JMJD4/SMPD2/EIF4G2/PASK/TNFRSF1A/MRPS27/LARP1/RCC1L/CCL5/NAT10/TNF/PKM/EIF4G1/CASP3/PRKCD/UHMK1/YTHDF2/EIF2B5/DAZL/EIF5A/FASTKD3/CDK4/DHX29/GSK3A/MTOR/UQCC1 |
| GOBP | ENDOCRINE HORMONE SECRETION | 43 | -0.452417917 | -1.66486461 | 0.006272923 | 0.031735131 | 0.022167502 | 6096 | tags=56%, list=29%, signal=40% | RAB8B/INHBA/TACR2/PTPN11/SMAD4/GDF9/C1QTNF3/CRY1/BMP6/TRPV6/TAC1/GAL/GNAS/AGT/TBX3/LEP/SELENOM/RAB11FIP5/RAB11FIP3/FOXD1/FZD4/AGTR1/INHBB/GATA3 |
| GOBP | PYRIMIDINE NUCLEOSIDE MONOPHOSPHATE METABOLIC PROCESS | 18 | 0.618814311 | 1.754858714 | 0.006266994 | 0.031735131 | 0.022167502 | 4210 | tags=44%, list=20%, signal=36% | TYMP/UPP1/UCK2/TYMS/UMPS/DCTD/CAD/NT5C |
| GOBP | NEGATIVE REGULATION OF ACTIN FILAMENT BUNDLE ASSEMBLY | 30 | -0.503073866 | -1.705093308 | 0.006262654 | 0.031735131 | 0.022167502 | 4553 | tags=53%, list=22%, signal=42% | PAK2/PHLDB2/S1PR1/FRMD7/PRKN/TACSTD2/WASF2/CLASP1/MET/SHANK3/ARHGAP6/MYOC/CORO2B/PIK3R1/DLC1/CGNL1 |
| GOBP | CELL MIGRATION INVOLVED IN SPROUTING ANGIOGENESIS | 52 | -0.424663974 | -1.61812664 | 0.006260242 | 0.031735131 | 0.022167502 | 4235 | tags=48%, list=20%, signal=38% | KDR/MIA3/HDAC9/HDAC5/MAP2K5/FOXC2/NRP1/EGR3/ABL1/SRPX2/JCAD/CLEC14A/SLIT2/SPRED1/MMRN2/GREM1/KLF4/AKT3/RHOJ/STARD13/MEOX2/FBXW7/GATA2/MAP3K3/EFNB2 |
| GOBP | DETECTION OF EXTERNAL BIOTIC STIMULUS | 22 | 0.570044629 | 1.687457319 | 0.006246503 | 0.031718824 | 0.022156111 | 5020 | tags=59%, list=24%, signal=45% | NOD2/CLEC7A/PGLYRP4/TLR2/PGLYRP2/LY96/TREM2/PGLYRP3/NLRC4/HLA-B/CD1D/TLR1/HLA-A |
| GOBP | REGULATION OF SKELETAL MUSCLE CELL DIFFERENTIATION | 18 | -0.592660243 | -1.721292211 | 0.006217272 | 0.031594278 | 0.022069114 | 5071 | tags=67%, list=24%, signal=51% | RBM24/TBX1/NR1D2/MSTN/GPC1/ARNTL/DDX17/MEF2C/MYOCD/NLN/EPHB1/CYP26B1 |
| GOBP | SMOOTHENED SIGNALING PATHWAY INVOLVED IN DORSAL VENTRAL NEURAL TUBE PATTERNING | 11 | -0.690411927 | -1.766826887 | 0.006215404 | 0.031594278 | 0.022069114 | 1698 | tags=64%, list=8%, signal=58% | TBC1D32/PRKACB/TULP3/PTCH1/WDR19/GLI2/GLI3 |
| GOBP | CYTOKINETIC PROCESS | 37 | 0.505301479 | 1.681906511 | 0.006193047 | 0.031518858 | 0.022016432 | 3946 | tags=41%, list=19%, signal=33% | KIF20A/CEP55/AURKB/ECT2/RACGAP1/ANLN/KIF20B/BIN3/PDCD6IP/MYH9/CHMP5/CHMP1B/CHMP6/CHMP1A/CHMP2A |
| GOBP | ORGANIC HYDROXY COMPOUND BIOSYNTHETIC PROCESS | 235 | 0.320864013 | 1.419556666 | 0.006170643 | 0.031428644 | 0.021953416 | 4693 | tags=36%, list=22%, signal=28% | RAN/CDH3/WNT5A/SLC7A11/GCH1/PTS/GOT1/MOXD1/PTAFR/CYP7B1/SPTLC2/IPPK/PNPO/SRD5A3/CYB5R2/SQLE/PLCG2/CH25H/CYP27B1/IFNG/ITPKC/NFKB1/ABCG1/HSD17B10/OSBPL2/IMPA2/STARD4/EBP/HSD17B4/PCK2/CTNS/DHFR/GIPC1/PMEL/SEC14L2/PLEK/LPCAT3/ARV1/KPNB1/NSDHL/PARK7/P2RY1/FAXDC2/ACACA/SREBF2/GPR37/TNF/LBR/G6PD/HMGCR/PSAT1/TYRP1/AGK/P2RY6/OPN3/PGP/SPTLC1/SPTLC3/CD244/PPIP5K1/CYP2R1/SREBF1/HSD17B1/AVPR1B/SPHK1/BAAT/NTSR1/ERG28/ACER3/ERLIN1/FDPS/SPR/PLPP6/HRH1/ELOVL6/ACOT8/FDFT1/ASAH2B/NFYC/PROX1/TYR/SLC45A2/CYP11B2/PLTP/NPC1L1 |
| GOBP | REGULATION OF BRANCHING INVOLVED IN URETERIC BUD MORPHOGENESIS | 20 | -0.582165531 | -1.76721812 | 0.00614308 | 0.031311999 | 0.021871937 | 3622 | tags=45%, list=17%, signal=37% | HOXB7/TACSTD2/LGR4/BMP4/AGT/NOG/SOX8/GREM1/WNT2B |
| GOBP | VENTRAL SPINAL CORD DEVELOPMENT | 41 | -0.471034141 | -1.706579728 | 0.006138738 | 0.031311999 | 0.021871937 | 3421 | tags=27%, list=16%, signal=22% | IFT172/TCTN1/LMO4/SOX4/DYNC2H1/ZC4H2/HOXC10/GATA2/PTCH1/GLI2/GLI3 |
| GOBP | ACTIN FILAMENT ORGANIZATION | 418 | -0.24888366 | -1.278317061 | 0.006127725 | 0.031281197 | 0.021850421 | 3843 | tags=28%, list=18%, signal=23% | ARHGEF10L/INPPL1/FAM171A1/ELN/TRIOBP/DMTN/PIK3CA/PRKN/TACSTD2/ARHGEF2/RDX/NRP1/PRKCE/TAC1/ASAP3/PXN/DLG1/WASHC1/ABL1/MICAL1/CCN2/TENM1/WASF2/RGCC/TMSB4X/CXCL12/FAM107A/SWAP70/CLASP1/S100A10/SLIT2/ELMO2/ARHGEF15/KANK4/RHOBTB1/RAPGEF3/ADD1/MET/ACTC1/NEDD9/FMN1/TMOD2/WASF3/ZBED3/ACTA1/CTNNA1/ALMS1/FER/PLEKHH2/TMSB15B/SHANK3/TESK1/HIP1/DSTN/ARHGAP12/BIN1/VILL/SORBS3/ARHGAP35/TTC17/PFN2/SPTAN1/RHOB/SORBS2/ARHGEF5/MCU/RICTOR/SH3KBP1/CALD1/EFS/AIF1L/ROCK2/ARHGAP6/SCIN/MYO5C/SMAD3/FERMT2/FHOD3/SVIL/DIAPH2/ABI2/SDC4/CFL2/MYOC/PAK3/TPM2/GDPD2/TSC1/LPAR1/EPS8/SPTBN2/LIMCH1/CORO2B/GAS7/NEBL/MYADM/PIK3R1/ADD3/LMOD1/ITGB5/DLC1/DBN1/DPYSL3/SYNPO2/TPM1/TMOD1/CGNL1/GSN/BCL2/SORBS1/ARHGEF10/KANK1/RHOBTB3/SPTBN1/ANG/COBL |
| GOBP | NEGATIVE REGULATION OF ADAPTIVE IMMUNE RESPONSE | 51 | 0.451450112 | 1.617232335 | 0.006117305 | 0.031251752 | 0.021829854 | 4902 | tags=43%, list=23%, signal=33% | ZC3H12A/NOD2/IL4R/SAMSN1/SUSD4/ARG1/JAK3/IL7R/LILRB4/XCL1/KLRD1/CLEC4G/HLA-F/LILRB1/HLA-G/FOXP3/PARP3/CR1/PTPRC/IL33/TBX21/IFNA2 |
| GOBP | LEYDIG CELL DIFFERENTIATION | 10 | -0.70083902 | -1.75528259 | 0.006116298 | 0.031251752 | 0.021829854 | 2491 | tags=50%, list=12%, signal=44% | MGST1/PLEKHA1/PDGFRA/AR/CCND1 |
| GOCC | 9PLUS0 NON MOTILE CILIUM | 120 | -0.327307568 | -1.442434361 | 0.006113236 | 0.031251752 | 0.021829854 | 5473 | tags=33%, list=26%, signal=25% | CCDC66/RPGR/CEP250/RGS9BP/CEP290/IFT140/OCRL/TOPORS/NPHP1/VCAN/NAPEPLD/IMPG1/KIF17/BBS7/PDE6A/SPATA7/GNAQ/KIAA1549/SHANK2/IFT52/MYRIP/NPHP4/SEPTIN2/LCA5/MERTK/GNB1/DHRS3/IQCB1/CFAP410/DYNLL2/TULP3/GNA11/ARL3/IFT122/CNGA1/C1orf115/WDR19/PHYH/MAP1B/CDHR1 |
| GOCC | TERTIARY GRANULE | 162 | 0.342429096 | 1.458403112 | 0.006107347 | 0.031251752 | 0.021829854 | 5459 | tags=41%, list=26%, signal=30% | TCN1/LTF/LRG1/CSTB/ILF2/CXCL1/GGH/PTAFR/PRSS3/MMP9/FPR1/CD53/RAP2B/ALDOA/LAMTOR2/DBNL/CD177/LYZ/PLAU/DYNLL1/CNN2/NIT2/ITGB2/CTSD/VAMP8/ADAM10/CLEC4C/CD58/GPR84/GSDMD/ADAM8/CAMP/CTSS/SLC2A3/VPS35L/QSOX1/CYFIP1/CYBA/KCNAB2/LAMTOR3/LILRB2/B2M/LAIR1/TBC1D10C/DIAPH1/ATAD3B/HP/CD300A/PTX3/TMC6/SERPINB12/CR1/OLR1/CANT1/FCER1G/SERPINB6/ATP6V0C/MCEMP1/STXBP2/CYBB/FPR2/ITGAX/SERPINB10/LAMTOR1/RAP2C/TMEM179B |
| GOBP | NUCLEOSIDE DIPHOSPHATE METABOLIC PROCESS | 150 | 0.357886994 | 1.50874652 | 0.006088341 | 0.031198686 | 0.021792786 | 4978 | tags=40%, list=24%, signal=31% | ENO1/TPI1/PGAM1/PGK1/AK2/HK2/NME1/TIGAR/ENTPD7/CMPK2/HIF1A/EIF6/DTYMK/NUP37/TJP2/NUP210/LDHA/ALDOA/FOXK2/NUP88/IFNG/GAPDH/NUDT5/GPI/AK1/GALK1/NUP50/RAE1/DHTKD1/SEC13/PFKFB2/PFKP/NUP155/CASK/PKM/NDC1/PFKFB4/ADPGK/NUP188/PRKAG1/NUP85/NUP205/CARD11/OGDHL/ENO3/PGK2/NME7/NUP58/NUP107/NUP93/POM121/HK3/NUP160/ZBTB7A/NUP35/ENTPD3/NUP42/ENTPD5/BPGM/NME6 |
| GOBP | NEGATIVE REGULATION OF PROTEIN POLYUBIQUITINATION | 10 | 0.715878827 | 1.728783567 | 0.006084811 | 0.031198686 | 0.021792786 | 3725 | tags=60%, list=18%, signal=49% | TRIP12/PPIA/OTUB2/PLAA/OTUB1/PARP10 |
| GOBP | REGULATION OF T CELL CHEMOTAXIS | 15 | 0.642330208 | 1.737399531 | 0.006084811 | 0.031198686 | 0.021792786 | 3616 | tags=73%, list=17%, signal=61% | WNT5A/S100A7/CXCL13/CXCL10/ADAM17/OXSR1/ADAM10/CCL5/XCL1/CCR2/TMEM102 |
| GOBP | DNA DAMAGE RESPONSE DETECTION OF DNA DAMAGE | 39 | 0.485473047 | 1.628433511 | 0.006072798 | 0.03119041 | 0.021787005 | 4746 | tags=51%, list=23%, signal=40% | MRPS11/PCNA/RPA3/DTL/PNKP/POLD2/RBX1/RFC2/RFC3/MRPS35/RAD18/POLD4/PARP1/RFC4/POLD3/RPA1/POLD1/MRPS26/DNAJA1/RFC5 |
| GOBP | POSITIVE REGULATION OF CHROMOSOME SEGREGATION | 13 | 0.665556321 | 1.724382784 | 0.006063272 | 0.031165313 | 0.021769475 | 3567 | tags=69%, list=17%, signal=57% | CCNB1/AURKB/CDC6/CDT1/RCC2/MAD2L1BP/RAD18/SMC6/BECN1 |
| GOMF | ANTIGEN BINDING | 67 | 0.436455757 | 1.622372361 | 0.006012201 | 0.030926469 | 0.021602638 | 5325 | tags=46%, list=26%, signal=35% | TAP1/SLC7A5/TAP2/FCN1/CD48/IL7R/ITGA4/LAG3/SLAMF1/HLA-E/TAPBP/KLRD1/CD1B/HLA-DRA/PPP2R1A/HLA-F/LILRA2/HLA-B/IGHV7-81/HLA-G/TRDC/CD1D/FCN3/CLEC4M/CD40/HLA-DQB1/HLA-A/CD209/HLA-DPB1/HLA-C/SLC7A8 |
| GOBP | REGULATION OF T CELL APOPTOTIC PROCESS | 33 | 0.524705173 | 1.705727553 | 0.005976401 | 0.030765872 | 0.021490459 | 2825 | tags=48%, list=14%, signal=42% | CD274/WNT5A/PRELID1/PRKCQ/HIF1A/IDO1/JAK3/ZC3H8/IL7R/RIPK3/LGALS9/CD27/DOCK8/ADAM8/FADD/CCL5 |
| GOBP | HEART PROCESS | 277 | -0.277008308 | -1.361542488 | 0.005968622 | 0.030749386 | 0.021478943 | 3508 | tags=25%, list=17%, signal=21% | EDN3/PIK3CA/NEDD4L/NOS1/TAC1/NPR1/PPP1R13L/CALM2/DLG1/CCN2/ATP2A3/AGT/KCND2/CACNA1H/ABCC9/BVES/RYR3/KCNH2/RGS2/CELF2/WWTR1/VEGFB/YAP1/NPR2/ATP2B1/EDN1/ASPH/CACNB2/ACTC1/ADRB1/FXYD1/FGF13/SGCD/HSPB7/DSG2/ANK2/DSP/BIN1/POPDC2/SLC8A1/THRA/AKAP13/ADRA1A/SMAD5/ATP2B4/PLN/CACNA2D2/AKAP9/ITPR3/SCN3B/TRPC1/TNNC1/ATP1A2/FYN/DES/TNNI2/CASQ2/GPD1L/CACNA2D1/DMD/TPM1/FXYD6/GSN/CAV1/SGCG/SOD1/CYP2J2/MEF2A |
| GOBP | SPINAL CORD MOTOR NEURON CELL FATE SPECIFICATION | 10 | -0.703165927 | -1.761110434 | 0.005947575 | 0.030664472 | 0.02141963 | 1373 | tags=30%, list=7%, signal=28% | HOXC10/GLI2/GLI3 |
| GOBP | REGULATION OF ANIMAL ORGAN FORMATION | 25 | -0.537434793 | -1.735355963 | 0.005899805 | 0.030441543 | 0.02126391 | 3925 | tags=48%, list=19%, signal=39% | WNT11/BMP2/FGF1/BMP4/ROBO2/DKK1/HOXA11/WNT2/BMP7/SPRY1/AR/WNT2B |
| GOBP | GLOMERULAR EPITHELIUM DEVELOPMENT | 24 | -0.535081356 | -1.694606609 | 0.005808601 | 0.029993991 | 0.020951288 | 3538 | tags=62%, list=17%, signal=52% | FOXC2/BMP4/JAG1/ASXL1/PECAM1/ADIPOQ/FOXC1/PODXL/LAMB2/IQGAP1/PTPRO/CD34/KLF15/MAGI2/NOTCH2 |
| GOBP | REGULATION OF ALTERNATIVE MRNA SPLICING VIA SPLICEOSOME | 56 | -0.416416612 | -1.611368183 | 0.005773784 | 0.029837139 | 0.020841724 | 5120 | tags=45%, list=25%, signal=34% | THRAP3/CELF6/RBM24/CELF1/KHDRBS3/RBM5/CELF5/RBM25/RBM15B/RBM11/RBM4/CELF2/SRSF6/NSRP1/MBNL2/DYRK1A/DDX17/MBNL1/FAM172A/HNRNPA1/RBFOX2/RBM8A/FXR1/NOVA1/YTHDC1 |
| GOBP | NEUROTROPHIN SIGNALING PATHWAY | 38 | -0.461981088 | -1.651689741 | 0.0057686 | 0.029833296 | 0.020839039 | 3623 | tags=37%, list=17%, signal=31% | SOS1/RAP1A/AGT/NTF4/NDN/NTF3/NTRK2/KIDINS220/ZDHHC17/RAPGEF2/TMEM108/MAGI2/SPRY1/SPRY2 |
| GOCC | CORNIFIED ENVELOPE | 38 | 0.490783505 | 1.643871733 | 0.005764335 | 0.029833296 | 0.020839039 | 763 | tags=29%, list=4%, signal=28% | DSC2/PI3/DSG3/TGM1/SPRR1B/SPRR3/CNFN/SPRR1A/IVL/CSTA/SPRR2G |
| GOBP | DEOXYRIBONUCLEOSIDE TRIPHOSPHATE METABOLIC PROCESS | 15 | 0.644876768 | 1.744287565 | 0.005755906 | 0.029813552 | 0.020825248 | 1988 | tags=53%, list=10%, signal=48% | NUDT15/TYMS/CMPK2/DTYMK/ITPA/ADK/DCTPP1/NUDT1 |
| GOBP | LYMPHOCYTE APOPTOTIC PROCESS | 70 | 0.427371291 | 1.597170431 | 0.005752747 | 0.029813552 | 0.020825248 | 3006 | tags=37%, list=14%, signal=32% | CD274/WNT5A/PRELID1/TNFRSF21/PRKCQ/AURKB/BAK1/HIF1A/IDO1/JAK3/LYN/ZC3H8/IL7R/RIPK3/LGALS9/CD27/DOCK8/NOC2L/CD3G/IL2RA/ADAM8/FADD/CCL5/DNAJA3/FASLG/BAX |
| GOBP | REGULATION OF BLOOD PRESSURE | 171 | -0.300970535 | -1.405857495 | 0.005726314 | 0.029706083 | 0.020750179 | 4002 | tags=29%, list=19%, signal=23% | RARRES2/LRP5/RAMP2/EDN3/HSD11B2/TRHDE/NOS1/SCPEP1/VEGFC/TAC1/NPR1/AGT/CTSG/LVRN/ECE1/NPR2/LEP/ATP2B1/EDN1/ADIPOQ/F2R/ADRB1/PPARG/ACVRL1/MECP2/ARHGAP42/GAS6/NISCH/NR2F2/PPARA/P2RX1/ADRA1A/CAMK2N1/NPY1R/PTPRO/SMAD3/KLK1/CD34/ACTA2/ATP1A2/CORO2B/AGTR1/LNPEP/AR/TPM1/POSTN/SOD1/DDAH1/ADRB2 |
| GOBP | TAIL ANCHORED MEMBRANE PROTEIN INSERTION INTO ER MEMBRANE | 16 | 0.641546588 | 1.776505887 | 0.005700191 | 0.029593418 | 0.020671481 | 5028 | tags=69%, list=24%, signal=52% | UBL4A/EMC6/EMC3/EMC8/EMC9/GET3/EMC1/EMC7/SGTA/EMC4/EMC10 |
| GOBP | LUTEINIZATION | 10 | -0.706016355 | -1.768249457 | 0.005694491 | 0.029586689 | 0.020666781 | 729 | tags=40%, list=3%, signal=39% | FZD4/STAT5B/PLEKHA1/PDGFRA |
| GOBP | MITOTIC CHROMOSOME CONDENSATION | 15 | 0.646263761 | 1.748039156 | 0.005690125 | 0.029586689 | 0.020666781 | 3800 | tags=53%, list=18%, signal=44% | NUSAP1/CDCA5/NCAPH/NCAPG/SMC2/SMC4/NCAPD2/CHMP1A |
| GOBP | CELLULAR RESPONSE TO RADIATION | 183 | 0.336462985 | 1.448819207 | 0.005682579 | 0.029570537 | 0.020655498 | 4910 | tags=38%, list=24%, signal=30% | IFI16/H2AX/PBK/AURKB/PCNA/RUVBL2/BAK1/NMT1/MAPK13/HUS1/RAD51/METAP1/EIF2S1/MMP9/HSPA5/ECT2/CDC25A/COPS9/ZMPSTE24/RAD51AP1/MYC/FIGNL1/MMP1/CDKN1A/XRCC6/BLM/ST20/XRCC5/SMPD1/METAP2/NOC2L/HRAS/CARD16/NPM1/CHEK2/ATF4/TRIAP1/INTS7/LIG4/SWI5/NIPBL/FBXO4/TMEM161A/BAX/ERCC1/GADD45A/PARP1/MDM2/PRKCD/OPN3/POLD3/KDM1A/MAPK14/ELK1/ATR/HYAL3/TLK2/POLD1/MMP3/TRPM1/NEDD4/DNM2/TANK/CASP9/USP28/GRB2/GRK4/RHNO1/OPN5/FNTA |
| GOBP | BIOMINERALIZATION | 149 | -0.315195444 | -1.433749096 | 0.005672125 | 0.029539021 | 0.020633484 | 4004 | tags=33%, list=19%, signal=27% | RFLNB/ACVR2B/BMP2/BMP6/ATRAID/LGR4/FGFR3/FOXO1/BMP4/ADGRV1/PTH1R/TFAP2A/ENPP1/LOX/DDR2/ZBTB40/LEP/ATP2B1/ANO6/FBLN7/TWIST1/OMD/KLF10/P2RX7/GPM6B/FGFR2/GREM1/MGP/GAS6/BMPR1A/PPARA/ROR2/GPC3/PTN/MEF2C/SLC8A1/ROCK2/BMPR1B/SMAD3/WDR72/BMP7/ASPN/OSR1/NBR1/ANKH/TUFT1/OSR2/AXIN2/ADRB2 |
| GOMF | OXIDOREDUCTASE ACTIVITY ACTING ON CH OH GROUP OF DONORS | 124 | 0.369003518 | 1.524339541 | 0.005655472 | 0.029475142 | 0.020588863 | 3099 | tags=29%, list=15%, signal=25% | AKR1B10/IDH3A/BDH1/GFUS/PGD/RDH10/DHRS9/SDR9C7/MDH2/IMPDH1/LDHA/CBR3/RDH16/RDH12/ADH7/PTGR1/DHRS13/HSD17B10/HSD17B2/IDH2/IDH3B/HSD17B4/CBR1/ALDH3A1/VKORC1/MDH1/SDR16C5/NSDHL/PHGDH/L2HGDH/ME1/KCNAB2/SDR42E1/CRYL1/G6PD/HMGCR |
| GOBP | REGULATION OF CELL SHAPE | 147 | -0.313200677 | -1.419005454 | 0.0056338 | 0.029384991 | 0.020525891 | 2596 | tags=28%, list=12%, signal=25% | BVES/SEMA3E/S100A13/CFAP410/RHOBTB1/RHOQ/WASF3/PARVA/PALM2AKAP2/PTK2/ARHGAP35/ITGA7/PHIP/RHOB/FN1/SEPTIN7/EPB41/PTN/SH3KBP1/RHOJ/DAG1/CFDP1/FERMT2/SYNE3/MYH14/WDPCP/MKLN1/PLXNA2/PALMD/LPAR1/FYN/FGD4/EPS8/PALM/MYH10/FGD5/DLC1/TPM1/KIT/DNMBP/RHOBTB3 |
| GOBP | CELLULAR RESPONSE TO CALCIUM ION | 77 | -0.379584882 | -1.565819024 | 0.005628972 | 0.029382623 | 0.020524237 | 3727 | tags=36%, list=18%, signal=30% | DMTN/CPNE2/CAPN3/SYT1/CLIC4/ADGRV1/RASA4/CPNE8/RYR3/EDN1/NLGN1/FOS/CPNE3/ADD1/JUND/CACYBP/AKR1C3/ITPKB/PRKAA2/PKD2/MEF2C/SYT17/IQGAP1/BRAF/SYT8/MEF2A/EEF2K/CHP2 |
| GOBP | PEPTIDE CROSS LINKING | 34 | 0.510219447 | 1.674954253 | 0.005610454 | 0.029308732 | 0.020472623 | 1088 | tags=26%, list=5%, signal=25% | PI3/TGM1/SPRR1B/SPRR3/SPRR1A/IVL/TGM3/CSTA/SPOCK2 |
| GOBP | REGULATION OF VIRAL TRANSCRIPTION | 42 | 0.490513874 | 1.674316175 | 0.005596287 | 0.029257474 | 0.020436818 | 3714 | tags=45%, list=18%, signal=37% | TRIM14/POLR2H/POLR2F/TARBP2/NELFCD/TRIM62/TRIM21/IFITM3/POLR2L/ZFP36/POLR2G/POLR2D/POLR2E/UBP1/NELFE/CDK9/POLR2I/TRIM11/SUPT4H1 |
| GOMF | ADP BINDING | 38 | 0.492587711 | 1.649914894 | 0.005525401 | 0.028909382 | 0.020193671 | 4407 | tags=61%, list=21%, signal=48% | PGK1/RUVBL1/VCP/RUVBL2/TAP1/ABCG1/P2RY1/CHORDC1/ME1/LONP1/PKM/ATP1A1/MYH9/ATP5F1D/MIEF1/ATP5F1A/PRKAG1/GCLC/PGK2/PRPS2/MYO6/PPP5C/MYO7A |
| GOBP | POSITIVE REGULATION OF PROTEIN LOCALIZATION TO NUCLEUS | 85 | 0.40049896 | 1.550677506 | 0.005524726 | 0.028909382 | 0.020193671 | 3991 | tags=42%, list=19%, signal=34% | ZC3H12A/RAN/PARP9/CDK1/CCT5/DTX3L/CCT2/ZPR1/CCT3/ECT2/CCT7/IFNG/LAMTOR5/PINX1/BAG3/PLK1/LIMK2/SESN2/TFRC/TARDBP/TCP1/PARK7/PIK3R2/DKC1/HCLS1/CCT6A/CCT8/PARP1/MCRS1/PRKCD/NUTF2/CARD10/HDAC3/CDH1/JUP/MAPK14 |
| GOBP | IMP METABOLIC PROCESS | 14 | 0.659218427 | 1.741708339 | 0.005502555 | 0.028834764 | 0.020141549 | 1143 | tags=43%, list=5%, signal=41% | GART/ATIC/NT5C2/HPRT1/AMPD3/ADSL |
| GOBP | REGULATION OF EPIDERMIS DEVELOPMENT | 66 | 0.429552477 | 1.599362695 | 0.00546466 | 0.028658539 | 0.020018453 | 2810 | tags=35%, list=13%, signal=30% | SERPINB13/PPARD/MACROH2A1/PRKCH/EZH2/TMEM79/TRIM16/ZBED2/IL20/SFN/CTSL/CYP27B1/CBFB/SULT2B1/OVOL2/ZFP36/ETV4/GRHL1/TP63/AQP3/MACROH2A2/PLAAT4/KEAP1 |
| GOBP | T CELL CYTOKINE PRODUCTION | 36 | 0.501045614 | 1.661629414 | 0.005456867 | 0.028640023 | 0.020005519 | 5586 | tags=61%, list=27%, signal=45% | FZD5/RSAD2/ARG1/SASH3/IL1B/IL6/LILRB4/TNFRSF1B/XCL1/DENND1B/CCR2/B2M/HLA-F/NLRP3/FOXP3/TRAF2/MALT1/TBX21/IFNA2/HLA-A/IL1R1/CLC |
| GOBP | INNER CELL MASS CELL PROLIFERATION | 12 | 0.686010462 | 1.738980562 | 0.005436657 | 0.028556266 | 0.019947013 | 6361 | tags=92%, list=30%, signal=64% | GINS1/ZPR1/NCAPG2/COPS2/BRCA2/NDEL1/PRPF19/PELO/GINS4/TAF8/SALL4 |
| GOBP | REGULATION OF MAST CELL ACTIVATION | 41 | 0.49223732 | 1.67555002 | 0.005436081 | 0.028556266 | 0.019947013 | 4232 | tags=49%, list=20%, signal=39% | IL4R/PLA2G3/PLSCR1/LYN/HMOX1/SYK/RAC2/NR4A3/LGALS9/VAMP8/C12orf4/VAMP7/TSLP/ADGRE2/UNC13D/NECTIN2/CD300A/FGR/CRLF2/CD300LF |
| GOBP | UREA METABOLIC PROCESS | 13 | 0.672251176 | 1.741728414 | 0.00540779 | 0.028449127 | 0.019872175 | 1618 | tags=38%, list=8%, signal=36% | NMRAL1/ARG1/ASL/SLC25A15/NAGS |
| GOBP | FORELIMB MORPHOGENESIS | 34 | -0.502373305 | -1.752161337 | 0.005350593 | 0.028170285 | 0.0196774 | 3223 | tags=50%, list=15%, signal=42% | HOXD9/ATRX/TFAP2A/ZNF358/TBX3/SHOX2/RECK/EN1/FMN1/TWIST1/LNPK/HOXA11/ALDH1A2/TFAP2B/OSR1/ZBTB16/OSR2 |
| GOBP | NEGATIVE REGULATION OF CELL KILLING | 25 | 0.565768967 | 1.704360106 | 0.005341611 | 0.028145053 | 0.019659774 | 5020 | tags=56%, list=24%, signal=43% | SERPINB4/KRT6A/SERPINB9/IL7R/LGALS9/HLA-E/KLRD1/HLA-F/HLA-B/LILRB1/HLA-G/PTPRC/ARRB2/HLA-A |
| GOBP | MULTICELLULAR ORGANISMAL SIGNALING | 195 | -0.282334836 | -1.325806361 | 0.00531938 | 0.028049917 | 0.01959332 | 5098 | tags=31%, list=24%, signal=23% | FGF12/ATP1B2/GRIK2/ITPR2/KCNIP2/HRC/CAMK2D/CASQ1/ITPR1/SCN1B/S1PR1/TMEM65/AVPR1A/TNNI3K/CACNA2D4/SCN3A/GPER1/NFASC/SCN9A/NOS1/NPR1/CALM2/ATP2A3/AGT/KCND2/CACNA1H/ABCC9/RYR3/KCNH2/NPR2/ATP2B1/ASPH/CACNB2/FXYD1/DSG2/ANK2/DSP/BIN1/NTRK2/SLC8A1/P2RX1/SCN7A/MYH14/ATP2B4/PLN/CACNA2D2/AKAP9/ITPR3/SCN3B/TRPC1/PAFAH1B1/ATP1A2/CASQ2/CACNA2D1/JAM3/FXYD6/CAV1/SOD1/MEF2A/KCNMB4 |
| GOCC | COATED VESICLE | 287 | 0.308684546 | 1.394143908 | 0.00531007 | 0.028022821 | 0.019574394 | 3861 | tags=29%, list=19%, signal=24% | WNT5A/FZD5/CTSC/AP2S1/SERPINA1/LDLR/TGFA/DNAJC5/CNIH4/EPN3/RAB27A/AREG/COPB2/HBEGF/PCSK9/SCYL2/FCGR1B/MYO1E/MALL/STX6/LMAN2/BCAP31/YIF1A/YIF1B/DBNL/RAB5A/CD3D/CNIH1/HAX1/HSPA8/ACTR1A/SEC23B/AP1M2/PDCD6/IL7R/KDELR2/AP3B1/SEC24C/TMED5/CD3G/EREG/TFRC/VAMP8/ADAM10/COPE/SEC13/RGS19/HLA-E/VAMP7/VPS33B/STON2/FURIN/HSPD1/SFTPD/SREBF2/CLINT1/AP1B1/CLTA/AP1G2/WIPI1/SNX3/LDLRAP1/TYRP1/HLA-DRA/LMAN1/HIP1R/TMEM199/DENND1B/M6PR/TMED7/RAB8A/B2M/VPS16/HLA-F/GAD1/HLA-B/CEMIP/UNC13D/SREBF1/EPN1/HLA-G/TMED9 |
| GOMF | ORGANIC ACID TRANSMEMBRANE TRANSPORTER ACTIVITY | 146 | 0.35894235 | 1.505663942 | 0.005302033 | 0.028002403 | 0.019560131 | 5325 | tags=39%, list=26%, signal=29% | SLC6A14/SLC26A9/SLC7A1/SLC7A11/SFXN1/ABCC1/SLC16A1/SLC23A2/SLC16A6/SLC16A10/SLC7A5/SLC25A13/SLC25A15/SLC5A6/SLC38A5/SLC25A10/SLC36A1/SLC25A44/SFXN2/SLC6A15/SLC3A2/CD36/CTNS/SLC16A9/SLC25A11/SLC9A3R1/SLC1A3/SLC26A4/SLC51A/SLC19A1/SLC16A3/SFXN5/SLC38A9/SLC66A1/SLC38A7/SLC6A20/SLC23A1/SLC7A7/SLC36A4/SLC6A1/SLC25A32/SLC43A2/SLC1A1/SLC1A5/SLC1A4/SLC10A3/SLC25A12/SLC6A19/SLC6A11/SLC16A14/SLC15A4/SLC6A6/SLC29A4/MPC2/SLCO2B1/SLC38A6/SLC7A8 |
| GOBP | VASCULAR ENDOTHELIAL GROWTH FACTOR SIGNALING PATHWAY | 40 | -0.475016284 | -1.723202397 | 0.005297856 | 0.028002359 | 0.019560101 | 4633 | tags=55%, list=22%, signal=43% | MYO1C/DAB2IP/KDR/PRKD2/PIK3CA/NRP1/VEGFC/JCAD/SMOC2/PDGFRB/PRKD1/VEGFB/PGF/SEMA6A/CD63/ADGRA2/FOXC1/PIK3CB/CCBE1/DCN/PDGFRA/SPRY2 |
| GOBP | MECHANORECEPTOR DIFFERENTIATION | 60 | -0.405393046 | -1.576023294 | 0.00528332 | 0.027947513 | 0.019521789 | 5630 | tags=43%, list=27%, signal=32% | CECR2/IFT88/TPRN/DLL1/TRIP11/CTHRC1/FAT4/PJVK/IFT27/TRIOBP/BMP4/ADGRV1/HES1/JAG1/NTF4/TSKU/JAG2/SEC24B/NTRK2/MYCL/RBPJ/SDC4/WDPCP/MCOLN3/SLITRK6/SOD1 |
| GOMF | CGMP BINDING | 14 | -0.649306434 | -1.794784608 | 0.005260701 | 0.027849796 | 0.019453533 | 3219 | tags=36%, list=15%, signal=30% | PDE5A/PRKG2/PRKG1/PDE2A/CNGA1 |
| GOBP | MUSCLE ADAPTATION | 99 | -0.347482699 | -1.498057338 | 0.005245319 | 0.027790263 | 0.019411948 | 3291 | tags=32%, list=16%, signal=27% | CAMTA2/PDE9A/FOXO1/JARID2/AGT/RGS2/EDN1/IL6ST/PRKCA/ACTA1/FOXO3/SGCA/CAMK2G/KLF4/PPARA/FBXO32/PPARGC1A/IGFBP5/DAG1/LMCD1/ROCK2/ADRA1A/SMAD3/ATP2B4/TNNT1/MYOC/KLF15/TNNC1/GSN/GATM/MEF2A/UTRN |
| GOBP | REGULATION OF PROTEIN SERINE THREONINE KINASE ACTIVITY | 466 | 0.278891237 | 1.308220841 | 0.005203066 | 0.02758816 | 0.019270776 | 3699 | tags=27%, list=18%, signal=23% | SERPINB3/S100A12/CD24/CCNB1/WNT5A/NOD2/LTF/CDK1/CCNB2/CXCR4/FZD5/PBK/UBE2N/CKS2/CDKN3/PYCARD/SERTAD1/MACROH2A1/CCNE1/IRAK2/TGFA/GSTP1/EZH2/PPP2CA/CDC6/CCNA2/CCNE2/PPIA/CXCL10/FPR1/IRAK1/MLKL/RALB/SFN/ADAM17/CDC25A/EPHB2/DBF4/TRIB2/MAPKAPK3/CCNC/DUSP2/RGS14/DUSP14/IFNG/MAP3K9/CCNF/DBNL/GTPBP4/IL1B/LYN/PDCD10/CSK/CBLC/CDKN1A/ADORA2B/SHC1/PSRC1/PLK1/RASGRP1/PIH1D1/SYK/BCCIP/CKS1B/BLM/SESN2/RGS3/CCL19/PTPN22/DUSP7/CXCL17/CIB1/RASIP1/DUSP5/PKMYT1/SMPD1/CCND2/HRAS/TRAF7/IKBKG/HERC5/MAP2K3/FAM20A/PRKAR2A/ADAM8/TPD52L1/CAB39/CCNO/MAP2K6/MAP2K4/CCNQ/DUSP18/SYAP1/CDK7/CHORDC1/MEN1/TNF/MAP3K5/CDC37/CDKN2A/GPS1/CDC25C/RB1/CCNH/FGD2/GADD45A/MLST8/PSMD10/LPAR3/DUSP9/CASP3/PKIA/IRAK3/ARRB1/PRKCD/PRKAG1/PPP2R1A/TELO2/DAXX/MAPKAPK2/HTT/CCND3/CDKN2D/CDK4/CIT/CEMIP |
| GOBP | MAST CELL ACTIVATION | 60 | 0.433741394 | 1.587575705 | 0.005172345 | 0.027446929 | 0.019172123 | 4432 | tags=43%, list=21%, signal=34% | S100A12/IL4R/PLA2G3/PLSCR1/RHOH/LYN/HMOX1/SYK/RAC2/NR4A3/LGALS9/VAMP8/PIK3CD/LCP2/C12orf4/VAMP7/TSLP/LAT2/ADGRE2/UNC13D/NECTIN2/CD300A/FGR/CRLF2/CD300LF/LAT |
| GOMF | HYDROLASE ACTIVITY ACTING ON CARBON NITROGEN BUT NOT PEPTIDE BONDS | 113 | 0.372770637 | 1.515550173 | 0.005161974 | 0.027413551 | 0.019148808 | 5884 | tags=44%, list=28%, signal=32% | GDA/GCH1/VNN3/HDAC1/APOBEC3B/PGLYRP4/VNN1/ATIC/MTHFD1/MTHFD2/ARG1/AMPD3/FAAH2/SIRT7/RIDA/NIT2/ADAR/PGLYRP2/NADSYN1/ASPG/ADA2/NGLY1/HDAC2/PGLYRP3/SIRT6/PIGL/DCTD/NTAQ1/OPLAH/NIT1/HDAC3/CAD/ACER3/AGA/ADAT3/ASAH2B/VNN2/HINT1/APOBEC3C/MTHFD2L/HDAC8/CD101/ACY1/APOBEC3G/ADAT2/DARS1/ADAT1/SIRT2/ARG2/MTA2 |
| GOBP | REGULATION OF RESPONSE TO INTERFERON GAMMA | 24 | 0.571971928 | 1.723487141 | 0.005158014 | 0.027413551 | 0.019148808 | 2915 | tags=50%, list=14%, signal=43% | STAT1/PARP9/SOCS3/PTPN2/IFNGR1/PARP14/ARG1/IFNG/SOCS1/NLRC5/CDC37/IFNGR2 |
| GOBP | CHROMATIN ASSEMBLY OR DISASSEMBLY | 193 | 0.325941841 | 1.411709452 | 0.005121338 | 0.027240813 | 0.019028148 | 5042 | tags=38%, list=24%, signal=29% | RUVBL1/H2AX/CENPN/HDAC1/MACROH2A1/KNL1/CENPW/CENPX/MCM2/ASF1B/OIP5/HJURP/CENPM/DNMT1/CENPK/CENPA/IPO4/POLE3/CENPS/HAT1/SUV39H2/SMARCD3/RRP8/NASP/NOC2L/NPM1/GRWD1/CENPH/CENPI/NAP1L4/HMGB2/H2BC9/MACROH2A2/H3C10/HELLS/NRDE2/PAF1/MBD2/CDKN2A/MIS18A/SMARCA4/SIRT6/SMARCA5/HMGA1/BEND3/SPTY2D1/DAXX/CENPL/CHAF1A/NAA60/CENPO/PARP10/DNMT3A/H2BC10/CENPU/H1-2/RESF1/CHAF1B/TLK2/H3C7/ZNFX1/H2BC12/H3C8/H4C4/H4C9/H2BC13/SUV39H1/MBD3/HDAC8/H1-5/RBBP4/H2BC6/H4C2 |
| GOBP | CHAPERONE MEDIATED PROTEIN FOLDING | 58 | 0.444007376 | 1.610105928 | 0.005103268 | 0.027166207 | 0.018976035 | 4203 | tags=50%, list=20%, signal=40% | DNAJC5/HSPA5/CCT2/ERO1A/HSPA14/HSPA9/TRAP1/HSPE1/HSPA8/PDCD5/HSPH1/CSNK2A1/TOR1A/PDIA4/DNAJB1/FKBP1B/SDF2L1/DNAJC7/TOR2A/CHORDC1/UNC45A/PPIB/PTGES3/FKBP4/TOR1B/DNAJB5/SGTA/DNAJB12/SDF2 |
| GOBP | METANEPHRIC COLLECTING DUCT DEVELOPMENT | 11 | -0.697527851 | -1.785037182 | 0.005100938 | 0.027166207 | 0.018976035 | 3069 | tags=45%, list=15%, signal=39% | BMP4/WNT7B/DLG5/PTCH1/PKD1 |
| GOMF | GLYCOSAMINOGLYCAN BINDING | 219 | -0.290078171 | -1.386801886 | 0.005089617 | 0.027136545 | 0.018955315 | 3511 | tags=28%, list=17%, signal=23% | FSTL1/NRP1/ADAMTS5/LGR6/APLP2/FGF1/APP/BMP4/CCN2/SMOC2/TENM1/GNS/CTSG/THBS2/RSPO3/THBS3/RTN4R/VEGFB/FGFBP3/SLIT2/CCN3/LXN/PGF/SOD3/FBLN7/FGF9/PCOLCE2/SERPINA5/SLIT3/LTBP4/APOE/PTPRS/FGFR2/LPL/TNXB/FN1/FGFR1/PTPRF/PTN/ECM2/PRELP/CCN5/LYVE1/VIT/RPL22/CFH/RSPO1/NDNF/TGFBR2/LAMC2/BMP7/DCN/PTCH1/PAFAH1B1/RTN4RL1/DPYSL3/GPNMB/POSTN/GREM2/TGFBR3/ANG |
| GOBP | MICROGLIAL CELL ACTIVATION | 41 | 0.494180649 | 1.68216501 | 0.005087245 | 0.027136545 | 0.018955315 | 6073 | tags=61%, list=29%, signal=43% | CTSC/LDLR/IFNGR1/TLR2/CST7/IFNG/IL6/ITGB2/GRN/TLR8/TNF/TREM2/TYROBP/SPHK1/PTPRC/IL33/AIF1/AGER/TLR1/C5AR1/FPR2/AZU1/C1QA/TLR7/IL13 |
| GOCC | SARCOPLASM | 76 | -0.391961195 | -1.61279847 | 0.005084078 | 0.027136545 | 0.018955315 | 5198 | tags=47%, list=25%, signal=36% | THBS4/STRIT1/ITPR2/FABP3/RASD1/HRC/GSTM2/CAMK2D/S100A1/CASQ1/ITPR1/TMEM109/AKAP6/SLN/POMT1/RTN2/PLEC/NOS1/SPOCK1/ATP2A3/RYR3/MTMR12/ASPH/JPH2/SYNE2/CAMK2G/CMYA5/MEF2C/HABP4/PLN/ITPR3/FLNC/CASQ2/IRAG1/CACNA2D1/GSN |
| GOBP | REGULATION OF DEFENSE RESPONSE TO VIRUS BY VIRUS | 29 | 0.545586752 | 1.712068676 | 0.005081426 | 0.027136545 | 0.018955315 | 4419 | tags=48%, list=21%, signal=38% | AP2S1/LCK/AP1S3/CD247/AP1M2/HCK/ATP6V1H/SCRIB/AP1B1/B2M/DOCK2/EIF2AK2/AP2M1/CD28 |
| GOMF | NUCLEOSIDE TRIPHOSPHATASE REGULATOR ACTIVITY | 483 | -0.241724757 | -1.264416182 | 0.005078584 | 0.027136545 | 0.018955315 | 4067 | tags=29%, list=19%, signal=24% | RGL1/DENND1A/RUNDC1/ARHGEF17/GRTP1/RASGRF2/RAPGEF4/ARHGEF10L/PREX2/RABGEF1/GNAQ/ALS2/SOS1/ARHGEF2/ALDH1A1/RALGDS/MADD/NRP1/EEF1D/RAP1A/IQSEC1/RABEP1/TIAM2/ASAP3/TBC1D8/BAG5/AGAP9/SRGAP3/ANKRD27/CPEB2/SOS2/DENND3/RASA4/AGAP4/ARHGEF1/STXBP5/TBC1D2B/LARS1/ARHGAP24/SMCR8/LRRK2/ARHGAP10/RASA3/RGS2/CYTH3/HACD3/NGEF/DENND5B/SYDE1/SLIT2/CHML/PLCB1/IPO5/DENND5A/BAG2/ARHGEF15/EVI5/DOCK5/DOCK4/FBXO8/TBC1D15/RAPGEF3/ARHGAP20/ADRB1/RANBP3/ARHGEF6/FARP1/SPATA13/TRIO/EGF/ARHGAP26/RAPGEFL1/RALGAPB/AGFG2/FNIP1/TOR1AIP1/GIT2/SH3BP5/ARHGAP12/ARFGAP3/DNAJC24/ARHGAP35/AGAP1/TBC1D16/ARHGAP42/SH3BP4/ARHGEF5/DENND11/DOCK7/AGAP11/AHSA2P/STARD13/AKAP13/IQGAP1/HERC1/ARHGAP6/SBF2/DOCK1/VAV3/ARHGAP29/SGSM2/ARHGEF12/TBC1D5/DENND2A/RAPGEF2/TBC1D24/PLN/RAB3GAP1/RALGPS1/SRGAP1/DNAJB4/ARHGEF40/TSC1/RALGAPA1/FGD4/ITSN1/RASAL2/FNIP2/SERGEF/GARNL3/ARFGEF3/BNIP2/FGD5/ELMOD1/DLC1/ARHGAP31/FAM13A/MCF2L/ARHGEF7/ARHGEF28/ASAP2/DNMBP/MYO9A/SRGAP2/ARHGEF10/DNAJB2/BCAR3/DENND4C/RABGAP1L/LAMTOR4 |
| GOBP | NUCLEUS ORGANIZATION | 121 | 0.363930991 | 1.497609123 | 0.005050243 | 0.02703389 | 0.018883609 | 4021 | tags=36%, list=19%, signal=30% | CCNB1/CDK1/CCNB2/PSME4/PPP2CA/ZPR1/ZMPSTE24/PLK1/PPP2R2A/NOLC1/EMG1/FAM118B/TOR1A/SRPK1/REEP4/SERBP1/VRK1/PITPNB/POLR1B/TARDBP/HMGB2/NDEL1/NSFL1C/HIPK2/NUP155/LEMD2/PML/CHMP5/CHMP1B/NDC1/NEK6/RNF8/CHMP6/ANKLE2/PPP2R1A/CNEP1R1/NUP205/TOR1B/NECTIN2/CHMP1A/EMD/CHMP2A/NUP107/NUP93 |
| GOCC | KINESIN COMPLEX | 49 | 0.466348902 | 1.659338713 | 0.005042477 | 0.027013842 | 0.018869606 | 2964 | tags=35%, list=14%, signal=30% | KIF20A/KIF2C/KIF18B/KIF2A/KIF11/KIF14/BORCS5/KIF23/KIF15/KIF18A/KIF20B/NDE1/KIF22/NDEL1/KIFC1/KIF1B/KIF21B |
| GOBP | CELLULAR RESPONSE TO ABIOTIC STIMULUS | 319 | 0.310001281 | 1.413396631 | 0.005029512 | 0.026965891 | 0.018836111 | 4951 | tags=35%, list=24%, signal=27% | GPR68/IFI16/H2AX/PBK/MYD88/GOT1/AURKB/PTAFR/PCNA/RUVBL2/CASP1/BAK1/NMT1/MAPK13/HUS1/RAD51/METAP1/EIF2S1/MMP9/HSPA5/IRF1/ECT2/CDC25A/COPS9/ZMPSTE24/RAD51AP1/CASP5/LRRC8D/SLC2A1/NFKB1/RELB/IL1B/MYC/BAG3/FIGNL1/MMP1/CDKN1A/XRCC6/CNN2/BLM/ST20/XRCC5/SMPD1/METAP2/TNFRSF10A/NOC2L/HRAS/OXSR1/CARD16/KCNJ2/NPM1/CASP8/CHEK2/TNFRSF10B/FADD/BDKRB2/BNIP3/ATF4/LTBR/CAB39/MAP2K4/TRIAP1/INTS7/LIG4/TNFRSF1A/SWI5/NIPBL/TLR8/FBXO4/CRADD/TMEM161A/ATP1A1/BAX/ERCC1/GADD45A/PARP1/MDM2/TSPO/CASP3/PRKCD/BCL10/OPN3/POLD3/KDM1A/MAPK8/ERRFI1/GJA1/MAPK14/ELK1/ATR/HYAL3/EPO/TLK2/POLD1/MMP3/TRPM1/NEDD4/DNM2/TANK/TNFSF14/CASP9/USP28/CD40/PIEZO1/MAPK3/GRB2/WNK3/GRK4/RHNO1/OPN5/FNTA/CLN3 |
| GOBP | REGULATION OF CELLULAR RESPONSE TO TRANSFORMING GROWTH FACTOR BETA STIMULUS | 120 | -0.331471155 | -1.460783161 | 0.004990534 | 0.026778279 | 0.018705061 | 5769 | tags=46%, list=28%, signal=33% | HSP90AB1/PBLD/SMAD2/RNF111/CIDEA/FBN1/FLCN/EP300/NRROS/SMAD4/STUB1/LATS1/BAMBI/LEMD3/RASL11B/PEG10/PDPK1/HTRA1/UBC/SMURF1/LDLRAD4/SLC2A10/HTRA4/SIRT1/SPRED2/DAB2/LOX/GLG1/SNW1/CD109/SPRED1/TRIM33/LATS2/SNX25/CREBBP/DKK3/LTBP4/SMURF2/PMEPA1/PPARA/SDCBP/MYOCD/SKI/SMAD3/TGFB1I1/TGFBR2/ASPN/CDKN1C/IL17RD/SPRY1/ZNF703/SPRY2/CAV2/CAV1/TGFBR3 |
| GOBP | EAR MORPHOGENESIS | 106 | -0.345465427 | -1.499761601 | 0.004976314 | 0.026723322 | 0.018666672 | 2499 | tags=25%, list=12%, signal=22% | EDN1/NOG/DVL2/FZD3/TWIST1/FGFR2/SEC24B/SOBP/ROR2/FRZB/NTN1/TSHZ1/LRIG1/MAPK1/LRIG3/WDPCP/GATA2/OSR1/RPL38/WDR19/SLITRK6/GLI2/SPRY2/OSR2/SOD1/GATA3 |
| GOBP | SPINDLE MIDZONE ASSEMBLY | 11 | 0.702073263 | 1.742303598 | 0.004970515 | 0.026713533 | 0.018659835 | 1004 | tags=45%, list=5%, signal=43% | AURKB/PRC1/KIF4A/KIF23/RACGAP1 |
| GOMF | ALANINE TRANSMEMBRANE TRANSPORTER ACTIVITY | 10 | 0.721869282 | 1.743249982 | 0.004966534 | 0.026713511 | 0.018659819 | 5325 | tags=90%, list=26%, signal=67% | SLC6A14/SLC38A5/SLC36A1/SLC3A2/SLC38A7/SLC36A4/SLC1A4/SLC6A6/SLC7A8 |
| GOMF | PEPTIDASE REGULATOR ACTIVITY | 216 | 0.326640472 | 1.43421854 | 0.004952651 | 0.026660182 | 0.018622568 | 2538 | tags=21%, list=12%, signal=19% | SERPINB3/PI3/SERPINB4/PSME2/SERPINB13/PTTG1/LTF/SERPINB1/A2ML1/BIRC5/CTSC/SERPINA1/CSTB/PYCARD/PSME4/SLPI/PSME1/VCP/CASP1/CLPX/PRSS22/PI15/CSTA/CST7/PSENEN/PSMD14/GAPDH/SPOCK2/SERPINA3/UCHL5/WFDC12/SERPINB9/RPS6KA1/SERPINB8/SPINT2/BIRC3/ST20/CD27/PSMF1/ADRM1/PSME3/BEX3/CARD16/PRDX5/USP14/FURIN |
| GOBP | SPINDLE ASSEMBLY | 110 | 0.370385903 | 1.496507536 | 0.004939858 | 0.026612642 | 0.018589361 | 3974 | tags=35%, list=19%, signal=28% | CDC20/CCNB2/AURKB/NEK2/PRC1/KIF4A/AURKA/TPX2/SPAG5/RAB11A/KIF2A/KIF11/KIF23/RACGAP1/ZNF207/MYBL2/HAUS1/PLK1/BCCIP/RCC1/FBXO5/CHEK2/KPNB1/GPSM2/KIFC1/MAPRE1/WASHC5/DRG1/CHMP5/CHMP1B/NEK6/SAC3D1/CCDC69/HDAC3/CHMP1A/CHMP2A/WRAP73/TUBGCP2 |
| GOBP | ODONTOGENESIS | 120 | -0.331970069 | -1.462981859 | 0.004931475 | 0.026588803 | 0.018572709 | 4764 | tags=34%, list=23%, signal=27% | PERP/WNT6/ATF2/HTRA1/MSX1/TGFB2/BMP2/PITX2/AQP5/EDA/ADAMTS5/FOXO1/EDAR/BMP4/TFAP2A/BCL11B/EDN1/JAG2/TWIST1/CTNNA1/ZNF22/LRP6/FOXC1/PAM/FGFR2/BMPR1A/PPARA/CD34/BMP7/ASPN/OSR1/AQP1/LAMA5/PDGFRA/GLI2/FST/LRP4/GLI3/TUFT1/OSR2/AXIN2 |
| GOCC | LAMELLIPODIUM | 192 | -0.304774481 | -1.429055774 | 0.00491606 | 0.026526978 | 0.018529523 | 3835 | tags=34%, list=18%, signal=28% | INPPL1/APC/ILK/CSPG4/ITGAV/PIK3CA/ALS2/RDX/STX2/PLXND1/TIAM2/PKN2/PXN/APP/PARVB/KITLG/WASF2/TESC/ABLIM1/AMOT/SWAP70/PTPRM/ANTXR1/SH3RF1/RAPGEF3/ACTC1/NEDD9/ARHGEF6/NHS/WASF3/SPATA13/PARVA/ACTA1/CTNNA1/FER/SYNE2/PLEKHH2/PODXL/ITGB1/PLEKHG5/SORBS2/PKD2/APBB2/DAG1/PTPRO/FERMT2/ACTA2/ABI2/MYLK/CDC42BPA/GDPD2/ABLIM3/TSC1/FGD4/ACTG2/MYH10/ITSN1/FGD5/ARHGAP31/DBN1/DPYSL3/MCC/ARHGEF7/PDLIM4/GSN/SRGAP2 |
| GOBP | NEURAL PRECURSOR CELL PROLIFERATION | 134 | -0.330452019 | -1.484716136 | 0.004907737 | 0.026503356 | 0.018513023 | 3434 | tags=28%, list=16%, signal=23% | HOOK3/PTBP2/VEGFC/CEP120/NAP1L1/SOX10/LRRK2/FZD3/EML1/ORC3/FGF13/EGF/CTNNA1/RYK/FGFR2/POU3F3/LIMS2/PTN/DOCK7/ELL3/PCM1/WNT2/SLC16A2/NUMBL/RERE/GATA2/PAFAH1B1/EPHB1/DIXDC1/ZNF423/CDON/DBN1/GLI2/GLI3/SOX5/EMX2/RORA |
| GOBP | POSITIVE REGULATION OF OSTEOBLAST DIFFERENTIATION | 59 | -0.411499852 | -1.602747027 | 0.0049051 | 0.026503356 | 0.018513023 | 4360 | tags=56%, list=21%, signal=44% | ACVR2A/NELL1/SFRP2/IGF1/CTHRC1/TMEM119/CCN4/LRP5/ACVR2B/BMP2/BMP6/ATRAID/BMP4/WNT7B/GNAS/PRKD1/JAG1/DDR2/WWTR1/YAP1/LRP3/IL6ST/JUND/BMPR1A/MEF2C/SMAD5/BMPR1B/FERMT2/ZHX3/BMP7/GDPD2/CTNNBIP1/GLI3 |
| GOBP | SMAD PROTEIN SIGNAL TRANSDUCTION | 73 | -0.38965301 | -1.575054405 | 0.004883833 | 0.026416737 | 0.018452518 | 4040 | tags=34%, list=19%, signal=28% | TGFB2/BMP2/BMP6/MSTN/SLC2A10/BMP4/DAB2/WWTR1/CCN3/FOS/RBPMS/BMPR1A/GDF7/ROR2/ZMIZ1/SMAD9/SKI/SMAD5/SMAD3/ATOH8/CILP/BMP7/VIM/MAGI2/INHBB |
| GOBP | PROTEIN PHOSPHOPANTETHEINYLATION | 297 | 0.303826571 | 1.376824274 | 0.004869251 | 0.026359089 | 0.018412249 | 4163 | tags=31%, list=20%, signal=25% | ZC3H12A/UBE2F/TGM1/ZDHHC21/RABGGTA/UBE2L6/ABHD17C/UCHL3/GALNT6/ERP44/TGM3/HCCS/ERO1A/UFD1/OTUB2/ZDHHC13/USP15/UBL4A/PSMD14/UCHL5/GALNT18/USP18/P4HB/LYPLA2/ZDHHC12/COPS5/YKT6/GOLGA7B/RBX1/CRELD2/MOCS3/TTL/GLUL/PPT2/GALNT3/ZDHHC24/GLRX2/TTLL12/STAMBP/SAE1/USP38/ATG7/PDIA4/UBE2M/ALG13/USP43/ATG3/HERC5/TPST2/LYPLA1/JMJD6/BRCC3/USP3/B3GALT6/PDIA6/USP39/USP14/QSOX1/RNF7/UBA6/MDM2/ZDHHC6/DLAT/ZDHHC16/B3GNT3/USP35/DOHH/ZDHHC20/JOSD1/PLOD2/GCNT4/UBE2E1/B4GALT7/USP2/ABHD16A/TTLL5/B3GNT8/OTUB1/RABGGTB/UBA7/ZDHHC4/YOD1/MINDY1/JOSD2/OTULIN/UBE2E2/ABHD17B/ZDHHC5/STAMBPL1/GALNT5/GALNT14/ERP27 |
| GOBP | REGULATION OF CYTOKINESIS | 85 | 0.402727364 | 1.559305584 | 0.004858433 | 0.026321739 | 0.01838616 | 2062 | tags=24%, list=10%, signal=21% | KIF20A/AURKB/PRC1/AURKA/RAB11A/CDC6/KIF14/PLK2/CDC25B/ECT2/E2F8/KIF23/RACGAP1/PLK1/KIF20B/PLK3/PLK4/BRCA2/GIPC1/CETN2 |
| GOBP | DEOXYRIBONUCLEOTIDE BIOSYNTHETIC PROCESS | 14 | 0.66303942 | 1.751803712 | 0.004843575 | 0.02626242 | 0.018344725 | 1637 | tags=43%, list=8%, signal=40% | RRM2/TYMS/CMPK2/DTYMK/ADK/RRM1 |
| GOBP | CENTROSOME SEPARATION | 15 | 0.651424726 | 1.761998736 | 0.004834972 | 0.026236949 | 0.018326933 | 2583 | tags=47%, list=12%, signal=41% | NEK2/AURKA/KIF11/RANBP1/NDE1/NDEL1/NSFL1C |
| GOBP | PATHWAY RESTRICTED SMAD PROTEIN PHOSPHORYLATION | 62 | -0.407998866 | -1.596559305 | 0.004831035 | 0.026236777 | 0.018326813 | 4040 | tags=35%, list=19%, signal=29% | TGFB2/BMP2/BMP6/LDLRAD4/MSTN/BMP4/DAB2/NOG/DKK1/ACVRL1/SNX25/RBPMS/GREM1/PMEPA1/BMPR1A/GDF7/SDCBP/TGFBR2/BMP7/ACVR1B/INHBB/TGFBR3 |
| GOBP | MALE GENITALIA DEVELOPMENT | 20 | -0.590892184 | -1.793708694 | 0.004817262 | 0.026183143 | 0.018289348 | 4957 | tags=55%, list=24%, signal=42% | SYCP2/KLHL10/HSD17B3/BMP6/LGR4/TBX3/LHCGR/ROR2/PDGFRA/ASB1/AR |
| GOBP | HINDLIMB MORPHOGENESIS | 33 | -0.50529868 | -1.754201142 | 0.004812782 | 0.026179975 | 0.018287135 | 3223 | tags=42%, list=15%, signal=36% | HOXD9/BMP4/GNAS/TBX3/FMN1/TWIST1/BMPR1A/GPC3/AFF3/TFAP2B/OSR1/PTCH1/ZBTB16/OSR2 |
| GOBP | POSITIVE REGULATION OF INTERFERON BETA PRODUCTION | 33 | 0.529964982 | 1.7228263 | 0.004799712 | 0.026130038 | 0.018252254 | 3060 | tags=36%, list=15%, signal=31% | IRF7/POLR3G/IFIH1/IRF1/TLR2/TBK1/DHX58/POLR3B/HMGB2/POLR3D/TLR8/DDX58 |
| GOMF | CYSTEINE TYPE ENDOPEPTIDASE INHIBITOR ACTIVITY | 53 | 0.452996512 | 1.630909546 | 0.004721277 | 0.025723874 | 0.017968542 | 2979 | tags=28%, list=14%, signal=24% | SERPINB3/SERPINB13/PTTG1/LTF/BIRC5/CSTB/CSTA/CST7/SERPINB9/RPS6KA1/BIRC3/CD27/CARD16/PRDX5/TNFAIP8 |
| GOBP | PEPTIDYL ARGININE METHYLATION | 15 | 0.652531628 | 1.764992727 | 0.00470341 | 0.02564733 | 0.017915074 | 4579 | tags=53%, list=22%, signal=42% | PRMT5/PRMT3/PRMT6/PRDM4/PRMT1/PRMT2/PRDM14/NDUFAF7 |
| GOBP | TOLL LIKE RECEPTOR 4 SIGNALING PATHWAY | 36 | 0.504797198 | 1.674070883 | 0.004681582 | 0.025549042 | 0.017846419 | 4098 | tags=42%, list=20%, signal=34% | NMI/LTF/IRAK1/S100A14/TNIP3/IFI35/LYN/MFHAS1/ITGB2/PTPN22/LY96/PELI1/PIK3AP1/LILRA2/TRIL |
| GOBP | PROTEIN LOCALIZATION TO CHROMOSOME TELOMERIC REGION | 30 | 0.542045419 | 1.710175302 | 0.004668568 | 0.025498734 | 0.017811278 | 4453 | tags=63%, list=21%, signal=50% | CCT5/MACROH2A1/CCT2/CCT3/GNL3/CCT7/PINX1/XRCC5/BRCA2/TCP1/DKC1/CCT6A/CCT8/TINF2/GNL3L/NABP2/ATR/WRAP53/TPP1 |
| GOBP | VASCULAR PROCESS IN CIRCULATORY SYSTEM | 239 | -0.279526298 | -1.34716986 | 0.004664496 | 0.025497223 | 0.017810222 | 3897 | tags=31%, list=19%, signal=25% | BMP6/ITGA1/RAMP2/ANGPT1/GPER1/EDN3/SLC2A10/FOXC2/NOS1/SLC27A1/SCPEP1/SLC12A2/NPR1/PDE3A/SLC22A3/MFSD2A/ADCY6/ATP2A3/AGT/ABCC9/ECE1/RGS2/INSR/LEP/FGFBP3/ATP2B1/EDN1/LRP3/SLIT2/ATP8A1/SLC16A7/F2R/DOCK5/DOCK4/ADRB1/CLDN5/PER2/EGFR/SLC7A2/SLC22A5/SLC29A1/ABCC4/APOE/FOXC1/ARHGAP35/AKAP12/ARHGAP42/KLF2/SLC8A1/P2RX1/KCNMA1/ROCK2/ADRA1A/PRKG1/PDE2A/FERMT2/ACTA2/SLC16A2/ATP2B4/SLC2A13/ATP1A2/ADRA2A/ABCC3/AGTR1/SLC38A2/CTNNBIP1/BBS2/CAV1/LEPR/SOD1/KCNMB4/DDAH1/ADRB2/SLCO3A1 |
| GOMF | IMMUNE RECEPTOR ACTIVITY | 127 | 0.364966723 | 1.508743056 | 0.004663086 | 0.025497223 | 0.017810222 | 5533 | tags=46%, list=27%, signal=34% | CXCR2/CXCR4/IL4R/CCR7/OSMR/IL12RB2/FPR1/IFNGR1/CXCR6/FCGR1B/CSF2RA/IL12B/IL21R/CSF2RB/IL18RAP/FPR3/IL7R/CCR5/IL2RG/IL2RA/ACKR2/HLA-DOB/IL13RA1/EBI3/IFNGR2/KLRD1/IL1RL2/CCR1/HLA-DRA/CCRL2/IL12RB1/IFNAR2/CCR2/LILRB2/IL10RA/IL22RA1/LILRB1/IL15RA/CRLF2/CSF3R/C5AR2/CR1/C3AR1/CXCR1/CXCR3/HLA-DOA/FCER1G/IL10RB/HLA-DQB1/IL2RB/FLT3/C5AR1/FPR2/MPL/IL6R/CCR4/CXCR5/IL22RA2/IL1R1 |
| GOMF | VOLTAGE GATED SODIUM CHANNEL ACTIVITY | 23 | -0.547869251 | -1.729067103 | 0.004662114 | 0.025497223 | 0.017810222 | 4636 | tags=35%, list=22%, signal=27% | SCN1B/SCN3A/SCN9A/CACNA1H/TPCN1/PKD2/SCN7A/SCN3B |
| GOBP | PURINE NUCLEOSIDE MONOPHOSPHATE METABOLIC PROCESS | 43 | 0.487278894 | 1.671557154 | 0.004651339 | 0.025487515 | 0.017803441 | 4210 | tags=44%, list=20%, signal=35% | AK2/GART/TJP2/APRT/ATIC/IMPDH1/NT5C2/HPRT1/AMPD3/ADSL/XDH/AK1/ADK/GMPS/CASK/CARD11/GMPR2/PRPS2/NT5C |
| GOBP | PIGMENT METABOLIC PROCESS | 66 | 0.434976064 | 1.619556463 | 0.00461524 | 0.025310351 | 0.017679689 | 3354 | tags=39%, list=16%, signal=33% | CDH3/WNT5A/HMOX2/SLC7A11/SRRD/GART/SHMT2/ALAS1/APRT/HMBS/HPRT1/HMOX1/GPR143/FECH/CTNS/GIPC1/COX10/PMEL/SLC25A39/GMPS/UROS/UGT1A8/FXN/TSPO/TYRP1/OPN3 |
| GOBP | REGULATION OF TELOMERE MAINTENANCE VIA TELOMERE LENGTHENING | 56 | 0.44933238 | 1.632183563 | 0.004615065 | 0.025310351 | 0.017679689 | 3078 | tags=39%, list=15%, signal=34% | PRKCQ/AURKB/CCT5/NEK2/CCT2/HNRNPA2B1/CCT3/HNRNPC/CCT7/STN1/PINX1/PNKP/XRCC5/PIF1/TCP1/DKC1/FBXO4/CCT6A/NAT10/CCT8/PARP1/TINF2 |
| GOBP | CHROMOSOME ORGANIZATION INVOLVED IN MEIOTIC CELL CYCLE | 64 | 0.432130755 | 1.601715999 | 0.004601169 | 0.02527445 | 0.017654612 | 4365 | tags=30%, list=21%, signal=24% | BUB1B/CCNE1/BUB1/RAD51/TRIP13/CCNE2/BRIP1/SMC2/SIRT7/FANCD2/SMC4/SGO2/NCAPD2/NDC1/SYCE3/RAD50/REC8/RAD51C/HORMAD1 |
| GOBP | PYROPTOSIS | 17 | 0.625321403 | 1.74481236 | 0.004581349 | 0.025186175 | 0.01759295 | 3544 | tags=59%, list=17%, signal=49% | GZMB/CASP4/CASP1/GZMA/AIM2/GSDMC/CASP8/GSDMD/NLRC4/GSDME |
| GOCC | PROTON TRANSPORTING V TYPE ATPASE COMPLEX | 27 | 0.560080758 | 1.740361189 | 0.00454324 | 0.02499712 | 0.017460892 | 3724 | tags=48%, list=18%, signal=40% | ATP6V0D1/ATP6V1B2/ATP6V1G1/ATP6V0B/ATP6V1F/ATP6V1D/ATP6V1C1/ATP6V1C2/ATP6V1H/ATP6V1A/ATP6V0E1/TMEM199/ATP6V0A2 |
| GOCC | REPLISOME | 24 | 0.57543774 | 1.733930455 | 0.004517276 | 0.024874638 | 0.017375336 | 5394 | tags=58%, list=26%, signal=43% | PCNA/RPA3/DONSON/POLD2/MCM3/POLA2/PRIM1/PLRG1/POLD4/POLD3/RPA1/POLD1/PRPF19/PRIM2 |
| GOBP | GLUTATHIONE METABOLIC PROCESS | 54 | 0.462778318 | 1.676354493 | 0.004481427 | 0.024697477 | 0.017251587 | 4028 | tags=48%, list=19%, signal=39% | ETHE1/SLC7A11/CHAC1/CHAC2/GSTP1/GSTO1/CNDP2/GSTA4/GGCT/GPX1/GCLM/GLRX2/CTNS/PTGES2/GDAP1/GSTZ1/PARK7/MGST2/G6PD/GDAP1L1/GCLC/OPLAH/CLIC3/GSTM4/SLC1A1/GSTK1 |
| GOBP | CARBOHYDRATE BIOSYNTHETIC PROCESS | 199 | 0.324671308 | 1.412846413 | 0.004473205 | 0.024672404 | 0.017234073 | 4085 | tags=32%, list=20%, signal=26% | ENO1/TPI1/PGAM1/PGK1/PGM2/GOT2/HAS3/FUT3/GOT1/PTAFR/PGD/SMPD3/ST6GALNAC1/PTPN2/MAEA/MPDU1/MDH2/ALDOA/SLC25A13/GAPDH/SLC2A1/NFKB1/PPP1CA/SLC25A10/TALDO1/SIRT7/IMPA2/GPI/NANS/GYS1/SESN2/PCK2/SLC25A11/PLEK/MDH1/SLC37A4/GYG1/ATF4/CHST11/P2RY1/PASK/EXT1/SDHAF3/G6PD/SLC35B4/P2RY6/B3GNT3/PGP/EXT2/PC/ST3GAL4/B3GNT8/CD244/G6PC1/ENO3/GSK3A/AVPR1B/PHKG2/PPP1R3D/PGK2/NTSR1/MTOR/NDST1/TKT |
| GOBP | INTERFERON ALPHA PRODUCTION | 28 | 0.544794533 | 1.709264837 | 0.004473007 | 0.024672404 | 0.017234073 | 3140 | tags=43%, list=15%, signal=36% | IRF7/STAT1/NMI/IFIH1/MMP12/TBK1/LILRA4/HSPD1/TLR8/NLRC3/DDX58/CHUK |
| GOBP | ALPHA AMINO ACID METABOLIC PROCESS | 182 | 0.340231321 | 1.462056187 | 0.004469759 | 0.024672404 | 0.017234073 | 4610 | tags=35%, list=22%, signal=27% | KYNU/GOT2/SLC7A11/ALDH7A1/GOT1/AHCY/CTPS1/GPT2/GART/IDO1/PSPH/AASS/IL4I1/SHMT2/MTHFD1/ARG1/ASL/HAL/HSD17B10/NOS2/RIDA/ALDH4A1/TDO2/MTRR/ADI1/NAGS/THAP4/GLUL/NIT2/GCLM/DHFR/GCAT/TST/GSTZ1/SARS1/GMPS/ATF4/PHGDH/PARK7/GCDH/KYAT3/ALDH5A1/SERINC5/PSAT1/ILVBL/GCLC/PLOD2/DLD/ASNS/GAD1/SLC7A7/MTHFR/CAD/BAAT/PIPOX/AASDHPPT/ENOPH1/SLC25A12/ALDH18A1/TYR/ODC1/PYCR1/SARDH |
| GOBP | EXECUTION PHASE OF APOPTOSIS | 78 | 0.407945963 | 1.554423376 | 0.004465841 | 0.024672404 | 0.017234073 | 3321 | tags=29%, list=16%, signal=25% | ZC3H12A/CASP4/CASP1/CASP7/PLSCR1/CFLAR/TOP2A/DNASE1L3/CASP5/CASP10/IL6/ENDOG/DEDD2/ST20/CASP8/BOK/KPNB1/HMGB2/FASLG/BAX/HTRA2/CASP3/CIDEB |
| GOBP | SPROUTING ANGIOGENESIS | 120 | -0.333002369 | -1.467531174 | 0.004459004 | 0.024672404 | 0.017234073 | 4235 | tags=40%, list=20%, signal=32% | KDR/MIA3/HDAC9/HDAC5/RAMP2/ANGPT1/MAP2K5/FOXC2/NRP1/VEGFC/CREB3L1/FGF1/EGR3/ABL1/BMP4/ITGA5/SRPX2/JCAD/RSPO3/SEMA3E/RECK/VEGFB/CLEC14A/SLIT2/SPRED1/PGF/MMRN2/SEMA6A/PARVA/ACVRL1/ADGRA2/SYNJ2BP/PIK3CB/GREM1/KLF2/KLF4/AKT3/RHOJ/STARD13/MEOX2/FBXW7/CCBE1/GATA2/MAP3K3/AGTR1/JAK1/EFNB2/EPN2 |
| GOMF | DOUBLE STRANDED RNA BINDING | 75 | 0.409994206 | 1.548703138 | 0.004392548 | 0.024327399 | 0.016993082 | 4608 | tags=40%, list=22%, signal=31% | OAS2/OASL/OAS1/ILF2/IFIH1/OAS3/TARBP2/DDX60/DHX58/SLC3A2/ADAR/TFRC/ELAVL1/DHX33/EIF4H/STAU2/HSPD1/TLR8/YRDC/DDX21/DUS2/DDX58/APTX/MRPL44/SIDT1/MSN/TUBB4B/EIF2AK2/DHX15/TUBA1B |
| GOBP | POSITIVE REGULATION OF T CELL MEDIATED IMMUNITY | 48 | 0.463316074 | 1.637287461 | 0.004392548 | 0.024327399 | 0.016993082 | 4438 | tags=56%, list=21%, signal=44% | FZD5/RSAD2/CYRIB/SASH3/IL1B/IL12B/IL6/IL23A/FADD/HLA-E/HSPD1/XCL1/CD1B/HLA-DRA/IL12RB1/B2M/HLA-F/HLA-B/NECTIN2/NLRP3/HLA-G/FOXP3/TRAF2/FBXO38/CD1D/PTPRC/MALT1 |
| GOCC | PROTEIN DNA COMPLEX | 177 | 0.335764999 | 1.438480574 | 0.004364674 | 0.024212945 | 0.016913134 | 5934 | tags=42%, list=28%, signal=30% | GINS3/H2AX/MCM6/PCNA/MACROH2A1/H2AZ1/GINS2/TOP1/POLR2H/RPA3/MCM4/CDC45/GINS1/MCM5/MCM2/CENPA/STN1/H2AJ/DONSON/POLD2/WDR18/MCM7/XRCC6/MCM3/XRCC5/PRKDC/POLA2/NPM1/PRIM1/H2BC9/MACROH2A2/H3C10/LEF1/GTF2E1/PLRG1/POLD4/PARP1/TINF2/GTF2B/POLD3/H2AC4/H2AC13/JUP/H2BC10/SLF1/H1-2/RPA1/H3C7/POLD1/NFYC/H2BC12/H3C8/H4C4/H2AC16/H4C9/H2BC13/H1-5/PRPF19/H2BC6/H4C2/GLYR1/GINS4/H1-3/TERT/PRIM2/H4C5/H1-1/KDM1B/IRF4/MYOG/H2BC7/PRM3/H2BC14/H3C2 |
| GOBP | REGULATION OF EXTRACELLULAR MATRIX ASSEMBLY | 15 | -0.634383005 | -1.785958567 | 0.004344714 | 0.02412214 | 0.016849705 | 2962 | tags=80%, list=14%, signal=69% | HAS2/TIE1/EMILIN1/SOX9/PHLDB2/AGT/RGCC/CLASP1/ANTXR1/PHLDB1/DAG1/SMAD3 |
| GOMF | EXORIBONUCLEASE ACTIVITY | 39 | 0.492502935 | 1.65201403 | 0.00433773 | 0.024103282 | 0.016836532 | 3878 | tags=49%, list=19%, signal=40% | ZC3H12A/ISG20/EXOSC4/PNPT1/EXOSC3/ISG20L2/DIS3/PDE12/CNOT1/EXOSC2/ERI1/ERI2/USB1/EXOSC5/XRN2/NOCT/CNOT6/TOE1/DCPS |
| GOMF | CADHERIN BINDING | 328 | 0.302369852 | 1.377621034 | 0.004332869 | 0.024096202 | 0.016831587 | 4256 | tags=31%, list=20%, signal=25% | ENO1/MICALL1/STAT1/RAN/CDH3/EPHA2/CCNB2/RUVBL1/BZW1/TWF1/LRRC59/PSMB6/HSPA5/TXNDC9/DIAPH3/TJP2/S100A11/SFN/VAPA/ATIC/LDHA/S100P/RANBP1/ALDOA/TMPO/FSCN1/SNX1/LAD1/PRDX1/PPP1CA/DBNL/EXOC3/RARS1/BAG3/RAB1A/LYPLA2/CKAP5/BZW2/ANLN/YKT6/HSPA8/NOP56/CAPZA1/EPS8L2/SLC3A2/CNN2/RAB10/MYO1B/EHD4/GIPC1/DNAJB1/SERBP1/RANGAP1/PKP3/ARFIP2/SND1/TMOD3/EIF4H/UBFD1/YWHAZ/PARK7/EIF4G2/LARP1/H3C10/ABI1/SCRIB/MAPRE1/PFKP/CLINT1/PFN1/PLIN3/EFHD2/PKM/CCT8/MYH9/CHMP5/UNC45A/PRDX6/TBC1D2/ANXA1/BAIAP2/PROM1/EIF5/OLA1/TRIM25/PAK6/NIBAN2/PLCB3/P2RX4/CAPG/DHX29/CDC42EP1/SNX5/EMD/CDH1/JUP/FMNL2/ARHGAP1/LIMA1/VASP/TAGLN2/H3C7 |
| GOMF | OXIDOREDUCTASE ACTIVITY ACTING ON A SULFUR GROUP OF DONORS NAD P AS ACCEPTOR | 11 | 0.707592682 | 1.75600089 | 0.004320784 | 0.024048904 | 0.016798548 | 3457 | tags=64%, list=17%, signal=53% | TXNDC17/PGK1/SELENOT/TXNRD1/TXN/TXNRD3/DLD |
| GOMF | PEPTIDOGLYCAN BINDING | 18 | 0.628826782 | 1.783252485 | 0.004314483 | 0.024033746 | 0.01678796 | 3769 | tags=44%, list=18%, signal=36% | NOD2/PGLYRP4/TLR2/RNASE7/PGLYRP2/TREM2/PGLYRP3/NLRP3 |
| GOBP | RNA SPLICING VIA TRANSESTERIFICATION REACTIONS | 342 | 0.296125769 | 1.354478416 | 0.004310331 | 0.024030543 | 0.016785723 | 4998 | tags=35%, list=24%, signal=27% | EIF4A3/DDX39A/C1QBP/ESRP2/PPIL1/SNRPG/POLR2H/SNRPF/SNRPD1/HNRNPA2B1/NCBP1/SRSF9/POLR2F/PRMT5/HNRNPC/DDX41/LSM2/BUD31/TXNL4A/SF3B3/LSM7/SNRPD3/EFTUD2/WDR77/SNRNP25/SF3B5/LSM1/CD2BP2/SNU13/UBL5/CPSF3/PPIH/PSPC1/ALYREF/SNRPA1/GEMIN6/MAGOHB/CSTF2/SNRPC/GEMIN7/HSPA8/SF3B6/LSM5/LSM4/POLR2L/SNRPB/SRSF2/AQR/SRPK1/POLR2G/DAZAP1/LUC7L2/ELAVL1/POLR2D/PRPF31/JMJD6/PRPF38A/SRSF7/SRSF1/USP39/STRAP/HNRNPF/LSM6/HNRNPM/ISY1/CLP1/PRPF4/POLR2E/PLRG1/PTBP1/YBX1/PRPF4B/PRDX6/PQBP1/GEMIN2/SNRPA/CPSF4/PNN/MBNL3/CSTF1/MAGOH/WDR33/POLR2I/PRCC/SNRNP40/KDM1A/CSTF3/TFIP11/TRA2B/DCPS/SRSF3/CWF19L1/SMNDC1/POLR2J/TXNL4B/PRPF18/CWC25/DHX15/RBM7/HNRNPK/SRSF8/NCL/PAPOLA/PRPF40A/ZBTB7A/LSM3/UPF3B/SNRPD2/DDX46/YJU2/WDR83/PUF60/SRSF10/FUS/PRPF19/GEMIN8/CCAR1/SYNCRIP/SAP18 |
| GOBP | POSITIVE REGULATION OF TRANSCRIPTION OF NUCLEOLAR LARGE RRNA BY RNA POLYMERASE I | 10 | 0.727906023 | 1.757828172 | 0.004308725 | 0.024030543 | 0.016785723 | 4534 | tags=80%, list=22%, signal=63% | IPPK/MARS1/PIH1D1/SMARCA4/NOL11/MTOR/PWP1/NCL |
| GOMF | PROTEASOME BINDING | 15 | 0.656586487 | 1.775960465 | 0.004308725 | 0.024030543 | 0.016785723 | 2523 | tags=53%, list=12%, signal=47% | PSME4/ID1/PSMD14/PSMG1/UCHL5/PSMF1/ADRM1/USP14 |
| GOMF | LIGAND ACTIVATED TRANSCRIPTION FACTOR ACTIVITY | 50 | -0.446817047 | -1.698292223 | 0.004306498 | 0.024030543 | 0.016785723 | 3266 | tags=46%, list=16%, signal=39% | NR1D2/NR1I3/RARA/NR2C1/NR2F1/RORB/RORC/NR2C2/ESRRG/NR1H3/NR5A2/ESR1/PPARG/PGR/NR2F2/PPARA/THRA/ARNT/NR3C1/RXRA/AR/NR3C2/RORA |
| GOBP | T CELL SELECTION | 48 | 0.464361367 | 1.640981365 | 0.004277712 | 0.023928115 | 0.016714175 | 5438 | tags=62%, list=26%, signal=46% | CCR7/PTPN2/STAT3/CTSL/CD3D/ATG5/IL12B/SYK/IL6/IL23A/BATF/CD3G/THEMIS/CD3E/IL12RB1/FOXN1/IL15/CARD11/ZAP70/DOCK2/MTOR/FOXP3/CD1D/PTPRC/CD28/AIRE/TBX21/SLAMF6/STK11/IL6R |
| GOBP | POSITIVE REGULATION OF BIOMINERALIZATION | 47 | -0.448201061 | -1.690227486 | 0.0042776 | 0.023928115 | 0.016714175 | 5450 | tags=60%, list=26%, signal=44% | ACVR2A/NELL1/KL/PKDCC/WNT6/TMEM119/ACVR2B/BMP2/BMP6/ATRAID/BMP4/ADGRV1/TFAP2A/ATP2B1/ANO6/FBLN7/P2RX7/GPM6B/BMPR1A/PTN/MEF2C/SLC8A1/BMPR1B/SMAD3/BMP7/OSR1/OSR2/ADRB2 |
| GOMF | TRANSFERASE ACTIVITY TRANSFERRING ONE CARBON GROUPS | 216 | 0.328168668 | 1.440928569 | 0.004269532 | 0.023922201 | 0.016710044 | 4688 | tags=35%, list=22%, signal=28% | CIAPIN1/TYMS/ECE2/EZH2/SMYD2/GART/WDR4/DIMT1/SHMT2/COMTD1/PRMT5/PRDM1/ATIC/DNMT1/NOP2/FTSJ1/NTMT1/PRMT3/METTL6/METTL1/HENMT1/ICMT/FAM98A/TRMT10C/DNMT3B/TFB2M/PRMT6/SUV39H2/EMG1/PRDM4/COQ3/NSD2/NSUN2/RRP8/CMTR2/TRMT6/TRMT112/TPMT/SETD4/METTL8/LCMT1/SPOUT1/CARNMT1/ANTKMT/PRDM2/CSKMT/DOT1L/MTFMT/MEN1/MECOM/ARMT1/PRMT1/MRM2/TRMT1/LCMT2/METTL4/EED/MRM3/THUMPD3/TYW3/DNMT3A/FDXACB1/CAD/METTL22/BUD23/NSD1/PRDM11/PRMT2/METTL21EP/PRDM14/TRMT5/SMYD5/METTL5/NDUFAF7/SUV39H1/SETD9 |
| GOBP | NEGATIVE REGULATION OF LEUKOCYTE CELL CELL ADHESION | 120 | 0.372747015 | 1.531106155 | 0.004267098 | 0.023922201 | 0.016710044 | 3986 | tags=33%, list=19%, signal=27% | ZC3H12A/CD274/IL4R/TNFRSF21/IDO1/PTPN2/IRF1/PLA2G2F/ARG1/CEBPB/JAK3/SOCS1/CBFB/TIGIT/ZC3H8/GLMN/LGALS9/PTPN22/LAG3/IL2RA/LILRB4/LAPTM5/CD86/CRTAM/TMEM131L/SCRIB/SFTPD/XCL1/CLEC4G/ANXA1/PELI1/CASP3/LILRB2/PAG1/LILRB1/HLA-G/CD300A/CD80/IL10/FOXP3 |
| GOMF | 3 5 EXONUCLEASE ACTIVITY | 55 | 0.4522091 | 1.640102801 | 0.004264488 | 0.023922201 | 0.016710044 | 5259 | tags=49%, list=25%, signal=37% | TREX2/ISG20/EXOSC4/PNPT1/EXOSC3/ISG20L2/APEX2/DIS3/PDE12/CNOT1/EXOSC2/ERI1/ERI2/USB1/EXOSC5/XRN2/NOCT/CNOT6/TOE1/REXO4/RAD50/POLD1/ANGEL1/REXO2/POLG/APEX1/APLF |
| GOCC | MYELIN SHEATH | 39 | -0.456469492 | -1.645104295 | 0.004257838 | 0.023916519 | 0.016706075 | 1324 | tags=38%, list=6%, signal=36% | GDI1/MBP/PMP2/MPZ/PTEN/DLG1/PLP1/TSPAN2/ITPR3/CA13/JAM3/GSN/BCL2/TUBA1A/PLLP |
| GOBP | SOMITOGENESIS | 63 | -0.402996147 | -1.574210864 | 0.004241901 | 0.02384694 | 0.016657473 | 5538 | tags=46%, list=27%, signal=34% | SFRP1/POFUT1/MED12/SFRP2/DLL1/EP300/PCDH8/SMAD4/SEMA3C/ATM/KAT2A/XRCC2/FOXC2/MIB1/MEOX1/NKD1/DMRT2/LRP6/FOXC1/BMPR1A/ROR2/ALDH1A2/SMAD3/RBPJ/MEOX2/LFNG/PLXNA2/AXIN2/COBL |
| GOBP | OUTFLOW TRACT SEPTUM MORPHOGENESIS | 25 | -0.548012674 | -1.769511526 | 0.004220967 | 0.023749128 | 0.01658915 | 5056 | tags=48%, list=24%, signal=36% | SMAD4/TBX1/SEMA3C/TGFB2/NRP1/BMP4/ROBO2/PARVA/FGFR2/BMPR1A/TGFBR2/ZFPM2 |
| GOBP | POSITIVE T CELL SELECTION | 36 | 0.507488942 | 1.682997576 | 0.004204485 | 0.023676221 | 0.016538223 | 5931 | tags=67%, list=28%, signal=48% | PTPN2/STAT3/CTSL/CD3D/IL12B/IL6/IL23A/BATF/CD3G/THEMIS/CD3E/IL12RB1/FOXN1/ZAP70/DOCK2/MTOR/FOXP3/PTPRC/TBX21/SLAMF6/STK11/IL6R/IRF4/TNFSF18 |
| GOBP | TERMINATION OF RNA POLYMERASE II TRANSCRIPTION | 35 | 0.511401808 | 1.693596558 | 0.004204485 | 0.023676221 | 0.016538223 | 5659 | tags=63%, list=27%, signal=46% | SNRPG/SNRPF/NCBP1/SNRPD3/CPSF3/LSM10/CSTF2/SNRPB/CLP1/TTF2/CPSF4/SSU72/CSTF1/WDR33/CSTF3/PAPOLA/SLBP/SYMPK/SNRPE/NCBP2/MED18/ZNF473 |
| GOBP | PYRIMIDINE NUCLEOSIDE TRIPHOSPHATE BIOSYNTHETIC PROCESS | 17 | 0.627493719 | 1.750873696 | 0.004196907 | 0.023673234 | 0.016536137 | 615 | tags=35%, list=3%, signal=34% | UCK2/NME1/CTPS1/TYMS/CMPK2/DTYMK |
| GOMF | EXONUCLEASE ACTIVITY ACTIVE WITH EITHER RIBO OR DEOXYRIBONUCLEIC ACIDS AND PRODUCING 5 PHOSPHOMONOESTERS | 57 | 0.456723272 | 1.657837132 | 0.004195398 | 0.023673234 | 0.016536137 | 4366 | tags=44%, list=21%, signal=35% | TREX2/ISG20/EXOSC4/FEN1/PNPT1/EXOSC3/EXO1/ISG20L2/APEX2/DIS3/PDE12/CNOT1/EXOSC2/ERI1/ERI2/USB1/EXOSC5/APTX/XRN2/NOCT/CNOT6/TOE1/PLD4/POLD1/ANGEL1 |
| GOBP | HISTONE ARGININE METHYLATION | 11 | 0.70926359 | 1.760147508 | 0.004190838 | 0.023673234 | 0.016536137 | 4346 | tags=55%, list=21%, signal=43% | PRMT5/PRMT6/PRDM4/PRMT1/PRMT2/PRDM14 |
| GOMF | TRANSLATION FACTOR ACTIVITY RNA BINDING | 78 | 0.408719028 | 1.557369035 | 0.004181397 | 0.023645306 | 0.016516628 | 4350 | tags=45%, list=21%, signal=36% | GSPT1/EIF2B2/TSFM/EIF4E2/MCTS1/EIF2S2/EIF4E/EIF6/EIF3J/EIF2S1/EIF3I/MRPL58/COPS5/DENR/EIF3K/GTPBP2/TUFM/MTIF2/EIF3B/EIF2B1/EIF4H/EIF4G2/EIF2B4/ABCF1/EIF4G1/EIF5/EIF2B5/EIF5A/EIF2B3/DHX29/MTRF1L/EIF3A/CPEB4/EFL1/EIF3M |
| GOBP | PLASMINOGEN ACTIVATION | 21 | 0.601810526 | 1.753935866 | 0.004147184 | 0.023471591 | 0.016395286 | 1669 | tags=29%, list=8%, signal=26% | ENO1/PGK1/PLGRKT/F12/PLAT/PLAU |
| GOBP | REGULATION OF NEUROGENESIS | 332 | -0.263829938 | -1.323386906 | 0.004144011 | 0.023471591 | 0.016395286 | 4588 | tags=32%, list=22%, signal=26% | CUX2/NUMB/SHTN1/LRP1/HEY1/SEMA3A/CERS2/RND2/CTNNB1/TRPC6/OBSL1/RUFY3/SORL1/SERPINF1/BMP2/EGR2/XRCC2/AMIGO1/RNF112/GPER1/SS18L1/SEMA6D/PTEN/NRP1/HOOK3/PLXND1/VEGFC/EFNB3/RELN/TIAM2/ANKRD27/ROBO2/HES1/CXCL12/NAP1L1/RTN4R/SOX10/SPEN/ZNF365/SHOX2/SEMA3E/GOLGA4/NPTN/YAP1/MACF1/L1CAM/PRUNE1/SLIT2/SNW1/NOG/IL6ST/SOX8/BHLHB9/FZD3/SPART/SEMA4G/MAP6/ARNTL/SEMA6A/DUSP10/FGF13/PPARG/CTNNA1/SEMA3B/PER2/PLAG1/LIF/SHANK3/RYK/TMEM98/BIN1/PTPRS/ASPA/NTRK2/FN1/BMPR1A/CAPRIN2/TRAK2/PTN/DOCK7/MAP2/ELL3/NTN1/DAG1/ETV5/PCM1/SKI/WNT2/RTN4/NUMBL/BMP7/HOXB3/RAPGEF2/STAR/PAK3/DAAM2/NIN/PLXNA2/SEMA3G/BHLHE41/DBN1/LRP4/FBXO31/GLI3/KIT/MAP1B/EEF2K |
| GOBP | RESPONSE TO STEROID HORMONE | 320 | -0.26267555 | -1.314702618 | 0.004144011 | 0.023471591 | 0.016395286 | 4666 | tags=34%, list=22%, signal=27% | CALCOCO1/ABCA2/TRIM68/KCTD6/MAOB/MGARP/HEY1/HDAC6/NCOA1/CRY1/MED1/NCOA4/RBBP7/AVPR1A/CPS1/TGFB2/SERPINF1/BMP6/RAMP2/PCK1/GPER1/MSTN/PADI2/NCOA2/TFPI/HSD11B2/UBE3A/HSD3B1/NR2C1/DSG1/NCOR1/FOXO1/SIRT1/HNMT/GPAM/BMP4/KMT2D/CCN2/DAB2/PTPRU/SMYD3/LOX/SCGB2A2/UFSP2/FAM107A/SOX10/YAP1/ESRRG/NR1H3/ATP2B1/EDN1/GNRH1/SLIT2/ADIPOQ/LMO3/DUSP1/SCGB2A1/ACSBG1/FOS/UFM1/ARNTL/ESR1/PLPP1/ACTA1/EGFR/BCHE/CDO1/PGR/CLOCK/SLIT3/DSG2/AKR1C3/ABHD2/VPS11/PAM/DDX17/KLF9/ZFP36L1/PMEPA1/PPARA/FBXO32/PTN/ZMIZ1/PER1/RBFOX2/ALAD/TGFBR2/BMP7/STAR/ABCA3/ATP1A2/NR3C1/AQP1/NCOR2/RXRA/ZFP36L2/FIBIN/AR/TAF7/BCL2/CAV1/NR3C2/KANK2/PGRMC2/CCND1/CRY2/TXNIP/CLDN1 |
| GOBP | NEGATIVE REGULATION OF PROTEIN CONTAINING COMPLEX DISASSEMBLY | 79 | -0.374894934 | -1.54893123 | 0.004133829 | 0.023455289 | 0.016383899 | 3828 | tags=39%, list=18%, signal=32% | APC/TRIOBP/DMTN/CAMSAP2/PIK3CA/ARHGEF2/RDX/SCAF4/SWAP70/ATXN7/CLASP1/ADD1/TMOD2/FGF13/PLEKHH2/BMERB1/VILL/SPTAN1/CAMSAP3/TTBK2/SCIN/SVIL/SCAF8/EPS8/SPTBN2/ADD3/LMOD1/TMOD1/GSN/SPTBN1/MAP1B |
| GOBP | PROGRAMMED NECROTIC CELL DEATH | 43 | 0.489330426 | 1.678594712 | 0.004124779 | 0.023423726 | 0.016361851 | 3206 | tags=42%, list=15%, signal=36% | PPIF/PYGL/CASP1/CFLAR/MLKL/PGAM5/BIRC3/RIPK3/CASP8/FADD/BOK/ALKBH7/LY96/TNF/MAP3K5/FASLG/BAX/PELI1 |
| GOBP | TRNA 5 LEADER REMOVAL | 12 | 0.698073476 | 1.769559318 | 0.004118699 | 0.023408982 | 0.016351552 | 4057 | tags=67%, list=19%, signal=54% | RPP40/POP5/RPP25L/POP4/POP1/POP7/RPP30/RPP38 |
| GOMF | HYDROLASE ACTIVITY ACTING ON CARBON NITROGEN BUT NOT PEPTIDE BONDS IN CYCLIC AMIDINES | 33 | 0.532691571 | 1.73168998 | 0.004118473 | 0.023408982 | 0.016351552 | 5663 | tags=48%, list=27%, signal=35% | GDA/GCH1/APOBEC3B/ATIC/MTHFD1/MTHFD2/AMPD3/ADAR/ADA2/DCTD/ADAT3/APOBEC3C/MTHFD2L/APOBEC3G/ADAT2/ADAT1 |
| GOBP | RNA CATABOLIC PROCESS | 401 | 0.296521802 | 1.373011052 | 0.004101994 | 0.023353555 | 0.016312835 | 3629 | tags=29%, list=17%, signal=25% | ZC3H12A/PSME2/CARHSP1/GSPT1/PSMB2/EIF4A3/PSMA5/PSMB5/OAS2/PSMA3/MYD88/PSMD12/PSME4/PSMA2/ISG20/PSME1/SLIRP/PSMB6/EXOSC4/PSMB10/PSMB3/PSMD6/PSMB8/PPP2CA/PSMC4/RNASEH2A/PSMA4/ZPR1/PSMD2/NCBP1/FEN1/HNRNPC/PNPT1/HELZ2/LSM2/FASTKD2/MRTO4/PSMD11/PSMD1/LSM7/FASTKD5/PYM1/PSMD14/PSMC3/PSMB9/EXOSC3/LSM1/FASTKD1/CNOT11/RIDA/PSMA7/DIS3/PSMD9/PSMD8/MAGOHB/TBRG4/HSPA8/PDE12/PPP2R2A/PSMA1/DNA2/LSM5/EXOSC1/ZC3H12D/PSMD13/LSM4/DEDD2/TUT7/PSMF1/PSMC5/NSUN2/PSMC2/CNOT1/PSMB1/ZFP36/PSMC6/DDX49/POLR2G/PSME3/EXOSC2/PSMD7/SERBP1/PKP3/NPM1/ELAVL1/POLR2D/SND1/PSMC1/TNFRSF1B/GRSF1/TARDBP/YWHAZ/DKC1/EXOSC5/LSM6/LARP1/SKIV2L/HNRNPM/NRDE2/IGF2BP3/POP1/YBX1/PSMB7/EIF4G1/XRN2/PSMD10/NOCT/MAGOH/CNOT6/PRKCD/PPP2R1A/YTHDF2/MAPKAPK2/SMG8/EXOSC8/RPLP0/DAZL/FASTKD3 |
| GOBP | CELL CELL RECOGNITION | 74 | 0.420581778 | 1.585641276 | 0.004099102 | 0.023353555 | 0.016312835 | 5578 | tags=36%, list=27%, signal=27% | FUT3/CCT5/CCR7/CCT2/CCT3/CCT7/ALDOA/TEX101/PRF1/VDAC2/CD6/CCL19/DOCK8/TCP1/CATSPERB/CCT8/SPA17/DOCK2/MSN/CATSPER1/NCK2/CLEC4M/PAEP/CD209/UBAP2L/ADAM20/ADAM30 |
| GOMF | GROWTH FACTOR BINDING | 136 | -0.329562632 | -1.488236632 | 0.004045593 | 0.023071556 | 0.016115855 | 4621 | tags=40%, list=22%, signal=32% | PDGFB/COL6A1/HTRA1/FGFRL1/LRRC32/TEK/CRIM1/KDR/CCN4/ACVR2B/LTBP2/NRDC/ITGAV/NRP1/FGFR3/ACVR1C/ERBB3/CEP57/KLB/HTRA4/SRPX2/CCN2/PDGFRB/GLG1/S100A13/GPC1/INSR/FGFBP3/IL6ST/CCN3/CD109/DUSP1/IGF1R/TSKU/A2M/PDGFA/EGFR/FGFBP2/ACVRL1/KAZALD1/LTBP4/FGFR2/IL11RA/NTRK2/GHR/FGFR1/PTN/IGFBP5/IGFBP6/CCN5/TGFBR2/ACVR1B/LIFR/PDGFRA/TGFBR3 |
| GOBP | NEGATIVE REGULATION OF CANONICAL WNT SIGNALING PATHWAY | 171 | 0.339179527 | 1.449715059 | 0.004019927 | 0.022944665 | 0.016027219 | 2454 | tags=28%, list=12%, signal=25% | PSME2/PSMB2/WNT5A/PSMA5/PSMB5/PSMA3/PSMD12/CSNK1A1/PSME4/PSMA2/PSME1/HDAC1/PSMB6/RUVBL2/PSMB10/PSMB3/PSMD6/PSMB8/PSMC4/PSMA4/FERMT1/PSMD2/SCYL2/PSMD11/PSMD1/PSMD14/PSMC3/PSMB9/SOST/PSMA7/PSMD9/PSMD8/RBX1/PSMA1/PSMD13/PSMF1/PSMC5/PSMC2/MAD2L2/TLE3/PSMB1/PSMC6/SLC9A3R1/PSME3/PSMD7/PSMC1/SIAH2/TMEM64 |
| GOCC | PHAGOCYTIC VESICLE | 134 | 0.367874101 | 1.527862373 | 0.004017469 | 0.022944665 | 0.016027219 | 4044 | tags=33%, list=19%, signal=27% | TRIM14/RAB38/NOD2/LTF/ATP6V0D1/RAB11A/ATP6V0B/TAP1/TLR2/TAP2/STX6/CLCN3/RAB31/GNLY/RAB5A/ATG5/CORO1A/SYK/RAC2/CD36/RAB10/VAMP8/NCF2/ADAM8/CTSS/HLA-E/VAMP7/TAPBP/CYBA/ATP6V0E1/SNX3/STX4/RAB8A/B2M/HLA-F/BECN1/WAS/HLA-B/ATP6V0A2/MPEG1/HLA-G/MTOR/PLD4/ZDHHC5 |
| GOBP | DIGESTIVE SYSTEM DEVELOPMENT | 127 | -0.335296126 | -1.495512743 | 0.004007063 | 0.022910172 | 0.016003126 | 5374 | tags=40%, list=26%, signal=30% | AGR2/SFRP2/HOXA5/EDNRB/SPDEF/EPHB3/PKDCC/CHD8/FAT4/WNT11/CYP1A1/PCSK5/YIPF6/BBS7/CPS1/TGFB2/RARRES2/SAV1/SFRP5/LGR4/IFT172/CCKBR/BMP4/HES1/AHI1/SOX10/SHOX2/YAP1/IL6ST/DACT1/EGFR/FGFR2/NPHP3/CLMP/MYOCD/ALDH1A2/RBPMS2/SMAD3/TGFBR2/WDPCP/CBFA2T2/DCHS1/PKD1/WDR19/PDGFRA/GLI2/GLI3/KIT/BCL2/PDGFC/COBL |
| GOBP | POSITIVE REGULATION OF TELOMERE MAINTENANCE | 48 | 0.465282692 | 1.644237184 | 0.003990621 | 0.022835599 | 0.015951035 | 4174 | tags=48%, list=20%, signal=38% | PRKCQ/AURKB/CCT5/NEK2/CCT2/HNRNPA2B1/CCT3/GNL3/CCT7/PNKP/XRCC5/TCP1/DKC1/FBXO4/CCT6A/PML/CCT8/ERCC1/SIRT6/NABP2/RAD50/ATR/MAPKAPK5 |
| GOBP | GLYCOSYL COMPOUND CATABOLIC PROCESS | 40 | 0.493779472 | 1.666535455 | 0.003980703 | 0.022798263 | 0.015924955 | 2659 | tags=30%, list=13%, signal=26% | TYMP/UPP1/PNP/NT5C3A/AHCY/APOBEC3B/HPRT1/GBA2/XDH/NAGA/GLA/ADA2 |
| GOBP | CAMERA TYPE EYE MORPHOGENESIS | 114 | -0.336339117 | -1.476581921 | 0.003948564 | 0.022633494 | 0.015809861 | 3946 | tags=33%, list=19%, signal=27% | OBSL1/LRP5/PITX2/AQP5/NECTIN3/IFT172/COL8A1/MFSD2A/BMP4/RORB/TFAP2A/JAG1/AHI1/PTPRM/DIO3/SOX8/TSKU/SP3/TWIST1/LRP6/NTRK2/COL8A2/PTN/RBP4/ZHX2/SKI/WNT2/ABI2/IFT122/BMP7/TFAP2B/CDON/NOTCH2/GLI3/C12orf57/MEIS1/BCAR3/WNT2B |
| GOBP | POSITIVE REGULATION OF GLUCOSE IMPORT | 36 | -0.481140265 | -1.694976777 | 0.003941647 | 0.022613137 | 0.015795642 | 2533 | tags=33%, list=12%, signal=29% | INSR/ADIPOQ/RHOQ/GPC3/OSBPL8/KLF15/IRS1/PIK3R1/CREBL2/SORBS1/IRS2/MEF2A |
| GOBP | FEMALE SEX DIFFERENTIATION | 110 | -0.342859981 | -1.498963143 | 0.003924706 | 0.022535191 | 0.015741195 | 3121 | tags=30%, list=15%, signal=26% | SIRT1/ROBO2/KITLG/MERTK/TBX3/DACH1/LEP/GNRH1/SLIT2/LHCGR/ESR1/CTNNA1/PGR/SLIT3/FOXC1/ZFX/RBP4/BMPR1B/ZFPM2/FZD4/IMMP2L/STAT5B/PLEKHA1/ACVR1B/NUPR1/AXL/PDGFRA/INHBB/FST/KIT/BCL2/SOD1/ANG |
| GOBP | REGULATION OF HEART RATE | 91 | -0.365039255 | -1.555600087 | 0.003914326 | 0.022494815 | 0.015712992 | 4886 | tags=33%, list=23%, signal=25% | HRC/CAMK2D/SCN1B/SEMA3A/AVPR1A/TNNI3K/EDN3/TAC1/CALM2/BVES/KCNH2/EDN1/CACNB2/ADRB1/DSG2/ANK2/DSP/BIN1/POPDC2/SLC8A1/ADRA1A/PLN/AKAP9/SCN3B/CASQ2/GPD1L/CACNA2D1/DMD/TPM1/CAV1 |
| GOCC | VACUOLAR LUMEN | 165 | 0.342745859 | 1.461566328 | 0.003910711 | 0.022493287 | 0.015711924 | 4254 | tags=35%, list=20%, signal=28% | SERPINB3/HPSE/GM2A/FABP5/SERPINB13/CTSC/S100A7/PYCARD/GGH/VCP/CTSB/IFI30/PRSS2/CCT2/IMPDH1/CTSL/PLAC8/ARG1/PSMD1/TOLLIP/SERPINA3/LIPA/HSPA8/NSG1/NAPRT/LYZ/PA2G4/PPT2/PTGES2/CTSD/SMPD1/GRN/CTSA/GYG1/CPPED1/CTSS/GLA/ADA2/ARSA/ARHGAP45/FASLG/STK11IP/CCT8/PRDX6/PRKCD/LGMN/FRK/MNDA/UNC13D/TRAPPC1/CAP1/AGA/MAN2B1/NEU1/TUBB4B/CREG1/GALNS |
| GOCC | CAVEOLA | 80 | -0.37460315 | -1.557680174 | 0.003906229 | 0.022486757 | 0.015707364 | 3097 | tags=36%, list=15%, signal=31% | PACSIN2/EHD2/BVES/LRRK2/EFNA5/CAVIN3/INSR/SPRED1/F2R/NEU3/PLPP1/FXYD1/CTNNA1/LRP6/BMPR1A/KCNMA1/ADRA1A/MAPK1/ATP2B4/TGFBR2/CAVIN2/PTCH1/ATP1A2/IRS1/PTGIS/CAVIN1/DLC1/CAV2/CAV1 |
| GOBP | SULFUR COMPOUND TRANSPORT | 48 | 0.466376049 | 1.648100939 | 0.003875785 | 0.022330639 | 0.015598312 | 4983 | tags=44%, list=24%, signal=33% | SLC26A9/SLC7A11/ABCC1/RACGAP1/SLC5A6/LRRC8D/SLC25A10/SLC36A1/CTNS/SLC25A11/SLC9A3R1/SLC26A4/SLC25A19/SLC38A7/GJA1/SLC1A1/SLC1A4/SLC13A4/ABCC5/SLC19A3/SLC6A6 |
| GOBP | TUBE FORMATION | 139 | -0.32392953 | -1.462066628 | 0.00385883 | 0.022252037 | 0.015543407 | 5366 | tags=45%, list=26%, signal=34% | CECR2/FGF2/SFRP1/MED12/SFRP2/HESX1/CEP290/TSC2/TEAD2/IRX3/OPA1/CTHRC1/SEMA4C/WNT6/RPS7/DAB2IP/DVL1/PRKACB/DVL3/KAT2A/TGFB2/EDA/MIB1/IFT172/IFT52/SETD2/PRICKLE1/TCTN1/ABL1/EDAR/BMP4/IRX2/BCAS3/LMO4/SOX4/STK3/NOG/DVL2/SOX8/FZD3/TWIST1/EGF/TULP3/LRP6/ARHGAP35/PODXL/FGFR2/GREM1/SEC24B/GDF7/NPHP3/SKI/ATOH8/IFT122/SDC4/BMP7/OSR1/PTCH1/TSC1/IRX1/DLC1/COBL/GATA3 |
| GOCC | MITOCHONDRIAL RESPIRATORY CHAIN COMPLEX IV | 12 | 0.703242504 | 1.782662382 | 0.003855108 | 0.022249668 | 0.015541753 | 3812 | tags=67%, list=18%, signal=55% | UQCRFS1/COX5A/C15orf48/NDUFA4/COX6A1/UQCRC2/COX7C/COX4I1 |
| GOBP | REGULATION OF TUMOR NECROSIS FACTOR MEDIATED SIGNALING PATHWAY | 57 | 0.457748802 | 1.661559653 | 0.003848198 | 0.022228887 | 0.015527237 | 4189 | tags=44%, list=20%, signal=35% | CASP4/PYCARD/CASP1/GSTP1/PTPN2/SPPL2A/ADAM17/BIRC3/SYK/TRAF1/IKBKG/PYDC1/CARD16/CASP8/LAPTM5/TNFRSF1A/TNF/CHUK/TRADD/CPNE1/SPHK1/OTULIN/TRAF2/NLRP2B/RBCK1 |
| GOBP | PROTEIN LOCALIZATION TO CELL PERIPHERY | 323 | -0.270488676 | -1.358143103 | 0.003806717 | 0.022008194 | 0.01537308 | 4823 | tags=35%, list=23%, signal=27% | GOLPH3L/CPLX1/PKDCC/RILPL2/FLOT1/PDPK1/ABCA2/NUMB/LRP1/PDZK1/STAC2/ZDHHC8/ARHGAP44/TMBIM1/ACSL3/ZDHHC7/BBIP1/PTPN9/GPHN/MRAP/EFR3A/TNIK/APPL1/SMURF1/SEC16A/ZDHHC3/RAMP2/GRIP1/GPER1/SQSTM1/NFASC/VAMP2/RDX/C2CD5/PRKCE/NECTIN3/RAP1A/LIN7A/RABEP1/KRT18/ZDHHC2/GGA3/DLG1/PREPL/LGI1/EFCAB7/DAB2/EHD2/TESC/PACS2/GOPC/GOLGA4/S100A10/PRKG2/MACF1/ADIPOQ/RAB26/CLSTN1/RAB40B/TSPAN14/CACNB2/RHOQ/FGF13/GGA2/EGFR/EPB41L2/GPC4/ANK2/TUB/LRP6/CD81/CLIP3/ITGB1/NUMA1/ARL3/GPC6/GAS6/EPB41/RAB11FIP2/LIN7B/STX8/RAB3B/BBS1/DAG1/GOLGA7/ROCK2/OPTN/ATP2B4/STAC/STX7/LZTFL1/RAPGEF2/PTCH1/SCN3B/PID1/DCHS1/ARL6IP5/WDR19/RILPL1/PALM/RAB40C/LAMA5/KIF13A/GNAI1/MYADM/PIK3R1/BBS2/AR/LGALS3/CAV1/SORBS1/DENND4C/SPTBN1 |
| GOBP | INTRACILIARY TRANSPORT | 52 | -0.436475689 | -1.663133638 | 0.003795257 | 0.021960838 | 0.015340001 | 5964 | tags=67%, list=29%, signal=48% | TTC21A/TNPO1/TTC30B/LCA5L/KIF3C/IFT88/RPGR/IFT81/TRIP11/IFT140/KIF3A/BBS12/KIF17/IFT74/DYNC2LI1/IFT27/TTC30A/IFT172/IFT52/LCA5/TTC21B/DYNC2H1/DYNC2I1/TRAF3IP1/DYNLL2/IFT22/TUB/CLUAP1/ARL3/DYNLRB2/PCM1/IFT122/WDR35/WDR19/DYNLRB1 |
| GOBP | RESPONSE TO INTERLEUKIN 4 | 34 | 0.519798378 | 1.70640008 | 0.003770984 | 0.021839196 | 0.015255031 | 4232 | tags=59%, list=20%, signal=47% | IL4R/HSPA5/MCM2/PTPN2/PARP14/ARG1/JAK3/CORO1A/IL24/IL2RG/SHPK/KEAP1/LEF1/PML/XCL1/NFIL3/RPLP0/CDK4/XBP1/CD300LF |
| GOBP | POSITIVE REGULATION OF MITOCHONDRIAL TRANSLATION | 16 | 0.658246082 | 1.822748435 | 0.003767949 | 0.021839196 | 0.015255031 | 4523 | tags=75%, list=22%, signal=59% | C1QBP/FASTKD2/TRUB2/TRMT10C/COA3/UQCC2/MPV17L2/MRPS27/RCC1L/FASTKD3/UQCC1/RPUSD3 |
| GOBP | CELLULAR RESPONSE TO CHEMICAL STRESS | 329 | 0.306212669 | 1.395462108 | 0.003761767 | 0.021823444 | 0.015244028 | 3348 | tags=28%, list=16%, signal=24% | GJB2/ZC3H12A/CYCS/MELK/PPIF/SLC7A11/GCH1/GPX2/PCNA/SRXN1/GSTP1/VRK2/HIF1A/EZH2/MAPK13/NCOA7/CCNA2/EIF2S1/MMP9/SMPD3/PPARGC1B/CFLAR/VNN1/PPIA/ERO1A/ATP2A2/ADPRS/TXNRD1/ECT2/PNPT1/ARG1/MEAK7/PRDX1/TRAP1/LRRC8D/CHCHD2/SLC2A1/RELB/ATG5/P4HB/PDCD10/ROMO1/DNMT3B/HMOX1/XRCC6/FANCD2/GPX1/PDE8A/SOD2/IL18RAP/NR4A3/IL6/ENDOG/RIPK3/SESN2/CD36/XRCC5/GLRX2/GSKIP/ATG7/DHFR/OXSR1/NCF2/BDKRB2/BNIP3/ATF4/CAB39/TXN/PRDX5/PARK7/CYBA/GPR37/KEAP1/LONP1/HDAC2/DIABLO/CDK2/MAP3K5/TMEM161A/PML/FXN/PARP1/PRDX6/G6PD/HTRA2/CHUK/ANXA1/TSPO/SIGMAR1/CASP3/STX4/PRKCD |
| GOBP | REGULATION OF ANION TRANSMEMBRANE TRANSPORT | 104 | -0.351817901 | -1.532076545 | 0.003702031 | 0.02149546 | 0.015014926 | 3499 | tags=34%, list=17%, signal=28% | SLC43A1/C2CD5/RAP1A/ACSL1/C3/TCAF1/CLTRN/ENPP1/GOPC/RGS2/INSR/LEP/EDN1/ADIPOQ/RHOQ/PRKAG2/PER2/CLIP3/ITGB1/SLC25A27/GPC3/OSBPL8/PRKCB/BRAF/PID1/KLF15/ARL6IP5/ATP1A2/IRS1/PIK3R1/AZIN1/CREBL2/SORBS1/IRS2/MEF2A |
| GOMF | MISFOLDED PROTEIN BINDING | 27 | 0.566505785 | 1.760325932 | 0.00366594 | 0.021304312 | 0.014881406 | 2468 | tags=37%, list=12%, signal=33% | F12/HSPA5/DNAJB11/HSPA14/HSPA9/HSPA8/EDEM1/TOR1A/DERL2/SDF2L1 |
| GOBP | EPITHELIAL CELL DEVELOPMENT | 204 | -0.295952588 | -1.393531442 | 0.00366401 | 0.021304312 | 0.014881406 | 3952 | tags=47%, list=19%, signal=39% | FRS2/LRTOMT/RARB/HOXA13/NKX3-2/RHEB/EZR/SHROOM3/NOTCH4/INSM1/FLNA/SOX18/HEG1/COL18A1/PDE4D/TPRN/DLL1/HOXA5/S1PR3/SPDEF/FOXA1/CDH5/RILPL2/PDPK1/FASN/CDK6/PDGFB/PLOD3/POF1B/DACT2/YIPF6/ADAMTSL4/BMP6/IFT74/RAP1B/TRIOBP/GSTM3/ARID4A/FOXC2/RDX/WNT5B/RAP1A/VCL/CLIC4/FEM1B/BMP4/WNT7B/TFCP2L1/ATRX/CLDN3/JAG1/BCL11B/ASXL1/PECAM1/YAP1/IL6ST/ADIPOQ/PLCB1/SOX8/ARNTL/RAPGEF3/ADD1/MET/ESR1/RFX3/ROBO4/CLDN5/KRT2/PGR/CLOCK/PTPRS/PODXL/SEC24B/TNMD/LAMB2/GSK3B/IQGAP1/ROCK2/PDE2A/ACTA2/ABI2/WDPCP/RAPGEF2/VIM/RILPL1/EXPH5/MAGI2/MYADM/SLITRK6/AR/TMOD1/NOTCH2/SOD1/VEZF1/ARHGEF26/CLDN1 |
| GOBP | REGULATION OF T CELL MIGRATION | 42 | 0.505525836 | 1.725557886 | 0.003662514 | 0.021304312 | 0.014881406 | 3616 | tags=45%, list=17%, signal=37% | C10orf99/WNT5A/CCL20/S100A7/PYCARD/CXCL13/CXCL10/ADAM17/ITGA4/RIPK3/DOCK8/OXSR1/ADAM10/ADAM8/FADD/CCL5/XCL1/CCR2/TMEM102 |
| GOMF | STRUCTURAL CONSTITUENT OF MUSCLE | 42 | -0.478361368 | -1.750962531 | 0.003660423 | 0.021304312 | 0.014881406 | 4245 | tags=43%, list=20%, signal=34% | MYOM1/PLEC/CAPN3/KRT19/MYOM2/MYL9/MYOT/ASPH/NEXN/SORBS2/DAG1/SYNM/TPM2/CSRP1/NEBL/DMD/MYH11/TPM1 |
| GOBP | TRANSCRIPTION ELONGATION FROM RNA POLYMERASE II PROMOTER | 84 | 0.408879633 | 1.579516624 | 0.003647838 | 0.021272722 | 0.01485934 | 4252 | tags=39%, list=20%, signal=31% | POLR2H/EZH2/ELL2/NCBP1/POLR2F/ELOC/ELOB/NELFCD/POLR2L/ELOF1/LEO1/RNF168/ADRM1/POLR2G/POLR2D/ELL/EAF1/CDK7/IWS1/PAF1/POLR2E/CCNH/RNF8/NELFE/CDK9/CTR9/POLR2I/SSRP1/SUPT4H1/ENY2/CCNK/TCERG1/POLR2J |
| GOBP | GLYCOLYTIC PROCESS THROUGH FRUCTOSE 6 PHOSPHATE | 29 | 0.555964473 | 1.744634294 | 0.003647425 | 0.021272722 | 0.01485934 | 3165 | tags=45%, list=15%, signal=38% | ENO1/TPI1/PGAM1/PGK1/HK2/ALDOA/FOXK2/GAPDH/GPI/GALK1/PFKP/PKM/ADPGK |
| GOBP | RAS PROTEIN SIGNAL TRANSDUCTION | 331 | -0.261686353 | -1.313474253 | 0.003647274 | 0.021272722 | 0.01485934 | 3722 | tags=28%, list=18%, signal=23% | PLD1/SQSTM1/LPAR4/ALS2/SOS1/ARHGEF2/RALGDS/MADD/RDX/NRP1/PPP2CB/RAP1A/IQSEC1/GNA12/GNA13/RIPOR1/RABL3/RAB30/ABL1/DENND3/RASA4/SHC3/PDGFRB/KITLG/SHOC2/WASF2/PRKD1/ARHGEF1/GNB1/ARHGAP24/RAB33B/RTN4R/RASA3/KSR1/CYTH3/NET1/ERBIN/HACD3/BRAP/MRAS/F2R/ZNF304/FBXO8/AUTS2/RAPGEF3/MET/GARRE1/SHC2/RRAS/APOE/OGT/ARHGAP35/ITPKB/PIK3CB/RERG/ARHGAP42/PLEKHG5/RHOB/STMN3/SCAI/NISCH/NTN1/RHOJ/CTNNAL1/STARD13/AKAP13/ROCK2/ARHGAP6/ADRA1A/ARHGAP29/ABI2/RAB9B/ARHGEF12/TIMP2/RAPGEF2/MYOC/RALGPS1/BCL6/LPAR1/EPS8/ADRA2A/AGTR1/ARFGEF3/SPRY1/DLC1/NOTCH2/SPRY2/ARHGEF28/ARHGEF10/KANK1/IRS2/KANK2/DENND4C |
| GOBP | LOOP OF HENLE DEVELOPMENT | 11 | -0.71367166 | -1.826350658 | 0.003646928 | 0.021272722 | 0.01485934 | 3051 | tags=82%, list=15%, signal=70% | DLL1/IRX3/WNT7B/IRX2/JAG1/POU3F3/PKD2/PKD1/IRX1 |
| GOBP | REGULATION OF HEMOPOIESIS | 390 | 0.290192476 | 1.342473645 | 0.003642421 | 0.021272722 | 0.01485934 | 4929 | tags=36%, list=24%, signal=28% | ZC3H12A/NFKBIZ/IRF7/STAT1/PNP/LTF/GPR68/PRELID1/IL4R/NME1/PRKCQ/HDAC1/HMGB3/PLA2G3/ISG15/CD83/HIF1A/EIF6/PPARGC1B/VNN1/IL20/PTPN2/IRF1/PRDM1/HSPA9/STAT3/CD2/SASH3/NCAPG2/CEBPB/RHOH/AP3D1/JAK3/IFNG/SMAP1/MYC/LYN/SOCS1/HAX1/CBFB/SLAMF8/IL12B/XRCC6/FANCD2/ZC3H8/SYK/RCOR1/IKZF3/IL7R/NR4A3/PRMT6/LGALS9/CCL19/CD27/AP3B1/CIB1/IL23A/LEO1/PITHD1/ZFP36/LAG3/PRKDC/DPY30/IL2RA/PGLYRP2/GPR137B/CASP8/ADAM8/FADD/LILRB4/TMEM64/TNFSF9/HMGB2/CD86/FAXDC2/CRTAM/TMEM131L/H3C10/GABPA/GPR171/HCLS1/PAF1/TNF/LEF1/IL36B/NFKBID/TREM2/RB1/IL1RL2/PGLYRP3/CCR1/PRMT1/ANXA1/HLA-DRA/IL12RB1/TYROBP/CCR2/CTR9/LILRB2/B2M/FOXN1/IL15/TMEM176B/PURB/CARD11/HLA-B/ZAP70/LILRB1/C1QC/NLRP3/HLA-G/MTOR/CD80/IL10/FOXP3/MAPK14/XBP1/ZNF683/CSF3R/H3C7/CR1/PTPRC/CD28/MALT1/FANCA/H3C8/H4C4/HLA-DOA/TRIB1/H4C9/NKAP/AGER/INHA/PPP2R3C/FSHB/TMEM176A/TBX21/POU4F2/RASSF2/IFNA2/CAMK4/MIR17HG |
| GOCC | CATALYTIC STEP 2 SPLICEOSOME | 85 | 0.408973408 | 1.583489416 | 0.0036369 | 0.021272722 | 0.01485934 | 3696 | tags=35%, list=18%, signal=29% | EIF4A3/PPIL1/SNRPG/SNRPF/SNRPD1/HNRNPA2B1/HNRNPC/DDX41/LSM2/BUD31/SF3B3/LSM7/SNRPD3/EFTUD2/ALYREF/SNRPA1/MAGOHB/SF3B6/SNRPB/AQR/SRSF1/HNRNPF/HNRNPM/ISY1/PLRG1/PRPF4B/PNN/MAGOH/SNRNP40/TFIP11 |
| GOBP | PROTEIN COMPLEX OLIGOMERIZATION | 223 | 0.331852246 | 1.462940406 | 0.003620898 | 0.021226169 | 0.014826822 | 4230 | tags=28%, list=20%, signal=23% | ZC3H12A/TK1/PYCARD/HOMER1/KCTD5/ALDH1A3/TNFAIP1/STOML2/SHMT2/GBP5/MLKL/ECT2/PNPT1/HPRT1/ALDOA/CPSF6/PRF1/FARSA/MIF/HSD17B10/OSBPL2/TDO2/KCTD11/SOD2/KCTD1/RRM1/BLM/STEAP4/EHD4/FARSB/POLQ/KCNJ2/ELAVL1/JMJD6/GSDMD/TWNK/ACOT13/BOK/TP63/KCTD21/PDCD6IP/ACACA/KCTD4/KCNS3/KCND3/PRMT1/SIGMAR1/RNF213/BEND3/NLRC4/SHKBP1/B2M/ARC/SAMHD1/ROM1/ALOX5AP/HLA-G/KCNC3/CHMP2A/THG1L/SLC1A5/VASP/KCNG1 |
| GOBP | MITOTIC SPINDLE ASSEMBLY | 63 | 0.43950812 | 1.628302942 | 0.003613885 | 0.02120356 | 0.01481103 | 2985 | tags=35%, list=14%, signal=30% | CDC20/AURKB/NEK2/PRC1/KIF4A/TPX2/RAB11A/KIF2A/KIF11/KIF23/RACGAP1/ZNF207/MYBL2/PLK1/BCCIP/RCC1/CHEK2/KPNB1/KIFC1/DRG1/CHMP5/CHMP1B |
| GOBP | MITOTIC G1 S TRANSITION CHECKPOINT | 62 | 0.446993045 | 1.647197893 | 0.003601444 | 0.021149041 | 0.014772947 | 3325 | tags=40%, list=16%, signal=34% | CCNB1/CDK1/TFDP1/PCNA/AURKA/PLK2/SFN/E2F8/CNOT11/CDKN1A/PLK3/CNOT1/PRKDC/CHEK2/TRIAP1/CDK2/CRADD/CDC25C/PML/BAX/GADD45A/PRMT1/MDM2/RFWD3/CNOT6 |
| GOBP | LOCOMOTORY BEHAVIOR | 156 | -0.313113883 | -1.433201826 | 0.003591647 | 0.02110996 | 0.014745649 | 3817 | tags=28%, list=18%, signal=23% | HEXB/PREX2/NCOA2/PRKN/PTEN/PRKCE/EFNB3/HOXD9/NCOR1/APP/CXCL12/KCND2/LRRK2/CIART/SNCG/NTF4/LGI4/EN1/PPP1R1B/PPP3CB/APOE/GLRB/MECP2/OXR1/SOBP/NEGR1/NPY1R/DDHD2/FZD4/PAFAH1B1/TSC1/ATP1A2/MCOLN3/APH1B/NOVA1/ZFHX3/PUM1/HPGDS/SLITRK6/SLC18A2/ANKH/TMOD1/MEIS1/SOD1 |
| GOBP | MONOCYTE CHEMOTAXIS | 58 | 0.453878902 | 1.645903087 | 0.003586888 | 0.021100451 | 0.014739006 | 3579 | tags=41%, list=17%, signal=34% | S100A12/CCL20/S100A7/CXCL10/CCL18/CCL4/CCL22/S100A14/CCL2/LYN/SLAMF8/DEFB124/CCL8/IL6/CCL7/CCL19/CXCL17/CCL5/XCL1/CCR1/ANXA1/CCR2/CCL17/LGMN |
| GOBP | POSITIVE REGULATION OF INFLAMMATORY RESPONSE | 119 | 0.384161705 | 1.571533685 | 0.00358493 | 0.021100451 | 0.014739006 | 2901 | tags=29%, list=14%, signal=25% | S100A12/S100A9/NFKBIZ/NMI/WNT5A/S100A8/CTSC/ABCC1/CCR7/PLA2G3/OSMR/MAPK13/IDO1/TLR2/IFI35/CEBPB/IFNG/IL1B/IL12B/IL6/IL23A/GRN/FFAR2/VAMP8/ADAM8/TNIP1/HLA-E/VAMP7/PTGER3/TSLP/PARK7/TNFRSF1A/MGST2/TNF/TREM2 |
| GOBP | RESPONSE TO ENDOPLASMIC RETICULUM STRESS | 287 | 0.314543803 | 1.420606679 | 0.003546432 | 0.02089906 | 0.014598331 | 4964 | tags=36%, list=24%, signal=27% | MANF/CHAC1/CASP4/NRBF2/VCP/ATP6V0D1/ERP44/BAK1/TMEM117/EIF2S1/HSPA5/FBXO6/HM13/SERP1/ERO1A/ATP2A2/PTPN2/CXCL8/DNAJB11/UFD1/AIFM1/SEC61B/CEBPB/BCAP31/YIF1A/CCL2/FOXRED2/P4HB/COPS5/TMCO1/SRPRB/UBXN8/HDGF/SHC1/TMX1/HYOU1/EDEM1/SESN2/PREB/TOR1A/DERL2/PDIA4/PSMC6/AUP1/LPCAT3/DDRGK1/TNFRSF10B/PDIA6/ATF4/BOK/SDF2L1/TARDBP/USP14/PARK7/PIK3R2/EDEM2/GET3/UBE2J2/CANX/EDEM3/MAP3K5/PML/BAX/PPP1R15B/WIPI1/EIF4G1/SSR1/UBAC2/MYDGF/TRIM25/ASNS/EIF2B5/TMEM129/NPLOC4/EXTL3/PPP2R5B/STT3B/GSK3A/YOD1/SGTA/TMEM33/XBP1/ERLIN1/TRAF2/UBE2J1/DNAJB12/ERP27/NCK2/ABCA7/UBA5/ACADVL/TPP1/DNAJB9/PMAIP1/PDIA3/IGFBP1/HSP90B1/SELENOS/TRIB3/TTC23L/ANKS4B/APAF1 |
| GOBP | RNA PHOSPHODIESTER BOND HYDROLYSIS EXONUCLEOLYTIC | 43 | 0.494597029 | 1.69666122 | 0.003539715 | 0.020877792 | 0.014583475 | 3878 | tags=49%, list=19%, signal=40% | ZC3H12A/ISG20/EXOSC4/PNPT1/EXOSC3/ISG20L2/DIS3/PDE12/EXOSC1/CNOT1/EXOSC2/ERI1/ERI2/USB1/EXOSC5/XRN2/NOCT/CNOT6/EXOSC8/TOE1/DCPS |
| GOCC | OUTER MEMBRANE | 216 | 0.330733961 | 1.452192299 | 0.003537621 | 0.020877792 | 0.014583475 | 4460 | tags=37%, list=21%, signal=29% | TRIM14/IFI27/HK2/NLRX1/NME1/TIGAR/BCL2A1/MFN1/GK/ATP5MC3/TOMM22/BAK1/PHB/RSAD2/STMP1/ABCB6/TOMM5/MTX2/HSPA9/MARCHF5/TOMM40L/MICOS10/ARG1/SLC25A46/CYP27B1/VDAC2/TOMM40/CNP/PGAM5/COASY/BID/MICOS13/HAX1/CYP24A1/MTX1/CHCHD3/IMMT/RTN4IP1/GDAP1/CASP8/VDAC1/BNIP3/BOK/TOMM34/AGPAT5/CISD1/MFF/VDAC3/CPTP/BAX/CPT1C/MIEF1/TSPO/SIGMAR1/AGK/FUNDC2/MYO19/ITPRIP/PHB2/APOO/STARD7/DNAJC11/NUTF2/MCL1/TRAF3IP3/GJA1/EMD/PLA2G2A/LRPPRC/MTOR/THG1L/SAMM50/NAV3/QTRT2/GK2/CISD2/CYB5B/MIGA2/MTERF3 |
| GOBP | REGULATION OF ANTIGEN PROCESSING AND PRESENTATION | 20 | 0.613366418 | 1.772754008 | 0.003525673 | 0.020831547 | 0.014551172 | 3632 | tags=50%, list=17%, signal=41% | NOD2/PYCARD/CCR7/CCL19/HLA-DOB/TAPBPL/TREM2/LILRB2/CD68/WAS |
| GOBP | REGULATION OF SMOOTH MUSCLE CELL DIFFERENTIATION | 30 | -0.519827569 | -1.761877466 | 0.003519436 | 0.020813005 | 0.01453822 | 3985 | tags=53%, list=19%, signal=43% | ANKRD17/GPER1/EFEMP2/SIRT1/BMP4/FGF9/NFATC2/FGFR2/MECP2/MYOCD/RBPMS2/PDCD4/NFATC1/PRDM6/PIAS1/KIT |
| GOBP | MACROAUTOPHAGY | 301 | 0.309246406 | 1.40440387 | 0.003512373 | 0.020789536 | 0.014521827 | 3946 | tags=32%, list=19%, signal=26% | NOD2/TIGAR/MFN1/VCP/ATP6V0D1/TOMM22/SPTLC2/HIF1A/ATP6V1B2/ATP6V1G1/ATP6V0B/VPS25/RALB/TOMM5/GAPDH/LAMTOR5/LAMTOR2/ATG2A/TOMM40/SNAP29/EPG5/RAB5A/ATG13/BAG3/RAB1A/PGAM5/ATG5/CDK5/SNX6/EMC6/HMOX1/STAM/CAPN1/VTA1/TBK1/CSNK2A1/ATP6V1D/DYNLL1/SESN2/PHF23/ATP6V1C1/POLDIP2/ATP6V1C2/GNAI3/PIP4K2C/ATG7/ATG4B/PLAA/NPC1/ATG3/IKBKG/AUP1/VAMP8/VDAC1/VPS37A/ATP6V1H/ATP6V1A/DDRGK1/BNIP3/SNF8/QSOX1/SNAPIN/RRAGC/NSFL1C/RUBCNL/LARP1/ATP6V0E1/CDC37/VPS37B/SLC38A9/WIPI1/MLST8/HTRA2/UBQLN4/CASP3/GPSM1/LAMTOR3/CHMP6/MTMR14/PHB2/TEX264/PRKAG1/SPTLC1/VPS16/EI24/HTT/BECN1/MAPK8/MVB12A/ATP6V0A2/SCFD1/YOD1/SNX5/MTOR/TMEM39B/CHMP2A |
| GOBP | SNRNA METABOLIC PROCESS | 37 | 0.517195092 | 1.721494651 | 0.003500114 | 0.020735243 | 0.014483902 | 3570 | tags=41%, list=17%, signal=34% | EXOSC4/NHP2/NOP10/EXOSC3/EXOSC2/USB1/INTS7/DKC1/EXOSC5/INTS5/METTL4/INTS9/INTS14/EXOSC8/TOE1 |
| GOBP | POSITIVE REGULATION OF HEMOPOIESIS | 149 | 0.362242229 | 1.528706411 | 0.003478597 | 0.020625963 | 0.014407569 | 4704 | tags=42%, list=23%, signal=33% | NFKBIZ/PNP/GPR68/IL4R/PLA2G3/CD83/PPARGC1B/VNN1/IL20/SASH3/RHOH/AP3D1/IFNG/SOCS1/HAX1/CBFB/IL12B/XRCC6/SYK/IL7R/LGALS9/CCL19/CD27/AP3B1/IL23A/PRKDC/IL2RA/CASP8/ADAM8/FADD/LILRB4/TMEM64/TNFSF9/CD86/HCLS1/TNF/LEF1/IL36B/NFKBID/TREM2/RB1/IL1RL2/CCR1/ANXA1/HLA-DRA/IL12RB1/TYROBP/LILRB2/IL15/ZAP70/NLRP3/HLA-G/CD80/IL10/FOXP3/XBP1/CR1/PTPRC/MALT1/TRIB1/NKAP/AGER/PPP2R3C |
| GOBP | KILLING OF CELLS IN OTHER ORGANISM INVOLVED IN SYMBIOTIC INTERACTION | 22 | 0.584965385 | 1.731626034 | 0.00345622 | 0.020511385 | 0.014327534 | 2324 | tags=36%, list=11%, signal=32% | KRT6A/APOL1/TUSC2/ARG1/GAPDH/ROMO1/CXCL6/CAMP |
| GOBP | REGULATION OF CELLULAR RESPIRATION | 22 | 0.585245744 | 1.732455959 | 0.00345622 | 0.020511385 | 0.014327534 | 3354 | tags=55%, list=16%, signal=46% | PRELID1/VCP/HIF1A/SHMT2/PNPT1/IFNG/TRAP1/NOS2/BNIP3/CISD1/IDE/OPN3 |
| GOBP | NECROPTOTIC PROCESS | 35 | 0.517276307 | 1.713050989 | 0.003429205 | 0.020387076 | 0.014240702 | 3206 | tags=40%, list=15%, signal=34% | PPIF/PYGL/CFLAR/MLKL/PGAM5/BIRC3/RIPK3/CASP8/FADD/BOK/LY96/TNF/FASLG/PELI1 |
| GOMF | 3 5 DNA HELICASE ACTIVITY | 17 | 0.635080714 | 1.772043424 | 0.003428025 | 0.020387076 | 0.014240702 | 1757 | tags=35%, list=8%, signal=32% | ASCC3/MCM6/MCM5/MCM2/MCM7/BLM |
| GOBP | POSITIVE REGULATION OF CHROMOSOME SEPARATION | 17 | 0.635774648 | 1.773979683 | 0.003428025 | 0.020387076 | 0.014240702 | 3150 | tags=53%, list=15%, signal=45% | DLGAP5/PLSCR1/ANAPC7/CDT1/ESPL1/MAD2L1BP/ANAPC11/RB1/CDC23 |
| GOBP | REGULATION OF CELLULAR AMIDE METABOLIC PROCESS | 397 | 0.291520025 | 1.349904601 | 0.00339022 | 0.020208963 | 0.014116287 | 3964 | tags=31%, list=19%, signal=25% | ZC3H12A/GSPT1/PGAM1/NOD2/EIF4A3/EIF4EBP1/GZMB/SLC7A11/C1QBP/EIF2B2/TSFM/TIGAR/EIF4E2/MRPL13/PTAFR/EIF4E/TYMS/ORMDL2/EIF6/KRT17/METAP1/EIF2S1/SMPD3/SHMT2/SELENOT/NCBP1/BCL3/IFNGR1/TACO1/STAT3/CDC123/RPS6KB2/TARBP2/FASTKD2/TRUB2/PYM1/IFNG/GAPDH/TRAP1/ABCG1/PPP1CA/DPH2/PUM3/CNOT11/MTG1/RIDA/TRMT10C/COA3/KBTBD8/EIF3K/RPS6KA1/UQCC2/NOLC1/PA2G4/IL6/ZC3H12D/SESN2/SHFL/SARNP/MTIF2/CNOT1/MPV17L2/DHFR/ZFP36/POLR2G/PRKDC/GSTZ1/EIF3B/NPM1/ELAVL1/POLR2D/JMJD4/EIF2B1/EIF4H/SMPD2/ATF4/TARDBP/TRNAU1AP/PUS7/SAMD8/EIF4G2/PASK/TNFRSF1A/CYFIP1/MRPS27/CAPRIN1/EIF2B4/LARP1/RCC1L/ZNF598/IGF2BP3/CCL5/NAT10/TNF/GRB7/PKM/YBX1/PPP1R15B/EIF4G1/EPRS1/CASP3/GLE1/EIF5/MAGOH/CNOT6/PRKCD/UHMK1/YTHDF2/EIF2B5/DAZL/MTG2/EIF5A/FASTKD3/CDK4/DHX29/UPF1/GSK3A/DIO2/SPHK1/LRPPRC/MTOR/UQCC1 |
| GOBP | COLLAGEN FIBRIL ORGANIZATION | 51 | -0.443357313 | -1.685063172 | 0.003386096 | 0.020202304 | 0.014111636 | 6090 | tags=53%, list=29%, signal=38% | FMOD/ADAMTS3/P3H4/COL5A1/SFRP2/COL1A2/COL5A3/LOXL1/LOXL3/NF1/ATP7A/PLOD3/TGFB2/EFEMP2/FOXC2/CYP1B1/LOX/AEBP1/DDR2/CHADL/COL12A1/DPT/FOXC1/GREM1/TNXB/CRTAP/LOXL4 |
| GOBP | REGULATION OF T HELPER 17 TYPE IMMUNE RESPONSE | 21 | 0.609137585 | 1.77529008 | 0.003375632 | 0.020157772 | 0.01408053 | 4814 | tags=52%, list=23%, signal=40% | ZC3H12A/NFKBIZ/PRKCQ/IL12B/IL23A/NFKBID/IL12RB1/FOXP3/NLRP10/MALT1/TBX21 |
| GOBP | AORTIC VALVE MORPHOGENESIS | 31 | -0.523940033 | -1.783075574 | 0.003344253 | 0.019988161 | 0.013962053 | 3784 | tags=35%, list=18%, signal=29% | ELN/BMP4/ROBO2/JAG1/SLIT2/EFNA1/TWIST1/SLIT3/ROCK2/NFATC1/GATA3 |
| GOBP | RETROGRADE VESICLE MEDIATED TRANSPORT GOLGI TO ENDOPLASMIC RETICULUM | 82 | 0.413143522 | 1.588334833 | 0.003338795 | 0.019973311 | 0.01395168 | 4662 | tags=43%, list=22%, signal=33% | RER1/KIF2C/KIF4A/KIF2A/KIF11/COPB2/CENPE/KIF23/RACGAP1/LMAN2/RAB1A/KIF15/KIF18A/KDELR2/NSF/ARF3/SURF4/NAPA/COPE/PITPNB/KIF22/TAPBP/COG4/RAB6A/TMED7/HTT/ARF5/SCFD1/TMED9/KIFAP3/RINT1/BNIP1/ARF4/DNAJC28/STX18 |
| GOBP | NEGATIVE REGULATION OF LEUKOCYTE PROLIFERATION | 83 | 0.416697797 | 1.60553759 | 0.003338795 | 0.019973311 | 0.01395168 | 4262 | tags=41%, list=20%, signal=33% | CD274/TNFRSF21/GSTP1/IDO1/PLA2G2F/ARG1/CEBPB/LYN/CCL8/GLMN/LGALS9/IL2RA/LILRB4/CD86/CRTAM/TMEM131L/SCRIB/SFTPD/XCL1/CLEC4G/PELI1/CASP3/TYROBP/TNFRSF13B/LILRB2/MNDA/LILRB1/HLA-G/CD300A/CD80/IL10/FOXP3/LST1/CR1 |
| GOBP | NEGATIVE REGULATION OF INTERLEUKIN 1 PRODUCTION | 32 | 0.518317815 | 1.673250913 | 0.003324494 | 0.019923237 | 0.013916703 | 3970 | tags=41%, list=19%, signal=33% | ZC3H12A/SERPINB1/GSTP1/CARD16/LILRB4/TREM2/PML/CPTP/NLRP7/CARD18/ERRFI1/NLRP3/IL10 |
| GOBP | METANEPHRIC GLOMERULUS DEVELOPMENT | 15 | -0.644063213 | -1.813210953 | 0.003294962 | 0.019763889 | 0.013805396 | 3438 | tags=53%, list=16%, signal=45% | PDGFB/LGR4/PDGFRB/ADIPOQ/LAMB2/CD34/OSR1/PDGFRA |
| GOBP | ELASTIC FIBER ASSEMBLY | 10 | -0.725757014 | -1.817690819 | 0.003252989 | 0.01952956 | 0.013641713 | 4774 | tags=70%, list=23%, signal=54% | ATP7A/EFEMP2/LOX/MFAP4/TNXB/FBLN5/MYH11 |
| GOBP | PROTEIN AUTOPROCESSING | 24 | 0.588920666 | 1.774557708 | 0.003235805 | 0.019443771 | 0.013581789 | 5027 | tags=54%, list=24%, signal=41% | KLK6/CASP4/F12/CASP1/PCSK9/CTSL/FXN/PARP1/HTRA2/MYRF/SPRTN/AFG3L2/PIDD1 |
| GOBP | SKELETAL MUSCLE ORGAN DEVELOPMENT | 152 | -0.318711827 | -1.453611084 | 0.003213969 | 0.019329853 | 0.013502215 | 4329 | tags=36%, list=21%, signal=29% | CCNT2/DLL1/EP300/ATF3/RBM24/TBX1/FLOT1/CASQ1/HDAC4/NR1D2/MYOM1/VGLL2/DNER/HDAC9/EGR2/ELN/MSTN/HOXD9/MYMX/KCNAB1/ZBTB18/MYOM2/BVES/SHOX2/GPC1/HLF/SOX8/FOS/ARNTL/BCL9/TWIST1/ACTA1/LARGE1/DDX17/POPDC2/NR2F2/MEF2C/MYOCD/MEF2D/NLN/DAG1/SKI/SVIL/MEOX2/MYH14/CFL2/DCN/EPHB1/FXR1/CYP26B1/NUPR1/CDON/CAV2/BCL2/CAV1 |
| GOBP | POSITIVE REGULATION OF CYTOKINE PRODUCTION INVOLVED IN INFLAMMATORY RESPONSE | 20 | 0.616232432 | 1.781037374 | 0.003208053 | 0.019311562 | 0.013489438 | 4090 | tags=55%, list=20%, signal=44% | NOD2/CLEC7A/MYD88/PLA2G3/GBP5/STAT3/CD6/IL6/TNF/IL17F/GPSM3 |
| GOBP | MALE SEX DIFFERENTIATION | 150 | -0.331787182 | -1.512065183 | 0.003173841 | 0.01912275 | 0.01335755 | 2992 | tags=25%, list=14%, signal=22% | PDGFRB/KITLG/ATRX/CSDE1/TESC/TBX3/SPATA2/MGST1/LHCGR/SOX8/WDR48/ESR1/FGF9/CTNNA1/KDM5A/AKR1C3/BMPR1A/ROR2/HOXA11/SMAD5/STAR/ZFPM2/HOXA10/STAT5B/PLEKHA1/NUPR1/AGO4/PDGFRA/ASB1/RNF38/AR/PATZ1/KIT/BCL2/RHOBTB3/WNT2B/CCND1/GATA3 |
| GOBP | POSITIVE REGULATION OF MITOCHONDRION ORGANIZATION | 83 | 0.417115793 | 1.60714813 | 0.003170457 | 0.019119506 | 0.013355284 | 3775 | tags=42%, list=18%, signal=35% | TFDP1/PPIF/GZMB/FAM162A/PYCARD/BAK1/HIF1A/NMT1/BIK/YWHAQ/MMP9/TNFSF10/SFN/MARCHF5/BID/PDCD5/PLAUR/CASP8/VDAC1/BNIP3/BOK/TP63/YWHAZ/PARK7/TREM2/MFF/BAX/HTRA2/MIEF1/ZDHHC6/HIP1R/CIDEB/HTT/MAPK8/GSK3A |
| GOCC | SPLICEOSOMAL COMPLEX | 181 | 0.344553776 | 1.479379726 | 0.003159049 | 0.019067824 | 0.013319184 | 4985 | tags=40%, list=24%, signal=31% | EIF4A3/PPIL1/SNRPG/SNRPF/SNRPD1/HNRNPA2B1/HNRNPC/DDX41/LSM2/BUD31/TXNL4A/SF3B3/LSM7/SNRPD3/EFTUD2/SNRNP25/SF3B5/SNU13/PPIH/ALYREF/SNRPA1/MAGOHB/SNRPC/HSPA8/SF3B6/LSM5/LSM4/SNRPB/AQR/ADAR/LUC7L2/PRPF31/PRPF38A/SRSF1/USP39/HNRNPF/LSM6/RBM28/HNRNPM/ISY1/PRPF4/PLRG1/TTF2/YBX1/PRPF4B/GEMIN2/SNRPA/PNN/MAGOH/SNRNP40/DHX32/TFIP11/DQX1/UPF1/TRA2B/PRPF38B/CWF19L1/SMNDC1/TXNL4B/PRPF18/CWC25/DHX15/HNRNPK/NCL/PRPF40A/LSM3/SNRPD2/YJU2/WDR83/IVNS1ABP/PRPF19/SYNCRIP |
| GOBP | AXON EXTENSION | 111 | -0.348223018 | -1.519136595 | 0.00315643 | 0.019067824 | 0.013319184 | 4893 | tags=41%, list=23%, signal=31% | GDI1/SEMA4C/SEMA3C/MAPT/SHTN1/LRP1/USP9X/SEMA3A/DVL1/RUFY3/SEMA6D/NRP1/VCL/ABL1/SIN3A/CXCL12/RTN4R/SEMA3E/GOLGA4/MACF1/L1CAM/SLIT2/SEMA4G/AUTS2/SEMA6A/SEMA3B/PPP3CB/NDN/RYK/SLIT3/APOE/PTPRS/DCLK1/ULK2/LAMB2/FN1/GSK3B/MAP2/NTN1/RTN4/DPYSL2/SEMA3G/DBN1/ALCAM/MAP1B |
| GOBP | PURINE NUCLEOSIDE MONOPHOSPHATE BIOSYNTHETIC PROCESS | 23 | 0.593882849 | 1.762664367 | 0.003113673 | 0.018827775 | 0.013151505 | 2322 | tags=39%, list=11%, signal=35% | GART/APRT/ATIC/IMPDH1/HPRT1/AMPD3/ADSL/ADK/GMPS |
| GOCC | TELOMERASE HOLOENZYME COMPLEX | 21 | 0.610830527 | 1.780224046 | 0.003087089 | 0.01868384 | 0.013050964 | 5343 | tags=67%, list=26%, signal=50% | NHP2/NOP10/HNRNPC/SNRPD3/SNRPB/GAR1/DKC1/NAT10/GNL3L/PTGES3/WRAP53/SMG5/TERT/SNRPE |
| GOBP | REGULATION OF IMMUNOGLOBULIN PRODUCTION | 66 | 0.444662156 | 1.655620914 | 0.003086289 | 0.01868384 | 0.013050964 | 6335 | tags=59%, list=30%, signal=41% | IL4R/SASH3/EXOSC3/CGAS/GPI/IL6/NSD2/MAD2L2/PRKDC/TFRC/HMCES/HLA-E/TNF/XCL1/STX4/TNFRSF4/MZB1/IL10/FOXP3/XBP1/PARP3/TRAF2/CR1/TNFSF13/PTPRC/CD28/IL33/CD40/TBX21/SLC15A4/MLH1/KMT5C/CLCF1/TP53BP1/PAXIP1/IL13/CD40LG/IL5/TLR9 |
| GOBP | PURINE NUCLEOBASE METABOLIC PROCESS | 18 | 0.642075602 | 1.820824027 | 0.003055424 | 0.018525579 | 0.012940416 | 4568 | tags=61%, list=22%, signal=48% | GDA/ACP3/GART/SHMT2/APRT/HPRT1/XDH/GMPS/KDM1A/GMPR2/PAICS |
| GOCC | RNA POLYMERASE III COMPLEX | 18 | 0.642425052 | 1.82181501 | 0.003055424 | 0.018525579 | 0.012940416 | 2784 | tags=50%, list=13%, signal=43% | POLR3G/POLR2H/POLR2F/POLR3K/POLR3B/POLR2L/POLR1C/POLR3D/POLR2E |
| GOBP | REGULATION OF ANATOMICAL STRUCTURE SIZE | 477 | -0.250024271 | -1.301801885 | 0.00304849 | 0.018516956 | 0.012934393 | 3784 | tags=27%, list=18%, signal=23% | ELN/GPER1/TRIOBP/DMTN/EDN3/PIK3CA/ALS2/SEMA6D/PTEN/FOXC2/RDX/NRP1/NOS1/SCPEP1/PRKCE/SLC12A2/HP1BP3/NPR1/PEX11A/DLG1/WASHC1/ABL1/SIN3A/ADCY6/DEPTOR/TENM1/AGT/WASF2/TMSB4X/CXCL12/LARS1/ECE1/ASXL1/RTN4R/LRRK2/RGS2/SEMA3E/GOLGA4/SWAP70/LEP/ATP2B1/EDN1/MACF1/L1CAM/SLIT2/ANO6/F2R/SPART/SEMA4G/DOCK5/DOCK4/SLC12A7/RAB22A/KANK4/ADD1/SEMA6A/ADRB1/FMN1/TMOD2/RB1CC1/WASF3/FGF13/SEMA3B/CLCN6/PER2/EGFR/FER/P2RX7/PLEKHH2/TMSB15B/SHANK3/RYK/DSTN/APOE/BIN1/VILL/PTPRS/FOXC1/ARHGAP35/PFN2/ARHGAP42/SPTAN1/FN1/KLF2/GSK3B/RICTOR/MAP2/SLC8A1/AKT3/P2RX1/RAB3B/NTN1/KCNMA1/ROCK2/ADRA1A/PRKG1/SCIN/VAV3/FHOD3/RTN4/SVIL/ACTA2/ABI2/CFL2/DPYSL2/PAK3/TSC1/ATP1A2/SEMA3G/AQP1/EPS8/ADRA2A/SPTBN2/AGTR1/MYADM/ADD3/LMOD1/DBN1/BBS2/TMOD1/GSN/CAV1/SOD1/KANK1/SPTBN1/KCNMB4/MAP1B/ADRB2/LAMTOR4 |
| GOMF | TRANSLATION INITIATION FACTOR ACTIVITY | 47 | 0.47987458 | 1.677611249 | 0.003047592 | 0.018516956 | 0.012934393 | 4031 | tags=51%, list=19%, signal=41% | EIF2B2/EIF4E2/MCTS1/EIF2S2/EIF4E/EIF6/EIF3J/EIF2S1/EIF3I/COPS5/DENR/EIF3K/MTIF2/EIF3B/EIF2B1/EIF4H/EIF4G2/EIF2B4/EIF4G1/EIF5/EIF2B5/EIF2B3/DHX29/EIF3A |
| GOBP | AROMATIC AMINO ACID FAMILY METABOLIC PROCESS | 32 | 0.520691322 | 1.68091315 | 0.003045329 | 0.018516956 | 0.012934393 | 2894 | tags=28%, list=14%, signal=24% | KYNU/IDO1/IL4I1/TDO2/HPDL/THAP4/GSTZ1/PARK7/GCDH |
| GOBP | REGULATION OF LYMPHOCYTE CHEMOTAXIS | 22 | 0.591521229 | 1.751032772 | 0.0030448 | 0.018516956 | 0.012934393 | 3616 | tags=64%, list=17%, signal=53% | WNT5A/S100A7/CXCL13/CXCL10/CCL4/ADAM17/CCL2/CCL7/OXSR1/ADAM10/CCL5/XCL1/CCR2/TMEM102 |
| GOCC | APICAL JUNCTION COMPLEX | 131 | -0.331008375 | -1.482366251 | 0.003041021 | 0.018516956 | 0.012934393 | 5848 | tags=49%, list=28%, signal=35% | ESAM/ANK3/CLDN16/SHROOM3/YBX3/FRMD4B/CLDN4/RHOA/IGSF5/LIN7C/NPHP1/STRN/ARHGAP17/CDH5/ASH1L/UBN1/SYNPO/POF1B/SHROOM4/CLDN15/MAGI3/APC/ARHGEF2/NECTIN3/LIN7A/CGN/PKN2/NPHP4/MXRA8/MPDZ/DLG1/CLDN3/JAM2/BVES/CLDN11/CYTH3/AMOT/AMOTL2/TJAP1/PARD6G/USP53/NHS/CLDN5/CTNNA1/EPCAM/AMOTL1/CLMP/LIN7B/CAMSAP3/FRMD4A/EPPK1/RAPGEF2/PARD3B/PARD3/MAGI1/MAGI2/CLDN8/EPB41L4B/JAM3/CGNL1/SORBS1/CLDN23/CCND1/CLDN1 |
| GOBP | ACTIVATION OF TRANSMEMBRANE RECEPTOR PROTEIN TYROSINE KINASE ACTIVITY | 12 | -0.699081774 | -1.852281294 | 0.003036205 | 0.018516956 | 0.012934393 | 3778 | tags=67%, list=18%, signal=55% | ANGPT1/NRG3/EFNA5/EGF/GREM1/PRLR/ADRB2/PDGFC |
| GOBP | MYD88 INDEPENDENT TOLL LIKE RECEPTOR SIGNALING PATHWAY | 33 | 0.542661222 | 1.764099626 | 0.003035144 | 0.018516956 | 0.012934393 | 4807 | tags=55%, list=23%, signal=42% | IRF7/UBE2D3/TNIP3/IKBKE/UBE2D1/TBK1/BIRC3/TRAF3/IKBKG/CASP8/FADD/UBE2D2/LY96/CHUK/CD300LF/TANK/CD40/TICAM1 |
| GOBP | REGULATION OF CD4 POSITIVE ALPHA BETA T CELL ACTIVATION | 59 | 0.446941802 | 1.632542686 | 0.003005199 | 0.018370271 | 0.012831931 | 3986 | tags=41%, list=19%, signal=33% | ZC3H12A/CD274/NFKBIZ/IL4R/PRKCQ/CD83/SASH3/JAK3/IFNG/SOCS1/CBFB/IL12B/LGALS9/CCL19/IL23A/CD86/NFKBID/XCL1/ANXA1/HLA-DRA/IL12RB1/NLRP3/CD80/FOXP3 |
| GOCC | OLIGOSACCHARYLTRANSFERASE COMPLEX | 13 | 0.693731935 | 1.797382682 | 0.002984126 | 0.018258068 | 0.012753555 | 4675 | tags=77%, list=22%, signal=60% | OSTC/TUSC3/OST4/RPN1/DDOST/TMEM258/RPN2/STT3B/DAD1/MAGT1 |
| GOBP | POSITIVE REGULATION OF RELEASE OF CYTOCHROME C FROM MITOCHONDRIA | 27 | 0.574428377 | 1.784944118 | 0.002977558 | 0.018234489 | 0.012737085 | 3321 | tags=52%, list=16%, signal=44% | PPIF/FAM162A/PYCARD/BAK1/BIK/MMP9/TNFSF10/BID/PDCD5/PLAUR/BNIP3/MFF/BAX/CIDEB |
| GOBP | RESPONSE TO PEPTIDOGLYCAN | 12 | 0.71024184 | 1.800405128 | 0.002967268 | 0.018188055 | 0.012704651 | 3310 | tags=58%, list=16%, signal=49% | NOD2/MYD88/DEFB124/IL6/CARD9/TREM2/IRAK3 |
| GOBP | NUCLEOSOME ASSEMBLY | 118 | 0.386926989 | 1.580157457 | 0.002942322 | 0.018051614 | 0.012609344 | 5270 | tags=43%, list=25%, signal=32% | RUVBL1/H2AX/CENPN/MACROH2A1/KNL1/CENPW/CENPX/MCM2/ASF1B/OIP5/HJURP/CENPM/CENPK/CENPA/IPO4/CENPS/HAT1/NASP/NPM1/GRWD1/CENPH/CENPI/NAP1L4/HMGB2/H2BC9/MACROH2A2/H3C10/MIS18A/SMARCA5/SPTY2D1/DAXX/CENPL/CHAF1A/NAA60/CENPO/H2BC10/CENPU/H1-2/CHAF1B/H3C7/H2BC12/H3C8/H4C4/H4C9/H2BC13/H1-5/RBBP4/H2BC6/H4C2/CENPQ/H1-3 |
| GOBP | TRANSITION METAL ION HOMEOSTASIS | 130 | 0.371449644 | 1.537922502 | 0.00292951 | 0.017989443 | 0.012565917 | 2252 | tags=22%, list=11%, signal=20% | LCN2/S100A9/LTF/S100A8/ATOX1/HMOX2/GLRX3/SCO2/SLC39A6/ATP6V0D1/HIF1A/ATP6V1G1/LCK/SLC25A28/ABCB6/AP3D1/NUBP1/IFNG/SCO1/MYC/HMOX1/SLC31A1/STEAP4/SLC39A4/COMMD1/AP3B1/TFRC/CUL1/ATP6V1A |
| GOMF | DYSTROGLYCAN BINDING | 10 | -0.732078213 | -1.833522543 | 0.002917201 | 0.017930245 | 0.012524566 | 3420 | tags=70%, list=16%, signal=59% | AGR2/VCL/CLASP1/AGR3/MAP2/DAG1/DMD |
| GOBP | ENDOTHELIAL CELL MIGRATION | 213 | -0.299237495 | -1.41822312 | 0.002915433 | 0.017930245 | 0.012524566 | 4056 | tags=38%, list=19%, signal=31% | HDAC9/HDAC5/SERPINF1/PRKD2/ANGPT1/PIK3CA/SCARB1/MAP2K5/TNFSF12/PTEN/FOXC2/FSTL1/NRP1/CYP1B1/PLXND1/VEGFC/FGF1/EGR3/PXN/SIRT1/ABL1/BMP4/SRPX2/JCAD/SMOC2/AGT/PRKD1/RGCC/BCAS3/TMSB4X/AMOT/PTPRM/STAT5A/EDN1/CLEC14A/SLIT2/CCN3/SPRED1/PRKCA/SVBP/MMRN2/MET/EFNA1/PPARG/EGF/SASH1/RRAS/ACVRL1/ADGRA2/SYNJ2BP/APOE/PTK2/PIK3CB/GREM1/MECP2/PLEKHG5/RHOB/KLF4/NR2F2/FGFR1/PTN/MEF2C/AKT3/RHOJ/STARD13/ROCK2/DNAJA4/ATOH8/MEOX2/ATP2B4/FBXW7/SP1/CCBE1/DCN/GATA2/MAP3K3/PLPP3/PATZ1/EFNB2/GATA3 |
| GOBP | CENTRAL NERVOUS SYSTEM PROJECTION NEURON AXONOGENESIS | 26 | -0.555895291 | -1.8120502 | 0.002915329 | 0.017930245 | 0.012524566 | 2440 | tags=42%, list=12%, signal=37% | SLIT2/EPHB6/TSKU/DCLK1/ADARB1/NFIB/PAFAH1B1/EPHB1/NIN/GLI2/C12orf57 |
| GOMF | HMG BOX DOMAIN BINDING | 14 | -0.67227984 | -1.858286697 | 0.002910571 | 0.017930245 | 0.012524566 | 3891 | tags=71%, list=19%, signal=58% | HOXA3/EGR2/PRRX1/MEOX1/HOXC4/PAX3/TCF12/POU3F3/SP1/GATA3 |
| GOBP | MODULATION OF PROCESS OF OTHER ORGANISM INVOLVED IN SYMBIOTIC INTERACTION | 92 | 0.414555427 | 1.623237545 | 0.002889893 | 0.017827642 | 0.012452896 | 3477 | tags=36%, list=17%, signal=30% | ZC3H12A/KPNA2/IFI27/LTF/HDAC1/TYMS/PHB/VAPA/CCL4/PSMC3/ATG5/HSPA8/SERPINB9/CCL8/ATG7/CASP8/TNIP1/SUGT1/TARDBP/KPNB1/HIPK2/SCRIB/SFTPD/CCL5/LEF1/CFL1/SMARCA4/TAF11/PPIB/CPSF4/PHB2/PC/KPNA3 |
| GOBP | POLYOL BIOSYNTHETIC PROCESS | 59 | 0.448006929 | 1.636433271 | 0.002862239 | 0.017673275 | 0.012345068 | 4351 | tags=49%, list=21%, signal=39% | GCH1/PTS/GOT1/PTAFR/SPTLC2/IPPK/PLCG2/CYP27B1/ITPKC/IMPA2/PCK2/DHFR/PLEK/P2RY1/AGK/P2RY6/PGP/SPTLC1/SPTLC3/CD244/PPIP5K1/CYP2R1/AVPR1B/SPHK1/NTSR1/ACER3/SPR/HRH1/ASAH2B |
| GOBP | CHAPERONE MEDIATED PROTEIN TRANSPORT | 11 | 0.723294229 | 1.794966714 | 0.002860968 | 0.017673275 | 0.012345068 | 1930 | tags=64%, list=9%, signal=58% | TIMM8B/TIMM13/TIMM10/BAG3/TIMM8A/HSPA8/TOR1A |
| GOBP | RESPIRATORY BURST | 32 | 0.52371153 | 1.690663085 | 0.002859828 | 0.017673275 | 0.012345068 | 2650 | tags=34%, list=13%, signal=30% | CD24/PGAM1/CLEC7A/SLAMF8/HCK/RAC2/GRN/NCF2/PIK3CD/CD52/CYBA |
| GOBP | SOMATIC RECOMBINATION OF IMMUNOGLOBULIN GENE SEGMENTS | 53 | 0.468362216 | 1.686230223 | 0.002855412 | 0.017673275 | 0.012345068 | 5845 | tags=58%, list=28%, signal=42% | POLB/EXOSC3/EXO1/BATF/NSD2/MAD2L2/RNF168/PRKDC/TFRC/HMCES/XRCC4/LIG4/HSPD1/ERCC1/MSH3/RNF8/TCF3/IL10/FOXP3/PARP3/TNFSF13/PTPRC/CD28/CD40/TBX21/SLC15A4/MLH1/KMT5C/CLCF1/TP53BP1/PAXIP1 |
| GOBP | ENTRY INTO HOST | 148 | 0.36685922 | 1.542845186 | 0.002814486 | 0.017442544 | 0.012183899 | 4109 | tags=38%, list=20%, signal=31% | SERPINB3/KRT6A/TRIM14/EPHA2/CDK1/CXCR4/TMPRSS4/LDLR/PLSCR1/DYNLT1/PHB/TRIM22/PPIA/CXCL8/CTSL/ACE2/IFITM1/P4HB/TRIM62/TRIM21/FCN1/TRIM10/IFITM3/LGALS9/CCR5/SLC52A2/SMPD1/XPR1/SELPLG/ITCH/NPC1/TFRC/VAMP8/TRIM5/SLAMF1/SIVA1/CD86/IDE/EXOC2/SNX3/CLEC4G/ICAM1/CIITA/TRIM25/KPNA3/IFITM2/TNFRSF4/TRIM11/NECTIN2/SLC20A2/CDH1/CD80/PTX3/SLC1A5/HYAL3/GPR15 |
| GOBP | NEGATIVE REGULATION OF T CELL PROLIFERATION | 61 | 0.451243936 | 1.656042139 | 0.00280528 | 0.017401541 | 0.012155258 | 4262 | tags=43%, list=20%, signal=34% | CD274/TNFRSF21/IDO1/PLA2G2F/ARG1/CEBPB/GLMN/LGALS9/IL2RA/LILRB4/CD86/CRTAM/TMEM131L/SCRIB/SFTPD/XCL1/CLEC4G/PELI1/CASP3/LILRB2/LILRB1/HLA-G/CD80/IL10/FOXP3/CR1 |
| GOBP | POSITIVE REGULATION OF MEMBRANE POTENTIAL | 16 | 0.669995418 | 1.855283507 | 0.002804045 | 0.017401541 | 0.012155258 | 1945 | tags=38%, list=9%, signal=34% | MFN1/VCP/STOML2/BID/MTLN/CTNS |
| GOBP | ERROR FREE TRANSLESION SYNTHESIS | 22 | 0.594088115 | 1.75863132 | 0.002792384 | 0.017353592 | 0.012121765 | 4746 | tags=59%, list=23%, signal=46% | PCNA/VCP/RPA3/UFD1/RFC2/RFC3/POLDIP2/RFC4/NPLOC4/RPA1/POLD1/SPRTN/RFC5 |
| GOBP | NEPHRON TUBULE FORMATION | 17 | -0.633549338 | -1.83631804 | 0.002759654 | 0.017166073 | 0.011990779 | 2967 | tags=53%, list=14%, signal=45% | IRX3/WNT6/IRX2/NOG/SOX8/GREM1/OSR1/IRX1/GATA3 |
| GOBP | HISTONE MRNA METABOLIC PROCESS | 24 | 0.595838027 | 1.795401359 | 0.002757611 | 0.017166073 | 0.011990779 | 1958 | tags=38%, list=9%, signal=34% | EXOSC4/SNRPG/SNRPF/NCBP1/SNRPD3/LSM1/CPSF3/LSM10/SNRPB |
| GOBP | MITOCHONDRIAL RNA PROCESSING | 17 | 0.64436346 | 1.797944744 | 0.002757611 | 0.017166073 | 0.011990779 | 2661 | tags=53%, list=13%, signal=46% | MTO1/PNPT1/FASTKD5/HSD17B10/TRMT10C/ELAC2/TBRG4/PUS1/TRNT1 |
| GOBP | POSITIVE REGULATION OF TYPE I INTERFERON MEDIATED SIGNALING PATHWAY | 13 | 0.696959979 | 1.805746188 | 0.002755835 | 0.017166073 | 0.011990779 | 2306 | tags=54%, list=11%, signal=48% | IRF7/WNT5A/MMP12/IKBKE/TBK1/NLRC5/FADD |
| GOCC | PHAGOCYTIC VESICLE MEMBRANE | 76 | 0.443889496 | 1.684210216 | 0.002728274 | 0.017033964 | 0.011898499 | 5241 | tags=43%, list=25%, signal=33% | RAB38/ATP6V0D1/ATP6V0B/TAP1/TLR2/TAP2/RAB31/RAB5A/ATG5/CORO1A/RAC2/RAB10/VAMP8/HLA-E/VAMP7/TAPBP/CYBA/ATP6V0E1/STX4/RAB8A/B2M/HLA-F/HLA-B/ATP6V0A2/HLA-G/DNM2/TLR1/HLA-A/ATP6V0C/RAB32/SYT7/CYBB/HLA-C |
| GOBP | NUCLEOBASE METABOLIC PROCESS | 33 | 0.546050557 | 1.775117779 | 0.00272701 | 0.017033964 | 0.011898499 | 3928 | tags=52%, list=19%, signal=42% | GDA/TYMP/CTPS1/TYMS/ACP3/GART/SHMT2/APRT/HPRT1/XDH/RRM1/UMPS/GMPS/KDM1A/CAD/GMPR2/MTOR |
| GOMF | EXTRACELLULAR MATRIX STRUCTURAL CONSTITUENT CONFERRING COMPRESSION RESISTANCE | 22 | -0.582379605 | -1.813570999 | 0.002667021 | 0.016682537 | 0.011653022 | 5149 | tags=50%, list=25%, signal=38% | VCAN/PRG4/BGN/OGN/CHADL/HSPG2/PODN/PRELP/DCN/ASPN/TUFT1 |
| GOBP | MONOSACCHARIDE METABOLIC PROCESS | 263 | 0.312743136 | 1.397495501 | 0.002655832 | 0.016628036 | 0.011614952 | 4139 | tags=31%, list=20%, signal=25% | ENO1/FABP5/TPI1/PGAM1/PGK1/PGM2/GOT2/PPARD/HK2/GOT1/TIGAR/OAS1/GALE/PHLDA2/PGD/FUT2/GSTO1/SLC23A2/PTPN2/MAEA/MDH2/ZMPSTE24/ALDOA/SLC25A13/PMM2/FOXK2/FUT1/GAPDH/SLC2A1/FUOM/PPP1CA/SLC25A10/TALDO1/SIRT7/NUDT5/GPI/ADIPOR1/GALK1/IGFBP3/SESN2/PCK2/MPI/BCKDK/SLC25A11/MDH1/SLC37A4/RBKS/LCMT1/ATF4/SLC2A3/CHST1/PASK/RUBCNL/SDHAF3/PFKFB2/PFKP/PDHB/GLYCTK/TNF/PKM/G6PD/PFKFB4/ADPGK/SLC35B4/DLAT/CREM/PGP/PC/SLC23A1/G6PC1/ENO3/GSK3A/PHKG2/PPP1R3D/DUSP12/PGK2/MTOR/MAPK14/MAN2B1/TKT/PDHA1 |
| GOBP | PROTEIN KINASE B SIGNALING | 242 | -0.287938624 | -1.387006199 | 0.002654913 | 0.016628036 | 0.011614952 | 3778 | tags=31%, list=18%, signal=26% | ANGPT1/GPER1/MSTN/PIK3CA/PTEN/GCNT2/PHLPP1/SFRP5/FGFR3/ERBB3/KLB/FGF1/IL18/ERBB4/DLG1/SIRT1/PHLPP2/PDGFRB/KITLG/LOX/MERTK/NRG2/PEAR1/INSR/LEP/STK3/IGF1R/TNFAIP8L3/MET/ESR1/PDGFA/FGF9/EGF/EGFR/RRAS/OTUD3/HIP1/DDIT3/AKR1C3/PTK2/PIK3CB/FGFR2/ITGB1/GAS6/NTRK2/ZFP36L1/KLF4/FGFR1/PPARA/RICTOR/OSBPL8/IGFBP5/F10/PINK1/DAG1/FERMT2/RTN4/MYOC/TSPYL5/FYN/PLEKHA1/PPP2R5C/IRS1/MAGI2/PIK3R1/AXL/PDGFRA/TCF7L2/SPRY2/KIT/IRS2/NOP53/GATA3/F3/BTC |
| GOBP | REGULATION OF INTRACELLULAR PROTEIN TRANSPORT | 243 | 0.322413902 | 1.432287336 | 0.002652343 | 0.016628036 | 0.011614952 | 4118 | tags=35%, list=20%, signal=28% | ZC3H12A/RAN/IFI27/TFDP1/GZMB/FZD5/NMT1/UBE2L3/YWHAQ/FERMT1/ZPR1/UBE2D3/SFN/ECT2/TMEM30B/IFNG/BCAP31/IL1B/UBL5/ATG13/SIRT7/BAG3/PDCD10/BID/CDK5/HAX1/MTCL1/PDCD5/TCAF2/TMEM30A/EDEM1/NOLC1/RAC2/KIF20B/PLK3/CD36/ITGB2/CIB1/SAE1/DERL2/HRAS/RANGAP1/CASP8/TP63/TXN/TARDBP/YWHAZ/ARIH2/NDEL1/PARK7/PIK3R2/EDEM2/UBE2J2/HCLS1/SREBF2/OAZ1/MFF/HTRA2/MIEF1/DYNLT2B/SP100/LCP1/UBAC2/PKIA/LMAN1/PRKCD/RIOK2/UHMK1/NUTF2/MAPK8/CEMIP/SREBF1/HDAC3/GSK3A/YOD1/CACNB3/NUP58/EMD/CDH1/SLC1A1/JUP/MAPK14/ATP5IF1/UBE2J1 |
| GOBP | NUCLEOSOME ORGANIZATION | 156 | 0.356637749 | 1.51331701 | 0.002645396 | 0.016609133 | 0.011601748 | 5270 | tags=38%, list=25%, signal=29% | RUVBL1/H2AX/PSME4/CENPN/MACROH2A1/KNL1/CENPW/CENPX/MCM2/ASF1B/OIP5/HJURP/CENPM/CENPK/CENPA/IPO4/POLE3/CENPS/HAT1/SMARCD3/NASP/NPM1/GRWD1/CENPH/CENPI/NAP1L4/HMGB2/H2BC9/MACROH2A2/H3C10/PAF1/ZNHIT1/MIS18A/SMARCA4/SMARCA5/HMGA1/RNF8/SPTY2D1/DAXX/CENPL/CHAF1A/NAA60/CENPO/H2BC10/CENPU/H1-2/CHAF1B/H3C7/H2BC12/H3C8/H4C4/H4C9/H2BC13/H1-5/RBBP4/H2BC6/H4C2/SRCAP/CENPQ/H1-3 |
| GOBP | HEART GROWTH | 81 | -0.389920234 | -1.622973852 | 0.002640493 | 0.016593856 | 0.011591076 | 4567 | tags=42%, list=22%, signal=33% | AKAP6/PI16/S1PR1/TGFB2/KCNK2/PARP2/SAV1/PTEN/FOXC2/VGLL4/ERBB4/JARID2/RGS2/PDLIM5/YAP1/EDN1/NOG/FOXC1/FGFR2/SORBS2/BMPR1A/PPARA/DIPK2A/MEF2C/RBP4/AKAP13/WNT2/ADRA1A/RBPJ/MAPK1/TGFBR2/ZFPM2/MEIS1/TGFBR3 |
| GOBP | ENDOCARDIAL CUSHION FORMATION | 24 | -0.569610615 | -1.803961026 | 0.00262928 | 0.01653886 | 0.011552661 | 5056 | tags=50%, list=24%, signal=38% | SMAD4/MSX1/TMEM100/TGFB2/BMP2/ROBO2/NOG/BMPR1A/RBPJ/TGFBR2/BMP7/DCHS1 |
| GOBP | SCHWANN CELL DEVELOPMENT | 27 | -0.564895433 | -1.850708429 | 0.002622779 | 0.016513429 | 0.011534897 | 2995 | tags=41%, list=14%, signal=35% | POU3F1/LGI4/FA2H/NTRK2/LAMB2/DAG1/SKI/MYOC/PARD3/SOD1/ARHGEF10 |
| GOBP | CYTOKINE PRODUCTION INVOLVED IN INFLAMMATORY RESPONSE | 48 | 0.476541623 | 1.684024509 | 0.002614002 | 0.016473606 | 0.01150708 | 4090 | tags=42%, list=20%, signal=34% | ZC3H12A/NOD2/CLEC7A/MYD88/PYCARD/PLA2G3/HIF1A/GBP5/STAT3/NOS2/CD6/IL6/LILRB4/TNF/NLRC3/NLRP7/PLD4/MAPK14/IL17F/GPSM3 |
| GOBP | PYRIMIDINE NUCLEOTIDE METABOLIC PROCESS | 47 | 0.483510235 | 1.690321268 | 0.002613801 | 0.016473606 | 0.01150708 | 1935 | tags=28%, list=9%, signal=25% | TYMP/UPP1/UCK2/NME1/CTPS1/TYMS/ENTPD7/CMPK2/DTYMK/TDG/UMPS/DCTPP1/SMUG1 |
| GOBP | REGULATION OF LEUKOCYTE DEGRANULATION | 42 | 0.512627561 | 1.749798857 | 0.00260946 | 0.016473606 | 0.01150708 | 4049 | tags=48%, list=19%, signal=38% | IL4R/PTAFR/PLA2G3/LYN/HMOX1/CD177/SYK/RAC2/LGALS9/ITGB2/VAMP8/C12orf4/VAMP7/ADGRE2/STX4/CCR2/HLA-F/UNC13D/CD300A/FGR |
| GOBP | SOMITE DEVELOPMENT | 81 | -0.390366978 | -1.624833344 | 0.002586879 | 0.016348688 | 0.011419823 | 5538 | tags=46%, list=27%, signal=34% | NUP133/SFRP1/POFUT1/MED12/SFRP2/DLL1/EP300/PCDH8/SMAD4/LOXL3/SEMA3C/WNT11/PPP2R3A/ATM/KAT2A/XRCC2/FOXC2/MIB1/MEOX1/NKD1/NOG/DMRT2/LRP6/FOXC1/BMPR1A/ROR2/FRZB/ALDH1A2/SMAD3/RBPJ/MEOX2/PTCH1/LFNG/PLXNA2/WDR19/AXIN2/COBL |
| GOBP | SOMATIC DIVERSIFICATION OF IMMUNOGLOBULINS | 61 | 0.453758454 | 1.665270292 | 0.002577442 | 0.016304382 | 0.011388874 | 5845 | tags=54%, list=28%, signal=39% | POLB/EXOSC3/EXO1/BATF/NSD2/POLQ/MAD2L2/RNF168/PRKDC/TFRC/HMCES/XRCC4/LIG4/HSPD1/ERCC1/MSH3/RNF8/TCF3/SAMHD1/IL10/FOXP3/PARP3/TNFSF13/PTPRC/CD28/CD40/TBX21/SLC15A4/MLH1/KMT5C/CLCF1/TP53BP1/PAXIP1 |
| GOBP | RHO PROTEIN SIGNAL TRANSDUCTION | 133 | -0.324864635 | -1.459825878 | 0.002571158 | 0.016279965 | 0.011371819 | 3680 | tags=30%, list=18%, signal=25% | LPAR4/ARHGEF2/NRP1/GNA12/GNA13/RIPOR1/ABL1/PDGFRB/ARHGEF1/RTN4R/NET1/F2R/MET/APOE/ARHGAP35/ARHGAP42/PLEKHG5/RHOB/SCAI/NTN1/RHOJ/CTNNAL1/STARD13/AKAP13/ROCK2/ARHGAP6/ADRA1A/ARHGAP29/ARHGEF12/MYOC/BCL6/LPAR1/EPS8/ADRA2A/AGTR1/DLC1/ARHGEF28/ARHGEF10/KANK1/KANK2 |
| GOBP | NEGATIVE REGULATION OF CELL ACTIVATION | 185 | 0.341842429 | 1.468748659 | 0.002542916 | 0.016116329 | 0.011257516 | 4814 | tags=37%, list=23%, signal=29% | ZC3H12A/CD274/IL4R/TNFRSF21/LDLR/HMGB3/IDO1/PTPN2/IRF1/PLA2G2F/SAMSN1/CST7/ARG1/CEBPB/JAK3/LYN/SOCS1/CBFB/HMOX1/TIGIT/ZC3H8/GLMN/LGALS9/PTPN22/THBD/LAG3/GRN/IL2RA/PGLYRP2/LILRB4/LAPTM5/CD86/CRTAM/TMEM131L/SCRIB/SFTPD/XCL1/PGLYRP3/CLEC4G/ANXA1/PELI1/CASP3/TYROBP/PRKCD/CCR2/TNFRSF13B/LILRB2/PAG1/HLA-F/TBC1D10C/MNDA/LILRB1/HLA-G/CD300A/CD80/IL10/FOXP3/FGR/PARP3/CD300LF/LST1/CR1/PTPRC/SH2B3/INHA/FOXF1/SERPINE2/RHBDD3/TBX21 |
| GOMF | TUMOR NECROSIS FACTOR RECEPTOR SUPERFAMILY BINDING | 49 | 0.4848047 | 1.725007185 | 0.002537198 | 0.016095272 | 0.011242808 | 4500 | tags=51%, list=22%, signal=40% | STAT1/MYD88/CFLAR/TNFSF10/LTB/TRAP1/BID/TRAF1/TRAF3/BEX3/CASP8/FADD/BABAM2/SIVA1/TNFSF9/TNF/FASLG/TNFSF15/TRADD/CASP3/TNFSF13B/TRAF4/TRAF2/TNFSF13/TNFSF14 |
| GOBP | TRANSFERRIN TRANSPORT | 35 | 0.525584255 | 1.740564211 | 0.002536993 | 0.016095272 | 0.011242808 | 4452 | tags=46%, list=21%, signal=36% | ATP6V0D1/ATP6V1B2/ATP6V1G1/ATP6V0B/ATP6V1F/ATP6V1D/ATP6V1C1/ATP6V1C2/TFRC/ATP6V1H/ATP6V1A/ATP6V0E1/STEAP3/ATP6V0A2/ARHGAP1/DNM2 |
| GOBP | NUCLEAR TRANSCRIBED MRNA CATABOLIC PROCESS EXONUCLEOLYTIC | 35 | 0.525636061 | 1.740735777 | 0.002536993 | 0.016095272 | 0.011242808 | 3878 | tags=49%, list=19%, signal=40% | EXOSC4/LSM2/LSM7/EXOSC3/LSM1/DIS3/LSM5/EXOSC1/LSM4/POLR2G/EXOSC2/EXOSC5/LSM6/SKIV2L/CNOT6/EXOSC8/DCPS |
| GOBP | POSITIVE REGULATION OF RRNA PROCESSING | 10 | 0.744049576 | 1.796813414 | 0.002536522 | 0.016095272 | 0.011242808 | 4137 | tags=80%, list=20%, signal=64% | DIMT1/SIRT7/TRMT112/UTP15/RIOK1/RIOK2/HEATR1/BUD23 |
| GOCC | MYOFILAMENT | 23 | -0.570874782 | -1.801672213 | 0.002536381 | 0.016095272 | 0.011242808 | 2023 | tags=48%, list=10%, signal=43% | TMOD2/ACTA1/TNNC2/FHOD3/TNNT1/TPM2/TNNC1/TNNI2/LMOD1/TPM1/TMOD1 |
| GOBP | ALCOHOL METABOLIC PROCESS | 352 | 0.30220981 | 1.386039461 | 0.002535025 | 0.016095272 | 0.011242808 | 4357 | tags=32%, list=21%, signal=26% | AKR1B10/IDH3A/TPI1/RAN/GDPD3/GCH1/PTS/PPARD/APOL1/GOT1/MOXD1/LDLR/GK/PTAFR/CYP7B1/ALDH1A3/SPTLC2/IPPK/RDH10/DHRS9/DPAGT1/SRD5A3/PCSK9/SQLE/APOL2/PLCG2/CH25H/TTC39B/RDH16/PLCD1/CYP27B1/ITPKC/RDH12/ADH7/NFKB1/GBA2/ABCG1/DGAT1/DHRS13/ALDH2/IMPA2/IDH2/IDH3B/FDXR/INPP1/COQ2/EBP/ALDH3B2/GALK1/SULT2B1/PCK2/COQ3/MTMR2/DHFR/SMPD1/SEC14L2/NPC1/PLEK/LPCAT3/MINPP1/CYP27C1/BPNT1/ARV1/SDR16C5/KPNB1/SULT1E1/NSDHL/PLCD4/PARK7/P2RY1/ACACA/SGPP2/DPM2/SREBF2/LBR/SYNJ2/PNPLA4/G6PD/HMGCR/LDLRAP1/AGK/P2RY6/CLN6/DPM1/CYP3A7/PGP/CYP2C9/SPTLC1/SPTLC3/GALR2/PLCB3/VLDLR/CD244/PPIP5K1/CYP2R1/SREBF1/AVPR1B/SPHK1/NTSR1/ACER3/PLD4/ERLIN1/FDPS/PLPP2/SPR/PLPP6/LIMA1/GK2/HRH1/ELOVL6/FDFT1/ASAH2B/NFYC |
| GOBP | REGULATION OF NERVOUS SYSTEM DEVELOPMENT | 401 | -0.261001323 | -1.333746604 | 0.002529871 | 0.016095272 | 0.011242808 | 4588 | tags=32%, list=22%, signal=26% | CUX2/NUMB/SHTN1/LRP1/HEY1/SEMA3A/NRXN1/CERS2/RND2/FLRT2/CTNNB1/TRPC6/OBSL1/RUFY3/SORL1/SERPINF1/BMP2/EGR2/XRCC2/AMIGO1/RNF112/LINGO2/GPER1/SS18L1/SEMA6D/PTEN/NRP1/HOOK3/PLXND1/VEGFC/EFNB3/RELN/TIAM2/SLITRK2/TG/DLG1/ANKRD27/SRPX2/ROBO2/HES1/CBLN1/CXCL12/NAP1L1/JAM2/RTN4R/SOX10/SPEN/ZNF365/SHOX2/SEMA3E/GOLGA4/NPTN/YAP1/MACF1/L1CAM/PRUNE1/SLIT2/SNW1/NOG/IL6ST/CLSTN1/LGI4/SOX8/BHLHB9/FZD3/SPART/SEMA4G/LRRN1/NLGN1/MAP6/ARNTL/SEMA6A/WASF3/DUSP10/FGF13/PPARG/CTNNA1/SEMA3B/PER2/PLAG1/LIF/SHANK3/RYK/TMEM98/BIN1/PTPRS/ASPA/NTRK2/FN1/BMPR1A/CAPRIN2/TRAK2/PTN/DOCK7/NPHP3/MAP2/ELL3/NTN1/CLSTN2/DAG1/ETV5/PCM1/SKI/WNT2/RTN4/NUMBL/BMP7/HOXB3/RAPGEF2/DLG5/STAR/PAK3/ELAPOR2/DAAM2/EPHB1/NIN/PLXNA2/SEMA3G/PARD3/BHLHE41/SLITRK6/DBN1/LRP4/FBXO31/GLI3/KIT/TPPP/MAP1B/EEF2K |
| GOCC | MITOTIC SPINDLE MIDZONE | 14 | 0.684691372 | 1.809009918 | 0.002508249 | 0.016017503 | 0.011188485 | 1894 | tags=50%, list=9%, signal=45% | AURKB/KIF18B/PRC1/CENPE/KIF18A/KIF20B/RCC2 |
| GOBP | REGULATION OF MUSCLE ADAPTATION | 78 | -0.385547733 | -1.595519076 | 0.00249443 | 0.015944413 | 0.01113743 | 3200 | tags=33%, list=15%, signal=28% | PDE9A/FOXO1/JARID2/AGT/RGS2/EDN1/IL6ST/PRKCA/FOXO3/SGCA/CAMK2G/KLF4/PPARA/FBXO32/PPARGC1A/IGFBP5/DAG1/LMCD1/ROCK2/ADRA1A/SMAD3/ATP2B4/TNNT1/TNNC1/MEF2A/UTRN |
| GOCC | CYCLIN DEPENDENT PROTEIN KINASE HOLOENZYME COMPLEX | 42 | 0.51396821 | 1.754375019 | 0.002492837 | 0.015944413 | 0.01113743 | 3679 | tags=52%, list=18%, signal=43% | CCNB1/CDK1/CCNB2/CKS2/PCNA/CCNE1/CCNA2/CCNE2/CCNF/CDKN1A/BCCIP/CKS1B/CCND2/CCNO/CDK7/CDK2/RB1/CCNH/CDK9/CCND3/CDKN2D/CDK4 |
| GOBP | NUCLEOTIDE PHOSPHORYLATION | 128 | 0.374210102 | 1.550538975 | 0.002489717 | 0.015944413 | 0.01113743 | 4978 | tags=42%, list=24%, signal=32% | ENO1/TPI1/PGAM1/PGK1/HK2/NME1/TIGAR/CMPK2/HIF1A/EIF6/DTYMK/NUP37/NUP210/LDHA/ALDOA/FOXK2/NUP88/IFNG/GAPDH/PNKP/GPI/AK1/GALK1/NUP50/RAE1/DHTKD1/SEC13/PFKFB2/PFKP/NUP155/PKM/NDC1/PFKFB4/ADPGK/NUP188/PRKAG1/NUP85/NUP205/OGDHL/ENO3/PGK2/NME7/NUP58/NUP107/NUP93/POM121/HK3/NUP160/ZBTB7A/NUP35/NUP42/ENTPD5/BPGM/NME6 |
| GOBP | T HELPER 17 TYPE IMMUNE RESPONSE | 32 | 0.528697733 | 1.706759713 | 0.002488827 | 0.015944413 | 0.01113743 | 5931 | tags=62%, list=28%, signal=45% | ZC3H12A/NFKBIZ/PRKCQ/ENTPD7/PHB/STAT3/IL12B/IL6/IL23A/BATF/NFKBID/IL12RB1/FOXP3/NLRP10/MALT1/TBX21/SLAMF6/IL6R/IRF4/TNFSF18 |
| GOMF | HEME COPPER TERMINAL OXIDASE ACTIVITY | 27 | 0.579375018 | 1.80031501 | 0.002447527 | 0.015704379 | 0.010969763 | 4183 | tags=56%, list=20%, signal=44% | COX6B1/COX7B/COX5A/COX8A/C15orf48/COX7A2P2/COX7A2/NDUFA4/COX6A1/COX10/COX7C/COX5B/COX4I1/COX7B2/COX6C |
| GOBP | CALCIUM DEPENDENT CELL CELL ADHESION VIA PLASMA MEMBRANE CELL ADHESION MOLECULES | 42 | -0.490164797 | -1.794167025 | 0.002440672 | 0.015675367 | 0.010949497 | 5439 | tags=52%, list=26%, signal=39% | CDH13/AJUBA/PCDHB3/CDH6/PCDHB6/CDH5/PCDHB13/PCDH12/CDHR3/PCDHB11/DSG1/CDH10/PCDHB14/PCDHB2/PCDHB10/NLGN1/PCDHB4/PCDHB5/CDH12/CDH19/DCHS1/PCDHB16 |
| GOBP | REGULATION OF PROTEIN POLYUBIQUITINATION | 24 | 0.599559717 | 1.806615696 | 0.002438816 | 0.015675367 | 0.010949497 | 3725 | tags=46%, list=18%, signal=38% | NMI/NOD2/TRIP12/PPIA/OTUB2/UBE2D1/PTPN22/PLAA/FBXO4/OTUB1/PARP10 |
| GOBP | MITOTIC NUCLEAR MEMBRANE ORGANIZATION | 10 | 0.745036681 | 1.799197185 | 0.002405607 | 0.015479757 | 0.010812861 | 3897 | tags=70%, list=19%, signal=57% | PPP2CA/PPP2R2A/REEP4/VRK1/ANKLE2/PPP2R1A/EMD |
| GOBP | ADRENERGIC RECEPTOR SIGNALING PATHWAY | 28 | -0.535226256 | -1.767414064 | 0.002404466 | 0.015479757 | 0.010812861 | 2662 | tags=39%, list=13%, signal=34% | RGS2/ADCY9/ADRB1/AKAP13/ADRA1A/ATP2B4/RAPGEF2/PLN/LMBRD2/ADRA2A/ADRB2 |
| GOBP | SPECIFICATION OF ANIMAL ORGAN IDENTITY | 33 | -0.529796703 | -1.839248781 | 0.002394566 | 0.015438288 | 0.010783894 | 3262 | tags=42%, list=16%, signal=36% | FGF1/BMP4/ROBO2/DKK1/FGFR2/MEF2C/HOXA11/WNT2/RBPJ/SPRY1/AR/GLI3/WNT2B/AXIN2 |
| GOBP | SOMATIC DIVERSIFICATION OF IMMUNE RECEPTORS | 72 | 0.436638401 | 1.64046148 | 0.002378084 | 0.01534675 | 0.010719953 | 4824 | tags=44%, list=23%, signal=34% | POLB/EXOSC3/EXO1/BATF/NSD2/POLQ/MAD2L2/RNF168/ADAR/PRKDC/TFRC/HMCES/XRCC4/HMGB2/LIG4/HSPD1/LEF1/ERCC1/MSH3/RNF8/TCF3/SAMHD1/IL10/FOXP3/DCAF1/PARP3/TNFSF13/PTPRC/CD28/CD40/TBX21/SLC15A4 |
| GOBP | RIBOSOMAL LARGE SUBUNIT BIOGENESIS | 69 | 0.435716696 | 1.627786662 | 0.002370755 | 0.015314167 | 0.010697193 | 3572 | tags=43%, list=17%, signal=36% | RPL26L1/NOP16/WDR12/EIF6/NHP2/HEATR3/NOP2/DDX28/FASTKD2/NIP7/MRTO4/SNU13/GTPBP4/MRPL20/EBNA1BP2/PPAN/RRP15/BOP1/WDR74/NOC2L/TRAF7/NPM1/RPF2/PES1/NIFK/MRPL1/NLE1/RRS1/PAK1IP1/RPLP0 |
| GOMF | WIDE PORE CHANNEL ACTIVITY | 26 | 0.590870909 | 1.810426882 | 0.002366002 | 0.015298174 | 0.010686022 | 3884 | tags=42%, list=19%, signal=34% | GJB2/GJB6/PANX1/TOMM40L/PRF1/VDAC2/TOMM40/VDAC1/VDAC3/GJA5/GJA1 |
| GOMF | PROTEIN HETERODIMERIZATION ACTIVITY | 288 | 0.31943183 | 1.439827788 | 0.002360229 | 0.015275546 | 0.010670216 | 4656 | tags=36%, list=22%, signal=28% | PDSS1/RAN/H2AX/PAFAH1B3/BCL2A1/KATNB1/MACROH2A1/H2AZ1/AURKA/IRAK2/BAK1/HIF1A/PPP2CA/CENPW/PGLYRP4/PHB/IRAK1/POLE4/TOP2A/PRMT5/VAPA/CENPA/ABCG4/CEBPB/SNX1/ABCG1/CD3D/H2AJ/P4HB/POLE3/IL12B/ALG2/CENPS/NOLC1/LSM5/GTF2A2/PSMF1/TENM2/RCC1/SAE1/TAF13/CD3G/ATP1B1/IKBKG/TRMT112/SLC51A/SUPT7L/BTBD11/BDKRB2/ATF4/BOK/TPM4/H2BC9/RRAGC/PIK3R2/P2RY1/MACROH2A2/EXT1/CYBA/LSM6/H3C10/MLX/ATP1A1/BAX/PGLYRP3/TAF11/GADD45A/CD3E/CHUK/RALGAPA2/HIP1R/IRAK3/PHB2/KATNA1/TCF3/PPP2R1A/EXT2/BHLHE40/DRAP1/H2AC4/ITGA3/MCL1/SUPT4H1/H2AC13/H2BC10/SRI/IL17F/NPAS4/XBP1/BUD23/QTRT2/H3C7/SMC1A/SUPT3H/NFYC/H2BC12/MIGA2/H3C8/H4C4/H2AC16/PDSS2/H4C9/H2BC13 |
| GOBP | RNA CAPPING | 33 | 0.550655815 | 1.79008869 | 0.00235725 | 0.015270981 | 0.010667027 | 5055 | tags=48%, list=24%, signal=37% | POLR2H/NCBP1/POLR2F/POLR2L/CMTR2/POLR2G/POLR2D/NCBP3/CDK7/POLR2E/CCNH/POLR2I/POLR2J/RAMAC/RNGTT/GTF2H1 |
| GOBP | POSITIVE REGULATION OF GLUCOSE TRANSMEMBRANE TRANSPORT | 43 | -0.477273634 | -1.756331816 | 0.002347905 | 0.01522512 | 0.010634993 | 3484 | tags=40%, list=17%, signal=33% | C2CD5/RAP1A/C3/INSR/ADIPOQ/RHOQ/CLIP3/GPC3/OSBPL8/BRAF/KLF15/IRS1/PIK3R1/CREBL2/SORBS1/IRS2/MEF2A |
| GOCC | PLASMA MEMBRANE BOUNDED CELL PROJECTION CYTOPLASM | 210 | -0.302539993 | -1.429771617 | 0.002346586 | 0.01522512 | 0.010634993 | 4849 | tags=31%, list=23%, signal=24% | MAPK8IP3/DNAH8/INPP5E/MAPT/MGARP/DYNC1H1/KIF17/ARMCX3/DNAH9/CFAP36/CEP162/BBS7/AK8/ARL6/DYNC2LI1/EFHC2/SPATA7/DNAL1/AP3M2/TTC30A/IFT172/ZC3H14/SEPTIN2/KIF5C/DRC3/KCNAB1/ARL8A/LCA5/CFAP91/DZIP1L/LRRK2/TRAK1/DYNC2H1/SFPQ/DNAI7/DYNC2I1/TRAF3IP1/PURA/TERF2/TULP3/DST/TRAK2/SEPTIN7/CCDC113/MAP2/CAMSAP3/BBS1/GABARAPL1/AP3S1/MAPK1/WDR35/WDPCP/MAP1A/PAFAH1B1/ATG14/TMEM108/SPAG16/DLG2/EFHC1/GLI2/GLI3/SYBU/SOD1/CCSAP/MAP4/WLS |
| GOCC | GOLGI LUMEN | 91 | -0.375849208 | -1.601666267 | 0.002344998 | 0.01522512 | 0.010634993 | 4870 | tags=36%, list=23%, signal=28% | PROS1/WNT6/GOLIM4/PDGFB/BGN/PCSK5/MUC1/CSPG4/BPNT2/WNT5B/APP/WNT7B/MUCL1/MMP16/OGN/RAB33B/MUC7/GPC1/HSPG2/SOD3/PDGFA/OMD/MUC15/GPC4/GPC6/GAS6/GPC3/PRELP/F10/DAG1/SDC4/DCN/SDC2 |
| GOBP | SEQUESTERING OF METAL ION | 14 | 0.686212172 | 1.813027996 | 0.002344313 | 0.01522512 | 0.010634993 | 1034 | tags=36%, list=5%, signal=34% | LCN2/S100A9/S100A8/S100A7/AP3D1 |
| GOBP | REGULATION OF CARDIAC MUSCLE CELL DIFFERENTIATION | 15 | -0.656325618 | -1.847732917 | 0.002329575 | 0.015164811 | 0.010592865 | 5282 | tags=80%, list=25%, signal=60% | DLL1/SMAD4/KAT2A/BMP2/BMP4/DKK1/FZD7/GREM1/MEF2C/MYOCD/SOX6/EFNB2 |
| GOMF | PROTEIN KINASE A BINDING | 51 | -0.453115795 | -1.722152126 | 0.002311594 | 0.015062353 | 0.010521297 | 4594 | tags=45%, list=22%, signal=35% | AKAP8/AKAP6/DACT2/RARA/AKAP3/RDX/CRYBG3/MYRIP/WASF2/DACT3/PRRC1/LRRK2/DACT1/C2orf88/WASF3/PRKAR2B/AKAP1/AKAP12/GSK3B/AKAP11/AKAP13/AKAP9/PJA2 |
| GOBP | REGULATION OF CD8 POSITIVE ALPHA BETA T CELL ACTIVATION | 18 | 0.651184722 | 1.846656038 | 0.002303328 | 0.015023063 | 0.010493852 | 3674 | tags=56%, list=18%, signal=46% | CD274/IRF1/SOCS1/CBFB/PTPN22/LILRB4/HLA-E/CRTAM/XCL1/LILRB1 |
| GOBP | LENS DEVELOPMENT IN CAMERA TYPE EYE | 76 | -0.409546233 | -1.685155433 | 0.002298424 | 0.015005647 | 0.010481687 | 3687 | tags=36%, list=18%, signal=29% | SIX5/CRYBG3/WNT5B/NECTIN3/DLG1/SPRED2/BMP4/WNT7B/SPRED1/NHS/SKI/WNT2/MAF/SMAD3/ABI2/TGFBR2/VIM/CDON/SPRY1/SLITRK6/TMOD1/SPRY2/MEIS1/BCAR3/WNT2B/CRYAB/GATA3 |
| GOBP | POSITIVE REGULATION OF LYMPHOCYTE DIFFERENTIATION | 98 | 0.401136847 | 1.592493737 | 0.002296365 | 0.015005647 | 0.010481687 | 4704 | tags=48%, list=23%, signal=37% | NFKBIZ/PNP/IL4R/CD83/VNN1/SASH3/RHOH/AP3D1/IFNG/SOCS1/CBFB/IL12B/XRCC6/SYK/IL7R/LGALS9/CCL19/CD27/AP3B1/IL23A/PRKDC/IL2RA/ADAM8/LILRB4/TNFSF9/CD86/LEF1/IL36B/NFKBID/IL1RL2/ANXA1/HLA-DRA/IL12RB1/LILRB2/IL15/ZAP70/NLRP3/HLA-G/CD80/IL10/FOXP3/XBP1/CR1/PTPRC/MALT1/NKAP/PPP2R3C |
| GOBP | DNA DEPENDENT DNA REPLICATION MAINTENANCE OF FIDELITY | 47 | 0.486207489 | 1.6997507 | 0.002296105 | 0.015005647 | 0.010481687 | 4287 | tags=43%, list=21%, signal=34% | TIMELESS/PCNA/RAD51/CENPX/DONSON/ALYREF/CENPS/DNA2/GEN1/BLM/BRCA2/MSH3/EME1/RFWD3/CDK9/MCM9/SAMHD1/ATR/TIPIN/POLD1 |
| GOBP | POSITIVE REGULATION OF NEURON DIFFERENTIATION | 87 | -0.376723093 | -1.594938379 | 0.002285399 | 0.014964242 | 0.010452765 | 3925 | tags=43%, list=19%, signal=35% | IRX3/FOXA1/TRIM32/KDM4C/NCOA1/TRPC6/BMP2/BMP6/RNF112/FEZ1/SOCS2/ARHGEF2/PTEN/SIN3A/BMP4/MMD/CXCL12/ZC4H2/TCF12/NBL1/GPRC5B/CSNK1D/FGFR1/GDF7/MEF2C/ZEB1/ETV5/TIMP2/BMP7/GATA2/BCL6/PCP4/NAP1L2/CDON/GLI2/TCF4/MAP1B |
| GOMF | GTPASE ACTIVATOR ACTIVITY | 249 | -0.287567043 | -1.397910891 | 0.002282586 | 0.0149604 | 0.010450081 | 5401 | tags=43%, list=26%, signal=32% | SIPA1L1/CHM/OPHN1/SYDE2/ACAP3/FLCN/ARHGAP33/TSC2/OCRL/TBC1D4/ARHGAP23/TBC1D8B/ARHGAP17/GAPVD1/TBC1D12/IQGAP2/GDI1/ARHGAP21/ARHGAP18/TBC1D9B/NF1/RGS5/TBC1D1/RASGRP3/WNT11/DAB2IP/FAM13B/NPRL3/ARHGAP19/ARHGAP44/TBC1D17/RAB3GAP2/RUNDC1/GRTP1/ARHGEF10L/PREX2/GNAQ/ALS2/SOS1/ALDH1A1/NRP1/RABEP1/TIAM2/ASAP3/TBC1D8/AGAP9/SRGAP3/ANKRD27/RASA4/AGAP4/ARHGEF1/STXBP5/TBC1D2B/LARS1/ARHGAP24/LRRK2/ARHGAP10/RASA3/RGS2/HACD3/SYDE1/CHML/PLCB1/ARHGEF15/EVI5/DOCK5/DOCK4/TBC1D15/ARHGAP20/RANBP3/ARHGEF6/ARHGAP26/RALGAPB/AGFG2/GIT2/ARHGAP12/ARFGAP3/ARHGAP35/AGAP1/TBC1D16/ARHGAP42/AGAP11/STARD13/IQGAP1/ARHGAP6/DOCK1/VAV3/ARHGAP29/SGSM2/ARHGEF12/TBC1D5/RAPGEF2/TBC1D24/RAB3GAP1/SRGAP1/RALGAPA1/RASAL2/GARNL3/BNIP2/ELMOD1/DLC1/ARHGAP31/FAM13A/ASAP2/MYO9A/SRGAP2/RABGAP1L |
| GOMF | NEUTRAL AMINO ACID TRANSMEMBRANE TRANSPORTER ACTIVITY | 32 | 0.53148665 | 1.715762987 | 0.00227241 | 0.014908255 | 0.010413657 | 5325 | tags=62%, list=26%, signal=47% | SLC6A14/SLC7A11/SFXN1/SLC7A5/SLC38A5/SLC36A1/SFXN2/SLC3A2/SLC38A9/SLC38A7/SLC6A20/SLC36A4/SLC43A2/SLC1A1/SLC1A5/SLC1A4/SLC6A19/SLC6A6/SLC38A6/SLC7A8 |
| GOBP | REGULATION OF LEUKOCYTE APOPTOTIC PROCESS | 78 | 0.419212183 | 1.597351795 | 0.002250253 | 0.014777321 | 0.010322198 | 3351 | tags=42%, list=16%, signal=36% | CD274/IRF7/WNT5A/NOD2/SLC7A11/PRELID1/PRKCQ/AURKB/CCR7/HIF1A/IDO1/ADAM17/JAK3/LYN/ZC3H8/IL7R/RIPK3/LGALS9/CCL19/CD27/DOCK8/NOC2L/CD3G/ADAM8/FADD/PIK3CD/TCP1/HCLS1/CCL5/CDKN2A/BAX/ANXA1/BCL10 |
| GOBP | DENDRITIC CELL CHEMOTAXIS | 24 | 0.6033222 | 1.817952948 | 0.00221566 | 0.014564386 | 0.010173459 | 3377 | tags=50%, list=16%, signal=42% | CXCR2/CXCR4/C1QBP/CCR7/SLAMF8/LGALS9/CCL19/CCR5/GPR183/CCL5/CCR1/CCR2 |
| GOBP | MACROPHAGE ACTIVATION | 93 | 0.418447403 | 1.643637573 | 0.002209524 | 0.014538281 | 0.010155225 | 2901 | tags=29%, list=14%, signal=25% | ZC3H12A/NMI/WNT5A/CTSC/IL4R/LDLR/PLA2G3/IFNGR1/TLR2/IFI35/TNIP2/CST7/IFNG/MIF/MFHAS1/SBNO2/SYK/IL6/ITGB2/GRN/GPR137B/JMJD6/HSPD1/SHPK/TLR8/TNF/TREM2 |
| GOBP | SCHWANN CELL DIFFERENTIATION | 35 | -0.512740326 | -1.790608973 | 0.002201767 | 0.01450144 | 0.01012949 | 3891 | tags=46%, list=19%, signal=37% | EGR2/LAMA2/ERBB3/POU3F1/GPC1/LGI4/FA2H/NTRK2/LAMB2/DAG1/SKI/MYOC/PARD3/NAB1/SOD1/ARHGEF10 |
| GOCC | DNA POLYMERASE COMPLEX | 20 | 0.627685591 | 1.814139337 | 0.002196657 | 0.014481987 | 0.010115902 | 3406 | tags=55%, list=16%, signal=46% | POLE2/POLE4/POLE3/POLD2/MCM3/DNA2/MAD2L2/POLA2/PRIM1/POLD4/POLD3 |
| GOBP | MESENCHYMAL CELL PROLIFERATION | 38 | -0.493782289 | -1.765386424 | 0.002196003 | 0.014481987 | 0.010115902 | 4986 | tags=58%, list=24%, signal=44% | TBX1/FAT4/WNT11/MSX1/LRP5/BMP2/PRRX1/BMP4/SHOX2/PDGFA/FGFR2/BMPR1A/GPC3/PTN/WNT2/NFIB/TGFBR2/BMP7/OSR1/DCHS1/CTNNBIP1/IRS2 |
| GOCC | LARGE RIBOSOMAL SUBUNIT | 108 | 0.397933733 | 1.606724886 | 0.002179933 | 0.014399987 | 0.010058624 | 3344 | tags=42%, list=16%, signal=35% | MRPL15/MRPL51/RPL26L1/MRPL35/MRPL47/MRPL13/MRPL46/MRPL37/MRPL3/MRPL27/MRPL36/MRPL11/MRPL17/MRPL42/MRPL52/NDUFAB1/MRPL32/MRPL14/MRPL20/MRPL58/MRPL54/MRPL19/MRPL50/MRPL4/MRPL16/MRPL22/MRPL12/MRPL21/MRPL41/MRPL39/MRPL23/MPV17L2/MRPL28/MRPL18/MRPS30/MRPL55/MRPL48/MRPL57/MRPS18A/RPL39L/MRPL34/MRPL10/MRPL1/MRPL44/MRPL9 |
| GOBP | CYTOPLASMIC PATTERN RECOGNITION RECEPTOR SIGNALING PATHWAY | 68 | 0.447603137 | 1.66794423 | 0.002165339 | 0.014317664 | 0.01000112 | 3551 | tags=37%, list=17%, signal=31% | IRF7/NOD2/UBE2N/C1QBP/NLRX1/IFIH1/IRAK2/PHB/IRAK1/TRIM15/UFD1/USP15/DDX60/CLPB/DHX58/BIRC3/PTPN22/ITCH/IKBKG/CASP8/MAP2K6/DDX58/SLC15A3/PHB2/NPLOC4 |
| GOBP | REGULATION OF DNA BINDING TRANSCRIPTION FACTOR ACTIVITY | 415 | 0.298338753 | 1.384501219 | 0.002163017 | 0.014316404 | 0.010000239 | 4261 | tags=31%, list=20%, signal=25% | S100A12/S100A9/ZC3H12A/TRIM14/WNT5A/NOD2/LTF/S100A8/TFDP1/UBE2N/MYD88/PRKCQ/SGK1/PYCARD/IRAK2/PRKCH/EZH2/LRP8/ID1/PPARGC1B/CFLAR/TRIM22/PPIA/CARD14/IRAK1/TLR2/STAT3/TRIM15/BUD31/AIM2/FOSL1/ADGRF1/LAMTOR5/RIPK4/NFKB1/IL1B/TRIM62/TRIM21/NFKBIE/COPS5/HMOX1/FANCD2/HCK/SYK/IL18RAP/RPS6KA4/TRAF1/IL6/RIPK3/CD36/TRAF3/LGALS9/COMMD1/ITGB2/CIB1/PIM1/MAD2L2/ITCH/IKBKG/PYDC1/CARD16/TFRC/NPM1/TRIM5/NLRC5/DDRGK1/DHX33/FLOT2/ADAM8/BRMS1/COMMD7/NLRP2/HIPK2/PARK7/HCLS1/MEN1/LPAR5/KEAP1/EOMES/HDAC2/TNF/CDKN2A/NLRC3/SMARCA4/RB1/XCL1/DDX58/PPRC1/SP100/CHUK/TRIM41/PSMD10/TRADD/PELI1/ICAM1/IRAK3/ARRB1/PHB2/TRIM25/BCL10/TCF3/BHLHE40/NFKBIB/NLRC4/HES6/KDM1A/TNFRSF4/CARD11/MAPK8/PPP2R5B/ZIC2/PARP10/NLRP3/SPHK1/NFKB2/JUP/OTULIN/IL10/FOXP3/MAPK14/SRI/MDFI/TRAF2/NLRP2B/FZD6/RBCK1/EGLN1/PRMT2/NR0B1 |
| GOBP | NEGATIVE REGULATION OF MUSCLE CONTRACTION | 20 | -0.61741808 | -1.874230543 | 0.002155247 | 0.014279044 | 0.009974143 | 2662 | tags=55%, list=13%, signal=48% | RGS2/DOCK5/DOCK4/BIN1/ARHGAP42/KCNMA1/PRKG1/TNNT1/ATP1A2/SOD1/ADRB2 |
| GOBP | DEVELOPMENT OF PRIMARY FEMALE SEXUAL CHARACTERISTICS | 96 | -0.367502785 | -1.582091889 | 0.002151679 | 0.014269479 | 0.009967462 | 4413 | tags=38%, list=21%, signal=30% | DMC1/ATM/TIPARP/GAS2/TAF4/UBE3A/ZNF830/SIRT1/ROBO2/KITLG/DACH1/LEP/GNRH1/SLIT2/LHCGR/ESR1/CTNNA1/PGR/SLIT3/FOXC1/ZFX/BMPR1B/ZFPM2/FZD4/IMMP2L/STAT5B/PLEKHA1/ACVR1B/NUPR1/PDGFRA/INHBB/FST/KIT/BCL2/SOD1/ANG |
| GOBP | POSITIVE REGULATION OF LYMPHOCYTE APOPTOTIC PROCESS | 15 | 0.679232423 | 1.837214067 | 0.002143776 | 0.014231116 | 0.009940665 | 3006 | tags=60%, list=14%, signal=51% | CD274/WNT5A/PRELID1/IDO1/ZC3H8/LGALS9/ADAM8/CCL5/BAX |
| GOMF | OLFACTORY RECEPTOR ACTIVITY | 86 | 0.422975801 | 1.640199325 | 0.002135812 | 0.014192267 | 0.009913528 | 9971 | tags=77%, list=48%, signal=40% | OR2L2/OR51E1/OR1E3/OR7C2/OR51E2/OR1F2P/OR1J2/OR11A1/OR2W1/OR1G1/OR2J3/OR5P3/OR7A17/OR4D2/OR51I1/OR2F1/OR1D2/OR1E1/OR4D1/OR51M1/OR7C1/OR1F1/OR10H2/OR2B2/OR2C1/OR51I2/OR2K2/OR3A1/OR5L2/OR1C1/OR1I1/OR2S2/OR8B8/OR2I1P/OR1J4/OR5V1/OR1Q1/OR7E24/OR10A4/OR8D2/OR1A2/OR2F2/OR5H1/OR10H1/OR51B5/OR8D1/OR2J2/OR10A5/OR2B3/OR51B4/OR2L13/OR7A5/OR5P2/OR12D3/OR10D3/OR14J1/OR51J1/OR52D1/OR5J2/OR51B6/OR9A1P/OR2H2/OR1A1/OR6A2/OR10C1/OR12D2 |
| GOBP | POSITIVE REGULATION OF MUSCLE CELL DIFFERENTIATION | 69 | -0.40624808 | -1.635012821 | 0.002116697 | 0.014079177 | 0.009834533 | 5524 | tags=48%, list=26%, signal=35% | CAMK1/MYLK3/IGF1/RBM24/TRIM32/TBX1/MAMSTR/AKAP6/CTNNB1/KAT2A/PARP2/GPER1/EFEMP2/SETD3/SIRT1/ABL1/BMP4/RBM4/SHOX2/EDN1/CTNNA1/GREM1/MEF2C/MYOCD/CYP26B1/BNIP2/CDON/PIAS1/KIT/BCL2/EFNB2/MEF2A/BOC |
| GOBP | EMBRYONIC SKELETAL JOINT MORPHOGENESIS | 10 | -0.741985027 | -1.858334601 | 0.002098826 | 0.013974143 | 0.009761165 | 4184 | tags=80%, list=20%, signal=64% | CTNNB1/BMP4/SHOX2/NOG/HOXA11/BMP7/OSR1/OSR2 |
| GOBP | PIGMENT BIOSYNTHETIC PROCESS | 55 | 0.465645408 | 1.688834519 | 0.002093287 | 0.013951093 | 0.009745064 | 4573 | tags=51%, list=22%, signal=40% | CDH3/WNT5A/SLC7A11/SRRD/GART/SHMT2/ALAS1/APRT/HMBS/HPRT1/GPR143/FECH/CTNS/GIPC1/COX10/PMEL/SLC25A39/GMPS/UROS/FXN/TSPO/TYRP1/OPN3/CPOX/ATP5IF1/TYR/PAICS/SLC45A2 |
| GOCC | STRIATED MUSCLE THIN FILAMENT | 20 | -0.617826997 | -1.87547185 | 0.002089606 | 0.013940389 | 0.009737587 | 2023 | tags=55%, list=10%, signal=50% | TMOD2/ACTA1/TNNC2/FHOD3/TNNT1/TPM2/TNNC1/TNNI2/LMOD1/TPM1/TMOD1 |
| GOBP | MATURATION OF 5 8S RRNA FROM TRICISTRONIC RRNA TRANSCRIPT SSU RRNA 5 8S RRNA LSU RRNA | 24 | 0.607427348 | 1.830322735 | 0.002088142 | 0.013940389 | 0.009737587 | 4612 | tags=58%, list=22%, signal=45% | WDR12/EXOSC3/BOP1/NOP14/RPP40/EXOSC2/ERI1/PES1/ERI2/ABT1/RRS1/EXOSC8/NOL9/URB1 |
| GOMF | FMN BINDING | 15 | 0.679669441 | 1.838396129 | 0.002078319 | 0.013892682 | 0.009704263 | 3024 | tags=53%, list=14%, signal=46% | PNPO/POR/NOS2/MTRR/NDUFV1/NDOR1/PPCDC/DUS2 |
| GOBP | PYRIMIDINE CONTAINING COMPOUND BIOSYNTHETIC PROCESS | 37 | 0.52968842 | 1.763078953 | 0.002068904 | 0.01384352 | 0.009669923 | 1723 | tags=30%, list=8%, signal=27% | TYMP/UPP1/TK1/UCK2/NME1/CTPS1/TYMS/CMPK2/DTYMK/PUDP/UMPS |
| GOBP | DORSAL VENTRAL NEURAL TUBE PATTERNING | 19 | -0.629782651 | -1.864369067 | 0.002058352 | 0.013786649 | 0.009630197 | 5031 | tags=53%, list=24%, signal=40% | FOXA1/TBC1D32/PRKACB/TCTN1/BMP4/TULP3/PTCH1/WDR19/GLI2/GLI3 |
| GOBP | MORPHOGENESIS OF EMBRYONIC EPITHELIUM | 140 | -0.337220691 | -1.526403358 | 0.002055417 | 0.013780728 | 0.009626061 | 3524 | tags=34%, list=17%, signal=28% | MIB1/IFT172/VEGFC/IFT52/SETD2/PRICKLE1/TCTN1/ABL1/BMP4/WNT7B/IRX2/TFAP2A/LMO4/SOX4/STK3/NOG/DVL2/SOX8/FZD3/JAG2/TWIST1/TULP3/LRP6/ARHGAP35/FGFR2/GREM1/SEC24B/GDF7/NPHP3/ALDH1A2/SKI/WNT2/TGFB1I1/IFT122/SDC4/BMP7/OSR1/PTCH1/TSC1/IRX1/LAMA5/DLC1/GLI2/AR/WNT2B/COBL/GATA3 |
| GOMF | RNA POLYMERASE II CORE PROMOTER SEQUENCE SPECIFIC DNA BINDING | 18 | 0.654925614 | 1.857264611 | 0.002052629 | 0.013775788 | 0.009622611 | 1000 | tags=39%, list=5%, signal=37% | STAT1/HDAC1/MACROH2A1/H2AZ1/RUVBL2/EZH2/CEBPB |
| GOBP | POSITIVE REGULATION OF METAPHASE ANAPHASE TRANSITION OF CELL CYCLE | 15 | 0.68020203 | 1.839836697 | 0.00204559 | 0.013742274 | 0.009599201 | 3150 | tags=53%, list=15%, signal=45% | DLGAP5/ANAPC7/CDT1/ESPL1/MAD2L1BP/ANAPC11/RB1/CDC23 |
| GOBP | NEGATIVE REGULATION OF PROTEIN POLYMERIZATION | 75 | -0.4027637 | -1.6534178 | 0.002041619 | 0.013729329 | 0.009590158 | 2440 | tags=32%, list=12%, signal=28% | SLIT2/KANK4/ADD1/TMOD2/TMSB15B/VILL/DYRK1A/PFN2/CLIP3/SPTAN1/MAP2/SNCA/SCIN/FHOD3/SVIL/EPS8/SPTBN2/MYADM/ADD3/LMOD1/TMOD1/GSN/KANK1/SPTBN1 |
| GOBP | MULTICELLULAR ORGANISM GROWTH | 128 | -0.341381293 | -1.520225495 | 0.002025843 | 0.013636886 | 0.009525585 | 3677 | tags=32%, list=18%, signal=27% | PIK3CA/SOCS2/SOS1/MBD5/RAI1/PPP1R13L/CLIC4/MFSD2A/APP/GPAM/GNAS/ATRX/FTO/WWTR1/STAT5A/DIO3/ETNK2/EN1/ADD1/WDR48/ADRB1/SELENOM/PLAG1/TNS2/ZFP36L1/GHR/KLF2/ZFX/ADARB1/NPY1R/PTCH1/STAT5B/PLEKHA1/SPTBN2/SLITRK6/BBS2/AR/NOTCH2/BCL2/SOD1/ADRB2 |
| GOBP | REGULATION OF CD4 POSITIVE ALPHA BETA T CELL DIFFERENTIATION | 48 | 0.482818749 | 1.706206901 | 0.002014074 | 0.013571262 | 0.009479746 | 3986 | tags=44%, list=19%, signal=35% | ZC3H12A/NFKBIZ/IL4R/CD83/SASH3/JAK3/IFNG/SOCS1/CBFB/IL12B/LGALS9/CCL19/IL23A/CD86/NFKBID/ANXA1/HLA-DRA/IL12RB1/NLRP3/CD80/FOXP3 |
| GOBP | REGULATION OF BONE MINERALIZATION | 70 | -0.417814812 | -1.688622518 | 0.001975646 | 0.013325695 | 0.009308213 | 4360 | tags=54%, list=21%, signal=43% | ACVR2A/NELL1/KL/BCOR/ECM1/PKDCC/TMEM119/S1PR1/RFLNB/ACVR2B/BMP2/BMP6/ATRAID/BMP4/ADGRV1/TFAP2A/ENPP1/DDR2/ATP2B1/ANO6/TWIST1/OMD/P2RX7/GPM6B/GREM1/MGP/BMPR1A/PTN/MEF2C/SLC8A1/BMPR1B/SMAD3/BMP7/OSR1/NBR1/ANKH/OSR2/ADRB2 |
| GOBP | CELL FATE DETERMINATION | 40 | -0.504158824 | -1.828921917 | 0.001973332 | 0.01332346 | 0.009306652 | 3069 | tags=32%, list=15%, signal=28% | PRRX1/BMP4/HES1/JAG1/NTF4/EBF2/KLF4/MEF2C/GATA2/PTCH1/CYP26B1/NOTCH2/GATA3 |
| GOCC | RIBONUCLEASE P COMPLEX | 13 | 0.707064823 | 1.83192672 | 0.001973123 | 0.01332346 | 0.009306652 | 4057 | tags=69%, list=19%, signal=56% | HSD17B10/TRMT10C/RPP40/POP5/POP4/POP1/POP7/RPP30/RPP38 |
| GOBP | PYRIMIDINE CONTAINING COMPOUND METABOLIC PROCESS | 80 | 0.419484474 | 1.608415566 | 0.001968299 | 0.013316249 | 0.009301615 | 1935 | tags=24%, list=9%, signal=22% | TYMP/UPP1/TK1/UCK2/NME1/NT5C3A/CTPS1/TYMS/ENTPD7/CMPK2/ACP3/APOBEC3B/DTYMK/TDG/PUDP/RRM1/UMPS/DCTPP1/SMUG1 |
| GOBP | REGULATION OF CELL CYCLE ARREST | 103 | 0.401713092 | 1.611745552 | 0.001954754 | 0.013237939 | 0.009246914 | 3679 | tags=34%, list=18%, signal=28% | CCNB1/CDK1/TFDP1/FOXM1/PCNA/AURKA/PLK2/SFN/E2F8/MIF/CNOT11/CDK5/SLC25A33/CDKN1A/MYBBP1A/CRLF3/PLK3/RRP8/CNOT1/ADAM10/CHEK2/TRIAP1/BRCA1/CDK2/CRADD/CDC25C/PML/BAX/GADD45A/PRMT1/MDM2/CDK9/CNOT6/PPP2R5B/CDK4 |
| GOBP | MODULATION BY SYMBIONT OF ENTRY INTO HOST | 46 | 0.486465132 | 1.69105087 | 0.001952588 | 0.013236617 | 0.009245991 | 4424 | tags=52%, list=21%, signal=41% | KRT6A/TRIM14/TMPRSS4/TRIM22/CXCL8/IFITM1/P4HB/TRIM62/TRIM21/FCN1/TRIM10/IFITM3/LGALS9/TRIM5/EXOC2/SNX3/CIITA/TRIM25/IFITM2/TRIM11/NECTIN2/PTX3/LY6E/FCN3 |
| GOBP | NUCLEOBASE CONTAINING SMALL MOLECULE CATABOLIC PROCESS | 47 | 0.491848441 | 1.71947111 | 0.001949528 | 0.013229219 | 0.009240824 | 4881 | tags=40%, list=23%, signal=31% | TYMP/UPP1/PNP/NT5C3A/AHCY/NUDT15/ENTPD7/APOBEC3B/HPRT1/NUDT5/XDH/NUDT1/ADA2/DERA/GSK3A/NT5C/APOBEC3C/ENTPD3/ENTPD5 |
| GOBP | DEVELOPMENTAL GROWTH INVOLVED IN MORPHOGENESIS | 218 | -0.305030893 | -1.456140475 | 0.001948686 | 0.013229219 | 0.009240824 | 4795 | tags=37%, list=23%, signal=29% | KIF26B/SEMA4C/SEMA3C/WNT11/MAPT/DDR1/SHTN1/LRP1/HDAC6/USP9X/SEMA3A/DVL1/MED1/S1PR1/RND2/CTNNB1/RUFY3/SMURF1/PRKN/SEMA6D/NEDD4L/NRP1/TNN/VCL/FGF1/SYT1/APP/ABL1/SIN3A/BMP4/WNT7B/CXCL12/RTN4R/SEMA3E/GOLGA4/YAP1/NKD1/MACF1/L1CAM/SLIT2/SPART/SEMA4G/AUTS2/SEMA6A/FMN1/ESR1/FGF13/SEMA3B/PPP3CB/NDN/RYK/SLIT3/APOE/LRP6/PTPRS/FGFR2/DCLK1/ULK2/LAMB2/FN1/GSK3B/MAP2/SYT17/NTN1/IQGAP1/RTN4/LZTS2/TGFBR2/DPYSL2/NIN/SEMA3G/TMEM108/MAGI2/SPRY1/DBN1/SPRY2/POSTN/ALCAM/MAP1B/COBL |
| GOBP | DENDRITIC CELL MIGRATION | 29 | 0.574913411 | 1.80409667 | 0.001938834 | 0.013183284 | 0.009208737 | 3377 | tags=48%, list=16%, signal=41% | CXCR2/CXCR4/C1QBP/CCR7/SLAMF8/LGALS9/CCL19/CCR5/GPR183/DOCK8/EXT1/CCL5/CCR1/CCR2 |
| GOBP | NON RECOMBINATIONAL REPAIR | 98 | 0.404076882 | 1.604165534 | 0.001934519 | 0.013167271 | 0.009197551 | 5699 | tags=49%, list=27%, signal=36% | PARP9/H2AX/UBE2N/DTX3L/POLB/AUNIP/PSMD14/SIRT7/PNKP/XRCC6/XRCC5/NSD2/POLQ/MAD2L2/PAXX/RNF168/PRKDC/HMCES/BRCC3/UBE2V2/BABAM2/XRCC4/HMGB2/LIG4/BRCA1/ERCC8/RNF138/ERCC1/RNF8/CYREN/REXO4/RAD50/TFIP11/PARP3/WRAP53/H4C4/H4C9/ZBTB7A/PRPF19/BABAM1/H4C2/NUDT16L1/ERCC6/MLH1/KMT5C/H4C5/RBBP8/TP53BP1 |
| GOBP | TETRAHYDROFOLATE METABOLIC PROCESS | 17 | 0.65455322 | 1.826376872 | 0.001928745 | 0.013141287 | 0.009179402 | 1973 | tags=53%, list=9%, signal=48% | GCH1/TYMS/GART/SHMT2/ATIC/MTHFD1/MTHFD2/MTHFD1L/DHFR |
| GOBP | ANTIGEN PROCESSING AND PRESENTATION OF ENDOGENOUS PEPTIDE ANTIGEN | 17 | 0.654771387 | 1.826985617 | 0.001928745 | 0.013141287 | 0.009179402 | 5675 | tags=76%, list=27%, signal=56% | TAP1/TAP2/HLA-E/TAPBP/IDE/HLA-DRA/B2M/HLA-F/HLA-B/HLA-G/HLA-A/HLA-C/ERAP2 |
| GOBP | CHROMOSOME CONDENSATION | 46 | 0.486684834 | 1.691814596 | 0.001923662 | 0.013133291 | 0.009173816 | 1573 | tags=22%, list=8%, signal=20% | CCNB1/NUSAP1/CDCA5/NCAPH/NCAPG/TOP2A/AIFM1/NCAPG2/SMC2/SMC4 |
| GOBP | REGULATION OF DNA REPAIR | 126 | 0.382456806 | 1.58059555 | 0.00192056 | 0.013125453 | 0.009168341 | 3696 | tags=34%, list=18%, signal=28% | PARP9/H2AX/UBE2N/FOXM1/TIGAR/TIMELESS/PCNA/DTX3L/TRIP12/RAD51/PPP4C/PARPBP/OTUB2/PPP4R2/AUNIP/RAD51AP1/SIRT7/PNKP/FIGNL1/CGAS/NSD2/POLQ/MAD2L2/RNF168/PRKDC/BRCC3/UBE2V2/BABAM2/BRCA1/TMEM161A/ERCC8/PML/SIRT6/PARP1/RNF8/UBQLN4/CDK9/CYREN/KDM1A/RMI2/WAS/OTUB1/TFIP11 |
| GOBP | NEGATIVE REGULATION OF ACTIVATED T CELL PROLIFERATION | 14 | 0.693068692 | 1.831143473 | 0.001918082 | 0.013121869 | 0.009165838 | 3986 | tags=57%, list=19%, signal=46% | CD274/ARG1/LGALS9/LILRB4/CRTAM/SCRIB/CASP3/FOXP3 |
| GOBP | POSITIVE REGULATION OF EPIDERMIS DEVELOPMENT | 30 | 0.565392498 | 1.783836285 | 0.001917497 | 0.013121869 | 0.009165838 | 2653 | tags=47%, list=13%, signal=41% | PPARD/MACROH2A1/PRKCH/TMEM79/TRIM16/ZBED2/IL20/SFN/CYP27B1/SULT2B1/OVOL2/ETV4/MACROH2A2/PLAAT4 |
| GOBP | POSITIVE REGULATION OF EPIDERMAL CELL DIFFERENTIATION | 25 | 0.595655888 | 1.794393456 | 0.001916763 | 0.013121869 | 0.009165838 | 2653 | tags=48%, list=13%, signal=42% | MACROH2A1/PRKCH/TRIM16/ZBED2/IL20/SFN/CYP27B1/SULT2B1/OVOL2/ETV4/MACROH2A2/PLAAT4 |
| GOMF | U3 SNORNA BINDING | 10 | 0.75205488 | 1.816145509 | 0.001914675 | 0.013121869 | 0.009165838 | 2998 | tags=70%, list=14%, signal=60% | ISG20/TSR1/SNU13/XRCC5/RRP9/PRKDC/TBL3 |
| GOBP | REGULATION OF CARDIAC MUSCLE CELL PROLIFERATION | 37 | -0.513224277 | -1.826275228 | 0.001903024 | 0.013072101 | 0.009131074 | 4035 | tags=51%, list=19%, signal=41% | KCNK2/SAV1/PTEN/VGLL4/ERBB4/JARID2/YAP1/NOG/FGFR2/BMPR1A/MEF2C/RBP4/WNT2/RBPJ/MAPK1/TGFBR2/ZFPM2/MEIS1/TGFBR3 |
| GOBP | POSITIVE REGULATION OF VIRAL PROCESS | 89 | 0.412186399 | 1.609012961 | 0.001896035 | 0.013037421 | 0.009106849 | 4419 | tags=42%, list=21%, signal=33% | KPNA2/TMPRSS4/POLR2H/PPIA/POLR2F/TOP2A/VAPA/IFIT1/TARBP2/NELFCD/PPIH/P4HB/TRIM21/PDE12/POLR2L/LGALS9/ADAR/SRPK1/POLR2G/POLR2D/LARP1/POLR2E/CCL5/VPS37B/CLEC4G/NELFE/CDK9/POLR2I/PC/TRIM11/SUPT4H1/TMEM39B/CHMP2A/FKBP6/POLR2J/TMEM250/CD28 |
| GOCC | U2 TYPE SPLICEOSOMAL COMPLEX | 91 | 0.414199195 | 1.617588039 | 0.001896035 | 0.013037421 | 0.009106849 | 2861 | tags=32%, list=14%, signal=28% | EIF4A3/PPIL1/SNRPG/SNRPF/SNRPD1/LSM2/BUD31/TXNL4A/SF3B3/LSM7/SNRPD3/EFTUD2/SF3B5/SNU13/SNRPA1/MAGOHB/SNRPC/SF3B6/LSM5/LSM4/SNRPB/AQR/LUC7L2/PRPF31/PRPF38A/LSM6/ISY1/PRPF4/PLRG1 |
| GOMF | PROTEIN FOLDING CHAPERONE | 33 | 0.556914037 | 1.810433111 | 0.001895052 | 0.013037421 | 0.009106849 | 2945 | tags=36%, list=14%, signal=31% | CCT5/HSPA5/CCT2/CCT3/HSPA14/HSPA9/CCT7/HSPA8/DNAJB1/TCP1/CCT6A/CCT8 |
| GOBP | T CELL MEDIATED CYTOTOXICITY | 43 | 0.510932055 | 1.752696745 | 0.001877598 | 0.012950417 | 0.009046075 | 4624 | tags=60%, list=22%, signal=47% | CTSC/RAB27A/CYRIB/HPRT1/PRF1/IL12B/IL7R/RIPK3/IL23A/FADD/HLA-E/XCL1/KLRD1/CD1B/HLA-DRA/IL12RB1/B2M/HLA-F/HLA-B/LILRB1/NECTIN2/HLA-G/EMP2/CD1D/PTPRC/AGER |
| GOBP | DNA UNWINDING INVOLVED IN DNA REPLICATION | 16 | 0.680841596 | 1.885317645 | 0.001874717 | 0.012943831 | 0.009041475 | 4208 | tags=69%, list=20%, signal=55% | MCM6/RAD51/MCM4/MCM2/SSBP1/MCM7/BLM/TWNK/HMGA1/RECQL4/RPA1 |
| GOBP | VESICLE LOCALIZATION | 216 | 0.341966464 | 1.501512166 | 0.001857227 | 0.01283627 | 0.008966342 | 4463 | tags=34%, list=21%, signal=27% | CDH3/CTSC/MREG/SERPINA1/MX1/TGFA/RAB11A/RAB27A/SHROOM2/AREG/MKKS/BORCS5/TFG/MYO1E/BLOC1S2/FBXW11/AP3D1/SNAP29/MX2/CNIH1/RAB1A/CDK5/BICDL2/TRAPPC2L/YKT6/GPR143/AP1M2/MYO5A/PDCD6/LIMK2/MYO1F/TRAPPC5/ITGA4/MYO1B/NDE1/AP3B1/PREB/NSF/SEC24C/TOR1A/PPP6R1/IKBKG/PPP6C/NAPA/WASL/SEC13/VAMP7/VPS33B/SNAPIN/NDEL1/MYO1H/MYO5B/BLOC1S4/KIF1B/WIPI1/CEP19/LMAN1/MYO19/PPP6R3/HTT/SCFD1/TMED9/TRAPPC1/DCTN2/KIFAP3/TRAPPC3/MYO6/BLOC1S6/MYO1G/MYO7A/MAP4K2/DNM2/SEC23IP |
| GOMF | OXIDOREDUCTASE ACTIVITY ACTING ON THE CH NH GROUP OF DONORS | 27 | 0.587984615 | 1.827067955 | 0.001855141 | 0.012835054 | 0.008965493 | 5197 | tags=67%, list=25%, signal=50% | AASS/IL4I1/SMOX/MTHFD1/MTHFD2/ALDH4A1/MTHFD1L/DHFR/PAOX/MTHFR/PIPOX/ETFDH/PYCR1/SARDH/MTHFD2L/DMGDH/PYCR3/BLVRB |
| GOBP | REGULATION OF RESPONSE TO DNA DAMAGE STIMULUS | 213 | 0.338885463 | 1.487116401 | 0.001850012 | 0.012812766 | 0.008949924 | 3696 | tags=31%, list=18%, signal=26% | PARP9/H2AX/UBE2N/FOXM1/TIGAR/TIMELESS/PCNA/DTX3L/TRIP12/SMYD2/RAD51/PPP4C/PARPBP/DDIAS/ZMPSTE24/OTUB2/PPP4R2/AUNIP/RAD51AP1/EEF1E1/MIF/MYC/SIRT7/PNKP/FIGNL1/CGAS/BID/BCL2L12/NSD2/POLQ/MAD2L2/RNF168/FBXO5/PRKDC/WDR76/CHEK2/BRCC3/UBE2V2/BABAM2/TRIAP1/THOC5/BRCA1/FBXO4/TMEM161A/ERCC8/PML/ARMT1/ING2/ERCC1/SIRT6/PARP1/MDM2/RNF8/UBQLN4/RFWD3/PSMD10/CDK9/PRKCD/CYREN/KDM1A/MCL1/RMI2/WAS/OTUB1/CDKN2D/TFIP11 |
| GOBP | POSITIVE REGULATION OF ANION TRANSMEMBRANE TRANSPORT | 59 | -0.431708694 | -1.681458262 | 0.001846521 | 0.012801783 | 0.008942253 | 4280 | tags=42%, list=21%, signal=34% | CFTR/ABCB1/APPL1/C2CD5/RAP1A/ACSL1/C3/TCAF1/CLTRN/INSR/ADIPOQ/RHOQ/CLIP3/ITGB1/GPC3/OSBPL8/BRAF/KLF15/IRS1/PIK3R1/AZIN1/CREBL2/SORBS1/IRS2/MEF2A |
| GOCC | NUCLEAR REPLICATION FORK | 34 | 0.53838541 | 1.767417802 | 0.00184572 | 0.012801783 | 0.008942253 | 4287 | tags=47%, list=21%, signal=37% | TIMELESS/PCNA/MCM10/RPA3/POLD2/MCM3/WDHD1/POLA2/PRIM1/PLRG1/POLD4/SMARCA5/POLD3/RPA1/TIPIN/POLD1 |
| GOBP | POSITIVE REGULATION OF PROTEIN MODIFICATION BY SMALL PROTEIN CONJUGATION OR REMOVAL | 132 | 0.372836842 | 1.545488437 | 0.001829312 | 0.012708702 | 0.008877234 | 3843 | tags=34%, list=18%, signal=28% | ZC3H12A/CDC20/NMI/NOD2/DCUN1D5/UBE2C/UBE2N/VCP/FANCI/UBE2L3/HSPA5/CENPX/GNL3/TBC1D7/UBE2S/TOLLIP/CHFR/AIMP2/RAB1A/UBE2D1/RBX1/PLK1/BIRC3/CENPS/PDCD6/PHF23/COMMD1/PTPN22/SAE1/HSPBP1/LAPTM5/TNIP1/BRCA1/FBXO4/PSMD10/PELI1/CDK9/ARRB1/BCL10/CTR9/KDM1A/DCUN1D3/HDAC3/GSK3A/SPHK1 |
| GOBP | CD8 POSITIVE ALPHA BETA T CELL ACTIVATION | 25 | 0.600187557 | 1.808044959 | 0.001820122 | 0.012657948 | 0.008841781 | 3674 | tags=52%, list=18%, signal=43% | CD274/IRF1/SOCS1/CBFB/PTPN22/WDFY4/LILRB4/HLA-E/CRTAM/CLEC4A/EOMES/XCL1/LILRB1 |
| GOBP | LIPOPOLYSACCHARIDE MEDIATED SIGNALING PATHWAY | 56 | 0.475991379 | 1.729021407 | 0.00181129 | 0.012609583 | 0.008807998 | 4807 | tags=46%, list=23%, signal=36% | LTF/MYD88/PTAFR/IRAK2/IRAK1/TLR2/IL1B/CCL2/LYN/CD6/HCK/CD36/PTPN22/CARD16/TRIM5/LY96/CCL5/TNF/BCL10/LY86/LILRA2/MAPK14/MALT1/TRIB1/MAPK3/TICAM1 |
| GOBP | NEGATIVE REGULATION OF LYMPHOCYTE MEDIATED IMMUNITY | 44 | 0.512815641 | 1.771735983 | 0.001793874 | 0.012501293 | 0.008732355 | 5251 | tags=59%, list=25%, signal=44% | SERPINB4/NOD2/SUSD4/ARG1/SERPINB9/IL7R/LGALS9/LILRB4/HLA-E/XCL1/KLRD1/CLEC4G/CD96/HLA-F/HLA-B/LILRB1/HLA-G/FOXP3/PARP3/CR1/PTPRC/TBX21/ARRB2/IFNA2/HLA-A/C4BPA |
| GOBP | T CELL CHEMOTAXIS | 23 | 0.61224284 | 1.817157438 | 0.001772415 | 0.012364573 | 0.008636854 | 3616 | tags=65%, list=17%, signal=54% | WNT5A/S100A7/CXCL13/CXCL16/CXCL10/ADAM17/GPR183/OXSR1/ADAM10/CXCL11/PIK3CD/CCL5/XCL1/CCR2/TMEM102 |
| GOBP | CELLULAR TRANSITION METAL ION HOMEOSTASIS | 109 | 0.399821708 | 1.617649946 | 0.001768631 | 0.012350998 | 0.008627372 | 2252 | tags=24%, list=11%, signal=21% | LCN2/S100A9/LTF/S100A8/ATOX1/HMOX2/GLRX3/SCO2/SLC39A6/ATP6V0D1/HIF1A/ATP6V1G1/LCK/ABCB6/AP3D1/NUBP1/IFNG/SCO1/MYC/HMOX1/SLC31A1/SLC39A4/AP3B1/TFRC/CUL1/ATP6V1A |
| GOBP | UREA CYCLE | 11 | 0.740092993 | 1.83665545 | 0.001761855 | 0.012316483 | 0.008603262 | 1618 | tags=45%, list=8%, signal=42% | NMRAL1/ARG1/ASL/SLC25A15/NAGS |
| GOBP | CELLULAR MODIFIED AMINO ACID BIOSYNTHETIC PROCESS | 43 | 0.511322541 | 1.754036265 | 0.001761161 | 0.012316483 | 0.008603262 | 3574 | tags=47%, list=17%, signal=39% | SLC7A11/GCH1/ALDH7A1/CHAC1/CHAC2/PLSCR1/CNDP2/GART/ATIC/MTHFD1/GGCT/GCLM/MTHFD1L/DHFR/PARK7/MGST2/PTDSS2/GCLC/PLOD2/OPLAH |
| GOBP | MULTI ORGANISM LOCALIZATION | 65 | 0.460423372 | 1.711195073 | 0.001760959 | 0.012316483 | 0.008603262 | 4816 | tags=49%, list=23%, signal=38% | RAN/KPNA2/DYNLT1/NUP37/CTSL/NUP210/TRIM15/IFIT1/NUP88/NUP50/VPS37A/RAE1/SEC13/KPNB1/BST2/NUP155/VPS37B/NDC1/UBAP1/NUP188/NUP85/KPNA3/NUP205/MVB12A/NUP58/NUP107/NUP93/POM121/NUP160/CLEC4M/NUP35/NUP42 |
| GOMF | TRNA SPECIFIC RIBONUCLEASE ACTIVITY | 14 | 0.694892228 | 1.835961405 | 0.001754148 | 0.012301006 | 0.008592451 | 4342 | tags=71%, list=21%, signal=57% | ELAC2/RPP40/POP5/POP4/TSEN34/POP1/POP7/RPP30/RPP38/TSEN2 |
| GOBP | NUCLEOSIDE CATABOLIC PROCESS | 32 | 0.536823524 | 1.732991664 | 0.001746828 | 0.012262479 | 0.00856554 | 1345 | tags=25%, list=6%, signal=23% | TYMP/UPP1/PNP/NT5C3A/AHCY/APOBEC3B/HPRT1/XDH |
| GOBP | IMPORT INTO NUCLEUS | 151 | 0.370360405 | 1.560971607 | 0.001745545 | 0.012262479 | 0.00856554 | 4021 | tags=36%, list=19%, signal=30% | ZC3H12A/RAN/KPNA2/SNRPG/MMP12/SNRPF/FERMT1/SNRPD1/ZPR1/HEATR3/ECT2/STAT3/IPO4/SNRPD3/NUP88/IFNG/NUP62CL/BAG3/GEMIN6/GEMIN7/CDKN1A/SYK/NOLC1/NUP50/CD36/HTATIP2/HIKESHI/SNRPB/NXT1/E2F3/CSE1L/ELAVL1/SEC13/TARDBP/KPNB1/PIK3R2/HCLS1/NUP155/PML/GEMIN2/PKIA/NUP188/PHB2/PRKCD/PRKAG1/NUP85/KPNA3/NUTF2/HDAC3/NUP58/CDH1/JUP/NUP107/MAPK14/NUP93 |
| GOBP | NEGATIVE REGULATION OF TRANSFORMING GROWTH FACTOR BETA RECEPTOR SIGNALING PATHWAY | 79 | -0.391081023 | -1.615806334 | 0.001734134 | 0.012198863 | 0.008521103 | 5707 | tags=47%, list=27%, signal=34% | PBLD/SMAD2/CIDEA/FBN1/NRROS/STUB1/BAMBI/LEMD3/RASL11B/PEG10/PDPK1/HTRA1/UBC/SMURF1/LDLRAD4/SLC2A10/HTRA4/SIRT1/SPRED2/GLG1/CD109/SPRED1/SNX25/SMURF2/PMEPA1/PPARA/SKI/SMAD3/TGFB1I1/TGFBR2/ASPN/IL17RD/SPRY1/SPRY2/CAV2/CAV1/TGFBR3 |
| GOBP | NEGATIVE REGULATION OF INTERLEUKIN 1 BETA PRODUCTION | 26 | 0.599233086 | 1.836048568 | 0.00170792 | 0.012027053 | 0.008401091 | 3769 | tags=46%, list=18%, signal=38% | ZC3H12A/SERPINB1/GSTP1/CARD16/LILRB4/TREM2/PML/CPTP/NLRP7/CARD18/ERRFI1/NLRP3 |
| GOBP | REGULATION OF ALPHA BETA T CELL DIFFERENTIATION | 64 | 0.454484733 | 1.684572227 | 0.001699413 | 0.0119797 | 0.008368014 | 4101 | tags=45%, list=20%, signal=37% | ZC3H12A/NFKBIZ/PNP/IL4R/CD83/PRDM1/SASH3/AP3D1/JAK3/IFNG/SOCS1/CBFB/IL12B/SYK/LGALS9/CCL19/AP3B1/IL23A/LILRB4/CD86/NFKBID/ANXA1/HLA-DRA/IL12RB1/ZAP70/NLRP3/CD80/FOXP3/ZNF683 |
| GOCC | CAJAL BODY | 55 | 0.472660798 | 1.714278414 | 0.001694572 | 0.011958128 | 0.008352946 | 2845 | tags=38%, list=14%, signal=33% | ISG20/NHP2/HNRNPA2B1/ZPR1/TRIM22/NOP10/OIP5/EFTUD2/LSM10/NOP58/SNRPC/ZC3H8/NOLC1/FAM118B/GAR1/PRPF31/ELL/EAF1/DKC1/PRPF4/CDK2 |
| GOBP | REGULATION OF TRANS SYNAPTIC SIGNALING | 393 | -0.257675445 | -1.31607766 | 0.001691462 | 0.011948726 | 0.008346378 | 3924 | tags=25%, list=19%, signal=20% | RASGRF2/EGR2/RAP1B/ZDHHC3/LRRC4C/GPER1/SQSTM1/PRKN/GRIN3A/VAMP2/PTEN/SHANK2/LAMA2/PRKCE/RAP1A/SLC24A1/SLC12A2/RELN/TAC1/ZDHHC2/SYT1/TSHZ3/IGSF11/PXK/APP/ABL1/PREPL/LGI1/PACSIN2/CBLN1/PRRT2/NRG3/STXBP5/FAM107A/LRRK2/NPTN/EDN1/ADIPOQ/PLCB1/SNCG/RAB26/NTF4/CLSTN1/NLGN1/SNCAIP/CACNB2/PNKD/EGFR/CNTN4/BCHE/NPTX1/PRKAR2B/SHANK3/PPP3CB/NPY5R/RHOT1/APOE/RNF19A/PLCL2/PTPRS/NTF3/MECP2/AKAP12/ITGB1/NTRK2/NISCH/GSK3B/ROR2/PTN/MEF2C/P2RX1/NPTX2/CLSTN2/PINK1/PRKCB/PLCB4/ADRA1A/RPL22/MAPK1/EIF4EBP2/RAPGEF2/STAR/AKAP9/RAB3GAP1/ITPR3/ABHD6/MAP1A/ATP1A2/FYN/TMEM108/ADRA2A/GNAI1/DBN1/KIT/KCNMB4/MAP1B/ADRB2 |
| GOBP | DNA DAMAGE RESPONSE SIGNAL TRANSDUCTION BY P53 CLASS MEDIATOR | 105 | 0.398816495 | 1.601310426 | 0.00168637 | 0.011925302 | 0.008330016 | 3325 | tags=34%, list=16%, signal=29% | CCNB1/CDK1/TFDP1/FOXM1/PCNA/AURKA/SMYD2/PLK2/BCL3/SFN/E2F8/ZMPSTE24/EEF1E1/MIF/CNOT11/CDKN1A/PLK3/SESN2/BRCA2/BATF/CNOT1/CHEK2/TRIAP1/HIPK2/BRCA1/CDK2/CRADD/CDC25C/PML/BAX/GADD45A/PRMT1/MDM2/SP100/PSMD10/CNOT6 |
| GOBP | REGULATION OF GTPASE ACTIVITY | 447 | -0.251788156 | -1.30170917 | 0.001684773 | 0.011925302 | 0.008330016 | 3631 | tags=28%, list=17%, signal=24% | ALS2/SOS1/ALDH1A1/RDX/NRP1/PLXND1/RAP1A/IQSEC1/RABEP1/TIAM2/ASAP3/TBC1D8/AGAP9/CBLB/SRGAP3/ANKRD27/CPEB2/RASA4/AGAP4/BCAS3/ARHGEF1/STXBP5/TBC1D2B/BVES/LARS1/ARHGAP24/RTN4R/LRRK2/ARHGAP10/RASA3/RGS2/EFNA5/NET1/HACD3/AMOT/NGEF/S100A10/SYDE1/SLIT2/CHML/WNK1/PLCB1/DVL2/IPO5/F2R/ARHGEF15/EVI5/DOCK5/DOCK4/TBC1D15/FZD10/RAPGEF3/MET/ARHGAP20/NEDD9/ADRB1/RANBP3/ARHGEF6/ARHGAP26/RALGAPB/AGFG2/GIT2/ARHGAP12/ARFGAP3/PTK2/ARHGAP35/NTF3/AGAP1/TBC1D16/ITGB1/ARHGAP42/SH3BP4/STMN3/NTRK2/ARHGEF5/GSK3B/RICTOR/RAB11FIP2/DOCK7/AGAP11/SNX18/STARD13/IQGAP1/ARHGAP6/PRKG1/SBF2/DOCK1/PBXIP1/FERMT2/VAV3/ARHGAP29/SGSM2/ARHGEF12/TBC1D5/RAPGEF2/TBC1D24/RAB3GAP1/SRGAP1/BCL6/TSC1/PLXNA2/RALGAPA1/FGD4/RASAL2/GARNL3/BNIP2/FGD5/ELMOD1/SPRY1/DLC1/LRCH1/ARHGAP31/FAM13A/ARHGEF7/SPRY2/CAV2/ASAP2/MYO9A/SOD1/SRGAP2/ARHGEF10/BCAR3/TRAPPC6A/RABGAP1L/ARHGEF26 |
| GOBP | PROSTATE GLAND GROWTH | 10 | -0.749107315 | -1.876172689 | 0.001681153 | 0.011913485 | 0.008321762 | 4499 | tags=80%, list=22%, signal=63% | PSAP/UBE3A/PTEN/ESR1/PLAG1/FGFR2/PSAPL1/AR |
| GOCC | SAM COMPLEX | 13 | 0.712107346 | 1.844991341 | 0.001679608 | 0.011913485 | 0.008321762 | 4058 | tags=77%, list=19%, signal=62% | MTX2/HSPA9/MICOS10/MICOS13/MTX1/CHCHD3/IMMT/APOO/DNAJC11/SAMM50 |
| GOBP | REGULATION OF LYMPHOCYTE MIGRATION | 59 | 0.45931979 | 1.677755717 | 0.001666093 | 0.011831726 | 0.008264651 | 3616 | tags=41%, list=17%, signal=34% | C10orf99/WNT5A/CCL20/S100A7/PYCARD/CXCL13/CXCL10/CCL4/ADAM17/CCL2/ITGA4/RIPK3/CCL7/DOCK8/OXSR1/ADAM10/ADAM8/FADD/WASL/CCL5/XCL1/CCR2/GCSAM/TMEM102 |
| GOBP | TRANSITION METAL ION TRANSPORT | 121 | 0.38368457 | 1.578896897 | 0.001661857 | 0.011814136 | 0.008252365 | 3017 | tags=26%, list=14%, signal=22% | TCN1/LCN2/TMEM165/LTF/ATOX1/SLC39A6/FLVCR2/ATP6V0D1/ATP6V1B2/ATP6V1G1/ATP6V0B/SLC25A28/ABCB6/ATP6V1F/IFNG/ATP13A1/SLC39A2/ATP6V1D/SLC31A1/ATP6V1C1/STEAP4/SLC39A4/ATP6V1C2/TMEM163/TFRC/ATP6V1H/ATP6V1A/COX17/ATP6V0E1/STEAP3/SLC39A11 |
| GOBP | REGULATION OF MORPHOGENESIS OF A BRANCHING STRUCTURE | 48 | -0.465469539 | -1.749274969 | 0.001656414 | 0.011787912 | 0.008234047 | 3622 | tags=42%, list=17%, signal=35% | TACSTD2/LGR4/ABL1/BMP4/AGT/LRRK2/NTN4/BTBD7/SHOX2/NOG/SOX8/ESR1/PDGFA/FGFR2/GREM1/WNT2/RTN4/BMP7/AR/WNT2B |
| GOBP | POSITIVE REGULATION OF CELL CYCLE ARREST | 78 | 0.426191634 | 1.623946057 | 0.001655856 | 0.011787912 | 0.008234047 | 3325 | tags=35%, list=16%, signal=29% | CCNB1/CDK1/TFDP1/PCNA/AURKA/PLK2/SFN/E2F8/CNOT11/CDKN1A/MYBBP1A/CRLF3/PLK3/RRP8/CNOT1/CHEK2/TRIAP1/BRCA1/CDK2/CRADD/CDC25C/PML/BAX/GADD45A/PRMT1/MDM2/CNOT6 |
| GOBP | VASCULAR ASSOCIATED SMOOTH MUSCLE CELL MIGRATION | 20 | -0.625656139 | -1.899237945 | 0.001651997 | 0.011781436 | 0.008229523 | 2420 | tags=75%, list=12%, signal=66% | PAK1/ATP7A/PDGFB/LRP1/ADIPOQ/DOCK5/DOCK4/FGF9/DOCK7/MEF2C/IGFBP5/MYOCD/IQGAP1/PRKG1/TPM1 |
| GOMF | ORGANIC ANION TRANSMEMBRANE TRANSPORTER ACTIVITY | 168 | 0.365589166 | 1.561004241 | 0.001648019 | 0.01176556 | 0.008218434 | 5370 | tags=37%, list=26%, signal=28% | SLC6A14/SLC26A9/SLC25A5/SLC7A1/SLC7A11/SFXN1/ABCC1/SLC35B1/SLC16A1/SLC23A2/SLC7A5/SLC25A13/SLC25A15/SLC5A6/SLC2A1/SLC38A5/SLC25A10/SLC36A1/SLC25A25/SFXN2/SLCO4A1/SLC3A2/CD36/SLC4A11/SLC52A2/CTNS/SLC25A11/SLC9A3R1/SLC1A3/SLC26A4/SLC25A19/SLC19A1/SLC16A3/SFXN5/SLC38A9/MFSD10/SLC66A1/SLC38A7/SLC35B4/SLC6A20/SLC35D2/SLC23A1/SLC36A4/SLC6A1/SLC25A32/GJA1/SLC1A1/SLC1A5/SLC1A4/SLC25A12/ABCC5/SLC6A11/SLCO2A1/SLC15A4/SLC4A8/SLC6A6/SLC22A12/MPC2/SLCO2B1/SLC38A6/SLC7A8/SLC52A3 |
| GOBP | STRIATED MUSCLE CELL PROLIFERATION | 60 | -0.432209406 | -1.680275719 | 0.001644553 | 0.011753308 | 0.008209876 | 4040 | tags=45%, list=19%, signal=36% | TGFB2/KCNK2/ANGPT1/MSTN/SIX5/SAV1/PTEN/FOXC2/VGLL4/ERBB4/JARID2/YAP1/NOG/FOXC1/FGFR2/BMPR1A/DIPK2A/MEF2C/RBP4/WNT2/RBPJ/MAPK1/TGFBR2/ZFPM2/EPHB1/MEIS1/TGFBR3 |
| GOBP | REGULATION OF ATTACHMENT OF SPINDLE MICROTUBULES TO KINETOCHORE | 13 | 0.713967474 | 1.849810726 | 0.001630769 | 0.011667205 | 0.008149731 | 1894 | tags=54%, list=9%, signal=49% | CCNB1/AURKB/NEK2/SPAG5/ECT2/RACGAP1/RCC2 |
| GOBP | TRANSLATIONAL INITIATION | 180 | -0.314831821 | -1.47649308 | 0.001627919 | 0.01165923 | 0.008144161 | 3736 | tags=37%, list=18%, signal=30% | RPL24/RPS11/RPS5/RPS9/MIF4GD/RPL41/RPL23A/RPS25/RPS15A/MTIF3/RPS15/CSDE1/RBM4/TPR/RPL13A/RPL32/EIF1B/RPL7A/RPS27/RPL31/RPL18/EIF2A/RPL27/RPS8/RPL4/RPL12/RPL9/METTL3/RPS6/RPL13/RPL10A/RPL11/RPL37/RPL14/RPS4X/HABP4/RPLP2/RPS14/RPLP1/RPL8/RPS17/EIF4B/RPS24/RPL22/RPS23/EIF4EBP2/EIF3E/EIF3H/RPL23/EIF4A2/RPL38/CTIF/RPS20/RPL3/RPL15/RPL34/RPS3A/EIF4E3/RPL5/EIF3L/RPL10/RPL7/RPL35A/RPL30/C8orf88/PAIP2B |
| GOBP | DENDRITE MORPHOGENESIS | 137 | -0.348577249 | -1.577717605 | 0.001620475 | 0.011618302 | 0.008115571 | 5062 | tags=42%, list=24%, signal=32% | SLITRK5/EPHB3/OPA1/DHX36/LZTS3/CUX2/HDAC6/SEMA3A/DVL1/ARHGAP44/TRPC6/OBSL1/TNIK/PHACTR1/PREX2/SS18L1/UBE3A/PTEN/NEDD4L/NRP1/SHANK2/RELN/ANKRD27/SARM1/HECW2/LRRK2/PDLIM5/ZNF365/NGEF/BHLHB9/NLGN1/MAP6/EFNA1/FARP1/ZDHHC15/DIP2A/SHANK3/TMEM106B/DCLK1/CAPRIN2/TRAK2/GSK3B/PTN/MAP2/RBFOX2/ABI2/KIDINS220/RERE/RAPGEF2/PAK3/EPHB1/FYN/SDC2/DBN1/LRP4/FBXO31/MEF2A/EEF2K |
| GOCC | DNA PACKAGING COMPLEX | 87 | 0.415619001 | 1.614145926 | 0.001617612 | 0.011610168 | 0.00810989 | 7180 | tags=59%, list=34%, signal=39% | H2AX/NCAPH/NCAPG/MACROH2A1/H2AZ1/CENPA/NCAPG2/H2AJ/SMC2/SMC4/H2BC9/MACROH2A2/H3C10/NCAPD2/H2AC4/H2AC13/H2BC10/SLF1/H1-2/H3C7/H2BC12/H3C8/H4C4/H2AC16/H4C9/H2BC13/H1-5/H2BC6/H4C2/GLYR1/H1-3/H4C5/H1-1/KDM1B/IRF4/H2BC7/PRM3/H2BC14/H3C2/H1-9P/H4C1/H1-4/H1-6/H3C11/H2BC17/PRM2/H3C1/H2BW2/H3C3/H3C12/TNP1 |
| GOBP | T CELL APOPTOTIC PROCESS | 48 | 0.489740434 | 1.73066707 | 0.001614123 | 0.01159752 | 0.008101055 | 3351 | tags=46%, list=16%, signal=39% | CD274/WNT5A/PRELID1/PRKCQ/BAK1/HIF1A/IDO1/JAK3/ZC3H8/IL7R/RIPK3/LGALS9/CD27/DOCK8/IL2RA/ADAM8/FADD/CCL5/DNAJA3/FASLG/BAX/BCL10 |
| GOBP | NUCLEOSIDE PHOSPHATE CATABOLIC PROCESS | 79 | 0.424442883 | 1.623357638 | 0.001602929 | 0.011529417 | 0.008053484 | 1988 | tags=28%, list=10%, signal=25% | GDA/TYMP/UPP1/PNP/ACOT7/NUDT15/VCP/ENTPD7/TDG/NT5C2/HPRT1/AMPD3/ITPA/CNP/NUDT5/XDH/DNPH1/GPX1/PDE8A/DCTPP1/SMUG1/NUDT1 |
| GOMF | AMIDE TRANSMEMBRANE TRANSPORTER ACTIVITY | 51 | 0.484740375 | 1.736488236 | 0.001599247 | 0.011515262 | 0.008043597 | 4456 | tags=47%, list=21%, signal=37% | ABCC1/TOMM22/SEC61G/TIMM17A/TAP1/TIMM23/TOMM40L/TAP2/TOMM40/SLC5A6/SLC38A5/SLC15A1/SEC61A1/AQP3/TAPBP/SLC19A1/SLC38A7/SLC15A3/TIMM17B/MCL1/SLC25A32/GJA1/ABCC5/SEC61A2 |
| GOBP | RETINA DEVELOPMENT IN CAMERA TYPE EYE | 143 | -0.341289479 | -1.547703936 | 0.001588533 | 0.011450392 | 0.007998284 | 5282 | tags=38%, list=25%, signal=28% | DLL1/HIPK1/TOPORS/NPHP1/MDM1/FJX1/OPA1/TBC1D32/MED1/LRP5L/OBSL1/TGFB2/SERPINF1/LRP5/ACVR2B/PDE6A/RPL24/NRP1/CYP1B1/RAB11FIP4/NECTIN3/NPHP4/CLIC4/MFSD2A/RORB/PDGFRB/TFAP2A/MERTK/AHI1/GNB1/SLC17A7/PTPRM/DIO3/ATP2B1/SOX8/ARHGEF15/MAX/ACVRL1/TUB/LRP6/NTRK2/LAMB2/PTN/RBP4/RHOJ/ZHX2/SKI/BMPR1B/LAMC3/ATP2B4/TFAP2B/FZD4/PDGFRA/CDON |
| GOBP | PURINE CONTAINING COMPOUND SALVAGE | 15 | 0.686225744 | 1.856129871 | 0.00158771 | 0.011450392 | 0.007998284 | 1633 | tags=33%, list=8%, signal=31% | PNP/APRT/HPRT1/AMPD3/ADK |
| GOBP | PTERIDINE CONTAINING COMPOUND BIOSYNTHETIC PROCESS | 13 | 0.714912094 | 1.852258132 | 0.00158209 | 0.011428471 | 0.007982972 | 1973 | tags=54%, list=9%, signal=49% | GCH1/PTS/GART/ATIC/MTHFD1/MTHFD1L/DHFR |
| GOBP | CARBOHYDRATE CATABOLIC PROCESS | 190 | 0.353660465 | 1.527939364 | 0.00157979 | 0.011424146 | 0.007979951 | 4470 | tags=36%, list=21%, signal=29% | GM2A/ENO1/TPI1/PGAM1/PGK1/PGM2/HK2/TIGAR/GALE/GK/PYGL/PGD/FUT2/HIF1A/EIF6/NUP37/NUP210/LDHA/ALDOA/FOXK2/NUP88/IFNG/FUT1/GAPDH/PPP1CA/NUDT5/GPI/PHKA1/NAGA/AOAH/GALK1/NUP50/RAE1/DHTKD1/RBKS/SEC13/PFKFB2/PFKP/NUP155/GLYCTK/NEU2/PKM/NDC1/PFKFB4/ADPGK/NUP188/PRKAG1/DERA/NUP85/NUP205/G6PC1/OGDHL/ENO3/GSK3A/PHKG2/PPP1R3D/PGK2/NUP58/NUP107/MAN2B1/NUP93/NEU1/POM121/ENOSF1/GK2/HK3/SLC25A12/NUP160/CHIA |
| GOBP | EPITHELIAL CELL DIFFERENTIATION INVOLVED IN KIDNEY DEVELOPMENT | 48 | -0.467660545 | -1.75750896 | 0.001579373 | 0.011424146 | 0.007979951 | 3069 | tags=44%, list=15%, signal=37% | BMP4/JAG1/ASXL1/WWTR1/YAP1/ADIPOQ/LIF/MTSS1/PODXL/GREM1/LAMB2/MEF2C/IQGAP1/PTPRO/CD34/ACTA2/OSR1/KLF15/MAGI2/NOTCH2/GATA3 |
| GOBP | EXTRACELLULAR MATRIX ASSEMBLY | 44 | -0.481041306 | -1.769293288 | 0.001566212 | 0.011350388 | 0.007928429 | 4774 | tags=55%, list=23%, signal=42% | NTNG2/COL1A2/ATP7A/PLOD3/PHLDB2/RAMP2/EFEMP2/AGT/RGCC/LOX/NTN4/MFAP4/CLASP1/ANTXR1/GPM6B/GAS6/TNXB/LAMB2/PHLDB1/FBLN5/DAG1/SMAD3/MYH11/LAMB4 |
| GOBP | POSITIVE REGULATION OF CELL ADHESION | 411 | 0.298992281 | 1.38913213 | 0.001562561 | 0.011336162 | 0.007918492 | 4733 | tags=36%, list=23%, signal=28% | CD24/CD274/NFKBIZ/WNT5A/NOD2/PNP/SLC7A1/C1QBP/IL4R/FUT3/PRKCQ/PYCARD/CCR7/CXCL13/PTAFR/GFUS/CD83/PRSS2/FERMT1/VNN1/LCK/IRAK1/CYRIB/SASH3/RHOH/AP3D1/JAK3/IFNG/FUT1/SPOCK2/IL1B/CCL2/ICOS/P4HB/LYN/CSK/CD6/CORO1A/SOCS1/CBFB/IL12B/EPHA1/HSPH1/SYK/SELE/ARPC2/IL7R/NR4A3/IL6/ITGA4/CD36/LGALS9/CCL19/CD27/RELL2/ITGB2/AP3B1/PTPN22/CIB1/IL23A/BTLA/ADAM19/CD5/DOCK8/PLAUR/RSU1/IL2RA/TFRC/HLA-DMB/FLOT2/ADAM8/FADD/LILRB4/HLA-E/TNFSF9/CD86/HSPD1/SIRPG/PDPN/CCL5/DNAJA3/TNF/LEF1/ADAM9/EPB41L5/EBI3/IL36B/NFKBID/RIN2/XCL1/IL1RL2/MAP3K8/CD3E/ANXA1/HLA-DRA/ICAM1/IL12RB1/STX4/BCL10/CCR2/LILRB2/YES1/SELP/ST3GAL4/ITGA3/IL15/CARD11/TMEM102/ZAP70/LILRB1/RASAL3/UNC13D/GRAP2/NLRP3/TNFSF13B/HLA-G/STX3/VAV1/JUP/CD80/KIFAP3/IL10/FOXP3/THY1/XBP1/EPO/PLEKHA2/EMP2/VCAM1/NCK2/CR1/CCDC88B/CD1D/CLECL1/SERPINF2/PTPRC/CD28/MALT1/DNM2/TNFSF14/AIF1/ITGA6/NKAP/AGER/FOXF1/SOX2/PIEZO1 |
| GOBP | NEGATIVE REGULATION OF INTERLEUKIN 10 PRODUCTION | 19 | 0.655153962 | 1.881758594 | 0.001561669 | 0.011336162 | 0.007918492 | 4624 | tags=63%, list=22%, signal=49% | CD274/TNFRSF21/IDO1/TRIB2/JAK3/IL12B/IL23A/LILRB4/TYROBP/LILRB1/FOXP3/AGER |
| GOBP | GLUCOSE CATABOLIC PROCESS | 35 | 0.535689289 | 1.774028801 | 0.001557818 | 0.011326211 | 0.007911541 | 4360 | tags=49%, list=21%, signal=38% | ENO1/TPI1/PGAM1/PGK1/HK2/TIGAR/ALDOA/FOXK2/GAPDH/GPI/PFKFB2/PFKP/PKM/ADPGK/ENO3/HK3/SLC25A12 |
| GOBP | REGULATION OF NATURAL KILLER CELL MEDIATED IMMUNITY | 43 | 0.516044822 | 1.770235536 | 0.001557395 | 0.011326211 | 0.007911541 | 3933 | tags=47%, list=19%, signal=38% | SERPINB4/RAET1E/IL12B/SERPINB9/RASGRP1/IL18RAP/LGALS9/SH2D1A/LAG3/HLA-E/NCR3/CRTAM/KLRD1/CD96/HLA-F/HLA-B/LILRB1/NECTIN2/HLA-G/VAV1 |
| GOBP | REGULATION OF MITOCHONDRIAL MEMBRANE PERMEABILITY | 69 | 0.44623876 | 1.667095863 | 0.001547392 | 0.011274812 | 0.007875639 | 3775 | tags=38%, list=18%, signal=31% | TFDP1/PPIF/SLC25A5/GZMB/HK2/BAK1/NMT1/DYNLT1/YWHAQ/SFN/BLOC1S2/VDAC2/CNP/BID/CASP8/BNIP3/TMEM14A/BOK/TP63/ALKBH7/YWHAZ/BAX/HIP1R/TMEM102/MAPK8/GSK3A |
| GOBP | ENDOTHELIUM DEVELOPMENT | 125 | -0.35396063 | -1.568226876 | 0.001547298 | 0.011274812 | 0.007875639 | 5512 | tags=49%, list=26%, signal=36% | SOX18/HEG1/RHOA/COL18A1/PDE4D/DLL1/S1PR3/SMAD4/CDH5/FASN/PLOD3/HEY1/TMEM100/BTG1/S1PR1/KDR/CTNNB1/ACVR2B/BMP6/RAP1B/PRKD2/GSTM3/FOXC2/RDX/FSTL1/NRP1/RAP1A/VCL/FGF1/CLIC4/BMP4/WNT7B/CLDN3/HOXB5/JAG1/PECAM1/PLCB1/RAPGEF3/ADD1/MET/ROBO4/CLDN5/ACVRL1/PTPRS/RHOB/TNMD/NR2F2/PTN/ZEB1/STARD13/ROCK2/PDE2A/CD34/RBPJ/ATOH8/RAPGEF2/MYADM/FOXJ2/VEZF1/ARHGEF26/CLDN1 |
| GOCC | PROTON TRANSPORTING TWO SECTOR ATPASE COMPLEX CATALYTIC DOMAIN | 17 | 0.660438252 | 1.842797671 | 0.001546506 | 0.011274812 | 0.007875639 | 3154 | tags=65%, list=15%, signal=55% | ATP6V1B2/ATP5F1B/ATP6V1F/ATP5F1C/ATP6V1C1/ATP6V1C2/ATP5PO/ATP6V1H/ATP6V1A/ATP5F1D/ATP5F1A |
| GOBP | POSITIVE REGULATION OF FOCAL ADHESION ASSEMBLY | 25 | -0.584189469 | -1.886324985 | 0.001545774 | 0.011274812 | 0.007875639 | 4390 | tags=52%, list=21%, signal=41% | TEK/KDR/NRP1/ABL1/S100A10/FMN1/COL16A1/IQGAP1/SMAD3/FERMT2/SDC4/MYOC/TSC1 |
| GOBP | NEGATIVE REGULATION OF ACTIN FILAMENT POLYMERIZATION | 61 | -0.430654368 | -1.683403109 | 0.001536621 | 0.011245119 | 0.007854898 | 2440 | tags=39%, list=12%, signal=35% | TRIOBP/DMTN/RDX/TMSB4X/SLIT2/KANK4/ADD1/TMOD2/TMSB15B/VILL/PFN2/SPTAN1/SCIN/FHOD3/SVIL/EPS8/SPTBN2/MYADM/ADD3/LMOD1/TMOD1/GSN/KANK1/SPTBN1 |
| GOBP | REGULATION OF KERATINOCYTE DIFFERENTIATION | 39 | 0.522348381 | 1.752125302 | 0.001533083 | 0.011231465 | 0.00784536 | 2653 | tags=44%, list=13%, signal=38% | SERPINB13/MACROH2A1/PRKCH/TRIM16/ZBED2/IL20/CTSL/CYP27B1/CBFB/OVOL2/ZFP36/ETV4/GRHL1/TP63/AQP3/MACROH2A2/PLAAT4 |
| GOBP | POSITIVE REGULATION OF FIBROBLAST MIGRATION | 14 | -0.695578084 | -1.92268669 | 0.001531479 | 0.011231465 | 0.00784536 | 3727 | tags=79%, list=18%, signal=65% | BAG4/PAK1/DMTN/PRKCE/DDR2/PTK2/AKAP12/ITGB1/SLC8A1/PAK3/ARHGEF7 |
| GOBP | REGULATION OF INTRINSIC APOPTOTIC SIGNALING PATHWAY | 151 | 0.373239407 | 1.573105843 | 0.001483062 | 0.010888756 | 0.007605972 | 4246 | tags=35%, list=20%, signal=28% | S100A9/ENO1/S100A8/PPIF/PYCARD/HDAC1/HIF1A/NDUFA13/IL19/MMP9/VNN1/PPIA/PTPN2/LCK/DDIAS/NDUFS3/VDAC2/BCAP31/TRAP1/MIF/P4HB/FIGNL1/BID/BCL2L12/GPX1/SOD2/HYOU1/RIPK3/ARMC10/NOC2L/SLC9A3R1/PLAUR/BDKRB2/BOK/PTPMT1/TRIAP1/PARK7/TMEM161A/BAX/ING2/PARP1/HTRA2/MDM2/GSDME/BECN1/KDM1A/MCL1/CDKN2D/STYXL1/MAPK7/XBP1/EPO/NCK2 |
| GOBP | POSITIVE REGULATION OF WNT SIGNALING PATHWAY | 169 | 0.356612593 | 1.524097563 | 0.001477735 | 0.010861513 | 0.007586942 | 2159 | tags=27%, list=10%, signal=25% | PSME2/PSMB2/CDH3/WNT5A/PSMA5/PSMB5/RUVBL1/PSMA3/PSMD12/PSME4/PSMA2/PSME1/VCP/PSMB6/DEPDC1B/PSMB10/PSMB3/PSMD6/PSMB8/PSMC4/PSMA4/PSMD2/TLR2/PSMD11/PSMD1/PSMD14/PSMC3/PSMB9/NFKB1/PSMA7/PSMD9/PSMD8/CSNK2A1/PSMA1/PSMD13/ATP6V1C2/PSMF1/GSKIP/PSMC5/PSMC2/PSMB1/CSNK1G2/PSMC6/PSME3/PSMD7/CSNK1G3 |
| GOBP | ANATOMICAL STRUCTURE HOMEOSTASIS | 445 | 0.292907647 | 1.371546142 | 0.001472763 | 0.010836826 | 0.007569698 | 4287 | tags=28%, list=21%, signal=23% | GJB6/CDH3/NOD2/LTF/PRKCQ/AURKB/CCT5/NEK2/POLE2/HOMER1/PCNA/CCNE1/ABCA12/HIF1A/NHP2/HUS1/RPA3/RAD51/CCNE2/CCT2/PPARGC1B/HNRNPA2B1/MKKS/CCT3/FEN1/GNL3/NOP10/POLE4/TJP2/HNRNPC/CCT7/STN1/ALDOA/SASH3/RDH12/PRDX1/CLCN3/EXO1/SLC2A1/SERPINA3/PINX1/LIPA/CIB2/MYC/BAG3/PNKP/POLE3/CDK5/CSK/CORO1A/POLD2/XRCC6/RFC2/TPRKB/SYK/SNX10/LYZ/DNA2/RAC2/IL6/BLM/RFC3/XRCC5/FH/BRCA2/PIF1/PRKDC/GAR1/POLA2/TFRC/GPR137B/CLDN12/ADAM8/PRIM1/TMEM64/TCP1/P2RY1/STRAP/SMC6/DKC1/ACACA/LPCAT1/SFTPD/FBXO4/CCT6A/NAT10/ZG16B/POLD4/PML/CCT8/RB1/BAX/ERCC1/SIRT6/PARP1/TINF2/SP100/MTF1/PROM1/RFC4/TELO2/POLD3/GNL3L/CD38/PTGES3/B2M/RAB3D/NABP2/RAD50/P2RX4/ACP5/TFIP11/UPF1/RECQL4/GJA1/SPP1/SLC1A1/ATR/LSR/PARP3/MAPKAPK5/WRAP53/RPA1/TNFRSF11B/RAD51C/POLD1 |
| GOBP | NEURAL CREST CELL DIFFERENTIATION | 89 | -0.377528937 | -1.604899094 | 0.001470604 | 0.010832805 | 0.00756689 | 5087 | tags=47%, list=24%, signal=36% | EDNRB/FBXL17/SMAD4/TBX1/ALX1/SEMA4C/SEMA3C/SEMA3A/RADIL/PITX2/TAPT1/EDN3/SEMA6D/FOXC2/NRP1/ERBB4/BMP4/HES1/KITLG/NRTN/JAG1/SOX10/SEMA3E/EDN1/SOX8/SEMA4G/SEMA6A/TWIST1/SEMA3B/KLHL12/LRP6/FOXC1/FN1/BMPR1A/FRZB/MEF2C/FAM172A/ALDH1A2/MAPK1/BMP7/SEMA3G/LAMA5 |
| GOBP | POSITIVE REGULATION OF CD4 POSITIVE ALPHA BETA T CELL ACTIVATION | 36 | 0.534610278 | 1.772940706 | 0.001468948 | 0.010832481 | 0.007566663 | 3986 | tags=56%, list=19%, signal=45% | NFKBIZ/IL4R/PRKCQ/CD83/SASH3/IFNG/SOCS1/IL12B/LGALS9/CCL19/IL23A/CD86/NFKBID/XCL1/ANXA1/HLA-DRA/IL12RB1/NLRP3/CD80/FOXP3 |
| GOBP | REGULATION OF PROTEIN LOCALIZATION TO CHROMOSOME TELOMERIC REGION | 14 | 0.700902708 | 1.851841576 | 0.001460031 | 0.010778557 | 0.007528996 | 4190 | tags=86%, list=20%, signal=69% | CCT5/MACROH2A1/CCT2/CCT3/GNL3/CCT7/TCP1/DKC1/CCT6A/CCT8/GNL3L/WRAP53 |
| GOCC | METHYLOSOME | 12 | 0.740279613 | 1.87654843 | 0.001460031 | 0.010778557 | 0.007528996 | 3056 | tags=67%, list=15%, signal=57% | SNRPG/SNRPF/SNRPD1/PRMT5/SNRPD3/WDR77/SNRPB/PRMT1 |
| GOBP | IRE1 MEDIATED UNFOLDED PROTEIN RESPONSE | 64 | 0.459888732 | 1.70460244 | 0.001446614 | 0.010703029 | 0.007476239 | 4557 | tags=44%, list=22%, signal=34% | ATP6V0D1/BAK1/HSPA5/SERP1/DNAJB11/YIF1A/COPS5/SRPRB/HDGF/SHC1/HYOU1/EDEM1/PREB/DDRGK1/PDIA6/GET3/BAX/WIPI1/SSR1/MYDGF/EXTL3/PPP2R5B/GSK3A/TMEM33/XBP1/ACADVL/TPP1/DNAJB9 |
| GOBP | REGULATION OF GENERATION OF PRECURSOR METABOLITES AND ENERGY | 156 | 0.365350974 | 1.550289739 | 0.001446511 | 0.010703029 | 0.007476239 | 4158 | tags=37%, list=20%, signal=30% | CCNB1/PGAM1/CDK1/PPIF/PRELID1/GHITM/TIGAR/VCP/PHLDA2/HIF1A/EIF6/SHMT2/NUP37/PNPT1/TACO1/NUP210/NUP88/IFNG/TRAP1/CHCHD2/COX7A2P2/PPP1CA/NOS2/SLC25A33/PDE12/UQCC2/NUP50/COX7A2/DNAJC15/RAE1/BNIP3/SEC13/COX17/ANTKMT/PARK7/PASK/RUBCNL/PFKFB2/NUP155/CISD1/IDE/NDC1/PFKFB4/NUP188/PHB2/OPN3/PRKAG1/NUP85/NUP205/GSK3A/PHKG2/PPP1R3D/NUP58/MTOR/NUP107/NUP93/DNAJC30/POM121 |
| GOBP | T HELPER 1 TYPE IMMUNE RESPONSE | 40 | 0.524341879 | 1.76968542 | 0.001437975 | 0.010662604 | 0.007448001 | 4509 | tags=50%, list=22%, signal=39% | IL4R/BCL3/JAK3/RELB/IL1B/IL12B/CCL19/IL23A/HRAS/LEF1/EBI3/SEMA4A/XCL1/ANXA1/IL12RB1/CCR2/MTOR/CD80/NLRP10/IL33 |
| GOCC | CILIARY PLASM | 126 | -0.347933988 | -1.548375264 | 0.001436288 | 0.010661859 | 0.007447481 | 4489 | tags=30%, list=22%, signal=24% | DNAH8/INPP5E/KIF17/DNAH9/CFAP36/CEP162/BBS7/AK8/ARL6/DYNC2LI1/EFHC2/SPATA7/DNAL1/TTC30A/IFT172/SEPTIN2/DRC3/LCA5/CFAP91/DZIP1L/DYNC2H1/DNAI7/DYNC2I1/TRAF3IP1/TULP3/SEPTIN7/CCDC113/CAMSAP3/BBS1/WDR35/WDPCP/ATG14/SPAG16/EFHC1/GLI2/GLI3/CCSAP/MAP4 |
| GOMF | PEPTIDE ANTIGEN BINDING | 21 | 0.635424309 | 1.851900952 | 0.001424544 | 0.010586378 | 0.007394756 | 5325 | tags=76%, list=26%, signal=57% | TAP1/SLC7A5/TAP2/HLA-E/TAPBP/HLA-DRA/HLA-F/HLA-B/HLA-G/CLEC4M/HLA-DQB1/HLA-A/CD209/HLA-DPB1/HLA-C/SLC7A8 |
| GOBP | POSITIVE REGULATION OF MORPHOGENESIS OF AN EPITHELIUM | 32 | -0.519058603 | -1.775314269 | 0.00141134 | 0.010499869 | 0.007334328 | 3467 | tags=41%, list=17%, signal=34% | WNT5B/LGR4/ABL1/BMP4/AGT/NOG/SOX8/EGF/LIF/GREM1/AR/WNT2B/GATA3 |
| GOBP | POSITIVE REGULATION OF CHONDROCYTE DIFFERENTIATION | 16 | -0.679884211 | -1.930520776 | 0.001406341 | 0.010474279 | 0.007316453 | 1785 | tags=50%, list=9%, signal=46% | ACVRL1/HOXA11/BMPR1B/SMAD3/ZBTB16/GLI3/SOX6/SOX5 |
| GOCC | CELL LEADING EDGE | 404 | -0.260645508 | -1.330107626 | 0.001406095 | 0.010474279 | 0.007316453 | 4828 | tags=35%, list=23%, signal=27% | ARHGAP18/CTTNBP2NL/ATP7A/FLOT1/PIP5K1C/INPP5E/CDC42BPB/ARPIN/MAPT/CDK6/TLR4/MYO1C/SHTN1/RAPH1/LAYN/HDAC6/S100A6/BMX/PTPN13/PSD3/PHLDB2/ARHGAP44/RUFY3/APPL1/TRPV1/SH3YL1/INPPL1/APC/GPER1/ILK/CSPG4/ITGAV/PIK3CA/GABRA2/ALS2/ARHGEF2/RDX/C2CD5/STX2/PLXND1/TIAM2/ASAP3/PKN2/PDE9A/PXN/APP/ABL1/PACSIN2/PTPRK/ITGA5/ROBO2/JCAD/ADGRV1/PARVB/KITLG/WASF2/BCAS3/TLN2/TESC/LDB2/LMO4/FAM107A/KSR1/CYTH3/ABLIM1/AMOT/SWAP70/INSR/PTPRM/ATP2B1/MACF1/ANTXR1/SH3RF1/AMPH/RAB22A/RAPGEF3/ACTC1/NEDD9/ARHGEF6/EEF1A1/NHS/WASF3/SPATA13/PARVA/ACTA1/CTNNA1/FER/SYNE2/PLEKHH2/MTSS1/DST/PODXL/ITGA8/ITGB1/PLEKHG5/SORBS2/PKD2/AIF1L/APBB2/DAG1/IQGAP1/GABARAPL1/PTPRO/FERMT2/PDE4A/ACTA2/ABI2/MYLK/SGCE/CDC42BPA/MKLN1/PAFAH1B1/GDPD2/ABLIM3/TSC1/VIM/FGD4/PLEKHA1/EPS8/PALM/ACTG2/MYH10/ITSN1/MYADM/FGD5/DLC1/ARHGAP31/DBN1/DPYSL3/MCC/ARHGEF7/TPM1/PDLIM4/SPRY2/GSN/SRGAP2/KANK1/SPTBN1/WLS/COBL/ARHGEF26 |
| GOBP | CILIUM ORGANIZATION | 397 | -0.263204886 | -1.345840862 | 0.001401033 | 0.010457935 | 0.007305037 | 5653 | tags=37%, list=27%, signal=27% | SFI1/INTU/KIF3C/CDC14B/FLNA/TCHP/EHD3/IFT88/UNC119B/CCDC66/CCP110/RPGR/IFT81/CEP250/CYLD/CEP290/TRIP11/IFT140/OCRL/NEK1/NPHP1/KIF3A/TXNDC15/TMEM216/ARMC2/HAUS4/TTC8/DNAH8/BBS12/RILPL2/CDKL1/HDAC6/DYNC1H1/KIF17/TBC1D32/PTPN23/TOGARAM1/TTLL1/BBIP1/CEP162/BBS7/SPAG1/ARL6/IFT74/DYNC2LI1/IFT27/C2CD3/CCDC57/NME5/TAPT1/ROPN1/DNAL1/TTC30A/IFT172/TEKT3/IFT52/CEP57/FAM149B1/NPHP4/SEPTIN2/TCTN1/CEP120/BBS9/EHD2/LCA5/RAB23/TTC21B/HAUS2/AHI1/DZIP1L/CEP350/DYNC2H1/WWTR1/SEPTIN6/ABLIM1/YAP1/IQCB1/CLASP1/DZIP1/CFAP410/DYNC2I1/CPLANE1/CFAP69/LRRC49/TRAF3IP1/CEP63/PTPDC1/MACIR/RFX3/CFAP44/PARVA/DYNLL2/TMEM80/OFD1/ALMS1/CIBAR1/NINL/RSPH1/SYNE2/PRKAR2B/IFT22/TESK1/ABCC4/SPEF2/BBS10/FNBP1L/PIFO/TUB/CSNK1D/CLUAP1/ARHGAP35/TTC17/RO60/PIBF1/ARL3/SEPTIN7/DYNLRB2/PKD2/GALNT11/CCDC113/NPHP3/BBS1/CROCC/JHY/PCM1/TTBK2/SPATA6/IFT122/WDR35/WDPCP/FAM161B/AKAP9/PAFAH1B1/ABLIM3/WDR19/RILPL1/LAMA5/SPAG16/CEP126/ZNF423/BBS2/GSN/TUBA1A/DYNLRB1/MAP4 |
| GOBP | DNA CATABOLIC PROCESS | 39 | 0.524220034 | 1.758403431 | 0.001384829 | 0.010348475 | 0.007228577 | 4220 | tags=44%, list=20%, signal=35% | TREX2/ISG20/DNASE1L3/IL6/ENDOG/ST20/ERI1/ERI2/KPNB1/HMGB2/BAX/XRN2/CASP3/DNASE1L1/REXO4/EXOG/DNASE1L2 |
| GOBP | ACTIN FILAMENT DEPOLYMERIZATION | 56 | -0.452218534 | -1.749907512 | 0.00137779 | 0.010307345 | 0.007199847 | 3750 | tags=39%, list=18%, signal=32% | TRIOBP/DMTN/PIK3CA/RDX/MICAL1/SWAP70/ADD1/TMOD2/PLEKHH2/DSTN/VILL/SPTAN1/SCIN/SVIL/CFL2/EPS8/SPTBN2/ADD3/LMOD1/TMOD1/GSN/SPTBN1 |
| GOBP | POSITIVE REGULATION OF PROTEIN CONTAINING COMPLEX ASSEMBLY | 247 | 0.328873819 | 1.46231194 | 0.001369881 | 0.0102596 | 0.007166496 | 4294 | tags=33%, list=21%, signal=27% | FCHSD1/CLEC7A/PYCARD/CCR7/CXCL13/ARPC5L/VCP/BAK1/PSMC4/ARPC1A/RPA3/BIK/WARS1/FERMT1/GBP5/STMP1/ARPC3/CDC42EP5/ARPC1B/FSCN1/IFNG/PSMC3/ACTR3/CDT1/BID/CKAP5/CORO1A/MMP1/RBX1/PSRC1/HCK/MTLN/PIH1D1/SYK/ARPC2/CD36/RASIP1/PSMC5/PSMC2/BRK1/PSMC6/PLEK/TFRC/ARFIP2/DHX33/C15orf62/WASL/ARPC5/PARK7/CYFIP1/MAPRE1/WASHC5/TNF/PFN1/DRG1/BAX/ERCC1/PARP1/CAND1/EIF4G1/LCP1/BAIAP2/ICAM1/HIP1R/GMFG/GNL3L/WAS/PPP2R5B/CDC42EP1/MTOR/MSN/LMOD2/ATR/MED25/BAIAP2L2/NAV3/SLF1/TCL1A/VASP/RPA1/NCK2/MMP3 |
| GOBP | POSITIVE REGULATION OF CHEMOKINE PRODUCTION | 58 | 0.472488722 | 1.71338796 | 0.001364373 | 0.010229752 | 0.007145647 | 4807 | tags=47%, list=23%, signal=36% | WNT5A/NOD2/CLEC7A/IL4R/MYD88/PYCARD/HIF1A/ADAM17/TLR2/IFNG/IL1B/HMOX1/DEFB124/SYK/IL6/LGALS9/FFAR2/TSLP/TNF/MCOLN2/IL17F/EIF2AK2/CHIA/IL33/AIF1/AGER/TICAM1 |
| GOMF | CARD DOMAIN BINDING | 16 | 0.690497514 | 1.912055836 | 0.001363245 | 0.010229752 | 0.007145647 | 3704 | tags=62%, list=18%, signal=51% | NOD2/CASP4/CASP1/CARD14/CARD16/CARD9/BCL10/CARD18/CARD11/CARD10 |
| GOBP | NEGATIVE REGULATION OF CYSTEINE TYPE ENDOPEPTIDASE ACTIVITY | 77 | 0.429398007 | 1.633573517 | 0.001361097 | 0.010228016 | 0.007144435 | 3676 | tags=38%, list=18%, signal=31% | LTF/IFI16/DNAJB6/LAMP3/IFI6/MMP9/SFN/CST7/LAMTOR5/SERPINB9/BCL2L12/RPS6KA1/GPX1/CSNK2A1/PIH1D1/CD27/CAAP1/PLAUR/BEX3/CARD16/SIAH2/PRDX5/TRIAP1/PARK7/TNF/TNFAIP8/NLE1/CARD18/CDKN2D |
| GOBP | POSITIVE REGULATION OF CELLULAR PROTEIN LOCALIZATION | 290 | 0.32164933 | 1.449964011 | 0.001360928 | 0.010228016 | 0.007144435 | 4044 | tags=34%, list=19%, signal=28% | ZC3H12A/RAN/EPHA2/PARP9/CDK1/TFDP1/GZMB/NDC80/FZD5/ABHD17C/RER1/CCT5/ABCA12/DTX3L/PRKCH/RAB11A/NMT1/UBE2L3/YWHAQ/CCT2/ZPR1/CCT3/GNL3/UBE2D3/SFN/ECT2/EPHB2/CCT7/MEAK7/IFNG/LAMTOR5/MESD/PINX1/CDT1/UBL5/ATG13/BAG3/BID/CDK5/CD247/MTCL1/PDCD5/PLK1/TCAF2/LIMK2/RAC2/SESN2/ITGB2/CIB1/SAE1/HRAS/TFRC/GPR137B/CASP8/DDRGK1/TP63/RHOG/TARDBP/TCP1/YWHAZ/ARIH2/PARK7/PIK3R2/DKC1/GPSM2/UBE2J2/NIPBL/HCLS1/CCT6A/SREBF2/RANGRF/TNF/TREM2/MFF/CCT8/PARP1/HTRA2/MIEF1/TYROBP/MCRS1/STX4/PRKCD/PLS1/GNL3L/ENTR1/ITGA3/NUTF2/MAPK8/CEMIP/CARD10/HDAC3/GSK3A/CACNB3/STX3/CDH1/MSN/JUP/ABHD17B/MAPK14/ZDHHC5 |
| GOCC | HOST CELLULAR COMPONENT | 59 | 0.466366767 | 1.703496183 | 0.001353238 | 0.010191761 | 0.00711911 | 4816 | tags=51%, list=23%, signal=39% | RAN/KPNA2/DYNLT1/NUP37/NUP210/IFIT1/NUP88/NUP50/VPS37A/RAE1/SEC13/KPNB1/NUP155/VPS37B/NDC1/UBAP1/NUP188/NUP85/KPNA3/NUP205/MVB12A/NUP58/NUP107/NUP93/POM121/RAB29/NUP160/CLEC4M/NUP35/NUP42 |
| GOBP | REGULATION OF GENE SILENCING BY RNA | 112 | 0.390893015 | 1.585991324 | 0.001351432 | 0.010189584 | 0.007117589 | 4816 | tags=41%, list=23%, signal=32% | ZC3H12A/EIF4E2/POLR2H/NUP37/POLR2F/STAT3/NUP210/ZMPSTE24/TARBP2/NUP88/NUP50/IL6/POLR2L/ZFP36/ADAR/POLR2G/ELAVL1/POLR2D/RAE1/XPO5/SEC13/H3C10/NUP155/POLR2E/TNF/MYCN/NDC1/EIF4G1/NUP188/NUP85/POLR2I/NUP205/NUP58/MAP2K1/NUP107/NUP93/POM121/POLR2J/H3C7/NUP160/H3C8/H4C4/H4C9/NUP35/MAP2K2/NUP42 |
| GOBP | CARDIAC SEPTUM MORPHOGENESIS | 63 | -0.428749484 | -1.674810291 | 0.001330988 | 0.010046718 | 0.007017795 | 5056 | tags=43%, list=24%, signal=33% | SMAD4/TBX1/SEMA3C/WNT11/HEY1/TGFB2/SAV1/NRP1/BMP4/ROBO2/HES1/JAG1/TBX3/DHRS3/SOX4/SLIT2/NOG/PARVA/SLIT3/FGFR2/BMPR1A/RBPJ/TGFBR2/BMP7/ZFPM2/NOTCH2/TGFBR3 |
| GOBP | INTRINSIC APOPTOTIC SIGNALING PATHWAY IN RESPONSE TO DNA DAMAGE | 98 | 0.410343758 | 1.629044725 | 0.001322575 | 0.009994444 | 0.006981281 | 3676 | tags=38%, list=18%, signal=31% | EPHA2/IFI16/PYCARD/BCL2A1/POLB/BAK1/DDIAS/BCL3/SFN/AEN/MIF/IKBKE/BID/HMOX1/CDKN1A/BCL2L12/BRCA2/PRKDC/CHEK2/TNFRSF1B/BOK/TP63/TRIAP1/HIPK2/TNFRSF1A/BRCA1/TNF/TMEM161A/PML/BAX/ING2/HTRA2/CIDEB/SHISA5/KDM1A/MCL1/CDKN2D |
| GOBP | REGULATION OF TRANSCRIPTION INVOLVED IN G1 S TRANSITION OF MITOTIC CELL CYCLE | 34 | 0.544880038 | 1.78873844 | 0.001312969 | 0.009933028 | 0.00693838 | 2984 | tags=44%, list=14%, signal=38% | RRM2/TFDP1/PCNA/CCNE1/TYMS/CDC6/CDC45/ZPR1/CDT1/CDK5/ORC1/DHFR/FBXO5/E2F6/RB1 |
| GOBP | CHEMOKINE PRODUCTION | 89 | 0.419016346 | 1.635674378 | 0.001306721 | 0.009896901 | 0.006913146 | 2901 | tags=29%, list=14%, signal=25% | S100A9/WNT5A/NOD2/S100A8/EPHA2/CLEC7A/IL4R/MYD88/PYCARD/GSTP1/HIF1A/ADAM17/TLR2/IFNG/IL1B/HMOX1/DEFB124/SYK/IL6/LGALS9/FFAR2/CXCL6/LILRB4/TSLP/TNF/TREM2 |
| GOBP | REGULATION OF DNA REPLICATION | 105 | 0.403487818 | 1.620066513 | 0.001302894 | 0.009879055 | 0.006900679 | 3537 | tags=34%, list=17%, signal=29% | TIMELESS/PCNA/TNFAIP1/PPP2CA/CDC6/CCNA2/STOML2/S100A11/E2F8/DBF4/ZMPSTE24/STN1/CDT1/GTPBP4/DONSON/WDR18/RFC2/DNA2/BLM/RFC3/BRCA2/ATG7/CDC7/EREG/FBXO5/HRAS/CHEK2/ESCO2/MAP2K4/ORC5/ATAD5/MSH3/METTL4/RFC4/PPP2R1A/RBBP6 |
| GOBP | NECROTIC CELL DEATH | 54 | 0.487651343 | 1.766453803 | 0.001290778 | 0.009798244 | 0.006844231 | 4189 | tags=44%, list=20%, signal=36% | PPIF/PYGL/CASP1/CFLAR/MLKL/PGAM5/BIRC3/RIPK3/TMEM123/CASP8/FADD/BNIP3/BOK/ALKBH7/LY96/TNF/MAP3K5/FASLG/BAX/TSPO/PELI1/GSDME/TRAF2/RBCK1 |
| GOMF | SINGLE STRANDED DNA HELICASE ACTIVITY | 19 | 0.660208057 | 1.896275162 | 0.001281969 | 0.009742386 | 0.006805214 | 4746 | tags=74%, list=23%, signal=57% | MCM6/RAD51/MCM5/MCM2/MCM7/RFC2/DNA2/RFC3/POLQ/PIF1/RFC4/WRNIP1/CHTF18/RFC5 |
| GOBP | MOVEMENT IN HOST ENVIRONMENT | 169 | 0.35866381 | 1.532864089 | 0.001246695 | 0.009485051 | 0.006625462 | 4109 | tags=37%, list=20%, signal=30% | SERPINB3/KRT6A/TRIM14/EPHA2/CDK1/CXCR4/TMPRSS4/LDLR/PLSCR1/DYNLT1/PHB/TRIM22/PPIA/CXCL8/VAPA/CTSL/TRIM15/ACE2/IFITM1/P4HB/TRIM62/TRIM21/FCN1/TRIM10/IFITM3/LGALS9/CCR5/SLC52A2/SMPD1/XPR1/SELPLG/ITCH/NPC1/TFRC/VAMP8/TRIM5/SLAMF1/SIVA1/CD86/IDE/VPS37B/EXOC2/PML/SNX3/CLEC4G/ICAM1/CIITA/TRIM25/PC/KPNA3/IFITM2/TNFRSF4/TRIM11/NECTIN2/SLC20A2/CDH1/CHMP2A/CD80/PTX3/SLC1A5/HYAL3/GPR15 |
| GOCC | RESPIRATORY CHAIN COMPLEX III | 11 | 0.749061534 | 1.858912274 | 0.00124653 | 0.009485051 | 0.006625462 | 3899 | tags=82%, list=19%, signal=67% | UQCRQ/UQCRH/UQCRFS1/UQCR10/CYC1/UQCC3/UQCRC2/UQCRC1/BCS1L |
| GOBP | POSITIVE REGULATION OF T CELL MIGRATION | 29 | 0.58556888 | 1.837533871 | 0.001242468 | 0.009474348 | 0.006617985 | 3616 | tags=59%, list=17%, signal=49% | WNT5A/CCL20/S100A7/PYCARD/CXCL13/CXCL10/ADAM17/ITGA4/DOCK8/OXSR1/ADAM10/ADAM8/FADD/CCL5/XCL1/CCR2/TMEM102 |
| GOBP | NEURAL TUBE PATTERNING | 33 | -0.545292099 | -1.893042789 | 0.001236785 | 0.00944173 | 0.006595201 | 5211 | tags=45%, list=25%, signal=34% | IFT140/FOXA1/TBC1D32/PRKACB/TCTN1/BMP4/HES1/DZIP1L/EN1/TULP3/LRP6/PTCH1/WDR19/GLI2/GLI3 |
| GOBP | DEVELOPMENT OF PRIMARY SEXUAL CHARACTERISTICS | 208 | -0.305001738 | -1.44218083 | 0.001233557 | 0.009427798 | 0.006585469 | 3121 | tags=26%, list=15%, signal=22% | SIRT1/ROBO2/PDGFRB/KITLG/ATRX/CSDE1/TESC/DACH1/LEP/SPATA2/MGST1/GNRH1/SLIT2/LHCGR/SOX8/WDR48/ESR1/FGF9/CTNNA1/KDM5A/PGR/SLIT3/AKR1C3/FOXC1/ZFX/HOXA11/BMPR1B/IRX5/STAR/ZFPM2/OSR1/FZD4/HOXA10/IMMP2L/WDR19/STAT5B/PLEKHA1/ACVR1B/NUPR1/AGO4/PDGFRA/INHBB/FST/RNF38/AR/PATZ1/KIT/BCL2/SOD1/RHOBTB3/WNT2B/CCND1/ANG/GATA3 |
| GOBP | CYCLIC NUCLEOTIDE MEDIATED SIGNALING | 88 | -0.396912619 | -1.692687176 | 0.00122557 | 0.009377425 | 0.006550283 | 5683 | tags=38%, list=27%, signal=27% | MGRN1/APLNR/PDE4D/DGKQ/EDNRB/PEX5L/AKAP6/CAP2/NDUFS4/PCLO/GUCY1B1/RAPGEF4/NPR1/PDE3A/PDE9A/GAL/RGS2/KSR1/NPR2/RAPGEF3/PRKAR2B/APOE/PRKG1/PDE2A/PDE4A/EIF4EBP2/RAPGEF2/CRTC3/LPAR1/AQP1/GNAI1/IRAG1/ADCY2 |
| GOCC | RNA POLYMERASE I COMPLEX | 13 | 0.723223073 | 1.873790957 | 0.001225109 | 0.009377425 | 0.006550283 | 3892 | tags=69%, list=19%, signal=56% | POLR2H/POLR2F/POLR1H/POLR2L/POLR1C/POLR1B/POLR2E/POLR1G/POLR1A |
| GOBP | REGULATION OF T CELL DIFFERENTIATION | 142 | 0.375667347 | 1.569374755 | 0.001200105 | 0.009203542 | 0.006428823 | 4618 | tags=44%, list=22%, signal=34% | ZC3H12A/NFKBIZ/PNP/PRELID1/IL4R/CD83/VNN1/PTPN2/IRF1/PRDM1/CD2/SASH3/RHOH/AP3D1/JAK3/IFNG/SOCS1/CBFB/IL12B/FANCD2/ZC3H8/SYK/IL7R/LGALS9/CCL19/CD27/AP3B1/IL23A/LAG3/IL2RA/ADAM8/LILRB4/TNFSF9/CD86/CRTAM/TMEM131L/LEF1/IL36B/NFKBID/IL1RL2/ANXA1/HLA-DRA/IL12RB1/CCR2/LILRB2/FOXN1/IL15/CARD11/ZAP70/NLRP3/HLA-G/CD80/FOXP3/XBP1/ZNF683/CR1/PTPRC/CD28/MALT1/FANCA/HLA-DOA/NKAP |
| GOBP | REGULATION OF NON CANONICAL WNT SIGNALING PATHWAY | 23 | -0.591144312 | -1.865642543 | 0.001197245 | 0.009192103 | 0.006420833 | 3467 | tags=61%, list=17%, signal=51% | WNT5B/ABL1/DAB2/RSPO3/MLLT3/NKD1/DACT1/DKK1/CSNK1D/ANKRD6/GPC3/NPHP3/DAAM2/ZNRF3 |
| GOBP | FEMALE MEIOTIC NUCLEAR DIVISION | 25 | 0.612254901 | 1.844397429 | 0.001194089 | 0.00917836 | 0.006411233 | 2785 | tags=40%, list=13%, signal=35% | CCNB2/AURKA/TTK/TRIP13/CDC25B/TOP2A/PLK1/EREG/FBXO5/WASHC5 |
| GOBP | SEGMENT SPECIFICATION | 16 | -0.683701729 | -1.941360559 | 0.001191637 | 0.009170009 | 0.0064054 | 5282 | tags=75%, list=25%, signal=56% | DLL1/IRX3/BMI1/MAFB/MEOX1/IRX2/MLLT3/DVL2/MEOX2/OSR1/IRX1/COBL |
| GOBP | REGULATION OF PHAGOCYTOSIS | 87 | 0.420275069 | 1.632228767 | 0.001182745 | 0.009112016 | 0.006364891 | 5233 | tags=48%, list=25%, signal=36% | NOD2/CLEC7A/PYCARD/PLSCR1/RAB27A/TLR2/LMAN2/IFNG/RAB31/IL1B/CCL2/ATG5/CSK/HCK/SYK/LYAR/CD36/IL2RG/ATG3/C2/SIRPG/CYBA/SFTPD/TNF/TREM2/SNX3/IL15/DOCK2/SPHK1/CD300A/PTX3/IL15RA/FGR/CD300LF/ABCA7/PTPRC/DNM2/FCER1G/IL2RB/SYT7/FPR2/PRTN3 |
| GOMF | DNA POLYMERASE BINDING | 17 | 0.668835911 | 1.866229364 | 0.001181693 | 0.009112016 | 0.006364891 | 3748 | tags=65%, list=18%, signal=53% | PCNA/RAD51/FANCI/CDT1/FANCD2/LONP1/NAT10/SMARCA4/PTGES3/NABP2/CDK2AP1 |
| GOBP | MEIOTIC CHROMOSOME SEPARATION | 25 | 0.613305792 | 1.847563202 | 0.001178061 | 0.009096795 | 0.006354259 | 1635 | tags=28%, list=8%, signal=26% | TTK/CENPX/TOP2A/SHOC1/CHFR/ESPL1/CENPS |
| GOBP | LYMPHOCYTE ACTIVATION INVOLVED IN IMMUNE RESPONSE | 184 | 0.34734075 | 1.493791427 | 0.001172948 | 0.009067735 | 0.006333959 | 4902 | tags=38%, list=23%, signal=29% | ZC3H12A/NFKBIZ/IL4R/ENTPD7/RAB27A/BCL3/STAT3/PLCG2/JAK3/IFNG/EXOSC3/EXO1/RELB/CORO1A/IL12B/IL6/LGALS9/CCL19/IL23A/BATF/NSD2/MAD2L2/GPR183/RNF168/PGLYRP2/TFRC/HMCES/HLA-DMB/ITGAL/VAMP7/CD86/LIG4/HSPD1/DOCK10/EOMES/LEF1/SEMA4A/NFKBID/PGLYRP3/ERCC1/RNF8/ANXA1/LCP1/HLA-DRA/ICAM1/IL12RB1/HLA-F/CD244/LILRB1/UNC13D/NLRP3/MTOR/CD80/IL10/FOXP3/XBP1/PARP3/ZNF683/CR1/TNFSF13/PTPRC/CD28/MALT1/FCER1G/CD40/TBX21/SLC15A4/IFNE/IFNA2 |
| GOBP | RHYTHMIC PROCESS | 287 | -0.283989308 | -1.399274651 | 0.001170925 | 0.009062526 | 0.006330321 | 4492 | tags=34%, list=22%, signal=27% | USP9X/MAPK10/NCOA1/CRY1/NR1D2/MTA1/CIPC/MAGEL2/NONO/TGFB2/GAS2/SERPINF1/EGR2/MSTN/GNAQ/NCOA2/RBM4B/BTRC/UBE3A/PTEN/PHLPP1/LGR4/ZNF830/RAI1/EGR3/NCOR1/SIRT1/SIN3A/RORB/NPAS2/ROBO2/KCND2/RBM4/RORC/HS3ST2/CAVIN3/SFPQ/NR1H3/LEP/CIART/HLF/GNRH1/PER3/SLIT2/ADIPOQ/LHCGR/DBP/NLGN1/TIMP4/ARNTL/ADRB1/TWIST1/ESR1/METTL3/JUND/PPARG/PER2/EGFR/KDM5A/PGR/KLF10/CLOCK/NPY5R/CREBBP/SLIT3/DYRK1A/OGT/CSNK1D/PAM/KLF9/GNA11/PRKAA2/NTRK2/TEF/GSK3B/PPARA/PTN/C3orf70/PPARGC1A/PER1/ROCK2/BMPR1B/FBXW7/SP1/STAR/FZD4/KMT2A/STAT5B/PLEKHA1/ZFHX3/NCOR2/BHLHE41/AXL/PDGFRA/FBXL3/HEBP1/CRY2/RORA/ID4 |
| GOBP | ORGANOPHOSPHATE CATABOLIC PROCESS | 146 | 0.379897503 | 1.593565017 | 0.001168857 | 0.009056953 | 0.006326428 | 3538 | tags=31%, list=17%, signal=26% | GDA/TYMP/PLBD1/UPP1/PNP/GDPD3/PGM2/ACOT7/PLA2G4D/LDLR/NUDT15/VCP/ENTPD7/LIPG/SMPD3/TDG/NT5C2/HPRT1/PLCG2/GPCPD1/AMPD3/ITPA/CNP/IMPA2/NUDT5/XDH/DNPH1/INPP1/GPX1/PDE8A/DCTPP1/SMUG1/MTMR2/NUDT1/SMPD1/BPNT1/SMPD2/SYNJ2/PRDX6/PRKCD/DERA/NUDT13/PNPLA8/ABHD16A/SAMHD1 |
| GOBP | PROTEIN LOCALIZATION TO NUCLEAR BODY | 12 | 0.746107584 | 1.891321864 | 0.001166397 | 0.009048329 | 0.006320404 | 2945 | tags=67%, list=14%, signal=57% | CCT5/CCT2/CCT3/CCT7/TCP1/DKC1/CCT6A/CCT8 |
| GOBP | REGULATION OF EPITHELIAL CELL MIGRATION | 227 | -0.303572482 | -1.450630167 | 0.001159544 | 0.009005565 | 0.006290533 | 4621 | tags=41%, list=22%, signal=32% | PDGFB/DAB2IP/CTSH/HDAC6/PTPN23/TEK/SEMA3A/CAPN7/KDR/HDAC9/HDAC5/TGFB2/SERPINF1/PRKD2/ANGPT1/SCARB1/TACSTD2/MAP2K5/PTEN/FOXC2/NRP1/PRKCE/IQSEC1/VEGFC/TAC1/FGF1/SIRT1/ABL1/BMP4/SRPX2/JCAD/SMOC2/AGT/PRKD1/RGCC/BCAS3/TMSB4X/CLASP1/PTPRM/STAT5A/EDN1/MACF1/SLIT2/SPRED1/PRKCA/SVBP/DOCK5/MMRN2/MET/EFNA1/DUSP10/PPARG/EGF/SASH1/RRAS/ACVRL1/ADGRA2/SYNJ2BP/APOE/PTK2/PIK3CB/PFN2/MECP2/RHOB/KLF4/NR2F2/FGFR1/PTN/MEF2C/AKT3/RHOJ/STARD13/ROCK2/DOCK1/DNAJA4/EPPK1/RTN4/ATOH8/MEOX2/ATP2B4/FBXW7/TGFBR2/SP1/CCBE1/DCN/GATA2/MAP3K3/PLPP3/MCC/EPB41L4B/PATZ1/GATA3 |
| GOBP | NEGATIVE REGULATION OF INTERLEUKIN 12 PRODUCTION | 17 | 0.669640203 | 1.868473553 | 0.00114997 | 0.008941552 | 0.006245818 | 3970 | tags=65%, list=19%, signal=52% | NOD2/C1QBP/CCR7/JAK3/NFKB1/TIGIT/SLAMF1/TLR8/IRAK3/LILRB1/IL10 |
| GOBP | POSITIVE REGULATION OF IMMUNOGLOBULIN PRODUCTION | 43 | 0.523258098 | 1.794979894 | 0.001136951 | 0.008850567 | 0.006182264 | 6698 | tags=74%, list=32%, signal=51% | IL4R/SASH3/EXOSC3/GPI/IL6/NSD2/MAD2L2/TFRC/HMCES/HLA-E/XCL1/STX4/TNFRSF4/MZB1/IL10/XBP1/TNFSF13/PTPRC/CD28/IL33/CD40/TBX21/MLH1/KMT5C/CLCF1/TP53BP1/PAXIP1/IL13/IL5/TLR9/TNFSF4/TGFB1 |
| GOBP | DE NOVO PROTEIN FOLDING | 39 | 0.528705147 | 1.773447948 | 0.001133044 | 0.008830385 | 0.006168167 | 5046 | tags=64%, list=24%, signal=49% | HSPA5/CCT2/ERO1A/HSPA14/HSPA9/CHCHD4/HSPE1/HSPA8/HSPH1/TOR1A/DNAJB1/FKBP1B/SDF2L1/DNAJC7/TOR2A/HSPD1/DNAJC2/PTGES3/TOR1B/DNAJB5/DNAJB12/SDF2/ENTPD5/FKBP1A/SELENOF |
| GOBP | REGULATION OF PROTEIN DEPOLYMERIZATION | 82 | -0.397201596 | -1.657736103 | 0.001127034 | 0.008793741 | 0.00614257 | 3828 | tags=39%, list=18%, signal=32% | APC/TRIOBP/DMTN/CAMSAP2/PIK3CA/ARHGEF2/RDX/SWAP70/ATXN7/CLASP1/ADD1/TMOD2/FGF13/PLEKHH2/BMERB1/DSTN/VILL/SPTAN1/CAMSAP3/TTBK2/SCIN/SVIL/CFL2/MAP1A/EPS8/SPTBN2/ADD3/LMOD1/TMOD1/GSN/SPTBN1/MAP1B |
| GOBP | NEGATIVE REGULATION OF CYTOSKELETON ORGANIZATION | 150 | -0.346619242 | -1.579659842 | 0.001121304 | 0.008759206 | 0.006118447 | 3828 | tags=35%, list=18%, signal=29% | APC/TRIOBP/DMTN/CAMSAP2/PIK3CA/PRKN/TACSTD2/ARHGEF2/RDX/WASF2/TMSB4X/SWAP70/ATXN7/CLASP1/CENATAC/SLIT2/KANK4/ADD1/MET/TMOD2/FGF13/PLEKHH2/TMSB15B/SHANK3/BMERB1/VILL/DYRK1A/PFN2/CLIP3/SPTAN1/MAP2/SNCA/CAMSAP3/ARHGAP6/TTBK2/SCIN/FHOD3/SVIL/MYOC/EPS8/SPTBN2/CORO2B/MYADM/PIK3R1/ADD3/LMOD1/DLC1/TMOD1/CGNL1/GSN/KANK1/SPTBN1/MAP1B |
| GOBP | STRIATED MUSCLE CELL DEVELOPMENT | 101 | -0.378981435 | -1.632556128 | 0.001112727 | 0.008702324 | 0.006078714 | 3113 | tags=34%, list=15%, signal=29% | DNER/OBSL1/KRT8/HDAC9/CAPN3/KRT19/BMP4/PDGFRB/LOX/SHOX2/EDN1/ACTC1/TMOD2/ACTA1/MYH3/AKAP13/SKI/FHOD3/CFL2/TNNT1/FLNC/CSRP1/SGCB/NEBL/PDGFRA/LMOD1/DMD/MYH11/TPM1/TMOD1/PGM5/CAV2/BCL2/MEF2A |
| GOBP | MORPHOGENESIS OF A POLARIZED EPITHELIUM | 142 | 0.376504409 | 1.572871637 | 0.001108294 | 0.008677763 | 0.006061558 | 3178 | tags=33%, list=15%, signal=28% | PSME2/PSMB2/WNT5A/PSMA5/PSMB5/FZD5/AP2S1/PSMA3/PSMD12/PSME4/PSMA2/PSME1/GRHL3/PSMB6/PSMB10/PSMB3/PSMD6/PSMB8/PSMC4/PSMA4/PSMD2/PSMD11/PSMD1/PSMD14/PSMC3/PSMB9/PSMA7/PSMD9/PSMD8/PSMA1/PSMD13/RAB10/PSMF1/PSMC5/PSMC2/PSMB1/PSMC6/SLC9A3R1/PSME3/PSMD7/PSMC1/TP63/SCRIB/PFN1/PSMB7/PSMD10/SAPCD2 |
| GOCC | SMN SM PROTEIN COMPLEX | 16 | 0.696671504 | 1.929152226 | 0.001108138 | 0.008677763 | 0.006061558 | 5481 | tags=81%, list=26%, signal=60% | SNRPG/SNRPF/SNRPD1/SNRPD3/GEMIN6/GEMIN7/SNRPB/STRAP/GEMIN2/SNRPD2/GEMIN8/SNRPE/DDX20 |
| GOBP | REGULATION OF CYCLIN DEPENDENT PROTEIN KINASE ACTIVITY | 98 | 0.412242302 | 1.636581852 | 0.001100994 | 0.008640746 | 0.006035701 | 3679 | tags=39%, list=18%, signal=32% | CCNB1/CCNB2/CKS2/CDKN3/SERTAD1/CCNE1/CDC6/CCNA2/CCNE2/SFN/ADAM17/CDC25A/CCNC/CCNF/GTPBP4/CDKN1A/PSRC1/PLK1/BCCIP/CKS1B/BLM/PKMYT1/CCND2/HERC5/CCNO/CCNQ/CDK7/MEN1/CDC37/CDKN2A/CDC25C/CCNH/GADD45A/PSMD10/CASP3/CCND3/CDKN2D/CDK4 |
| GOBP | PROTEIN LOCALIZATION TO NUCLEUS | 265 | 0.327069726 | 1.462584025 | 0.001098001 | 0.008627338 | 0.006026335 | 4034 | tags=34%, list=19%, signal=28% | ZC3H12A/RAN/KPNA2/PARP9/CDK1/CCT5/DTX3L/MMP12/FERMT1/CCT2/ZPR1/GBP2/CCT3/BCL3/HEATR3/ECT2/STAT3/BYSL/CCT7/IPO4/NUP88/IFNG/LAMTOR5/NUP62CL/PINX1/BAG3/CDK5/MFHAS1/CDKN1A/PLK1/SYK/NOLC1/LIMK2/NUP50/INTS13/GLUL/SESN2/CD36/HIKESHI/TOR1A/NXT1/E2F3/TFRC/CSE1L/RANGAP1/RPF2/ELAVL1/SUPT7L/LILRB4/SEC13/TXN/TARDBP/KPNB1/TCP1/PARK7/PIK3R2/DKC1/HCLS1/FBXO4/PAF1/NUP155/CCT6A/PLRG1/PML/CCT8/PARP1/MDM2/SP100/PKIA/NUP188/MCRS1/RRS1/PHB2/PRKCD/NUP85/CNEP1R1/KPNA3/NUTF2/TOR1B/TOR1AIP2/CARD10/HDAC3/NUP58/POLR1A/CDH1/JUP/NUP107/MAPK14/NUP93/ILRUN |
| GOBP | CELLULAR RESPONSE TO CAMP | 51 | -0.471567144 | -1.792279963 | 0.00108514 | 0.008536263 | 0.005962718 | 4567 | tags=45%, list=22%, signal=35% | AKAP6/SLC26A3/CFTR/CPS1/RAP1B/PCK1/DMTN/RAP1A/APP/GPD1/ADIPOQ/RAPGEF3/ZFP36L1/PKD2/SLC8A1/IGFBP5/PDE2A/RAPGEF2/STAR/AKAP9/AQP1/AQP9/EEF2K |
| GOBP | ADHERENS JUNCTION ORGANIZATION | 65 | -0.430694521 | -1.703058387 | 0.001081387 | 0.008516713 | 0.005949062 | 5439 | tags=49%, list=26%, signal=37% | CDH13/HIPK1/CDH6/CDH5/PIP5K1C/NUMB/PAK2/PTPN23/CTNNB1/BMP6/CDHR3/RAMP2/CADM1/RDX/NECTIN3/VCL/CADM2/CDH10/ADD1/CTNNA1/CADM3/DSP/CDH12/CDH19/CAMSAP3/FERMT2/NUMBL/DLG5/DCHS1/JAM3/ZNF703/ANG |
| GOBP | RENAL SYSTEM PROCESS | 113 | -0.360656908 | -1.575550039 | 0.001077019 | 0.008492267 | 0.005931985 | 3338 | tags=30%, list=16%, signal=25% | TAC1/NPR1/KLHL3/PKN1/ATP6V1B1/ADCY6/ADGRF5/BMP4/AGT/MLLT6/EDN1/ADIPOQ/F2R/ADCY9/ADCY4/PRKAR2B/AKR1C3/GAS6/RAB11FIP2/AKAP11/MAGED2/KCNMA1/ADRA1A/PTPRO/CD34/TFAP2B/AQP1/CORO2B/AGTR1/GSN/BCL2/WFS1/ADCY2/BTC |
| GOBP | POSITIVE REGULATION OF KERATINOCYTE DIFFERENTIATION | 17 | 0.670908503 | 1.872012445 | 0.001070663 | 0.008452076 | 0.005903911 | 2653 | tags=59%, list=13%, signal=51% | MACROH2A1/PRKCH/TRIM16/ZBED2/IL20/CYP27B1/OVOL2/ETV4/MACROH2A2/PLAAT4 |
| GOBP | RNA DEPENDENT DNA BIOSYNTHETIC PROCESS | 68 | 0.460580651 | 1.716303517 | 0.001063267 | 0.008403559 | 0.005870022 | 4208 | tags=47%, list=20%, signal=38% | PRKCQ/AURKB/CCT5/NEK2/NHP2/CCT2/PPIA/CCT3/NOP10/HNRNPC/CCT7/STN1/PINX1/PNKP/XRCC5/PIF1/GAR1/TCP1/DKC1/FBXO4/CCT6A/NAT10/CCT8/TINF2/TELO2/GNL3L/PTGES3/RAD50/ATR/MAPKAPK5/WRAP53/RPA1 |
| GOBP | POSITIVE REGULATION OF ANIMAL ORGAN MORPHOGENESIS | 29 | -0.561880311 | -1.875677378 | 0.001056519 | 0.008360065 | 0.005839641 | 3262 | tags=48%, list=16%, signal=41% | BMP2/FGF1/BMP4/ROBO2/EDN1/SOX8/DKK1/HOXA11/WNT2/CD34/SPRY1/AR/WNT2B/GATA3 |
| GOBP | MYD88 DEPENDENT TOLL LIKE RECEPTOR SIGNALING PATHWAY | 35 | 0.544230053 | 1.802313035 | 0.00105558 | 0.008360065 | 0.005839641 | 4232 | tags=46%, list=20%, signal=37% | IRF7/MYD88/IRAK2/IRF1/IRAK1/TLR2/CD36/TNIP1/HSPD1/LY96/TLR8/IRAK3/CD300A/IRAK4/TLR10/CD300LF |
| GOBP | POSITIVE REGULATION OF OSSIFICATION | 48 | -0.47819456 | -1.797096704 | 0.001054125 | 0.008360065 | 0.005839641 | 4360 | tags=65%, list=21%, signal=51% | PTPN11/ACVR2A/NELL1/KL/PKDCC/TMEM119/TOB2/TGFB2/ACVR2B/BMP2/BMP6/ATRAID/TAC1/BMP4/ADGRV1/TFAP2A/ATP2B1/ANO6/P2RX7/GPM6B/BMPR1A/PTN/MEF2C/SLC8A1/BMPR1B/SMAD3/BMP7/OSR1/ZBTB16/OSR2/ADRB2 |
| GOBP | MEIOTIC SPINDLE ORGANIZATION | 11 | 0.754656359 | 1.872796698 | 0.001053522 | 0.008360065 | 0.005839641 | 3156 | tags=73%, list=15%, signal=62% | CCNB2/AURKA/TUBG1/ESPL1/FBXO5/WASHC5/MYH9/TUBG2 |
| GOBP | PROTEIN LOCALIZATION TO KINETOCHORE | 19 | 0.665542983 | 1.91159834 | 0.001048887 | 0.008338963 | 0.0058249 | 3940 | tags=63%, list=19%, signal=51% | CDK1/NDC80/AURKB/TTK/KNL1/ZWILCH/CDT1/RCC2/HASPIN/MIS12/SPDL1/BUB3 |
| GOBP | PTERIDINE CONTAINING COMPOUND METABOLIC PROCESS | 31 | 0.579650479 | 1.846849619 | 0.001040402 | 0.008281303 | 0.005784624 | 4157 | tags=55%, list=20%, signal=44% | GCH1/PTS/GGH/TYMS/GART/SHMT2/ATIC/MTHFD1/MTHFD2/MTRR/MTHFD1L/DHFR/SLC19A1/SLC25A32/MTHFR/PIPOX/SPR |
| GOBP | POSITIVE REGULATION OF DEFENSE RESPONSE TO VIRUS BY HOST | 31 | 0.580045278 | 1.848107505 | 0.001040402 | 0.008281303 | 0.005784624 | 3674 | tags=45%, list=18%, signal=37% | ZC3H12A/STAT1/PARP9/PYCARD/DTX3L/AIM2/CGAS/IL12B/PTPN22/IL23A/DDX58/PQBP1/IL12RB1/LILRB1 |
| GOBP | KINETOCHORE ASSEMBLY | 17 | 0.671596768 | 1.873932887 | 0.001038941 | 0.008281303 | 0.005784624 | 3189 | tags=65%, list=15%, signal=55% | DLGAP5/CENPN/CENPW/CENPF/CENPX/CENPE/CENPK/CENPA/CENPS/CENPH/MIS12 |
| GOBP | POSITIVE REGULATION OF I KAPPAB KINASE NF KAPPAB SIGNALING | 174 | 0.363431889 | 1.556698292 | 0.0010351 | 0.008268489 | 0.005775673 | 3351 | tags=32%, list=16%, signal=27% | S100A12/TRIM14/NOD2/LTF/UBE2N/MYD88/CCR7/CASP1/PLK2/CFLAR/TNFSF10/TRIM22/IRAK1/VAPA/ECT2/TFG/ZDHHC13/TNIP2/LAMTOR5/IKBKE/TRIM62/TRIM21/CASP10/HMOX1/TBK1/IFIT5/PIM2/BIRC3/CD36/LGALS9/CCL19/SLC44A2/SLC20A1/IKBKG/CARD16/TFRC/CASP8/TRIM5/TNFRSF10B/FADD/REL/LTBR/TNFRSF1A/BST2/TNF/CARD9/DDX21/FASLG/NEK6/CHUK/TRADD/PELI1/SECTM1/SHISA5/TRIM25/BCL10 |
| GOCC | CHAPERONIN CONTAINING T COMPLEX | 10 | 0.769788982 | 1.858971786 | 0.001034053 | 0.008268489 | 0.005775673 | 2945 | tags=70%, list=14%, signal=60% | CCT5/CCT2/CCT3/CCT7/TCP1/CCT6A/CCT8 |
| GOCC | VACUOLAR MEMBRANE | 420 | 0.300831842 | 1.397572997 | 0.001025751 | 0.008213347 | 0.005737155 | 4905 | tags=35%, list=24%, signal=28% | HPSE/TMEM165/AP2S1/MREG/LAMP3/LDLR/CKAP4/ATP6V0D1/DTX3L/DNAJC5/ATP10B/ATP6V1B2/ACP3/TMEM79/VNN1/ATP6V1G1/ATP6V0B/PCSK9/FPR1/SPPL2A/ABCB6/VAPA/BORCS5/SLC7A5/ATP6V1F/BLOC1S2/MEAK7/AP3D1/LAMTOR5/LAMTOR2/CLCN3/AP1S3/SNAP29/KPTN/PI4K2A/BORCS8/SLC36A1/HSPA8/GPR143/AP1M2/IFITM3/TMEM30A/SLC49A4/ATP6V1D/TM9SF1/SYNGR1/SLC3A2/ATP6V1C1/LAMP5/ATP6V1C2/AP3B1/NSF/GNAI3/AP5S1/CTNS/MTMR2/CTSD/THBD/SLC44A2/GRN/NPC1/ZNRF2/VAMP8/SURF4/GPR137B/HLA-DMB/ATP6V1H/ATP6V1A/CTSA/NAPA/LAPTM5/HLA-DOB/SEC13/VAMP7/DDOST/VPS33B/CLCN5/SNAPIN/RRAGC/RUBCNL/CMTM6/LPCAT1/BST2/TLR8/TOM1/AP1B1/OSTM1/CLTA/STK11IP/SLC38A9/CD1B/WIPI1/SLC66A1/AP5M1/SLC15A3/KXD1/HLA-DRA/LAMTOR3/RPN2/TMEM199/ARRB1/CPNE1/TEX264/RMC1/M6PR/CD68/VPS16/HLA-F/ABCC10/RAB3D/VLDLR/P2RX4/ATP6V0A2/SPNS1/MARCHF1/TRAF3IP3/GPR137C/MTOR/AP5B1/HPS6/MAN2B1/WDR41/NEU1/ARL8B/MYO6/CLCN7/CD1D/ATP11B/AP2M1/MYO7A/C3AR1/VAC14/RNF152/CLCN4/HLA-DOA/ACP2/UBA1/PSEN2/MAGT1/OCLN/HLA-DMA/MIOS/SLC15A4/ATG9B/HLA-DQB1/NAPG/PGAP6/STARD3NL |
| GOBP | PROTEIN TRANSPORT ALONG MICROTUBULE | 67 | -0.442168444 | -1.7656766 | 0.001017264 | 0.008155106 | 0.005696473 | 5820 | tags=64%, list=28%, signal=46% | TTC21A/TNPO1/TTC30B/LCA5L/KIF3C/IFT88/RPGR/IFT81/TRIP11/IFT140/KIF3A/MAPK8IP3/BBS12/KIF17/IFT74/DYNC2LI1/IFT27/TTC30A/IFT172/IFT52/KIF5C/LCA5/TTC21B/DYNC2H1/SFPQ/DYNC2I1/TRAF3IP1/PURA/DYNLL2/TERF2/IFT22/TUB/CLUAP1/ARL3/DYNLRB2/CAMSAP3/PCM1/IFT122/WDR35/MAP1A/WDR19/DLG2/DYNLRB1 |
| GOBP | REGULATION OF GENE SILENCING | 131 | 0.385144174 | 1.597679909 | 0.001007693 | 0.008088032 | 0.005649621 | 4879 | tags=40%, list=23%, signal=31% | ZC3H12A/EIF4E2/POLR2H/CDC45/NUP37/POLR2F/STAT3/NUP210/ZMPSTE24/TARBP2/NUP88/ATAD2/NUP50/IL6/POLR2L/ZFP36/ADAR/POLR2G/ELAVL1/POLR2D/RAE1/XPO5/SEC13/H3C10/NUP155/POLR2E/TNF/CDK2/MYCN/NDC1/SIRT6/HMGA1/EIF4G1/NUP188/NUP85/POLR2I/NUP205/NUP58/MAP2K1/NUP107/NUP93/H1-2/POM121/POLR2J/H3C7/NUP160/H3C8/H4C4/H4C9/NUP35/MAP2K2/NUP42/H1-5 |
| GOBP | NATURAL KILLER CELL ACTIVATION | 80 | 0.432047122 | 1.656584118 | 0.001007095 | 0.008088032 | 0.005649621 | 4902 | tags=41%, list=23%, signal=32% | SLAMF7/ULBP2/RAB27A/PRDM1/TUSC2/CD2/PRDX1/CORO1A/IL12B/IL21R/RASGRP1/ITGB2/PTPN22/IL23A/PGLYRP2/CASP8/HLA-E/PIK3CD/VAMP7/NCR3/PGLYRP3/TYROBP/HLA-F/IL15/CD244/UNC13D/FGR/ZNF683/PTPRC/RHBDD3/TICAM1/IFNE/IFNA2 |
| GOBP | MYELOID LEUKOCYTE MIGRATION | 202 | 0.35089046 | 1.530543119 | 0.001002945 | 0.008069202 | 0.005636468 | 4673 | tags=37%, list=22%, signal=29% | S100A12/S100A9/CXCR2/NOD2/S100A8/CCL20/C1QBP/MYD88/S100A7/CCR7/CXCL1/CXCL13/CXCL2/PPIA/CXCL10/CXCL8/CCL18/CCL4/CCL22/CXCL9/S100A14/RHOH/MIF/CCL2/LYN/SLAMF8/DEFB124/CD177/CCL8/SYK/RAC2/IL6/CCL7/CCL19/ITGB2/CXCL17/IL23A/CXCL3/CXCL6/CXCL11/ADAM8/PIK3CD/RHOG/SFTPD/CCL5/TREM2/XCL1/CCR1/PPIB/SRP54/ANXA1/ADGRE2/MCOLN2/WDR1/CCR2/NUP85/CCL17/LGMN/P2RX4/JAML/SERPINE1/CD300A/IRAK4/VAV1/CKLF/CSF3R/C5AR2/HRH1/AKIRIN1/C3AR1/CXCR1/CXCL5/AIF1/AGER/FCER1G |
| GOBP | REGULATION OF BIOMINERALIZATION | 89 | -0.385663367 | -1.639479063 | 0.000982171 | 0.007911541 | 0.005526339 | 5450 | tags=49%, list=26%, signal=37% | ACVR2A/NELL1/KL/BCOR/ECM1/PKDCC/WNT6/HEY1/TMEM119/S1PR1/RFLNB/ACVR2B/BMP2/BMP6/ATRAID/BMP4/ADGRV1/TFAP2A/ENPP1/DDR2/ATP2B1/ANO6/FBLN7/TWIST1/OMD/P2RX7/GPM6B/GREM1/MGP/GAS6/BMPR1A/PTN/MEF2C/SLC8A1/ROCK2/BMPR1B/SMAD3/BMP7/ASPN/OSR1/NBR1/ANKH/OSR2/ADRB2 |
| GOBP | T CELL MIGRATION | 62 | 0.477791213 | 1.760691107 | 0.000961732 | 0.007756205 | 0.005417834 | 4734 | tags=53%, list=23%, signal=41% | C10orf99/WNT5A/CCL20/S100A7/PYCARD/CXCL13/CXCL16/CXCL10/ADAM17/CCL2/ITGA4/RIPK3/GPR183/DOCK8/OXSR1/ADAM10/CXCL11/ITGAL/ADAM8/FADD/PIK3CD/CCL5/XCL1/ICAM1/CCR2/TMEM102/ZAP70/MSN/GPR15/MYO1G/CXCR3/AIF1/AIRE |
| GOMF | WNT PROTEIN BINDING | 30 | -0.565724567 | -1.917438448 | 0.00096002 | 0.007751702 | 0.005414689 | 4256 | tags=63%, list=20%, signal=50% | APCDD1L/LRP5/SFRP5/FZD1/RECK/FZD3/ROR1/FZD10/FZD8/FZD7/RYK/LRP6/APCDD1/ROR2/FRZB/PTPRO/FZD4/WIF1/WLS |
| GOCC | NUCLEAR PORE | 75 | 0.445820955 | 1.684034317 | 0.000959447 | 0.007751702 | 0.005414689 | 4992 | tags=48%, list=24%, signal=37% | RAN/MAD2L1/NUP37/NUP210/RANBP1/NUP88/NUP62CL/MX2/NUP50/XPOT/NXT1/CETN2/RANGAP1/RAE1/MVP/SEC13/KPNB1/NUP155/NDC1/GLE1/NUP188/NUP85/KPNA3/NUP205/NUTF2/EIF5A/AGFG1/ENY2/NUP58/NUP107/NUP93/POM121/NUP160/NUP35/NUP42/NXT2 |
| GOBP | REGULATION OF TELOMERE MAINTENANCE | 75 | 0.445924084 | 1.684423875 | 0.000959447 | 0.007751702 | 0.005414689 | 4174 | tags=44%, list=20%, signal=35% | PRKCQ/AURKB/CCT5/NEK2/CCT2/HNRNPA2B1/CCT3/GNL3/HNRNPC/CCT7/STN1/PINX1/MYC/PNKP/XRCC5/PIF1/TCP1/DKC1/FBXO4/CCT6A/NAT10/PML/CCT8/ERCC1/SIRT6/PARP1/TINF2/GNL3L/NABP2/RAD50/UPF1/ATR/MAPKAPK5 |
| GOBP | NEGATIVE REGULATION OF NATURAL KILLER CELL MEDIATED IMMUNITY | 18 | 0.673616808 | 1.910269858 | 0.000958905 | 0.007751702 | 0.005414689 | 5020 | tags=67%, list=24%, signal=51% | SERPINB4/SERPINB9/LGALS9/HLA-E/KLRD1/CD96/HLA-F/HLA-B/LILRB1/HLA-G/ARRB2/HLA-A |
| GOBP | RIBOSOMAL SMALL SUBUNIT BIOGENESIS | 72 | 0.455115482 | 1.709880342 | 0.000956481 | 0.007751702 | 0.005414689 | 3675 | tags=44%, list=18%, signal=37% | MRPS11/MRPS7/RRP36/TSR1/DCAF13/BYSL/SNU13/NOL10/WDR46/XRCC5/EMG1/UTP4/DDX52/NOP14/FAM207A/RPP40/PRKDC/NPM1/UTP6/RRP7A/LSM6/ABT1/RIOK1/NAT10/NOB1/TBL3/WDR3/NOL11/RRS1/RIOK2/HEATR1/UTP3 |
| GOBP | POSITIVE REGULATION OF ESTABLISHMENT OF PROTEIN LOCALIZATION TO TELOMERE | 10 | 0.772793713 | 1.866227944 | 0.000952633 | 0.007738562 | 0.005405511 | 2945 | tags=80%, list=14%, signal=69% | CCT5/CCT2/CCT3/CCT7/TCP1/DKC1/CCT6A/CCT8 |
| GOBP | MITOCHONDRIAL ELECTRON TRANSPORT CYTOCHROME C TO OXYGEN | 20 | 0.652577103 | 1.886080883 | 0.000951404 | 0.007737929 | 0.005405068 | 4183 | tags=60%, list=20%, signal=48% | CYCS/COX6B1/COX7B/COX5A/COX8A/NDUFA4/COX6A1/COX10/COX7C/COX5B/COX4I1/COX6C |
| GOBP | NOTCH SIGNALING PATHWAY | 179 | -0.320247481 | -1.503411222 | 0.000946271 | 0.007705517 | 0.005382428 | 4123 | tags=34%, list=20%, signal=28% | DNER/TGFB2/GAS2/BMP2/NOTCH3/IFT74/ANXA4/FOXC2/MIB1/PLXND1/IFT172/CNTN1/APP/KRT19/ROBO2/HES1/DTX1/EGFL7/JAG1/DTX4/SPEN/YAP1/SNW1/IL6ST/CCN3/TIMP4/JAG2/TSPAN14/METTL3/EGF/EGFR/NEPRO/ENHO/CREBBP/SYNJ2BP/FOXC1/TM2D3/PBX1/NEURL1B/SORBS2/GALNT11/ZMIZ1/HIF1AN/CFD/RBPJ/FBXW7/TGFBR2/BMP7/PLN/GATA2/CBFA2T2/BCL6/NOTCH2NLA/LFNG/APH1B/ZNF423/NOTCH2/KIT/POSTN/EPN2/MAML2 |
| GOBP | NEURAL TUBE DEVELOPMENT | 144 | -0.342805128 | -1.557835546 | 0.00094402 | 0.007696516 | 0.005376141 | 5735 | tags=47%, list=27%, signal=34% | SHROOM3/NUP133/INTU/CECR2/SFRP1/MED12/SFRP2/TSC2/TEAD2/IFT140/FOXA1/OPA1/CTHRC1/SEMA4C/SEMA3C/RPS7/PLOD3/TBC1D32/DVL1/PRKACB/DVL3/KAT2A/TGFB2/C2CD3/MIB1/IFT172/IFT52/SETD2/PRICKLE1/TCTN1/ABL1/BMP4/HES1/ZNF358/LMO4/DZIP1L/SOX4/STK3/NOG/DVL2/FZD3/DACT1/EN1/TWIST1/TULP3/LRP6/ARHGAP35/SEC24B/ZFP36L1/GDF7/PKD2/NPHP3/ALDH1A2/SKI/IFT122/SDC4/SALL2/BMP7/PTCH1/DCHS1/TSC1/PLXNA2/PKD1/WDR19/DLC1/GLI2/GLI3/COBL |
| GOMF | GUANYL NUCLEOTIDE EXCHANGE FACTOR ACTIVITY | 195 | -0.308072812 | -1.446668431 | 0.000938124 | 0.007657739 | 0.005349055 | 4172 | tags=34%, list=20%, signal=28% | RAB3GAP2/FRMD7/RGL1/DENND1A/ARHGEF17/RASGRF2/RAPGEF4/PREX2/RABGEF1/ALS2/SOS1/ARHGEF2/RALGDS/MADD/EEF1D/RAP1A/IQSEC1/ANKRD27/SOS2/DENND3/ARHGEF1/SMCR8/CYTH3/NGEF/DENND5B/DENND5A/ARHGEF15/DOCK5/DOCK4/FBXO8/RAPGEF3/ADRB1/ARHGEF6/FARP1/SPATA13/TRIO/EGF/RAPGEFL1/FNIP1/SH3BP5/ARHGEF5/DENND11/DOCK7/AKAP13/HERC1/SBF2/DOCK1/VAV3/ARHGEF12/DENND2A/RAPGEF2/RAB3GAP1/RALGPS1/ARHGEF40/FGD4/ITSN1/SERGEF/ARFGEF3/FGD5/MCF2L/ARHGEF7/ARHGEF28/DNMBP/ARHGEF10/BCAR3/DENND4C/LAMTOR4 |
| GOBP | GLAND DEVELOPMENT | 402 | -0.268138974 | -1.366531197 | 0.00093095 | 0.007608422 | 0.005314606 | 3956 | tags=29%, list=19%, signal=24% | SERPINF1/LRP5/BMP2/PITX2/EDA/UPF2/PCK1/PIK3CA/SOCS2/DUT/BTRC/UBE3A/PTEN/ORAI1/PLXND1/MAFB/RAP1A/CCKBR/PTPN3/KRT18/HMGCL/FSTL3/TG/FGF1/HOXD9/ERBB4/ABL1/EDAR/FEM1B/BMP4/WNT7B/JARID2/HES1/TFCP2L1/NRG3/TBX3/LMO4/CSNK2A2/BCL11B/ASXL1/SOX10/NTN4/BTBD7/STAT5A/MST1/NOG/FA2H/DBP/SP3/MET/ESR1/PDGFA/RB1CC1/IRF6/SERPINA5/AACS/EGF/EGFR/PLAG1/CDO1/PGR/SLC29A1/DKK3/LRP6/FOXC1/ARHGAP35/PAM/FGFR2/PBX1/BMPR1A/GDF7/PKD2/LIMS2/PTN/FRZB/NPHP3/PHF2/IGFBP5/NTN1/THRA/DAG1/ALDH1A2/WNT2/PSAPL1/SMAD3/RTN4/RBPJ/MAPK1/WDR35/FBXW7/TGFBR2/BMP7/HOXB3/GATA2/PTCH1/PKD1/PRLR/STAT5B/LAMA5/NCOR2/PDGFRA/GLI2/RPL30/ZNF703/AR/NOTCH2/GLI3/BCL2/CAV1/SOD1/SLC46A2/IRS2/TGFBR3/CRIP1/CCND1/COBL/GATA3/CLDN1 |
| GOBP | PROTEIN DENEDDYLATION | 10 | 0.773206718 | 1.867225314 | 0.000920065 | 0.007528624 | 0.005258865 | 3853 | tags=80%, list=18%, signal=65% | COPS3/COPS5/COPS2/TOR1A/GPS1/COPS6/COPS4/COPS7A |
| GOBP | BONE MINERALIZATION | 105 | -0.379589367 | -1.655251243 | 0.000919212 | 0.007528624 | 0.005258865 | 4004 | tags=41%, list=19%, signal=33% | RFLNB/ACVR2B/BMP2/BMP6/ATRAID/LGR4/FGFR3/BMP4/ADGRV1/PTH1R/TFAP2A/ENPP1/LOX/DDR2/ZBTB40/LEP/ATP2B1/ANO6/TWIST1/OMD/KLF10/P2RX7/GPM6B/FGFR2/GREM1/MGP/BMPR1A/ROR2/GPC3/PTN/MEF2C/SLC8A1/BMPR1B/SMAD3/BMP7/ASPN/OSR1/NBR1/ANKH/TUFT1/OSR2/AXIN2/ADRB2 |
| GOBP | METANEPHRIC EPITHELIUM DEVELOPMENT | 25 | -0.593674599 | -1.916952101 | 0.000917601 | 0.007526791 | 0.005257585 | 3438 | tags=52%, list=16%, signal=43% | LGR4/WNT7B/HES1/WWTR1/YAP1/ADIPOQ/SOX8/LIF/POU3F3/LAMB2/PKD2/OSR1/PKD1 |
| GOBP | RIBONUCLEOSIDE METABOLIC PROCESS | 68 | 0.463348474 | 1.72661751 | 0.000908356 | 0.007460068 | 0.005210978 | 2926 | tags=31%, list=14%, signal=27% | RAN/UPP1/PNP/NME1/AHCY/MFN1/ENTPD7/ACP3/APOBEC3B/TJP2/APRT/IMPDH1/NT5C2/HPRT1/XDH/ADK/GNAI3/GMPS/ADA2/AMD1/CASK |
| GOBP | PROTEASOME ASSEMBLY | 13 | 0.732485427 | 1.897788693 | 0.000900582 | 0.007405278 | 0.005172706 | 4553 | tags=69%, list=22%, signal=54% | POMP/PSMD11/PSMG1/PSMD9/PSMD13/ADRM1/PSMD10/PSMG2/PSMD4 |
| GOCC | LAMININ COMPLEX | 12 | -0.736654463 | -1.951833582 | 0.000886379 | 0.007297422 | 0.005097367 | 2654 | tags=58%, list=13%, signal=51% | NTN4/LAMA3/LAMB2/LAMC1/LAMC2/LAMA5/LAMB4 |
| GOBP | SIGNAL TRANSDUCTION IN RESPONSE TO DNA DAMAGE | 129 | 0.397331928 | 1.64366398 | 0.000872831 | 0.007194697 | 0.005025612 | 5027 | tags=45%, list=24%, signal=34% | CCNB1/CDK1/TFDP1/FOXM1/PCNA/AURKA/SMYD2/PLK2/BCL3/SFN/E2F8/ZMPSTE24/DTL/EEF1E1/MIF/CNOT11/BID/CDKN1A/PLK1/PLK3/SESN2/BRCA2/BATF/MAD2L2/CNOT1/PRKDC/CHEK2/BRCC3/BABAM2/TRIAP1/HIPK2/BRCA1/CDK2/CRADD/CDC25C/PML/BAX/GADD45A/PRMT1/MDM2/SP100/PSMD10/CNOT6/KDM1A/MAPK14/ATR/MYO6/RINT1/CASP9/PMAIP1/CNOT10/YJU2/USP10/GRB2/PYHIN1/PRPF19/BABAM1/PIDD1 |
| GOBP | NUCLEOSIDE MONOPHOSPHATE CATABOLIC PROCESS | 13 | 0.734555554 | 1.903152162 | 0.00086813 | 0.007164737 | 0.005004685 | 1371 | tags=46%, list=7%, signal=43% | TYMP/UPP1/HPRT1/AMPD3/XDH/DNPH1 |
| GOCC | ACTIN FILAMENT BUNDLE | 70 | -0.434146423 | -1.754627658 | 0.000867327 | 0.007164737 | 0.005004685 | 4751 | tags=47%, list=23%, signal=37% | PDLIM2/ACTN4/SYNPO/TEK/SHROOM4/DAAM1/RFLNB/ILK/PXN/FAM107A/PDLIM5/ABLIM1/MYL9/ROR1/ACTA1/PTK2/SEPTIN7/SEPTIN11/PDLIM3/FERMT2/MYH14/MYLK/ABLIM3/LIMCH1/LPP/MYH10/NEBL/SYNPO2/TPM1/PDLIM4/PGM5/SORBS1/CRYAB |
| GOMF | FRIZZLED BINDING | 35 | -0.538329258 | -1.8799715 | 0.000856029 | 0.007082267 | 0.004947078 | 4919 | tags=60%, list=24%, signal=46% | BAMBI/CTHRC1/WNT6/WNT11/DVL1/DVL3/WNT5B/WNT7B/FZD1/RSPO3/RNF43/DVL2/FZD7/RYK/LRP6/ROR2/SDCBP/WNT2/MYOC/ZNRF3/WNT2B |
| GOBP | REGULATION OF CELL MORPHOGENESIS | 298 | -0.288895571 | -1.434011971 | 0.000843675 | 0.006988666 | 0.004881696 | 3850 | tags=30%, list=18%, signal=25% | GRIP1/FAM171A1/HEXB/ILK/TRIOBP/DMTN/SS18L1/PRKN/TACSTD2/RDX/NEDD4L/NRP1/PLXND1/RELN/ZMYM4/SYT1/MFSD2A/DLG1/ANKRD27/ABL1/PARVB/CLDN3/BVES/EFNA5/SEMA3E/S100A13/S100A10/CFAP410/MACF1/FITM2/DVL2/BHLHB9/DOCK5/RHOBTB1/RHOQ/NEDD9/WASF3/PARVA/PALM2AKAP2/TESK1/PTK2/ARHGAP35/ITGA7/PHIP/RHOB/FN1/CAPRIN2/SEPTIN7/SH3D19/LIMS2/EPB41/PTN/SH3KBP1/SYT17/RHOJ/DAG1/CFDP1/FBLN1/DOCK1/FERMT2/SYNE3/MYH14/WDPCP/MYOC/PAK3/FZD4/MKLN1/PLXNA2/PALMD/LPAR1/FYN/FGD4/EPS8/PALM/MYH10/AGO4/MYADM/FGD5/DLC1/DBN1/ARHGEF7/TPM1/FBXO31/KIT/DNMBP/POSTN/MYO9A/KANK1/RHOBTB3/EEF2K |
| GOBP | REGULATION OF ENDOTHELIAL CELL MIGRATION | 166 | -0.333631696 | -1.551030376 | 0.000839875 | 0.006965777 | 0.004865708 | 4056 | tags=40%, list=19%, signal=32% | HDAC9/HDAC5/SERPINF1/PRKD2/ANGPT1/SCARB1/MAP2K5/FOXC2/NRP1/VEGFC/FGF1/SIRT1/ABL1/BMP4/SRPX2/JCAD/SMOC2/AGT/PRKD1/RGCC/BCAS3/TMSB4X/PTPRM/STAT5A/EDN1/SLIT2/SPRED1/PRKCA/SVBP/MMRN2/MET/EFNA1/PPARG/EGF/SASH1/RRAS/ACVRL1/ADGRA2/SYNJ2BP/APOE/PTK2/PIK3CB/MECP2/RHOB/KLF4/NR2F2/FGFR1/PTN/MEF2C/AKT3/RHOJ/STARD13/ROCK2/DNAJA4/ATOH8/MEOX2/ATP2B4/FBXW7/SP1/CCBE1/DCN/GATA2/MAP3K3/PLPP3/PATZ1/GATA3 |
| GOBP | REGULATION OF CELLULAR RESPONSE TO HEAT | 77 | 0.437488697 | 1.664353207 | 0.000839231 | 0.006965777 | 0.004865708 | 4816 | tags=51%, list=23%, signal=39% | DNAJB6/RPA3/NUP37/NUP210/HSBP1/NUP88/IER5/BAG3/HSPA8/HSPH1/NUP50/HIKESHI/DNAJB1/RAE1/SEC13/DNAJC7/CHORDC1/NUP155/NDC1/MLST8/DNAJC2/NUP188/NUP85/PTGES3/FKBP4/MAPKAPK2/NUP205/NUP58/MTOR/NUP107/NUP93/ATR/POM121/RPA1/NUP160/AKT1S1/NUP35/MAPK3/NUP42 |
| GOBP | REGULATION OF LYMPHOCYTE DIFFERENTIATION | 171 | 0.363483514 | 1.553594725 | 0.000828207 | 0.006886008 | 0.004809988 | 4929 | tags=44%, list=24%, signal=34% | ZC3H12A/NFKBIZ/PNP/PRELID1/IL4R/HMGB3/CD83/VNN1/PTPN2/IRF1/PRDM1/CD2/SASH3/RHOH/AP3D1/JAK3/IFNG/SOCS1/CBFB/SLAMF8/IL12B/XRCC6/FANCD2/ZC3H8/SYK/IKZF3/IL7R/LGALS9/CCL19/CD27/AP3B1/IL23A/LAG3/PRKDC/IL2RA/PGLYRP2/ADAM8/LILRB4/TNFSF9/CD86/CRTAM/TMEM131L/LEF1/IL36B/NFKBID/IL1RL2/PGLYRP3/ANXA1/HLA-DRA/IL12RB1/CCR2/LILRB2/FOXN1/IL15/CARD11/ZAP70/NLRP3/HLA-G/CD80/IL10/FOXP3/XBP1/ZNF683/CR1/PTPRC/CD28/MALT1/FANCA/HLA-DOA/NKAP/INHA/PPP2R3C/TBX21/IFNA2/CAMK4/MIR17HG |
| GOBP | NUCLEOTIDE EXCISION REPAIR | 105 | 0.410946896 | 1.650015875 | 0.000825399 | 0.006871167 | 0.004799622 | 3451 | tags=35%, list=17%, signal=30% | COPS3/PCNA/POLR2H/HUS1/RPA3/POLR2F/BRIP1/PNKP/COPS5/POLD2/RBX1/RFC2/COPS2/POLR2L/RFC3/COMMD1/BRCA2/AQR/CETN2/POLR2G/HMGN1/POLR2D/LIG4/CDK7/ISY1/POLR2E/POLD4/ERCC8/GPS1/CCNH/COPS6/ERCC1/PARP1/RFC4/COPS4/POLD3/POLR2I |
| GOBP | POSITIVE REGULATION OF DNA BINDING TRANSCRIPTION FACTOR ACTIVITY | 259 | 0.336337987 | 1.502020347 | 0.000824162 | 0.006869378 | 0.004798372 | 3970 | tags=31%, list=19%, signal=25% | S100A12/S100A9/TRIM14/WNT5A/NOD2/LTF/S100A8/TFDP1/UBE2N/MYD88/PRKCQ/PYCARD/IRAK2/PRKCH/LRP8/PPARGC1B/CFLAR/TRIM22/PPIA/CARD14/IRAK1/TLR2/STAT3/TRIM15/BUD31/AIM2/FOSL1/ADGRF1/LAMTOR5/RIPK4/NFKB1/IL1B/TRIM62/TRIM21/COPS5/IL18RAP/RPS6KA4/TRAF1/IL6/RIPK3/CD36/LGALS9/ITGB2/CIB1/IKBKG/CARD16/TFRC/NPM1/TRIM5/DDRGK1/DHX33/FLOT2/ADAM8/HIPK2/PARK7/HCLS1/LPAR5/TNF/SMARCA4/DDX58/PPRC1/CHUK/TRIM41/TRADD/ICAM1/IRAK3/PHB2/TRIM25/BCL10/TCF3/NLRC4/KDM1A/CARD11/PPP2R5B/ZIC2/NLRP3/SPHK1/NFKB2/JUP/IL10 |
| GOMF | CYSTEINE TYPE ENDOPEPTIDASE ACTIVITY INVOLVED IN APOPTOTIC SIGNALING PATHWAY | 10 | 0.776424965 | 1.874997092 | 0.000822362 | 0.006862893 | 0.004793842 | 3226 | tags=70%, list=15%, signal=59% | CASP4/CASP1/CFLAR/CASP5/CASP10/CASP8/CASP3 |
| GOCC | RNA POLYMERASE II CORE COMPLEX | 14 | 0.716604725 | 1.893327575 | 0.000807593 | 0.006748018 | 0.0047136 | 4846 | tags=64%, list=23%, signal=49% | POLR2H/POLR2F/POLR2L/POLR2G/POLR2D/POLR2E/POLR2I/POLR2J/POLR2J2 |
| GOBP | NCRNA EXPORT FROM NUCLEUS | 37 | 0.549163471 | 1.827902066 | 0.000807207 | 0.006748018 | 0.0047136 | 4816 | tags=59%, list=23%, signal=46% | RAN/NUP37/NUP210/NUP88/NUP50/XPOT/NOL6/NPM1/RAE1/SEC13/NUP155/NDC1/NUP188/NUP85/NUP205/NUP58/NUP107/NUP93/POM121/NUP160/NUP35/NUP42 |
| GOBP | RNA SPLICING | 428 | 0.300156733 | 1.397369752 | 0.000803505 | 0.006730604 | 0.004701435 | 4998 | tags=36%, list=24%, signal=28% | EIF4A3/DDX39A/C1QBP/ESRP2/PPIL1/SNRPG/POLR2H/PPP2CA/SNRPF/SNRPD1/HNRNPA2B1/ZPR1/ZBTB8OS/NCBP1/SRSF9/POLR2F/PRMT5/HNRNPC/DDX41/LSM2/BUD31/PPP4R2/TXNL4A/SF3B3/LSM7/SNRPD3/EFTUD2/WDR77/SNRNP25/SF3B5/LSM1/CD2BP2/SNU13/UBL5/CPSF3/PPIH/LSM10/PSPC1/ALYREF/SNRPA1/GEMIN6/MAGOHB/CSTF2/SNRPC/RTCB/GEMIN7/HSPA8/SF3B6/ZNF326/LSM5/LSM4/POLR2L/SNRPB/SRSF2/AQR/SRPK1/POLR2G/DAZAP1/LUC7L2/ELAVL1/POLR2D/PRPF31/JMJD6/PRPF38A/SRSF7/SRSF1/GRSF1/TSEN15/TSEN54/USP39/USB1/TARDBP/RRAGC/STRAP/HNRNPF/THOC5/LSM6/RBM28/IWS1/HNRNPM/ISY1/CLP1/PRPF4/TSEN34/POLR2E/THOC6/PLRG1/TRPT1/ECD/THOC7/TTF2/PTBP1/YBX1/PRPF4B/PRDX6/PQBP1/GEMIN2/SNRPA/METTL4/CPSF4/PNN/MBNL3/CSTF1/MAGOH/RBM12/SREK1IP1/WDR33/PPP2R1A/POLR2I/PRCC/SNRNP40/KDM1A/ESRP1/CSTF3/TFIP11/TRA2B/DCPS/PRPF38B/SRSF3/CWF19L1/SMNDC1/TCERG1/POLR2J/TXNL4B/PRPF18/TSEN2/CWC25/RPS26/DHX15/RBM7/HNRNPK/SRSF8/NCL/PAPOLA/PRPF40A/ZBTB7A/LSM3/UPF3B/SNRPD2/DDX46/RTRAF/YJU2/WDR83/PUF60/SRSF10/IVNS1ABP/FUS/PRPF19/GEMIN8/CCAR1/SYNCRIP/SAP18 |
| GOBP | NEGATIVE REGULATION OF EPITHELIAL CELL MIGRATION | 70 | -0.435805922 | -1.761334619 | 0.000803082 | 0.006730604 | 0.004701435 | 4590 | tags=46%, list=22%, signal=36% | DAB2IP/PTPN23/SEMA3A/HDAC5/SERPINF1/TACSTD2/MAP2K5/PTEN/RGCC/PTPRM/SLIT2/SPRED1/SVBP/MMRN2/DUSP10/PPARG/ACVRL1/SYNJ2BP/APOE/PFN2/MECP2/KLF4/NR2F2/MEF2C/STARD13/DNAJA4/EPPK1/MEOX2/ATP2B4/DCN/MCC/PATZ1 |
| GOCC | ENDOLYSOSOME | 24 | 0.631461502 | 1.902743344 | 0.000801021 | 0.00672657 | 0.004698618 | 4824 | tags=54%, list=23%, signal=42% | AP2S1/LDLR/CTSB/PCSK9/CTSL/SMPD1/CTSS/TLR8/CLTA/PRKCD/LGMN/AP2M1/SLC15A4 |
| GOBP | EPITHELIAL TO MESENCHYMAL TRANSITION | 144 | -0.346122676 | -1.572911733 | 0.000792981 | 0.00666739 | 0.00465728 | 5115 | tags=44%, list=25%, signal=34% | GLIPR2/SMAD4/FOXA1/ALX1/BAMBI/LOXL3/WNT11/DAB2IP/HEY1/MSX1/TMEM100/PHLDB2/CTNNB1/TGFB2/RFLNB/BMP2/LDLRAD4/USF3/PTEN/GCNT2/SPRED2/BMP4/DAB2/RGCC/DACT3/JAG1/WWTR1/CLASP1/NOG/SPRED1/EFNA1/TWIST1/KLHL12/LRP6/FOXC1/S100A4/FGFR2/GREM1/DDX17/GSK3B/FGFR1/PHLDB1/ELL3/SDCBP/DAG1/WNT2/SMAD3/FERMT2/TGFB1I1/RTN4/RBPJ/TGFBR2/BMP7/DLG5/TASOR/PDCD4/IL17RD/SPRY1/TCF7L2/ZNF703/SPRY2/AXIN2/TGFBR3/GATA3 |
| GOBP | LEUKOCYTE APOPTOTIC PROCESS | 100 | 0.426569953 | 1.69792697 | 0.000790352 | 0.006653614 | 0.004647657 | 3351 | tags=41%, list=16%, signal=35% | CD274/IRF7/WNT5A/NOD2/SLC7A11/PRELID1/TNFRSF21/PRKCQ/AURKB/CCR7/BAK1/HIF1A/IDO1/ADAM17/CTSL/JAK3/LYN/ZC3H8/IL7R/IL6/RIPK3/LGALS9/CCL19/CD27/DOCK8/NOC2L/CD3G/IL2RA/ADAM8/FADD/PIK3CD/TCP1/HCLS1/CCL5/DNAJA3/CDKN2A/FASLG/BAX/ANXA1/CASP3/BCL10 |
| GOBP | SEGMENTATION | 91 | -0.396876543 | -1.691273409 | 0.000771239 | 0.006500854 | 0.004540952 | 5538 | tags=48%, list=27%, signal=36% | SFRP1/POFUT1/MED12/SFRP2/DLL1/EP300/PCDH8/SMAD4/IRX3/SEMA3C/SEMA3A/ATM/KAT2A/BMI1/EGR2/XRCC2/FOXC2/MIB1/HOXD8/MAFB/MEOX1/BMP4/IRX2/TBX3/MLLT3/NKD1/DVL2/DMRT2/LRP6/FOXC1/BMPR1A/ROR2/ALDH1A2/SMAD3/RBPJ/MEOX2/PLD6/OSR1/LFNG/PLXNA2/IRX1/TASOR/AXIN2/COBL |
| GOBP | REGULATION OF ACTIN FILAMENT ORGANIZATION | 260 | -0.294157414 | -1.437960909 | 0.000767499 | 0.006477458 | 0.004524609 | 3843 | tags=31%, list=18%, signal=26% | ARHGEF10L/ELN/TRIOBP/DMTN/PIK3CA/PRKN/TACSTD2/RDX/NRP1/PRKCE/TAC1/ASAP3/PXN/DLG1/WASHC1/ABL1/CCN2/TENM1/WASF2/RGCC/TMSB4X/CXCL12/SWAP70/CLASP1/S100A10/SLIT2/ARHGEF15/KANK4/RAPGEF3/ADD1/MET/FMN1/TMOD2/WASF3/ALMS1/FER/PLEKHH2/TMSB15B/SHANK3/TESK1/DSTN/BIN1/VILL/SORBS3/ARHGAP35/PFN2/SPTAN1/ARHGEF5/RICTOR/ROCK2/ARHGAP6/SCIN/SMAD3/FERMT2/FHOD3/SVIL/ABI2/SDC4/CFL2/MYOC/PAK3/TSC1/LPAR1/EPS8/SPTBN2/LIMCH1/CORO2B/MYADM/PIK3R1/ADD3/LMOD1/DLC1/DBN1/SYNPO2/TPM1/TMOD1/CGNL1/GSN/ARHGEF10/KANK1/SPTBN1 |
| GOBP | PROTEIN K63 LINKED UBIQUITINATION | 55 | 0.489911118 | 1.776843049 | 0.000758104 | 0.006406218 | 0.004474847 | 4605 | tags=53%, list=22%, signal=41% | NOD2/UBE2N/UBE2T/TRIP12/OTUB2/UBE2S/TRIM21/TRAF1/TRAF3/PTPN22/RNF168/ITCH/PLAA/TRIM5/UBE2G1/UBE2V2/ARIH2/HECTD1/RNF126/RNF8/PELI1/OTUB1/PARP10/TRAF4/UBE2E2/TRAF2/NEDD4/RNF152/TRAF3IP2 |
| GOMF | ENDORIBONUCLEASE ACTIVITY | 58 | 0.484873428 | 1.758298674 | 0.000757993 | 0.006406218 | 0.004474847 | 4614 | tags=47%, list=22%, signal=36% | ZC3H12A/RNASEH2A/FEN1/EXO1/CPSF3/RIDA/ELAC2/ENDOU/ZC3H12C/ZC3H12D/ENDOG/RPP40/POP5/SND1/POP4/TSEN34/POP1/NOB1/KHNYN/MRPL44/SLFN13/POP7/RPP30/RPP38/EXOG/TSEN2/ENDOV |
| GOBP | FOLIC ACID CONTAINING COMPOUND METABOLIC PROCESS | 25 | 0.625246606 | 1.883534505 | 0.000753601 | 0.006384227 | 0.004459486 | 4071 | tags=60%, list=20%, signal=48% | GCH1/GGH/TYMS/GART/SHMT2/ATIC/MTHFD1/MTHFD2/MTRR/MTHFD1L/DHFR/SLC19A1/SLC25A32/MTHFR/PIPOX |
| GOBP | OVULATION CYCLE | 61 | -0.442905925 | -1.731293742 | 0.000751686 | 0.006376044 | 0.00445377 | 4040 | tags=43%, list=19%, signal=34% | TGFB2/GAS2/SERPINF1/MSTN/ZNF830/SIRT1/ROBO2/LEP/GNRH1/SLIT2/LHCGR/TIMP4/ESR1/EGFR/PGR/NPY5R/SLIT3/PAM/PTN/BMPR1B/FZD4/STAT5B/PLEKHA1/NCOR2/AXL/PDGFRA |
| GOBP | RENAL SYSTEM VASCULATURE DEVELOPMENT | 25 | -0.598400621 | -1.932212242 | 0.000747873 | 0.006351723 | 0.004436781 | 4621 | tags=64%, list=22%, signal=50% | PDGFB/TEK/ANGPT1/FOXC2/BMP4/HES1/PDGFRB/PECAM1/PKD2/PDGFD/CD34/ACTA2/BMP7/OSR1/PDGFRA/NOTCH2 |
| GOBP | AORTA DEVELOPMENT | 51 | -0.478359532 | -1.818095715 | 0.000747257 | 0.006351723 | 0.004436781 | 5058 | tags=47%, list=24%, signal=36% | LOXL1/TBX1/LRP1/HEY1/NPRL3/TGFB2/EFEMP2/PLXND1/PRICKLE1/ROBO2/HES1/PDGFRB/LOX/JAG1/SOX4/LEP/ACVRL1/SEC24B/BMPR1A/PKD2/PDE2A/RBPJ/TFAP2B/MYLK |
| GOBP | PYRUVATE METABOLIC PROCESS | 147 | 0.376634946 | 1.584467982 | 0.000746692 | 0.006351723 | 0.004436781 | 4423 | tags=39%, list=21%, signal=31% | ENO1/TPI1/PGAM1/PGK1/HK2/TIGAR/HIF1A/EIF6/SLC16A1/NUP37/NUP210/LDHA/ALDOA/FOXK2/NUP88/IFNG/GAPDH/GPI/GALK1/NUP50/NR4A3/PCK2/GSTZ1/VDAC1/RAE1/DHTKD1/SEC13/ME1/PFKFB2/PFKP/NUP155/PDHB/SLC16A3/PKM/NDC1/PFKFB4/ADPGK/DLAT/NUP188/PRKAG1/NUP85/DLD/PC/ME2/NUP205/OGDHL/ENO3/PGK2/NUP58/NUP107/NUP93/PDHA1/POM121/LDHB/HK3/PDP2/NUP160 |
| GOCC | CHAPERONE COMPLEX | 27 | 0.608316654 | 1.890246508 | 0.00074481 | 0.006349788 | 0.00443543 | 4265 | tags=67%, list=20%, signal=53% | CCT5/CCT2/CCT3/DNAJB11/CCT7/PSMG1/BAG3/HSPA8/STIP1/SDF2L1/TCP1/CCT6A/CDC37/CCT8/PTGES3/PSMG2/SDF2/PPP5C |
| GOBP | TELOMERE MAINTENANCE VIA SEMI CONSERVATIVE REPLICATION | 26 | 0.621822922 | 1.905263766 | 0.000740852 | 0.006324075 | 0.004417468 | 4287 | tags=69%, list=21%, signal=55% | POLE2/PCNA/RPA3/FEN1/POLE4/POLE3/POLD2/RFC2/DNA2/RFC3/POLA2/PRIM1/POLD4/RFC4/POLD3/UPF1/RPA1/POLD1 |
| GOBP | CELL REDOX HOMEOSTASIS | 43 | 0.534511465 | 1.833583344 | 0.000724216 | 0.006189924 | 0.004323762 | 5548 | tags=58%, list=27%, signal=43% | ERP44/SELENOT/ERO1A/TXNRD1/PRDX1/NOS2/GPX1/RAC2/GLRX2/NCF2/TXN/PRDX5/TXN2/TXNRD3/CYBA/PRDX6/GCLC/DLD/PRDX4/LPO/SELENOS/APEX1/CYBB/NQO1/NCF4 |
| GOBP | POSITIVE REGULATION OF LYMPHOCYTE MIGRATION | 35 | 0.557608831 | 1.846619199 | 0.000716174 | 0.006128991 | 0.004281199 | 3616 | tags=54%, list=17%, signal=45% | WNT5A/CCL20/S100A7/PYCARD/CXCL13/CXCL10/CCL4/ADAM17/ITGA4/CCL7/DOCK8/OXSR1/ADAM10/ADAM8/FADD/CCL5/XCL1/CCR2/TMEM102 |
| GOBP | POSITIVE REGULATION OF MITOCHONDRIAL MEMBRANE POTENTIAL | 11 | 0.765350551 | 1.899335992 | 0.000700228 | 0.006000165 | 0.004191212 | 1945 | tags=55%, list=9%, signal=49% | MFN1/VCP/STOML2/BID/MTLN/CTNS |
| GOBP | CARDIAC SEPTUM DEVELOPMENT | 95 | -0.393160507 | -1.681416067 | 0.000697088 | 0.005980894 | 0.004177751 | 5212 | tags=47%, list=25%, signal=36% | FRS2/RARB/HOXA13/MAML1/HEYL/HEG1/TRIP11/SMAD4/TBX1/SEMA3C/WNT11/HEY1/NPRL3/CRELD1/TGFB2/SAV1/NRP1/PLXND1/BMP4/ROBO2/HES1/JAG1/MATR3/TBX3/LMO4/DHRS3/SOX4/SLIT2/NOG/PARVA/ZBTB14/SLIT3/ANK2/FGFR2/BMPR1A/NPHP3/PDE2A/RBPJ/MDM4/TGFBR2/BMP7/ZFPM2/NOTCH2/TGFBR3/GATA3 |
| GOBP | REGULATION OF MEMBRANE PERMEABILITY | 81 | 0.44109119 | 1.696609967 | 0.000694589 | 0.005967066 | 0.004168092 | 4105 | tags=40%, list=20%, signal=32% | TFDP1/PPIF/SLC25A5/GZMB/HK2/BAK1/NMT1/DYNLT1/YWHAQ/TJP2/SFN/BLOC1S2/VDAC2/CNP/BID/RASIP1/CASP8/LAPTM5/BNIP3/TMEM14A/BOK/TP63/ALKBH7/PDCD6IP/YWHAZ/BAX/HIP1R/TMEM102/MAPK8/GSK3A/MTOR/ATP5IF1 |
| GOCC | TERTIARY GRANULE LUMEN | 55 | 0.492089786 | 1.78474479 | 0.000687394 | 0.005912823 | 0.004130203 | 2640 | tags=36%, list=13%, signal=32% | TCN1/LTF/LRG1/CSTB/ILF2/CXCL1/GGH/PRSS3/MMP9/ALDOA/DBNL/LYZ/CNN2/NIT2/CTSD/GSDMD/CAMP/CTSS/QSOX1/CYFIP1 |
| GOBP | CANONICAL WNT SIGNALING PATHWAY | 318 | -0.28677316 | -1.431283704 | 0.000683902 | 0.005890328 | 0.004114489 | 3828 | tags=36%, list=18%, signal=29% | TMEM170B/LRP5/BMP2/APC/PPM1B/EDA/ILK/GNAQ/PRKN/BTRC/JRK/PTEN/TNN/WNT5B/SFRP5/LGR4/RNF220/LGR6/NPHP4/PRICKLE1/WNT7B/DAB2/DACT3/PTPRU/FZD1/RSPO3/SOX10/LRRK2/MLLT3/SOX13/SOX4/WWTR1/RECK/YAP1/NKD1/STK3/WNK1/NOG/DVL2/FZD3/DACT1/DKK1/ARNTL/BCL9/FZD10/LGR5/LATS2/ZBED3/EGF/EGFR/FZD8/FZD7/ADGRA2/RYK/DDIT3/DKK3/GPRC5B/APOE/KLHL12/CSNK1D/LRP6/RNF146/FGFR2/GREM1/SMURF2/ANKRD6/USP34/KLF4/CAPRIN2/GSK3B/CCNY/ROR2/GPC3/FRZB/TLE5/NPHP3/DKK2/CDK14/IGFBP6/WNT2/PTPRO/SMAD3/RBPJ/RSPO1/LZTS2/FZD4/TNKS/DAAM2/MITF/TCF7L1/TLE4/WNK2/JADE1/ZNRF3/PFDN5/TLE2/DIXDC1/PLPP3/RBMS3/TCF7L2/TBL1XR1/MCC/ZNF703/LRP4/GLI3/CAV1/KANK1/WNT2B/TLE1/AXIN2/SCEL/WLS/GATA3 |
| GOMF | TRANSMEMBRANE RECEPTOR PROTEIN SERINE THREONINE KINASE ACTIVITY | 18 | -0.661010838 | -1.919806196 | 0.000677615 | 0.005843672 | 0.004081899 | 3930 | tags=50%, list=19%, signal=41% | ACVR2B/ACVR1C/ACVRL1/LTBP4/BMPR1A/BMPR1B/TGFBR2/ACVR1B/TGFBR3 |
| GOBP | NEGATIVE REGULATION OF PROTEIN CONTAINING COMPLEX ASSEMBLY | 132 | -0.352430304 | -1.584450967 | 0.000672858 | 0.005810106 | 0.004058453 | 4098 | tags=36%, list=20%, signal=29% | TRIM9/CLU/SORL1/LDLRAD4/TRIOBP/DMTN/RDX/ANKRD27/PRRT2/TMSB4X/LMO4/RPL13A/SLIT2/DACT1/DKK1/TRAF3IP1/KANK4/ADD1/TMOD2/TMSB15B/VILL/DYRK1A/PFN2/AIDA/CLIP3/SPTAN1/PMEPA1/GSK3B/MAP2/SNCA/THRA/SCIN/FHOD3/SVIL/SVIP/EPS8/SPTBN2/MYADM/ADD3/LMOD1/CTNNBIP1/JAM3/TMOD1/GSN/KANK1/SPTBN1/CRYAB/NOP53 |
| GOMF | ALPHA ACTININ BINDING | 24 | -0.606350422 | -1.920316265 | 0.000672392 | 0.005810106 | 0.004058453 | 2623 | tags=46%, list=13%, signal=40% | PDLIM2/RARA/PDLIM5/MYOT/PPARG/PKD2/DAG1/PDLIM3/MAGI1/SYNPO2/PDLIM4 |
| GOBP | NEGATIVE REGULATION OF DEFENSE RESPONSE | 191 | 0.367903848 | 1.593338703 | 0.000669159 | 0.005793052 | 0.00404654 | 4047 | tags=34%, list=19%, signal=27% | SERPINB4/NMI/NOD2/IFI16/PPARD/PBK/C1QBP/NLRX1/LDLR/SOCS3/GSTP1/MMP12/PTPN2/PARP14/SUSD4/UFD1/TARBP2/CST7/ARG1/NFKB1/TRIM21/MFHAS1/SLAMF8/IL12B/SERPINB9/DHX58/GPX1/PSMA1/AOAH/LYAR/LGALS9/TTLL12/CXCL17/ADAR/ITCH/GRN/IL2RA/LPCAT3/NLRC5/HLA-E/TNFRSF1B/TNFRSF1A/NLRC3/MICB/RB1/KLRD1/IRAK3/CD96/PRKCD/YTHDF2/HLA-F/SAMHD1/NPLOC4/SIGLEC10/HLA-B/LILRB1/NLRP3/HLA-G/OTULIN/IL10/RNF26/FOXP3/ILRUN/MAPK7 |
| GOBP | NADH METABOLIC PROCESS | 42 | 0.546326439 | 1.864826342 | 0.000667622 | 0.005787204 | 0.004042455 | 4360 | tags=50%, list=21%, signal=40% | ENO1/TPI1/PGAM1/PGK1/HK2/TIGAR/VCP/MDH2/ALDOA/FOXK2/GAPDH/GPI/PCK2/MDH1/PFKP/PKM/ADPGK/NUDT13/ENO3/HK3/SLC25A12 |
| GOBP | DNA REPLICATION CHECKPOINT | 17 | 0.684331856 | 1.909467155 | 0.000666861 | 0.005787204 | 0.004042455 | 2420 | tags=47%, list=12%, signal=42% | TIMELESS/CDC6/HUS1/CDC45/CDT1/ORC1/DNA2/CLSPN |
| GOBP | PYRIMIDINE NUCLEOSIDE BIOSYNTHETIC PROCESS | 16 | 0.711251299 | 1.969525117 | 0.000662407 | 0.005756857 | 0.004021258 | 1723 | tags=44%, list=8%, signal=40% | TYMP/UPP1/TK1/UCK2/DTYMK/PUDP/UMPS |
| GOBP | REGULATION OF PROTEIN MODIFICATION BY SMALL PROTEIN CONJUGATION OR REMOVAL | 228 | 0.344625077 | 1.519249574 | 0.000647089 | 0.005631014 | 0.003933354 | 3843 | tags=32%, list=18%, signal=26% | ZC3H12A/CDC20/NMI/NOD2/DCUN1D5/UBE2C/UBE2N/MAD2L1/VCP/ISG15/DTX3L/TRIP12/HIF1A/FANCI/UBE2L3/HSPA5/PPIA/CENPX/GNL3/COPS9/TBC1D7/OTUB2/UBE2S/PRMT3/TOLLIP/PINX1/GTPBP4/CHFR/AIMP2/SIRT7/RAB1A/ATG5/TRIM21/CDK5/UBE2D1/RBX1/PLK1/BIRC3/GLMN/CENPS/PDCD6/PHF23/COMMD1/PTPN22/SAE1/MAD2L2/ITCH/FBXO5/PLAA/HSPBP1/LAPTM5/TNIP1/SIAH2/PARK7/BRCA1/FBXO4/TSPO/PSMD10/PELI1/CDK9/ARRB1/BCL10/CTR9/GNL3L/DAXX/KDM1A/DCUN1D3/UBE2I/OTUB1/PARP10/HDAC3/GSK3A/SPHK1 |
| GOCC | RNA POLYMERASE COMPLEX | 99 | 0.416699751 | 1.655974325 | 0.000643286 | 0.005605179 | 0.003915308 | 4846 | tags=43%, list=23%, signal=34% | POLR3G/POLR2H/POLR2F/TAF10/POLR3K/POLR1H/MCM3/POLR3B/GTF2A2/POLR2L/POLR1C/LEO1/GTF2E2/TAF13/POLR2G/POLA2/POLR2D/POLR1B/PRIM1/POLR3D/CDK7/PAF1/POLR2E/POLR1G/GTF2E1/CCNH/TAF11/PEX2/GTF2B/CTR9/POLR2I/POLR1A/RPRD1B/TADA3/MYO6/POLR2J/ZNFX1/SUPT3H/TAF5/RPAP2/TAF2/POLR3A/POLR2J2 |
| GOBP | MYOFIBRIL ASSEMBLY | 57 | -0.461990709 | -1.801327886 | 0.000636346 | 0.005551916 | 0.003878103 | 3113 | tags=40%, list=15%, signal=34% | OBSL1/KRT8/CAPN3/KRT19/PDGFRB/EDN1/ACTC1/TMOD2/ACTA1/MYH3/AKAP13/FHOD3/CFL2/TNNT1/CSRP1/NEBL/PDGFRA/LMOD1/MYH11/TPM1/TMOD1/PGM5/MEF2A |
| GOBP | POSITIVE REGULATION OF CANONICAL WNT SIGNALING PATHWAY | 139 | 0.387928626 | 1.613575287 | 0.000627696 | 0.005483568 | 0.003830361 | 2337 | tags=30%, list=11%, signal=27% | PSME2/PSMB2/CDH3/PSMA5/PSMB5/RUVBL1/PSMA3/PSMD12/PSME4/PSMA2/PSME1/VCP/PSMB6/PSMB10/PSMB3/PSMD6/PSMB8/PSMC4/PSMA4/PSMD2/PSMD11/PSMD1/PSMD14/PSMC3/PSMB9/NFKB1/PSMA7/PSMD9/PSMD8/PSMA1/PSMD13/PSMF1/GSKIP/PSMC5/PSMC2/PSMB1/CSNK1G2/PSMC6/PSME3/PSMD7/CSNK1G3/PSMC1 |
| GOBP | NEGATIVE REGULATION OF ACTIN FILAMENT DEPOLYMERIZATION | 43 | -0.508021433 | -1.869481455 | 0.000624275 | 0.005460779 | 0.003814442 | 3750 | tags=44%, list=18%, signal=36% | TRIOBP/DMTN/PIK3CA/RDX/SWAP70/ADD1/TMOD2/PLEKHH2/VILL/SPTAN1/SCIN/SVIL/EPS8/SPTBN2/ADD3/LMOD1/TMOD1/GSN/SPTBN1 |
| GOBP | RESPONSE TO CALCIUM ION | 138 | -0.347880873 | -1.572283392 | 0.00062394 | 0.005460779 | 0.003814442 | 3788 | tags=32%, list=18%, signal=26% | TRPV6/DMTN/CPNE2/ENTPD6/CAPN3/SEC31A/SYT1/CALM2/CLIC4/ADGRV1/RASA4/CPNE8/KCNMB1/RYR3/EDN1/DUSP1/NLGN1/FOS/CPNE3/ADD1/JUND/EGFR/CACYBP/AKR1C3/ANXA11/ITPKB/PRKAA2/PKD2/MEF2C/SYT17/KCNMA1/IQGAP1/BRAF/ITPR3/TRPC1/CASQ2/CAV1/SYT8/MEF2A/KCNMB4/CCND1/EEF2K/TXNIP/CHP2 |
| GOBP | PROTEIN HOMOOLIGOMERIZATION | 172 | 0.36412772 | 1.559241711 | 0.000607561 | 0.005328453 | 0.00372201 | 4230 | tags=31%, list=20%, signal=25% | TK1/PYCARD/KCTD5/ALDH1A3/TNFAIP1/SHMT2/GBP5/MLKL/ECT2/PNPT1/HPRT1/ALDOA/PRF1/MIF/HSD17B10/OSBPL2/TDO2/KCTD11/SOD2/KCTD1/BLM/STEAP4/EHD4/POLQ/KCNJ2/ELAVL1/JMJD6/GSDMD/ACOT13/KCTD21/PDCD6IP/ACACA/KCTD4/KCNS3/KCND3/PRMT1/SIGMAR1/RNF213/BEND3/NLRC4/SHKBP1/B2M/ARC/SAMHD1/ROM1/ALOX5AP/HLA-G/KCNC3/CHMP2A/THG1L/SLC1A5/VASP/KCNG1 |
| GOBP | REGULATION OF NOTCH SIGNALING PATHWAY | 103 | -0.39488653 | -1.708197997 | 0.00060544 | 0.00531679 | 0.003713864 | 3048 | tags=33%, list=15%, signal=28% | ROBO2/HES1/DTX1/EGFL7/JAG1/YAP1/SNW1/IL6ST/CCN3/JAG2/TSPAN14/METTL3/EGF/EGFR/NEPRO/ENHO/CREBBP/SYNJ2BP/TM2D3/GALNT11/ZMIZ1/HIF1AN/RBPJ/FBXW7/BMP7/GATA2/CBFA2T2/BCL6/NOTCH2NLA/LFNG/KIT/POSTN/EPN2/MAML2 |
| GOBP | NADPH REGENERATION | 17 | 0.689585542 | 1.92412633 | 0.000595835 | 0.005239292 | 0.00365973 | 4085 | tags=65%, list=20%, signal=52% | PGAM1/PGM2/TIGAR/PGD/TALDO1/RBKS/SHPK/PGLS/G6PD/DERA/TKT |
| GOBP | PYRIMIDINE NUCLEOSIDE METABOLIC PROCESS | 34 | 0.565914842 | 1.857791737 | 0.000592843 | 0.005219811 | 0.003646123 | 1723 | tags=26%, list=8%, signal=24% | TYMP/UPP1/TK1/UCK2/NT5C3A/APOBEC3B/DTYMK/PUDP/UMPS |
| GOBP | POSITIVE REGULATION OF DNA METABOLIC PROCESS | 193 | 0.35441482 | 1.535030757 | 0.000587275 | 0.005177576 | 0.003616621 | 4965 | tags=38%, list=24%, signal=29% | PARP9/H2AX/UBE2N/FOXM1/PRKCQ/TIGAR/AURKB/CCT5/NEK2/TIMELESS/PCNA/DTX3L/RAD51/CCT2/HNRNPA2B1/CCT3/GNL3/CCT7/RAD51AP1/EXOSC3/MYC/PNKP/RFC2/IL6/ENDOG/ST20/RFC3/XRCC5/NSD2/MAD2L2/RNF168/PRKDC/TFRC/HMCES/BRCC3/UBE2V2/BABAM2/TCP1/DKC1/BRCA1/FBXO4/CCT6A/TMEM161A/ERCC8/PML/CCT8/BAX/ERCC1/SIRT6/PARP1/RNF8/RFC4/PRKCD/PTGES3/WAS/NABP2/RAD50/ATR/PARP3/SLF1/MAPKAPK5/WRAP53/CHTF18/TNFSF13/PTPRC/CD28/CD40/RFC5/MAPK3/TBX21/ARRB2/FUS/BABAM1 |
| GOBP | MATURATION OF SSU RRNA FROM TRICISTRONIC RRNA TRANSCRIPT SSU RRNA 5 8S RRNA LSU RRNA | 36 | 0.555067449 | 1.840783303 | 0.000576613 | 0.005090261 | 0.003555629 | 3675 | tags=47%, list=18%, signal=39% | MRPS11/RRP36/TSR1/DCAF13/BYSL/NOL10/WDR46/UTP4/NOP14/FAM207A/RPP40/UTP6/ABT1/TBL3/RRS1/HEATR1/UTP3 |
| GOBP | DNA INTEGRITY CHECKPOINT | 152 | 0.373588718 | 1.578087679 | 0.000569372 | 0.005032949 | 0.003515596 | 3136 | tags=32%, list=15%, signal=28% | CCNB1/CDK1/TFDP1/H2AX/TIMELESS/PCNA/AURKA/CDC6/HUS1/CDC45/FBXO6/PLK2/SFN/E2F8/DTL/CDT1/BRIP1/DONSON/CNOT11/CDKN1A/ORC1/PLK1/FANCD2/DNA2/PLK3/BLM/CNOT1/PRKDC/WDR76/CHEK2/BRCC3/BABAM2/CLSPN/TRIAP1/INTS7/THOC5/BRCA1/DOT1L/FBXO4/CDK2/CRADD/CDC25C/PML/BAX/GADD45A/PRMT1/MDM2/EME1/RFWD3 |
| GOMF | PSEUDOURIDINE SYNTHASE ACTIVITY | 13 | 0.742735717 | 1.924346059 | 0.000569178 | 0.005032949 | 0.003515596 | 4523 | tags=85%, list=22%, signal=66% | TRUB2/PUSL1/PUS10/PUS1/PUS7/DKC1/RPUSD1/TRUB1/RPUSD2/PUS3/RPUSD3 |
| GOMF | TUMOR NECROSIS FACTOR RECEPTOR BINDING | 31 | 0.594038194 | 1.892690943 | 0.000564349 | 0.005001708 | 0.003493774 | 4500 | tags=65%, list=22%, signal=51% | STAT1/TNFSF10/LTB/TRAP1/TRAF1/TRAF3/CASP8/FADD/BABAM2/SIVA1/TNFSF9/TNF/FASLG/TNFSF15/TRADD/TNFSF13B/TRAF4/TRAF2/TNFSF13/TNFSF14 |
| GOBP | POSITIVE REGULATION OF PHOSPHATIDYLINOSITOL 3 KINASE SIGNALING | 88 | -0.410032535 | -1.748638818 | 0.000557601 | 0.004948435 | 0.003456562 | 3778 | tags=43%, list=18%, signal=36% | TEK/KDR/TGFB2/ANGPT1/GPER1/PIK3CA/UBE3A/ERBB3/IL18/ERBB4/SIRT1/PDGFRB/AGT/INSR/LEP/F2R/IGF1R/ROR1/PDGFA/EGF/PTK2/NTF3/PIK3CB/NTRK2/FN1/FGFR1/PDGFD/ROR2/MYOC/DCN/FYN/PIK3R1/PDGFRA/KIT/IRS2/CAT/SERPINA12/PDGFC |
| GOBP | ACTIVATION OF CYSTEINE TYPE ENDOPEPTIDASE ACTIVITY INVOLVED IN APOPTOTIC PROCESS | 85 | 0.444472361 | 1.720936538 | 0.000557363 | 0.004948435 | 0.003456562 | 3496 | tags=42%, list=17%, signal=35% | S100A9/CYCS/S100A8/FAM162A/PYCARD/VCP/BAK1/CFLAR/TNFSF10/LCK/AIFM1/HSPE1/XDH/BID/CASP10/PDCD6/ST20/TNFRSF10A/BEX3/CASP8/TNFRSF10B/FADD/LAPTM5/BOK/HSPD1/TNF/DIABLO/CRADD/PML/FASLG/BAX/TNFSF15/TRADD/HIP1R/NLRC4/PDCD2 |
| GOBP | CELLULAR DEFENSE RESPONSE | 51 | 0.513018984 | 1.837790858 | 0.000557048 | 0.004948435 | 0.003456562 | 4509 | tags=49%, list=22%, signal=39% | CXCR2/LGALS3BP/CXCL9/PRF1/FOSL1/GNLY/ADORA2B/CCR5/SH2D1A/NCF2/ITK/FCMR/LY96/IL1RL2/TYROBP/BCL10/CCR2/LILRB2/BECN1/MNDA/HLA-G/NCR2/KLRG1/KLRC3/IL33 |
| GOBP | GENE SILENCING | 268 | 0.330316798 | 1.479209878 | 0.000553176 | 0.004928692 | 0.003442771 | 4929 | tags=36%, list=24%, signal=28% | ZC3H12A/RAN/H2AX/EIF4E2/MACROH2A1/H2AZ1/EIF6/POLR2H/EZH2/CDC45/HNRNPA2B1/NCBP1/NUP37/POLR2F/STAT3/NUP210/ZMPSTE24/TARBP2/NUP88/ATAD2/HENMT1/MIR155HG/H2AJ/CNOT11/MIR3142HG/PUS10/NUP50/MSL3/IL6/POLR2L/HAT1/TUT7/CNOT1/ZFP36/ADAR/POLR2G/DPY30/ELAVL1/POLR2D/SND1/RAE1/XPO5/ERI1/SEC13/SPOUT1/MACROH2A2/H3C10/DOT1L/NRDE2/CLP1/NUP155/POLR2E/HDAC2/TNF/CDK2/MYCN/NDC1/SIRT6/HMGA1/EIF4G1/EED/NUP188/CNOT6/MRPL44/NUP85/POLR2I/MORF4L2/H2AC4/NUP205/TDRKH/MTHFR/H2AC13/CHMP1A/NUP58/MAP2K1/NUP107/NUP93/MIR22HG/FKBP6/H1-2/POM121/POLR2J/H3C7/NUP160/H3C8/H4C4/H2AC16/H4C9/NUP35/DYDC2/MAP2K2/CNOT10/TDRD9/MIR34AHG/NUP42/H1-5/MIR17HG |
| GOBP | PURINE CONTAINING COMPOUND BIOSYNTHETIC PROCESS | 197 | 0.365635396 | 1.58564103 | 0.000542296 | 0.004838172 | 0.003379541 | 3901 | tags=32%, list=19%, signal=27% | ENO1/ATP5MF/PNP/AK2/NME1/ACOT7/VCP/ATP5MC3/ATP5PF/GART/ATP5F1B/STOML2/ATP5MC1/SHMT2/APRT/ATIC/IMPDH1/HPRT1/MTHFD1/ATP5PB/ALDOA/SLC25A13/ELOVL7/AMPD3/ADSL/NOS2/ATP5ME/COASY/AK1/ATP5MG/ATP5F1C/ADK/PPT2/UQCC3/HACD1/GSTZ1/ATP5PO/ATP6V1A/GMPS/ANTKMT/ADA2/ACACA/AMD1/PDHB/ADCY3/GCDH/TREM2/PPCDC/PKM/ATP5F1D/PARP1/PRPSAP2/ATP5F1A/ELOVL4/DLAT/PAPSS2/DLD/ATP5PD/ELOVL1/PANK2/VPS9D1/NME7/GMPR2/PRPS2 |
| GOMF | CYSTEINE TYPE ENDOPEPTIDASE ACTIVITY INVOLVED IN APOPTOTIC PROCESS | 15 | 0.71306358 | 1.928721885 | 0.000538792 | 0.004813303 | 0.00336217 | 3226 | tags=60%, list=15%, signal=51% | CASP4/PYCARD/CASP1/CASP7/CFLAR/CASP5/CASP10/CASP8/CASP3 |
| GOBP | ATTACHMENT OF MITOTIC SPINDLE MICROTUBULES TO KINETOCHORE | 15 | 0.713143811 | 1.928938898 | 0.000538792 | 0.004813303 | 0.00336217 | 3567 | tags=60%, list=17%, signal=50% | NDC80/KIF2C/AURKB/NUF2/CENPE/CDT1/RMDN1/MIS12/BECN1 |
| GOBP | REGULATION OF DNA DEPENDENT DNA REPLICATION | 46 | 0.516274776 | 1.794675202 | 0.000533487 | 0.00477862 | 0.003337944 | 3251 | tags=43%, list=16%, signal=37% | TIMELESS/PCNA/STOML2/E2F8/DBF4/ZMPSTE24/CDT1/DONSON/WDR18/RFC2/BLM/RFC3/BRCA2/ATG7/CDC7/FBXO5/CHEK2/MSH3/METTL4/RFC4 |
| GOBP | MUSCLE CELL DEVELOPMENT | 155 | -0.346866058 | -1.589430549 | 0.000529306 | 0.004747499 | 0.003316204 | 4123 | tags=32%, list=20%, signal=26% | DNER/OBSL1/KRT8/HDAC9/RAMP2/PARP2/EFEMP2/CAPN3/KRT19/BMP4/HES1/PDGFRB/SMYD3/LOX/TBX3/BVES/RGS2/PDLIM5/SHOX2/EDN1/ACTC1/TMOD2/ACTA1/MYH3/ANK2/BIN1/NFATC2/SORBS2/PPARA/SLC8A1/AKAP13/SKI/ADRA1A/FHOD3/CFL2/TNNT1/FLNC/CSRP1/SGCB/NEBL/PDGFRA/LMOD1/DMD/MYH11/TPM1/TMOD1/PGM5/CAV2/BCL2/MEF2A |
| GOBP | NEGATIVE REGULATION OF TYPE I INTERFERON PRODUCTION | 45 | 0.530834589 | 1.839839766 | 0.000524679 | 0.004712289 | 0.00329161 | 4034 | tags=53%, list=19%, signal=43% | NMI/UBE2L6/NLRX1/PYCARD/IFIH1/ISG15/UFD1/RELB/IKBKE/TBK1/DHX58/ITCH/HERC5/NLRC5/REL/LILRA4/NLRC3/DDX58/TRIM25/NPLOC4/LILRB1/UBA7/IL10/ILRUN |
| GOBP | ACTIN FILAMENT BASED MOVEMENT | 145 | -0.34389606 | -1.565831039 | 0.000522884 | 0.00470246 | 0.003284744 | 2151 | tags=24%, list=10%, signal=22% | CACNB2/ACTC1/PARVA/FGF13/ACTA1/MYH3/SYNE2/EPDR1/TNNC2/DSG2/FNBP1L/ANK2/DSP/BIN1/MYO5C/MYH14/TNNT1/PLN/AKAP9/TPM2/SCN3B/TNNC1/ATP1A2/VIM/DES/TNNI2/LIMCH1/MYH10/GPD1L/CACNA2D1/DMD/TPM1/TMOD1/GSN/CAV1 |
| GOMF | RIBOSOME BINDING | 53 | 0.506889495 | 1.824938814 | 0.000511862 | 0.004609505 | 0.003219814 | 4674 | tags=55%, list=22%, signal=43% | ZC3H12A/C1QBP/EIF6/EIF2S1/HSPA5/TACO1/NAA10/PYM1/MAIP1/PRMT3/SRP19/EIF3K/SHFL/SEC61A1/ERI1/MRPS27/ZNF598/SRP72/RPN2/OLA1/EIF5A/NAA15/MTOR/CPEB4/EFL1/SRP68/SEC61A2/CPEB3/OXA1L |
| GOMF | ENDONUCLEASE ACTIVITY | 112 | 0.408193361 | 1.656184952 | 0.000503454 | 0.004539868 | 0.003171171 | 5625 | tags=47%, list=27%, signal=35% | ZC3H12A/RNASEH2A/FEN1/DNASE1L3/PGBD5/EXO1/APEX2/CPSF3/RIDA/PNKP/DIS3/ELAC2/ENDOU/ZC3H12C/DNA2/RNASE7/ZC3H12D/ENDOG/GEN1/RPP40/POP5/SND1/POP4/TSEN34/POP1/NOB1/ERCC1/EME1/KHNYN/MRPL44/DNASE1L1/REXO4/RAD50/SLFN13/POP7/RPP30/RPP38/EXOG/DNASE1L2/RAD51C/TSEN2/PXDNL/N4BP2/ENDOV/NTHL1/PELO/APEX1/RNASET2/APLF/EME2/ZRANB3/RBBP8/PIWIL1 |
| GOBP | TRNA MODIFICATION | 85 | 0.446853404 | 1.73015561 | 0.000502653 | 0.004538743 | 0.003170386 | 4521 | tags=45%, list=22%, signal=35% | WDR4/MTO1/FTSJ1/PUSL1/METTL6/METTL1/HSD17B10/TRMT10C/ELP6/PUS10/DUS1L/TPRKB/MOCS3/TYW5/NSUN2/PUS1/TRMT6/TRMT112/METTL8/TRMU/CTU2/PUS7/MTFMT/NAT10/OSGEP/DUS2/TRMT1/LCMT2/TRUB1/DUS3L/THUMPD3/TYW3/THG1L/QTRT2/ADAT3/ELP5/TRMT5/PUS3 |
| GOBP | REGULATION OF MIRNA METABOLIC PROCESS | 10 | 0.788710156 | 1.904664735 | 0.000498282 | 0.004505328 | 0.003147044 | 2093 | tags=40%, list=10%, signal=36% | ZC3H12A/PNPT1/NFKB1/HRAS |
| GOBP | PROTEIN LOCALIZATION TO CILIUM | 58 | -0.467556988 | -1.828911876 | 0.000497814 | 0.004505328 | 0.003147044 | 4701 | tags=50%, list=23%, signal=39% | INPP5E/TBC1D32/BBIP1/ARL6/ZDHHC3/SPATA7/ROPN1/RABEP1/FAM149B1/NPHP4/TCTN1/EFCAB7/BBS9/TTC21B/DZIP1L/DYNC2H1/DZIP1/TULP3/TUB/CSNK1D/ARL3/BBS1/CROCC/RAB11FIP3/IFT122/WDR35/LZTFL1/WDR19/ZNF423 |
| GOBP | RESPONSE TO HEAT | 152 | 0.375511964 | 1.58621172 | 0.000484781 | 0.004395082 | 0.003070036 | 4816 | tags=43%, list=23%, signal=34% | EIF2B2/DNAJB6/VCP/RPA3/EIF2S1/DNAJA2/CXCL10/ATP2A2/NUP37/IRAK1/NUP210/HSBP1/NUP88/CLPB/IER5/BAG3/LYN/HMOX1/HSPA8/CDKN1A/HSPH1/PDCD6/NUP50/HIKESHI/DNAJB1/POLR2D/EIF2B1/RAE1/SEC13/DNAJC7/TFEC/EIF2B4/CHORDC1/NUP155/HDAC2/DNAJA3/TRPV3/MICB/NDC1/MLST8/HTRA2/DNAJC2/NUP188/NUP85/GCLC/PTGES3/FKBP4/EIF2B5/DAXX/MAPKAPK2/NUP205/EIF2B3/NUP58/MTOR/NUP107/NUP93/ATR/POM121/RPA1/NUP160/AKT1S1/DNAJA1/NUP35/MAPK3/SST/NUP42 |
| GOBP | POSITIVE REGULATION OF MONONUCLEAR CELL MIGRATION | 61 | 0.483436167 | 1.774185979 | 0.000482941 | 0.004384319 | 0.003062518 | 3874 | tags=48%, list=19%, signal=39% | WNT5A/CCL20/C1QBP/S100A7/PYCARD/CCR7/CXCL13/CXCL10/CCL4/ADAM17/S100A14/DEFB124/ITGA4/LGALS9/CCL7/CXCL17/DOCK8/OXSR1/ADAM10/ADAM8/FADD/CCL5/TNF/XCL1/CCR1/CCR2/LGMN/TMEM102/SERPINE1 |
| GOBP | IMP BIOSYNTHETIC PROCESS | 10 | 0.790260712 | 1.90840919 | 0.000482078 | 0.004382409 | 0.003061184 | 1143 | tags=50%, list=5%, signal=47% | GART/ATIC/HPRT1/AMPD3/ADSL |
| GOBP | REGULATION OF MUSCLE CONTRACTION | 151 | -0.347360335 | -1.584964342 | 0.000480387 | 0.004372955 | 0.003054579 | 2662 | tags=26%, list=13%, signal=23% | RGS2/MYL9/ATP2B1/EDN1/F2R/DOCK5/PPP1R12B/DOCK4/TACR1/FGF13/TNNC2/DSG2/ANK2/DSP/BIN1/ARHGAP42/SLC8A1/P2RX1/ORMDL3/MYOCD/KCNMA1/ADRA1A/PRKG1/TNNT1/PLN/AKAP9/TNNC1/ATP1A2/ADRA2A/TNNI2/CASQ2/CNN1/DMD/TPM1/C12orf57/KIT/CAV1/SOD1/ADRB2 |
| GOBP | REGULATION OF LEUKOCYTE DIFFERENTIATION | 267 | 0.335872688 | 1.503633405 | 0.000472185 | 0.004304126 | 0.003006502 | 4929 | tags=40%, list=24%, signal=31% | ZC3H12A/NFKBIZ/IRF7/PNP/LTF/GPR68/PRELID1/IL4R/NME1/HMGB3/PLA2G3/CD83/PPARGC1B/VNN1/IL20/PTPN2/IRF1/PRDM1/CD2/SASH3/CEBPB/RHOH/AP3D1/JAK3/IFNG/MYC/LYN/SOCS1/HAX1/CBFB/SLAMF8/IL12B/XRCC6/FANCD2/ZC3H8/SYK/IKZF3/IL7R/LGALS9/CCL19/CD27/AP3B1/IL23A/LAG3/PRKDC/IL2RA/PGLYRP2/GPR137B/CASP8/ADAM8/FADD/LILRB4/TMEM64/TNFSF9/CD86/CRTAM/TMEM131L/HCLS1/TNF/LEF1/IL36B/NFKBID/TREM2/RB1/IL1RL2/PGLYRP3/CCR1/ANXA1/HLA-DRA/IL12RB1/TYROBP/CCR2/LILRB2/FOXN1/IL15/TMEM176B/CARD11/HLA-B/ZAP70/LILRB1/C1QC/NLRP3/HLA-G/MTOR/CD80/IL10/FOXP3/XBP1/ZNF683/CR1/PTPRC/CD28/MALT1/FANCA/HLA-DOA/TRIB1/NKAP/AGER/INHA/PPP2R3C/FSHB/TMEM176A/TBX21/POU4F2/RASSF2/IFNA2/CAMK4/MIR17HG |
| GOMF | TRANSMEMBRANE RECEPTOR PROTEIN TYROSINE KINASE ACTIVITY | 61 | -0.454509793 | -1.776652593 | 0.000469891 | 0.004289028 | 0.002995955 | 4635 | tags=57%, list=22%, signal=45% | EPHA7/FLT1/MST1R/CSF1R/EPHB3/DDR1/FGFRL1/TEK/CRIM1/KDR/NRP1/EFNA3/FGFR3/EFNB3/ERBB3/ERBB4/PDGFRB/MERTK/DDR2/INSR/IGF1R/EPHB6/MET/EGFR/PDGFRL/FGFR2/EFNA4/NTRK2/FGFR1/ROR2/EPHB1/EFEMP1/AXL/PDGFRA/KIT |
| GOBP | EMBRYONIC DIGIT MORPHOGENESIS | 51 | -0.486729462 | -1.849907213 | 0.00046878 | 0.004284709 | 0.002992939 | 4466 | tags=51%, list=21%, signal=40% | MSX1/TBC1D32/LRP5/C2CD3/BPNT2/FREM2/IFT52/BMP4/TBX3/ECE1/NOG/TWIST1/LNPK/MAP3K20/TULP3/CREBBP/BMPR1A/ROR2/HOXA11/WDPCP/OSR1/ZBTB16/GLI2/LRP4/GLI3/OSR2 |
| GOBP | MIDDLE EAR MORPHOGENESIS | 17 | -0.678749016 | -1.967327545 | 0.000468359 | 0.004284709 | 0.002992939 | 4986 | tags=71%, list=24%, signal=54% | NKX3-2/TBX1/MSX1/PRKRA/INSIG1/PRRX1/EDN1/NOG/TSHZ1/OSR1/RPL38/OSR2 |
| GOBP | DEOXYRIBONUCLEOTIDE METABOLIC PROCESS | 39 | 0.544136227 | 1.82520878 | 0.000467941 | 0.004284709 | 0.002992939 | 1988 | tags=36%, list=10%, signal=33% | TYMP/RRM2/UPP1/NUDT15/TYMS/CMPK2/DTYMK/TDG/XDH/ADK/RRM1/DCTPP1/SMUG1/NUDT1 |
| GOBP | EAR DEVELOPMENT | 195 | -0.315928163 | -1.4835561 | 0.000466757 | 0.00428371 | 0.00299224 | 4040 | tags=31%, list=19%, signal=25% | TGFB2/KCNK2/BMP2/PJVK/IFT27/PRRX1/TRIOBP/FREM2/MAFB/LIN7A/ATP6V1B1/BMP4/ADGRV1/HES1/PDGFRB/TFAP2A/JAG1/AHI1/ECE1/EDN1/NOG/DVL2/FZD3/ROR1/TSKU/JAG2/LGR5/TWIST1/FGFR2/SEC24B/CXCL14/SLC25A27/SOBP/ROR2/FRZB/NTN1/TSHZ1/MYCL/MAF/RBPJ/LRIG1/MAPK1/LRIG3/SDC4/WDPCP/GATA2/OSR1/RPL38/DCHS1/ANP32B/MCOLN3/WDR19/SLITRK6/GLI2/GLI3/SPRY2/BCL2/OSR2/SOD1/GATA3 |
| GOBP | REGULATION OF MITOCHONDRION ORGANIZATION | 148 | 0.392073455 | 1.648884938 | 0.000463277 | 0.004257584 | 0.002973991 | 3775 | tags=38%, list=18%, signal=31% | TFDP1/PPIF/SLC25A5/GZMB/FZD5/PRELID1/GHITM/TIGAR/FAM162A/PYCARD/BAK1/HIF1A/NMT1/BIK/YWHAQ/MMP9/TNFSF10/SFN/CYRIB/MARCHF5/SIRT7/BID/HAX1/PDCD5/GPX1/PRMT6/STAT2/PLAUR/TFRC/CASP8/VDAC1/BNIP3/TMEM14A/BOK/TP63/TRIAP1/YWHAZ/PARK7/SREBF2/TREM2/MFF/BAX/FXN/HTRA2/MIEF1/TSPO/PSMD10/ZDHHC6/HIP1R/MYO19/CIDEB/HTT/TMEM102/MAPK8/SREBF1/GSK3A |
| GOBP | SNRNA TRANSCRIPTION | 74 | 0.464281015 | 1.750392387 | 0.000462505 | 0.004256316 | 0.002973105 | 4345 | tags=41%, list=21%, signal=32% | NABP1/POLR2H/ELL2/NCBP1/POLR2F/ZC3H8/INTS13/GTF2A2/POLR2L/GTF2E2/TAF13/POLR2G/POLR2D/ELL/INTS7/CDK7/POLR2E/GTF2E1/INTS5/TAF11/INTS9/CDK9/GTF2B/INTS14/POLR2I/NABP2/RPRD1B/CCNK/POLR2J/TAF5 |
| GOBP | PSEUDOURIDINE SYNTHESIS | 18 | 0.690076193 | 1.956946049 | 0.000461582 | 0.004253642 | 0.002971238 | 4523 | tags=83%, list=22%, signal=65% | NHP2/NOP10/TRUB2/PUSL1/PUS10/PUS1/GAR1/PUS7/DKC1/RPUSD1/TRUB1/TSR3/RPUSD2/PUS3/RPUSD3 |
| GOMF | CCR CHEMOKINE RECEPTOR BINDING | 30 | 0.602546759 | 1.901059487 | 0.000459239 | 0.004237863 | 0.002960216 | 3438 | tags=57%, list=16%, signal=47% | CCL20/CXCL13/CNIH4/CCL18/CCL4/CCL22/CCL2/CCL8/NARS1/CCL7/CCL19/CCL5/NES/XCL1/CCRL2/CCR2/CCL17 |
| GOBP | SPINAL CORD DEVELOPMENT | 94 | -0.398650217 | -1.70475751 | 0.000457296 | 0.004225741 | 0.002951749 | 3432 | tags=27%, list=16%, signal=22% | PTBP2/IFT172/RELN/TCTN1/LMO4/SOX13/SOX4/DYNC2H1/NOG/ZC4H2/TULP3/PTPRS/HOXC10/GDF7/PKD2/PTN/ADARB1/VIT/GATA2/PTCH1/DAAM2/PKD1/GLI2/GLI3/SOX6 |
| GOBP | NEGATIVE REGULATION OF DEVELOPMENTAL GROWTH | 98 | -0.396267265 | -1.703857369 | 0.000450571 | 0.004169333 | 0.002912346 | 3748 | tags=40%, list=18%, signal=33% | MSTN/WWC3/SAV1/SOCS2/SEMA6D/PTEN/VGLL4/NRP1/FGFR3/RAI1/BMP4/JARID2/RTN4R/RGS2/SEMA3E/NKD1/STK3/NOG/SPART/SEMA4G/SEMA6A/ADRB1/DUSP10/FGF13/SEMA3B/RYK/PTPRS/PPARA/RBP4/MAP2/NTN1/CDKN1B/RTN4/TGFBR2/PTCH1/SEMA3G/BBS2/MEIS1/ADRB2 |
| GOBP | POSITIVE REGULATION OF CELL SUBSTRATE JUNCTION ORGANIZATION | 30 | -0.584964047 | -1.982647777 | 0.00044452 | 0.004119014 | 0.002877198 | 4390 | tags=57%, list=21%, signal=45% | MAPRE2/TEK/KDR/NRP1/IQSEC1/ABL1/S100A10/DUSP3/FMN1/COL16A1/IQGAP1/SMAD3/FERMT2/SDC4/MYOC/TSC1/PIK3R1 |
| GOBP | TRNA TRANSPORT | 35 | 0.568664522 | 1.883232051 | 0.000444398 | 0.004119014 | 0.002877198 | 4816 | tags=63%, list=23%, signal=48% | RAN/NUP37/NUP210/NUP88/NUP50/XPOT/NOL6/RAE1/SEC13/NUP155/NDC1/YBX1/NUP188/NUP85/NUP205/NUP58/NUP107/NUP93/POM121/NUP160/NUP35/NUP42 |
| GOBP | NATURAL KILLER CELL ACTIVATION INVOLVED IN IMMUNE RESPONSE | 29 | 0.611496174 | 1.91889455 | 0.000441561 | 0.004102913 | 0.002865951 | 5627 | tags=48%, list=27%, signal=35% | RAB27A/CORO1A/IL12B/PGLYRP2/VAMP7/PGLYRP3/HLA-F/CD244/UNC13D/ZNF683/IFNE/IFNA2/IFNK/IFNW1 |
| GOBP | NEGATIVE REGULATION OF LYMPHOCYTE ACTIVATION | 143 | 0.393791005 | 1.647452517 | 0.000440488 | 0.004098614 | 0.002862948 | 4262 | tags=38%, list=20%, signal=30% | ZC3H12A/CD274/IL4R/TNFRSF21/HMGB3/IDO1/PTPN2/IRF1/PLA2G2F/SAMSN1/ARG1/CEBPB/JAK3/LYN/SOCS1/CBFB/TIGIT/ZC3H8/GLMN/LGALS9/PTPN22/LAG3/IL2RA/PGLYRP2/LILRB4/LAPTM5/CD86/CRTAM/TMEM131L/SCRIB/SFTPD/XCL1/PGLYRP3/CLEC4G/ANXA1/PELI1/CASP3/TYROBP/TNFRSF13B/LILRB2/PAG1/HLA-F/TBC1D10C/MNDA/LILRB1/HLA-G/CD300A/CD80/IL10/FOXP3/FGR/PARP3/LST1/CR1 |
| GOCC | CILIARY TRANSITION ZONE | 68 | -0.438435362 | -1.758425887 | 0.000436451 | 0.004066678 | 0.00284064 | 5478 | tags=49%, list=26%, signal=36% | UNC119B/CCDC66/CEP290/IFT140/TOPORS/NPHP1/TMEM216/CDKL1/KIF17/CFAP36/DYNC2LI1/SPATA7/KIAA1549/IFT52/NPHP4/SEPTIN2/TCTN1/BBS9/LCA5/AHI1/IQCB1/CFAP410/CPLANE1/TRAF3IP1/MACIR/TMEM80/CIBAR1/ARL3/PCM1/TTBK2/IFT122/WDR19/CCSAP |
| GOMF | STRUCTURAL CONSTITUENT OF RIBOSOME | 153 | 0.38550661 | 1.62876063 | 0.000432724 | 0.004037559 | 0.0028203 | 2788 | tags=36%, list=13%, signal=31% | MRPL15/MRPL51/RPL26L1/MRPL35/MRPS11/MRPS7/MRPL47/MRPL13/MRPS16/MRPS15/MRPL46/MRPS12/MRPL37/MRPS17/MRPL3/MRPL27/MRPL36/RPL22L1/MRPL11/MRPL17/MRPL42/MRPS25/NDUFA7/MRPL52/MRPL32/MRPL14/MRPS21/MRPS22/MRPS14/MRPL20/MRPS33/MRPL54/MRPS18C/MRPL19/MRPL4/MRPL16/MRPL22/MRPS36/MRPL12/MRPL21/MRPL41/MRPL23/MRPL28/MRPS2/MRPS35/MRPS6/MRPL18/MRPS30/MRPL55/MRPL57/MRPS18A/MRPS23/MRPS34/RPL39L/MRPL34 |
| GOBP | INTERLEUKIN 17 PRODUCTION | 35 | 0.568990248 | 1.884310751 | 0.000429708 | 0.004014989 | 0.002804535 | 1898 | tags=31%, list=9%, signal=29% | NOD2/MYD88/IL36RN/PRKCQ/PHB/TUSC2/SLC7A5/IFNG/IL12B/IL6/IL23A |
| GOBP | REGULATION OF WNT SIGNALING PATHWAY | 347 | -0.277866207 | -1.409240794 | 0.000428899 | 0.00401301 | 0.002803152 | 3828 | tags=33%, list=18%, signal=28% | APC/PPM1B/EDA/CBY1/ILK/GNAQ/PRKN/BTRC/JRK/VGLL4/TNN/WNT5B/SFRP5/LGR4/RNF220/LGR6/NPHP4/PRICKLE1/APP/ABL1/DAB2/DACT3/PTPRU/RSPO3/SOX10/RNF43/LRRK2/MLLT3/SOX13/SOX4/WWTR1/RECK/YAP1/NKD1/MACF1/STK3/WNK1/NOG/DVL2/DACT1/DKK1/TSKU/ARNTL/LGR5/ESR1/HIC1/LATS2/ZBED3/EGF/EGFR/FZD7/ADGRA2/DDIT3/DKK3/GPRC5B/APOE/KLHL12/CSNK1D/LRP6/APCDD1/RNF146/FGFR2/GREM1/SMURF2/ANKRD6/CDC73/USP34/CAPRIN2/GSK3B/CCNY/ROR2/GPC3/FRZB/TLE5/NPHP3/DKK2/CDK14/IGFBP6/SKI/PTPRO/SMAD3/RBPJ/RSPO1/LZTS2/SPIN1/FZD4/TNKS/DAAM2/TCF7L1/KLF15/TLE4/WNK2/MDFIC/JADE1/ZNRF3/PFDN5/TLE2/NFATC1/DIXDC1/CMAHP/PLPP3/RBMS3/TCF7L2/CTNNBIP1/TBL1XR1/MCC/ZNF703/LRP4/GLI3/CAV1/KANK1/TLE1/AXIN2/SCEL/WIF1/WLS |
| GOBP | PROTEIN IMPORT INTO MITOCHONDRIAL MATRIX | 17 | 0.69752688 | 1.946284767 | 0.000422217 | 0.003956005 | 0.002763333 | 4029 | tags=82%, list=19%, signal=67% | GRPEL1/TIMM50/TIMM17A/TIMM23/TOMM40L/TOMM40/ROMO1/DNAJC15/TIMM44/TIMM21/GRPEL2/DNLZ/TIMM17B/DNAJC19 |
| GOBP | TELOMERE MAINTENANCE VIA TELOMERE LENGTHENING | 75 | 0.461417725 | 1.742949215 | 0.000420791 | 0.003948144 | 0.002757843 | 4208 | tags=45%, list=20%, signal=36% | PRKCQ/AURKB/CCT5/NEK2/NHP2/RAD51/CCT2/HNRNPA2B1/CCT3/NOP10/HNRNPC/CCT7/STN1/PINX1/PNKP/XRCC5/PIF1/GAR1/TCP1/DKC1/FBXO4/CCT6A/NAT10/CCT8/PARP1/TINF2/TELO2/GNL3L/PTGES3/RAD50/ATR/MAPKAPK5/WRAP53/RPA1 |
| GOBP | TRANSCRIPTION BY RNA POLYMERASE I | 67 | 0.484142296 | 1.79963047 | 0.000417554 | 0.003923254 | 0.002740456 | 5250 | tags=54%, list=25%, signal=40% | MACROH2A1/IPPK/POLR2H/POLR2F/MARS1/ZMPSTE24/SIRT7/POLR1H/PIH1D1/LYAR/POLR2L/POLR1C/TAF1D/BNC1/DHX33/POLR1B/ATF4/MACROH2A2/CDK7/UTP15/POLR2E/POLR1G/SMARCA4/CCNH/NOL11/HEATR1/POLR1A/MTOR/PWP1/TAF1B/NCL/MAPK3/TCOF1/GTF2H1/ERCC6/WDR75 |
| GOCC | PRECATALYTIC SPLICEOSOME | 52 | 0.51628525 | 1.850437082 | 0.000417554 | 0.003923254 | 0.002740456 | 2761 | tags=40%, list=13%, signal=35% | SNRPG/SNRPF/SNRPD1/LSM2/TXNL4A/SF3B3/LSM7/SNRPD3/EFTUD2/SF3B5/SNU13/SNRPA1/MAGOHB/SF3B6/LSM5/LSM4/SNRPB/PRPF31/PRPF38A/LSM6/PRPF4 |
| GOBP | RESPONSE TO GAMMA RADIATION | 52 | 0.51654384 | 1.851363905 | 0.000417554 | 0.003923254 | 0.002740456 | 4406 | tags=60%, list=21%, signal=47% | H2AX/TIGAR/POLB/BAK1/RAD51/HSPA5/CXCL10/ZMPSTE24/MYC/CDKN1A/XRCC6/FANCD2/GPX1/XRCC5/CCL7/BRCA2/HRAS/PRKDC/CHEK2/LIG4/MEN1/PML/BAX/PARP1/MDM2/KDM1A/DCUN1D3/ELK1/ATR/TLK2/PTPRC |
| GOBP | NEGATIVE REGULATION OF EXTRINSIC APOPTOTIC SIGNALING PATHWAY VIA DEATH DOMAIN RECEPTORS | 38 | 0.556398827 | 1.863649239 | 0.000415687 | 0.003922167 | 0.002739697 | 4079 | tags=47%, list=20%, signal=38% | CFLAR/TNFSF10/FAIM/HMOX1/GPX1/TNFRSF10A/CASP8/TNFRSF10B/FADD/HMGB2/PARK7/BRCA1/FASLG/TRADD/ICAM1/ITPRIP/SERPINE1/TRAF2 |
| GOBP | EPIDERMIS DEVELOPMENT | 371 | 0.313726064 | 1.443000198 | 0.000414411 | 0.003915626 | 0.002735128 | 3288 | tags=25%, list=16%, signal=22% | KRT16/DSC2/PI3/KRT6A/HPSE/KLK13/DSG3/FABP5/SERPINB13/LCE3D/TGM1/SPRR1B/CDH3/WNT5A/EPHA2/ZDHHC21/TFDP1/SPRR3/KRT6B/CERS3/PPARD/CNFN/CRABP2/S100A7/SPRR1A/IVL/HDAC1/MACROH2A1/GRHL3/ABCA12/PRKCH/TGM3/EZH2/KRT17/TMEM79/FERMT1/ST14/CSTA/TRIM16/ZBED2/IL20/ATP2A2/SPRR2G/BCR/SFN/CTSL/ZMPSTE24/CYP27B1/CBFB/CAPN1/KRTDAP/PRSS8/KLK12/SULT2B1/KLK7/AP3B1/NSUN2/PCSK6/TRPC4AP/OVOL2/KRT24/OVOL1/ZFP36/BNC1/SLC9A3R1/EREG/ETV4/KRT78/PKP3/WNT10A/CALML5/FLOT2/GRHL1/TP63/AQP3/HOXC13/KRT37/FURIN/NSDHL/MACROH2A2/PLAAT4/KEAP1/HDAC2/TNF/ADAM9/RPTN/STS/UGCG/YBX1/ANXA1/TRADD/CASP3/KRT14/DKK4 |
| GOBP | ANIMAL ORGAN FORMATION | 59 | -0.464715335 | -1.810015527 | 0.000412354 | 0.003901681 | 0.002725387 | 3262 | tags=37%, list=16%, signal=32% | FGF1/BMP4/ROBO2/HES1/NOG/NTF4/DKK1/FGFR2/BMPR1A/MEF2C/HOXA11/WNT2/RBPJ/MAPK1/TGFBR2/BMP7/SPRY1/GLI2/AR/GLI3/WNT2B/AXIN2 |
| GOCC | CELL CELL JUNCTION | 471 | -0.261729433 | -1.361127284 | 0.00041087 | 0.003893123 | 0.002719409 | 4764 | tags=33%, list=23%, signal=26% | PERP/PDLIM2/FLOT1/PIP5K1C/CDC42BPB/DSC1/SCN1B/SYNPO/AKAP6/PAK2/POF1B/FGFRL1/TEK/AQP7/CCDC85B/GJB5/SHROOM4/TMEM65/FLRT2/CTNNB1/TRPC6/CLDN15/OBSL1/KRT8/PCDH12/MAGI3/CDHR3/RAP1B/APC/PIK3CA/SAV1/ARHGEF2/CADM1/NFASC/RDX/PIKFYVE/NECTIN3/TMEM204/VCL/KLHL24/LIN7A/KRT18/CGN/DSG1/IGSF11/PKN2/NPHP4/MXRA8/CLIC4/MPDZ/PXN/DLG1/APP/PACSIN2/PTPRK/ITGA5/JCAD/ANKRD23/WASF2/ABCB4/HEPACAM/CLDN3/PTPRU/JAM2/JAG1/AHI1/BVES/COL17A1/ARHGAP24/ANXA2/CLDN11/PCDH9/CYTH3/PDLIM5/CDH10/GJB3/PECAM1/AMOT/PTPRM/AMOTL2/TJAP1/CCN3/PARD6G/ADD1/USP53/NEXN/FMN1/NHS/FXYD1/FGF13/CLDN5/CTNNA1/EPCAM/VSIG10L2/CADM3/DSG2/SGCA/ANK2/DSP/PODXL/CDH12/PLEKHG5/ADGRL3/PKD2/LIMS2/SH3KBP1/AMOTL1/CLMP/CDH19/SLC8A1/LIN7B/CD99L2/CAMSAP3/SDCBP/FRMD4A/VSIG10/DAG1/IQGAP1/PDLIM3/EPPK1/FERMT2/ABI2/RAPGEF2/DLG5/SYNM/FZD4/CDC42BPA/DCHS1/ATP1A2/PARD3B/PARD3/MAGI1/DES/MAGI2/PLPP3/MYADM/TMEM47/PIK3R1/ADD3/CLDN8/DBN1/PDZD2/EPB41L4B/JAM3/PDLIM4/AHNAK/PGM5/KIT/CGNL1/SORBS1/DNMBP/EFNB2/STXBP6/CLDN23/CCND1/CLDN1 |
| GOBP | REGULATION OF TRANSCRIPTION BY RNA POLYMERASE I | 33 | 0.591387464 | 1.922500378 | 0.000408096 | 0.003872303 | 0.002704866 | 4534 | tags=58%, list=22%, signal=45% | MACROH2A1/IPPK/MARS1/ZMPSTE24/SIRT7/PIH1D1/LYAR/POLR2L/BNC1/DHX33/ATF4/MACROH2A2/UTP15/SMARCA4/NOL11/HEATR1/MTOR/PWP1/NCL |
| GOBP | POSITIVE REGULATION OF TRANSCRIPTION BY RNA POLYMERASE I | 23 | 0.646468404 | 1.91874007 | 0.000407138 | 0.003868677 | 0.002702334 | 4534 | tags=61%, list=22%, signal=48% | IPPK/MARS1/PIH1D1/LYAR/BNC1/DHX33/ATF4/UTP15/SMARCA4/NOL11/HEATR1/MTOR/PWP1/NCL |
| GOBP | REGULATION OF CARDIOCYTE DIFFERENTIATION | 26 | -0.613435734 | -1.999614609 | 0.000402925 | 0.00383407 | 0.00267816 | 5282 | tags=65%, list=25%, signal=49% | DLL1/SMAD4/DHX36/KAT2A/TGFB2/BMP2/GPER1/PRICKLE1/BMP4/DKK1/EGFR/FZD7/GREM1/MEF2C/MYOCD/SOX6/EFNB2 |
| GOBP | MUSCLE FILAMENT SLIDING | 39 | -0.521788411 | -1.880511996 | 0.000399985 | 0.003811485 | 0.002662384 | 2095 | tags=33%, list=10%, signal=30% | ACTC1/ACTA1/MYH3/TNNC2/TNNT1/TPM2/TNNC1/VIM/DES/TNNI2/DMD/TPM1/TMOD1 |
| GOBP | RIBONUCLEOSIDE MONOPHOSPHATE METABOLIC PROCESS | 56 | 0.508393602 | 1.846721306 | 0.000398956 | 0.003807086 | 0.002659311 | 3901 | tags=41%, list=19%, signal=33% | UPP1/UCK2/AK2/GART/TJP2/APRT/ATIC/IMPDH1/NT5C2/HPRT1/AMPD3/ADSL/XDH/AK1/ADK/UMPS/GMPS/CASK/RFK/CARD11/CAD/GMPR2/PRPS2 |
| GOBP | ADENYLATE CYCLASE ACTIVATING ADRENERGIC RECEPTOR SIGNALING PATHWAY | 24 | -0.621815421 | -1.969294032 | 0.000398775 | 0.003807086 | 0.002659311 | 2089 | tags=38%, list=10%, signal=34% | ADCY9/ADRB1/AKAP13/ADRA1A/ATP2B4/RAPGEF2/PLN/ADRA2A/ADRB2 |
| GOBP | POSITIVE REGULATION OF ALPHA BETA T CELL DIFFERENTIATION | 46 | 0.522815816 | 1.817413176 | 0.000390272 | 0.003734821 | 0.002608833 | 3986 | tags=54%, list=19%, signal=44% | NFKBIZ/PNP/IL4R/CD83/SASH3/AP3D1/IFNG/SOCS1/CBFB/IL12B/SYK/LGALS9/CCL19/AP3B1/IL23A/LILRB4/CD86/NFKBID/ANXA1/HLA-DRA/IL12RB1/ZAP70/NLRP3/CD80/FOXP3 |
| GOBP | SMALL GTPASE MEDIATED SIGNAL TRANSDUCTION | 491 | -0.255505403 | -1.337416037 | 0.000386749 | 0.003706393 | 0.002588975 | 3722 | tags=28%, list=18%, signal=23% | PLD1/SQSTM1/LPAR4/ALS2/SOS1/ARHGEF2/RALGDS/MADD/RDX/NRP1/PPP2CB/RAP1A/IQSEC1/RELN/GNA12/TIAM2/GNA13/RIPOR1/RABL3/RAB30/SRGAP3/ABL1/SOS2/DENND3/RASA4/SHC3/PDGFRB/KITLG/SHOC2/WASF2/PRKD1/ARHGEF1/GNB1/ARHGAP24/RAB33B/RTN4R/ARHGAP10/RASA3/KSR1/CYTH3/NET1/ERBIN/HACD3/AMOT/NGEF/BRAP/SYDE1/SLIT2/CHML/MRAS/F2R/ZNF304/DOCK5/DOCK4/FBXO8/AUTS2/RHOBTB1/RAPGEF3/RHOQ/MET/ARHGAP20/A2M/GARRE1/ARHGEF6/SHC2/TRIO/RRAS/ARHGAP26/RAPGEFL1/RALGAPB/RHOT1/ARHGAP12/APOE/OGT/ARHGAP35/ITPKB/PIK3CB/RERG/ARHGAP42/ARL3/PLEKHG5/RHOB/STMN3/ARHGEF5/SCAI/NISCH/DOCK7/NTN1/RHOJ/CTNNAL1/STARD13/AKAP13/ROCK2/ARHGAP6/ADRA1A/DOCK1/VAV3/ARHGAP29/ABI2/RAB9B/ARHGEF12/TIMP2/RAPGEF2/MYOC/RALGPS1/SRGAP1/BCL6/RALGAPA1/LPAR1/FGD4/EPS8/ADRA2A/ITSN1/GARNL3/AGTR1/ARFGEF3/CDON/SPRY1/DLC1/ARHGAP31/FAM13A/MCF2L/ARHGEF7/NOTCH2/SPRY2/ARHGEF28/CGNL1/MYO9A/SRGAP2/ARHGEF10/KANK1/IRS2/KANK2/BCAR3/DENND4C/ARHGEF26 |
| GOBP | RIBONUCLEOPROTEIN COMPLEX SUBUNIT ORGANIZATION | 189 | 0.366317013 | 1.580694549 | 0.000386409 | 0.003706393 | 0.002588975 | 2748 | tags=30%, list=13%, signal=26% | RUVBL1/MRPS11/MRPS7/MCTS1/EIF2S2/VCP/RUVBL2/SNRPG/EIF6/EIF3J/SNRPF/SNRPD1/NCBP1/SRSF9/PRMT5/LSM2/NOP2/TXNL4A/DDX28/TARBP2/EIF3I/FASTKD2/CPSF6/MRTO4/SNRPD3/WDR77/CD2BP2/MRPL20/GEMIN6/SNRPC/GEMIN7/DENR/EIF3K/PIH1D1/PPAN/LSM4/XRCC5/BOP1/SNRPB/ADAR/SRPK1/TRAF7/PRKDC/EIF3B/LUC7L2/RPF2/POLR2D/PRPF31/EIF4H/RRP7A/SRSF1/USP39/SHQ1/STRAP/ABT1/ISY1/CLP1 |
| GOCC | SPINDLE MIDZONE | 36 | 0.563931856 | 1.870180547 | 0.000385636 | 0.003706302 | 0.002588912 | 1894 | tags=36%, list=9%, signal=33% | AURKB/KIF18B/PRC1/AURKA/CDC6/KIF14/CDCA8/CENPE/RACGAP1/KIF18A/PLK1/KIF20B/RCC2 |
| GOBP | GLUCOSE 6 PHOSPHATE METABOLIC PROCESS | 24 | 0.650521939 | 1.960176963 | 0.000382836 | 0.003684662 | 0.002573796 | 5106 | tags=71%, list=24%, signal=54% | PGAM1/PGM2/HK2/TIGAR/PGD/TALDO1/GPI/RBKS/SHPK/PGLS/G6PD/DERA/G6PC1/TKT/HK3/RPE/G6PC2 |
| GOBP | BODY MORPHOGENESIS | 44 | -0.515541335 | -1.896186071 | 0.00038141 | 0.003676202 | 0.002567887 | 5323 | tags=61%, list=26%, signal=46% | PTPN11/MAB21L2/ZNF281/EP300/TBX1/MSX1/PHLDB2/TIPARP/MMP2/GNAS/CRISPLD1/CLASP1/NOG/CLDN5/MYH3/RRAS/LRP6/GPC3/PHLDB1/DAG1/SKI/IFT122/RAB3GAP1/PLEKHA1/PDGFRA/CDON/GREM2 |
| GOBP | POSITIVE REGULATION OF CYTOKINE PRODUCTION INVOLVED IN IMMUNE RESPONSE | 50 | 0.522423075 | 1.865012921 | 0.000376027 | 0.003629526 | 0.002535282 | 4079 | tags=50%, list=20%, signal=40% | WNT5A/NOD2/FZD5/CLEC7A/RSAD2/SLC7A5/SASH3/IL1B/SEMA7A/NR4A3/IL6/CD36/FFAR2/LAPTM5/HLA-E/DDX21/XCL1/DDX58/BCL10/B2M/HLA-F/LILRB1/NLRP3/HLA-G/TRAF2 |
| GOBP | AORTA MORPHOGENESIS | 29 | -0.590080925 | -1.969817094 | 0.000375476 | 0.003629419 | 0.002535208 | 4986 | tags=55%, list=24%, signal=42% | TBX1/LRP1/HEY1/NPRL3/TGFB2/EFEMP2/HES1/PDGFRB/JAG1/SOX4/ACVRL1/SEC24B/BMPR1A/RBPJ/TFAP2B/MYLK |
| GOBP | DNA STRAND ELONGATION | 26 | 0.63552157 | 1.947236385 | 0.000372466 | 0.003605511 | 0.002518508 | 3666 | tags=54%, list=18%, signal=44% | GINS3/PCNA/GINS2/MCM4/GINS1/POLE3/POLD2/MCM7/MCM3/DNA2/RFC3/RFC4/POLD3/RAD50 |
| GOBP | TISSUE MIGRATION | 299 | -0.294483747 | -1.459140373 | 0.000372178 | 0.003605511 | 0.002518508 | 3677 | tags=34%, list=18%, signal=29% | PIK3CA/SCARB1/TACSTD2/MAP2K5/TNFSF12/PTEN/FOXC2/FSTL1/NRP1/CYP1B1/PRKCE/PLXND1/IQSEC1/VEGFC/TAC1/FGF1/PKN2/EGR3/PKN1/PXN/SIRT1/ABL1/BMP4/SRPX2/JCAD/SMOC2/AGT/PRKD1/RGCC/BCAS3/TMSB4X/AMOT/CLASP1/PTPRM/STAT5A/EDN1/CLEC14A/MACF1/SLIT2/CCN3/SPRED1/PRKCA/SVBP/DOCK5/MMRN2/MET/ACTC1/EFNA1/DUSP10/PPARG/ACTA1/EGF/SASH1/KRT2/RRAS/ACVRL1/ADGRA2/TESK1/SYNJ2BP/APOE/PTK2/PIK3CB/PFN2/GREM1/MECP2/ZEB2/PLEKHG5/RHOB/KLF4/NR2F2/FGFR1/PTN/MEF2C/AKT3/RHOJ/STARD13/ROCK2/DOCK1/DNAJA4/EPPK1/RTN4/ACTA2/ATOH8/MEOX2/ATP2B4/FBXW7/TGFBR2/SP1/WDPCP/CCBE1/DCN/GATA2/ACTG2/MAP3K3/PLPP3/MCC/EPB41L4B/PATZ1/KIT/EFNB2/KANK1/KANK2/GATA3 |
| GOBP | POSITIVE REGULATION OF BMP SIGNALING PATHWAY | 32 | -0.556242096 | -1.902491403 | 0.000371736 | 0.003605511 | 0.002518508 | 3069 | tags=53%, list=15%, signal=45% | CCN1/SOX11/SMAD2/SMAD4/CDH5/MSX1/BMP4/HES1/ACVRL1/NUMA1/GPC3/NEO1/RBPJ/FOXD1/ELAPOR2/ZNF423/NOTCH2 |
| GOCC | NEURON TO NEURON SYNAPSE | 335 | -0.285771228 | -1.439306787 | 0.000369251 | 0.003589908 | 0.002507609 | 4908 | tags=32%, list=24%, signal=25% | GRIN2D/ARHGEF9/LZTS3/GRIA2/SEMA4C/GRID1/RPS18/PDPK1/ITPR1/SYNPO/PAK2/CAP2/DVL1/PSD3/ARHGAP44/INPP4A/PCLO/GPHN/TANC1/TNIK/PRR12/RAPGEF4/GRIP1/LRRC4C/RNF112/GPER1/DMTN/SOS1/GRIN3A/CHRM1/MIB1/NOS1/SHANK2/SPOCK1/NECTIN3/LIN7A/IQSEC1/EFNB3/ZDHHC2/SYT1/IGSF11/RPS25/MPDZ/ERBB4/DLG1/PCBP2/PRRT2/KCND2/STXBP5/GOPC/PDLIM5/NPTN/RPS27/CLSTN1/NLGN1/SYN2/ADD1/RPL12/ND2/ZDHHC15/DYNLL2/SHANK3/SYNJ2BP/RNF19A/PTPRS/DCLK1/ITGA8/CPEB1/NTRK2/SEPTIN11/EPB41/INSYN1/RPL14/SLC8A1/LIN7B/RPS14/CLSTN2/RPL8/PLCB4/CAMK2N1/CNKSR2/DNM1/RTN4/MAPK1/LRFN5/RPL23/DLG5/PAK3/RPL38/PLEKHA5/PTCH1/TSC1/FYN/FXR1/PJA2/TMEM108/PALM/MAGI2/DLG2/RPL7/ADD3/DBN1/RPL30/LRP4/SRGAP2/CRYAB/MAP1B/EEF2K |
| GOBP | NEGATIVE REGULATION OF INTRINSIC APOPTOTIC SIGNALING PATHWAY | 90 | 0.435390272 | 1.698265142 | 0.000367301 | 0.003576127 | 0.002497982 | 4159 | tags=39%, list=20%, signal=31% | ENO1/PPIF/HDAC1/HIF1A/NDUFA13/MMP9/VNN1/PPIA/DDIAS/NDUFS3/VDAC2/TRAP1/MIF/FIGNL1/BID/BCL2L12/GPX1/SOD2/HYOU1/ARMC10/NOC2L/PLAUR/BDKRB2/TRIAP1/PARK7/TMEM161A/ING2/HTRA2/MDM2/KDM1A/MCL1/CDKN2D/MAPK7/XBP1/EPO |
| GOBP | CELLULAR COMPONENT ASSEMBLY INVOLVED IN MORPHOGENESIS | 96 | -0.396061207 | -1.705035306 | 0.000367231 | 0.003576127 | 0.002497982 | 3516 | tags=39%, list=17%, signal=32% | TNNT3/CASQ1/ABCA2/CD9/PHLDB2/OBSL1/KRT8/CAPN3/PIKFYVE/KRT19/PDGFRB/GPC1/CLASP1/EDN1/ACTC1/TMOD2/ACTA1/MYH3/GNPAT/PHLDB1/DAG1/AKAP13/FHOD3/CFL2/TNNT1/PAFAH1B1/CSRP1/NEBL/PDGFRA/LMOD1/MYH11/TPM1/TMOD1/PGM5/PMP22/MEF2A/TPPP |
| GOBP | REGULATION OF T CELL MEDIATED IMMUNITY | 73 | 0.46756943 | 1.763643172 | 0.000366738 | 0.003576127 | 0.002497982 | 5020 | tags=58%, list=24%, signal=44% | NOD2/FZD5/RSAD2/CYRIB/ARG1/SASH3/IL1B/IL12B/IL7R/IL6/RIPK3/IL23A/FADD/LILRB4/HLA-E/TNFRSF1B/HSPD1/XCL1/KLRD1/CD1B/CLEC4G/HLA-DRA/IL12RB1/CCR2/B2M/HLA-F/WAS/HLA-B/LILRB1/NECTIN2/NLRP3/HLA-G/FOXP3/TRAF2/FBXO38/CD1D/PTPRC/MALT1/AGER/TBX21/IFNA2/HLA-A |
| GOBP | MRNA TRANSPORT | 140 | 0.392823068 | 1.633909813 | 0.000365844 | 0.003576127 | 0.002497982 | 4158 | tags=41%, list=20%, signal=33% | EIF4A3/DDX39A/EIF4E/HNRNPA2B1/NCBP1/SRSF9/NUP37/NUP210/NUP88/MX2/CPSF3/ALYREF/MAGOHB/NUP50/SARNP/NSUN2/SRSF2/NXT1/ZFP36/CETN2/POLR2D/RAE1/NCBP3/MVP/SRSF7/SEC13/SRSF1/THOC5/IWS1/NUP155/IGF2BP3/THOC6/THOC7/NDC1/CPSF4/GLE1/MAGOH/NUP188/DDX19A/WDR33/NUP85/FYTTD1/ARC/CHTOP/NUP205/NUTF2/EIF5A/AGFG1/UPF1/ENY2/NUP58/LRPPRC/NUP107/NUP93/SRSF3/IGF2BP1/POM121 |
| GOBP | POSITIVE REGULATION OF RESPONSE TO CYTOKINE STIMULUS | 52 | 0.518855363 | 1.859648719 | 0.000361414 | 0.003539324 | 0.002472275 | 3342 | tags=40%, list=16%, signal=34% | IRF7/WNT5A/PARP9/CXCR4/CASP4/IFIH1/CASP1/HIF1A/MMP12/PARP14/ADAM17/TLR2/IKBKE/TBK1/NLRC5/FADD/LAPTM5/TSLP/TREM2/DDX58/CPNE1 |
| GOBP | CARDIAC EPITHELIAL TO MESENCHYMAL TRANSITION | 30 | -0.590714671 | -2.002138653 | 0.000356169 | 0.00349306 | 0.002439959 | 4512 | tags=57%, list=22%, signal=44% | SMAD4/HEY1/MSX1/TMEM100/TGFB2/BMP2/JAG1/NOG/EFNA1/TWIST1/WNT2/RTN4/RBPJ/TGFBR2/PDCD4/SPRY1/TGFBR3 |
| GOBP | ATP BIOSYNTHETIC PROCESS | 50 | 0.523795791 | 1.869913419 | 0.000351445 | 0.003451767 | 0.002411115 | 3460 | tags=48%, list=17%, signal=40% | ENO1/ATP5MF/VCP/ATP5MC3/ATP5PF/ATP5F1B/STOML2/ATP5MC1/ATP5PB/ALDOA/SLC25A13/ATP5ME/ATP5MG/ATP5F1C/UQCC3/ATP5PO/ATP6V1A/ANTKMT/TREM2/PKM/ATP5F1D/PARP1/ATP5F1A/ATP5PD |
| GOBP | INTERLEUKIN 2 PRODUCTION | 59 | 0.493858196 | 1.803914027 | 0.000349024 | 0.003433004 | 0.002398009 | 4438 | tags=54%, list=21%, signal=43% | NOD2/PNP/CLEC7A/PRKCQ/CD83/STOML2/GBP1/SASH3/IL1B/GLMN/ZFP36/LAG3/LILRB4/LAPTM5/HOMER3/CD86/SFTPD/CARD9/XCL1/CD3E/ANXA1/CCR2/CARD11/CD80/FOXP3/IL17F/NAV3/TRAF2/CR1/PTPRC/CD28/MALT1 |
| GOBP | ESTABLISHMENT OF PROTEIN LOCALIZATION TO CHROMOSOME | 27 | 0.622253608 | 1.933553357 | 0.000347534 | 0.003423366 | 0.002391276 | 4190 | tags=67%, list=20%, signal=53% | CCT5/MACROH2A1/RUVBL2/CCT2/CCT3/CCT7/PIH1D1/BRCA2/LRWD1/TCP1/MACROH2A2/DKC1/NIPBL/CCT6A/CCT8/NABP2/ATR/WRAP53 |
| GOBP | ANTIGEN PROCESSING AND PRESENTATION OF PEPTIDE OR POLYSACCHARIDE ANTIGEN VIA MHC CLASS II | 99 | 0.42519097 | 1.689718624 | 0.000347447 | 0.003423366 | 0.002391276 | 5007 | tags=45%, list=24%, signal=35% | AP2S1/KIF2C/PYCARD/KIF4A/KIF2A/KIF11/IFI30/CENPE/KIF23/CTSL/RACGAP1/AP1S3/KIF15/KIF18A/ACTR1A/AP1M2/CAPZA1/DYNLL1/SEC24C/CTSD/LAG3/HLA-DMB/CTSS/HLA-DOB/SEC13/KIF22/DCTN5/DCTN6/CANX/AP1B1/TREM2/CLTA/HLA-DRA/LGMN/MARCHF1/DCTN2/KIFAP3/AP2M1/DNM2/HLA-DOA/DCTN3/FCER1G/HLA-DMA/HLA-DQB1/SEC24A |
| GOBP | POSITIVE REGULATION OF CD4 POSITIVE ALPHA BETA T CELL DIFFERENTIATION | 30 | 0.607924283 | 1.918025792 | 0.000343694 | 0.003395496 | 0.002371809 | 4438 | tags=63%, list=21%, signal=50% | NFKBIZ/IL4R/CD83/SASH3/IFNG/SOCS1/IL12B/LGALS9/CCL19/IL23A/CD86/NFKBID/ANXA1/HLA-DRA/IL12RB1/NLRP3/CD80/FOXP3/MALT1 |
| GOBP | BIOLOGICAL PROCESS INVOLVED IN INTERACTION WITH SYMBIONT | 84 | 0.449953649 | 1.738187016 | 0.000339577 | 0.003359757 | 0.002346845 | 3131 | tags=35%, list=15%, signal=29% | KRT6A/ZC3H12A/IFI27/LTF/APOL1/HDAC1/PHB/VAPA/CCL4/TUSC2/ARG1/PSMC3/GAPDH/ROMO1/HSPA8/GPX1/CCL8/CXCL6/CAMP/SUGT1/TARDBP/HSPD1/SFTPD/CCL5/LEF1/CFL1/SMARCA4/TAF11/PPIB |
| GOBP | NUCLEAR TRANSCRIBED MRNA CATABOLIC PROCESS | 203 | -0.321621467 | -1.517786609 | 0.000335764 | 0.003326933 | 0.002323916 | 3808 | tags=36%, list=18%, signal=30% | UPF2/RPL24/RPS11/RPS5/RPS9/RPL41/SMG6/RPL23A/SMG1/RPS25/RPS15A/TTC37/RPS15/CNOT8/CSDE1/UPF3A/RPL13A/TENT4A/RPL32/RPL7A/DCP1B/RPS27/RPL31/RPL18/EIF4ENIF1/RPL27/RPS8/RPL4/RPL12/RPL9/XRN1/PAN2/EXOSC7/TNRC6B/RPS6/PAN3/RPL13/RPL10A/RPL11/RPL37/ZFP36L1/SAMD4B/TNRC6C/RPL14/RPS4X/RPLP2/RPS14/TUT4/RPLP1/RPL8/GSPT2/RPS17/RPS24/RPL22/RPS23/EIF3E/RBM8A/RPL23/RPL38/CTIF/RPS20/RPL3/SAMD4A/RPL15/RPL34/NBAS/RPS3A/CASC3/RPL5/RPL10/RPL7/ZFP36L2/RPL35A/RPL30 |
| GOBP | RNA MODIFICATION | 161 | 0.380971422 | 1.62222516 | 0.000334937 | 0.003323647 | 0.002321621 | 4577 | tags=40%, list=22%, signal=31% | NHP2/APOBEC3B/WDR4/DIMT1/NOP10/MTO1/NOP2/FTSJ1/TRUB2/PUSL1/METTL6/METTL1/HENMT1/HSD17B10/TRMT10C/TFB2M/ELP6/PUS10/DUS1L/TPRKB/RBM47/MOCS3/TYW5/EMG1/NSUN2/PUS1/CMTR2/ADAR/GAR1/TRMT6/TRMT112/JMJD6/METTL8/TRMU/CTU2/PUS7/DKC1/MTFMT/NAT10/OSGEP/RPUSD1/DUS2/MRM2/TRMT1/LCMT2/METTL4/TRUB1/DUS3L/MRM3/THUMPD3/TYW3/FDXACB1/THG1L/ALKBH3/BUD23/TSR3/QTRT2/ADAT3/ELP5/RPUSD2/TRMT5/PUS3/RPUSD3/METTL5 |
| GOBP | CARDIAC MUSCLE CELL PROLIFERATION | 46 | -0.490943165 | -1.833199754 | 0.000332661 | 0.00330594 | 0.002309252 | 4040 | tags=50%, list=19%, signal=40% | TGFB2/KCNK2/SAV1/PTEN/FOXC2/VGLL4/ERBB4/JARID2/YAP1/NOG/FOXC1/FGFR2/BMPR1A/DIPK2A/MEF2C/RBP4/WNT2/RBPJ/MAPK1/TGFBR2/ZFPM2/MEIS1/TGFBR3 |
| GOMF | SULFUR COMPOUND BINDING | 245 | -0.313363944 | -1.517306123 | 0.00032896 | 0.003274 | 0.002286942 | 3955 | tags=33%, list=19%, signal=27% | SOAT1/LIPC/METTL17/LTBP2/MSTN/GSTM3/EFEMP2/CHST15/FSTL1/NRP1/ADAMTS5/BMT2/LGR6/APLP2/HMGCL/FGF1/APP/BMP4/CCN2/SMOC2/SULT1A1/ZCCHC4/TENM1/CTSG/ENPP1/THBS2/RSPO3/THBS3/ACBD4/RTN4R/VEGFB/FGFBP3/MGST1/SLIT2/CCN3/ECI2/LXN/PGF/SOD3/FBLN7/METTL3/FGF9/PCOLCE2/SERPINA5/SLIT3/APOE/ALDH6A1/PTPRS/FGFR2/DBT/LPL/TNXB/FN1/PNPLA3/FGFR1/PTPRF/PTN/ECM2/HACL1/PRELP/CCN5/PANK3/RPL22/CD34/CFH/RSPO1/NDNF/LAMC2/BMP7/PTCH1/PAFAH1B1/RTN4RL1/GAL3ST4/LANCL1/DPYSL3/FST/GPNMB/POSTN/GREM2/TGFBR3/ACADL/ANG |
| GOBP | CELL CHEMOTAXIS | 274 | 0.340636958 | 1.526813117 | 0.000328358 | 0.003272866 | 0.00228615 | 4443 | tags=35%, list=21%, signal=28% | S100A12/S100A9/CXCR2/C10orf99/WNT5A/NOD2/S100A8/EPHA2/TPBG/CCL20/CXCR4/C1QBP/S100A7/PRKCQ/ABCC1/CCR7/CXCL1/CXCL13/CXCL2/CYP7B1/GSTP1/CXCL16/HBEGF/PPIA/CXCL10/CXCR6/CXCL8/CCL18/CCL4/CCL22/ADAM17/CXCL9/S100A14/CH25H/MIF/CCL2/LYN/CORO1A/SLAMF8/DEFB124/CCL8/SYK/RAC2/IL6/LGALS9/CCL7/CCL19/CCR5/ITGB2/CXCL17/IL23A/CXCL3/GPR183/FFAR2/OXSR1/CXCL6/ADAM10/ACKR2/CXCL11/ADAM8/PIK3CD/RHOG/HMGB2/SFTPD/BIN2/CCL5/LEF1/XCL1/CCR1/PPIB/SRP54/ANXA1/ADGRE2/CCRL2/PRKCD/CCR2/NUP85/CCL17/LGMN/TMEM102/P2RX4/JAML/SERPINE1/VAV1/IL10/GPSM3/CKLF/VCAM1/CSF3R/C5AR2/HRH1/AKIRIN1/C3AR1/CXCR1/CXCR3 |
| GOMF | EXONUCLEASE ACTIVITY | 82 | 0.450313208 | 1.731234103 | 0.000321517 | 0.003209438 | 0.002241845 | 4366 | tags=43%, list=21%, signal=34% | ZC3H12A/TREX2/ISG20/EXOSC4/TDP1/FEN1/PNPT1/AEN/EXOSC3/EXO1/ISG20L2/APEX2/CPSF3/DIS3/PDE12/POLQ/CNOT1/EXOSC2/ERI1/ERI2/USB1/EXOSC5/APTX/XRN2/NOCT/CNOT6/TOE1/REXO4/RAD50/DCPS/PLD4/DXO/EXOG/POLD1/ANGEL1 |
| GOBP | ESTABLISHMENT OF PROTEIN LOCALIZATION TO MITOCHONDRIAL MEMBRANE | 52 | 0.521744688 | 1.870004457 | 0.00031948 | 0.003193854 | 0.002230958 | 4058 | tags=46%, list=19%, signal=37% | TFDP1/GZMB/HSPA4/TIMM8B/TOMM22/NMT1/NDUFA13/YWHAQ/TIMM13/SFN/TIMM10/MAIP1/BID/ROMO1/PDCD5/AP3B1/CASP8/TP63/YWHAZ/BAX/AGK/MAPK8/BCS1L/SAMM50 |
| GOBP | REGULATION OF DEVELOPMENTAL GROWTH | 294 | -0.298832946 | -1.484309965 | 0.000318803 | 0.003191832 | 0.002229546 | 3748 | tags=30%, list=18%, signal=25% | MSTN/WWC3/PIK3CA/PRKN/SAV1/SOCS2/MBD5/SEMA6D/PTEN/FOXC2/CAPN3/VGLL4/NEDD4L/NRP1/FGFR3/RAI1/SYT1/ERBB4/MFSD2A/APP/ABL1/SIN3A/GPAM/BMP4/JARID2/CXCL12/RTN4R/RGS2/FTO/SEMA3E/GOLGA4/YAP1/INSR/STAT5A/LEP/DIO3/EDN1/NKD1/MACF1/L1CAM/STK3/NOG/PLCB1/SPART/SEMA4G/SEMA6A/ADRB1/DUSP10/FGF13/LATS2/SEMA3B/RYK/APOE/PTPRS/FOXC1/FGFR2/GHR/FN1/BMPR1A/GSK3B/PPARA/MEF2C/RBP4/MAP2/SYT17/NTN1/CDKN1B/WNT2/NPY1R/EPPK1/RTN4/RBPJ/MAPK1/TGFBR2/DPYSL2/ZFPM2/PTCH1/SEMA3G/STAT5B/DBN1/BBS2/AR/BCL2/MEIS1/SOD1/TGFBR3/MAP1B/ADRB2 |
| GOBP | ATP DEPENDENT CHROMATIN REMODELING | 83 | 0.456415586 | 1.758570327 | 0.000318078 | 0.00318933 | 0.002227799 | 5247 | tags=48%, list=25%, signal=36% | RUVBL1/PSME4/CENPN/HDAC1/KNL1/CENPW/CENPX/HNRNPC/OIP5/HJURP/CENPM/CENPK/CENPA/CENPS/NASP/NPM1/CENPH/ACTR8/CENPI/HDAC2/MBD2/ZNHIT1/MIS18A/SMARCA4/SMARCA5/RNF8/SPTY2D1/ACTL6A/CENPL/CENPO/CENPU/INO80B/H4C4/H4C9/MBD3/RBBP4/H4C2/SRCAP/ERCC6/CENPQ |
| GOBP | NUCLEOSIDE MONOPHOSPHATE BIOSYNTHETIC PROCESS | 41 | 0.563735313 | 1.918925438 | 0.000315312 | 0.003166317 | 0.002211724 | 3901 | tags=44%, list=19%, signal=36% | UPP1/TK1/UCK2/TYMS/GART/APRT/ATIC/IMPDH1/HPRT1/AMPD3/ADSL/ADK/UMPS/GMPS/DCTD/RFK/CAD/PRPS2 |
| GOBP | REGULATION OF RELEASE OF CYTOCHROME C FROM MITOCHONDRIA | 43 | 0.552216125 | 1.894317251 | 0.000313719 | 0.003155038 | 0.002203845 | 3321 | tags=47%, list=16%, signal=39% | PPIF/PRELID1/GHITM/FAM162A/PYCARD/BAK1/BIK/MMP9/TNFSF10/BID/PDCD5/GPX1/PLAUR/BNIP3/TRIAP1/MFF/BAX/FXN/PSMD10/CIDEB |
| GOBP | CELLULAR RESPONSE TO INTERFERON BETA | 21 | 0.670088216 | 1.952926553 | 0.000313243 | 0.003154976 | 0.002203802 | 3979 | tags=43%, list=19%, signal=35% | STAT1/IFI16/NDUFA13/IRF1/PNPT1/AIM2/HTRA2/MNDA/CDC34 |
| GOBP | MESENCHYME MORPHOGENESIS | 48 | -0.5047489 | -1.89689022 | 0.000311555 | 0.003142684 | 0.002195215 | 4675 | tags=50%, list=22%, signal=39% | WNT11/HEY1/MSX1/TMEM100/TGFB2/BMP2/FOXC2/ROBO2/NOG/ACTC1/TWIST1/ACTA1/ACVRL1/FOXC1/BMPR1A/SMAD3/ACTA2/RBPJ/MDM4/TGFBR2/BMP7/OSR1/DCHS1/ACTG2 |
| GOBP | HINDBRAIN DEVELOPMENT | 137 | -0.369650398 | -1.673098119 | 0.000311067 | 0.003142478 | 0.002195072 | 3432 | tags=29%, list=16%, signal=25% | PTBP2/MAFB/CNTN1/ABL1/HES1/ND4/CBLN1/COX1/AHI1/ZNF365/NOG/EN1/HOXB2/EGF/GNPAT/LRP6/PTPRS/FOXC1/MECP2/SEC24B/PTN/PPARGC1A/ALDH1A2/HERC1/TTBK2/RBFOX2/BMP7/HOXB3/RERE/GATA2/FZD4/CKB/PLXNA2/LPAR1/SPTBN2/ZNF423/DLC1/GLI2/BCL2/RORA |
| GOBP | REGULATION OF HISTONE UBIQUITINATION | 12 | 0.776058846 | 1.967245869 | 0.000308932 | 0.003125608 | 0.002183288 | 3652 | tags=67%, list=18%, signal=55% | UBE2N/TRIP12/OTUB2/PARK7/CDK9/CTR9/KDM1A/OTUB1 |
| GOBP | CGMP METABOLIC PROCESS | 17 | -0.689659088 | -1.998949962 | 0.000307781 | 0.003118662 | 0.002178436 | 4263 | tags=53%, list=20%, signal=42% | GUCY1B1/PDE1A/NPR1/PDE5A/PDE9A/NPR2/GUCY1A2/PDE2A/RORA |
| GOBP | CARDIAC CELL DEVELOPMENT | 71 | -0.437016338 | -1.765065218 | 0.000307531 | 0.003118662 | 0.002178436 | 3069 | tags=34%, list=15%, signal=29% | BMP4/PDGFRB/JAG1/TBX3/BVES/RGS2/PDLIM5/SHOX2/EDN1/ACTC1/SORBS2/PPARA/SLC8A1/AKAP13/ADRA1A/FHOD3/PDCD4/SGCB/NEBL/PDGFRA/SPRY1/MYH11/MEF2A/TGFBR3 |
| GOBP | PHAGOCYTOSIS | 275 | 0.341442863 | 1.530889482 | 0.000306283 | 0.00311287 | 0.00217439 | 5595 | tags=43%, list=27%, signal=32% | NOD2/CLEC7A/MYD88/PYCARD/LDLR/PLSCR1/RAB27A/ARPC1A/TLR2/ARPC3/TUSC2/PLCG2/ARPC1B/IRF8/RHOH/LMAN2/IFNG/ACTR3/CLCN3/MESD/RAB31/IL1B/RAB5A/CCL2/ATG5/LYN/CSK/FCN1/CORO1A/CD247/HCK/SYK/ARPC2/RAC2/LYAR/CD36/ITGB2/ARHGAP25/BRK1/CD3G/IL2RG/ATG3/PLD2/NCF2/JMJD6/ITGAL/C2/SLAMF1/WASL/VAMP7/RHOG/ARPC5/PIK3R2/SIRPG/CYFIP1/CYBA/ABI1/SFTPD/BIN2/TNF/TREM2/MYH9/SNX3/ICAM3/ANXA1/BAIAP2/C1orf43/P2RY6/TYROBP/PRKCD/ELMO3/YES1/IL15/BECN1/WAS/DOCK2/UNC13D/IGHV7-81/SPHK1/CD300A/VAV1/PLD4/PTX3/IL15RA/FGR/TRDC/CYFIP2/CD300LF/MYO1G/ABCA7/VAV2/PTPRC/MYO7A/FCN3/DNM2/AIF1/CEACAM4/CORO1C/FCER1G/MAPK3/CRP/MYO10/GRB2/IL2RB/CLN3/TM9SF4/SYT7/FPR2/RUBCN/PRTN3/C4BPA/SRPX/AZU1/CEBPE/SH3BP1/NCF4/ICAM5/LIMK1/CALR |
| GOBP | NEGATIVE REGULATION OF SMOOTH MUSCLE CONTRACTION | 13 | -0.755757502 | -2.058028208 | 0.000303818 | 0.003092503 | 0.002160163 | 2662 | tags=62%, list=13%, signal=54% | RGS2/DOCK5/DOCK4/ARHGAP42/KCNMA1/PRKG1/SOD1/ADRB2 |
| GOBP | SMOOTH MUSCLE CELL DIFFERENTIATION | 56 | -0.48631636 | -1.881852661 | 0.000302915 | 0.003087984 | 0.002157006 | 3985 | tags=46%, list=19%, signal=38% | PDGFB/HEY1/ANKRD17/RAMP2/GPER1/EFEMP2/TMEM204/SIRT1/BMP4/HES1/TBX3/FGF9/NFATC2/FGFR2/MECP2/MEF2C/MYOCD/RBPMS2/MRTFB/PDCD4/SGCB/NFATC1/PRDM6/EPC1/PIAS1/KIT |
| GOCC | REPLICATION FORK | 64 | 0.488350182 | 1.810096341 | 0.00029926 | 0.003055365 | 0.002134222 | 4287 | tags=44%, list=21%, signal=35% | UHRF1/H2AX/TIMELESS/PCNA/MCM10/RPA3/DONSON/POLD2/RFC2/MCM3/BLM/RFC3/WDHD1/PIF1/POLA2/HMCES/PRIM1/RAD18/PLRG1/POLD4/SMARCA5/RFC4/TEX264/POLD3/RPA1/TIPIN/RAD51C/POLD1 |
| GOBP | POSITIVE REGULATION OF BONE MINERALIZATION | 39 | -0.527902327 | -1.902546395 | 0.00029925 | 0.003055365 | 0.002134222 | 5450 | tags=67%, list=26%, signal=49% | ACVR2A/NELL1/KL/PKDCC/TMEM119/ACVR2B/BMP2/BMP6/ATRAID/BMP4/ADGRV1/TFAP2A/ATP2B1/ANO6/P2RX7/GPM6B/BMPR1A/PTN/MEF2C/SLC8A1/BMPR1B/SMAD3/BMP7/OSR1/OSR2/ADRB2 |
| GOBP | RESPIRATORY CHAIN COMPLEX IV ASSEMBLY | 26 | 0.639812391 | 1.960383451 | 0.000295272 | 0.003023833 | 0.002112196 | 3899 | tags=58%, list=19%, signal=47% | SCO2/TACO1/COA4/COA1/COA6/SCO1/COA3/COX10/PET100/COX14/COX17/TIMM21/COX16/FASTKD3/BCS1L |
| GOBP | SYNAPSE ASSEMBLY | 166 | -0.347251872 | -1.614349621 | 0.000291835 | 0.002993206 | 0.002090803 | 4366 | tags=36%, list=21%, signal=29% | DVL1/NRXN1/PCDHB13/PCLO/FLRT2/DNER/OBSL1/AMIGO1/PCDHB11/LINGO2/GABRA2/PTEN/GABRB3/SHANK2/PLXND1/SLITRK2/ERBB4/APP/SRPX2/ROBO2/CBLN1/SETD5/FZD1/PDLIM5/PCDHB14/NPTN/SLITRK4/CLSTN1/PCDHB2/BHLHB9/LRRN1/PCDHB10/NLGN1/PCDHB4/FARP1/PCDHB5/FGF13/GNPAT/SHANK3/RYK/GPC4/PTPRS/MECP2/GPC6/NTRK2/ADGRL3/MEF2C/SNCA/NTN1/CLSTN2/LRFN5/DLG5/EPHB1/SPTBN2/PCDHB16/SLITRK6/LRP4/EFNB2/MAP1B/EEF2K |
| GOBP | DNA TEMPLATED TRANSCRIPTION ELONGATION | 112 | 0.415042752 | 1.683975354 | 0.000287474 | 0.002952986 | 0.002062709 | 4252 | tags=40%, list=20%, signal=32% | TSFM/POLR2H/EZH2/ELL2/NCBP1/POLR2F/ELOC/ELOB/NELFCD/ALYREF/POLR1H/ZNF326/POLR2L/ELOF1/POLR1C/TAF1D/LEO1/RNF168/ADRM1/POLR2G/HMGN1/POLR2D/POLR1B/ELL/EAF1/THOC5/CDK7/IWS1/PAF1/POLR2E/POLR1G/CCNH/RNF8/NELFE/TEFM/CDK9/CTR9/POLR2I/SSRP1/SUPT4H1/ENY2/POLR1A/CCNK/TCERG1/POLR2J |
| GOBP | NEGATIVE REGULATION OF IMMUNE EFFECTOR PROCESS | 123 | 0.414822152 | 1.711520268 | 0.000285341 | 0.002935562 | 0.002050538 | 5020 | tags=46%, list=24%, signal=35% | SERPINB4/ZC3H12A/NOD2/C1QBP/IL4R/NLRX1/SUSD4/UFD1/TARBP2/ARG1/JAK3/HMOX1/SLAMF8/SERPINB9/DHX58/IL7R/LGALS9/ITCH/GRN/PRKDC/IL2RA/PGLYRP2/LILRB4/HLA-E/BST2/TNF/MICB/XCL1/KLRD1/PGLYRP3/CLEC4G/ANXA1/IRAK3/CD96/CCR2/HLA-F/NPLOC4/HLA-B/LILRB1/HLA-G/CD300A/IL10/RNF26/FOXP3/ILRUN/PARP3/CR1/PTPRC/IL33/FOXF1/TBX21/ARRB2/IFNA2/GPATCH3/MUL1/HLA-A |
| GOBP | TRNA 5 END PROCESSING | 15 | 0.730161752 | 1.97496968 | 0.000276134 | 0.0028452 | 0.001987418 | 4057 | tags=73%, list=19%, signal=59% | HSD17B10/TRMT10C/RPP40/POP5/RPP25L/POP4/POP1/POP7/THG1L/RPP30/RPP38 |
| GOBP | SMOOTHENED SIGNALING PATHWAY | 130 | -0.372085226 | -1.665274413 | 0.000275985 | 0.0028452 | 0.001987418 | 3867 | tags=45%, list=19%, signal=37% | GLI1/MGRN1/INTU/RAB34/SFRP1/PDCL/IFT81/HIPK1/IFT140/FBXL17/TXNDC15/FOXA1/KCTD6/TBC1D32/PRKACB/BBS7/IFT27/C2CD3/PRRX1/BTRC/IFT172/IFT52/SEPTIN2/TCTN1/PTCHD1/HES1/ENPP1/DZIP1L/DYNC2H1/SHOX2/DZIP1/EVC/CTNNA1/CIBAR1/GLIS2/EVC2/TULP3/CREBBP/RO60/FGFR2/ARL3/ROR2/GPC3/MOSMO/TTBK2/IFT122/TGFBR2/WDPCP/DLG5/PTCH1/WDR19/GAS1/ZNF423/CDON/GLI2/GLI3/BOC/RORA |
| GOBP | PROTEIN LOCALIZATION TO CHROMOSOME CENTROMERIC REGION | 25 | 0.645813023 | 1.945490149 | 0.000263854 | 0.002727035 | 0.001904878 | 3940 | tags=60%, list=19%, signal=49% | CDK1/NDC80/BUB1B/AURKB/TTK/KNL1/ZWILCH/CENPA/CDT1/RCC2/HASPIN/RB1/MIS12/SPDL1/BUB3 |
| GOBP | PRODUCTION OF MOLECULAR MEDIATOR INVOLVED IN INFLAMMATORY RESPONSE | 67 | 0.493902264 | 1.835909756 | 0.000263616 | 0.002727035 | 0.001904878 | 4090 | tags=43%, list=20%, signal=35% | ZC3H12A/NOD2/CLEC7A/IL4R/MYD88/PYCARD/PLA2G3/HIF1A/GBP5/STAT3/NOS2/LYN/CD6/SLAMF8/SYK/IL6/CD36/GRN/VAMP8/LILRB4/VAMP7/TNF/NLRC3/NLRP7/SERPINE1/PLD4/MAPK14/IL17F/GPSM3 |
| GOBP | SKIN DEVELOPMENT | 323 | 0.334322104 | 1.52203723 | 0.000260173 | 0.00269729 | 0.001884101 | 3354 | tags=26%, list=16%, signal=22% | KRT16/DSC2/PI3/KRT6A/HPSE/KLK13/DSG3/SERPINB13/LCE3D/TGM1/SPRR1B/CDH3/WNT5A/EPHA2/ZDHHC21/SPRR3/KRT6B/CERS3/CNFN/S100A7/SPRR1A/IVL/HDAC1/MACROH2A1/GRHL3/ABCA12/PRKCH/TGM3/KRT17/TMEM79/FERMT1/ST14/CSTA/TRIM16/ZBED2/IL20/ALOX12B/SLC27A4/SPRR2G/BCR/ABCB6/SFN/CTSL/ZMPSTE24/LTB/CYP27B1/STMN1/CBFB/CAPN1/PRSS8/KLK12/AP3B1/NSUN2/PCSK6/TRPC4AP/OVOL2/KRT24/OVOL1/ZFP36/EREG/ETV4/KRT78/PKP3/WNT10A/GRHL1/TP63/AQP3/HOXC13/KRT37/FURIN/NSDHL/MACROH2A2/PLAAT4/HDAC2/TNF/ADAM9/RPTN/COMP/UGCG/ANXA1/TRADD/CASP3/KRT14/DKK4/OPN3 |
| GOBP | MRNA PROCESSING | 488 | 0.303323603 | 1.425844171 | 0.000258931 | 0.002688555 | 0.001877999 | 5066 | tags=35%, list=24%, signal=27% | CCNB1/EIF4A3/DDX39A/C1QBP/ESRP2/PPIL1/SNRPG/POLR2H/SNRPF/SNRPD1/HNRNPA2B1/ZPR1/AURKAIP1/NCBP1/SRSF9/POLR2F/PRMT5/HNRNPC/PNPT1/DDX41/LSM2/BUD31/PPP4R2/TXNL4A/CPSF6/SF3B3/TRUB2/LSM7/SNRPD3/FASTKD5/EFTUD2/WDR77/SNRNP25/SF3B5/LSM1/CD2BP2/SNU13/UBL5/CPSF3/PPIH/LSM10/PSPC1/ALYREF/SNRPA1/GEMIN6/MAGOHB/CSTF2/SNRPC/TBRG4/GEMIN7/HSPA8/PDE12/SF3B6/ZNF326/LSM5/LSM4/POLR2L/SARNP/LEO1/SNRPB/SRSF2/CMTR2/AQR/ADAR/SRPK1/POLR2G/DAZAP1/LUC7L2/ELAVL1/POLR2D/PRPF31/JMJD6/NCBP3/PRPF38A/SRSF7/SRSF1/GRSF1/TSEN15/TSEN54/USP39/TARDBP/PDCD11/STRAP/HNRNPF/THOC5/LSM6/RBM28/CDK7/IWS1/HNRNPM/ISY1/CLP1/PAF1/PRPF4/TSEN34/POLR2E/THOC6/PLRG1/ECD/THOC7/TTF2/PTBP1/CCNH/YBX1/PRPF4B/PRDX6/PQBP1/GEMIN2/SNRPA/NELFE/CPSF4/XRN2/PNN/MBNL3/SSU72/CSTF1/MAGOH/CDK9/SREK1IP1/WDR33/CTR9/POLR2I/PRCC/SNRNP40/RBBP6/CHTOP/KDM1A/ESRP1/CSTF3/TFIP11/SUPT4H1/TRA2B/DCPS/PRPF38B/RPRD1B/SRSF3/CWF19L1/SMNDC1/TCERG1/POLR2J/TXNL4B/PRPF18/TSEN2/CWC25/DHX15/RBM7/HNRNPK/PTCD2/RPUSD3/SRSF8/NCL/PAPOLA/CPEB3/PRPF40A/ZBTB7A/LSM3/UPF3B/SNRPD2/DDX46/RAMAC/YJU2/WDR83/PUF60/SRSF10/FUS/PRPF19/GEMIN8/CCAR1/SYNCRIP/SAP18/RNGTT/GTF2H1/LOC107984784 |
| GOBP | INTERFERON BETA PRODUCTION | 51 | 0.526425669 | 1.885817702 | 0.000256238 | 0.00266472 | 0.00186135 | 3060 | tags=37%, list=15%, signal=32% | IRF7/NMI/POLR3G/NLRX1/PYCARD/IFIH1/IRF1/TLR2/RELB/TBK1/DHX58/POLR3B/TRAF3/REL/HMGB2/POLR3D/TLR8/NLRC3/DDX58 |
| GOBP | MITOCHONDRIAL CYTOCHROME C OXIDASE ASSEMBLY | 22 | 0.651019649 | 1.927161165 | 0.000254546 | 0.00265123 | 0.001851927 | 3899 | tags=59%, list=19%, signal=48% | SCO2/TACO1/COA4/COA1/SCO1/COA3/PET100/COX14/COX17/TIMM21/COX16/FASTKD3/BCS1L |
| GOCC | PROTON TRANSPORTING ATP SYNTHASE COMPLEX COUPLING FACTOR F O | 12 | 0.779712111 | 1.976506597 | 0.000252396 | 0.002632915 | 0.001839134 | 2184 | tags=67%, list=10%, signal=60% | ATP5MF/ATP5MC3/ATP5PF/ATP5MC1/ATP5PB/ATP5ME/ATP5MG/ATP5PO |
| GOBP | DENDRITE DEVELOPMENT | 230 | -0.318500665 | -1.530947291 | 0.000250673 | 0.00261901 | 0.001829421 | 4590 | tags=34%, list=22%, signal=27% | DAB2IP/CUX2/PAK2/HDAC6/SEMA3A/DVL1/ARHGAP44/TRPC6/OBSL1/TNIK/PHACTR1/GRIP1/PREX2/SS18L1/CAMSAP2/GRIN3A/UBE3A/PTEN/NEDD4L/NRP1/SHANK2/IQSEC1/RELN/MFSD2A/ANKRD27/APP/SARM1/HECW2/CAMK1D/LRRK2/PDLIM5/ZNF365/NGEF/BHLHB9/NLGN1/MAP6/EFNA1/FARP1/ZDHHC15/DIP2A/FOXO6/SHANK3/APOE/TMEM106B/PTPRS/DCLK1/MECP2/CAPRIN2/TRAK2/GSK3B/PTN/MEF2C/MAP2/NTN1/IQGAP1/PRKG1/RBFOX2/ABI2/KIDINS220/LAMC2/BMP7/RERE/RAPGEF2/DLG5/PAK3/MAP1A/EPHB1/LPAR1/FYN/ITSN1/SDC2/DBN1/LRP4/FBXO31/SRGAP2/MEF2A/MAP1B/EEF2K/COBL |
| GOBP | REPRODUCTIVE SYSTEM DEVELOPMENT | 395 | -0.282387819 | -1.447731461 | 0.00024532 | 0.00256707 | 0.00179314 | 3630 | tags=29%, list=17%, signal=24% | STOX2/VASH2/UBE3A/ARID4A/PTEN/LGR4/ZNF830/C3/FSTL3/SETD2/DLG1/SIRT1/KRT19/FEM1B/BMP4/WNT7B/ROBO2/HES1/PDGFRB/KITLG/ATRX/CSDE1/TESC/MERTK/TBX3/RSPO3/GJB3/LEP/SPATA2/MGST1/GNRH1/SLIT2/STK3/NOG/ETNK2/LHCGR/SOX8/BPTF/SP3/WDR48/ESR1/FGF9/PPARG/SERPINA5/CTNNA1/PLAG1/LIF/KDM5A/PGR/RPS6/GGNBP2/SLIT3/AKR1C3/PTK2/LRP6/FOXC1/FGFR2/TPPP3/ZNF568/ZFP36L1/NR2F2/GDF7/PKD2/ZFX/ROR2/PTN/RBP4/HOXA11/MYOCD/WNT2/PSAPL1/BMPR1B/ARNT/IRX5/RBPJ/MAPK1/BMP7/STAR/DCN/ZFPM2/GATA2/OSR1/FZD4/PLEKHA5/PTCH1/HOXA10/PKD1/IMMP2L/WDR19/STAT5B/PLEKHA1/NUPR1/AGO4/AXL/PDGFRA/ASB1/INHBB/GLI2/FST/RNF38/AR/NOTCH2/GLI3/PATZ1/KIT/BCL2/SOD1/RHOBTB3/WNT2B/CRIP1/CCND1/ANG/GATA3 |
| GOMF | CYTOKINE ACTIVITY | 219 | 0.363632188 | 1.59503062 | 0.000245315 | 0.00256707 | 0.00179314 | 4636 | tags=33%, list=22%, signal=26% | NAMPT/IL36G/C10orf99/WNT5A/CCL20/IL36RN/CXCL1/CXCL13/CXCL2/IL36A/IL19/AREG/CXCL16/TNFSF10/IL20/CXCL10/CXCL8/CCL18/CCL4/CCL22/CXCL9/AIMP1/LTB/IFNG/IL26/MIF/IL1B/FAM3D/CCL2/IL1RN/GPI/IL12B/CCL8/IL6/CCL7/CCL19/IL17C/IL24/IL23A/CXCL3/GRN/CXCL6/IL32/CXCL11/WNT10A/TNFSF9/TSLP/CCL5/TNF/EBI3/IL36B/FASLG/XCL1/TNFSF15/SECTM1/IL22/CCL17/IL15/TNFSF13B/SPP1/IL10/IL17F/EPO/CKLF/TNFRSF11B/TNFSF13/INHBC/TNFSF14/IL33/WNT7A/CXCL5/SLURP1/INHA |
| GOBP | ACUTE PHASE RESPONSE | 43 | 0.560324796 | 1.922133165 | 0.000242013 | 0.002540379 | 0.001774495 | 5680 | tags=56%, list=27%, signal=41% | SERPINA1/PLSCR1/APOL2/CEBPB/SERPINA3/IL1B/IL6/PTGER3/TNF/SAA4/IL22/PTGES/HP/EPO/SERPINF2/CRP/REG3A/F2/HAMP/TFR2/PTGS2/IL6R/INS/TNFRSF11A |
| GOBP | PYRIMIDINE CONTAINING COMPOUND SALVAGE | 11 | 0.791112401 | 1.963268015 | 0.000240891 | 0.002532556 | 0.001769031 | 964 | tags=45%, list=5%, signal=43% | TYMP/UPP1/TK1/UCK2/PUDP |
| GOBP | B CELL MEDIATED IMMUNITY | 130 | 0.40669545 | 1.683851616 | 0.000238812 | 0.002514641 | 0.001756517 | 5845 | tags=45%, list=28%, signal=32% | IRF7/NOD2/C1QBP/IL4R/MYD88/BCL3/SUSD4/EXOSC3/EXO1/CD27/BATF/NSD2/MAD2L2/RNF168/TFRC/HMCES/C2/C1QB/HLA-E/SLA2/LIG4/HSPD1/TLR8/TNF/XCL1/ERCC1/RNF8/PRKCD/BCL10/NECTIN2/C1QC/IGHV7-81/HLA-G/IL10/FOXP3/PARP3/TRDC/CR1/TNFSF13/PTPRC/CD28/FCER1G/CD40/CRP/TBX21/SLC15A4/HLA-DQB1/C1R/C4BPA/MLH1/KMT5C/CLCF1/LTA/TP53BP1/C1QA/IGHG1/CD226/PAXIP1 |
| GOBP | POSITIVE REGULATION OF LEUKOCYTE PROLIFERATION | 141 | 0.399541189 | 1.665829717 | 0.000236071 | 0.00248968 | 0.001739082 | 4807 | tags=45%, list=23%, signal=35% | CD24/CD274/PNP/SLC7A1/MYD88/PRKCQ/PYCARD/SASH3/JAK3/MIF/IL1B/LYN/CD6/CORO1A/IL12B/CDKN1A/SYK/IL6/LGALS9/CCL19/PTPN22/IL23A/GPR183/IL2RA/TFRC/HLA-DMB/FADD/HLA-E/TNFSF9/CD86/BST2/CCL5/DNAJA3/EBI3/XCL1/CD3E/ANXA1/PELI1/IL12RB1/CCR2/CD38/LILRB2/IL15/TNFRSF4/CARD11/ZAP70/RASAL3/TNFSF13B/CD80/FOXP3/EPO/VCAM1/NCK2/CCDC88B/CD1D/CLECL1/PTPRC/CD28/AIF1/AGER/CD40/MAPK3/TICAM1 |
| GOBP | ORGAN GROWTH | 144 | -0.363025839 | -1.64972607 | 0.000229952 | 0.002428963 | 0.00169667 | 4040 | tags=47%, list=19%, signal=38% | GLI1/YBX3/SMAD2/FGF2/PTPN11/ARID2/HEG1/PAK1/DLL1/IGF1/IL7/ECM1/LATS1/AKAP6/PSAP/PI16/S1PR1/TGFB2/KCNK2/PARP2/WWC3/SAV1/UBE3A/PTEN/FOXC2/VGLL4/FGFR3/ERBB4/JARID2/PDGFRB/THBS3/DDR2/RGS2/PDLIM5/YAP1/LEP/EDN1/STK3/NOG/TSKU/EVC/ESR1/LATS2/PLAG1/FOXC1/FGFR2/SORBS2/BMPR1A/PPARA/DIPK2A/MEF2C/RBP4/AKAP13/WNT2/ADRA1A/PSAPL1/RBPJ/MAPK1/TGFBR2/ZFPM2/AR/SPRY2/BCL2/LEPR/MEIS1/SOD1/TGFBR3 |
| GOBP | SPINAL CORD MOTOR NEURON DIFFERENTIATION | 29 | -0.600504577 | -2.004613488 | 0.000225562 | 0.002386335 | 0.001666894 | 3421 | tags=34%, list=16%, signal=29% | IFT172/TCTN1/LMO4/SOX4/DYNC2H1/ZC4H2/HOXC10/PTCH1/GLI2/GLI3 |
| GOCC | ORGANELLE ENVELOPE LUMEN | 93 | 0.45613501 | 1.791672351 | 0.000224216 | 0.00237584 | 0.001659562 | 4094 | tags=49%, list=20%, signal=40% | CYCS/AK2/PRELID1/CIAPIN1/TIMM8B/PRELID3B/STOML2/TIMM13/DTYMK/SHMT2/STMP1/TIMM23/PNPT1/TIMM10/COA4/AIFM1/CLPB/TRAP1/CHCHD2/CHCHD4/COA6/LYN/TIMM8A/HAX1/NDUFB7/UQCC2/CHCHD5/IMMT/CHCHD7/NDUFA8/COX17/TRIAP1/PARK7/SDHAF3/DIABLO/CDC25C/COA7/HTRA2/THOP1/AGK/PTGES/NDUFS5/PANK2/CPOX/FGR/NDUFS1 |
| GOBP | CLEAVAGE INVOLVED IN RRNA PROCESSING | 27 | 0.635239902 | 1.973906184 | 0.000216971 | 0.0023027 | 0.001608473 | 3999 | tags=59%, list=19%, signal=48% | RRP36/TSR1/NHP2/EXOSC3/BOP1/NOP14/RPP40/EXOSC2/ERI1/ERI2/ABT1/NOB1/TBL3/RRS1/EXOSC8/NOL9 |
| GOMF | MOLECULAR CARRIER ACTIVITY | 64 | 0.494269526 | 1.83203671 | 0.00021277 | 0.002261691 | 0.001579828 | 3589 | tags=38%, list=17%, signal=31% | RAN/KPNA2/ATOX1/SCO2/NDUFAB1/IPO4/SCO1/EMC6/EMC3/EMC8/HIKESHI/EMC9/XPO6/CSE1L/XPO5/COX17/KPNB1/PARK7/IPCEF1/EMC1/FXN/EMC7/KPNA3/NUTF2 |
| GOBP | POSITIVE REGULATION OF ACTIN FILAMENT BUNDLE ASSEMBLY | 61 | -0.465624832 | -1.820100641 | 0.000211525 | 0.002252024 | 0.001573075 | 3502 | tags=43%, list=17%, signal=36% | ARHGEF10L/NRP1/TAC1/PXN/ABL1/CCN2/RGCC/SWAP70/S100A10/ARHGEF15/RAPGEF3/TESK1/SORBS3/PFN2/ARHGEF5/ROCK2/SMAD3/FERMT2/SDC4/MYOC/TSC1/LPAR1/LIMCH1/SYNPO2/TPM1/ARHGEF10 |
| GOBP | REGULATION OF MYELOID LEUKOCYTE MEDIATED IMMUNITY | 52 | 0.529924233 | 1.899321066 | 0.000211243 | 0.002252024 | 0.001573075 | 4049 | tags=50%, list=19%, signal=40% | IL4R/PTAFR/PLA2G3/DNASE1L3/ARG1/LYN/HMOX1/CD177/SYK/RAC2/LGALS9/ITGB2/CXCL6/VAMP8/HLA-E/C12orf4/VAMP7/DDX21/DDX58/ADGRE2/TYROBP/STX4/CCR2/UNC13D/CD300A/FGR |
| GOCC | RESPIRATORY CHAIN COMPLEX IV | 21 | 0.677626176 | 1.974895423 | 0.000210801 | 0.002251445 | 0.001572671 | 3949 | tags=62%, list=19%, signal=50% | COX6B1/COX7B/UQCRFS1/COX5A/COX8A/C15orf48/COA6/NDUFA4/COX6A1/UQCRC2/COX7C/COX4I1/COX7B2 |
| GOBP | ZYMOGEN ACTIVATION | 56 | 0.523087031 | 1.900094652 | 0.000209037 | 0.002236167 | 0.001561999 | 4996 | tags=43%, list=24%, signal=33% | ENO1/PGK1/IFI16/PYCARD/PLGRKT/F12/BAK1/PRSS3/PLAT/CTSL/PLAU/CASP8/FADD/FURIN/CIDEB/NLRC4/LGMN/HP/SERPINE1/CYFIP2/SERPINF2/SERPINE2/F9/C1R |
| GOCC | COSTAMERE | 18 | -0.685826338 | -1.991879069 | 0.000208655 | 0.002235639 | 0.00156163 | 4126 | tags=83%, list=20%, signal=67% | PLEC/KRT8/VCL/KRT19/ANK2/DAG1/SVIL/SDC4/SYNM/FLNC/FXR1/DMD/AHNAK/PGM5/AHNAK2 |
| GOMF | ATPASE ACTIVITY COUPLED TO TRANSMEMBRANE MOVEMENT OF IONS ROTATIONAL MECHANISM | 22 | 0.654696427 | 1.938045235 | 0.000207913 | 0.002231245 | 0.001558561 | 2676 | tags=55%, list=13%, signal=48% | ATP6V0D1/ATP6V1B2/ATP5F1B/ATP6V1G1/ATP6V0B/ATP6V1F/ATP6V1D/ATP6V1C1/ATP6V1C2/ATP6V1H/ATP6V1A/ATP6V0E1 |
| GOBP | EXTERNAL ENCAPSULATING STRUCTURE ORGANIZATION | 383 | -0.281628998 | -1.438865124 | 0.000207188 | 0.002227018 | 0.001555608 | 5389 | tags=37%, list=26%, signal=28% | ANGPTL7/NTNG2/ITGB3/ADAMTS17/COL5A1/SFRP2/FBN1/VWF/COL1A2/SULF2/COL7A1/COL5A3/VCAN/CMA1/LOXL1/LOXL3/ELANE/NF1/ATP7A/FLOT1/DDR1/PDGFB/MELTF/COL6A1/BGN/ITGAM/HTRA1/PLOD3/LRP1/HPSE2/PHLDB2/COL6A2/KDR/FLRT2/CCDC80/POMT1/ITGB8/TPSAB1/TGFB2/GAS2/ADAMTSL4/BMP2/MMP2/ITGA1/RAMP2/CTSK/ELN/ITGAV/EFEMP2/COL4A5/MMP27/SLC2A10/MATN4/FOXC2/CYP1B1/ADAMTS5/COL28A1/LAMA2/CREB3L1/SH3PXD2B/COL8A1/MFAP2/WASHC1/APP/ABL1/ICAM2/COL23A1/ITGA5/CCN2/SMOC2/MMP16/AGT/RGCC/CTSG/LOX/AEBP1/JAM2/COL17A1/DDR2/NTN4/MFAP4/RECK/ITGA11/PECAM1/CLASP1/FBLN2/ADAMTSL1/CHADL/ANTXR1/LAMA4/COL12A1/MMP28/HSPG2/SH3PXD2A/DPT/A2M/PDGFA/CTSV/MFAP5/KAZALD1/GPM6B/FOXC1/ITGA7/GREM1/LAMA3/ITGA8/ITGB1/GAS6/TNXB/LAMB2/FN1/COL8A2/ATXN1L/ECM2/COL16A1/PHLDB1/SCUBE3/FBLN5/DAG1/VIT/LAMC3/FBLN1/SMAD3/LAMC1/NDNF/LAMC2/TIMP2/DCN/ITGA9/CRTAP/LAMA5/LOXL4/PDGFRA/ITGB5/PAPLN/MYH11/JAM3/CAV2/CAV1/LAMB4/POSTN/OLFML2A/TGFBI |
| GOCC | PROTON TRANSPORTING TWO SECTOR ATPASE COMPLEX PROTON TRANSPORTING DOMAIN | 22 | 0.656301049 | 1.942795271 | 0.000204027 | 0.002196559 | 0.001534332 | 3724 | tags=59%, list=18%, signal=49% | ATP5MF/ATP5MC3/ATP6V0D1/ATP5PF/ATP5MC1/ATP6V0B/ATP5PB/ATP5ME/ATP5MG/ATP5PO/ATP6V0E1/ATP5PD/ATP6V0A2 |
| GOBP | SPLICEOSOMAL SNRNP ASSEMBLY | 37 | 0.57743462 | 1.922003176 | 0.00020349 | 0.002194293 | 0.001532749 | 3127 | tags=43%, list=15%, signal=37% | SNRPG/SNRPF/SNRPD1/PRMT5/LSM2/SNRPD3/WDR77/CD2BP2/GEMIN6/SNRPC/GEMIN7/LSM4/SNRPB/PRPF31/STRAP/GEMIN2 |
| GOBP | PROTEIN INSERTION INTO MEMBRANE | 76 | 0.487880494 | 1.851121326 | 0.000198806 | 0.002147229 | 0.001499874 | 4058 | tags=43%, list=19%, signal=35% | TFDP1/GZMB/HSPA4/TIMM8B/RTP4/TOMM22/NMT1/NDUFA13/YWHAQ/TIMM13/SFN/TIMM10/UBL4A/MAIP1/BID/ROMO1/EMC6/PDCD5/EMC3/EMC8/EMC9/CASP8/TP63/YWHAZ/GET3/EMC1/BAX/AGK/EMC7/MAPK8/SGTA/BCS1L/SAMM50 |
| GOBP | POSITIVE REGULATION OF INTERLEUKIN 8 PRODUCTION | 56 | 0.524925297 | 1.90677209 | 0.000194985 | 0.002109352 | 0.001473417 | 3874 | tags=45%, list=19%, signal=36% | WNT5A/NOD2/CLEC7A/MYD88/AFAP1L2/PYCARD/TLR2/STAT3/CD2/LAMTOR5/IL1B/FCN1/SYK/IL6/LGALS9/FFAR2/CD58/FADD/PARK7/TLR8/TNF/DDX58/BCL10/CD244/SERPINE1 |
| GOBP | POSITIVE REGULATION OF CELL KILLING | 58 | 0.514182667 | 1.8645829 | 0.000194788 | 0.002109352 | 0.001473417 | 4406 | tags=55%, list=21%, signal=44% | CLEC7A/CYRIB/ARG1/PRF1/IFNG/NOS2/RAET1E/IL12B/RASGRP1/SYK/IL18RAP/SH2D1A/IL23A/LAG3/FADD/HLA-E/NCR3/CRTAM/XCL1/KLRD1/CD1B/HLA-DRA/IL12RB1/TYROBP/B2M/HLA-F/HLA-B/NECTIN2/HLA-G/VAV1/CD1D/PTPRC |
| GOBP | DNA TEMPLATED TRANSCRIPTION TERMINATION | 72 | 0.483601515 | 1.816903086 | 0.000194683 | 0.002109352 | 0.001473417 | 5659 | tags=57%, list=27%, signal=42% | SNRPG/POLR2H/SNRPF/NCBP1/POLR2F/PRMT5/ZMPSTE24/SNRPD3/CPSF3/LSM10/CSTF2/POLR3K/POLR1H/POLR2L/POLR1C/TAF1D/SNRPB/POLR1B/CDK7/CLP1/POLR2E/POLR1G/TTF2/CCNH/CPSF4/XRN2/SSU72/CSTF1/WDR33/CSTF3/POLR1A/TAF1B/PAPOLA/GTF2H1/SLBP/SYMPK/SNRPE/NCBP2/TAF1A/MED18/ZNF473 |
| GOMF | PEPTIDE TRANSMEMBRANE TRANSPORTER ACTIVITY | 32 | 0.591466433 | 1.909391731 | 0.000192303 | 0.002090442 | 0.001460207 | 4824 | tags=59%, list=23%, signal=46% | ABCC1/TOMM22/SEC61G/TIMM17A/TAP1/TIMM23/TOMM40L/TAP2/TOMM40/SLC15A1/SEC61A1/TAPBP/SLC15A3/TIMM17B/MCL1/GJA1/ABCC5/SEC61A2/SLC15A4 |
| GOBP | REGULATION OF ACTIN FILAMENT BASED PROCESS | 374 | -0.283865513 | -1.450773178 | 0.000190922 | 0.002078793 | 0.00145207 | 3136 | tags=27%, list=15%, signal=23% | PXN/DLG1/WASHC1/ABL1/CCN2/PDGFRB/TENM1/WASF2/RGCC/BCAS3/TMSB4X/CXCL12/FAM107A/EFNA5/SEMA3E/SWAP70/BST1/CLASP1/S100A10/EDN1/SLIT2/ARHGEF15/KANK4/RHOBTB1/FZD10/RAPGEF3/RHOQ/ADD1/MET/FMN1/PDGFA/TMOD2/WASF3/FGF13/ALMS1/FER/PLEKHH2/TMSB15B/SHANK3/TESK1/GPM6B/DSTN/DSG2/ANK2/DSP/BIN1/VILL/SORBS3/ARHGAP35/NTF3/PAM/PFN2/SPTAN1/RHOB/ARHGEF5/RICTOR/AKAP13/IQGAP1/ROCK2/ARHGAP6/SCIN/SMAD3/FERMT2/FHOD3/SVIL/ABI2/SDC4/CFL2/PLN/MYOC/AKAP9/PAK3/TNNC1/TSC1/ATP1A2/LPAR1/EPS8/SPTBN2/LIMCH1/CORO2B/DIXDC1/MYADM/PIK3R1/ADD3/PDGFRA/LMOD1/DLC1/DBN1/JAM3/SYNPO2/TPM1/TMOD1/NOTCH2/CGNL1/GSN/CAV1/ARHGEF10/KANK1/RHOBTB3/SPTBN1/CCL27 |
| GOBP | PROTEIN DNA COMPLEX SUBUNIT ORGANIZATION | 250 | 0.351788669 | 1.565694088 | 0.000189129 | 0.002062609 | 0.001440766 | 3657 | tags=30%, list=18%, signal=25% | DLGAP5/RUVBL1/H2AX/PSME4/CENPN/MCM6/MACROH2A1/PSMC4/KNL1/RPA3/RAD51/CENPW/MCM4/CDC45/CENPF/MCM5/CENPX/MCM2/ASF1B/CENPE/OIP5/HJURP/CENPM/CENPK/CENPA/IPO4/PSMC3/CDT1/MYC/POLE3/MCM7/RBX1/PIH1D1/CENPS/MCM3/GTF2A2/HAT1/PSMC5/SMARCD3/PSMC2/NASP/PSMC6/CETN2/NPM1/GRWD1/CENPH/CENPI/NAP1L4/HMGB2/H2BC9/MACROH2A2/CDK7/H3C10/HELLS/PAF1/GTF2E1/ZNHIT1/MIS18A/SMARCA4/RB1/CCNH/TAF11/PARP1/SMARCA5/HMGA1/RNF8/CAND1/MIS12/GTF2B/SPTY2D1/DAXX/CENPL/CHAF1A/NAA60/CENPO |
| GOBP | NEGATIVE REGULATION OF GROWTH | 233 | -0.323065435 | -1.552782746 | 0.000186872 | 0.002041312 | 0.001425889 | 4470 | tags=39%, list=21%, signal=31% | PI16/MSX1/SEMA3A/UBAP2/WFDC1/CCDC85B/BTG1/NDRG3/RBBP7/TGFB2/KCNK2/CGRRF1/MSTN/WWC3/SAV1/IP6K2/SOCS2/SEMA6D/PTEN/VGLL4/NRP1/MT1H/OSGIN2/FGFR3/NPR1/RAI1/SIRT1/ING5/BMP4/MEG3/JARID2/SCGB3A1/DACT3/ST7L/ENPP1/ING1/RTN4R/RGS2/SEMA3E/NKD1/SLIT2/STK3/NOG/CCN3/SPART/SEMA4G/SERTAD2/SEMA6A/ADRB1/DUSP10/FGF13/PPARG/SEMA3B/ACVRL1/RYK/SLIT3/PTPRS/RERG/SMARCA2/GREM1/TSPYL2/SH3BP4/CAPRIN2/PPARA/GPC3/FRZB/MT1X/RBP4/MAP2/IGFBP5/NTN1/CDKN1B/APBB2/ING4/DCBLD2/SMAD3/RTN4/TGFBR2/PTCH1/BCL6/SEMA3G/JADE1/ACVR1B/BBS2/NOTCH2/BCL2/MEIS1/DNAJB2/FHL1/CRYAB/ADRB2 |
| GOCC | RIBOSOMAL SUBUNIT | 176 | 0.382613425 | 1.637557242 | 0.00018569 | 0.002031707 | 0.00141918 | 2788 | tags=35%, list=13%, signal=30% | MRPL15/MRPL51/RPL26L1/MRPL35/MRPS11/MRPS7/MRPL47/MRPL13/MCTS1/MRPS16/MRPS15/ISG15/MRPL46/MRPS12/MRPL37/MRPS17/MRPL3/MRPL27/MRPL36/MRPL11/MRPL17/MRPL42/MRPL52/NDUFAB1/MRPL32/MRPL14/MRPS21/MRPS22/MRPS14/MRPL20/MRPS33/MRPL58/MRPL54/MRPS18C/MRPL19/MRPL50/MRPL4/MRPL16/MRPL22/MRPS36/MRPL12/MRPL21/MRPL41/MRPL39/MRPL23/MPV17L2/MRPL28/MRPS28/MRPS2/MRPS35/MRPS6/MRPL18/MRPS30/MRPL55/MRPL48/MRPL57/MRPS27/MRPS18A/MRPS34/RPL39L/MRPL34 |
| GOCC | SMALL NUCLEAR RIBONUCLEOPROTEIN COMPLEX | 69 | 0.48605223 | 1.815834335 | 0.000183593 | 0.002012033 | 0.001405437 | 2761 | tags=36%, list=13%, signal=32% | SNRPG/SNRPF/SNRPD1/LSM2/TXNL4A/SF3B3/LSM7/SNRPD3/EFTUD2/SF3B5/CD2BP2/SNU13/PPIH/LSM10/SNRPA1/SNRPC/SF3B6/LSM5/LSM4/SNRPB/LUC7L2/PRPF31/USP39/LSM6/PRPF4 |
| GOMF | UBIQUITIN LIKE PROTEIN LIGASE BINDING | 302 | 0.336456311 | 1.529098603 | 0.000182088 | 0.001998804 | 0.001396197 | 4190 | tags=35%, list=20%, signal=28% | CCNB1/TPI1/STAT1/PARP9/SLC25A5/CXCR4/UBE2C/FZD5/UBE2L6/UBE2N/PSMA3/UBE2T/EIF4E2/VCP/AURKA/ISG15/DTX3L/HIF1A/UBE2L3/HSPA5/CCT2/HM13/RALB/HSPA9/TRIB2/ELOB/CEBPB/PSMD1/TOLLIP/IKBKE/LYN/BID/GPI/CASP10/MFHAS1/UBE2D1/STAM/HSPA8/CDKN1A/RBX1/UBE2A/GLMN/PA2G4/TRAF1/TRAF3/XRCC5/PTPN22/CACUL1/ITCH/STAT2/IKBKG/AUP1/RANGAP1/CASP8/CUL1/CHEK2/ARRDC1/UBE2G1/DDRGK1/PRKAR2A/HSPBP1/LAPTM5/LTBR/TNFRSF1B/BOK/RAD18/MID1/TCP1/YWHAZ/H2BC9/PIAS2/BLZF1/HSPD1/SMC6/BRCA1/UBE2J2/UQCRC1/GPR37/PML/NDUFS2/RB1/NEK6/DDX58/PRDX6/MDM2/RNF8/ARRB1/BCL10/USP2/DAXX/NPLOC4/BECN1/OTUB1/UBE2W/SCAMP3/DIO2/YOD1/TRAF4/LRPPRC/ERLIN1/TRAF2/SLF1/UBE2J1/FZD6/WRAP53 |
| GOBP | SEX DIFFERENTIATION | 252 | -0.307130062 | -1.486959744 | 0.000179735 | 0.001976208 | 0.001380413 | 3121 | tags=26%, list=15%, signal=23% | SIRT1/ROBO2/PDGFRB/KITLG/ATRX/CSDE1/TESC/MERTK/TBX3/DACH1/LEP/SPATA2/MGST1/GNRH1/SLIT2/LHCGR/SOX8/WDR48/ESR1/FGF9/CTNNA1/KDM5A/PGR/DMRT2/SLIT3/AKR1C3/LRP6/FOXC1/PBX1/BMPR1A/ZFX/ROR2/RBP4/HOXA11/SMAD5/BMPR1B/IRX5/STAR/ZFPM2/OSR1/FZD4/HOXA10/PKD1/IMMP2L/WDR19/STAT5B/PLEKHA1/ACVR1B/NUPR1/AGO4/AXL/PDGFRA/ASB1/INHBB/FST/RNF38/AR/PATZ1/KIT/BCL2/SOD1/RHOBTB3/WNT2B/CCND1/ANG/GATA3 |
| GOBP | RESPONSE TO TOPOLOGICALLY INCORRECT PROTEIN | 199 | 0.363190723 | 1.580468298 | 0.000179175 | 0.001973274 | 0.001378364 | 4453 | tags=38%, list=21%, signal=30% | HSPA4/MANF/CHAC1/VCP/HSPA4L/F12/ATP6V0D1/ERP44/BAK1/EIF2S1/HSPA5/FBXO6/SERP1/ERO1A/PTPN2/CXCL8/DNAJB11/HSPA14/HSPA9/UFD1/YIF1A/HSPE1/CCL2/BAG3/COPS5/SRPRB/HSPA8/HDGF/SHC1/HSPH1/HYOU1/EDEM1/PREB/TOR1A/DERL2/DNAJB1/AUP1/DDRGK1/PDIA6/ATF4/BOK/SDF2L1/HSPD1/EDEM2/GET3/UBE2J2/CANX/RNF126/EDEM3/COMP/BAX/PPP1R15B/WIPI1/SSR1/MYDGF/ASNS/DAXX/TMEM129/TOR1B/EXTL3/PPP2R5B/UBE2W/STT3B/GSK3A/DNAJB5/YOD1/TMEM33/XBP1/DNAJB12/ERP27/NCK2/ABCA7/DNAJA1/ACADVL/TPP1 |
| GOCC | TIM23 MITOCHONDRIAL IMPORT INNER MEMBRANE TRANSLOCASE COMPLEX | 13 | 0.770130597 | 1.995323162 | 0.000178872 | 0.00197317 | 0.001378291 | 4029 | tags=85%, list=19%, signal=68% | GRPEL1/TIMM50/TIMM17A/TIMM23/TIMM10/ROMO1/DNAJC15/TIMM21/GRPEL2/TIMM17B/DNAJC19 |
| GOMF | TOLL LIKE RECEPTOR BINDING | 12 | 0.78525375 | 1.9905542 | 0.000177817 | 0.001964766 | 0.00137242 | 2707 | tags=67%, list=13%, signal=58% | S100A9/S100A8/MYD88/TLR2/TOLLIP/SYK/CD36/LY96 |
| GOBP | MONONUCLEAR CELL MIGRATION | 174 | 0.382994537 | 1.640491546 | 0.000176662 | 0.001955217 | 0.001365751 | 4814 | tags=42%, list=23%, signal=33% | S100A12/CXCR2/C10orf99/WNT5A/CCL20/CXCR4/C1QBP/S100A7/PYCARD/CCR7/CXCL13/CYP7B1/CXCL16/CXCL10/CCL18/CCL4/CCL22/ADAM17/S100A14/CH25H/CCL2/LYN/SLAMF8/SPNS2/DEFB124/CCL8/IL6/ITGA4/RIPK3/LGALS9/CCL7/CCL19/CCR5/CXCL17/GPR183/DOCK8/OXSR1/ADAM10/CXCL11/ITGAL/ADAM8/FADD/WASL/PIK3CD/EXT1/CRTAM/CCL5/TNF/XCL1/CCR1/ANXA1/ICAM1/CCR2/CCL17/GCSAM/LGMN/TMEM102/ZAP70/JAML/SERPINE1/MSN/GPR15/CKLF/MYO1G/AKIRIN1/C3AR1/CXCR1/CXCR3/AIF1/AGER/AIRE/MAPK3/TBX21 |
| GOBP | AUTONOMIC NERVOUS SYSTEM DEVELOPMENT | 46 | -0.503397677 | -1.879705358 | 0.000174794 | 0.001937732 | 0.001353537 | 5087 | tags=52%, list=24%, signal=40% | RET/SOX11/PLXNA4/INSM1/EDNRB/TBX1/NF1/SEMA3A/CTNNB1/EGR2/NRP1/HES1/TFAP2A/SOX10/SOX4/SOX8/FZD3/HOXB2/GFRA3/FN1/ADARB1/TFAP2B/KIF26A/GATA3 |
| GOBP | POSITIVE REGULATION OF CELL JUNCTION ASSEMBLY | 91 | -0.419675789 | -1.788431477 | 0.000174602 | 0.001937732 | 0.001353537 | 5183 | tags=47%, list=25%, signal=36% | NPHP1/AMIGO2/LRRTM2/SLITRK5/EPHB3/FLOT1/CUX2/TEK/NRXN1/KDR/FLRT2/AMIGO1/LINGO2/NRP1/SLITRK2/NPHP4/ABL1/SRPX2/CBLN1/AGT/CLDN3/S100A10/CLSTN1/BHLHB9/LRRN1/NLGN1/FMN1/CLDN5/NTRK2/COL16A1/CLSTN2/IQGAP1/SMAD3/FERMT2/SDC4/DLG5/MYOC/EPHB1/TSC1/SLITRK6/CAV1/EEF2K/CLDN1 |
| GOBP | REGULATION OF DEFENSE RESPONSE TO VIRUS BY HOST | 40 | 0.568989343 | 1.9203733 | 0.000171716 | 0.001909914 | 0.001334106 | 3674 | tags=45%, list=18%, signal=37% | ZC3H12A/STAT1/PARP9/PYCARD/DTX3L/MMP12/TARBP2/AIM2/CGAS/IL12B/PTPN22/IL23A/MICB/DDX58/PQBP1/IL12RB1/IL15/LILRB1 |
| GOBP | HEART VALVE DEVELOPMENT | 60 | -0.471024712 | -1.831175757 | 0.000170656 | 0.001901273 | 0.00132807 | 4040 | tags=42%, list=19%, signal=34% | TGFB2/BMP2/ELN/BMP4/ROBO2/JAG1/MATR3/SOX4/SHOX2/SLIT2/EFNA1/TWIST1/ZBTB14/SLIT3/BMPR1A/MEF2C/ROCK2/PDE2A/RBPJ/MDM4/TGFBR2/DCHS1/NFATC1/NOTCH2/GATA3 |
| GOBP | ALPHA BETA T CELL DIFFERENTIATION | 103 | 0.438374358 | 1.75883718 | 0.000170049 | 0.001897652 | 0.00132554 | 4101 | tags=43%, list=20%, signal=34% | ZC3H12A/NFKBIZ/PNP/IL4R/CD83/ENTPD7/RSAD2/BCL3/IRF1/PRDM1/STAT3/CTSL/SASH3/AP3D1/JAK3/IFNG/RELB/SOCS1/CBFB/IL12B/SYK/IL6/LGALS9/CCL19/AP3B1/IL23A/BATF/GPR183/ITK/LILRB4/CD86/EOMES/LEF1/SEMA4A/NFKBID/ANXA1/HLA-DRA/IL12RB1/ZAP70/NLRP3/MTOR/CD80/FOXP3/ZNF683 |
| GOBP | T CELL DIFFERENTIATION | 238 | 0.359177729 | 1.594995963 | 0.000169921 | 0.001897652 | 0.00132554 | 4101 | tags=38%, list=20%, signal=31% | ZC3H12A/NFKBIZ/PNP/FZD5/PRELID1/IL4R/CCR7/CD83/ENTPD7/VNN1/RSAD2/PTPN2/LCK/BCL3/IRF1/ADAM17/PRDM1/STAT3/CTSL/CD2/SASH3/RHOH/AP3D1/JAK3/IFNG/RELB/IL1B/CD3D/ATG5/SOCS1/CBFB/IL12B/FANCD2/ZC3H8/SYK/IL7R/IL6/RIPK3/LGALS9/CCL19/CD27/AP3B1/PTPN22/IL23A/BATF/GPR183/LAG3/CD3G/PRKDC/IL2RA/JMJD6/ITK/ADAM8/FADD/LILRB4/PIK3CD/TNFSF9/CD86/LIG4/CRTAM/TMEM131L/CD8A/EOMES/DNAJA3/LEF1/SEMA4A/IL36B/NFKBID/THEMIS/IL1RL2/CD3E/ANXA1/HLA-DRA/IL12RB1/CHD7/CCR2/LILRB2/B2M/FOXN1/IL15/CARD11/ZAP70/DOCK2/NLRP3/HLA-G/MTOR/VAV1/CD80/FOXP3/XBP1/ZNF683 |
| GOCC | MITOTIC SPINDLE | 157 | 0.393269299 | 1.669182243 | 0.00016877 | 0.001889663 | 0.00131996 | 2746 | tags=30%, list=13%, signal=26% | CXCR2/CDK1/NUSAP1/AURKB/KIF18B/PRC1/MAD2L1/ASPM/AURKA/TPX2/SPAG5/TUBG1/CDC6/KIF11/TACC3/CENPE/ECT2/KIF23/SKA3/SKA1/FAM83D/RACGAP1/ESPL1/RMDN1/HAUS1/KIF18A/PLK1/RMDN2/BCCIP/LIMK2/DYNLL1/KIF20B/RCC2/TAF1D/CDC7/IKBKG/RANGAP1/RAE1/CKAP2L/NUDCD2/SPOUT1/KIF22/TRAPPC14/SMC6/GPSM2/KIFC1/MAPRE1 |
| GOBP | TRANSCRIPTION COUPLED NUCLEOTIDE EXCISION REPAIR | 72 | 0.486456707 | 1.827630113 | 0.000166673 | 0.001869301 | 0.001305737 | 5193 | tags=53%, list=25%, signal=40% | COPS3/PCNA/POLR2H/RPA3/POLR2F/COPS5/POLD2/RBX1/RFC2/COPS2/POLR2L/RFC3/AQR/POLR2G/HMGN1/POLR2D/CDK7/ISY1/POLR2E/POLD4/ERCC8/GPS1/CCNH/COPS6/ERCC1/RFC4/COPS4/POLD3/POLR2I/COPS7A/RPA1/POLR2J/POLD1/RFC5/PRPF19/LIG1/GTF2H1/ERCC6 |
| GOBP | MONOSACCHARIDE CATABOLIC PROCESS | 60 | 0.49976106 | 1.829220195 | 0.000164674 | 0.001849962 | 0.001292228 | 3165 | tags=37%, list=15%, signal=31% | ENO1/TPI1/PGAM1/PGK1/PGM2/HK2/TIGAR/GALE/FUT2/ALDOA/FOXK2/FUT1/GAPDH/NUDT5/GPI/GALK1/RBKS/PFKFB2/PFKP/GLYCTK/PKM/ADPGK |
| GOBP | SYNAPSE ORGANIZATION | 399 | -0.27553147 | -1.408929351 | 0.000164084 | 0.001846428 | 0.001289759 | 4590 | tags=32%, list=22%, signal=25% | DAB2IP/ITGAM/CUX2/SYNPO/ERC1/HDAC6/DVL1/ARHGAP44/NRXN1/PCDHB13/PCLO/FLRT2/GPHN/DNER/OBSL1/TANC1/AMIGO1/LRRC4C/PCDHB11/LINGO2/GABRA2/COL4A5/NFASC/UBE3A/PTEN/PDZRN3/GABRB3/SHANK2/PPFIBP1/PLXND1/RELN/C3/ZDHHC2/SLITRK2/CTTNBP2/ERBB4/DLG1/APP/ABL1/WNT7B/SRPX2/ROBO2/DOK7/CBLN1/WASF2/SETD5/FZD1/LRRK2/PDLIM5/ZNF365/SEMA3E/PCDHB14/NPTN/NGEF/INSR/L1CAM/SNCG/SLITRK4/F2R/CLSTN1/MYOT/PCDHB2/BHLHB9/ARHGEF15/ZC4H2/LRRN1/PCDHB10/IGF1R/NLGN1/DKK1/CACNB2/PCDHB4/EFNA1/FARP1/ZDHHC15/PCDHB5/DIP2A/WASF3/FGF13/GNPAT/SHANK3/RYK/APOE/GPC4/GLRB/PTPRS/MECP2/GPC6/NTRK2/LAMB2/ADGRL3/CAPRIN2/SEPTIN11/PTPRF/PTN/MEF2C/SNCA/NTN1/APBB2/CLSTN2/DAG1/CNKSR2/ABI2/LRFN5/DLG5/PAK3/EPHB1/TSC1/SPARCL1/FYN/TMEM108/PALM/SPTBN2/PCDHB16/MYH10/ITSN1/SLITRK6/DBN1/LRP4/TUBA1A/NFIA/SYBU/EFNB2/CAST/MAP1B/UTRN/EEF2K |
| GOBP | PROTEIN TRANSMEMBRANE TRANSPORT | 57 | 0.518699172 | 1.882800377 | 0.000163203 | 0.001839597 | 0.001284988 | 3595 | tags=47%, list=17%, signal=39% | TOMM22/GRPEL1/SEC61G/TIMM50/HSPA5/TIMM17A/TIMM23/TOMM40L/AIFM1/SEC61B/TOMM40/CHCHD4/ROMO1/HSPA8/DNAJC15/PEX10/SEC61A1/TIMM44/TIMM21/HSPD1/GRPEL2/DNLZ/SRP54/PEX13/PEX2/TIMM17B/MCL1 |
| GOCC | OUTER MITOCHONDRIAL MEMBRANE PROTEIN COMPLEX | 20 | 0.696038703 | 2.011693767 | 0.000159791 | 0.001804165 | 0.001260238 | 1597 | tags=60%, list=8%, signal=55% | MFN1/TOMM22/TOMM5/MTX2/HSPA9/TOMM40L/MICOS10/TOMM40/MICOS13/MTX1/CHCHD3/IMMT |
| GOBP | CARDIAC CHAMBER DEVELOPMENT | 150 | -0.371339458 | -1.692318135 | 0.000158776 | 0.001795715 | 0.001254336 | 4733 | tags=37%, list=23%, signal=29% | SEMA3C/WNT11/HEY1/NPRL3/TEK/MED1/TMEM65/CRELD1/TGFB2/KCNK2/BMP2/SAV1/SOS1/FOXC2/NRP1/PLXND1/PPP1R13L/BMP4/ROBO2/HES1/JAG1/MATR3/TBX3/LMO4/DHRS3/SOX4/SHOX2/SLIT2/NOG/CPE/PARVA/ZBTB14/NPY5R/SLIT3/ANK2/DSP/FOXC1/FGFR2/BMPR1A/NPHP3/MEF2C/RBP4/MYOCD/WNT2/PDE2A/RBPJ/MDM4/TGFBR2/BMP7/ZFPM2/TNNC1/TPM1/NOTCH2/TGFBR3/GATA3 |
| GOMF | ANION TRANSMEMBRANE TRANSPORTER ACTIVITY | 435 | 0.308090117 | 1.435913462 | 0.000158241 | 0.001792691 | 0.001252224 | 4573 | tags=29%, list=22%, signal=23% | SLC6A14/SLC26A9/SLC5A1/SLC25A5/SLC7A1/SLC7A11/SFXN1/CLCA2/APOL1/ABCC1/FLVCR2/SLC35B1/ABCA12/TOMM22/SEC61G/SLC16A1/LRRC8B/SLC23A2/SLC16A6/SLC16A10/TIMM17A/TAP1/TIMM23/ABCB6/SLC7A5/TOMM40L/SLC25A13/TAP2/CLDN17/VDAC2/SLC28A3/CLCN3/SLC25A15/TOMM40/SLC5A6/SLC24A3/LRRC8D/SLC2A1/SLC38A5/ABCG1/SLC25A10/PACC1/SLC36A1/SLC25A25/SLC25A33/SLC25A51/SLC25A44/FXYD3/SFXN2/SLCO4A1/SLC6A15/SLC3A2/CD36/SLC4A11/SLC15A1/SLC52A2/CTNS/SLC16A9/XPR1/SLC25A11/SLC44A2/SLC9A3R1/SLC1A3/SLC26A4/SEC61A1/SLC51A/VDAC1/SLC37A4/ANO10/SLC2A3/AQP3/CLCN5/SLC37A2/TAPBP/SLC2A5/SLC25A19/GET3/SLC19A1/SLC35A4/SLC16A3/SFXN5/VDAC3/SLC38A9/MFSD10/GABRA4/SLC66A1/SLC38A7/SLC35B4/SLC15A3/SLC12A8/CLCN1/BSND/SLC6A20/SLC35D2/TIMM17B/SLC23A1/ABCC10/CLCA3P/SLC50A1/MCL1/SLC7A7/TTYH3/SLC36A4/SLC6A1/SLC25A32/SLC43A2/CLIC3/GJA1/SIDT1/SLC1A1/SLC9A3/SLC1A5/SLC1A4/SLC10A3/CLCN7/GABRQ/SLC13A4/SLC35A2/SLC25A12/MFSD4B/CLCA1/ABCC5/SLC6A19/SEC61A2/SLC6A11/CLCN4/SLC45A2 |
| GOBP | POSITIVE REGULATION OF ADAPTIVE IMMUNE RESPONSE | 98 | 0.444043795 | 1.76283223 | 0.00015778 | 0.001790479 | 0.001250678 | 5845 | tags=57%, list=28%, signal=41% | CD274/NFKBIZ/NOD2/FZD5/PRKCQ/PYCARD/RSAD2/CYRIB/SASH3/EXOSC3/IL1B/IL12B/IL6/IL23A/NSD2/MAD2L2/TFRC/HMCES/FADD/HLA-E/HSPD1/TNF/NFKBID/XCL1/CD1B/HLA-DRA/IL12RB1/CCR2/B2M/HLA-F/HLA-B/NECTIN2/NLRP3/TNFSF13B/HLA-G/FOXP3/TRAF2/FBXO38/TNFSF13/CD1D/NLRP10/PTPRC/CD28/MALT1/CD40/TBX21/HLA-A/PVR/MLH1/KMT5C/IL1R1/CLCF1/LTA/TP53BP1/CD226/PAXIP1 |
| GOMF | PROTON TRANSPORTING ATP SYNTHASE ACTIVITY ROTATIONAL MECHANISM | 15 | 0.741266835 | 2.005007137 | 0.000157445 | 0.001789706 | 0.001250139 | 3460 | tags=80%, list=17%, signal=67% | ATP5MF/ATP5PF/ATP5F1B/ATP5PB/ATP5ME/ATP5MG/ATP5F1C/ATP5PO/ATP6V1A/ATP5F1D/ATP5F1A/ATP5PD |
| GOBP | PHAGOSOME ACIDIFICATION | 28 | 0.630487433 | 1.978121905 | 0.000153547 | 0.001748357 | 0.001221255 | 2676 | tags=46%, list=13%, signal=41% | RAB38/ATP6V0D1/ATP6V1B2/ATP6V1G1/ATP6V0B/ATP6V1F/SLAMF8/ATP6V1D/ATP6V1C1/ATP6V1C2/ATP6V1H/ATP6V1A/ATP6V0E1 |
| GOBP | RESPONSE TO INTERLEUKIN 12 | 48 | 0.5474124 | 1.93447089 | 0.000153185 | 0.001747191 | 0.001220441 | 3970 | tags=54%, list=19%, signal=44% | PSME2/GSTO1/IL12RB2/HNRNPA2B1/PPIA/HSPA9/IFNG/MIF/TALDO1/P4HB/SNRPA1/IL12B/SOD2/CAPZA1/CNN2/LMNB1/TCP1/HNRNPF/CFL1/PITPNA/LCP1/IL12RB1/RPLP0/MSN/SERPINB2/IL10 |
| GOCC | WNT SIGNALOSOME | 11 | -0.791205102 | -2.024765785 | 0.000152912 | 0.001747049 | 0.001220342 | 4366 | tags=100%, list=21%, signal=79% | CTNNB1/LRP5/APC/FZD1/LRRK2/RECK/ADGRA2/LRP6/GSK3B/WNT2 |
| GOBP | ENDOCARDIAL CUSHION MORPHOGENESIS | 33 | -0.586769135 | -2.03703498 | 0.00015233 | 0.001743364 | 0.001217768 | 5056 | tags=48%, list=24%, signal=37% | SMAD4/HEY1/MSX1/TMEM100/TGFB2/BMP2/ROBO2/NOG/TWIST1/ACVRL1/BMPR1A/RBPJ/MDM4/TGFBR2/BMP7/DCHS1 |
| GOBP | POSITIVE REGULATION OF T CELL APOPTOTIC PROCESS | 12 | 0.78897994 | 1.999999787 | 0.000151698 | 0.001739087 | 0.00121478 | 2825 | tags=67%, list=14%, signal=58% | CD274/WNT5A/PRELID1/IDO1/ZC3H8/LGALS9/ADAM8/CCL5 |
| GOBP | RIBONUCLEOSIDE MONOPHOSPHATE BIOSYNTHETIC PROCESS | 34 | 0.594222165 | 1.95071934 | 0.000150497 | 0.001728267 | 0.001207223 | 5291 | tags=56%, list=25%, signal=42% | UPP1/UCK2/GART/APRT/ATIC/IMPDH1/HPRT1/AMPD3/ADSL/ADK/UMPS/GMPS/RFK/CAD/PRPS2/PAICS/LHPP/PPAT/CMPK1 |
| GOBP | NEGATIVE REGULATION OF NUCLEAR DIVISION | 54 | 0.529863944 | 1.919363481 | 0.000148668 | 0.001710194 | 0.001194598 | 2381 | tags=39%, list=11%, signal=35% | CCNB1/CDC20/NDC80/BUB1B/AURKB/MAD2L1/ZWINT/TTK/BUB1/TRIP13/AURKAIP1/CENPF/CDT1/ZNF207/PLK1/GEN1/MAD2L2/FBXO5/MAD2L1BP/TOM1L1/LCMT1 |
| GOCC | PROTEASOME REGULATORY PARTICLE BASE SUBCOMPLEX | 12 | 0.789427466 | 2.001134229 | 0.000147679 | 0.001701731 | 0.001188686 | 3166 | tags=83%, list=15%, signal=71% | PSMC4/PSMD2/PSMD1/PSMC3/PSMD9/PSMC5/PSMC2/PSMC6/PSMC1/PSMD10 |
| GOBP | CELLULAR RESPONSE TO HEAT | 113 | 0.429987409 | 1.748172811 | 0.000145729 | 0.001682143 | 0.001175004 | 4434 | tags=45%, list=21%, signal=36% | DNAJB6/VCP/RPA3/EIF2S1/CXCL10/ATP2A2/NUP37/IRAK1/NUP210/HSBP1/NUP88/CLPB/IER5/BAG3/LYN/HMOX1/HSPA8/CDKN1A/HSPH1/PDCD6/NUP50/HIKESHI/DNAJB1/POLR2D/RAE1/SEC13/DNAJC7/TFEC/CHORDC1/NUP155/HDAC2/NDC1/MLST8/HTRA2/DNAJC2/NUP188/NUP85/PTGES3/FKBP4/DAXX/MAPKAPK2/NUP205/NUP58/MTOR/NUP107/NUP93/ATR/POM121/RPA1/NUP160/AKT1S1 |
| GOBP | PROTEIN TARGETING TO MEMBRANE | 193 | -0.335492399 | -1.575801026 | 0.0001436 | 0.001660425 | 0.001159834 | 4893 | tags=44%, list=23%, signal=34% | GDI1/RPS12/TRAM2/RPS7/RPS18/MYO1C/RPL19/AQP11/ITGAM/PDZK1/RPL35/ZDHHC7/SRP14/RPL37A/ARL6/ZDHHC3/VPS37C/TRAM1/RPL24/RABGEF1/DMTN/RPS11/RPS5/RPS9/NACAD/RPL41/PIKFYVE/C2CD5/RPL23A/SGTB/ZDHHC2/TCAF1/RPS25/RPS15A/RPS15/RPL13A/RPL32/RPL7A/RPS27/RPL31/RPL18/RPL27/RPS8/RPL4/CHMP4B/RPL12/RPL9/ZDHHC15/RPS6/RPL13/NACA4P/RPL10A/SEC62/RPL11/RPL37/RPL14/RPS4X/RPLP2/SDCBP/RPS14/RPLP1/RPL8/GOLGA7/RPS17/RPS24/RPL22/RPS23/RPL23/ZDHHC9/ZDHHC11/RPL38/TRAM1L1/RPS20/RPL3/RPL15/RPL34/FYN/PARD3/RPS3A/RPL5/NACA/RPL10/RPL7/RPL35A/RPL30 |
| GOBP | NEGATIVE REGULATION OF VASCULAR PERMEABILITY | 15 | -0.727864185 | -2.049133199 | 0.000142758 | 0.001653527 | 0.001155016 | 3852 | tags=67%, list=18%, signal=54% | RAMP2/ANGPT1/PDE3A/SLIT2/CLDN5/ARHGAP35/AKAP12/PDE2A/FERMT2/DDAH1 |
| GOBP | EPIDERMAL CELL DIFFERENTIATION | 269 | 0.350080891 | 1.567769485 | 0.000141149 | 0.001637715 | 0.001143971 | 2941 | tags=24%, list=14%, signal=21% | KRT16/DSC2/PI3/KRT6A/KLK13/DSG3/SERPINB13/LCE3D/TGM1/SPRR1B/CDH3/WNT5A/EPHA2/SPRR3/KRT6B/CERS3/CNFN/S100A7/SPRR1A/IVL/HDAC1/MACROH2A1/ABCA12/PRKCH/TGM3/EZH2/KRT17/TMEM79/ST14/CSTA/TRIM16/ZBED2/IL20/SPRR2G/BCR/SFN/CTSL/CYP27B1/CBFB/CAPN1/PRSS8/KLK12/SULT2B1/PCSK6/OVOL2/KRT24/OVOL1/ZFP36/SLC9A3R1/EREG/ETV4/KRT78/PKP3/GRHL1/TP63/AQP3/KRT37/FURIN/MACROH2A2/PLAAT4/KEAP1/HDAC2/ADAM9/RPTN/UGCG |
| GOBP | REGULATION OF CELL DEVELOPMENT | 464 | -0.270412006 | -1.407499825 | 0.000141142 | 0.001637715 | 0.001143971 | 4184 | tags=31%, list=20%, signal=25% | CTNNB1/TRPC6/OBSL1/RUFY3/HDAC9/RFLNB/SORL1/SERPINF1/BMP2/EGR2/XRCC2/AMIGO1/RNF112/GPER1/ILK/TRIOBP/DMTN/SS18L1/TACSTD2/SEMA6D/PTEN/NRP1/HOOK3/PLXND1/VCL/VEGFC/EFNB3/RELN/TIAM2/PDE3A/ANKRD27/ABL1/BMP4/ROBO2/HES1/CXCL12/NAP1L1/RTN4R/SOX10/LRRK2/EFNA5/SPEN/ZNF365/SHOX2/SEMA3E/RECK/GOLGA4/NPTN/YAP1/NPR2/DIO3/S100A10/MACF1/L1CAM/PRUNE1/SLIT2/SNW1/NOG/IL6ST/ADIPOQ/PLCB1/SOX8/BHLHB9/FZD3/SPART/SEMA4G/DOCK5/MAP6/ARNTL/ADD1/SEMA6A/NEDD9/RFX3/DUSP10/FGF13/PPARG/CLDN5/CTNNA1/SEMA3B/PER2/PLAG1/LIF/SHANK3/CLOCK/ADGRA2/RYK/TESK1/TMEM98/BIN1/PTPRS/ASPA/NTRK2/FN1/BMPR1A/CAPRIN2/TRAK2/GSK3B/LIMS2/PTN/FRZB/DOCK7/MAP2/ELL3/HOXA11/NTN1/DAG1/ETV5/PCM1/SKI/WNT2/ROCK2/FBLN1/DOCK1/FERMT2/RTN4/NUMBL/FBXW7/BMP7/HOXB3/RAPGEF2/STAR/MYOC/PAK3/DAAM2/NIN/PLXNA2/SEMA3G/MYADM/BHLHE41/DBN1/ARHGEF7/LRP4/FBXO31/NOTCH2/GLI3/KIT/BCL2/POSTN/KANK1/AXIN2/MAP1B/EEF2K |
| GOBP | PHAGOSOME MATURATION | 46 | 0.541747111 | 1.883222173 | 0.000140927 | 0.001637715 | 0.001143971 | 2676 | tags=35%, list=13%, signal=30% | RAB38/MREG/ATP6V0D1/ATP6V1B2/ATP6V1G1/ATP6V0B/ATP6V1F/RAB31/CORO1A/SLAMF8/ATP6V1D/ATP6V1C1/ATP6V1C2/ATP6V1H/ATP6V1A/ATP6V0E1 |
| GOBP | PROTEIN PROCESSING | 215 | 0.37450761 | 1.644219307 | 0.00013984 | 0.001630982 | 0.001139268 | 3579 | tags=30%, list=17%, signal=25% | KLK13/ENO1/KLK6/PGK1/IFI16/TMPRSS4/CHAC1/CASP4/PYCARD/PLGRKT/F12/CASP1/ECE2/BAK1/PRSS3/STOML2/HM13/TIMM17A/PLAT/PCSK9/TMEM208/ADAM17/AOPEP/CTSL/ZMPSTE24/CST7/PSENEN/ACE2/SRGN/SEC11C/BCL2L12/PLAU/PCSK7/PCSK6/ADAM19/METAP2/PCSK1/SNX12/CARD16/LPCAT3/ADAM10/CASP8/ADAM8/FADD/CTSS/SPCS3/RCE1/FURIN/REN/PMPCA/COMP/MYH9/FXN/NLRP7/PARP1/PHEX/HTRA2/CASP3/DDI2/CIDEB/MYRF/NLRC4/BACE1/CARD18/LGMN |
| GOBP | REGULATION OF PHOSPHATIDYLINOSITOL 3 KINASE SIGNALING | 122 | -0.386955705 | -1.708475148 | 0.000128599 | 0.001502484 | 0.00104951 | 4723 | tags=45%, list=23%, signal=35% | PIP5K1C/INPP5E/PDGFB/DAB2IP/PTPN13/TEK/KDR/TGFB2/PIP4K2A/ANGPT1/GPER1/PIK3CA/UBE3A/PTEN/PIP5K1B/ERBB3/IL18/ERBB4/SIRT1/PDGFRB/AGT/INSR/LEP/F2R/IGF1R/ROR1/TWIST1/PDGFA/EGF/EGFR/PTK2/NTF3/PIK3CB/PIK3IP1/NTRK2/FN1/KLF4/FGFR1/PDGFD/ROR2/DIPK2A/MAPK1/PIP4K2B/MYOC/DCN/FYN/PPP2R5C/PIK3R1/PDGFRA/KIT/IRS2/CAT/SERPINA12/NOP53/PDGFC |
| GOBP | REGULATION OF OSSIFICATION | 106 | -0.406071397 | -1.762868991 | 0.00012745 | 0.001491659 | 0.001041948 | 4360 | tags=42%, list=21%, signal=34% | TMEM119/S1PR1/TOB2/TGFB2/RFLNB/ACVR2B/BMP2/BMP6/EGR2/ATRAID/TAC1/BMP4/ADGRV1/TFAP2A/ENPP1/DDR2/DHRS3/ATP2B1/ANO6/DKK1/TWIST1/OMD/P2RX7/GPM6B/LRP6/GREM1/PBX1/MGP/BMPR1A/PTN/MEF2C/SLC8A1/BMPR1B/SMAD3/RBPJ/MAPK1/BMP7/OSR1/ZBTB16/NBR1/ANKH/LRP4/BCL2/OSR2/ADRB2 |
| GOBP | REGULATION OF SUPRAMOLECULAR FIBER ORGANIZATION | 352 | -0.291936717 | -1.48116398 | 0.000125514 | 0.001471564 | 0.001027911 | 3843 | tags=31%, list=18%, signal=26% | ARHGEF10L/APC/ELN/TRIOBP/DMTN/CAMSAP2/PIK3CA/PRKN/EFEMP2/TACSTD2/ARHGEF2/RDX/NRP1/PRKCE/TAC1/ASAP3/PXN/DLG1/WASHC1/APP/ABL1/CCN2/TENM1/WASF2/RGCC/TMSB4X/CXCL12/AEBP1/SWAP70/ATXN7/CLASP1/S100A10/EDN1/CHADL/PRUNE1/SLIT2/ARHGEF15/KANK4/RAPGEF3/ADD1/MET/FMN1/TMOD2/WASF3/FGF13/ALMS1/FER/PLEKHH2/TMSB15B/SHANK3/TESK1/BMERB1/DSTN/APOE/BIN1/VILL/DYRK1A/SORBS3/ARHGAP35/PFN2/MECP2/CLIP3/SPTAN1/NUMA1/ARHGEF5/RICTOR/MAP2/SNCA/CAMSAP3/SLAIN2/AKAP13/ROCK2/ARHGAP6/TTBK2/SCIN/SMAD3/FERMT2/FHOD3/SVIL/ABI2/SDC4/CFL2/MYOC/AKAP9/PAK3/MAP1A/NIN/TSC1/LPAR1/EPS8/SPTBN2/LIMCH1/CORO2B/PFDN5/MYADM/PIK3R1/ADD3/LMOD1/DLC1/DBN1/SYNPO2/TPM1/TMOD1/CGNL1/GSN/ARHGEF10/KANK1/SPTBN1/CRYAB/MAP1B |
| GOMF | METAL CLUSTER BINDING | 62 | 0.511252836 | 1.883999323 | 0.00012537 | 0.001471564 | 0.001027911 | 4690 | tags=47%, list=22%, signal=36% | GLRX3/CIAPIN1/KIF4A/UQCRFS1/RSAD2/NDUFS7/SDHB/NUBP1/NDUFV2/BRIP1/XDH/ACO2/NDUFS8/DNA2/CISD3/GLRX2/NDUFV1/FECH/NUBP2/CISD1/NDUFS2/FXN/CIAO3/NDUFS1/POLD1/CISD2/ETFDH/FDX2/NTHL1 |
| GOMF | PROTON CHANNEL ACTIVITY | 20 | 0.700624536 | 2.024947761 | 0.000124928 | 0.001469821 | 0.001026694 | 3460 | tags=65%, list=17%, signal=54% | ATP5MF/ATP5PF/ATP5F1B/ATP5PB/ATP5ME/ATP5MG/ATP5F1C/SLC4A11/ATP5PO/ATP6V1A/ATP5F1D/ATP5F1A/ATP5PD |
| GOBP | PHARYNGEAL SYSTEM DEVELOPMENT | 23 | -0.642247839 | -2.026924505 | 0.000123601 | 0.001456752 | 0.001017565 | 4040 | tags=48%, list=19%, signal=39% | TBX1/TGFB2/ADGRF5/BMP4/HES1/NOG/BMPR1A/BMP7/PTCH1/PLXNA2/GATA3 |
| GOCC | CONDENSED NUCLEAR CHROMOSOME | 87 | 0.459755915 | 1.78556114 | 0.000123263 | 0.001455329 | 0.001016571 | 4365 | tags=41%, list=21%, signal=33% | CCNB1/NDC80/H2AX/BUB1B/NEK2/BUB1/TUBG1/HUS1/RAD51/NUF2/CENPK/CENPA/SHOC1/PLK1/RGS12/BLM/NOL6/BRCA2/RCC1/NIFK/BRCA1/PMF1/MIS12/PELI1/RRS1/SYCE3/UBE2I/CENPO/RAD50/REC8/CHMP1A/LRPPRC/KIFAP3/FKBP6/SMC1A/HORMAD1 |
| GOMF | TRANSFORMING GROWTH FACTOR BETA ACTIVATED RECEPTOR ACTIVITY | 12 | -0.785505021 | -2.081267616 | 0.00011428 | 0.001351637 | 0.00094414 | 1785 | tags=50%, list=9%, signal=46% | ACVRL1/LTBP4/BMPR1A/BMPR1B/TGFBR2/TGFBR3 |
| GOBP | CARDIAC CHAMBER MORPHOGENESIS | 114 | -0.388496189 | -1.705559717 | 0.00011356 | 0.001345496 | 0.000939851 | 4733 | tags=37%, list=23%, signal=29% | SEMA3C/WNT11/HEY1/TEK/MED1/TGFB2/BMP2/SAV1/SOS1/FOXC2/NRP1/PPP1R13L/BMP4/ROBO2/HES1/JAG1/TBX3/DHRS3/SOX4/SHOX2/SLIT2/NOG/CPE/PARVA/NPY5R/SLIT3/DSP/FOXC1/FGFR2/BMPR1A/MEF2C/RBP4/WNT2/RBPJ/TGFBR2/BMP7/ZFPM2/TNNC1/TPM1/NOTCH2/TGFBR3/GATA3 |
| GOCC | ACTOMYOSIN | 71 | -0.451729336 | -1.824489543 | 0.000109069 | 0.001294568 | 0.000904277 | 4751 | tags=49%, list=23%, signal=38% | PDLIM2/ACTN4/CDC42BPB/SYNPO/HDAC4/TEK/SHROOM4/DAAM1/ILK/PXN/FAM107A/PDLIM5/ABLIM1/MYL9/ROR1/ACTA1/PTK2/SEPTIN7/SEPTIN11/PDLIM3/FERMT2/MYH14/MYLK/CDC42BPA/ABLIM3/LIMCH1/LPP/MYH10/NEBL/DBN1/SYNPO2/TPM1/PDLIM4/PGM5/SORBS1 |
| GOBP | CELL FATE COMMITMENT | 240 | -0.323584261 | -1.561082401 | 0.000107289 | 0.001275689 | 0.000891089 | 3139 | tags=23%, list=15%, signal=20% | ERBB4/OLIG1/BMP4/WNT7B/HES1/JAG1/TBX3/LMO4/BCL11B/SOX13/MGA/NTF4/SOX8/EBF2/DKK1/JAG2/FGF13/PPARG/FZD7/TM2D3/FGFR2/HOXC10/CDC73/KLF4/BMPR1A/NR2F2/GDF7/ROR2/DOCK7/MEF2C/HOXA11/SATB2/WNT2/SMAD5/TGFB1I1/RTF1/RBPJ/GATA2/ZNF521/PTCH1/MITF/TBX15/CYP26B1/GAS1/CDON/TCF7L2/GLI2/AR/NOTCH2/GLI3/SPRY2/BCL2/SOX6/WNT2B/SOX5/GATA3 |
| GOBP | RESPIRATORY SYSTEM DEVELOPMENT | 185 | -0.33397216 | -1.577214312 | 0.000106967 | 0.00127412 | 0.000889994 | 3320 | tags=31%, list=16%, signal=27% | MAN1A2/FSTL3/FGF1/BMP4/KCNAB1/WNT7B/CCN2/HES1/PDGFRB/LOX/ASXL1/YAP1/NOG/SRSF6/SP3/PDGFA/FGF9/EGFR/LIF/PGR/SPEF2/TULP3/LRP6/FGFR2/SEC24B/KLF2/BMPR1A/ATXN1L/GPC3/PTN/NPHP3/RBP4/IGFBP5/THRA/MYOCD/DAG1/ALDH1A2/SKI/WNT2/NFIB/RBPJ/MAPK1/FBXW7/TGFBR2/WDPCP/DLG5/CCBE1/ZFPM2/ABCA3/PKD1/LAMA5/PDGFRA/SPRY1/GLI2/GLI3/SPRY2/HSD11B1/WNT2B |
| GOBP | NUCLEOBASE CONTAINING SMALL MOLECULE BIOSYNTHETIC PROCESS | 109 | 0.441728085 | 1.78720014 | 0.000106702 | 0.001273226 | 0.000889369 | 1988 | tags=28%, list=10%, signal=26% | GDA/TYMP/UPP1/TK1/PNP/UCK2/NME1/ACOT7/NUDT15/VCP/ENTPD7/DTYMK/APRT/TDG/IMPDH1/NT5C2/HPRT1/PUDP/AMPD3/ITPA/CNP/NUDT5/XDH/DNPH1/GPX1/PDE8A/ADK/UMPS/DCTPP1/SMUG1/NUDT1 |
| GOBP | CD4 POSITIVE ALPHA BETA T CELL ACTIVATION | 92 | 0.464294542 | 1.81799654 | 0.000105221 | 0.00125778 | 0.00087858 | 3986 | tags=40%, list=19%, signal=33% | ZC3H12A/CD274/NFKBIZ/IL4R/PRKCQ/CD83/ENTPD7/STOML2/RSAD2/BCL3/STAT3/CTSL/SASH3/JAK3/IFNG/RELB/SOCS1/CBFB/IL12B/IL6/LGALS9/CCL19/IL23A/BATF/GPR183/CD86/LEF1/SEMA4A/NFKBID/XCL1/ANXA1/HLA-DRA/IL12RB1/NLRP3/MTOR/CD80/FOXP3 |
| GOBP | NEGATIVE REGULATION OF FAT CELL DIFFERENTIATION | 45 | -0.540083389 | -2.00790434 | 0.000104471 | 0.001251048 | 0.000873877 | 4368 | tags=60%, list=21%, signal=48% | CCDC85B/INSIG1/CCN4/GPER1/FOXO1/SIRT1/ENPP1/JAG1/ASXL1/JDP2/WWTR1/YAP1/LRP3/ADIPOQ/ARNTL/TRIO/DDIT3/SMAD3/FERMT2/TGFB1I1/ZADH2/ZFPM2/GATA2/RUNX1T1/ZFP36L2/GATA3/RORA |
| GOBP | LYMPHOCYTE MIGRATION | 104 | 0.447819595 | 1.79549825 | 0.000103496 | 0.001241579 | 0.000867263 | 4245 | tags=44%, list=20%, signal=35% | C10orf99/WNT5A/CCL20/S100A7/PYCARD/CCR7/CXCL13/CYP7B1/CXCL16/CXCL10/CCL18/CCL4/CCL22/ADAM17/CH25H/CCL2/SPNS2/CCL8/ITGA4/RIPK3/CCL7/CCL19/GPR183/DOCK8/OXSR1/ADAM10/CXCL11/ITGAL/ADAM8/FADD/WASL/PIK3CD/EXT1/CRTAM/CCL5/XCL1/ICAM1/CCR2/CCL17/GCSAM/TMEM102/ZAP70/MSN/GPR15/CKLF/MYO1G |
| GOMF | PATTERN RECOGNITION RECEPTOR ACTIVITY | 23 | 0.671943257 | 1.994350294 | 9.88E-05 | 0.001187725 | 0.000829645 | 3029 | tags=57%, list=15%, signal=48% | NOD2/CLEC7A/PTAFR/PGLYRP4/TLR2/FCN1/CLEC4E/CD36/PGLYRP2/TRIM5/LY96/TLR8/PGLYRP3 |
| GOBP | MORPHOGENESIS OF A BRANCHING STRUCTURE | 183 | -0.34933018 | -1.634385635 | 9.81E-05 | 0.001180943 | 0.000824907 | 4763 | tags=39%, list=23%, signal=31% | FAT4/WNT6/SEMA3C/DDR1/CTSH/SEMA3A/MED1/LRP5L/KDR/CTNNB1/LRP5/BMP2/TACSTD2/BTRC/FOXC2/NRP1/LGR4/PLXND1/FGF1/SETD2/CLIC4/DLG1/ABL1/FEM1B/BMP4/AGT/TBX3/RSPO3/SOX10/LRRK2/NTN4/BTBD7/SHOX2/SEMA3E/YAP1/EDN1/SLIT2/NOG/SOX8/MET/ESR1/PDGFA/EGF/PGR/LRP6/FGFR2/GREM1/PBX1/GDF7/PKD2/GPC3/HOXA11/DAG1/WNT2/RTN4/TGFBR2/BMP7/RERE/FOXD1/DLG5/PTCH1/DCHS1/PKD1/LAMA5/SPRY1/CTNNBIP1/GLI2/AR/GLI3/SPRY2/BCL2/WNT2B |
| GOBP | TRANSLESION SYNTHESIS | 42 | 0.58604361 | 2.000396617 | 9.38E-05 | 0.001131354 | 0.000790269 | 4808 | tags=64%, list=23%, signal=50% | PCLAF/UBE2L6/POLE2/PCNA/VCP/ISG15/RPA3/UFD1/DTL/POLD2/RFC2/RFC3/POLDIP2/MAD2L2/USP43/FAAP20/POLD4/RFC4/TRIM25/POLD3/NPLOC4/PARP10/RPA1/POLD1/SPRTN/RFC5/USP10 |
| GOBP | REGULATION OF TYPE I INTERFERON MEDIATED SIGNALING PATHWAY | 34 | 0.604559241 | 1.984654011 | 9.35E-05 | 0.001129515 | 0.000788984 | 3538 | tags=47%, list=17%, signal=39% | IRF7/WNT5A/MMP12/PTPN2/IKBKE/USP18/ABCE1/TBK1/TTLL12/ADAR/NLRC5/FADD/CDC37/IFNAR2/YTHDF2/SAMHD1 |
| GOBP | INTERLEUKIN 8 PRODUCTION | 82 | 0.468194185 | 1.799977718 | 9.32E-05 | 0.00112868 | 0.000788401 | 4406 | tags=45%, list=21%, signal=36% | WNT5A/NOD2/CLEC7A/MYD88/AFAP1L2/PYCARD/BCL3/TLR2/STAT3/CD2/LAMTOR5/IL1B/NOS2/RAB1A/FCN1/SYK/IL6/LGALS9/PTPN22/FFAR2/CD58/FADD/PARK7/TLR8/TNF/DDX58/ANXA1/ARRB1/BCL10/CD244/LILRA2/SERPINE1/IL10/IL17F/C5AR2/NLRP10/PTPRC |
| GOBP | SIGNAL TRANSDUCTION INVOLVED IN CELL CYCLE CHECKPOINT | 74 | 0.49201579 | 1.85495565 | 9.18E-05 | 0.00111283 | 0.000777329 | 3325 | tags=41%, list=16%, signal=34% | CCNB1/CDK1/TFDP1/PCNA/AURKA/ZWILCH/PLK2/SFN/E2F8/DTL/CNOT11/CDKN1A/PLK1/PLK3/CNOT1/PRKDC/CHEK2/BRCC3/BABAM2/TRIAP1/BRCA1/CDK2/CRADD/CDC25C/PML/BAX/GADD45A/PRMT1/MDM2/CNOT6 |
| GOBP | NEGATIVE REGULATION OF SUPRAMOLECULAR FIBER ORGANIZATION | 152 | -0.36442626 | -1.662109798 | 9.05E-05 | 0.001099971 | 0.000768348 | 3828 | tags=37%, list=18%, signal=30% | APC/TRIOBP/DMTN/CAMSAP2/PIK3CA/PRKN/TACSTD2/ARHGEF2/RDX/WASF2/TMSB4X/SWAP70/ATXN7/CLASP1/CHADL/SLIT2/KANK4/ADD1/MET/TMOD2/FGF13/PLEKHH2/TMSB15B/SHANK3/BMERB1/APOE/VILL/DYRK1A/PFN2/CLIP3/SPTAN1/MAP2/SNCA/CAMSAP3/ARHGAP6/TTBK2/SCIN/FHOD3/SVIL/MYOC/EPS8/SPTBN2/CORO2B/PFDN5/MYADM/PIK3R1/ADD3/LMOD1/DLC1/TMOD1/CGNL1/GSN/KANK1/SPTBN1/CRYAB/MAP1B |
| GOCC | PROTEASOME CORE COMPLEX BETA SUBUNIT COMPLEX | 10 | 0.829768265 | 2.003816408 | 9.03E-05 | 0.001099105 | 0.000767742 | 3025 | tags=90%, list=14%, signal=77% | PSMB2/PSMB5/PSMB6/PSMB10/PSMB3/PSMB8/PSMB9/PSMB1/PSMB7 |
| GOBP | POSITIVE REGULATION OF MITOTIC CELL CYCLE | 106 | 0.442845044 | 1.785183467 | 8.91E-05 | 0.001086385 | 0.000758858 | 3972 | tags=43%, list=19%, signal=35% | CCNB1/DLGAP5/CDK1/TFDP1/EIF4EBP1/UBE2C/CDCA5/EIF4E/RAB11A/CDC6/CDC25B/ADAM17/CDC25A/DTL/ANAPC7/KLHL18/CDT1/ESPL1/LSM10/TTL/POLDIP2/BRCA2/RCC2/SMARCD3/CDC7/CCND2/FBXO5/MAD2L1BP/TMOD3/ANAPC11/MTA3/PLRG1/CDC25C/RB1/MDM2/EIF4G1/ANXA1/CDC23/PHB2/ASNS/USP2/CCND3/LGMN/CDK4/SPHK1/UBE2E2 |
| GOBP | REGULATION OF CYTOSKELETON ORGANIZATION | 491 | -0.266324398 | -1.394046928 | 8.88E-05 | 0.001084057 | 0.000757231 | 3156 | tags=26%, list=15%, signal=23% | CLIC4/PXN/DLG1/WASHC1/ABL1/CCN2/CEP120/PDGFRB/TENM1/WASF2/RGCC/BCAS3/TMSB4X/CXCL12/TPR/FAM107A/EFNA5/SEMA3E/SWAP70/BST1/ATXN7/CLASP1/CENATAC/S100A10/EDN1/SYDE1/PRUNE1/SLIT2/ARHGEF15/MAP6/TRAF3IP1/KANK4/RHOBTB1/FZD10/RAPGEF3/RHOQ/ADD1/NEXN/MET/CHMP4B/FMN1/PDGFA/TMOD2/WASF3/FGF13/ALMS1/FER/P2RX7/PLEKHH2/TMSB15B/SHANK3/TESK1/BMERB1/GPM6B/DSTN/BIN1/VILL/DYRK1A/SORBS3/PTK2/ARHGAP35/NTF3/PAM/PFN2/MECP2/CLIP3/SPTAN1/NUMA1/RHOB/PRKAA2/STMN3/ARHGEF5/GSK3B/RICTOR/PHLDB1/MAP2/SNCA/CAMSAP3/SLAIN2/AKAP13/IQGAP1/ROCK2/ARHGAP6/TTBK2/SCIN/SMAD3/FERMT2/FHOD3/SVIL/ABI2/MAPK1/SDC4/CFL2/MYOC/AKAP9/PAK3/MAP1A/NIN/TSC1/PKD1/LPAR1/EPS8/SPTBN2/LIMCH1/CORO2B/DIXDC1/GNAI1/MYADM/PIK3R1/ADD3/PDGFRA/LMOD1/DLC1/DBN1/JAM3/SYNPO2/TPM1/TMOD1/NOTCH2/CGNL1/GSN/CCSAP/ARHGEF10/KANK1/RHOBTB3/SPTBN1/TPPP/MAP1B/CCL27 |
| GOBP | REGULATION OF LEUKOCYTE MEDIATED CYTOTOXICITY | 73 | 0.486684357 | 1.835743503 | 8.56E-05 | 0.001047551 | 0.000731731 | 5048 | tags=56%, list=24%, signal=43% | SERPINB4/DNASE1L3/CYRIB/ARG1/NOS2/RAET1E/IL12B/SERPINB9/RASGRP1/IL18RAP/IL7R/RIPK3/LGALS9/SH2D1A/IL23A/LAG3/CXCL6/FADD/HLA-E/NCR3/CRTAM/XCL1/KLRD1/CD1B/HLA-DRA/ICAM1/IL12RB1/TYROBP/B2M/HLA-F/HLA-B/LILRB1/NECTIN2/HLA-G/VAV1/CD1D/PTPRC/AGER/ARRB2/HLA-A/SLAMF6 |
| GOBP | ESTABLISHMENT OF TISSUE POLARITY | 121 | 0.428900902 | 1.764966214 | 8.55E-05 | 0.001047551 | 0.000731731 | 2469 | tags=33%, list=12%, signal=29% | PSME2/PSMB2/WNT5A/PSMA5/PSMB5/FZD5/AP2S1/PSMA3/PSMD12/PSME4/PSMA2/PSME1/GRHL3/PSMB6/PSMB10/PSMB3/PSMD6/PSMB8/PSMC4/PSMA4/PSMD2/PSMD11/PSMD1/PSMD14/PSMC3/PSMB9/PSMA7/PSMD9/PSMD8/PSMA1/PSMD13/PSMF1/PSMC5/PSMC2/PSMB1/PSMC6/PSME3/PSMD7/PSMC1/TP63 |
| GOBP | MRNA EXPORT FROM NUCLEUS | 105 | 0.442277638 | 1.775813692 | 8.32E-05 | 0.001021654 | 0.000713642 | 4158 | tags=45%, list=20%, signal=36% | EIF4A3/DDX39A/EIF4E/HNRNPA2B1/NCBP1/SRSF9/NUP37/NUP210/NUP88/CPSF3/ALYREF/MAGOHB/NUP50/SARNP/NSUN2/SRSF2/NXT1/POLR2D/RAE1/SRSF7/SEC13/SRSF1/THOC5/IWS1/NUP155/THOC6/THOC7/NDC1/CPSF4/GLE1/MAGOH/NUP188/DDX19A/WDR33/NUP85/FYTTD1/CHTOP/NUP205/EIF5A/AGFG1/UPF1/ENY2/NUP58/NUP107/NUP93/SRSF3/POM121 |
| GOCC | PIGMENT GRANULE | 105 | 0.443510094 | 1.780762193 | 8.07E-05 | 0.000992666 | 0.000693393 | 4573 | tags=45%, list=22%, signal=35% | RAB38/RAN/MREG/AHCY/GGH/DNAJC5/CTSB/ATP6V1B2/RAB27A/ATP1B3/HSPA5/PRDX1/SLC2A1/CNP/RAB5A/RAB1A/P4HB/HSPA8/GPR143/MYO5A/SYNGR1/SLC3A2/CTNS/CTSD/PDIA4/PMEL/TFRC/SND1/RPN1/PDIA6/PDCD6IP/YWHAZ/CANX/ATP1A1/PPIB/TYRP1/CAPG/MLANA/STX3/TMEM33/SLC1A5/SLC1A4/RAB29/MYO7A/TPP1/TYR/SLC45A2 |
| GOBP | TELOMERASE RNA LOCALIZATION | 19 | 0.722713657 | 2.075806166 | 8.05E-05 | 0.000991851 | 0.000692824 | 2945 | tags=68%, list=14%, signal=59% | RUVBL1/CCT5/RUVBL2/NHP2/CCT2/CCT3/NOP10/CCT7/TCP1/SHQ1/DKC1/CCT6A/CCT8 |
| GOBP | RESPONSE TO RADIATION | 434 | 0.318520417 | 1.486043279 | 7.99E-05 | 0.000987319 | 0.000689658 | 4917 | tags=36%, list=24%, signal=28% | PCLAF/SERPINB13/IFI16/SLC7A11/H2AX/PBK/COPS3/TIGAR/AURKB/IVL/NABP1/PCNA/RUVBL2/POLB/BAK1/HIF1A/NMT1/MAPK13/FKBPL/HUS1/RAD51/METAP1/EIF2S1/MMP9/HSPA5/CXCL10/FEN1/BCL3/ECT2/CDC25A/NDRG4/COPS9/ZMPSTE24/DTL/RGS14/RAD51AP1/AEN/PPP1CA/MYC/PNKP/FIGNL1/CDK5/DNMT3B/MMP1/IL12B/CDKN1A/XRCC6/FANCD2/GPX1/SOD2/UBE2A/RRM1/PLK3/BLM/ST20/XRCC5/CCL7/BRCA2/FECH/CTNS/SMPD1/RNF168/THBD/METAP2/NR2F6/NOC2L/HRAS/PRKDC/CARD16/NPM1/RAD54L/CHEK2/BRCC3/FANCG/ATF4/BABAM2/TXN/RAD18/XRCC4/TRIAP1/INTS7/LIG4/EXT1/SWI5/BRCA1/NIPBL/FBXO4/MEN1/PITPNM1/RAD54B/FBXL6/TMEM161A/ERCC8/PML/BAX/ERCC1/GADD45A/PARP1/MDM2/RNF8/RFWD3/ANXA1/ELOVL4/ICAM1/CASP3/KRT14/PRKCD/OPN3/POLD3/BHLHE40/ASNS/USP2/BACE1/KDM1A/DCUN1D3/LZIC/ROM1/MAPK8/NABP2/CDKN2D/GNGT2/CYP2R1/DNMT3A/MTOR/MAPK14/ELK1/ATR/HYAL3/VCAM1/TIPIN/TLK2/ABCA7/SMC1A/HRH1/POLD1/MMP3/TRPM1/PDE1B/NEDD4/PTPRC/DNM2/TANK/RIC8A/TYR/NMU/CASP9/BRSK1/SPRTN/USP28/GRB2/HMGCS1/GRK4/RHNO1/OPN5/FNTA/POLG |
| GOCC | PRERIBOSOME SMALL SUBUNIT PRECURSOR | 15 | 0.755600416 | 2.043777161 | 7.93E-05 | 0.000981516 | 0.000685605 | 3370 | tags=60%, list=16%, signal=50% | TSR1/BYSL/RRP1/NOC4L/NOP14/FAM207A/RIOK1/NOB1/RIOK2 |
| GOBP | TRANSFORMING GROWTH FACTOR BETA RECEPTOR SIGNALING PATHWAY | 187 | -0.348838122 | -1.649685697 | 7.89E-05 | 0.000978351 | 0.000683394 | 5601 | tags=45%, list=27%, signal=34% | SMAD2/GDF15/RNF111/CIDEA/RHOA/NLK/FBN1/GDF10/COL1A2/FLCN/EP300/NRROS/SMAD4/CDH5/STUB1/LATS1/BAMBI/GDF9/LEMD3/RASL11B/PEG10/PDPK1/HTRA1/USP9X/UBC/LRRC32/ITGB8/APPL1/TGFB2/SMURF1/LTBP2/LDLRAD4/MSTN/SLC2A10/GCNT2/TGFBRAP1/CGN/HTRA4/PXN/SIRT1/SPRED2/PTPRK/DAB2/LOX/GLG1/SNW1/CD109/SPRED1/FUT8/FOS/TRIM33/LATS2/CLDN5/ACVRL1/SNX25/CREBBP/DKK3/LTBP4/PTK2/HPGD/SMURF2/PMEPA1/BMPR1A/PPARA/ZMIZ1/SDCBP/SMAD9/MYOCD/SKI/SMAD5/SMAD3/FERMT2/TGFB1I1/TGFBR2/ASPN/CDKN1C/PARD3/IL17RD/ITGB5/SPRY1/ZNF703/SPRY2/CAV2/CAV1/TGFBR3 |
| GOBP | RIBOSE PHOSPHATE BIOSYNTHETIC PROCESS | 186 | 0.384201731 | 1.655271824 | 7.66E-05 | 0.000950912 | 0.000664228 | 4401 | tags=38%, list=21%, signal=30% | ENO1/ATP5MF/UPP1/UCK2/AK2/NME1/ACOT7/CTPS1/PYGL/VCP/ATP5MC3/ATP5PF/GART/ATP5F1B/STOML2/ATP5MC1/APRT/ATIC/IMPDH1/HPRT1/ATP5PB/ALDOA/SLC25A13/ELOVL7/AMPD3/ADSL/ATP5ME/COASY/AK1/ATP5MG/ATP5F1C/ADK/UMPS/PPT2/UQCC3/HACD1/GSTZ1/ATP5PO/ATP6V1A/GMPS/ANTKMT/ACACA/PDHB/ADCY3/GCDH/TREM2/PPCDC/PKM/ATP5F1D/PARP1/PRPSAP2/G6PD/ATP5F1A/ELOVL4/DLAT/PAPSS2/DLD/ATP5PD/RFK/ELOVL1/PANK2/VPS9D1/NME7/CAD/PRPS2/TKT/DNAJC30/PDHA1/ELOVL6/SLC25A12/PDP2 |
| GOCC | 90S PRERIBOSOME | 27 | 0.655060661 | 2.035496015 | 7.65E-05 | 0.000950912 | 0.000664228 | 3641 | tags=59%, list=17%, signal=49% | RRP36/WDR12/IMP4/NOL6/UTP4/NOC4L/BOP1/NOP14/FAM207A/NOC2L/UTP6/PES1/RRP7A/TBL3/WDR3/HEATR1 |
| GOBP | CD4 POSITIVE ALPHA BETA T CELL DIFFERENTIATION | 78 | 0.483009363 | 1.840442392 | 7.64E-05 | 0.000950912 | 0.000664228 | 3986 | tags=42%, list=19%, signal=34% | ZC3H12A/NFKBIZ/IL4R/CD83/ENTPD7/RSAD2/BCL3/STAT3/CTSL/SASH3/JAK3/IFNG/RELB/SOCS1/CBFB/IL12B/IL6/LGALS9/CCL19/IL23A/BATF/GPR183/CD86/LEF1/SEMA4A/NFKBID/ANXA1/HLA-DRA/IL12RB1/NLRP3/MTOR/CD80/FOXP3 |
| GOBP | RIBONUCLEOSIDE TRIPHOSPHATE BIOSYNTHETIC PROCESS | 65 | 0.511293328 | 1.900256759 | 7.60E-05 | 0.000949339 | 0.000663129 | 4092 | tags=49%, list=20%, signal=40% | ENO1/ATP5MF/UCK2/NME1/CTPS1/VCP/ATP5MC3/ATP5PF/ATP5F1B/STOML2/ATP5MC1/IMPDH1/ATP5PB/ALDOA/SLC25A13/ATP5ME/ATP5MG/ATP5F1C/UQCC3/ATP5PO/ATP6V1A/ANTKMT/TREM2/PKM/ATP5F1D/PARP1/ATP5F1A/ATP5PD/VPS9D1/NME7/CAD/DNAJC30 |
| GOBP | DNA STRAND ELONGATION INVOLVED IN DNA REPLICATION | 19 | 0.723775924 | 2.07885725 | 7.57E-05 | 0.000947064 | 0.00066154 | 3406 | tags=68%, list=16%, signal=57% | GINS3/PCNA/GINS2/MCM4/GINS1/POLE3/POLD2/MCM7/MCM3/DNA2/RFC3/RFC4/POLD3 |
| GOMF | THREONINE TYPE ENDOPEPTIDASE ACTIVITY | 13 | 0.79004115 | 2.046909203 | 7.51E-05 | 0.000940963 | 0.000657278 | 3025 | tags=77%, list=14%, signal=66% | PSMB2/PSMB5/PSMB6/PSMB10/PSMB3/PSMB8/PSMA4/PSMB9/PSMB1/PSMB7 |
| GOBP | RESPONSE TO OXIDATIVE STRESS | 421 | 0.320428747 | 1.489979601 | 7.31E-05 | 0.000917386 | 0.000640809 | 3743 | tags=30%, list=18%, signal=25% | GJB2/ZC3H12A/CYCS/MELK/PSMB5/PPIF/ATOX1/HMOX2/SLC7A11/GCH1/GPX2/S100A7/CCR7/NUDT15/PCNA/MACROH2A1/SRXN1/GSTP1/VRK2/BAK1/HIF1A/EZH2/MAPK13/NCOA7/CCNA2/SLC23A2/EIF2S1/NDUFB4/MMP9/SMPD3/PPARGC1B/CFLAR/VNN1/PPIA/ERO1A/ATP2A2/ADPRS/TXNRD1/ECT2/PNPT1/LDHA/ARG1/RGS14/FOSL1/MEAK7/PRDX1/TRAP1/CHCHD2/DUOX1/NDUFA6/PNKP/P4HB/PDCD10/ROMO1/HMOX1/FANCD2/GPX1/PDE8A/SOD2/NDUFS8/NAPRT/IL18RAP/NR4A3/IL6/CYP2E1/ENDOG/PLK3/RIPK3/SESN2/CD36/GCLM/GLRX2/CCL19/GSKIP/TOR1A/ATG7/DHFR/NUDT1/OXSR1/FKBP1B/NCF2/BNIP3/ATF4/TXN/PRDX5/TXN2/PARK7/CYBA/IPCEF1/HNRNPM/GPR37/KEAP1/LONP1/HDAC2/DIABLO/ADAM9/CDK2/MAP3K5/TMEM161A/ERCC8/PML/MICB/NDUFS2/FXN/PPP1R15B/ERCC1/PARP1/PRDX6/G6PD/HTRA2/APTX/CHUK/ANXA1/MTF1/SIGMAR1/CASP3/STX4/PRKCD/GCLC/CD38/BECN1/NDUFA12/MCL1/PXDN/MAPK8/PRDX4/UCP2/TRA2B |
| GOBP | NCRNA TRANSCRIPTION | 107 | 0.44608869 | 1.795709034 | 6.93E-05 | 0.000871616 | 0.000608838 | 4534 | tags=45%, list=22%, signal=35% | GTF3C6/NABP1/MACROH2A1/IPPK/POLR2H/ELL2/NCBP1/POLR2F/MARS1/SIRT7/PIH1D1/ZC3H8/INTS13/GTF2A2/POLR2L/GTF2E2/TAF13/POLR2G/NPM3/POLR2D/POLR1B/NIFK/ELL/INTS7/MACROH2A2/CDK7/POLR2E/POLR1G/GTF2E1/INTS5/SMARCA4/TAF11/INTS9/CDK9/NOL11/GTF2B/INTS14/POLR2I/NABP2/MTOR/GTF3C2/RPRD1B/CCNK/PWP1/POLR2J/TAF5/TAF1B/NCL |
| GOCC | INTRINSIC COMPONENT OF ORGANELLE MEMBRANE | 388 | 0.329264521 | 1.519775104 | 6.82E-05 | 0.000859023 | 0.000600042 | 5271 | tags=41%, list=25%, signal=32% | MREG/SFXN1/SCO2/RER1/GHITM/MFN1/SLC35B1/DNAJC5/MCUB/ATP10B/BAK1/DHRS9/DPAGT1/HSPA5/HM13/TIMM17A/TAP1/SPPL2A/TIMM23/ABCB6/MTX2/HSPA9/TMEM70/ZMPSTE24/MICOS10/TMEM177/TAP2/ELOVL7/STX6/BCAP31/TOMM40/YIF1A/COA1/YIF1B/RAB5A/SCO1/PEX3/SFXN4/BID/MICOS13/TMCO1/COA3/EMC6/CCDC51/UBXN8/MTX1/CHCHD3/SFXN2/COQ2/IMMT/MTLN/EDEM1/SYNGR1/EMC3/CYP2E1/EMC8/PREB/UQCC3/PCSK7/DERL2/EMC9/TMEM163/HACD1/NPC1/PET100/GDAP1/AUP1/SEC61A1/CPLX3/SLC37A4/BNIP3/HLA-E/VAMP7/RCE1/QSOX1/DOLK/FURIN/SLC37A2/TAPBP/SAMD8/SLC25A19/EXT1/L2HGDH/DPM2/CANX/SREBF2/SLC35A4/LEMD2/LBR/SFXN5/MFF/COX16/EMC1/SLC38A9/CPT1C/SLC66A1/DOLPP1/ELOVL4/SLC35B4/PEX13/HLA-DRA/ESYT3/AGK/PEX2/FUNDC2/EMC7/ANKLE2/STX10/ZDHHC20/ABCB10/APOO/TIMM17B/B2M/HLA-F/DNAJC11/ELOVL1/TMEM201/HLA-B/G6PC1/P2RX4/SLC25A3/PIGU/HLA-G/B4GALNT2/ACER3/GRAMD2A/ABHD17B/TMEM33/SAMM50/DNAJB12/BNIP1/ELOVL6/SLC35A2/CHST2/RNF152/ETFDH/MCUR1/GPAA1/ELOVL2/EMC4/OXA1L/CHCHD10/PDIA3/SLC15A4/PORCN/HLA-DQB1/SELENOS/CLN3/AFG3L2/MUL1/HLA-A/EMC10/TM7SF2/DERL1/ADCK1/TRABD2A/MPC2/HLA-DPB1/SLC8B1/HLA-C/PEX11G |
| GOBP | OSSIFICATION | 373 | -0.294081274 | -1.502181723 | 6.75E-05 | 0.000852301 | 0.000595346 | 4814 | tags=37%, list=23%, signal=29% | PKDCC/NF1/CTHRC1/TWIST2/FAT4/FASN/WNT11/CDK6/KREMEN2/COL6A1/HDAC4/MN1/TOB1/TEK/TMEM119/S1PR1/LRP5L/CRIM1/TOB2/SNRNP200/IGSF10/CCN4/TGFB2/RFLNB/RSL1D1/LRP5/ACVR2B/BMP2/MMP2/BMP6/EGR2/INPPL1/TAPT1/BPNT2/ATRAID/FOXC2/ATP6V0A4/TNN/LGR4/VEGFC/FGFR3/CREB3L1/TAC1/FSTL3/ATP6V1B1/BMP4/RORB/WNT7B/ADGRV1/CCN2/GNAS/RPS15/MMP16/PTH1R/PRKD1/TFAP2A/ASF1A/ENPP1/LOX/FZD1/SLC26A2/JAG1/THBS3/DDR2/DHRS3/ZBTB40/WWTR1/SHOX2/ITGA11/YAP1/NPR2/LEP/ATP2B1/LRP3/NOG/IL6ST/ANO6/SOX8/DKK1/SP3/TWIST1/OMD/JUND/PPARG/EGFR/KLF10/P2RX7/KAZALD1/GPM6B/MRC2/BCAP29/PTK2/LRP6/FOXC1/FGFR2/GREM1/PBX1/MGP/BMPR1A/ROR2/GPC3/PTN/MEF2C/SLC8A1/IGFBP5/THRA/MEF2D/CHRDL1/SATB2/SKI/SMAD5/BMPR1B/SMAD3/FERMT2/ZHX3/RBPJ/MAPK1/BMP7/MYOC/ASPN/OSR1/RPL38/PTCH1/GDPD2/DCHS1/ZBTB16/NAB1/CTNNBIP1/GLI2/NBR1/ANKH/LRP4/GLI3/TUFT1/BCL2/OSR2/CAT/AXIN2/ADRB2 |
| GOCC | CONDENSED CHROMOSOME OUTER KINETOCHORE | 14 | 0.777345511 | 2.053809638 | 6.75E-05 | 0.000852301 | 0.000595346 | 1507 | tags=64%, list=7%, signal=60% | CCNB1/NDC80/BUB1B/BUB1/CENPF/SKA3/SKA2/SKA1/PLK1 |
| GOBP | SIGNAL TRANSDUCTION BY P53 CLASS MEDIATOR | 257 | 0.355211175 | 1.588544656 | 6.62E-05 | 0.000839169 | 0.000586173 | 4898 | tags=41%, list=23%, signal=32% | CCNB1/IFI16/CDK1/TFDP1/FOXM1/AURKB/PYCARD/HDAC1/PCNA/AURKA/TPX2/SMYD2/HUS1/RPA3/PLK2/BCL3/PRMT5/SFN/E2F8/ZMPSTE24/NOP2/AEN/EXO1/EEF1E1/MIF/BRIP1/CNOT11/CDK5/TAF10/CDKN1A/BCL2L12/RFC2/CSNK2A1/DNA2/MYBBP1A/PLK3/BLM/SESN2/RFC3/PRMT6/BRCA2/BOP1/BATF/RRP8/WWOX/CNOT1/ARMC10/NOC2L/TAF13/RPF2/CHEK2/E2F2/BDKRB2/BOK/TP63/TRIAP1/HIPK2/BRCA1/HDAC2/CDK2/CRADD/MEAF6/CDC25C/PML/BAX/ING2/TAF11/GADD45A/PRMT1/TOP3A/MDM2/SP100/PSMD10/RFC4/CNOT6/SHISA5/RRS1/PRKAG1/PAK1IP1/KDM1A/RMI2/SSRP1/RAD50/MAPK14/ATR/MYO6/MAPKAPK5/RPA1/TAF5/HINT1/HNRNPK/CSNK2B/MBD3/SETD9/PMAIP1/USP28/RFC5/TAF2/CNOT10/YJU2/USP10/POU4F2/RHNO1/RBBP4/PYHIN1 |
| GOBP | NEGATIVE REGULATION OF LEUKOCYTE MEDIATED IMMUNITY | 52 | 0.547740264 | 1.963176164 | 6.61E-05 | 0.000839169 | 0.000586173 | 5251 | tags=62%, list=25%, signal=46% | SERPINB4/NOD2/SUSD4/ARG1/JAK3/HMOX1/SERPINB9/IL7R/LGALS9/LILRB4/HLA-E/BST2/XCL1/KLRD1/CLEC4G/CD96/CCR2/HLA-F/HLA-B/LILRB1/HLA-G/CD300A/FOXP3/PARP3/CR1/PTPRC/FOXF1/TBX21/ARRB2/IFNA2/HLA-A/C4BPA |
| GOBP | REGULATION OF CELL KILLING | 92 | 0.472275083 | 1.849245232 | 6.51E-05 | 0.000828557 | 0.00057876 | 3933 | tags=46%, list=19%, signal=37% | SERPINB4/KRT6A/CLEC7A/DNASE1L3/CYRIB/ARG1/PRF1/IFNG/NOS2/RAET1E/IL12B/SERPINB9/RASGRP1/SYK/IL18RAP/IL7R/RIPK3/LGALS9/SH2D1A/IL23A/LAG3/CXCL6/CASP8/FADD/HLA-E/NCR3/CRTAM/XCL1/KLRD1/CD1B/HLA-DRA/ICAM1/IL12RB1/TYROBP/B2M/HLA-F/CR1L/HLA-B/LILRB1/NECTIN2/HLA-G/VAV1 |
| GOBP | INOSITOL LIPID MEDIATED SIGNALING | 187 | -0.350817086 | -1.659044385 | 6.49E-05 | 0.000826752 | 0.000577499 | 3812 | tags=34%, list=18%, signal=28% | PIP4K2A/PREX2/ANGPT1/GPER1/PLD1/PIK3CA/UBE3A/PTEN/PIP5K1B/ERBB3/IL18/ERBB4/SIRT1/PDGFRB/AGT/PLCH2/PEAR1/INSR/LEP/EDN1/PLCB1/F2R/IGF1R/ROR1/TNFAIP8L3/TWIST1/PDGFA/EGF/EGFR/OGT/PTK2/PLCL2/NTF3/PIK3CB/PIK3IP1/NTRK2/ZFP36L1/FN1/KLF4/FGFR1/PDGFD/ROR2/DIPK2A/PLCB4/MAPK1/PIP4K2B/MYOC/DCN/FYN/PLEKHA1/PPP2R5C/IRS1/PIK3C2G/PIK3R1/PDGFRA/KIT/GSN/GAB2/IRS2/CAT/SERPINA12/NOP53/PDGFC/GATA3 |
| GOBP | ENDOCARDIAL CUSHION DEVELOPMENT | 42 | -0.566920089 | -2.075117057 | 6.38E-05 | 0.000815339 | 0.000569527 | 5071 | tags=50%, list=24%, signal=38% | RBM24/SMAD4/HEY1/MSX1/TMEM100/CRELD1/TGFB2/BMP2/ERBB3/BMP4/ROBO2/JAG1/NOG/TWIST1/ACVRL1/BMPR1A/RBPJ/MDM4/TGFBR2/BMP7/DCHS1 |
| GOBP | NEGATIVE REGULATION OF CELL SUBSTRATE ADHESION | 60 | -0.495076073 | -1.924678852 | 6.36E-05 | 0.000813397 | 0.000568171 | 4702 | tags=52%, list=23%, signal=40% | ACTN4/MELTF/LRP1/PHLDB2/ACER2/DMTN/TACSTD2/PTEN/GCNT2/SPOCK1/BCAS3/JAG1/FAM107A/EFNA5/SEMA3E/APOD/ACVRL1/FZD7/LGALS1/ARHGAP6/FBLN1/PTPRO/NEXMIF/MYOC/FZD4/BCL6/CORO2B/PIK3R1/DLC1/POSTN/KANK1 |
| GOBP | DICARBOXYLIC ACID METABOLIC PROCESS | 92 | 0.472787057 | 1.851249922 | 6.26E-05 | 0.000802142 | 0.000560309 | 3758 | tags=40%, list=18%, signal=33% | KYNU/GOT2/SLC7A11/GOT1/GPT2/SHMT2/MDH2/ATIC/MTHFD1/MTHFD2/HAL/SDHB/ALDH4A1/IDH2/MTRR/NAGS/MRPS36/GLUL/NIT2/FH/GCLM/PCK2/MTHFD1L/DHFR/MDH1/SDHAF3/L2HGDH/ME1/SLC19A1/KYAT3/ALDH5A1/GCLC/DLD/ME2/GAD1/SLC25A32/MTHFR |
| GOMF | THREONINE TYPE PEPTIDASE ACTIVITY | 20 | 0.714993027 | 2.066475628 | 6.12E-05 | 0.000786038 | 0.00054906 | 3526 | tags=55%, list=17%, signal=46% | PSMB2/PSMB5/PSMB6/PSMB10/PSMB3/PSMB8/PSMA4/PSMB9/PSMB1/PSMB7/BACE1 |
| GOBP | REGULATION OF MITOCHONDRIAL GENE EXPRESSION | 29 | 0.655735311 | 2.057718377 | 6.10E-05 | 0.000785132 | 0.000548428 | 4705 | tags=72%, list=23%, signal=56% | C1QBP/TSFM/SHMT2/TACO1/FASTKD2/TRUB2/MTG1/TRMT10C/COA3/UQCC2/MPV17L2/MRPS27/RCC1L/METTL4/MTG2/FASTKD3/LRPPRC/UQCC1/MALSU1/RPUSD3/CHCHD10 |
| GOBP | HOMOPHILIC CELL ADHESION VIA PLASMA MEMBRANE ADHESION MOLECULES | 149 | -0.373562235 | -1.699245742 | 6.04E-05 | 0.000778502 | 0.000543796 | 5268 | tags=40%, list=25%, signal=30% | PCDHB3/CDH6/PCDH8/AMIGO2/PCDHGA10/PCDHB6/CDH5/PCDHGA3/FAT4/DSC1/TRO/PCDHB12/PTPN23/PCDHB13/OBSL1/PCDH12/CDHR3/AMIGO1/PCDHB11/CADM1/ROBO3/NECTIN3/DSG1/IGSF11/ROBO2/FAT3/PCDH9/CDH10/PCDHB7/PCDHB14/PECAM1/NPTN/PTPRM/L1CAM/CLSTN1/MYOT/PCDHB2/PCDHB10/PCDH19/PCDH18/PCDHB4/NEXN/PCDHB5/ROBO4/CNTN4/CADM3/DSG2/ITGB1/CDH12/CDH19/CLSTN2/PCDH20/PCDHGA8/DCHS1/PKD1/PCDHB16/PCDHGA4/PCDH7/CDHR1 |
| GOBP | SMOOTH MUSCLE CONTRACTION | 95 | -0.428922418 | -1.834357805 | 6.01E-05 | 0.000776917 | 0.000542689 | 2762 | tags=35%, list=13%, signal=30% | GPER1/EDN3/SETD3/DLG1/TBX3/RGS2/ATP2B1/EDN1/F2R/DOCK5/DOCK4/TACR1/ARHGAP42/SLC8A1/P2RX1/ORMDL3/MYOCD/KCNMA1/ROCK2/ADRA1A/PRKG1/ACTA2/ATP2B4/MYLK/ATP1A2/ADRA2A/CNN1/MYH11/BBS2/KIT/CAV1/SOD1/ADRB2 |
| GOBP | T CELL MEDIATED IMMUNITY | 97 | 0.463659936 | 1.835911567 | 5.92E-05 | 0.000765796 | 0.000534921 | 5020 | tags=55%, list=24%, signal=42% | NOD2/FZD5/CTSC/RAB27A/RSAD2/CYRIB/HPRT1/ARG1/SASH3/PRF1/IL1B/IL12B/IL7R/IL6/RIPK3/IL23A/FADD/LILRB4/HLA-E/TNFRSF1B/HSPD1/CD8A/XCL1/KLRD1/CD1B/CLEC4G/HLA-DRA/ICAM1/IL12RB1/DENND1B/CCR2/B2M/HLA-F/WAS/HLA-B/LILRB1/BTN3A3/NECTIN2/NLRP3/HLA-G/FOXP3/TRAF2/FBXO38/EMP2/MYO1G/CD1D/PTPRC/MALT1/AGER/AIRE/TBX21/IFNA2/HLA-A |
| GOBP | ACTIN MEDIATED CELL CONTRACTION | 115 | -0.400874209 | -1.756307531 | 5.91E-05 | 0.000765796 | 0.000534921 | 2151 | tags=26%, list=10%, signal=24% | CACNB2/ACTC1/PARVA/FGF13/ACTA1/MYH3/EPDR1/TNNC2/DSG2/ANK2/DSP/BIN1/TNNT1/PLN/AKAP9/TPM2/SCN3B/TNNC1/ATP1A2/VIM/DES/TNNI2/LIMCH1/GPD1L/CACNA2D1/DMD/TPM1/TMOD1/GSN/CAV1 |
| GOBP | NEGATIVE REGULATION OF NEURON PROJECTION DEVELOPMENT | 128 | -0.390749021 | -1.740067882 | 5.82E-05 | 0.00075575 | 0.000527904 | 4765 | tags=42%, list=23%, signal=33% | SEMA4C/SEMA3C/INPP5F/LRP1/SEMA3A/CERS2/PTPN9/LRIG2/RUFY3/UBE3A/SEMA6D/PTEN/NRP1/SPOCK1/EFNB3/BAG5/ADCY6/NR2F1/MYLIP/RTN4R/LRRK2/ZNF365/SEMA3E/NGEF/DENND5A/SPART/SEMA4G/NLGN1/TSKU/SEMA6A/EFNA1/FGF13/SEMA3B/RYK/APOE/PTPRS/LGALS1/TRAK2/ITM2C/MAP2/NTN1/PTPRO/RTN4/CBFA2T2/TSC1/RTN4RL1/SEMA3G/LPAR1/VIM/RGMA/DPYSL3/LRP4/EFNB2/KANK1 |
| GOCC | SPLICEOSOMAL TRI SNRNP COMPLEX | 32 | 0.617833755 | 1.994511602 | 5.80E-05 | 0.000754779 | 0.000527225 | 2761 | tags=53%, list=13%, signal=46% | SNRPG/SNRPF/SNRPD1/LSM2/TXNL4A/LSM7/SNRPD3/EFTUD2/SNU13/PPIH/LSM5/LSM4/SNRPB/PRPF31/USP39/LSM6/PRPF4 |
| GOBP | CELL DIFFERENTIATION INVOLVED IN KIDNEY DEVELOPMENT | 58 | -0.508304454 | -1.988301059 | 5.75E-05 | 0.000750142 | 0.000523986 | 3538 | tags=48%, list=17%, signal=40% | FOXC2/LGR4/BMP4/HES1/JAG1/ASXL1/WWTR1/YAP1/ADIPOQ/LIF/GLIS2/MTSS1/PODXL/GREM1/POU3F3/LAMB2/MEF2C/IQGAP1/PTPRO/CD34/ACTA2/OSR1/PTCH1/KLF15/MAGI2/NOTCH2/GLI3/GATA3 |
| GOBP | ACTIN FILAMENT BUNDLE ORGANIZATION | 150 | -0.383601411 | -1.748199957 | 5.66E-05 | 0.000739252 | 0.000516379 | 4675 | tags=41%, list=22%, signal=32% | WNT11/SYNPO/PAK2/SHTN1/PHLDB2/S1PR1/FRMD7/RFLNB/PHACTR1/ARHGEF10L/FAM171A1/ELN/DMTN/PRKN/TACSTD2/RDX/NRP1/TAC1/ASAP3/PXN/ABL1/MICAL1/CCN2/WASF2/RGCC/FAM107A/SWAP70/CLASP1/S100A10/ARHGEF15/RAPGEF3/ADD1/MET/NEDD9/ALMS1/SHANK3/TESK1/SORBS3/PFN2/ARHGEF5/CALD1/AIF1L/ROCK2/ARHGAP6/SMAD3/FERMT2/SDC4/MYOC/TSC1/LPAR1/EPS8/LIMCH1/CORO2B/PIK3R1/ITGB5/DLC1/DPYSL3/SYNPO2/TPM1/CGNL1/SORBS1/ARHGEF10 |
| GOBP | REGULATION OF MUSCLE CELL DIFFERENTIATION | 128 | -0.391402247 | -1.742976802 | 5.64E-05 | 0.000738027 | 0.000515523 | 5524 | tags=48%, list=26%, signal=36% | CAMK1/MYLK3/PAK1/DLL1/IGF1/RBM24/SMAD4/TRIM32/TBX1/MAMSTR/PDGFB/AKAP6/HDAC4/PI16/MSX1/TMEM119/CTNNB1/KAT2A/HDAC9/HDAC5/PLPP7/ANKRD17/BMP2/PARP2/GPER1/EFEMP2/SETD3/SIRT1/ABL1/BMP4/RBM4/RGS2/SHOX2/EDN1/CCN3/DKK1/FGF9/CTNNA1/FZD7/NFATC2/FGFR2/GREM1/MECP2/PPARA/MEF2C/MYOCD/NLN/RBPMS2/PDCD4/CYP26B1/NFATC1/BHLHE41/PRDM6/BNIP2/CDON/PIAS1/KIT/BCL2/SOX6/EFNB2/MEF2A/BOC |
| GOBP | REGULATION OF BMP SIGNALING PATHWAY | 85 | -0.448769078 | -1.874834908 | 5.55E-05 | 0.000728077 | 0.000508574 | 6342 | tags=58%, list=30%, signal=40% | CCN1/SOX11/CTDSPL2/SMAD7/HOXA13/TMPRSS6/SFRP4/SMAD2/SFRP1/SFRP2/FBN1/SMAD4/CDH5/BAMBI/LEMD3/HTRA1/MSX1/TOB1/CRIM1/SMURF1/SORL1/FSTL1/SFRP5/FSTL3/ABL1/BMP4/HES1/NOG/SPART/TRIM33/PPARG/NBL1/ACVRL1/GREM1/SMURF2/NUMA1/GPC3/NEO1/CHRDL1/SKI/RBPMS2/RBPJ/FOXD1/TFAP2B/ELAPOR2/ZNF423/FST/NOTCH2/GREM2 |
| GOBP | MATURATION OF 5 8S RRNA | 35 | 0.610486256 | 2.02173204 | 5.47E-05 | 0.000719587 | 0.000502643 | 3999 | tags=51%, list=19%, signal=42% | MPHOSPH6/EXOSC4/WDR12/EIF6/EXOSC3/RRP15/BOP1/NOP14/RPP40/PRKDC/EXOSC2/ERI1/PES1/ERI2/ABT1/RRS1/EXOSC8/NOL9 |
| GOBP | DNA SYNTHESIS INVOLVED IN DNA REPAIR | 53 | 0.546355854 | 1.967028343 | 5.45E-05 | 0.000717468 | 0.000501163 | 4808 | tags=55%, list=23%, signal=42% | PCLAF/UBE2L6/POLE2/PCNA/VCP/ISG15/RPA3/UFD1/DTL/POLD2/RFC2/RFC3/POLDIP2/MAD2L2/USP43/FAAP20/POLD4/RFC4/TRIM25/POLD3/NPLOC4/CDKN2D/PARP10/RPA1/POLD1/WRNIP1/SPRTN/RFC5/USP10 |
| GOBP | CARDIAC MUSCLE CELL DIFFERENTIATION | 95 | -0.429579029 | -1.837165911 | 5.43E-05 | 0.000717074 | 0.000500888 | 4119 | tags=37%, list=20%, signal=30% | OBSL1/KAT2A/BMP2/PARP2/CBY1/BMP4/PDGFRB/TBX3/BVES/RGS2/PDLIM5/SHOX2/EDN1/DKK1/ACTC1/FZD7/CACYBP/GREM1/SORBS2/PPARA/MEF2C/SLC8A1/MYOCD/AKAP13/ADRA1A/FHOD3/RBPJ/TSC1/SGCB/NEBL/PDGFRA/MYH11/SOX6/EFNB2/MEF2A |
| GOBP | POSITIVE REGULATION OF STRESS FIBER ASSEMBLY | 52 | -0.527142361 | -2.008607158 | 5.36E-05 | 0.000708872 | 0.000495158 | 3843 | tags=46%, list=18%, signal=38% | ARHGEF10L/NRP1/TAC1/PXN/ABL1/CCN2/RGCC/S100A10/ARHGEF15/RAPGEF3/TESK1/SORBS3/PFN2/ARHGEF5/ROCK2/SMAD3/FERMT2/SDC4/MYOC/TSC1/LPAR1/LIMCH1/TPM1/ARHGEF10 |
| GOBP | PROTEIN TRANSMEMBRANE IMPORT INTO INTRACELLULAR ORGANELLE | 33 | 0.630247187 | 2.048826747 | 5.31E-05 | 0.000703365 | 0.000491312 | 3472 | tags=58%, list=17%, signal=48% | GRPEL1/TIMM50/TIMM17A/TIMM23/TOMM40L/AIFM1/TOMM40/CHCHD4/ROMO1/DNAJC15/PEX10/TIMM44/TIMM21/HSPD1/GRPEL2/DNLZ/PEX13/PEX2/TIMM17B |
| GOBP | RNA PHOSPHODIESTER BOND HYDROLYSIS ENDONUCLEOLYTIC | 74 | 0.500533207 | 1.887067283 | 5.17E-05 | 0.000687029 | 0.000479901 | 5169 | tags=51%, list=25%, signal=39% | ZC3H12A/TSR1/RNASEH2A/FEN1/EXO1/CPSF3/RIDA/ELAC2/ENDOU/ZC3H12C/ZC3H12D/ENDOG/BOP1/NOP14/RPP40/POP5/SND1/ABT1/POP4/TSEN34/POP1/NOB1/TBL3/KHNYN/MRPL44/RRS1/SLFN13/POP7/RPP30/NOL9/RPP38/EXOG/TSEN2/ENDOV/BMS1/APEX1/NUDT16L1/RNASET2 |
| GOBP | EMBRYONIC ORGAN MORPHOGENESIS | 268 | -0.320348484 | -1.564928878 | 5.03E-05 | 0.000668779 | 0.000467153 | 3538 | tags=28%, list=17%, signal=24% | FOXC2/MIB1/MAFB/IFT172/IFT52/SETD2/HOXD9/MFAP2/DLG1/ATP6V1B1/BMP4/HES1/GNAS/MMP16/TFAP2A/HOXB5/AHI1/TBX3/SHOX2/YAP1/EDN1/DYNC2I1/NOG/HOXB6/DVL2/FZD3/HOXC4/HOXB2/SP3/EFNA1/TWIST1/MFAP5/PCGF2/TULP3/HOXD4/LRP6/FGFR2/SEC24B/PKD2/SOBP/ROR2/FRZB/NPHP3/MEF2C/RBP4/HOXA11/NTN1/TSHZ1/SATB2/RBPMS2/SMAD3/IRX5/LRIG1/MAPK1/LRIG3/TGFBR2/WDPCP/BMP7/HOXB3/GATA2/OSR1/RPL38/TBX15/WDR19/EFEMP1/PDGFRA/SLITRK6/GLI2/HOXC9/NOTCH2/GLI3/SPRY2/OSR2/SOD1/GATA3 |
| GOBP | CHRONIC INFLAMMATORY RESPONSE | 19 | 0.732899599 | 2.105062624 | 5.00E-05 | 0.000666376 | 0.000465474 | 4213 | tags=74%, list=20%, signal=59% | S100A9/S100A8/AHCY/CXCL13/IDO1/VNN1/CCL5/TNF/PTGES/UNC13D/GJA1/IL10/FOXP3/VCAM1 |
| GOBP | NUCLEOSIDE TRIPHOSPHATE BIOSYNTHETIC PROCESS | 76 | 0.507815571 | 1.926759206 | 4.92E-05 | 0.000657298 | 0.000459133 | 3869 | tags=47%, list=19%, signal=39% | ENO1/ATP5MF/UCK2/NME1/CTPS1/VCP/ATP5MC3/TYMS/CMPK2/ATP5PF/ATP5F1B/STOML2/ATP5MC1/DTYMK/IMPDH1/ATP5PB/ALDOA/SLC25A13/ATP5ME/AK1/ATP5MG/ATP5F1C/ADK/UQCC3/ATP5PO/ATP6V1A/ANTKMT/TREM2/PKM/ATP5F1D/PARP1/ATP5F1A/ATP5PD/VPS9D1/NME7/CAD |
| GOBP | REGULATION OF ACTOMYOSIN STRUCTURE ORGANIZATION | 93 | -0.43830912 | -1.879501707 | 4.91E-05 | 0.000656576 | 0.000458629 | 4675 | tags=55%, list=22%, signal=43% | CCDC88A/ARAP1/BAG4/NOX4/SFRP1/RHOA/MYLK3/PAK1/SMAD4/WNT11/PAK2/PHLDB2/S1PR1/FRMD7/ARHGEF10L/TACSTD2/NRP1/TAC1/ASAP3/PXN/ABL1/CCN2/WASF2/RGCC/CLASP1/S100A10/EDN1/ARHGEF15/RAPGEF3/MET/ALMS1/TESK1/SORBS3/PFN2/ARHGEF5/AKAP13/ROCK2/ARHGAP6/SMAD3/FERMT2/SDC4/MYOC/TSC1/LPAR1/LIMCH1/CORO2B/PIK3R1/DLC1/TPM1/CGNL1/ARHGEF10 |
| GOBP | REGULATION OF NEURON PROJECTION DEVELOPMENT | 414 | -0.286383006 | -1.47006258 | 4.82E-05 | 0.000646568 | 0.000451638 | 4656 | tags=34%, list=22%, signal=27% | FBXO7/MAPT/SCN1B/DAB2IP/CUX2/ABL2/PAK2/INPP5F/SHTN1/LRP1/ALKAL2/SEMA3A/DVL1/ARHGAP44/CERS2/RND2/PTPN9/SF3A2/TRPC6/ULK4/FRMD7/OBSL1/FUT9/LRIG2/TNIK/RUFY3/SERPINF1/EFHC2/AMIGO1/PRRX1/LRRC4C/NCKIPSD/SERPINI1/SS18L1/CAMSAP2/FEZ1/UBE3A/SEMA6D/PTEN/NEDD4L/NRP1/SPOCK1/PLXND1/RAP1A/EFNB3/RELN/TIAM2/BAG5/CNTN1/MFSD2A/ANKRD27/ADCY6/NR2F1/ROBO2/SARM1/PRKD1/CXCL12/MYLIP/HECW2/FZD1/CAMK1D/RTN4R/LRRK2/RGS2/PDLIM5/ZNF365/SHOX2/SEMA3E/GOLGA4/NPTN/NGEF/MACF1/L1CAM/SLIT2/DENND5A/BHLHB9/SPART/SEMA4G/NLGN1/ROR1/MBOAT1/MAP6/TSKU/SEMA6A/EFNA1/ZDHHC15/FGF13/SEMA3B/SHANK3/RYK/APOE/PTPRS/ARHGAP35/CAMK2G/LGALS1/NTRK2/FN1/KLF4/CAPRIN2/TRAK2/GSK3B/PTPRF/ROR2/CREB3L2/PTN/ITM2C/MAP2/NEGR1/NTN1/IQGAP1/SCARB2/PTPRO/RTN4/ABI2/KIDINS220/NDNF/BMP7/RAPGEF2/PAK3/CBFA2T2/NIN/TSC1/RTN4RL1/PLXNA2/SEMA3G/LPAR1/FYN/VIM/RGMA/MAGI2/SDC2/DBN1/DPYSL3/UST/LRP4/FBXO31/EFNB2/KANK1/MAP1B/EEF2K/COBL/GATA3 |
| GOBP | URETER DEVELOPMENT | 18 | -0.716913428 | -2.082166831 | 4.69E-05 | 0.000630499 | 0.000440414 | 3069 | tags=50%, list=15%, signal=43% | BMP4/SOX8/NPHP3/ALDH1A2/LZTS2/OSR1/NFIA/EMX2/GATA3 |
| GOBP | REGULATION OF CELL MATRIX ADHESION | 120 | -0.401464137 | -1.769240073 | 4.61E-05 | 0.000621191 | 0.000433912 | 4798 | tags=46%, list=23%, signal=35% | NF1/CCL28/CDK6/DDR1/LRP1/TEK/PHLDB2/ACER2/KDR/DMTN/EFEMP2/PTEN/NRP1/VCL/ABL1/BCAS3/JAG1/FAM107A/EFNA5/SEMA3E/BST1/CLASP1/S100A10/MACF1/DUSP3/APOD/FMN1/RRAS/ACVRL1/GPM6B/PTK2/PIK3CB/GREM1/GSK3B/COL16A1/CAMSAP3/DAG1/IQGAP1/ROCK2/ARHGAP6/SMAD3/FERMT2/SDC4/WDPCP/NEXMIF/MYOC/BCL6/TSC1/LIMCH1/PIK3R1/PEAK1/DLC1/BCL2/POSTN/UTRN |
| GOBP | REGULATION OF LEUKOCYTE PROLIFERATION | 229 | 0.374322898 | 1.652123636 | 4.60E-05 | 0.000620821 | 0.000433654 | 4807 | tags=41%, list=23%, signal=32% | CD24/CD274/PNP/SLC7A1/MYD88/TNFRSF21/PRKCQ/PYCARD/GSTP1/IDO1/IRF1/PLA2G2F/ARG1/SASH3/CEBPB/JAK3/MIF/IL1B/LYN/CD6/CORO1A/IL12B/CDKN1A/CCL8/GLMN/SYK/IKZF3/RAC2/IL6/RIPK3/LGALS9/CCL19/PTPN22/IL23A/GPR183/IL2RA/TFRC/HLA-DMB/FADD/LILRB4/HLA-E/TNFRSF1B/TNFSF9/CD86/CRTAM/TMEM131L/SCRIB/BST2/SFTPD/CCL5/DNAJA3/EBI3/XCL1/CD3E/CLEC4G/ANXA1/PELI1/IL12RB1/CASP3/TYROBP/CCR2/CD38/TNFRSF13B/LILRB2/IL15/TNFRSF4/CARD11/ZAP70/MNDA/LILRB1/RASAL3/TNFSF13B/MZB1/HLA-G/CD300A/CD80/IL10/FOXP3/EPO/VCAM1/NCK2/LST1/CR1/CCDC88B/CD1D/CLECL1/PTPRC/CD28/IL33/AIF1/AGER/CD40/MAPK3/CRP/TICAM1 |
| GOBP | NUCLEAR EXPORT | 192 | 0.39662425 | 1.717987729 | 4.55E-05 | 0.00061561 | 0.000430013 | 4158 | tags=40%, list=20%, signal=32% | RAN/IFI27/EIF4A3/DDX39A/EIF4E/EIF6/HNRNPA2B1/NCBP1/SRSF9/NUP37/SFN/HSPA9/NUP210/CPSF6/NUP88/NUP62CL/IL1B/SIRT7/BAG3/CPSF3/ALYREF/CDK5/MAGOHB/ABCE1/NUP50/XPOT/NOL6/SARNP/NSUN2/SRSF2/NXT1/XPO6/CSE1L/RANGAP1/NPM1/POLR2D/RAE1/XPO5/SRSF7/SEC13/SRSF1/TXN/PARK7/THOC5/IWS1/NRDE2/NUP155/THOC6/THOC7/NDC1/SP100/CPSF4/GLE1/MAGOH/NUP188/DDX19A/RRS1/RIOK2/WDR33/NUP85/UHMK1/FYTTD1/CHTOP/NUP205/NUTF2/EIF5A/AGFG1/UPF1/ENY2/RITA1/NUP58/EMD/NUP107/NUP93/SRSF3/POM121 |
| GOBP | DNA PACKAGING | 212 | 0.379802049 | 1.666238476 | 4.53E-05 | 0.00061302 | 0.000428204 | 4684 | tags=38%, list=22%, signal=30% | CCNB1/RUVBL1/H2AX/NUSAP1/CDCA5/PSME4/NCAPH/NCAPG/CENPN/HDAC1/MACROH2A1/KNL1/CENPW/CENPX/MCM2/ASF1B/TOP2A/OIP5/HJURP/CENPM/DNMT1/CENPK/CENPA/NAA10/AIFM1/NCAPG2/IPO4/SMC2/POLE3/SMC4/CENPS/HAT1/RRP8/NASP/NOC2L/SRPK1/NPM1/GRWD1/CENPH/CENPI/NAP1L4/HMGB2/H2BC9/MACROH2A2/H3C10/HELLS/NRDE2/PAF1/NCAPD2/MBD2/CDKN2A/MIS18A/SIRT6/SMARCA5/HMGA1/RNF8/BEND3/SPTY2D1/DAXX/CENPL/CHAF1A/NAA60/CENPO/PARP10/CHMP1A/DNMT3A/H2BC10/CENPU/H1-2/RESF1/CHAF1B/H3C7/ZNFX1/H2BC12/H3C8/H4C4/H4C9/H2BC13/SUV39H1/MBD3 |
| GOBP | POSITIVE REGULATION OF ALPHA BETA T CELL ACTIVATION | 62 | 0.529014784 | 1.949453235 | 4.34E-05 | 0.000589679 | 0.0004119 | 4618 | tags=61%, list=22%, signal=48% | NFKBIZ/PNP/IL4R/PRKCQ/CD83/SASH3/AP3D1/IFNG/SOCS1/CBFB/IL12B/HSPH1/SYK/LGALS9/CCL19/AP3B1/PTPN22/IL23A/LILRB4/HLA-E/CD86/EBI3/NFKBID/XCL1/CD3E/ANXA1/HLA-DRA/IL12RB1/CCR2/ZAP70/RASAL3/NLRP3/CD80/FOXP3/PTPRC/CD28/MALT1/NKAP |
| GOBP | REGULATION OF MRNA METABOLIC PROCESS | 324 | 0.345447555 | 1.574770397 | 4.25E-05 | 0.000577469 | 0.000403372 | 3743 | tags=31%, list=18%, signal=26% | ZC3H12A/PSME2/CCNB1/CARHSP1/PSMB2/PSMA5/PSMB5/PSMA3/C1QBP/MYD88/PSMD12/PSME4/PSMA2/PSME1/PSMB6/EXOSC4/PSMB10/PSMB3/PSMD6/PSMB8/PSMC4/PSMA4/HNRNPA2B1/PSMD2/NCBP1/SRSF9/HNRNPC/PNPT1/FASTKD2/CPSF6/PSMD11/PSMD1/FASTKD5/PSMD14/PSMC3/PSMB9/EXOSC3/FASTKD1/RIDA/PSMA7/DIS3/PSMD9/PSMD8/TBRG4/HSPA8/PDE12/PSMA1/EXOSC1/ZC3H12D/PSMD13/PSMF1/PSMC5/PSMC2/LEO1/CNOT1/PSMB1/ZFP36/PSMC6/SRPK1/POLR2G/PSME3/EXOSC2/PSMD7/SERBP1/PKP3/DAZAP1/NPM1/ELAVL1/JMJD6/PSMC1/SRSF7/TARDBP/YWHAZ/EXOSC5/LARP1/IWS1/HNRNPM/PAF1/IGF2BP3/PTBP1/YBX1/PSMB7/PRDX6/EIF4G1/SNRPA/NELFE/CPSF4/PSMD10/MBNL3/NOCT/MAGOH/CDK9/PRKCD/CTR9/YTHDF2/MAPKAPK2/EXOSC8/DAZL/FASTKD3/UPF1/TRA2B |
| GOBP | REGULATION OF WNT SIGNALING PATHWAY PLANAR CELL POLARITY PATHWAY | 15 | -0.755249657 | -2.126230661 | 4.23E-05 | 0.000577006 | 0.000403048 | 3115 | tags=73%, list=15%, signal=62% | ABL1/DAB2/RSPO3/MLLT3/NKD1/DACT1/DKK1/ANKRD6/GPC3/NPHP3/ZNRF3 |
| GOBP | POSITIVE REGULATION OF CYSTEINE TYPE ENDOPEPTIDASE ACTIVITY | 146 | 0.419151179 | 1.758223341 | 4.20E-05 | 0.000573719 | 0.000400752 | 3579 | tags=38%, list=17%, signal=32% | S100A9/CYCS/SOX7/S100A8/IFI16/CLEC7A/FAM162A/PYCARD/VCP/CASP1/BAK1/NDUFA13/CFLAR/TNFSF10/LCK/AIM2/AIFM1/BCAP31/HSPE1/MYC/XDH/BID/CASP10/PDCD5/SYK/PDCD6/ST20/LGALS9/CTSD/TNFRSF10A/BEX3/CASP8/TNFRSF10B/FADD/LAPTM5/BOK/NLRP2/HSPD1/TNF/DIABLO/CRADD/MAP3K5/CARD9/PML/FASLG/BAX/HTRA2/TNFSF15/TRADD/HIP1R/ARRB1/CIDEB/BCL10/NLRC4/PDCD2/LGMN |
| GOBP | REGULATION OF SMALL GTPASE MEDIATED SIGNAL TRANSDUCTION | 315 | -0.311731237 | -1.557506703 | 4.06E-05 | 0.000556047 | 0.000388408 | 3710 | tags=31%, list=18%, signal=26% | SQSTM1/LPAR4/ALS2/SOS1/ARHGEF2/MADD/RDX/NRP1/PPP2CB/IQSEC1/RELN/TIAM2/GNA13/RABL3/SRGAP3/ABL1/SOS2/DENND3/RASA4/PDGFRB/KITLG/SHOC2/ARHGEF1/ARHGAP24/RTN4R/ARHGAP10/RASA3/CYTH3/NET1/ERBIN/AMOT/NGEF/SYDE1/SLIT2/F2R/FBXO8/AUTS2/RHOBTB1/RHOQ/MET/ARHGAP20/A2M/ARHGEF6/TRIO/ARHGAP26/RALGAPB/RHOT1/ARHGAP12/APOE/OGT/ARHGAP35/ITPKB/PIK3CB/ARHGAP42/PLEKHG5/RHOB/STMN3/ARHGEF5/SCAI/RHOJ/STARD13/AKAP13/ARHGAP6/ADRA1A/VAV3/ARHGAP29/ARHGEF12/TIMP2/MYOC/RALGPS1/SRGAP1/BCL6/RALGAPA1/LPAR1/FGD4/EPS8/ITSN1/GARNL3/ARFGEF3/CDON/SPRY1/DLC1/ARHGAP31/FAM13A/MCF2L/ARHGEF7/NOTCH2/SPRY2/ARHGEF28/CGNL1/MYO9A/SRGAP2/ARHGEF10/KANK1/IRS2/KANK2/DENND4C/ARHGEF26 |
| GOBP | REGULATION OF KILLING OF CELLS OF OTHER ORGANISM | 16 | 0.765290936 | 2.119166213 | 4.06E-05 | 0.000556047 | 0.000388408 | 2196 | tags=62%, list=11%, signal=56% | KRT6A/CLEC7A/ARG1/PRF1/IFNG/NOS2/SERPINB9/SYK/CXCL6/CASP8 |
| GOBP | RNA 3 END PROCESSING | 137 | 0.432414853 | 1.794078431 | 4.00E-05 | 0.000549803 | 0.000384046 | 3669 | tags=39%, list=18%, signal=32% | CCNB1/EIF4A3/DDX39A/EXOSC4/NCBP1/SRSF9/PNPT1/CPSF6/EXOSC3/HSD17B10/CPSF3/ALYREF/TRMT10C/MAGOHB/CSTF2/ELAC2/POLR3K/TUT7/SARNP/LEO1/SRSF2/EXOSC2/POLR2D/ERI1/SRSF7/SRSF1/GRSF1/ERI2/USB1/INTS7/DKC1/EXOSC5/THOC5/TRNT1/CLP1/PAF1/THOC6/INTS5/THOC7/SNRPA/NELFE/CPSF4/SSU72/CSTF1/MAGOH/CDK9/WDR33/CTR9/INTS14/CHTOP/EXOSC8/TOE1/CSTF3 |
| GOBP | POSITIVE REGULATION OF KILLING OF CELLS OF OTHER ORGANISM | 10 | 0.84439504 | 2.039138768 | 4.00E-05 | 0.000549803 | 0.000384046 | 1638 | tags=60%, list=8%, signal=55% | CLEC7A/ARG1/PRF1/IFNG/NOS2/SYK |
| GOBP | CRISTAE FORMATION | 34 | 0.619415216 | 2.033423378 | 3.91E-05 | 0.000539461 | 0.000376822 | 5190 | tags=71%, list=25%, signal=53% | ATP5MF/ATP5MC3/ATP5PF/ATP5F1B/ATP5MC1/ATP5PB/ATP5ME/MICOS13/CHCHD3/ATP5MG/ATP5F1C/IMMT/UQCC3/ATP5PO/ATP5F1D/ATP5F1A/APOO/ATP5PD/DNAJC11/SAMM50/CHCHD10/ATP5F1E/AFG3L2/ADCK1 |
| GOBP | CELL CELL ADHESION VIA PLASMA MEMBRANE ADHESION MOLECULES | 251 | -0.329630276 | -1.596522003 | 3.90E-05 | 0.000539434 | 0.000376803 | 5105 | tags=37%, list=24%, signal=28% | PCDH8/AMIGO2/PCDHGA10/PCDHB6/CDH5/PCDHGA3/FAT4/MBP/DSC1/ITGAM/TRO/FGFRL1/PCDHB12/PTPN23/PCDHB13/SCARF2/CLDN15/OBSL1/PCDH12/TGFB2/MPZ/BMP2/CDHR3/AMIGO1/LRRC4C/PCDHB11/CADM1/MAP2K5/ROBO3/NECTIN3/SLITRK2/DSG1/IGSF11/ITGA5/ROBO2/TENM1/CBLN1/CLDN3/FAT3/CD164/CLDN11/PCDH9/CDH10/PCDHB7/PCDHB14/PECAM1/NPTN/PTPRM/L1CAM/WNK1/ADIPOQ/CLSTN1/MYOT/PCDHB2/PCDHB10/PCDH19/NLGN1/PCDH18/PCDHB4/NEXN/PCDHB5/ROBO4/CLDN5/CNTN4/CADM3/DSG2/GPC4/PTPRS/ITGB1/CDH12/GPC6/KLF4/ADGRL3/PTPRF/CDH19/CLSTN2/LRFN5/TGFBR2/PCDH20/PCDHGA8/DCHS1/PKD1/SPARCL1/PCDHB16/PCDHGA4/MYADM/CLDN8/PCDH7/ALCAM/CDHR1/CLDN23/CLDN1 |
| GOBP | NUCLEOSIDE METABOLIC PROCESS | 100 | 0.470102777 | 1.871205834 | 3.88E-05 | 0.000536961 | 0.000375076 | 3901 | tags=37%, list=19%, signal=30% | TYMP/RAN/UPP1/TK1/PNP/UCK2/NME1/NT5C3A/AHCY/MFN1/ENTPD7/ACP3/APOBEC3B/DTYMK/TJP2/APRT/ATIC/IMPDH1/NT5C2/HPRT1/PUDP/XDH/DNPH1/ADK/UMPS/GNAI3/GMPS/ADA2/AMD1/CASK/PRPSAP2/DERA/CARD11/NME7/CAD/GMPR2/PRPS2 |
| GOMF | HEPARIN BINDING | 157 | -0.368109189 | -1.691142398 | 3.83E-05 | 0.000531521 | 0.000371276 | 3940 | tags=37%, list=19%, signal=30% | LIPC/LTBP2/MSTN/EFEMP2/FSTL1/NRP1/ADAMTS5/LGR6/APLP2/FGF1/APP/BMP4/CCN2/SMOC2/TENM1/CTSG/THBS2/RSPO3/THBS3/RTN4R/VEGFB/FGFBP3/SLIT2/CCN3/LXN/PGF/SOD3/FBLN7/FGF9/PCOLCE2/SERPINA5/SLIT3/APOE/PTPRS/FGFR2/LPL/TNXB/FN1/FGFR1/PTPRF/PTN/ECM2/PRELP/CCN5/RPL22/CFH/RSPO1/NDNF/LAMC2/BMP7/PTCH1/PAFAH1B1/RTN4RL1/GPNMB/POSTN/GREM2/TGFBR3/ANG |
| GOBP | REGULATION OF OSTEOBLAST DIFFERENTIATION | 114 | -0.402841304 | -1.768537039 | 3.83E-05 | 0.000531521 | 0.000371276 | 5583 | tags=50%, list=27%, signal=37% | HDAC7/SFRP1/ACVR2A/NELL1/SFRP2/GDF10/IGF1/BAMBI/CTHRC1/TWIST2/CDK6/HDAC4/TOB1/TMEM119/CRIM1/CCN4/LRP5/ACVR2B/BMP2/BMP6/ATRAID/TNN/VEGFC/BMP4/RORB/WNT7B/GNAS/PRKD1/JAG1/DDR2/WWTR1/YAP1/LRP3/NOG/IL6ST/TWIST1/JUND/PPARG/PTK2/FGFR2/GREM1/BMPR1A/MEF2C/IGFBP5/SKI/SMAD5/BMPR1B/SMAD3/FERMT2/ZHX3/BMP7/PTCH1/GDPD2/CTNNBIP1/NBR1/GLI3/AXIN2 |
| GOCC | MCM COMPLEX | 11 | 0.82884911 | 2.056917507 | 3.80E-05 | 0.000529723 | 0.00037002 | 1698 | tags=64%, list=8%, signal=58% | MCM6/MCM4/MCM5/MCM2/MCM7/MCM3/MCMBP |
| GOBP | REGULATION OF APOPTOTIC SIGNALING PATHWAY | 328 | 0.349177052 | 1.590878354 | 3.78E-05 | 0.000528255 | 0.000368994 | 4189 | tags=34%, list=20%, signal=27% | S100A9/ENO1/S100A8/PPIF/SLC25A5/CTSC/PRELID1/GHITM/PYCARD/HDAC1/IFI6/GSTP1/HIF1A/NDUFA13/IL19/MMP9/CFLAR/VNN1/TNFSF10/PPIA/PTPN2/LCK/DDIAS/NDUFS3/VDAC2/BCAP31/TRAP1/PPP1CA/MIF/IL1B/P4HB/FIGNL1/BID/FAIM/HMOX1/BCL2L12/GPX1/SOD2/CSNK2A1/PIH1D1/HYOU1/TRAF1/RIPK3/DEDD2/GCLM/GNAI3/CAAP1/WWOX/TNFRSF12A/ARMC10/TNFRSF10A/NOC2L/SLC9A3R1/PLAUR/TRAF7/PSME3/CASP8/PPP2R1B/TNFRSF10B/FADD/SIAH2/BDKRB2/TPD52L1/LTBR/TMEM14A/BOK/TP63/PTPMT1/TRIAP1/HMGB2/PARK7/BRCA1/TNF/CRADD/TMEM161A/PML/FASLG/RB1/BAX/ING2/FXN/PARP1/HTRA2/MDM2/SP100/PSMD10/TRADD/ICAM1/ITPRIP/NLE1/STX4/PRKCD/BCL10/UNC5B/HTT/GSDME/BECN1/KDM1A/MCL1/CDKN2D/ZC3HC1/SERPINE1/TMC8/QARS1/STYXL1/MAPK7/XBP1/TRAF2/EPO/RBCK1 |
| GOBP | NEGATIVE REGULATION OF TRANSMEMBRANE RECEPTOR PROTEIN SERINE THREONINE KINASE SIGNALING PATHWAY | 122 | -0.401822554 | -1.77411481 | 3.73E-05 | 0.000521389 | 0.000364199 | 5601 | tags=48%, list=27%, signal=35% | SMAD2/CIDEA/SFRP1/SFRP2/FBN1/NRROS/STUB1/BAMBI/LEMD3/RASL11B/PEG10/PDPK1/HTRA1/UBC/TOB1/DACT2/CRIM1/SMURF1/SORL1/LDLRAD4/SLC2A10/FSTL3/HTRA4/SIRT1/ABL1/SPRED2/GLG1/NOG/CCN3/CD109/SPRED1/SPART/DKK1/TRIM33/PPARG/NBL1/SNX25/GREM1/SMURF2/PMEPA1/PPARA/CHRDL1/SKI/RBPMS2/SMAD3/TGFB1I1/CILP/TGFBR2/ASPN/MAGI2/IL17RD/SPRY1/FST/SPRY2/CAV2/CAV1/GREM2/TGFBR3 |
| GOBP | RESPONSE TO BMP | 157 | -0.368541416 | -1.693128104 | 3.69E-05 | 0.0005173 | 0.000361342 | 5056 | tags=38%, list=24%, signal=29% | SMAD4/CDH5/BAMBI/GDF9/LEMD3/HTRA1/USP9X/MSX1/TOB1/TMEM100/CRIM1/TGFB2/SMURF1/SORL1/ACVR2B/BMP2/BMP6/FSTL1/SFRP5/FSTL3/ABL1/BMP4/HES1/NOG/SPART/TRIM33/PPARG/NBL1/ACVRL1/HIVEP1/GREM1/SMURF2/NUMA1/TNMD/BMPR1A/GDF7/ROR2/GPC3/NEO1/SMAD9/CHRDL1/SKI/RBPMS2/SMAD5/BMPR1B/SMAD3/RBPJ/BMP7/FOXD1/TFAP2B/ELAPOR2/RGMB/PDCD4/RGMA/ZNF423/FST/NOTCH2/GREM2/TGFBR3/GATA3 |
| GOBP | REGULATION OF CARTILAGE DEVELOPMENT | 61 | -0.499115235 | -1.951012696 | 3.58E-05 | 0.000503807 | 0.000351917 | 4102 | tags=52%, list=20%, signal=42% | TRPS1/PKDCC/WNT11/CCN4/KAT2A/RFLNB/BMP2/BMP6/TAPT1/BMP4/CCN2/GLG1/SHOX2/LEP/CHADL/NOG/LNPK/ACVRL1/GREM1/FRZB/HOXA11/SCIN/BMPR1B/MAF/SMAD3/EFEMP1/ZBTB16/GLI2/GLI3/SOX6/AXIN2/SOX5 |
| GOBP | SENSORY ORGAN MORPHOGENESIS | 237 | -0.335190404 | -1.609964655 | 3.58E-05 | 0.000503807 | 0.000351917 | 3430 | tags=28%, list=16%, signal=24% | NECTIN3/MAFB/IFT172/COL8A1/MFAP2/MFSD2A/ATP6V1B1/BMP4/RORB/TFAP2A/JAG1/AHI1/PTPRM/DIO3/EDN1/NKD1/NOG/DVL2/SOX8/FZD3/TSKU/SP3/TWIST1/MFAP5/LRP6/FGFR2/SEC24B/NTRK2/COL8A2/SOBP/ROR2/PTN/FRZB/RBP4/NTN1/TSHZ1/ZHX2/SKI/WNT2/ABI2/LRIG1/MAPK1/IFT122/LRIG3/WDPCP/BMP7/TFAP2B/GATA2/OSR1/RPL38/WDR19/EFEMP1/CYP26B1/CDON/SLITRK6/GLI2/NOTCH2/GLI3/SPRY2/C12orf57/BCL2/OSR2/MEIS1/SOD1/BCAR3/WNT2B/GATA3 |
| GOMF | CHEMOKINE ACTIVITY | 42 | 0.602725271 | 2.057337669 | 3.56E-05 | 0.000502506 | 0.000351008 | 3002 | tags=50%, list=14%, signal=43% | C10orf99/CCL20/CXCL1/CXCL13/CXCL2/CXCL16/CXCL10/CXCL8/CCL18/CCL4/CCL22/CXCL9/CCL2/CCL8/CCL7/CCL19/CXCL3/CXCL6/CXCL11/CCL5/XCL1 |
| GOBP | ESTABLISHMENT OF RNA LOCALIZATION | 188 | 0.385867114 | 1.66518799 | 3.55E-05 | 0.000502326 | 0.000350883 | 4158 | tags=40%, list=20%, signal=33% | RAN/EIF4A3/DDX39A/EIF4E/EIF6/HNRNPA2B1/NCBP1/SRSF9/NUP37/PNPT1/NUP210/CPSF6/NUP88/NUP62CL/MX2/CPSF3/ALYREF/CKAP5/MAGOHB/ABCE1/NUP50/XPOT/NOL6/SARNP/NSUN2/SRSF2/NXT1/ZFP36/TST/CETN2/NPM1/POLR2D/MRPL18/RAE1/NCBP3/MVP/XPO5/SRSF7/SEC13/SRSF1/THOC5/IWS1/NRDE2/NUP155/IGF2BP3/THOC6/THOC7/NDC1/YBX1/CPSF4/GLE1/MAGOH/NUP188/DDX19A/RRS1/RIOK2/WDR33/NUP85/FYTTD1/ARC/CHTOP/NUP205/NUTF2/EIF5A/AGFG1/UPF1/ENY2/NUP58/SIDT1/LRPPRC/NUP107/NUP93/ATR/SRSF3/IGF2BP1/POM121 |
| GOBP | DNA REPLICATION INDEPENDENT NUCLEOSOME ORGANIZATION | 48 | 0.571391088 | 2.019207872 | 3.49E-05 | 0.000494455 | 0.000345385 | 5572 | tags=62%, list=27%, signal=46% | RUVBL1/CENPN/KNL1/CENPW/CENPX/ASF1B/OIP5/HJURP/CENPM/CENPK/CENPA/IPO4/CENPS/HAT1/NASP/NPM1/CENPH/CENPI/MIS18A/SMARCA5/CENPL/CENPO/CENPU/H4C4/H4C9/RBBP4/H4C2/CENPQ/HIRA/H4C5 |
[truncated: 278,314 more chars]
