# Supplementary material for: Exploration of Biomarkers of Psoriasis through Combined Multiomics Analysis
Source: Mediators Inflamm. 2022 Sep 23;2022:7731082. doi: 10.1155/2022/7731082 (PMC9525798; doi:10.1155/2022/7731082)
Supplement: Supplementary Materials — Supplementary Figure 1 The PCA of gene expression in psoriasis lesions and healthy controls in GSE13355 database. Supplementary Figure 2 The PCA and methylation distribution density in psoriasis lesions and healthy controls from the GSE73894 dataset. (A) PCA in GSE73894. (B) Methylation distribution density in GSE73894. Supplementary Table 1 Identification of DEGs in the psoriatic lesions and healthy control group in GSE13355. Supplementary Table 2 GO analysis on 767 DEGs in GSE13355. Supplementary Table 3 KEGG analysis on 767 DEGs in GSE13355. Supplementary Table 4 Identification of hyper-MR-genes. Supplementary Table 5 Identification of hypo-MR-genes. Supplementary Table 6 GO analysis of hyper-MR-genes. Supplementary Table 7 GO analysis of hypo-MR-genes. Supplementary Table 8 KEGG analysis of hyper-MR-genes. Supplementary Table 9 KEGG analysis of hypo-MR-genes. Supplementary Table 10 GO analysis through single-gene GSEA of GJB2. Supplementary Table 11 KEGG analysis through single-gene GSEA of GJB2. [file 7731082.f1.zip › Supplementary Table 11 (1).docx]

| KEGG analysis through single gene GSEA of GJB2 | | | | | | | | | | |
| --- | --- | --- | --- | --- | --- | --- | --- | --- | --- | --- |
| ID | Description | setSize | enrichmentScore | NES | pvalue | p.adjust | qvalues | rank | leading_edge | core_enrichment |
| KEGG_ALZHEIMERS_DISEASE | KEGG_ALZHEIMERS_DISEASE | 159 | 0.535913016 | 2.279221542 | 1.00E-10 | 3.72E-09 | 2.17E-09 | 3701 | tags=50%, list=18%, signal=41% | 54205/27089/1340/7388/518/840/1349/522/4704/506/7386/9377/4710/516/1351/488/374291/4715/29796/10975/810/4701/6868/4716/4709/1537/4706/515/55851/4722/4726/6390/4708/2597/4729/3553/3028/4700/4694/637/1020/4713/823/4712/509/4728/1347/4697/4723/1337/7385/4702/4696/4714/539/102/841/51806/4695/8772/4717/4731/7132/1350/7384/3416/7124/1329/4720/6392/513/4707/498/836/4705/10476/23621/5331/4725 |
| KEGG_HUNTINGTONS_DISEASE | KEGG_HUNTINGTONS_DISEASE | 174 | 0.542180368 | 2.326671212 | 1.00E-10 | 3.72E-09 | 2.17E-09 | 3949 | tags=49%, list=19%, signal=40% | 54205/292/1175/27089/1340/3065/7388/518/1349/5437/522/4704/506/7386/9377/4710/516/1351/374291/4715/5435/29796/10975/4701/4716/4709/1537/4706/515/4722/4726/6390/4708/7417/4729/4700/4694/4713/4712/2876/509/6648/4728/23186/1347/4697/5441/4723/1337/7385/4702/4696/4714/5436/539/841/5433/7416/4695/4717/4731/1350/5434/7384/3066/1329/1211/4720/6392/7419/513/581/4707/7019/498/836/4705/5438/10476/3064/5331/4725/1327/10540/170712 |
| KEGG_OXIDATIVE_PHOSPHORYLATION | KEGG_OXIDATIVE_PHOSPHORYLATION | 122 | 0.67046978 | 2.761604181 | 1.00E-10 | 3.72E-09 | 2.17E-09 | 3154 | tags=61%, list=15%, signal=52% | 479/9551/27089/1340/7388/518/9114/1349/526/522/4704/506/7386/9377/4710/516/1351/9550/533/374291/4715/29796/10975/4701/4716/4709/1537/4706/9296/515/4722/4726/6390/4708/126328/27068/4729/521/5464/4700/4694/4713/4712/10632/509/4728/51382/1347/4697/528/245973/4723/1337/1352/7385/4702/4696/4714/539/51606/523/4695/4717/10063/4731/8992/1350/7384/1329/4720/6392/513/4707/498 |
| KEGG_PARKINSONS_DISEASE | KEGG_PARKINSONS_DISEASE | 118 | 0.61322901 | 2.507291691 | 1.00E-10 | 3.72E-09 | 2.17E-09 | 4183 | tags=64%, list=20%, signal=52% | 54205/292/9246/27089/1340/7388/518/1349/522/4704/506/7332/7386/9377/4710/516/1351/374291/4715/29796/10975/4701/4716/4709/1537/4706/515/4722/4726/6390/4708/7417/4729/4700/4694/4713/4712/509/4728/1347/4697/4723/1337/7385/4702/4696/4714/539/7416/7326/4695/4717/4731/11315/1350/118424/7384/2861/1329/4720/6392/7419/513/4707/27429/498/836/4705/10476/4725/7318/1327/170712/4719/51465/1345 |
| KEGG_PROTEASOME | KEGG_PROTEASOME | 42 | 0.841639274 | 2.872026213 | 1.00E-10 | 3.72E-09 | 2.17E-09 | 2337 | tags=88%, list=11%, signal=78% | 5721/5690/5686/5693/5684/5718/23198/5683/5720/5694/5699/51371/5691/9861/5696/5704/5685/5708/5717/5707/10213/3458/5702/5698/5688/5714/7979/5682/5719/9491/5705/5701/5689/5706/10197/5713/5700 |
| KEGG_RIBOSOME | KEGG_RIBOSOME | 83 | -0.579790225 | -2.372777421 | 5.27E-10 | 1.60E-08 | 9.32E-09 | 3936 | tags=73%, list=19%, signal=60% | 6159/6188/6170/6227/51187/6204/6207/140801/6206/6201/6222/6143/11224/6168/6152/6205/6193/6203/6171/6147/6230/6210/6209/23521/6161/6130/6232/6160/6141/6155/6202/6124/6136/6133/6194/6137/4736/6135/6167/9045/6191/6181/6176/6132/6218/6229/6146/6228/9349/51065/6169/6224/6122/6138/6164/6189/6125/6134/6129/6165/6156 |
| KEGG_CELL_CYCLE | KEGG_CELL_CYCLE | 123 | 0.540679054 | 2.220801284 | 6.02E-10 | 1.60E-08 | 9.32E-09 | 3233 | tags=49%, list=15%, signal=41% | 891/991/9232/983/9133/7027/701/3065/4175/5111/4085/7272/898/699/990/890/4173/8318/10971/9134/994/4174/4171/2810/993/10926/51434/9700/4609/6502/23594/4176/1026/9978/4998/5347/4172/8317/9088/10459/894/5591/1871/8454/11200/1870/51529/7534/1022/5001/3066/1017/1029/995/5925/902/1647/4193/8697/5933 |
| KEGG_PYRIMIDINE_METABOLISM | KEGG_PYRIMIDINE_METABOLISM | 95 | 0.572605753 | 2.265523959 | 8.80E-10 | 2.05E-08 | 1.19E-08 | 4978 | tags=56%, list=24%, signal=43% | 1890/6241/7378/7083/4860/10622/7371/4830/51251/5427/1503/7298/129607/5437/1841/7296/56655/5435/87178/22978/3704/54107/5425/51728/30834/6240/55703/7372/5441/9533/5436/23649/5433/84172/5557/661/5434/57804/1635/10714/5438/29922/790/25885/30833/5439/5424/124583/956/11128/246721/957/10201 |
| KEGG_FOCAL_ADHESION | KEGG_FOCAL_ADHESION | 197 | -0.43077595 | -2.035088739 | 1.78E-09 | 3.68E-08 | 2.14E-08 | 4723 | tags=44%, list=23%, signal=34% | 23396/81/5170/5155/1291/5062/5602/1292/3791/1499/3696/5908/3672/3611/3685/5290/6654/5728/63923/3908/5906/7414/7424/5649/5829/6655/3678/53358/29780/5159/83660/7058/7059/22801/7423/10398/5578/3910/3480/5228/4233/5154/25759/55742/1950/1956/1101/5747/2909/5291/3679/3909/8516/3688/7148/3913/2335/2932/80310/10000/4659/5579/9475/10319/1793/10451/3915/673/5594/3918/5063/4638/2318/2534/3680/3911/5295/5156/3693/2317/858/596/857/22798/56034/595 |
| KEGG_TOLL_LIKE_RECEPTOR_SIGNALING_PATHWAY | KEGG_TOLL_LIKE_RECEPTOR_SIGNALING_PATHWAY | 100 | 0.528553868 | 2.107904344 | 1.85E-08 | 3.44E-07 | 2.00E-07 | 4902 | tags=48%, list=23%, signal=37% | 3665/6772/4615/5603/3627/3654/3576/6351/7097/4283/54472/4790/3553/9641/29110/3593/3569/7187/8517/5606/841/6373/8772/5293/5608/6416/5296/942/23643/51311/6352/7124/1326/1147/3455/5599/6696/5604/51135/941/1432/23533/7096/958/5605/5595/148022/3440 |
| KEGG_CYTOSOLIC_DNA_SENSING_PATHWAY | KEGG_CYTOSOLIC_DNA_SENSING_PATHWAY | 54 | 0.622625668 | 2.238290305 | 1.83E-07 | 3.09E-06 | 1.80E-06 | 3414 | tags=43%, list=16%, signal=36% | 3665/10622/29108/834/3627/6351/9447/4790/3553/9641/29110/51728/55703/3569/11035/9533/103/8517/661/6352/23586/1147/4793 |
| KEGG_ANTIGEN_PROCESSING_AND_PRESENTATION | KEGG_ANTIGEN_PROCESSING_AND_PRESENTATION | 78 | 0.562477787 | 2.154604188 | 2.40E-07 | 3.71E-06 | 2.16E-06 | 5639 | tags=55%, list=27%, signal=40% | 5721/3308/5720/1508/10437/3309/6890/1514/6891/3312/10197/3109/5993/1520/3112/3133/8625/6892/821/925/3824/3122/4261/567/3134/5641/3106/3135/4802/3809/3823/3111/2923/3108/3119/3440/3803/3105/3115/3107/115653/811/4049 |
| KEGG_DNA_REPLICATION | KEGG_DNA_REPLICATION | 36 | 0.676205602 | 2.240532047 | 6.94E-07 | 9.93E-06 | 5.79E-06 | 3406 | tags=64%, list=16%, signal=54% | 5427/4175/5111/10535/6119/4173/4174/4171/2237/56655/6742/54107/5425/4176/5982/4172/1763/5983/23649/5557/57804/5984/10714 |
| KEGG_RIG_I_LIKE_RECEPTOR_SIGNALING_PATHWAY | KEGG_RIG_I_LIKE_RECEPTOR_SIGNALING_PATHWAY | 71 | 0.557618118 | 2.097550336 | 9.10E-07 | 1.21E-05 | 7.05E-06 | 4902 | tags=44%, list=23%, signal=34% | 3665/79671/64135/9636/5603/3627/3576/4790/9641/9474/843/29110/3593/79132/7187/8517/841/8772/7124/23586/1147/8717/7706/4793/5599/1432/7186/64343/10010/338376/3440 |
| KEGG_NOD_LIKE_RECEPTOR_SIGNALING_PATHWAY | KEGG_NOD_LIKE_RECEPTOR_SIGNALING_PATHWAY | 62 | 0.574613845 | 2.125587193 | 1.12E-06 | 1.39E-05 | 8.11E-06 | 3991 | tags=52%, list=19%, signal=42% | 64127/29108/2919/84674/2920/834/5603/3576/838/4790/3553/6347/330/6355/3569/6354/8517/260434/841/10910/6352/7124/64170/1147/4793/58484/59082/5599/9051/7205/114548/1432 |
| KEGG_GRAFT_VERSUS_HOST_DISEASE | KEGG_GRAFT_VERSUS_HOST_DISEASE | 34 | 0.680264328 | 2.199234663 | 2.07E-06 | 2.41E-05 | 1.40E-05 | 5241 | tags=74%, list=25%, signal=55% | 3002/5551/3458/3553/3569/3109/3112/3133/942/7124/356/3824/3122/3134/3106/3135/941/940/3111/3108/3119/3803/3105/3115/3107 |
| KEGG_AMINOACYL_TRNA_BIOSYNTHESIS | KEGG_AMINOACYL_TRNA_BIOSYNTHESIS | 41 | 0.631889471 | 2.150817914 | 2.65E-06 | 2.90E-05 | 1.69E-05 | 4072 | tags=56%, list=20%, signal=45% | 8565/7453/6897/2617/4141/3376/2193/5917/4677/54938/10056/92935/3035/7407/6301/51067/55157/123263/23395/2058/10667/5859/833 |
| KEGG_ALLOGRAFT_REJECTION | KEGG_ALLOGRAFT_REJECTION | 33 | 0.676437467 | 2.185818781 | 2.86E-06 | 2.96E-05 | 1.72E-05 | 5241 | tags=73%, list=25%, signal=55% | 3002/5551/3458/3593/3109/3112/3133/942/7124/356/3122/3134/3106/3135/941/3586/940/3111/958/3108/3119/3105/3115/3107 |
| KEGG_PRIMARY_IMMUNODEFICIENCY | KEGG_PRIMARY_IMMUNODEFICIENCY | 35 | 0.655153111 | 2.1489839 | 3.85E-06 | 3.77E-05 | 2.20E-05 | 4734 | tags=57%, list=23%, signal=44% | 29760/6890/3932/6891/3718/915/29851/3575/3561/8517/5993/8625/925/916/4261/23495/7535/5788/958/326 |
| KEGG_PURINE_METABOLISM | KEGG_PURINE_METABOLISM | 154 | 0.429509186 | 1.818220227 | 7.66E-06 | 7.13E-05 | 4.15E-05 | 4978 | tags=43%, list=24%, signal=33% | 9615/6241/4860/10622/204/4830/51251/5427/5437/2618/56655/5435/353/87178/471/3614/22978/3251/272/3704/158/11164/7498/54107/5425/203/5151/51728/30834/132/6240/55703/5441/9533/5436/23649/5433/8833/84172/5147/5557/661/5434/57804/109/5315/9060/10714/5438/29922/51292/25885/5634/30833/5439/5424/5153/10606/124583/5150/956/11128/246721/957/5142/10201 |
| KEGG_EPITHELIAL_CELL_SIGNALING_IN_HELICOBACTER_PYLORI_INFECTION | KEGG_EPITHELIAL_CELL_SIGNALING_IN_HELICOBACTER_PYLORI_INFECTION | 68 | 0.521264471 | 1.947877941 | 1.13E-05 | 0.000100063 | 5.83E-05 | 3724 | tags=43%, list=18%, signal=35% | 3579/2919/9114/526/5603/1839/9550/533/3576/6868/9296/5336/4790/4067/1445/51382/528/245973/8517/102/51606/523/6416/8992/6352/1147/836/5599/23545 |
| KEGG_SPLICEOSOME | KEGG_SPLICEOSOME | 121 | 0.445031204 | 1.827523743 | 3.17E-05 | 0.000268315 | 0.000156403 | 4922 | tags=49%, list=24%, signal=37% | 9775/51645/6637/6636/6632/4686/8683/3183/57819/8896/10907/23450/51690/6634/9343/83443/4809/10465/10189/6627/55110/6631/3312/51639/23658/25804/6628/6427/9716/26121/84950/6432/6426/10713/11157/4670/57461/9128/5356/10084/6626/4116/9410/6434/55119/6428/10285/10915/8559/1665/3190/10929/55660/27258/6633/9879/22827/10772/27339 |
| KEGG_HOMOLOGOUS_RECOMBINATION | KEGG_HOMOLOGOUS_RECOMBINATION | 28 | 0.66766775 | 2.078995556 | 4.64E-05 | 0.000372909 | 0.000217372 | 4287 | tags=61%, list=21%, signal=48% | 6119/5888/6742/5425/7979/641/675/8438/25788/57804/7156/146956/10714/10111/6117/5889/5424 |
| KEGG_TYPE_I_DIABETES_MELLITUS | KEGG_TYPE_I_DIABETES_MELLITUS | 39 | 0.608640398 | 2.055568378 | 4.81E-05 | 0.000372909 | 0.000217372 | 5639 | tags=72%, list=27%, signal=52% | 3002/5551/3458/3553/3593/3109/3112/3133/942/3329/7124/356/3122/3134/2571/3106/3135/941/940/5798/3111/3108/3119/3105/3115/3107/3630/4049 |
| KEGG_AXON_GUIDANCE | KEGG_AXON_GUIDANCE | 129 | -0.395171909 | -1.760913447 | 5.34E-05 | 0.000388048 | 0.000226197 | 3599 | tags=37%, list=17%, signal=31% | 64221/80031/8829/1944/1949/9901/25/6092/6387/59277/1946/9723/3983/25791/3897/9353/57715/2051/57556/4233/1942/7869/5532/6586/5747/4773/1945/3688/2932/9423/9475/5594/1073/1808/23365/5063/57522/2047/22885/5362/56920/2534/4772/2770/10725/1948/23380/63928 |
| KEGG_ECM_RECEPTOR_INTERACTION | KEGG_ECM_RECEPTOR_INTERACTION | 82 | -0.452954116 | -1.843913846 | 5.42E-05 | 0.000388048 | 0.000226197 | 5463 | tags=50%, list=26%, signal=37% | 22987/3690/1289/7450/1278/50509/7060/1291/1292/3696/51206/3672/9899/3685/63923/3908/5649/3678/7058/7059/22801/3910/3339/1101/3679/3909/8516/3688/7148/3913/2335/1605/10319/3915/6385/3918/3680/3911/6383/3693/22798 |
| KEGG_VASCULAR_SMOOTH_MUSCLE_CONTRACTION | KEGG_VASCULAR_SMOOTH_MUSCLE_CONTRACTION | 108 | -0.410987545 | -1.781185951 | 6.22E-05 | 0.000426953 | 0.000248875 | 4285 | tags=42%, list=21%, signal=33% | 5567/2983/552/10266/2776/5581/2768/4881/10672/805/8398/112/2778/3779/9138/4882/10398/23236/5578/4660/115/2977/94274/196883/2767/800/3778/4659/5579/5332/9475/148/5592/59/673/5594/23365/4638/3710/72/185/10335/4629/27345/108 |
| KEGG_AUTOIMMUNE_THYROID_DISEASE | KEGG_AUTOIMMUNE_THYROID_DISEASE | 48 | 0.561817178 | 1.966510158 | 6.43E-05 | 0.000426953 | 0.000248875 | 6251 | tags=54%, list=30%, signal=38% | 3002/5551/3109/3112/3133/942/356/3122/3134/3106/3135/941/3586/940/3111/958/3108/3119/3440/3105/7253/3115/3107/1493/959/3567 |
| KEGG_DILATED_CARDIOMYOPATHY | KEGG_DILATED_CARDIOMYOPATHY | 89 | -0.437123919 | -1.806046485 | 7.09E-05 | 0.000446633 | 0.000260346 | 4285 | tags=40%, list=21%, signal=32% | 5567/3696/7042/93589/3672/3685/3908/112/3678/2778/22801/783/70/115/153/6444/196883/6442/3679/8516/3688/6546/1605/5350/9254/7169/7134/1674/3680/6443/781/3693/1756/7168/6445/108 |
| KEGG_CYTOKINE_CYTOKINE_RECEPTOR_INTERACTION | KEGG_CYTOKINE_CYTOKINE_RECEPTOR_INTERACTION | 253 | 0.361645784 | 1.60747411 | 7.20E-05 | 0.000446633 | 0.000260346 | 5947 | tags=47%, list=29%, signal=34% | 3579/6364/7852/3566/27242/1236/2919/10563/2920/9180/29949/3595/58191/8743/50604/3627/3459/10663/3576/6362/6351/6367/4283/4050/3458/55801/1438/3553/6347/3593/50615/1439/6355/8807/3575/3569/6354/6363/939/1234/11009/51561/2921/84957/51330/8797/3561/3559/6372/6373/8795/4055/7133/3597/8744/85480/7132/6352/7124/3460/356/6375/1230/9966/3594/50616/3455/729230/6361/23495/3600/7293/3587/58985/10673/3586/3601/64109/2056/1441/4982/8741/3626/3577/3604/2833/8740/5617/6374/958/3588/338376/3440/3560/2322/6357/64806/4352/3570/1233/56832/643/8794/116379/3554/8200/23529/80301/3467/4049/3589/8792/6356/1524/6346/83729/1440/8995/3563 |
| KEGG_CITRATE_CYCLE_TCA_CYCLE | KEGG_CITRATE_CYCLE_TCA_CYCLE | 29 | 0.632276323 | 1.981221552 | 0.000136444 | 0.000818666 | 0.000477207 | 5304 | tags=69%, list=25%, signal=52% | 3419/8802/4191/6390/3418/3420/50/2271/5106/4190/5162/6392/1737/1738/5091/55753/5160/8803/47/6391 |
| KEGG_MELANOGENESIS | KEGG_MELANOGENESIS | 98 | -0.413627134 | -1.731491235 | 0.00016705 | 0.000970979 | 0.000565992 | 5218 | tags=50%, list=25%, signal=38% | 10488/1638/2033/1910/817/148327/7475/4893/7481/434/1855/5567/1857/1499/2776/81029/90993/805/112/7477/4254/2778/8321/1906/23236/1856/5578/7976/11211/115/196883/8325/8324/1387/818/2932/64764/5579/5332/7472/5594/8322/4286/83439/2770/6934/3815/7482/108 |
| KEGG_APOPTOSIS | KEGG_APOPTOSIS | 87 | 0.457341893 | 1.787544105 | 0.000189985 | 0.001070826 | 0.000624194 | 3310 | tags=37%, list=16%, signal=31% | 54205/4615/3656/840/8837/8743/3654/9131/4790/3553/637/843/823/1439/330/2021/8797/8517/841/5576/8795/8772/5293/5296/7132/7124/356/581/1147/8717/836/11213 |
| KEGG_SYSTEMIC_LUPUS_ERYTHEMATOSUS | KEGG_SYSTEMIC_LUPUS_ERYTHEMATOSUS | 105 | 0.429382073 | 1.718325662 | 0.000206805 | 0.001131344 | 0.00065947 | 6944 | tags=55%, list=33%, signal=37% | 3014/9555/3015/6632/6634/3458/55766/6737/2215/6628/3109/717/713/3112/8345/942/55506/8357/8331/7124/3122/8335/714/8329/941/3586/8346/8968/940/85236/8355/8360/3111/8332/8294/8340/958/3108/3119/2904/715/8344/8366/3115/8367/712/8343/8342/8358/959/8359/8354/8348/8350/735/286436/733/8352 |
| KEGG_RNA_POLYMERASE | KEGG_RNA_POLYMERASE | 29 | 0.618813527 | 1.939036227 | 0.000308279 | 0.001599831 | 0.000932556 | 4846 | tags=62%, list=23%, signal=48% | 10622/5437/5435/51728/30834/55703/5441/9533/5436/5433/84172/661/5434/5438/25885/5439/11128/246721 |
| KEGG_P53_SIGNALING_PATHWAY | KEGG_P53_SIGNALING_PATHWAY | 68 | 0.474131575 | 1.771750209 | 0.000309645 | 0.001599831 | 0.000932556 | 4041 | tags=47%, list=19%, signal=38% | 54205/891/6241/983/9133/898/9134/3732/2810/9540/637/1026/3486/83667/894/841/11200/8795/1017/1029/581/55240/1647/4193/836/51246/9538/896/1019/5054/63970/545 |
| KEGG_BASAL_CELL_CARCINOMA | KEGG_BASAL_CELL_CARCINOMA | 53 | -0.483600309 | -1.838778706 | 0.000374135 | 0.001833096 | 0.001068528 | 4754 | tags=49%, list=23%, signal=38% | 7475/7481/1855/1857/1499/650/324/81029/652/7477/8321/1856/7976/11211/8325/8324/2932/7472/8322/5727/83439/6934/2736/2737/7482/8313 |
| KEGG_UBIQUITIN_MEDIATED_PROTEOLYSIS | KEGG_UBIQUITIN_MEDIATED_PROTEOLYSIS | 132 | 0.403286528 | 1.661394987 | 0.000374503 | 0.001833096 | 0.001068528 | 4118 | tags=39%, list=20%, signal=31% | 140739/991/11065/9246/7334/9021/9320/7332/389898/7323/6921/6923/27338/51434/23291/6502/8651/7321/23624/9978/330/7319/10055/9040/83737/9690/8454/7326/51529/4281/9616/7322/9063/672/118424/26272/9817/1161/5371/55236/4193/8697/26091/7324/7329/55284/7318/7325/997/65264/51465 |
| KEGG_LEISHMANIA_INFECTION | KEGG_LEISHMANIA_INFECTION | 67 | 0.476925088 | 1.78232016 | 0.000390885 | 0.001864222 | 0.001086671 | 4874 | tags=48%, list=23%, signal=37% | 6772/4615/5603/3654/3459/7097/3458/4790/3553/4843/3593/2215/3676/3689/3109/4688/3112/1535/7124/3460/65108/3122/4793/51135/3586/1432/2002/1378/3111/3108/5595/3119 |
| KEGG_REGULATION_OF_ACTIN_CYTOSKELETON | KEGG_REGULATION_OF_ACTIN_CYTOSKELETON | 210 | -0.314572113 | -1.494392159 | 0.000425585 | 0.001978969 | 0.001153559 | 3872 | tags=33%, list=19%, signal=27% | 3672/324/5305/3685/5290/6654/1128/5962/200576/7414/2261/8395/2768/26230/10672/2246/5829/6655/3678/5159/10163/9138/7114/22801/10398/22808/2149/9459/5154/2254/2258/1950/1956/6237/5747/2909/5291/3679/2263/5217/8516/3688/2335/2260/80310/4659/8826/9475/55970/85477/1793/10451/1730/10152/673/5594/79784/1073/23365/8396/5063/4638/3680/4628/5295/5156/3693/8874/2934/56034 |
| KEGG_FRUCTOSE_AND_MANNOSE_METABOLISM | KEGG_FRUCTOSE_AND_MANNOSE_METABOLISM | 34 | 0.588442511 | 1.902382815 | 0.000527809 | 0.002394449 | 0.001395745 | 3454 | tags=44%, list=17%, signal=37% | 57016/7167/3099/29925/7264/226/5373/4351/8898/5208/29085/5214/5210/29926/8776 |
| KEGG_DRUG_METABOLISM_OTHER_ENZYMES | KEGG_DRUG_METABOLISM_OTHER_ENZYMES | 39 | 0.553644578 | 1.869830347 | 0.000621344 | 0.002751667 | 0.001603971 | 3336 | tags=36%, list=16%, signal=30% | 1890/7378/7083/7371/3614/3251/3704/7498/9/7372/7172/8833/54576/1551 |
| KEGG_GLYCOLYSIS_GLUCONEOGENESIS | KEGG_GLYCOLYSIS_GLUCONEOGENESIS | 61 | 0.47071079 | 1.737502029 | 0.000863134 | 0.003687203 | 0.002149303 | 4356 | tags=46%, list=21%, signal=36% | 2023/7167/5223/5230/55276/501/3099/220/3939/226/131/2597/217/2821/222/5106/218/5214/5162/5315/1737/1738/2538/2027/5232/5160/3945/3101 |
| KEGG_HYPERTROPHIC_CARDIOMYOPATHY_HCM | KEGG_HYPERTROPHIC_CARDIOMYOPATHY_HCM | 82 | -0.407533732 | -1.659013718 | 0.000872241 | 0.003687203 | 0.002149303 | 4107 | tags=38%, list=20%, signal=30% | 3696/7042/93589/3672/3685/3908/3678/22801/783/70/51422/6444/6442/3679/8516/3688/5563/6546/1605/9254/7169/5565/7134/1674/3680/6443/781/3693/1756/7168/6445 |
| KEGG_ARRHYTHMOGENIC_RIGHT_VENTRICULAR_CARDIOMYOPATHY_ARVC | KEGG_ARRHYTHMOGENIC_RIGHT_VENTRICULAR_CARDIOMYOPATHY_ARVC | 73 | -0.424413884 | -1.696028376 | 0.001181436 | 0.004883269 | 0.002846501 | 4184 | tags=40%, list=20%, signal=32% | 1499/3696/93589/3672/3685/3908/3678/22801/783/6444/1495/1829/6442/1832/3679/8516/3688/6546/1605/9254/83439/1674/3680/6443/781/3693/1756/6934/6445 |
| KEGG_BASE_EXCISION_REPAIR | KEGG_BASE_EXCISION_REPAIR | 33 | 0.556541525 | 1.798390801 | 0.001408706 | 0.005696072 | 0.003320291 | 5052 | tags=61%, list=24%, signal=46% | 5427/5111/5423/2237/56655/6996/27301/54107/5425/23583/55247/57804/142/4350/10714/10039/5424/4913/3978/328 |
| KEGG_WNT_SIGNALING_PATHWAY | KEGG_WNT_SIGNALING_PATHWAY | 146 | -0.340018151 | -1.542580962 | 0.001507475 | 0.005941317 | 0.003463246 | 4809 | tags=40%, list=23%, signal=31% | 817/57680/7475/7481/5602/6500/5533/1855/5528/5567/1857/1499/23002/4041/324/8945/5516/81029/6425/144165/7477/8321/1459/85407/23236/1856/5578/7976/22943/11211/8325/8324/5532/1387/27101/4773/4040/818/2932/27123/5579/5332/7472/9475/4088/8322/23500/83439/5527/4772/6934/56998/79718/10725/7482/8313/11197/595/63928 |
| KEGG_MISMATCH_REPAIR | KEGG_MISMATCH_REPAIR | 22 | 0.635758293 | 1.864914107 | 0.001533243 | 0.005941317 | 0.003463246 | 5452 | tags=73%, list=26%, signal=54% | 5111/6119/6742/9156/5425/5982/5983/57804/4437/5984/10714/6117/5424/5985/3978/4292 |
| KEGG_VIBRIO_CHOLERAE_INFECTION | KEGG_VIBRIO_CHOLERAE_INFECTION | 54 | 0.488258962 | 1.755252566 | 0.001655182 | 0.006282937 | 0.00366238 | 2893 | tags=37%, list=14%, signal=32% | 9114/23480/526/9550/533/30001/9414/9296/5336/10952/51382/528/245973/11014/9601/29927/51606/523/8992/109 |
| KEGG_CARDIAC_MUSCLE_CONTRACTION | KEGG_CARDIAC_MUSCLE_CONTRACTION | 75 | 0.426872537 | 1.626573355 | 0.001825683 | 0.006706869 | 0.003909493 | 2958 | tags=32%, list=14%, signal=28% | 27089/1340/7388/1349/483/7386/9377/1351/488/29796/10975/1537/55799/785/1347/7170/1337/7385/481/7171/1350/7384/1329/476 |
| KEGG_CIRCADIAN_RHYTHM_MAMMAL | KEGG_CIRCADIAN_RHYTHM_MAMMAL | 11 | -0.735401209 | -1.900635245 | 0.001856948 | 0.006706869 | 0.003909493 | 3056 | tags=91%, list=15%, signal=78% | 1407/4862/8863/406/8864/9575/1453/5187/79365/1408 |
| KEGG_NATURAL_KILLER_CELL_MEDIATED_CYTOTOXICITY | KEGG_NATURAL_KILLER_CELL_MEDIATED_CYTOTOXICITY | 128 | 0.383348972 | 1.581632818 | 0.001875039 | 0.006706869 | 0.003909493 | 5241 | tags=45%, list=25%, signal=34% | 3002/80328/8743/3932/3459/5336/5551/3458/637/135250/919/962/2215/6464/6850/5880/3689/4068/8797/3265/3683/8795/3133/5293/3937/259197/5296/7124/3460/4277/356/3824/3383/836/7305/3455/51744/3106/7535/3135/9436/5604/7409/23533/7410/3809/27040/3823/2207/5605/5595/2885/3440/3803/3105/80329/10870/3107 |
| KEGG_GAP_JUNCTION | KEGG_GAP_JUNCTION | 88 | -0.367523389 | -1.510195119 | 0.00324074 | 0.011373164 | 0.006629518 | 3734 | tags=44%, list=18%, signal=37% | 3709/3356/3708/4893/5155/5567/2983/2776/6654/5607/6655/112/5159/2778/5593/23236/5578/10746/115/153/5154/1950/2977/1956/196883/1453/2767/80310/5579/5332/5592/5594/3710/1902/2770/5156/7846/108/56034 |
| KEGG_ADHERENS_JUNCTION | KEGG_ADHERENS_JUNCTION | 72 | -0.395580064 | -1.596120062 | 0.004374479 | 0.015067648 | 0.008783066 | 3430 | tags=33%, list=16%, signal=28% | 25945/7414/10163/1459/3643/5797/3480/4233/10810/1495/1956/2241/1387/2260/5792/8826/4088/5594/7048/83439/2534/56288/6934/10580 |
| KEGG_RNA_DEGRADATION | KEGG_RNA_DEGRADATION | 57 | 0.45568245 | 1.662876377 | 0.004672934 | 0.015803014 | 0.009211717 | 3878 | tags=42%, list=19%, signal=34% | 2023/10200/54512/87178/3313/57819/51690/51010/27257/22894/23658/51013/25804/23019/23404/3329/56915/11157/6499/22803/57472/11340/2027/28960 |
| KEGG_FOLATE_BIOSYNTHESIS | KEGG_FOLATE_BIOSYNTHESIS | 11 | 0.710971756 | 1.749955489 | 0.005035488 | 0.016725013 | 0.009749158 | 2171 | tags=45%, list=10%, signal=41% | 2643/5805/8836/1719/249 |
| KEGG_INTESTINAL_IMMUNE_NETWORK_FOR_IGA_PRODUCTION | KEGG_INTESTINAL_IMMUNE_NETWORK_FOR_IGA_PRODUCTION | 44 | 0.480399287 | 1.651301942 | 0.005217492 | 0.016898643 | 0.009850369 | 5211 | tags=50%, list=25%, signal=38% | 7852/29851/3569/3676/3109/3112/4055/942/3122/23495/3600/10673/941/3586/3601/8741/940/3111/958/3108/3119/3115 |
| KEGG_GLUTATHIONE_METABOLISM | KEGG_GLUTATHIONE_METABOLISM | 45 | 0.478181932 | 1.658855405 | 0.005269469 | 0.016898643 | 0.009850369 | 4527 | tags=49%, list=22%, signal=38% | 51056/6241/2877/5226/2950/9446/6723/2941/79017/3418/2876/6240/2730/2954/4258/2539/2729/26873/2948/373156/2937/4953 |
| KEGG_OOCYTE_MEIOSIS | KEGG_OOCYTE_MEIOSIS | 110 | 0.368698014 | 1.484911196 | 0.005432954 | 0.017127618 | 0.009983841 | 2893 | tags=28%, list=14%, signal=24% | 891/991/9232/983/9133/4085/6790/898/699/5515/10971/9134/810/51434/23291/5499/9700/9978/6195/5347/9088/10459/26271/8454/5519/51806/51529/7534/1017/995/109 |
| KEGG_NUCLEOTIDE_EXCISION_REPAIR | KEGG_NUCLEOTIDE_EXCISION_REPAIR | 43 | 0.480972741 | 1.645216041 | 0.006607166 | 0.020254129 | 0.011806312 | 5193 | tags=53%, list=25%, signal=40% | 5427/5111/6119/56655/54107/5425/9978/5982/5983/1069/1022/57804/1161/902/2067/5984/10714/6117/5424/5985/3978/2965/2074 |
| KEGG_CHEMOKINE_SIGNALING_PATHWAY | KEGG_CHEMOKINE_SIGNALING_PATHWAY | 180 | 0.334295047 | 1.436229934 | 0.006642483 | 0.020254129 | 0.011806312 | 3933 | tags=33%, list=19%, signal=27% | 3579/6772/6364/7852/1236/2919/10563/2920/58191/3627/10663/3576/6362/6351/6367/4283/6774/2787/3718/4790/6347/4067/1445/6464/3055/6355/5880/6354/6363/1234/2773/2921/6773/3265/8517/6372/6373/3702/8976/5293/5296/6352/109/6375/1230/1147/10681/408/5580/729230/4793/6361/5331/7454/1794/2793/2931/5604/7409 |
| KEGG_CALCIUM_SIGNALING_PATHWAY | KEGG_CALCIUM_SIGNALING_PATHWAY | 174 | -0.293942686 | -1.358655663 | 0.007393277 | 0.022179831 | 0.012928821 | 4469 | tags=32%, list=21%, signal=26% | 5256/1131/5533/5260/5567/552/5136/2776/1128/4842/887/2065/805/10800/2066/489/5159/2778/8912/6263/683/490/23236/3973/2149/5578/2774/115/153/6869/1956/196883/5027/5532/7125/3707/818/2767/5737/6546/5023/5579/5332/148/291/493/5350/4638/3710/7220/7134/185/5156/154/108/63928 |
| KEGG_PENTOSE_PHOSPHATE_PATHWAY | KEGG_PENTOSE_PHOSPHATE_PATHWAY | 26 | 0.538999757 | 1.658750356 | 0.008058814 | 0.02379269 | 0.01386897 | 4085 | tags=46%, list=20%, signal=37% | 55276/5226/226/6888/2821/64080/25796/5214/2539/51071/5634/7086 |
| KEGG_TGF_BETA_SIGNALING_PATHWAY | KEGG_TGF_BETA_SIGNALING_PATHWAY | 84 | -0.355768054 | -1.456541337 | 0.008189472 | 0.023800654 | 0.013873613 | 4040 | tags=37%, list=19%, signal=30% | 7042/57154/93/650/5308/654/5516/130399/652/7058/7059/9241/94/1387/64750/657/151449/4093/9475/4090/658/4088/5594/7048/6667/655/1634/5934/3625/10468/3400 |
| KEGG_AMINO_SUGAR_AND_NUCLEOTIDE_SUGAR_METABOLISM | KEGG_AMINO_SUGAR_AND_NUCLEOTIDE_SUGAR_METABOLISM | 44 | 0.467836822 | 1.608120315 | 0.008789296 | 0.025150908 | 0.014660688 | 2456 | tags=34%, list=12%, signal=30% | 55276/3099/2582/29925/7264/5373/55907/2821/64841/54187/2584/4351/55577/80896/10007 |
| KEGG_GALACTOSE_METABOLISM | KEGG_GALACTOSE_METABOLISM | 26 | 0.532077148 | 1.637446302 | 0.009535212 | 0.02687196 | 0.015663904 | 5534 | tags=50%, list=27%, signal=37% | 55276/3099/2582/2584/2595/2717/5214/2538/3101/2585/3906/57818/2720 |
| KEGG_NON_HOMOLOGOUS_END_JOINING | KEGG_NON_HOMOLOGOUS_END_JOINING | 13 | 0.655616501 | 1.692175991 | 0.009804091 | 0.027217327 | 0.015865222 | 3666 | tags=54%, list=18%, signal=44% | 2237/2547/7520/5591/7518/3981/10111 |
| KEGG_NOTCH_SIGNALING_PATHWAY | KEGG_NOTCH_SIGNALING_PATHWAY | 47 | -0.426375474 | -1.580117343 | 0.011727626 | 0.032078505 | 0.018698846 | 5934 | tags=62%, list=28%, signal=44% | 9794/1487/196403/4855/8850/5986/28514/2033/55534/8650/1855/1857/2648/4854/3280/1840/9541/182/23220/22938/1856/3714/1387/3516/9253/3955/9612/4853/84441 |
| KEGG_JAK_STAT_SIGNALING_PATHWAY | KEGG_JAK_STAT_SIGNALING_PATHWAY | 153 | 0.327823756 | 1.388056972 | 0.011931317 | 0.03216268 | 0.018747912 | 4293 | tags=33%, list=21%, signal=26% | 6772/3566/10379/9021/9180/29949/3595/50604/3459/6774/3718/3458/55801/1438/4609/8651/23624/8027/3593/50615/1439/3575/3569/11009/51561/5292/894/3561/6773/3559/5293/3597/9063/85480/5296/3460/3594/50616/3455/3600/896/3587/58985/3586/3601/1154/64109/2056/1441/23533 |
| KEGG_TIGHT_JUNCTION | KEGG_TIGHT_JUNCTION | 131 | -0.299882814 | -1.338721499 | 0.013693518 | 0.036385633 | 0.021209509 | 3554 | tags=29%, list=17%, signal=24% | 5728/5516/5581/57530/8777/1365/58494/1459/5010/10398/93643/22808/5578/84552/7122/1495/4621/6237/51776/2037/6709/2035/154810/10000/5865/5521/5579/79784/56288/9223/9863/4628/2770/9073/4629/83700/137075/9076 |
| KEGG_ENDOMETRIAL_CANCER | KEGG_ENDOMETRIAL_CANCER | 52 | -0.405129428 | -1.540468284 | 0.014299067 | 0.037459527 | 0.021835491 | 4691 | tags=44%, list=22%, signal=34% | 5170/4893/1499/324/3611/5290/6654/5728/6655/1950/1495/1956/2309/5291/2932/10000/673/5594/83439/5295/6934/8313/595 |
| KEGG_CYSTEINE_AND_METHIONINE_METABOLISM | KEGG_CYSTEINE_AND_METHIONINE_METABOLISM | 33 | 0.485529362 | 1.568924326 | 0.015035371 | 0.038841375 | 0.022640983 | 1591 | tags=27%, list=8%, signal=25% | 2806/2805/191/259307/6723/1786/3939/1789/55256 |
| KEGG_N_GLYCAN_BIOSYNTHESIS | KEGG_N_GLYCAN_BIOSYNTHESIS | 45 | 0.447909639 | 1.553838144 | 0.019121851 | 0.048721429 | 0.028400154 | 4935 | tags=53%, list=24%, signal=41% | 1798/10195/56052/199857/85365/91869/79053/7991/79868/29880/6184/29929/1650/8818/57171/84920/6185/8813/201595/1603/4249/8703/146664/79087 |
