# Supplementary material for: Exploration of Biomarkers of Psoriasis through Combined Multiomics Analysis
Source: Mediators Inflamm. 2022 Sep 23;2022:7731082. doi: 10.1155/2022/7731082 (PMC9525798; doi:10.1155/2022/7731082)
Supplement: Supplementary Materials — Supplementary Figure 1 The PCA of gene expression in psoriasis lesions and healthy controls in GSE13355 database. Supplementary Figure 2 The PCA and methylation distribution density in psoriasis lesions and healthy controls from the GSE73894 dataset. (A) PCA in GSE73894. (B) Methylation distribution density in GSE73894. Supplementary Table 1 Identification of DEGs in the psoriatic lesions and healthy control group in GSE13355. Supplementary Table 2 GO analysis on 767 DEGs in GSE13355. Supplementary Table 3 KEGG analysis on 767 DEGs in GSE13355. Supplementary Table 4 Identification of hyper-MR-genes. Supplementary Table 5 Identification of hypo-MR-genes. Supplementary Table 6 GO analysis of hyper-MR-genes. Supplementary Table 7 GO analysis of hypo-MR-genes. Supplementary Table 8 KEGG analysis of hyper-MR-genes. Supplementary Table 9 KEGG analysis of hypo-MR-genes. Supplementary Table 10 GO analysis through single-gene GSEA of GJB2. Supplementary Table 11 KEGG analysis through single-gene GSEA of GJB2. [file 7731082.f1.zip › Supplementary Table 2 (1).docx]

| GO analysis on 767 DEGs in GSE13355 | | | | | | | | | |
| --- | --- | --- | --- | --- | --- | --- | --- | --- | --- |
| ONTOLOGY | ID | Description | GeneRatio | BgRatio | pvalue | p.adjust | qvalue | geneID | Count |
| BP | GO:0043588 | skin development | 51/706 | 415/18862 | 7.37E-14 | 3.60E-10 | 2.89E-10 | KRT77/GAL/KRT79/FA2H/LGR5/FLG2/SPRR4/SCEL/KRT15/CLDN1/LORICRIN/BCL2/KRT19/TFAP2B/IGFBP5/TRIM16/SLC27A4/LTB/TMEM79/IL20/KRT6B/FERMT1/ZBED2/EPHA2/EREG/COMP/IVL/GRHL3/CERS3/DSG3/DSC2/ABCA12/ZDHHC21/CDH3/SPRR1A/ALOX12B/SPRR1B/KRT6A/WNT5A/TGM3/CNFN/TGM1/SERPINB13/SPRR3/SPRR2G/KLK13/KRT16/HPSE/S100A7/LCE3D/PI3 | 51 |
| BP | GO:0008544 | epidermis development | 53/706 | 463/18862 | 4.19E-13 | 1.02E-09 | 8.21E-10 | KRT77/GAL/KRT79/FA2H/LGR5/CST6/FLG2/SPRR4/SCEL/SLITRK6/KRT15/MCOLN3/LORICRIN/BCL2/KRT19/C1orf68/IGFBP5/TRIM16/PPARD/EZH2/TMEM79/IL20/KRT6B/FERMT1/ZBED2/EPHA2/EREG/FABP5/IVL/GRHL3/CRABP2/CERS3/DSG3/DSC2/ABCA12/ZDHHC21/CDH3/SPRR1A/SPRR1B/KRT6A/WNT5A/TGM3/CNFN/TGM1/SERPINB13/SPRR3/SPRR2G/KLK13/KRT16/HPSE/S100A7/LCE3D/PI3 | 53 |
| BP | GO:0009615 | response to virus | 43/706 | 359/18862 | 1.66E-11 | 2.71E-08 | 2.17E-08 | CD207/GATA3/DCLK1/BCL2/IFI16/CCL19/IRF9/FOSL1/MX2/CCL22/PLSCR1/AIM2/TRIM22/NMI/DDX60/PYCARD/NT5C3A/IFIH1/STAT1/ISG20/APOBEC3B/IFIT1/GBP1/IFIT3/POLR3G/IFI44/IFI44L/PARP9/RTP4/CXCR4/OAS1/MX1/IRF7/OASL/IFI6/RSAD2/ISG15/CXCL9/CXCL10/ZC3H12A/IFI27/OAS2/MMP12 | 43 |
| BP | GO:0051607 | defense response to virus | 35/706 | 260/18862 | 5.24E-11 | 5.12E-08 | 4.11E-08 | CD207/BCL2/IFI16/IRF9/MX2/PLSCR1/AIM2/TRIM22/DDX60/PYCARD/NT5C3A/IFIH1/STAT1/ISG20/APOBEC3B/IFIT1/GBP1/IFIT3/POLR3G/IFI44L/PARP9/RTP4/OAS1/MX1/IRF7/OASL/IFI6/RSAD2/ISG15/CXCL9/CXCL10/ZC3H12A/IFI27/OAS2/MMP12 | 35 |
| BP | GO:0140546 | defense response to symbiont | 35/706 | 260/18862 | 5.24E-11 | 5.12E-08 | 4.11E-08 | CD207/BCL2/IFI16/IRF9/MX2/PLSCR1/AIM2/TRIM22/DDX60/PYCARD/NT5C3A/IFIH1/STAT1/ISG20/APOBEC3B/IFIT1/GBP1/IFIT3/POLR3G/IFI44L/PARP9/RTP4/OAS1/MX1/IRF7/OASL/IFI6/RSAD2/ISG15/CXCL9/CXCL10/ZC3H12A/IFI27/OAS2/MMP12 | 35 |
| BP | GO:0002237 | response to molecule of bacterial origin | 41/706 | 346/18862 | 6.97E-11 | 5.67E-08 | 4.56E-08 | IL37/PPARGC1A/CLDN1/MGST1/FOS/PPARD/CXCL11/CD36/IRF8/FZD5/SLPI/TNIP3/IDO1/GCH1/CCL2/PYCARD/CD24/IRAK2/SELE/NOD2/CCR7/GJB6/ARG1/CXCL2/IL36A/CMPK2/WNT5A/CD274/CXCL1/CXCL9/CXCL13/CXCL8/IL36RN/CXCL10/GJB2/ZC3H12A/S100A8/LTF/S100A7/IL36G/S100A9 | 41 |
| BP | GO:0019730 | antimicrobial humoral response | 25/706 | 142/18862 | 9.88E-11 | 6.90E-08 | 5.54E-08 | MUC7/ANG/PGLYRP4/CXCL11/SLPI/PRSS2/NOD2/RNASE7/PRSS3/CXCL2/WFDC12/KRT6A/CXCL1/CXCL9/CXCL13/CXCL8/IL36RN/CXCL10/S100A8/LCN2/LTF/S100A7/S100A12/S100A9/PI3 | 25 |
| BP | GO:0032496 | response to lipopolysaccharide | 39/706 | 326/18862 | 1.54E-10 | 9.40E-08 | 7.55E-08 | IL37/PPARGC1A/CLDN1/MGST1/FOS/PPARD/CXCL11/CD36/IRF8/SLPI/TNIP3/IDO1/GCH1/CCL2/PYCARD/IRAK2/SELE/NOD2/CCR7/GJB6/ARG1/CXCL2/IL36A/CMPK2/WNT5A/CD274/CXCL1/CXCL9/CXCL13/CXCL8/IL36RN/CXCL10/GJB2/ZC3H12A/S100A8/LTF/S100A7/IL36G/S100A9 | 39 |
| BP | GO:0070268 | cornification | 21/706 | 113/18862 | 1.12E-09 | 6.07E-07 | 4.87E-07 | KRT77/KRT79/KRT15/LORICRIN/KRT19/TMEM79/KRT6B/IVL/CERS3/DSG3/DSC2/SPRR1A/SPRR1B/KRT6A/TGM1/SPRR3/SPRR2G/KLK13/KRT16/LCE3D/PI3 | 21 |
| BP | GO:0060337 | type I interferon signaling pathway | 19/706 | 95/18862 | 1.91E-09 | 9.33E-07 | 7.50E-07 | IRF8/IRF9/MX2/STAT1/ISG20/IFIT1/IFIT3/XAF1/OAS1/MX1/IRF7/OASL/IFI6/WNT5A/RSAD2/ISG15/IFI27/OAS2/MMP12 | 19 |
| BP | GO:0071357 | cellular response to type I interferon | 19/706 | 96/18862 | 2.30E-09 | 1.02E-06 | 8.20E-07 | IRF8/IRF9/MX2/STAT1/ISG20/IFIT1/IFIT3/XAF1/OAS1/MX1/IRF7/OASL/IFI6/WNT5A/RSAD2/ISG15/IFI27/OAS2/MMP12 | 19 |
| BP | GO:0009913 | epidermal cell differentiation | 39/706 | 360/18862 | 2.79E-09 | 1.14E-06 | 9.13E-07 | KRT77/KRT79/FA2H/SPRR4/SCEL/SLITRK6/KRT15/MCOLN3/LORICRIN/KRT19/TRIM16/EZH2/TMEM79/IL20/KRT6B/ZBED2/EPHA2/EREG/IVL/CERS3/DSG3/DSC2/ABCA12/CDH3/SPRR1A/SPRR1B/KRT6A/WNT5A/TGM3/CNFN/TGM1/SERPINB13/SPRR3/SPRR2G/KLK13/KRT16/S100A7/LCE3D/PI3 | 39 |
| BP | GO:0030216 | keratinocyte differentiation | 35/706 | 302/18862 | 3.17E-09 | 1.19E-06 | 9.54E-07 | KRT77/KRT79/SPRR4/SCEL/KRT15/LORICRIN/KRT19/TRIM16/TMEM79/IL20/KRT6B/ZBED2/EPHA2/EREG/IVL/CERS3/DSG3/DSC2/ABCA12/CDH3/SPRR1A/SPRR1B/KRT6A/WNT5A/TGM3/CNFN/TGM1/SERPINB13/SPRR3/SPRR2G/KLK13/KRT16/S100A7/LCE3D/PI3 | 35 |
| BP | GO:0061844 | antimicrobial humoral immune response mediated by antimicrobial peptide | 17/706 | 78/18862 | 3.40E-09 | 1.19E-06 | 9.54E-07 | MUC7/ANG/PGLYRP4/CXCL11/NOD2/RNASE7/CXCL2/KRT6A/CXCL1/CXCL9/CXCL13/CXCL8/CXCL10/LTF/S100A7/S100A12/S100A9 | 17 |
| BP | GO:0034340 | response to type I interferon | 19/706 | 101/18862 | 5.60E-09 | 1.82E-06 | 1.47E-06 | IRF8/IRF9/MX2/STAT1/ISG20/IFIT1/IFIT3/XAF1/OAS1/MX1/IRF7/OASL/IFI6/WNT5A/RSAD2/ISG15/IFI27/OAS2/MMP12 | 19 |
| BP | GO:0140014 | mitotic nuclear division | 34/706 | 296/18862 | 6.69E-09 | 2.04E-06 | 1.64E-06 | BTC/KIF2A/NUF2/CDCA5/AURKB/CENPF/KIF18B/AURKA/BUB1/TGFA/ANLN/SMPD3/KIF11/PRC1/KIF4A/NCAPG/NEK2/ZWINT/EREG/TPX2/BUB1B/KIF2C/NUSAP1/UBE2C/CDK1/MAD2L1/NDC80/PTTG1/TTK/MKI67/CCNB2/CDC20/DLGAP5/CCNB1 | 34 |
| BP | GO:0050673 | epithelial cell proliferation | 42/706 | 428/18862 | 1.30E-08 | 3.74E-06 | 3.00E-06 | FABP7/F3/AGTR1/LGR5/OSR2/PTN/GATA3/CD34/CLDN1/AR/ANG/ESR1/TNMD/APOE/TGFBR3/CCND1/IGFBP5/PPARD/ESRP2/EHF/CYP7B1/HMOX1/TGFA/FERMT1/AREG/ID1/CCL2/KLK8/EPHA2/EREG/STAT1/NOD2/UHRF1/FUT2/SERPINB1/FGFBP1/CDH3/ARG1/LRG1/WNT5A/TGM1/MMP12 | 42 |
| BP | GO:0071216 | cellular response to biotic stimulus | 29/706 | 233/18862 | 1.45E-08 | 3.94E-06 | 3.16E-06 | IL37/PPARGC1A/PPARD/CXCL11/CD36/IRF8/FZD5/TNIP3/CCL2/PYCARD/IRAK2/NOD2/TIGAR/ARG1/CLEC7A/CXCL2/IL36A/CMPK2/WNT5A/CD274/CXCL1/CXCL9/CXCL13/CXCL8/IL36RN/CXCL10/ZC3H12A/LTF/IL36G | 29 |
| BP | GO:0060326 | cell chemotaxis | 34/706 | 306/18862 | 1.55E-08 | 4.00E-06 | 3.21E-06 | CCL27/GREM1/AGTR1/PTN/ACKR4/PDGFD/CCL19/CXCL11/CYP7B1/CH25H/CCL22/HBEGF/CCL2/TPBG/EPHA2/PRKCQ/NOD2/CCR7/CXCL2/CXCR4/CXCR2/WNT5A/CXCL1/CXCL9/CXCL13/CXCL8/CCL18/CXCL10/CCL20/S100A8/S100A7/C10orf99/S100A12/S100A9 | 34 |
| BP | GO:0071219 | cellular response to molecule of bacterial origin | 27/706 | 209/18862 | 2.07E-08 | 4.82E-06 | 3.87E-06 | IL37/PPARGC1A/PPARD/CXCL11/CD36/IRF8/FZD5/TNIP3/CCL2/PYCARD/IRAK2/NOD2/ARG1/CXCL2/IL36A/CMPK2/WNT5A/CD274/CXCL1/CXCL9/CXCL13/CXCL8/IL36RN/CXCL10/ZC3H12A/LTF/IL36G | 27 |
| BP | GO:0007088 | regulation of mitotic nuclear division | 19/706 | 109/18862 | 2.07E-08 | 4.82E-06 | 3.87E-06 | BTC/AURKB/CENPF/AURKA/BUB1/TGFA/SMPD3/NEK2/ZWINT/EREG/BUB1B/NUSAP1/MAD2L1/NDC80/TTK/MKI67/CDC20/DLGAP5/CCNB1 | 19 |
| BP | GO:0034341 | response to interferon-gamma | 26/706 | 197/18862 | 2.42E-08 | 5.14E-06 | 4.13E-06 | CLDN1/HLA-DQB2/CCL19/IFI30/IRF8/IRF9/FCGR1B/CCL22/TRIM22/NMI/GCH1/CCL2/STAT1/SOCS3/GBP1/GBP6/PARP9/ARG1/OAS1/IRF7/OASL/WNT5A/CCL18/CCL20/OAS2/KYNU | 26 |
| BP | GO:0071222 | cellular response to lipopolysaccharide | 26/706 | 197/18862 | 2.42E-08 | 5.14E-06 | 4.13E-06 | IL37/PPARGC1A/PPARD/CXCL11/CD36/IRF8/TNIP3/CCL2/PYCARD/IRAK2/NOD2/ARG1/CXCL2/IL36A/CMPK2/WNT5A/CD274/CXCL1/CXCL9/CXCL13/CXCL8/IL36RN/CXCL10/ZC3H12A/LTF/IL36G | 26 |
| BP | GO:0030593 | neutrophil chemotaxis | 18/706 | 103/18862 | 4.68E-08 | 9.52E-06 | 7.65E-06 | CCL19/CXCL11/CCL22/CCL2/NOD2/CCR7/CXCL2/CXCR2/CXCL1/CXCL9/CXCL13/CXCL8/CCL18/CXCL10/CCL20/S100A8/S100A12/S100A9 | 18 |
| BP | GO:0071346 | cellular response to interferon-gamma | 24/706 | 177/18862 | 4.94E-08 | 9.65E-06 | 7.75E-06 | CLDN1/HLA-DQB2/CCL19/IFI30/IRF8/IRF9/FCGR1B/CCL22/TRIM22/NMI/CCL2/STAT1/SOCS3/GBP1/GBP6/PARP9/ARG1/OAS1/IRF7/OASL/WNT5A/CCL18/CCL20/OAS2 | 24 |
| BP | GO:0045069 | regulation of viral genome replication | 16/706 | 83/18862 | 6.25E-08 | 1.17E-05 | 9.43E-06 | BCL2/IFI16/SLPI/PLSCR1/ISG20/APOBEC3B/IFIT1/TOP2A/OAS1/MX1/OASL/RSAD2/ISG15/CXCL8/OAS2/LTF | 16 |
| BP | GO:0045071 | negative regulation of viral genome replication | 13/706 | 54/18862 | 6.81E-08 | 1.19E-05 | 9.58E-06 | IFI16/SLPI/PLSCR1/ISG20/APOBEC3B/IFIT1/OAS1/MX1/OASL/RSAD2/ISG15/OAS2/LTF | 13 |
| BP | GO:0019079 | viral genome replication | 20/706 | 129/18862 | 6.84E-08 | 1.19E-05 | 9.58E-06 | BCL2/PIK3C2G/IFI16/SLPI/PLSCR1/CCL2/ISG20/APOBEC3B/IFIT1/TOP2A/OAS1/MX1/OASL/RSAD2/ISG15/CXCL8/ZC3H12A/IFI27/OAS2/LTF | 20 |
| BP | GO:0048247 | lymphocyte chemotaxis | 14/706 | 64/18862 | 7.87E-08 | 1.33E-05 | 1.06E-05 | CCL27/CCL19/CXCL11/CYP7B1/CH25H/CCL22/CCL2/WNT5A/CXCL13/CCL18/CXCL10/CCL20/S100A7/C10orf99 | 14 |
| BP | GO:0043903 | regulation of biological process involved in symbiotic interaction | 25/706 | 197/18862 | 9.79E-08 | 1.59E-05 | 1.28E-05 | BCL2/IFI16/SLPI/PLSCR1/TRIM22/KPNA2/STAT1/ISG20/TRIM14/APOBEC3B/IFIT1/TMPRSS4/TOP2A/ARG1/CXCR4/OAS1/MX1/LAMP3/OASL/KRT6A/RSAD2/ISG15/CXCL8/OAS2/LTF | 25 |
| BP | GO:1990868 | response to chemokine | 17/706 | 97/18862 | 1.06E-07 | 1.59E-05 | 1.28E-05 | ACKR4/CCL19/CXCL11/CCL22/CCL2/CCR7/CXCL2/CXCR4/CXCR2/CXCL1/CXCL9/CXCL13/CXCL8/CCL18/CXCL10/ZC3H12A/CCL20 | 17 |
| BP | GO:1990869 | cellular response to chemokine | 17/706 | 97/18862 | 1.06E-07 | 1.59E-05 | 1.28E-05 | ACKR4/CCL19/CXCL11/CCL22/CCL2/CCR7/CXCL2/CXCR4/CXCR2/CXCL1/CXCL9/CXCL13/CXCL8/CCL18/CXCL10/ZC3H12A/CCL20 | 17 |
| BP | GO:0030595 | leukocyte chemotaxis | 27/706 | 226/18862 | 1.07E-07 | 1.59E-05 | 1.28E-05 | CCL27/GREM1/PTN/CCL19/CXCL11/CYP7B1/CH25H/CCL22/CCL2/NOD2/CCR7/CXCL2/CXCR4/CXCR2/WNT5A/CXCL1/CXCL9/CXCL13/CXCL8/CCL18/CXCL10/CCL20/S100A8/S100A7/C10orf99/S100A12/S100A9 | 27 |
| BP | GO:0070098 | chemokine-mediated signaling pathway | 16/706 | 88/18862 | 1.47E-07 | 2.07E-05 | 1.66E-05 | ACKR4/CCL19/CXCL11/CCL22/CCL2/CCR7/CXCL2/CXCR4/CXCR2/CXCL1/CXCL9/CXCL13/CXCL8/CCL18/CXCL10/CCL20 | 16 |
| BP | GO:0051783 | regulation of nuclear division | 20/706 | 135/18862 | 1.48E-07 | 2.07E-05 | 1.66E-05 | BTC/AURKB/CENPF/AURKA/BUB1/TGFA/SMPD3/NEK2/ZWINT/EREG/BUB1B/NUSAP1/MAD2L1/NDC80/TTK/MKI67/CDC20/DLGAP5/WNT5A/CCNB1 | 20 |
| BP | GO:1903900 | regulation of viral life cycle | 21/706 | 148/18862 | 1.54E-07 | 2.09E-05 | 1.68E-05 | BCL2/IFI16/SLPI/PLSCR1/TRIM22/KPNA2/ISG20/TRIM14/APOBEC3B/IFIT1/TMPRSS4/TOP2A/OAS1/MX1/LAMP3/OASL/RSAD2/ISG15/CXCL8/OAS2/LTF | 21 |
| BP | GO:0048285 | organelle fission | 43/706 | 486/18862 | 1.71E-07 | 2.20E-05 | 1.77E-05 | BTC/PPARGC1A/DNM1/KIF2A/NUF2/CDCA5/AURKB/CENPF/KIF18B/AURKA/BUB1/MX2/TGFA/ANLN/SMPD3/KIF11/PRC1/KIF4A/CCNE2/NCAPG/NEK2/ZWINT/CCNE1/EREG/TPX2/BUB1B/KIF2C/NUSAP1/UBE2C/CDK1/MAD2L1/NDC80/PTTG1/TTK/TOP2A/MKI67/CCNB2/CDC20/DLGAP5/CKS2/MX1/WNT5A/CCNB1 | 43 |
| BP | GO:0051983 | regulation of chromosome segregation | 16/706 | 89/18862 | 1.73E-07 | 2.20E-05 | 1.77E-05 | AURKB/CENPF/BUB1/PLSCR1/ZWINT/BUB1B/KIF2C/UBE2C/MAD2L1/NDC80/PTTG1/TTK/MKI67/CDC20/DLGAP5/CCNB1 | 16 |
| BP | GO:0071621 | granulocyte chemotaxis | 19/706 | 124/18862 | 1.76E-07 | 2.20E-05 | 1.77E-05 | CCL19/CXCL11/CCL22/CCL2/NOD2/CCR7/CXCL2/CXCR2/CXCL1/CXCL9/CXCL13/CXCL8/CCL18/CXCL10/CCL20/S100A8/S100A7/S100A12/S100A9 | 19 |
| BP | GO:0001819 | positive regulation of cytokine production | 40/706 | 437/18862 | 1.90E-07 | 2.32E-05 | 1.86E-05 | F3/POSTN/GATA3/CD34/RORA/ADIPOQ/IL17D/LPL/TRIM16/IFI16/CCL19/LTB/CD36/IRF8/HMOX1/RASGRP1/FZD5/IL4R/FERMT1/AIM2/IDO1/PYCARD/AFAP1L2/CD2/EREG/PRKCQ/PLA2G3/IFIH1/STAT1/NOD2/POLR3G/SLC7A5/CCR7/CLEC7A/IRF7/WNT5A/RSAD2/CD274/MMP12/HPSE | 40 |
| BP | GO:0000070 | mitotic sister chromatid segregation | 22/706 | 164/18862 | 2.15E-07 | 2.56E-05 | 2.06E-05 | NUF2/CDCA5/AURKB/CENPF/KIF18B/BUB1/PRC1/KIF4A/NCAPG/NEK2/ZWINT/BUB1B/KIF2C/NUSAP1/UBE2C/MAD2L1/NDC80/PTTG1/TTK/CDC20/DLGAP5/CCNB1 | 22 |
| BP | GO:0060333 | interferon-gamma-mediated signaling pathway | 16/706 | 91/18862 | 2.38E-07 | 2.77E-05 | 2.23E-05 | HLA-DQB2/IFI30/IRF8/IRF9/FCGR1B/TRIM22/NMI/STAT1/SOCS3/GBP1/PARP9/ARG1/OAS1/IRF7/OASL/OAS2 | 16 |
| BP | GO:0033046 | negative regulation of sister chromatid segregation | 11/706 | 42/18862 | 2.77E-07 | 3.01E-05 | 2.42E-05 | AURKB/CENPF/BUB1/ZWINT/BUB1B/MAD2L1/NDC80/PTTG1/TTK/CDC20/CCNB1 | 11 |
| BP | GO:0033048 | negative regulation of mitotic sister chromatid segregation | 11/706 | 42/18862 | 2.77E-07 | 3.01E-05 | 2.42E-05 | AURKB/CENPF/BUB1/ZWINT/BUB1B/MAD2L1/NDC80/PTTG1/TTK/CDC20/CCNB1 | 11 |
| BP | GO:2000816 | negative regulation of mitotic sister chromatid separation | 11/706 | 42/18862 | 2.77E-07 | 3.01E-05 | 2.42E-05 | AURKB/CENPF/BUB1/ZWINT/BUB1B/MAD2L1/NDC80/PTTG1/TTK/CDC20/CCNB1 | 11 |
| BP | GO:1905818 | regulation of chromosome separation | 14/706 | 71/18862 | 3.11E-07 | 3.30E-05 | 2.65E-05 | AURKB/CENPF/BUB1/PLSCR1/ZWINT/BUB1B/UBE2C/MAD2L1/NDC80/PTTG1/TTK/CDC20/DLGAP5/CCNB1 | 14 |
| BP | GO:0072330 | monocarboxylic acid biosynthetic process | 26/706 | 224/18862 | 3.27E-07 | 3.40E-05 | 2.73E-05 | ELOVL3/ACSBG1/FADS1/FA2H/FADS2/SCD5/PDK4/APOC1/CYP39A1/ADIPOQ/HPGDS/ACADL/ACOX2/FASN/LPL/CYP7B1/ELOVL7/CH25H/IDO1/DHRS9/CYP2E1/LIPG/PLA2G3/FABP5/ALDH1A3/ALOX12B | 26 |
| BP | GO:0031424 | keratinization | 26/706 | 225/18862 | 3.57E-07 | 3.63E-05 | 2.91E-05 | KRT77/KRT79/SPRR4/KRT15/LORICRIN/KRT19/TMEM79/KRT6B/IVL/CERS3/DSG3/DSC2/ABCA12/CDH3/SPRR1A/SPRR1B/KRT6A/TGM3/CNFN/TGM1/SPRR3/SPRR2G/KLK13/KRT16/LCE3D/PI3 | 26 |
| BP | GO:0072676 | lymphocyte migration | 18/706 | 118/18862 | 3.95E-07 | 3.94E-05 | 3.16E-05 | CCL27/GATA3/APOD/CCL19/CXCL11/CYP7B1/CH25H/CCL22/CCL2/PYCARD/CCR7/WNT5A/CXCL13/CCL18/CXCL10/CCL20/S100A7/C10orf99 | 18 |
| BP | GO:0051985 | negative regulation of chromosome segregation | 11/706 | 44/18862 | 4.64E-07 | 4.44E-05 | 3.57E-05 | AURKB/CENPF/BUB1/ZWINT/BUB1B/MAD2L1/NDC80/PTTG1/TTK/CDC20/CCNB1 | 11 |
| BP | GO:1905819 | negative regulation of chromosome separation | 11/706 | 44/18862 | 4.64E-07 | 4.44E-05 | 3.57E-05 | AURKB/CENPF/BUB1/ZWINT/BUB1B/MAD2L1/NDC80/PTTG1/TTK/CDC20/CCNB1 | 11 |
| BP | GO:0000280 | nuclear division | 39/706 | 436/18862 | 4.88E-07 | 4.58E-05 | 3.68E-05 | BTC/KIF2A/NUF2/CDCA5/AURKB/CENPF/KIF18B/AURKA/BUB1/TGFA/ANLN/SMPD3/KIF11/PRC1/KIF4A/CCNE2/NCAPG/NEK2/ZWINT/CCNE1/EREG/TPX2/BUB1B/KIF2C/NUSAP1/UBE2C/CDK1/MAD2L1/NDC80/PTTG1/TTK/TOP2A/MKI67/CCNB2/CDC20/DLGAP5/CKS2/WNT5A/CCNB1 | 39 |
| BP | GO:0007059 | chromosome segregation | 33/706 | 337/18862 | 4.99E-07 | 4.58E-05 | 3.68E-05 | NUF2/SPC25/CDCA5/AURKB/CENPF/CENPW/KIF18B/BUB1/CENPN/PLSCR1/ECT2/PRC1/KIF4A/CCNE2/NCAPG/NEK2/ZWINT/CCNE1/BUB1B/KIF2C/NUSAP1/FAM83D/UBE2C/MAD2L1/NDC80/PTTG1/TTK/TOP2A/MKI67/BIRC5/CDC20/DLGAP5/CCNB1 | 33 |
| BP | GO:0050792 | regulation of viral process | 23/706 | 186/18862 | 5.06E-07 | 4.58E-05 | 3.68E-05 | BCL2/IFI16/SLPI/PLSCR1/TRIM22/KPNA2/STAT1/ISG20/TRIM14/APOBEC3B/IFIT1/TMPRSS4/TOP2A/CXCR4/OAS1/MX1/LAMP3/OASL/RSAD2/ISG15/CXCL8/OAS2/LTF | 23 |
| BP | GO:0010965 | regulation of mitotic sister chromatid separation | 13/706 | 64/18862 | 5.72E-07 | 5.08E-05 | 4.08E-05 | AURKB/CENPF/BUB1/ZWINT/BUB1B/UBE2C/MAD2L1/NDC80/PTTG1/TTK/CDC20/DLGAP5/CCNB1 | 13 |
| BP | GO:0033047 | regulation of mitotic sister chromatid segregation | 11/706 | 45/18862 | 5.93E-07 | 5.18E-05 | 4.16E-05 | AURKB/CENPF/BUB1/ZWINT/BUB1B/MAD2L1/NDC80/PTTG1/TTK/CDC20/CCNB1 | 11 |
| BP | GO:0007094 | mitotic spindle assembly checkpoint | 10/706 | 37/18862 | 7.05E-07 | 5.84E-05 | 4.69E-05 | AURKB/CENPF/BUB1/ZWINT/BUB1B/MAD2L1/NDC80/TTK/CDC20/CCNB1 | 10 |
| BP | GO:0071173 | spindle assembly checkpoint | 10/706 | 37/18862 | 7.05E-07 | 5.84E-05 | 4.69E-05 | AURKB/CENPF/BUB1/ZWINT/BUB1B/MAD2L1/NDC80/TTK/CDC20/CCNB1 | 10 |
| BP | GO:0071174 | mitotic spindle checkpoint | 10/706 | 37/18862 | 7.05E-07 | 5.84E-05 | 4.69E-05 | AURKB/CENPF/BUB1/ZWINT/BUB1B/MAD2L1/NDC80/TTK/CDC20/CCNB1 | 10 |
| BP | GO:0071674 | mononuclear cell migration | 23/706 | 190/18862 | 7.40E-07 | 5.95E-05 | 4.78E-05 | CCL27/GREM1/GATA3/APOD/PDGFD/CCL19/CXCL11/CYP7B1/CH25H/CCL22/CCL2/PYCARD/CCR7/CXCR4/CXCR2/WNT5A/CXCL13/CCL18/CXCL10/CCL20/S100A7/C10orf99/S100A12 | 23 |
| BP | GO:1990266 | neutrophil migration | 18/706 | 123/18862 | 7.43E-07 | 5.95E-05 | 4.78E-05 | CCL19/CXCL11/CCL22/CCL2/NOD2/CCR7/CXCL2/CXCR2/CXCL1/CXCL9/CXCL13/CXCL8/CCL18/CXCL10/CCL20/S100A8/S100A12/S100A9 | 18 |
| BP | GO:0051306 | mitotic sister chromatid separation | 13/706 | 66/18862 | 8.31E-07 | 6.55E-05 | 5.26E-05 | AURKB/CENPF/BUB1/ZWINT/BUB1B/UBE2C/MAD2L1/NDC80/PTTG1/TTK/CDC20/DLGAP5/CCNB1 | 13 |
| BP | GO:0031577 | spindle checkpoint | 10/706 | 38/18862 | 9.25E-07 | 7.18E-05 | 5.76E-05 | AURKB/CENPF/BUB1/ZWINT/BUB1B/MAD2L1/NDC80/TTK/CDC20/CCNB1 | 10 |
| BP | GO:0048525 | negative regulation of viral process | 15/706 | 89/18862 | 9.94E-07 | 7.59E-05 | 6.10E-05 | IFI16/SLPI/PLSCR1/STAT1/ISG20/TRIM14/APOBEC3B/IFIT1/OAS1/MX1/OASL/RSAD2/ISG15/OAS2/LTF | 15 |
| BP | GO:0019058 | viral life cycle | 33/706 | 348/18862 | 1.03E-06 | 7.73E-05 | 6.21E-05 | CLDN1/EFNB2/APOE/BCL2/PIK3C2G/IFI16/SLPI/PLSCR1/TRIM22/LDLR/CCL2/KPNA2/EPHA2/CDK1/ISG20/TRIM14/APOBEC3B/IFIT1/TMPRSS4/TOP2A/CXCR4/OAS1/MX1/LAMP3/OASL/RSAD2/ISG15/CXCL8/ZC3H12A/IFI27/OAS2/LTF/SERPINB3 | 33 |
| BP | GO:1990845 | adaptive thermogenesis | 20/706 | 153/18862 | 1.15E-06 | 8.55E-05 | 6.86E-05 | PM20D1/ELOVL3/LEPR/PPARGC1A/NOVA1/HOXC10/ADRB2/PRLR/ADIPOQ/ACADL/ZNF423/PDGFC/ADRB1/CD36/IL4R/ID1/FABP5/PLAC8/CXCR4/LCN2 | 20 |
| BP | GO:0045841 | negative regulation of mitotic metaphase/anaphase transition | 10/706 | 39/18862 | 1.20E-06 | 8.77E-05 | 7.04E-05 | AURKB/CENPF/BUB1/ZWINT/BUB1B/MAD2L1/NDC80/TTK/CDC20/CCNB1 | 10 |
| BP | GO:0030071 | regulation of mitotic metaphase/anaphase transition | 12/706 | 59/18862 | 1.53E-06 | 0.00011013 | 8.84E-05 | AURKB/CENPF/BUB1/ZWINT/BUB1B/UBE2C/MAD2L1/NDC80/TTK/CDC20/DLGAP5/CCNB1 | 12 |
| BP | GO:0061436 | establishment of skin barrier | 8/706 | 24/18862 | 1.60E-06 | 0.000113225 | 9.09E-05 | FA2H/FLG2/CLDN1/TMEM79/GRHL3/ABCA12/ALOX12B/KRT16 | 8 |
| BP | GO:1902850 | microtubule cytoskeleton organization involved in mitosis | 19/706 | 143/18862 | 1.66E-06 | 0.00011482 | 9.22E-05 | SPRY2/KIF2A/NUF2/SPC25/AURKB/AURKA/CENPA/KIF11/PRC1/KIF4A/NEK2/TPX2/NUSAP1/MAD2L1/NDC80/TTK/CDC20/DLGAP5/CCNB1 | 19 |
| BP | GO:0000819 | sister chromatid segregation | 23/706 | 199/18862 | 1.67E-06 | 0.00011482 | 9.22E-05 | NUF2/CDCA5/AURKB/CENPF/KIF18B/BUB1/PRC1/KIF4A/NCAPG/NEK2/ZWINT/BUB1B/KIF2C/NUSAP1/UBE2C/MAD2L1/NDC80/PTTG1/TTK/TOP2A/CDC20/DLGAP5/CCNB1 | 23 |
| BP | GO:0106106 | cold-induced thermogenesis | 19/706 | 144/18862 | 1.85E-06 | 0.000123556 | 9.92E-05 | ELOVL3/LEPR/PPARGC1A/NOVA1/HOXC10/ADRB2/PRLR/ADIPOQ/ACADL/ZNF423/PDGFC/ADRB1/CD36/IL4R/ID1/FABP5/PLAC8/CXCR4/LCN2 | 19 |
| BP | GO:0120161 | regulation of cold-induced thermogenesis | 19/706 | 144/18862 | 1.85E-06 | 0.000123556 | 9.92E-05 | ELOVL3/LEPR/PPARGC1A/NOVA1/HOXC10/ADRB2/PRLR/ADIPOQ/ACADL/ZNF423/PDGFC/ADRB1/CD36/IL4R/ID1/FABP5/PLAC8/CXCR4/LCN2 | 19 |
| BP | GO:1902100 | negative regulation of metaphase/anaphase transition of cell cycle | 10/706 | 41/18862 | 1.98E-06 | 0.000129488 | 0.000103971 | AURKB/CENPF/BUB1/ZWINT/BUB1B/MAD2L1/NDC80/TTK/CDC20/CCNB1 | 10 |
| BP | GO:0033045 | regulation of sister chromatid segregation | 13/706 | 71/18862 | 1.99E-06 | 0.000129488 | 0.000103971 | AURKB/CENPF/BUB1/ZWINT/BUB1B/UBE2C/MAD2L1/NDC80/PTTG1/TTK/CDC20/DLGAP5/CCNB1 | 13 |
| BP | GO:0007091 | metaphase/anaphase transition of mitotic cell cycle | 12/706 | 61/18862 | 2.23E-06 | 0.00014318 | 0.000114965 | AURKB/CENPF/BUB1/ZWINT/BUB1B/UBE2C/MAD2L1/NDC80/TTK/CDC20/DLGAP5/CCNB1 | 12 |
| BP | GO:0051304 | chromosome separation | 15/706 | 95/18862 | 2.34E-06 | 0.00014822 | 0.000119012 | AURKB/CENPF/BUB1/PLSCR1/ZWINT/BUB1B/UBE2C/MAD2L1/NDC80/PTTG1/TTK/TOP2A/CDC20/DLGAP5/CCNB1 | 15 |
| BP | GO:1902099 | regulation of metaphase/anaphase transition of cell cycle | 12/706 | 62/18862 | 2.67E-06 | 0.000167165 | 0.000134223 | AURKB/CENPF/BUB1/ZWINT/BUB1B/UBE2C/MAD2L1/NDC80/TTK/CDC20/DLGAP5/CCNB1 | 12 |
| BP | GO:0097530 | granulocyte migration | 19/706 | 148/18862 | 2.80E-06 | 0.000173037 | 0.000138938 | CCL19/CXCL11/CCL22/CCL2/NOD2/CCR7/CXCL2/CXCR2/CXCL1/CXCL9/CXCL13/CXCL8/CCL18/CXCL10/CCL20/S100A8/S100A7/S100A12/S100A9 | 19 |
| BP | GO:0120162 | positive regulation of cold-induced thermogenesis | 15/706 | 97/18862 | 3.06E-06 | 0.00018662 | 0.000149844 | ELOVL3/LEPR/PPARGC1A/ADRB2/PRLR/ADIPOQ/ACADL/PDGFC/ADRB1/CD36/IL4R/FABP5/PLAC8/CXCR4/LCN2 | 15 |
| BP | GO:0033561 | regulation of water loss via skin | 8/706 | 26/18862 | 3.18E-06 | 0.000191702 | 0.000153925 | FA2H/FLG2/CLDN1/TMEM79/GRHL3/ABCA12/ALOX12B/KRT16 | 8 |
| BP | GO:0044784 | metaphase/anaphase transition of cell cycle | 12/706 | 64/18862 | 3.79E-06 | 0.00022575 | 0.000181263 | AURKB/CENPF/BUB1/ZWINT/BUB1B/UBE2C/MAD2L1/NDC80/TTK/CDC20/DLGAP5/CCNB1 | 12 |
| BP | GO:0000302 | response to reactive oxygen species | 24/706 | 224/18862 | 3.84E-06 | 0.000225796 | 0.0001813 | PPARGC1A/APOD/CRYAB/FOS/RHOB/PDGFD/TPM1/APOE/BCL2/CCL19/CD36/EZH2/HMOX1/FOSL1/ECT2/SMPD3/GCH1/CYP2E1/CCNA2/PPIF/MMP9/CCR7/ARG1/S100A7 | 24 |
| BP | GO:0098813 | nuclear chromosome segregation | 27/706 | 273/18862 | 4.48E-06 | 0.000260696 | 0.000209323 | NUF2/CDCA5/AURKB/CENPF/KIF18B/BUB1/ECT2/PRC1/KIF4A/CCNE2/NCAPG/NEK2/ZWINT/CCNE1/BUB1B/KIF2C/NUSAP1/FAM83D/UBE2C/MAD2L1/NDC80/PTTG1/TTK/TOP2A/CDC20/DLGAP5/CCNB1 | 27 |
| BP | GO:0042119 | neutrophil activation | 40/706 | 500/18862 | 5.67E-06 | 0.000325738 | 0.000261547 | FLG2/MGST1/CTSC/CD36/SLPI/SERPINA3/AMPD3/PRSS2/S100P/PYCARD/FPR1/SERPINA1/PGM2/FCGR3B/FABP5/SELL/DNASE1L3/PLAC8/MMP9/GM2A/PRSS3/VNN1/GGH/SERPINB1/ACP3/ARG1/PNP/LRG1/CXCR2/CXCL1/CXCL8/S100A8/LCN2/HPSE/LTF/S100A7/SERPINB3/S100A12/S100A9/TCN1 | 40 |
| BP | GO:2000107 | negative regulation of leukocyte apoptotic process | 10/706 | 46/18862 | 6.09E-06 | 0.000345804 | 0.000277659 | PIP/SLC46A2/CCL19/AURKB/IDO1/IL7R/PRKCQ/NOD2/CCR7/IRF7 | 10 |
| BP | GO:0046394 | carboxylic acid biosynthetic process | 30/706 | 327/18862 | 6.16E-06 | 0.000345935 | 0.000277765 | ELOVL3/ACSBG1/FADS1/FA2H/FADS2/SCD5/PDK4/APOC1/CYP39A1/ADIPOQ/HPGDS/ACADL/ACOX2/FASN/LPL/PSPH/CYP7B1/ELOVL7/CH25H/IDO1/DHRS9/AASS/CYP2E1/LIPG/GPT2/PLA2G3/FABP5/ALDH1A3/ALOX12B/KYNU | 30 |
| BP | GO:0006959 | humoral immune response | 33/706 | 380/18862 | 6.93E-06 | 0.000384672 | 0.000308868 | MUC7/GATA3/ANG/BCL2/PGLYRP4/CXCL11/BLNK/C1QB/SLPI/TNFRSF21/PRSS2/CCL2/NOD2/RNASE7/PRSS3/CCR7/CXCL2/CFB/WFDC12/KRT6A/CXCL1/CXCL9/CXCL13/CXCL8/IL36RN/CXCL10/S100A8/LCN2/LTF/S100A7/S100A12/S100A9/PI3 | 33 |
| BP | GO:0045839 | negative regulation of mitotic nuclear division | 10/706 | 47/18862 | 7.47E-06 | 0.000410349 | 0.000329485 | AURKB/CENPF/BUB1/ZWINT/BUB1B/MAD2L1/NDC80/TTK/CDC20/CCNB1 | 10 |
| BP | GO:0002283 | neutrophil activation involved in immune response | 39/706 | 488/18862 | 7.59E-06 | 0.000412141 | 0.000330924 | FLG2/MGST1/CTSC/CD36/SLPI/SERPINA3/AMPD3/PRSS2/S100P/PYCARD/FPR1/SERPINA1/PGM2/FCGR3B/FABP5/SELL/DNASE1L3/PLAC8/MMP9/GM2A/PRSS3/VNN1/GGH/SERPINB1/ACP3/ARG1/PNP/LRG1/CXCR2/CXCL1/S100A8/LCN2/HPSE/LTF/S100A7/SERPINB3/S100A12/S100A9/TCN1 | 39 |
| BP | GO:2000106 | regulation of leukocyte apoptotic process | 13/706 | 80/18862 | 7.91E-06 | 0.00042474 | 0.00034104 | PIP/SLC46A2/CCL19/AURKB/IDO1/IL7R/PRKCQ/NOD2/CCR7/SLC7A11/IRF7/WNT5A/CD274 | 13 |
| BP | GO:0050679 | positive regulation of epithelial cell proliferation | 22/706 | 203/18862 | 8.01E-06 | 0.000425611 | 0.00034174 | F3/AGTR1/OSR2/PTN/CLDN1/AR/ANG/CCND1/ESRP2/CYP7B1/HMOX1/TGFA/AREG/ID1/NOD2/FGFBP1/CDH3/ARG1/LRG1/WNT5A/TGM1/MMP12 | 22 |
| BP | GO:0051346 | negative regulation of hydrolase activity | 37/706 | 456/18862 | 9.25E-06 | 0.000485804 | 0.000390071 | SERPINA12/LEPR/CST6/PTN/ARFGEF3/PAPLN/APOC1/PPP1R1B/CRYAB/PLA2R1/PLN/SPRY2/TIMP3/SLC27A4/IFI16/SERPINB8/SERPINB9/ANGPTL4/SLPI/SERPINA3/SERPINA1/GZMA/PPIF/MMP9/IFIT1/PI15/PTTG1/SERPINB1/WFDC12/LAMP3/IFI6/SERPINB13/A2ML1/LTF/SERPINB3/PI3/SERPINB4 | 37 |
| BP | GO:0016053 | organic acid biosynthetic process | 30/706 | 335/18862 | 9.92E-06 | 0.000515596 | 0.000413992 | ELOVL3/ACSBG1/FADS1/FA2H/FADS2/SCD5/PDK4/APOC1/CYP39A1/ADIPOQ/HPGDS/ACADL/ACOX2/FASN/LPL/PSPH/CYP7B1/ELOVL7/CH25H/IDO1/DHRS9/AASS/CYP2E1/LIPG/GPT2/PLA2G3/FABP5/ALDH1A3/ALOX12B/KYNU | 30 |
| BP | GO:0051383 | kinetochore organization | 7/706 | 22/18862 | 1.04E-05 | 0.000535863 | 0.000430265 | NUF2/CENPF/CENPW/CENPN/CENPA/NDC80/DLGAP5 | 7 |
| BP | GO:0032649 | regulation of interferon-gamma production | 15/706 | 107/18862 | 1.05E-05 | 0.000536084 | 0.000430442 | GATA3/IRF8/RASGRP1/FZD5/PYCARD/CD2/NOD2/SLC7A5/CCR7/CLEC7A/WNT5A/ISG15/CD274/IL36RN/ZC3H12A | 15 |
| BP | GO:0006979 | response to oxidative stress | 36/706 | 444/18862 | 1.24E-05 | 0.000623985 | 0.000501022 | PPARGC1A/APOD/MGST1/ADIPOQ/CRYAB/FOS/RHOB/PDGFD/ACOX2/PLA2R1/TPM1/APOE/BCL2/MSRB3/CCL19/SLC23A2/CD36/EZH2/HMOX1/FOSL1/ECT2/SMPD3/GCH1/CYP2E1/CCNA2/PPIF/GPX2/MMP9/CCR7/VNN1/MELK/ARG1/SLC7A11/GJB2/ZC3H12A/S100A7 | 36 |
| BP | GO:0002446 | neutrophil mediated immunity | 39/706 | 499/18862 | 1.27E-05 | 0.000633629 | 0.000508765 | FLG2/MGST1/CTSC/CD36/SLPI/SERPINA3/AMPD3/PRSS2/S100P/PYCARD/FPR1/SERPINA1/PGM2/FCGR3B/FABP5/SELL/DNASE1L3/PLAC8/MMP9/GM2A/PRSS3/VNN1/GGH/SERPINB1/ACP3/ARG1/PNP/LRG1/CXCR2/CXCL1/S100A8/LCN2/HPSE/LTF/S100A7/SERPINB3/S100A12/S100A9/TCN1 | 39 |
| BP | GO:0050678 | regulation of epithelial cell proliferation | 32/706 | 374/18862 | 1.29E-05 | 0.000635319 | 0.000510122 | F3/AGTR1/OSR2/PTN/GATA3/CLDN1/AR/ANG/TNMD/APOE/TGFBR3/CCND1/PPARD/ESRP2/CYP7B1/HMOX1/TGFA/AREG/ID1/CCL2/EREG/STAT1/NOD2/UHRF1/FUT2/FGFBP1/CDH3/ARG1/LRG1/WNT5A/TGM1/MMP12 | 32 |
| BP | GO:0042493 | response to drug | 31/706 | 359/18862 | 1.46E-05 | 0.000714985 | 0.000574089 | GAL/EMX2/PTN/PPARGC1A/GATA3/APOD/MGST1/ADIPOQ/FOS/MAP1B/LPL/BCL2/BCAR3/CCND1/TFAP2B/CTPS1/CENPF/HMOX1/FOSL1/CYP2C18/AIM2/LRP8/CYP2E1/TYMS/LCK/GGH/CDH3/ARG1/PNP/CXCR4/CCNB1 | 31 |
| BP | GO:0043312 | neutrophil degranulation | 38/706 | 485/18862 | 1.55E-05 | 0.000750279 | 0.000602428 | FLG2/MGST1/CTSC/CD36/SLPI/SERPINA3/AMPD3/PRSS2/S100P/PYCARD/FPR1/SERPINA1/PGM2/FCGR3B/FABP5/SELL/PLAC8/MMP9/GM2A/PRSS3/VNN1/GGH/SERPINB1/ACP3/ARG1/PNP/LRG1/CXCR2/CXCL1/S100A8/LCN2/HPSE/LTF/S100A7/SERPINB3/S100A12/S100A9/TCN1 | 38 |
| BP | GO:0048732 | gland development | 34/706 | 413/18862 | 1.57E-05 | 0.00075173 | 0.000603593 | FA2H/SLC46A2/PTN/COBL/GATA3/CLDN1/AR/PRLR/PSAPL1/CRIP1/ESR1/SLC29A1/FASN/HOXB3/FRZB/TGFBR3/BCL2/CCND1/IGFBP5/ESRP2/EZH2/CYP7B1/HMOX1/AURKA/E2F8/EPHA2/TYMS/HK2/ALDH1A3/ZDHHC21/CCNB2/ARG1/WNT5A/OAS2 | 34 |
| BP | GO:0006633 | fatty acid biosynthetic process | 19/706 | 168/18862 | 1.80E-05 | 0.00085179 | 0.000683935 | ELOVL3/ACSBG1/FADS1/FA2H/FADS2/SCD5/PDK4/APOC1/ADIPOQ/HPGDS/ACADL/FASN/LPL/ELOVL7/CYP2E1/LIPG/PLA2G3/FABP5/ALOX12B | 19 |
| BP | GO:0032609 | interferon-gamma production | 15/706 | 112/18862 | 1.84E-05 | 0.000866555 | 0.00069579 | GATA3/IRF8/RASGRP1/FZD5/PYCARD/CD2/NOD2/SLC7A5/CCR7/CLEC7A/WNT5A/ISG15/CD274/IL36RN/ZC3H12A | 15 |
| BP | GO:0048662 | negative regulation of smooth muscle cell proliferation | 11/706 | 63/18862 | 1.95E-05 | 0.0009074 | 0.000728586 | OGN/PPARGC1A/APOD/MYOCD/ANG/ADIPOQ/CNN1/TPM1/IGFBP5/PPARD/HMOX1 | 11 |
| BP | GO:0050727 | regulation of inflammatory response | 31/706 | 366/18862 | 2.14E-05 | 0.000984117 | 0.000790185 | IL37/AGTR1/GATA3/RORA/ADIPOQ/ESR1/LPL/APOE/CTSC/PPARD/MFHAS1/IL20/NMI/IDO1/DUOXA1/LDLR/PYCARD/F12/PLA2G3/DNASE1L3/SELE/MMP9/NFKBIZ/NOD2/SOCS3/CCR7/PBK/WNT5A/S100A8/S100A12/S100A9 | 31 |
| BP | GO:0001659 | temperature homeostasis | 19/706 | 171/18862 | 2.31E-05 | 0.001053787 | 0.000846127 | ELOVL3/LEPR/PPARGC1A/NOVA1/HOXC10/ADRB2/PRLR/ADIPOQ/ACADL/ZNF423/PDGFC/ADRB1/CD36/IL4R/ID1/FABP5/PLAC8/CXCR4/LCN2 | 19 |
| BP | GO:2000404 | regulation of T cell migration | 9/706 | 43/18862 | 2.46E-05 | 0.001115088 | 0.000895347 | CCL27/APOD/PYCARD/WNT5A/CXCL13/CXCL10/CCL20/S100A7/C10orf99 | 9 |
| BP | GO:0071887 | leukocyte apoptotic process | 14/706 | 102/18862 | 2.63E-05 | 0.001179253 | 0.000946868 | PIP/SLC46A2/CCL19/AURKB/TNFRSF21/IDO1/IL7R/PRKCQ/NOD2/CCR7/SLC7A11/IRF7/WNT5A/CD274 | 14 |
| BP | GO:0051784 | negative regulation of nuclear division | 10/706 | 54/18862 | 2.73E-05 | 0.00121144 | 0.000972712 | AURKB/CENPF/BUB1/ZWINT/BUB1B/MAD2L1/NDC80/TTK/CDC20/CCNB1 | 10 |
| BP | GO:0072678 | T cell migration | 11/706 | 66/18862 | 3.07E-05 | 0.001352169 | 0.001085709 | CCL27/APOD/CXCL11/CCL2/PYCARD/WNT5A/CXCL13/CXCL10/CCL20/S100A7/C10orf99 | 11 |
| BP | GO:0048660 | regulation of smooth muscle cell proliferation | 18/706 | 160/18862 | 3.18E-05 | 0.001380291 | 0.001108289 | OGN/PPARGC1A/APOD/MYOCD/ANG/ADIPOQ/CNN1/PDGFD/ELN/TPM1/IGFBP5/PPARD/HMOX1/SMPD3/HBEGF/EREG/STAT1/MMP9 | 18 |
| BP | GO:0051897 | positive regulation of protein kinase B signaling | 19/706 | 175/18862 | 3.19E-05 | 0.001380291 | 0.001108289 | BTC/F3/GATA3/ESR1/TSPYL5/SPRY2/MYOC/IGFBP5/CCL19/MFHAS1/TGFA/HBEGF/AREG/TPBG/EREG/LCK/CCR7/FAM110C/HPSE | 19 |
| BP | GO:0007052 | mitotic spindle organization | 15/706 | 118/18862 | 3.45E-05 | 0.001480795 | 0.001188987 | KIF2A/NUF2/SPC25/AURKB/AURKA/KIF11/PRC1/KIF4A/NEK2/TPX2/NDC80/TTK/CDC20/DLGAP5/CCNB1 | 15 |
| BP | GO:0018149 | peptide cross-linking | 8/706 | 35/18862 | 3.55E-05 | 0.001509313 | 0.001211886 | LORICRIN/IVL/SPRR1A/SPRR1B/TGM3/TGM1/SPRR3/PI3 | 8 |
| BP | GO:0048659 | smooth muscle cell proliferation | 18/706 | 162/18862 | 3.76E-05 | 0.001583874 | 0.001271753 | OGN/PPARGC1A/APOD/MYOCD/ANG/ADIPOQ/CNN1/PDGFD/ELN/TPM1/IGFBP5/PPARD/HMOX1/SMPD3/HBEGF/EREG/STAT1/MMP9 | 18 |
| BP | GO:0032731 | positive regulation of interleukin-1 beta production | 10/706 | 56/18862 | 3.79E-05 | 0.001584186 | 0.001272004 | LPL/TRIM16/IFI16/CCL19/FZD5/AIM2/PYCARD/NOD2/CLEC7A/WNT5A | 10 |
| BP | GO:0010466 | negative regulation of peptidase activity | 24/706 | 257/18862 | 3.86E-05 | 0.00159699 | 0.001282285 | SERPINA12/CST6/PAPLN/CRYAB/TIMP3/IFI16/SERPINB8/SERPINB9/SLPI/SERPINA3/SERPINA1/MMP9/PI15/PTTG1/SERPINB1/WFDC12/LAMP3/IFI6/SERPINB13/A2ML1/LTF/SERPINB3/PI3/SERPINB4 | 24 |
| BP | GO:0003012 | muscle system process | 35/706 | 453/18862 | 4.45E-05 | 0.001826699 | 0.001466727 | DES/SORBS1/ACTG2/SNTB1/PPARGC1A/SCN7A/ADRB2/CACNA2D1/MYH11/MYOCD/LMOD1/TPM2/SSPN/MYL9/DMD/CRYAB/SYNM/CNN1/ATP1A2/TPM1/PLN/MYLK/FBXO32/MYOC/IGFBP5/GLRX3/EZH2/HMOX1/LTB4R/SCO2/COMP/DSC2/ZDHHC21/CXCR4/ZC3H12A | 35 |
| BP | GO:0071900 | regulation of protein serine/threonine kinase activity | 37/706 | 492/18862 | 4.82E-05 | 0.001948758 | 0.001564733 | CAB39L/ADRB2/MYOCD/ADIPOQ/IGFBP6/PKIB/PDGFD/PDGFC/SPRY2/APOE/CCND1/CCL19/EZH2/MAP3K9/RASGRP1/FZD5/TGFA/CCNE2/PYCARD/FPR1/CCNE1/CCNA2/CD24/IRAK2/CDK1/NOD2/CDKN3/CCNB2/PBK/CXCR4/CKS2/WNT5A/CCNB1/CXCL10/LTF/SERPINB3/S100A12 | 37 |
| BP | GO:0008202 | steroid metabolic process | 28/706 | 329/18862 | 4.86E-05 | 0.001948758 | 0.001564733 | GAL/HSD11B1/HSD3B1/SERPINA12/LEPR/AGTR1/PPARGC1A/APOC1/CYP39A1/RORA/PRLR/HMGCS2/ESR1/ACADL/ACOX2/FASN/APOE/SQLE/CYB5R2/PPARD/ABCG1/CYP7B1/CH25H/HSD17B2/LDLR/DHRS9/APOL1/CYP2E1 | 28 |
| BP | GO:0052547 | regulation of peptidase activity | 35/706 | 455/18862 | 4.87E-05 | 0.001948758 | 0.001564733 | SERPINA12/F3/CST6/PAPLN/CRYAB/FBLN1/PCOLCE2/TIMP3/IFI16/SERPINB8/SERPINB9/SLPI/SERPINA3/AIM2/SOX7/PSME2/PYCARD/SERPINA1/LCK/MMP9/PI15/PTTG1/SERPINB1/CLEC7A/WFDC12/LAMP3/IFI6/SERPINB13/A2ML1/S100A8/LTF/SERPINB3/S100A9/PI3/SERPINB4 | 35 |
| BP | GO:0032611 | interleukin-1 beta production | 14/706 | 108/18862 | 5.03E-05 | 0.001997876 | 0.001604172 | LPL/TRIM16/IFI16/CCL19/CD36/FZD5/AIM2/PYCARD/NOD2/CCR7/SERPINB1/CLEC7A/WNT5A/ZC3H12A | 14 |
| BP | GO:0010951 | negative regulation of endopeptidase activity | 23/706 | 245/18862 | 5.12E-05 | 0.002018379 | 0.001620634 | SERPINA12/CST6/PAPLN/CRYAB/TIMP3/IFI16/SERPINB8/SERPINB9/SLPI/SERPINA3/SERPINA1/MMP9/PTTG1/SERPINB1/WFDC12/LAMP3/IFI6/SERPINB13/A2ML1/LTF/SERPINB3/PI3/SERPINB4 | 23 |
| BP | GO:0032602 | chemokine production | 13/706 | 95/18862 | 5.24E-05 | 0.00204677 | 0.001643431 | POSTN/APOD/ADIPOQ/LPL/HMOX1/IL4R/PYCARD/EPHA2/NOD2/CLEC7A/WNT5A/S100A8/S100A9 | 13 |
| BP | GO:0019216 | regulation of lipid metabolic process | 32/706 | 402/18862 | 5.32E-05 | 0.002063523 | 0.001656882 | THRSP/GAL/SERPINA12/FADS1/AGTR1/SORBS1/PDK4/PPARGC1A/APOC1/APOD/RORA/ADIPOQ/HMGCS2/PSAPL1/ACADL/FASN/APOE/SQLE/PPARD/CCL19/ABCG1/CD36/ANGPTL4/SERPINA3/RAB38/SMPD3/TNFRSF21/LDLR/PLA2G3/FABP5/NOD2/CCR7 | 32 |
| BP | GO:0006575 | cellular modified amino acid metabolic process | 20/706 | 198/18862 | 5.64E-05 | 0.00216828 | 0.001740996 | CRAT/ABHD12B/GATA3/GSTA3/HPGDS/ACADL/PLA2G2F/ETHE1/PLSCR1/GCH1/DUOXA1/TYMS/PLA2G3/CHAC2/SLC16A10/CHAC1/VNN1/GGH/SLC7A11/VNN3 | 20 |
| BP | GO:0090303 | positive regulation of wound healing | 10/706 | 59/18862 | 6.05E-05 | 0.002309474 | 0.001854366 | F3/CLDN1/MYLK/CD36/FERMT1/HBEGF/F12/CLEC7A/CXCR4/HPSE | 10 |
| BP | GO:0006721 | terpenoid metabolic process | 15/706 | 124/18862 | 6.19E-05 | 0.002345966 | 0.001883667 | RBP4/ADH1B/HMGCS2/LPL/SDC2/APOE/PPARD/RDH12/CYP2C18/LRP8/DHRS9/CYP2E1/ALDH1A3/CRABP2/AKR1B10 | 15 |
| BP | GO:0070372 | regulation of ERK1 and ERK2 cascade | 26/706 | 301/18862 | 7.00E-05 | 0.002630348 | 0.002112008 | ADIPOQ/MTURN/CAMK2N1/PDGFD/PDGFC/FBLN1/SPRY2/APOE/TIMP3/CCL19/MFHAS1/CD36/RASGRP1/CCL22/CCL2/PYCARD/TPBG/EPHA2/FAM83D/NOD2/GBP1/CCR7/CHRNA9/CCL18/CCL20/S100A7 | 26 |
| BP | GO:1903036 | positive regulation of response to wounding | 11/706 | 72/18862 | 7.05E-05 | 0.002630348 | 0.002112008 | F3/PTN/CLDN1/MYLK/CD36/FERMT1/HBEGF/F12/CLEC7A/CXCR4/HPSE | 11 |
| BP | GO:0045741 | positive regulation of epidermal growth factor-activated receptor activity | 5/706 | 13/18862 | 7.26E-05 | 0.002686069 | 0.002156748 | BTC/TGFA/HBEGF/AREG/EREG | 5 |
| BP | GO:0097529 | myeloid leukocyte migration | 21/706 | 218/18862 | 7.41E-05 | 0.002722725 | 0.002186181 | GREM1/PDGFD/CCL19/CXCL11/CCL22/CCL2/NOD2/CCR7/CXCL2/CXCR2/CXCL1/CXCL9/CXCL13/CXCL8/CCL18/CXCL10/CCL20/S100A8/S100A7/S100A12/S100A9 | 21 |
| BP | GO:0021700 | developmental maturation | 24/706 | 268/18862 | 7.51E-05 | 0.002739428 | 0.002199592 | GAL/GREM1/GATA3/ANG/SYBU/MAP1B/BCL2/GLDN/MYOC/CCL19/TMEM79/FZD5/AURKA/RAB38/FERMT1/EREG/TYMS/PLA2G3/CDH3/CDC20/CLEC7A/WNT5A/CCNB1/LTF | 24 |
| BP | GO:0045742 | positive regulation of epidermal growth factor receptor signaling pathway | 8/706 | 39/18862 | 8.14E-05 | 0.00292293 | 0.002346933 | BTC/BCAR3/TGFA/HBEGF/AREG/AFAP1L2/EREG/MMP9 | 8 |
| BP | GO:0050832 | defense response to fungus | 8/706 | 39/18862 | 8.14E-05 | 0.00292293 | 0.002346933 | RNASE7/ARG1/IL36RN/S100A8/LTF/C10orf99/S100A12/S100A9 | 8 |
| BP | GO:0002685 | regulation of leukocyte migration | 20/706 | 205/18862 | 9.16E-05 | 0.003259935 | 0.002617527 | CCL27/GREM1/PTN/APOD/PDGFD/CCL19/HMOX1/SMPD3/CCL2/PYCARD/SELE/NOD2/CCR7/WNT5A/CXCL13/CXCL8/CXCL10/CCL20/S100A7/C10orf99 | 20 |
| BP | GO:0006720 | isoprenoid metabolic process | 16/706 | 143/18862 | 9.21E-05 | 0.003259935 | 0.002617527 | RBP4/ADH1B/HMGCS2/LPL/SDC2/APOE/PPARD/RDH12/CYP2C18/LRP8/PDSS1/DHRS9/CYP2E1/ALDH1A3/CRABP2/AKR1B10 | 16 |
| BP | GO:0150104 | transport across blood-brain barrier | 12/706 | 87/18862 | 9.33E-05 | 0.003279358 | 0.002633123 | LEPR/ATP1A2/SLC29A1/SLC2A13/APOE/SLC27A4/CD36/SLC7A1/SLC16A1/FABP5/SLC7A5/SLC5A1 | 12 |
| BP | GO:2000406 | positive regulation of T cell migration | 7/706 | 30/18862 | 9.58E-05 | 0.003332871 | 0.002676091 | CCL27/PYCARD/WNT5A/CXCL13/CXCL10/CCL20/S100A7 | 7 |
| BP | GO:0033002 | muscle cell proliferation | 21/706 | 222/18862 | 9.62E-05 | 0.003332871 | 0.002676091 | OGN/RBP4/PPARGC1A/APOD/MYOCD/ANG/ADIPOQ/CNN1/PDGFD/ELN/TPM1/TGFBR3/IGFBP5/PPARD/HMOX1/SMPD3/HBEGF/EREG/STAT1/MMP9/CCNB1 | 21 |
| BP | GO:0032735 | positive regulation of interleukin-12 production | 8/706 | 40/18862 | 9.84E-05 | 0.003385613 | 0.002718439 | CCL19/LTB/CD36/IRF8/IDO1/NOD2/CCR7/CLEC7A | 8 |
| BP | GO:0010232 | vascular transport | 12/706 | 88/18862 | 0.000104408 | 0.00356739 | 0.002864395 | LEPR/ATP1A2/SLC29A1/SLC2A13/APOE/SLC27A4/CD36/SLC7A1/SLC16A1/FABP5/SLC7A5/SLC5A1 | 12 |
| BP | GO:2000401 | regulation of lymphocyte migration | 10/706 | 63/18862 | 0.000107575 | 0.003650096 | 0.002930803 | CCL27/APOD/CCL2/PYCARD/WNT5A/CXCL13/CXCL10/CCL20/S100A7/C10orf99 | 10 |
| BP | GO:0045861 | negative regulation of proteolysis | 28/706 | 346/18862 | 0.000116181 | 0.003914915 | 0.003143436 | SERPINA12/CST6/PAPLN/CRYAB/CAMK2N1/TIMP3/IFI16/SERPINB8/SERPINB9/SLPI/SERPINA3/SERPINA1/MMP9/PI15/PTTG1/CHAC1/SERPINB1/PBK/PLAT/WFDC12/LAMP3/IFI6/SERPINB13/A2ML1/LTF/SERPINB3/PI3/SERPINB4 | 28 |
| BP | GO:1901186 | positive regulation of ERBB signaling pathway | 8/706 | 41/18862 | 0.000118279 | 0.003958293 | 0.003178266 | BTC/BCAR3/TGFA/HBEGF/AREG/AFAP1L2/EREG/MMP9 | 8 |
| BP | GO:0071902 | positive regulation of protein serine/threonine kinase activity | 26/706 | 311/18862 | 0.000119618 | 0.003975879 | 0.003192387 | CAB39L/ADRB2/ADIPOQ/IGFBP6/PDGFD/PDGFC/SPRY2/CCND1/CCL19/EZH2/MAP3K9/RASGRP1/FZD5/TGFA/FPR1/CD24/IRAK2/CDK1/NOD2/PBK/CXCR4/CKS2/WNT5A/CCNB1/LTF/S100A12 | 26 |
| BP | GO:0032651 | regulation of interleukin-1 beta production | 13/706 | 103/18862 | 0.000122148 | 0.00403252 | 0.003237866 | LPL/TRIM16/IFI16/CCL19/FZD5/AIM2/PYCARD/NOD2/CCR7/SERPINB1/CLEC7A/WNT5A/ZC3H12A | 13 |
| BP | GO:0032732 | positive regulation of interleukin-1 production | 10/706 | 64/18862 | 0.000123282 | 0.004042653 | 0.003246002 | LPL/TRIM16/IFI16/CCL19/FZD5/AIM2/PYCARD/NOD2/CLEC7A/WNT5A | 10 |
| BP | GO:0001906 | cell killing | 18/706 | 179/18862 | 0.000138225 | 0.004488253 | 0.003603791 | MUC7/PGLYRP4/CTSC/RAET1E/SERPINB9/RASGRP1/IL7R/APOL1/ULBP2/DNASE1L3/SLAMF7/ARG1/CLEC7A/KRT6A/GZMB/LTF/S100A12/SERPINB4 | 18 |
| BP | GO:0009620 | response to fungus | 9/706 | 53/18862 | 0.000138708 | 0.004488253 | 0.003603791 | RNASE7/ARG1/CLEC7A/IL36RN/S100A8/LTF/C10orf99/S100A12/S100A9 | 9 |
| BP | GO:2001251 | negative regulation of chromosome organization | 12/706 | 91/18862 | 0.00014483 | 0.004641943 | 0.003727195 | AURKB/CENPF/BUB1/ZWINT/BUB1B/MAD2L1/NDC80/PTTG1/TTK/TOP2A/CDC20/CCNB1 | 12 |
| BP | GO:1903555 | regulation of tumor necrosis factor superfamily cytokine production | 17/706 | 164/18862 | 0.000145358 | 0.004641943 | 0.003727195 | IL37/CD34/ADIPOQ/LPL/CCL19/CD36/RASGRP1/FZD5/PYCARD/CD2/IFIH1/NOD2/CLEC7A/WNT5A/CD274/ZC3H12A/LTF | 17 |
| BP | GO:0042303 | molting cycle | 13/706 | 105/18862 | 0.000148795 | 0.0046904 | 0.003766103 | GAL/FA2H/LGR5/BCL2/IGFBP5/TMEM79/FERMT1/ZDHHC21/CDH3/WNT5A/TGM3/KRT16/HPSE | 13 |
| BP | GO:0042633 | hair cycle | 13/706 | 105/18862 | 0.000148795 | 0.0046904 | 0.003766103 | GAL/FA2H/LGR5/BCL2/IGFBP5/TMEM79/FERMT1/ZDHHC21/CDH3/WNT5A/TGM3/KRT16/HPSE | 13 |
| BP | GO:0052548 | regulation of endopeptidase activity | 32/706 | 426/18862 | 0.000157024 | 0.004918062 | 0.003948902 | SERPINA12/F3/CST6/PAPLN/CRYAB/TIMP3/IFI16/SERPINB8/SERPINB9/SLPI/SERPINA3/AIM2/SOX7/PSME2/PYCARD/SERPINA1/LCK/MMP9/PTTG1/SERPINB1/CLEC7A/WFDC12/LAMP3/IFI6/SERPINB13/A2ML1/S100A8/LTF/SERPINB3/S100A9/PI3/SERPINB4 | 32 |
| BP | GO:0030104 | water homeostasis | 11/706 | 79/18862 | 0.000166387 | 0.005178119 | 0.004157711 | AQP9/FA2H/FLG2/CLDN1/ADCY2/TFAP2B/TMEM79/GRHL3/ABCA12/ALOX12B/KRT16 | 11 |
| BP | GO:2000273 | positive regulation of signaling receptor activity | 8/706 | 43/18862 | 0.000168046 | 0.00519509 | 0.004171338 | BTC/GREM1/ADRB2/TGFA/HBEGF/AREG/CCL2/EREG | 8 |
| BP | GO:1901136 | carbohydrate derivative catabolic process | 19/706 | 198/18862 | 0.000169058 | 0.00519509 | 0.004171338 | OGN/LYVE1/OMD/SDC2/PGLYRP4/AMPD3/FBXO6/NT5C3A/PGM2/HMMR/APOBEC3B/GM2A/HYAL4/PNP/UPP1/TYMP/MMP12/CHI3L2/HPSE | 19 |
| BP | GO:0007051 | spindle organization | 18/706 | 182/18862 | 0.000170547 | 0.005208089 | 0.004181776 | TPPP/KIF2A/NUF2/SPC25/AURKB/AURKA/KIF11/PRC1/KIF4A/NEK2/TPX2/NDC80/TTK/ASPM/CCNB2/CDC20/DLGAP5/CCNB1 | 18 |
| BP | GO:0006631 | fatty acid metabolic process | 30/706 | 392/18862 | 0.000180955 | 0.005430693 | 0.004360513 | PM20D1/ELOVL3/ACSBG1/FADS1/FA2H/FADS2/CRAT/SCD5/PDK4/HAO2/PPARGC1A/APOC1/ADIPOQ/HPGDS/ACADL/ACOX2/PNPLA3/FASN/LPL/SLC27A4/PPARD/PLA2G2F/CD36/ELOVL7/CYP2C18/CYP2E1/LIPG/PLA2G3/FABP5/ALOX12B | 30 |
| BP | GO:0071706 | tumor necrosis factor superfamily cytokine production | 17/706 | 167/18862 | 0.000181176 | 0.005430693 | 0.004360513 | IL37/CD34/ADIPOQ/LPL/CCL19/CD36/RASGRP1/FZD5/PYCARD/CD2/IFIH1/NOD2/CLEC7A/WNT5A/CD274/ZC3H12A/LTF | 17 |
| BP | GO:0034368 | protein-lipid complex remodeling | 7/706 | 33/18862 | 0.000182283 | 0.005430693 | 0.004360513 | AGTR1/APOC1/LPL/APOE/ABCG1/LIPG/PLA2G3 | 7 |
| BP | GO:0034369 | plasma lipoprotein particle remodeling | 7/706 | 33/18862 | 0.000182283 | 0.005430693 | 0.004360513 | AGTR1/APOC1/LPL/APOE/ABCG1/LIPG/PLA2G3 | 7 |
| BP | GO:0001942 | hair follicle development | 11/706 | 80/18862 | 0.000186499 | 0.005522629 | 0.004434332 | GAL/LGR5/BCL2/IGFBP5/TMEM79/FERMT1/ZDHHC21/CDH3/WNT5A/TGM3/HPSE | 11 |
| BP | GO:0070371 | ERK1 and ERK2 cascade | 26/706 | 320/18862 | 0.000188874 | 0.005559271 | 0.004463753 | ADIPOQ/MTURN/CAMK2N1/PDGFD/PDGFC/FBLN1/SPRY2/APOE/TIMP3/CCL19/MFHAS1/CD36/RASGRP1/CCL22/CCL2/PYCARD/TPBG/EPHA2/FAM83D/NOD2/GBP1/CCR7/CHRNA9/CCL18/CCL20/S100A7 | 26 |
| BP | GO:0001523 | retinoid metabolic process | 13/706 | 108/18862 | 0.000198088 | 0.00579555 | 0.004653471 | RBP4/ADH1B/LPL/SDC2/APOE/PPARD/RDH12/CYP2C18/LRP8/DHRS9/ALDH1A3/CRABP2/AKR1B10 | 13 |
| BP | GO:0071695 | anatomical structure maturation | 20/706 | 218/18862 | 0.000211253 | 0.006143945 | 0.004933211 | GAL/GREM1/GATA3/ANG/BCL2/GLDN/MYOC/CCL19/TMEM79/FZD5/AURKA/FERMT1/EREG/TYMS/PLA2G3/CDH3/CLEC7A/WNT5A/CCNB1/LTF | 20 |
| BP | GO:0070232 | regulation of T cell apoptotic process | 7/706 | 34/18862 | 0.000222197 | 0.006408162 | 0.005145361 | PIP/SLC46A2/IDO1/IL7R/PRKCQ/WNT5A/CD274 | 7 |
| BP | GO:0002830 | positive regulation of type 2 immune response | 5/706 | 16/18862 | 0.000224273 | 0.006408162 | 0.005145361 | GATA3/IL4R/IDO1/NOD2/RSAD2 | 5 |
| BP | GO:0010819 | regulation of T cell chemotaxis | 5/706 | 16/18862 | 0.000224273 | 0.006408162 | 0.005145361 | CCL27/WNT5A/CXCL13/CXCL10/S100A7 | 5 |
| BP | GO:0032757 | positive regulation of interleukin-8 production | 9/706 | 57/18862 | 0.00024647 | 0.006960985 | 0.005589243 | F3/ADIPOQ/IL17D/PYCARD/AFAP1L2/CD2/NOD2/CLEC7A/WNT5A | 9 |
| BP | GO:0048008 | platelet-derived growth factor receptor signaling pathway | 9/706 | 57/18862 | 0.00024647 | 0.006960985 | 0.005589243 | F3/APOD/MYOCD/ADIPOQ/PDGFRL/PDGFD/PDGFC/SMPD3/PLAT | 9 |
| BP | GO:0071459 | protein localization to chromosome, centromeric region | 6/706 | 25/18862 | 0.000259059 | 0.007198351 | 0.005779834 | AURKB/CENPA/BUB1B/CDK1/NDC80/TTK | 6 |
| BP | GO:0022404 | molting cycle process | 11/706 | 83/18862 | 0.000259623 | 0.007198351 | 0.005779834 | GAL/LGR5/BCL2/IGFBP5/TMEM79/FERMT1/ZDHHC21/CDH3/WNT5A/TGM3/HPSE | 11 |
| BP | GO:0022405 | hair cycle process | 11/706 | 83/18862 | 0.000259623 | 0.007198351 | 0.005779834 | GAL/LGR5/BCL2/IGFBP5/TMEM79/FERMT1/ZDHHC21/CDH3/WNT5A/TGM3/HPSE | 11 |
| BP | GO:0071675 | regulation of mononuclear cell migration | 13/706 | 111/18862 | 0.000260767 | 0.007198351 | 0.005779834 | CCL27/GREM1/APOD/PDGFD/CCL2/PYCARD/CCR7/WNT5A/CXCL13/CXCL10/CCL20/S100A7/C10orf99 | 13 |
| BP | GO:0009991 | response to extracellular stimulus | 34/706 | 477/18862 | 0.000265879 | 0.007257887 | 0.005827637 | FADS1/WNT2B/PDK4/POSTN/PTN/PPARGC1A/ADRB2/MYOCD/ADIPOQ/FOS/MAP1B/LPL/APOE/BCL2/ADRB1/CCND1/PSPH/SLC27A4/IFI16/PPARD/HMOX1/FOSL1/EIF4EBP1/SLC16A1/LDLR/LIPG/TYMS/NOD2/DSC2/ARG1/UPP1/CXCL10/ZC3H12A/KYNU | 34 |
| BP | GO:0032612 | interleukin-1 production | 14/706 | 126/18862 | 0.000267386 | 0.007257887 | 0.005827637 | LPL/TRIM16/IFI16/CCL19/CD36/FZD5/AIM2/PYCARD/NOD2/CCR7/SERPINB1/CLEC7A/WNT5A/ZC3H12A | 14 |
| BP | GO:0008608 | attachment of spindle microtubules to kinetochore | 7/706 | 35/18862 | 0.000268866 | 0.007257887 | 0.005827637 | NUF2/AURKB/ECT2/NEK2/KIF2C/NDC80/CCNB1 | 7 |
| BP | GO:0034367 | protein-containing complex remodeling | 7/706 | 35/18862 | 0.000268866 | 0.007257887 | 0.005827637 | AGTR1/APOC1/LPL/APOE/ABCG1/LIPG/PLA2G3 | 7 |
| BP | GO:0048146 | positive regulation of fibroblast proliferation | 8/706 | 46/18862 | 0.000273932 | 0.007354017 | 0.005904824 | BTC/ESR1/PDGFD/PDGFC/EREG/CCNA2/WNT5A/CCNB1 | 8 |
| BP | GO:0098773 | skin epidermis development | 11/706 | 84/18862 | 0.000288825 | 0.007711465 | 0.006191833 | GAL/LGR5/BCL2/IGFBP5/TMEM79/FERMT1/ZDHHC21/CDH3/WNT5A/TGM3/HPSE | 11 |
| BP | GO:0043627 | response to estrogen | 10/706 | 71/18862 | 0.000297098 | 0.007889235 | 0.006334571 | GAL/GATA3/AR/ESR1/KRT19/CCND1/SERPINB9/HMOX1/IL4R/CD24 | 10 |
| BP | GO:0051382 | kinetochore assembly | 5/706 | 17/18862 | 0.000307974 | 0.008090119 | 0.006495868 | CENPF/CENPW/CENPN/CENPA/DLGAP5 | 5 |
| BP | GO:0051797 | regulation of hair follicle development | 5/706 | 17/18862 | 0.000307974 | 0.008090119 | 0.006495868 | GAL/FERMT1/CDH3/WNT5A/HPSE | 5 |
| BP | GO:0042634 | regulation of hair cycle | 6/706 | 26/18862 | 0.000326199 | 0.008477697 | 0.00680707 | GAL/FA2H/FERMT1/CDH3/WNT5A/HPSE | 6 |
| BP | GO:1901623 | regulation of lymphocyte chemotaxis | 6/706 | 26/18862 | 0.000326199 | 0.008477697 | 0.00680707 | CCL27/CCL2/WNT5A/CXCL13/CXCL10/S100A7 | 6 |
| BP | GO:0031099 | regeneration | 18/706 | 192/18862 | 0.00033089 | 0.008554121 | 0.006868433 | POSTN/PTN/CHL1/CLDN1/APOD/MAP1B/TGFBR3/BCL2/CCND1/PPARD/EZH2/HMOX1/AURKA/KLK8/TYMS/CCNA2/CCNB1/KLK6 | 18 |
| BP | GO:0050891 | multicellular organismal water homeostasis | 10/706 | 72/18862 | 0.000333642 | 0.008579867 | 0.006889106 | FA2H/FLG2/CLDN1/ADCY2/TFAP2B/TMEM79/GRHL3/ABCA12/ALOX12B/KRT16 | 10 |
| BP | GO:0016101 | diterpenoid metabolic process | 13/706 | 114/18862 | 0.000339666 | 0.008650828 | 0.006946084 | RBP4/ADH1B/LPL/SDC2/APOE/PPARD/RDH12/CYP2C18/LRP8/DHRS9/ALDH1A3/CRABP2/AKR1B10 | 13 |
| BP | GO:0032680 | regulation of tumor necrosis factor production | 16/706 | 160/18862 | 0.000339942 | 0.008650828 | 0.006946084 | IL37/CD34/ADIPOQ/LPL/CCL19/CD36/RASGRP1/FZD5/PYCARD/CD2/IFIH1/NOD2/CLEC7A/WNT5A/ZC3H12A/LTF | 16 |
| BP | GO:1901654 | response to ketone | 18/706 | 193/18862 | 0.000352497 | 0.008923842 | 0.007165297 | HSD3B1/PTN/CLDN1/AR/FIBIN/FOS/ADCY2/PLN/TGFBR3/CCND1/FBXO32/PSPH/CCL19/FOSL1/TYMS/CCR7/ARG1/GJB2 | 18 |
| BP | GO:0006936 | muscle contraction | 27/706 | 352/18862 | 0.00036103 | 0.009092748 | 0.007300918 | DES/SORBS1/ACTG2/SNTB1/SCN7A/ADRB2/CACNA2D1/MYH11/MYOCD/LMOD1/TPM2/SSPN/MYL9/DMD/CRYAB/SYNM/CNN1/ATP1A2/TPM1/PLN/MYLK/LTB4R/COMP/DSC2/ZDHHC21/CXCR4/ZC3H12A | 27 |
| BP | GO:0032722 | positive regulation of chemokine production | 9/706 | 60/18862 | 0.000366552 | 0.009137615 | 0.007336943 | POSTN/ADIPOQ/LPL/HMOX1/IL4R/PYCARD/NOD2/CLEC7A/WNT5A | 9 |
| BP | GO:0061098 | positive regulation of protein tyrosine kinase activity | 9/706 | 60/18862 | 0.000366552 | 0.009137615 | 0.007336943 | BTC/GREM1/TGFA/LRP8/HBEGF/AREG/AFAP1L2/EREG/CD24 | 9 |
| BP | GO:0003018 | vascular process in circulatory system | 21/706 | 245/18862 | 0.000375571 | 0.009314917 | 0.007479306 | LEPR/AGTR1/DDAH1/ADRB2/ATP1A2/SLC29A1/SLC2A13/APOE/KCNMB4/ADRB1/SLC27A4/CD36/SLC7A1/GCH1/SLC16A1/COMP/FABP5/SLC7A5/HRH2/ZDHHC21/SLC5A1 | 21 |
| BP | GO:2000403 | positive regulation of lymphocyte migration | 7/706 | 37/18862 | 0.000385761 | 0.009519182 | 0.007643318 | CCL27/PYCARD/WNT5A/CXCL13/CXCL10/CCL20/S100A7 | 7 |
| BP | GO:0097006 | regulation of plasma lipoprotein particle levels | 12/706 | 101/18862 | 0.000389602 | 0.009519182 | 0.007643318 | AGTR1/APOC1/ADIPOQ/LPL/APOE/ABCG1/CD36/HMOX1/LDLR/LIPG/PLA2G3/IL19 | 12 |
| BP | GO:0032640 | tumor necrosis factor production | 16/706 | 162/18862 | 0.000390922 | 0.009519182 | 0.007643318 | IL37/CD34/ADIPOQ/LPL/CCL19/CD36/RASGRP1/FZD5/PYCARD/CD2/IFIH1/NOD2/CLEC7A/WNT5A/ZC3H12A/LTF | 16 |
| BP | GO:0034599 | cellular response to oxidative stress | 24/706 | 299/18862 | 0.000392785 | 0.009519182 | 0.007643318 | PPARGC1A/MGST1/FOS/RHOB/PDGFD/PLA2R1/TPM1/BCL2/CD36/EZH2/HMOX1/ECT2/SMPD3/GCH1/CCNA2/PPIF/GPX2/MMP9/VNN1/MELK/ARG1/SLC7A11/GJB2/ZC3H12A | 24 |
| BP | GO:0032760 | positive regulation of tumor necrosis factor production | 11/706 | 87/18862 | 0.000393548 | 0.009519182 | 0.007643318 | LPL/CCL19/CD36/RASGRP1/FZD5/PYCARD/CD2/IFIH1/NOD2/CLEC7A/WNT5A | 11 |
| BP | GO:0061041 | regulation of wound healing | 14/706 | 131/18862 | 0.000400193 | 0.009632243 | 0.007734099 | TSPAN8/F3/CD34/CLDN1/APOE/MYLK/CD36/FERMT1/HBEGF/F12/CLEC7A/CXCR4/PLAT/HPSE | 14 |
| BP | GO:0010818 | T cell chemotaxis | 6/706 | 27/18862 | 0.000406235 | 0.009729723 | 0.00781237 | CCL27/CXCL11/WNT5A/CXCL13/CXCL10/S100A7 | 6 |
| BP | GO:0034375 | high-density lipoprotein particle remodeling | 5/706 | 18/18862 | 0.000413359 | 0.009756862 | 0.007834161 | APOC1/APOE/ABCG1/LIPG/PLA2G3 | 5 |
| BP | GO:0051546 | keratinocyte migration | 5/706 | 18/18862 | 0.000413359 | 0.009756862 | 0.007834161 | PPARD/FERMT1/HBEGF/MMP9/KRT16 | 5 |
| BP | GO:0060065 | uterus development | 5/706 | 18/18862 | 0.000413359 | 0.009756862 | 0.007834161 | RBP4/GATA3/MYOCD/ESR1/WNT5A | 5 |
| BP | GO:0070542 | response to fatty acid | 9/706 | 61/18862 | 0.000415942 | 0.009770645 | 0.007845227 | PDK4/GLDC/PPARGC1A/CLDN1/ADIPOQ/LPL/CD36/CCNB1/ZC3H12A | 9 |
| BP | GO:0044242 | cellular lipid catabolic process | 20/706 | 230/18862 | 0.000426441 | 0.009969343 | 0.00800477 | FABP7/CRAT/ABHD12B/HAO2/APOC1/ADIPOQ/ACADL/ACOX2/PNPLA3/LPL/SLC27A4/PPARD/SMPD3/LDLR/LIPG/FABP5/GM2A/PLBD1/GDPD3/AKR1B10 | 20 |
| BP | GO:0051235 | maintenance of location | 25/706 | 319/18862 | 0.000428768 | 0.009976008 | 0.008010122 | MCOLN3/DMD/ATP1A2/SLC18A2/PLN/LPL/APOE/DBN1/SQLE/PPARD/CCL19/CXCL11/ABCG1/CD36/LCK/HK2/GM2A/CCR7/CXCL9/CXCL10/ZC3H12A/S100A8/LCN2/S100A7/S100A9 | 25 |
| BP | GO:1903034 | regulation of response to wounding | 16/706 | 164/18862 | 0.000448351 | 0.01030185 | 0.008271753 | TSPAN8/F3/PTN/CD34/CLDN1/APOE/MYLK/CD36/FERMT1/HBEGF/KLK8/F12/CLEC7A/CXCR4/PLAT/HPSE | 16 |
| BP | GO:0001655 | urogenital system development | 25/706 | 320/18862 | 0.000449098 | 0.01030185 | 0.008271753 | EMX2/ZBTB16/WNT2B/GREM1/AGTR1/LGR5/OSR2/RBP4/GATA3/CD34/AR/MYOCD/ADIPOQ/PSAPL1/CRIP1/ESR1/PDGFD/BCL2/TFAP2B/CENPF/CYP7B1/CD24/STAT1/MMP9/WNT5A | 25 |
| BP | GO:0034612 | response to tumor necrosis factor | 25/706 | 320/18862 | 0.000449098 | 0.01030185 | 0.008271753 | POSTN/AFF3/PPARGC1A/GATA3/CLDN1/RORA/ADIPOQ/CCL19/LTB/CCL22/SMPD3/AIM2/PSME2/TNFRSF21/GCH1/CCL2/PYCARD/TXNDC17/SELE/STAT1/GBP1/CXCL8/CCL18/ZC3H12A/CCL20 | 25 |
| BP | GO:0010742 | macrophage derived foam cell differentiation | 7/706 | 38/18862 | 0.000457775 | 0.010403205 | 0.008353135 | AGTR1/ADIPOQ/LPL/ABCG1/CD36/PLA2G3/STAT1 | 7 |
| BP | GO:0090077 | foam cell differentiation | 7/706 | 38/18862 | 0.000457775 | 0.010403205 | 0.008353135 | AGTR1/ADIPOQ/LPL/ABCG1/CD36/PLA2G3/STAT1 | 7 |
| BP | GO:1901293 | nucleoside phosphate biosynthetic process | 22/706 | 267/18862 | 0.000474413 | 0.0107314 | 0.008616655 | ELOVL3/ACSBG1/SCD5/PDK4/PPARGC1A/GUCY1A2/ADCY2/FASN/CTPS1/ELOVL7/UCK2/AMPD3/IDO1/TYMS/TK1/NAMPT/PARP9/PNP/CMPK2/UPP1/RRM2/KYNU | 22 |
| BP | GO:0007584 | response to nutrient | 16/706 | 165/18862 | 0.000479686 | 0.010752108 | 0.008633282 | POSTN/PTN/ADIPOQ/MAP1B/LPL/CCND1/SLC27A4/PPARD/HMOX1/SLC16A1/LIPG/TYMS/NOD2/ARG1/CXCL10/KYNU | 16 |
| BP | GO:0032642 | regulation of chemokine production | 11/706 | 89/18862 | 0.00047973 | 0.010752108 | 0.008633282 | POSTN/APOD/ADIPOQ/LPL/HMOX1/IL4R/PYCARD/EPHA2/NOD2/CLEC7A/WNT5A | 11 |
| BP | GO:0016042 | lipid catabolic process | 26/706 | 340/18862 | 0.000482395 | 0.010762482 | 0.008641612 | FABP7/HSD11B1/CRAT/ABHD12B/HAO2/PLCB4/APOC1/CYP39A1/ADIPOQ/ACADL/PLIN1/ACOX2/PNPLA3/LPL/APOE/SLC27A4/PPARD/PLA2G2F/SMPD3/LDLR/LIPG/FABP5/GM2A/PLBD1/GDPD3/AKR1B10 | 26 |
| BP | GO:0042110 | T cell activation | 33/706 | 474/18862 | 0.000493679 | 0.010924993 | 0.008772098 | ZBTB16/LEPR/SLC46A2/GATA3/RORA/BCL2/CCL19/PLA2G2F/CTPS1/RASGRP1/FZD5/IL4R/SLC7A1/TNFRSF21/IDO1/CCL2/PYCARD/PRDM1/IL7R/CD2/LCK/CD24/PRKCQ/NFKBIZ/NOD2/CCR7/VNN1/ARG1/CLEC7A/PNP/RSAD2/CD274/ZC3H12A | 33 |
| BP | GO:0002931 | response to ischemia | 8/706 | 50/18862 | 0.000494151 | 0.010924993 | 0.008772098 | PPARGC1A/EEF2K/BCL2/EIF4EBP1/HK2/PPIF/TIGAR/GJB2 | 8 |
| BP | GO:1901988 | negative regulation of cell cycle phase transition | 22/706 | 268/18862 | 0.000499125 | 0.010985247 | 0.008820478 | SUSD2/BCL2/CCND1/FHL1/EZH2/AURKB/CENPF/AURKA/BUB1/E2F8/PSME2/CCL2/NABP1/ZWINT/BUB1B/CDK1/MAD2L1/NDC80/TTK/CDC20/CCNB1/C10orf99 | 22 |
| BP | GO:0045834 | positive regulation of lipid metabolic process | 15/706 | 150/18862 | 0.000515381 | 0.011248842 | 0.009032129 | AGTR1/SORBS1/PPARGC1A/APOC1/ADIPOQ/APOE/PPARD/CCL19/ABCG1/RAB38/SMPD3/LDLR/PLA2G3/NOD2/CCR7 | 15 |
| BP | GO:0032652 | regulation of interleukin-1 production | 13/706 | 119/18862 | 0.000516241 | 0.011248842 | 0.009032129 | LPL/TRIM16/IFI16/CCL19/FZD5/AIM2/PYCARD/NOD2/CCR7/SERPINB1/CLEC7A/WNT5A/ZC3H12A | 13 |
| BP | GO:0008655 | pyrimidine-containing compound salvage | 4/706 | 11/18862 | 0.000520311 | 0.011248842 | 0.009032129 | UCK2/TK1/UPP1/TYMP | 4 |
| BP | GO:0043097 | pyrimidine nucleoside salvage | 4/706 | 11/18862 | 0.000520311 | 0.011248842 | 0.009032129 | UCK2/TK1/UPP1/TYMP | 4 |
| BP | GO:1903557 | positive regulation of tumor necrosis factor superfamily cytokine production | 11/706 | 90/18862 | 0.000528415 | 0.01137372 | 0.009132399 | LPL/CCL19/CD36/RASGRP1/FZD5/PYCARD/CD2/IFIH1/NOD2/CLEC7A/WNT5A | 11 |
| BP | GO:0071677 | positive regulation of mononuclear cell migration | 9/706 | 63/18862 | 0.000531198 | 0.011383479 | 0.009140235 | CCL27/PDGFD/PYCARD/CCR7/WNT5A/CXCL13/CXCL10/CCL20/S100A7 | 9 |
| BP | GO:0031349 | positive regulation of defense response | 27/706 | 361/18862 | 0.000534302 | 0.011400006 | 0.009153504 | MUC7/AGTR1/LPL/IFI16/CTSC/RAET1E/RASGRP1/PLSCR1/AIM2/NMI/PSME2/IDO1/PYCARD/EREG/PLA2G3/NFKBIZ/NOD2/POLR3G/CCR7/PARP9/ARG1/IRF7/WNT5A/MMP12/S100A8/S100A12/S100A9 | 27 |
| BP | GO:0002544 | chronic inflammatory response | 5/706 | 19/18862 | 0.000543813 | 0.01155247 | 0.009275924 | IDO1/VNN1/CXCL13/S100A8/S100A9 | 5 |
| BP | GO:0006636 | unsaturated fatty acid biosynthetic process | 8/706 | 51/18862 | 0.000567149 | 0.011996059 | 0.009632099 | ELOVL3/FADS1/FADS2/SCD5/HPGDS/ELOVL7/PLA2G3/FABP5 | 8 |
| BP | GO:0042742 | defense response to bacterium | 26/706 | 344/18862 | 0.000575003 | 0.012079168 | 0.00969883 | ANG/PGLYRP4/CD36/IRF8/GSDMC/SLPI/PYCARD/IL7R/EPHA2/PLAC8/NOD2/RNASE7/GBP6/WFDC12/KRT6A/ISG15/CXCL13/CCL20/S100A8/LCN2/LTF/S100A7/C10orf99/S100A12/S100A9/PI3 | 26 |
| BP | GO:0002088 | lens development in camera-type eye | 10/706 | 77/18862 | 0.000577339 | 0.012079168 | 0.00969883 | WNT2B/SLITRK6/GATA3/CRYAB/SPRY2/CDON/BCAR3/EPHA2/SLC7A11/WNT5A | 10 |
| BP | GO:0002831 | regulation of response to biotic stimulus | 30/706 | 420/18862 | 0.000578495 | 0.012079168 | 0.00969883 | MUC7/APOE/IFI16/RAET1E/SERPINB9/CD36/RASGRP1/PLSCR1/AIM2/LRP8/NMI/PSME2/DDX60/PYCARD/EREG/STAT1/IFIT1/NOD2/SOCS3/POLR3G/PARP9/TIGAR/ARG1/IRF7/WNT5A/CD274/ZC3H12A/MMP12/LTF/SERPINB4 | 30 |
| BP | GO:0006638 | neutral lipid metabolic process | 14/706 | 136/18862 | 0.000585649 | 0.012124926 | 0.009735571 | THRSP/FABP7/SERPINA12/ABHD12B/APOC1/ANG/PNPLA3/LPL/APOE/GK/LDLR/CYP2E1/LIPG/FABP5 | 14 |
| BP | GO:0006639 | acylglycerol metabolic process | 14/706 | 136/18862 | 0.000585649 | 0.012124926 | 0.009735571 | THRSP/FABP7/SERPINA12/ABHD12B/APOC1/ANG/PNPLA3/LPL/APOE/GK/LDLR/CYP2E1/LIPG/FABP5 | 14 |
| BP | GO:0050863 | regulation of T cell activation | 25/706 | 327/18862 | 0.000616651 | 0.012712908 | 0.010207684 | ZBTB16/SLC46A2/GATA3/CCL19/PLA2G2F/IL4R/SLC7A1/TNFRSF21/IDO1/CCL2/PYCARD/PRDM1/IL7R/CD2/LCK/CD24/PRKCQ/NFKBIZ/NOD2/CCR7/VNN1/ARG1/PNP/CD274/ZC3H12A | 25 |
| BP | GO:0072528 | pyrimidine-containing compound biosynthetic process | 7/706 | 40/18862 | 0.000633815 | 0.013011842 | 0.01044771 | CTPS1/UCK2/TYMS/TK1/CMPK2/UPP1/TYMP | 7 |
| BP | GO:0043491 | protein kinase B signaling | 22/706 | 273/18862 | 0.000640256 | 0.01308909 | 0.010509736 | BTC/F3/GATA3/ESR1/TSPYL5/SPRY2/MYOC/IGFBP5/CCL19/MFHAS1/TGFA/SMPD3/HBEGF/AREG/CCL2/TPBG/EPHA2/EREG/LCK/CCR7/FAM110C/HPSE | 22 |
| BP | GO:0007159 | leukocyte cell-cell adhesion | 27/706 | 366/18862 | 0.000659151 | 0.013419214 | 0.010774805 | ZBTB16/GATA3/CCL19/PLA2G2F/IL4R/SLC7A1/TNFRSF21/IDO1/CCL2/PYCARD/IL7R/LCK/CD24/PRKCQ/SELL/SELE/NFKBIZ/NOD2/CCR7/VNN1/ZDHHC21/ARG1/PNP/CD274/ZC3H12A/S100A8/S100A9 | 27 |
| BP | GO:0002819 | regulation of adaptive immune response | 16/706 | 170/18862 | 0.00066612 | 0.013504818 | 0.010843539 | GATA3/CD48/CCL19/FZD5/IL4R/PYCARD/IL7R/PRKCQ/NFKBIZ/NOD2/SAMSN1/ARG1/IRF7/RSAD2/CD274/ZC3H12A | 16 |
| BP | GO:0009636 | response to toxic substance | 20/706 | 239/18862 | 0.000694603 | 0.014024099 | 0.01126049 | CLDN1/MGST1/FOS/MAP1B/PDZK1/SLC18A2/APOE/BCL2/CD36/RDH12/GCH1/TXNDC17/TYMS/GPX2/ARG1/SLC7A11/CCNB1/SLC6A14/AKR1B10/S100A9 | 20 |
| BP | GO:0070233 | negative regulation of T cell apoptotic process | 5/706 | 20/18862 | 0.000702907 | 0.014133343 | 0.011348207 | PIP/SLC46A2/IDO1/IL7R/PRKCQ | 5 |
| BP | GO:0035821 | modulation of process of other organism | 13/706 | 123/18862 | 0.000708468 | 0.014186789 | 0.01139112 | APOE/SERPINB9/SLPI/KPNA2/TYMS/RNASE7/ARG1/CLEC7A/KRT6A/ZC3H12A/IFI27/LTF/S100A9 | 13 |
| BP | GO:0006641 | triglyceride metabolic process | 12/706 | 108/18862 | 0.000718934 | 0.014337592 | 0.011512206 | THRSP/FABP7/SERPINA12/APOC1/PNPLA3/LPL/APOE/GK/LDLR/CYP2E1/LIPG/FABP5 | 12 |
| BP | GO:0002720 | positive regulation of cytokine production involved in immune response | 8/706 | 53/18862 | 0.000739271 | 0.01465086 | 0.011763741 | GATA3/CD36/FZD5/NOD2/SLC7A5/CLEC7A/WNT5A/RSAD2 | 8 |
| BP | GO:0002828 | regulation of type 2 immune response | 6/706 | 30/18862 | 0.000740639 | 0.01465086 | 0.011763741 | GATA3/IL4R/IDO1/NOD2/ARG1/RSAD2 | 6 |
| BP | GO:0048820 | hair follicle maturation | 4/706 | 12/18862 | 0.000757429 | 0.014922566 | 0.011981904 | GAL/FERMT1/CDH3/WNT5A | 4 |
| BP | GO:0070228 | regulation of lymphocyte apoptotic process | 8/706 | 54/18862 | 0.000839841 | 0.016360281 | 0.013136301 | PIP/SLC46A2/AURKB/IDO1/IL7R/PRKCQ/WNT5A/CD274 | 8 |
| BP | GO:0031640 | killing of cells of other organism | 9/706 | 67/18862 | 0.000840448 | 0.016360281 | 0.013136301 | MUC7/PGLYRP4/SERPINB9/APOL1/ARG1/CLEC7A/KRT6A/LTF/S100A12 | 9 |
| BP | GO:1900046 | regulation of hemostasis | 9/706 | 67/18862 | 0.000840448 | 0.016360281 | 0.013136301 | TSPAN8/F3/CD34/APOE/CD36/COMP/F12/PLAT/HPSE | 9 |
| BP | GO:0044282 | small molecule catabolic process | 30/706 | 431/18862 | 0.000877497 | 0.016797524 | 0.01348738 | CRAT/HAO2/GLDC/DDAH1/CYP39A1/ADIPOQ/ACADL/ACOX2/APOE/SLC27A4/PPARD/IL4I1/BDH1/GK/IDO1/AASS/NT5C3A/GPT2/PGM2/HK2/APOBEC3B/FUT2/TIGAR/ARG1/PNP/HAL/UPP1/TYMP/KYNU/AKR1B10 | 30 |
| BP | GO:0044843 | cell cycle G1/S phase transition | 23/706 | 298/18862 | 0.000881422 | 0.016797524 | 0.01348738 | SUSD2/BCL2/CCND1/FHL1/GSPT1/EZH2/POLE2/AURKA/MCM10/E2F8/EIF4EBP1/PSME2/CCNE2/CCL2/CCNE1/TYMS/CCNA2/FAM83D/CDK1/CDKN3/RRM2/CCNB1/C10orf99 | 23 |
| BP | GO:1902652 | secondary alcohol metabolic process | 15/706 | 158/18862 | 0.000886934 | 0.016797524 | 0.01348738 | SERPINA12/LEPR/CYP39A1/HMGCS2/ACADL/FASN/APOE/SQLE/PPARD/ABCG1/CYP7B1/CH25H/LDLR/APOL1/IDH3A | 15 |
| BP | GO:0007272 | ensheathment of neurons | 13/706 | 126/18862 | 0.000889387 | 0.016797524 | 0.01348738 | ACSBG1/TPPP/FA2H/PTN/PLLP/MYOC/CTSC/PPARD/TNFRSF21/KLK8/CXCR4/TYMP/KLK6 | 13 |
| BP | GO:0008366 | axon ensheathment | 13/706 | 126/18862 | 0.000889387 | 0.016797524 | 0.01348738 | ACSBG1/TPPP/FA2H/PTN/PLLP/MYOC/CTSC/PPARD/TNFRSF21/KLK8/CXCR4/TYMP/KLK6 | 13 |
| BP | GO:0070229 | negative regulation of lymphocyte apoptotic process | 6/706 | 31/18862 | 0.000889653 | 0.016797524 | 0.01348738 | PIP/SLC46A2/AURKB/IDO1/IL7R/PRKCQ | 6 |
| BP | GO:0097421 | liver regeneration | 6/706 | 31/18862 | 0.000889653 | 0.016797524 | 0.01348738 | CLDN1/CCND1/EZH2/HMOX1/AURKA/TYMS | 6 |
| BP | GO:0031667 | response to nutrient levels | 31/706 | 451/18862 | 0.000890413 | 0.016797524 | 0.01348738 | FADS1/WNT2B/PDK4/POSTN/PTN/PPARGC1A/ADRB2/ADIPOQ/MAP1B/LPL/APOE/BCL2/ADRB1/CCND1/PSPH/SLC27A4/IFI16/PPARD/HMOX1/EIF4EBP1/SLC16A1/LDLR/LIPG/TYMS/NOD2/DSC2/ARG1/UPP1/CXCL10/ZC3H12A/KYNU | 31 |
| BP | GO:0002548 | monocyte chemotaxis | 9/706 | 68/18862 | 0.000937093 | 0.017542669 | 0.014085686 | GREM1/CCL19/CCL22/CCL2/CCL18/CXCL10/CCL20/S100A7/S100A12 | 9 |
| BP | GO:0006026 | aminoglycan catabolic process | 9/706 | 68/18862 | 0.000937093 | 0.017542669 | 0.014085686 | OGN/LYVE1/OMD/SDC2/PGLYRP4/HMMR/HYAL4/CHI3L2/HPSE | 9 |
| BP | GO:0007093 | mitotic cell cycle checkpoint | 15/706 | 159/18862 | 0.000946335 | 0.017580964 | 0.014116434 | CCND1/AURKB/CENPF/AURKA/BUB1/E2F8/NABP1/ZWINT/BUB1B/CDK1/MAD2L1/NDC80/TTK/CDC20/CCNB1 | 15 |
| BP | GO:0051092 | positive regulation of NF-kappaB transcription factor activity | 15/706 | 159/18862 | 0.000946335 | 0.017580964 | 0.014116434 | GREM1/AR/CD36/AIM2/TRIM22/PYCARD/PRKCQ/IRAK2/TRIM14/NOD2/WNT5A/S100A8/LTF/S100A12/S100A9 | 15 |
| BP | GO:0006006 | glucose metabolic process | 18/706 | 210/18862 | 0.000957669 | 0.017657247 | 0.014177684 | SERPINA12/LEPR/SORBS1/PDK4/RBP4/PPARGC1A/PGM5/APOD/RORA/ADIPOQ/TFAP2B/PPARD/PHLDA2/PGM2/HK2/FABP5/TIGAR/OAS1 | 18 |
| BP | GO:0070374 | positive regulation of ERK1 and ERK2 cascade | 18/706 | 210/18862 | 0.000957669 | 0.017657247 | 0.014177684 | MTURN/PDGFD/PDGFC/SPRY2/APOE/CCL19/MFHAS1/CD36/RASGRP1/CCL22/CCL2/PYCARD/TPBG/NOD2/CCR7/CCL18/CCL20/S100A7 | 18 |
| BP | GO:0009165 | nucleotide biosynthetic process | 21/706 | 264/18862 | 0.000993191 | 0.018064948 | 0.014505044 | ELOVL3/ACSBG1/SCD5/PDK4/PPARGC1A/GUCY1A2/ADCY2/FASN/CTPS1/ELOVL7/UCK2/AMPD3/IDO1/TYMS/NAMPT/PARP9/PNP/CMPK2/UPP1/RRM2/KYNU | 21 |
| BP | GO:0045840 | positive regulation of mitotic nuclear division | 7/706 | 43/18862 | 0.000994154 | 0.018064948 | 0.014505044 | BTC/AURKA/TGFA/SMPD3/EREG/NUSAP1/DLGAP5 | 7 |
| BP | GO:0046189 | phenol-containing compound biosynthetic process | 7/706 | 43/18862 | 0.000994154 | 0.018064948 | 0.014505044 | GATA3/PMEL/GCH1/MOXD1/CDH3/SLC7A11/WNT5A | 7 |
| BP | GO:0030278 | regulation of ossification | 12/706 | 112/18862 | 0.00099457 | 0.018064948 | 0.014505044 | ZBTB16/GREM1/OSR2/PTN/ADRB2/OMD/BCL2/SOST/COMP/WNT5A/ISG15/LTF | 12 |
| BP | GO:0051896 | regulation of protein kinase B signaling | 20/706 | 247/18862 | 0.001044554 | 0.018865498 | 0.015147836 | BTC/F3/GATA3/ESR1/TSPYL5/SPRY2/MYOC/IGFBP5/CCL19/MFHAS1/TGFA/HBEGF/AREG/TPBG/EPHA2/EREG/LCK/CCR7/FAM110C/HPSE | 20 |
| BP | GO:0031145 | anaphase-promoting complex-dependent catabolic process | 10/706 | 83/18862 | 0.001047531 | 0.018865498 | 0.015147836 | AURKB/AURKA/PSME2/BUB1B/UBE2C/CDK1/MAD2L1/PTTG1/CDC20/CCNB1 | 10 |
| BP | GO:0010743 | regulation of macrophage derived foam cell differentiation | 6/706 | 32/18862 | 0.001060719 | 0.018865498 | 0.015147836 | AGTR1/ADIPOQ/LPL/ABCG1/CD36/PLA2G3 | 6 |
| BP | GO:0007171 | activation of transmembrane receptor protein tyrosine kinase activity | 4/706 | 13/18862 | 0.001061812 | 0.018865498 | 0.015147836 | GREM1/ADRB2/PRLR/PDGFC | 4 |
| BP | GO:0033700 | phospholipid efflux | 4/706 | 13/18862 | 0.001061812 | 0.018865498 | 0.015147836 | APOC1/APOE/ABCG1/ABCA12 | 4 |
| BP | GO:0051988 | regulation of attachment of spindle microtubules to kinetochore | 4/706 | 13/18862 | 0.001061812 | 0.018865498 | 0.015147836 | AURKB/ECT2/NEK2/CCNB1 | 4 |
| BP | GO:0022407 | regulation of cell-cell adhesion | 30/706 | 437/18862 | 0.001091756 | 0.01932725 | 0.015518595 | ZBTB16/GATA3/ADIPOQ/EPCAM/MYADM/CCL19/PLA2G2F/IL4R/SLC7A1/TNFRSF21/IDO1/CCL2/PYCARD/IL7R/FUT3/LCK/CD24/PRKCQ/SELE/NFKBIZ/NOD2/CCR7/VNN1/ZDHHC21/ARG1/PNP/WNT5A/CD274/CXCL13/ZC3H12A | 30 |
| BP | GO:0006582 | melanin metabolic process | 5/706 | 22/18862 | 0.001122096 | 0.019792648 | 0.01589228 | BCL2/PMEL/CDH3/SLC7A11/WNT5A | 5 |
| BP | GO:0002718 | regulation of cytokine production involved in immune response | 10/706 | 84/18862 | 0.001150059 | 0.020212901 | 0.016229718 | GATA3/CD36/HMOX1/FZD5/NOD2/SLC7A5/ARG1/CLEC7A/WNT5A/RSAD2 | 10 |
| BP | GO:0051785 | positive regulation of nuclear division | 8/706 | 57/18862 | 0.001208939 | 0.021116683 | 0.0169554 | BTC/AURKA/TGFA/SMPD3/EREG/NUSAP1/DLGAP5/WNT5A | 8 |
| BP | GO:1901991 | negative regulation of mitotic cell cycle phase transition | 20/706 | 250/18862 | 0.001210125 | 0.021116683 | 0.0169554 | BCL2/CCND1/FHL1/EZH2/AURKB/CENPF/AURKA/BUB1/E2F8/PSME2/CCL2/NABP1/ZWINT/BUB1B/CDK1/MAD2L1/NDC80/TTK/CDC20/CCNB1 | 20 |
| BP | GO:0030324 | lung development | 15/706 | 163/18862 | 0.001218715 | 0.021190898 | 0.017014989 | HSD11B1/WNT2B/RBP4/PTN/MYOCD/SPRY2/IGFBP5/ESRP2/SMPD3/EIF4EBP1/ABCA12/ARG1/SLC7A11/WNT5A/MMP12 | 15 |
| BP | GO:0033559 | unsaturated fatty acid metabolic process | 12/706 | 115/18862 | 0.001254637 | 0.021688225 | 0.017414313 | ELOVL3/FADS1/FADS2/SCD5/HPGDS/PLA2G2F/ELOVL7/CYP2C18/CYP2E1/PLA2G3/FABP5/ALOX12B | 12 |
| BP | GO:0051146 | striated muscle cell differentiation | 21/706 | 269/18862 | 0.001257351 | 0.021688225 | 0.017414313 | GREM1/BHLHE41/SOX6/PGM5/MYH11/MYOCD/LMOD1/DMD/EFNB2/TPM1/CDON/BCL2/KRT19/CFL2/IGFBP5/EZH2/IL4R/COMP/CXCL9/CCNB1/CXCL10 | 21 |
| BP | GO:0072527 | pyrimidine-containing compound metabolic process | 10/706 | 85/18862 | 0.001260634 | 0.021688225 | 0.017414313 | CTPS1/UCK2/NT5C3A/TYMS/TK1/APOBEC3B/ACP3/CMPK2/UPP1/TYMP | 10 |
| BP | GO:0097305 | response to alcohol | 19/706 | 233/18862 | 0.001269591 | 0.021765694 | 0.017476515 | HSD3B1/RBP4/GATA3/CLDN1/ADIPOQ/FOS/ADCY2/TGFBR3/CCND1/CCL19/SLC23A2/FOSL1/LRP8/EIF4EBP1/CYP2E1/TYMS/CCR7/GGH/S100A8 | 19 |
| BP | GO:0070227 | lymphocyte apoptotic process | 9/706 | 71/18862 | 0.001282326 | 0.021907143 | 0.01759009 | PIP/SLC46A2/AURKB/TNFRSF21/IDO1/IL7R/PRKCQ/WNT5A/CD274 | 9 |
| BP | GO:0030879 | mammary gland development | 13/706 | 132/18862 | 0.001368633 | 0.023300142 | 0.018708583 | GATA3/AR/PRLR/ESR1/SLC29A1/FASN/CCND1/IGFBP5/EPHA2/HK2/ARG1/WNT5A/OAS2 | 13 |
| BP | GO:0016125 | sterol metabolic process | 15/706 | 165/18862 | 0.001377927 | 0.023376912 | 0.018770225 | SERPINA12/LEPR/CYP39A1/HMGCS2/ACADL/FASN/APOE/SQLE/CYB5R2/PPARD/ABCG1/CYP7B1/CH25H/LDLR/APOL1 | 15 |
| BP | GO:0033032 | regulation of myeloid cell apoptotic process | 5/706 | 23/18862 | 0.001390066 | 0.02350125 | 0.01887006 | ADIPOQ/BCL2/NOD2/SLC7A11/IRF7 | 5 |
| BP | GO:0062197 | cellular response to chemical stress | 25/706 | 347/18862 | 0.00142893 | 0.024058355 | 0.019317382 | PPARGC1A/MGST1/FOS/RHOB/PDGFD/PLA2R1/TPM1/BCL2/MYLK/CD36/EZH2/HMOX1/ECT2/SMPD3/GCH1/CCNA2/PPIF/GPX2/MMP9/VNN1/MELK/ARG1/SLC7A11/GJB2/ZC3H12A | 25 |
| BP | GO:0008203 | cholesterol metabolic process | 14/706 | 149/18862 | 0.001435671 | 0.024058355 | 0.019317382 | SERPINA12/LEPR/CYP39A1/HMGCS2/ACADL/FASN/APOE/SQLE/PPARD/ABCG1/CYP7B1/CH25H/LDLR/APOL1 | 14 |
| BP | GO:0045785 | positive regulation of cell adhesion | 29/706 | 425/18862 | 0.00143779 | 0.024058355 | 0.019317382 | ZBTB16/PTN/GATA3/EDIL3/TPM1/MYADM/MYOC/CCL19/CD36/IL4R/SLC7A1/FERMT1/PRSS2/CCL2/PYCARD/IL7R/FUT3/LCK/CD24/PRKCQ/SELE/NFKBIZ/NOD2/CCR7/VNN1/PNP/WNT5A/CD274/CXCL13 | 29 |
| BP | GO:0010872 | regulation of cholesterol esterification | 4/706 | 14/18862 | 0.001442773 | 0.024059341 | 0.019318173 | AGTR1/APOC1/APOE/ABCG1 | 4 |
| BP | GO:0002697 | regulation of immune effector process | 31/706 | 465/18862 | 0.001450609 | 0.02410774 | 0.019357034 | RBP4/GATA3/CCL19/RAET1E/SERPINB9/CD36/C1QB/HMOX1/RASGRP1/FZD5/IL4R/AIM2/DDX60/PYCARD/IL7R/PLA2G3/DNASE1L3/STAT1/NFKBIZ/IFIT1/NOD2/SLC7A5/PARP9/ARG1/CLEC7A/CFB/WNT5A/RSAD2/ZC3H12A/MMP12/SERPINB4 | 31 |
| BP | GO:0001818 | negative regulation of cytokine production | 26/706 | 367/18862 | 0.001471643 | 0.024374396 | 0.019571143 | IL37/GATA3/CD34/APOD/ADIPOQ/RNF128/HMOX1/NMI/UBE2L6/TNFRSF21/IDO1/PYCARD/EPHA2/CD24/IFIH1/NOD2/GBP1/CCR7/SERPINB1/CDH3/ARG1/ISG15/CD274/IL36RN/ZC3H12A/LTF | 26 |
| BP | GO:0043094 | cellular metabolic compound salvage | 6/706 | 34/18862 | 0.001477441 | 0.024387763 | 0.019581876 | UCK2/AMPD3/TK1/PNP/UPP1/TYMP | 6 |
| BP | GO:0007204 | positive regulation of cytosolic calcium ion concentration | 23/706 | 310/18862 | 0.001488938 | 0.024494781 | 0.019667804 | AGTR1/CACNA2D1/MCOLN3/DMD/ESR1/ACKR4/ATP1A2/PLN/BCL2/CCL19/CXCL11/CD36/FPR1/GNA15/LCK/CD24/CCR7/CXCR4/CHRNA9/CXCR2/CXCL9/CXCL13/CXCL10 | 23 |
| BP | GO:0002699 | positive regulation of immune effector process | 18/706 | 219/18862 | 0.0015419 | 0.025280947 | 0.020299047 | RBP4/GATA3/CCL19/RAET1E/CD36/HMOX1/RASGRP1/FZD5/IL4R/DDX60/PLA2G3/NFKBIZ/NOD2/SLC7A5/ARG1/CLEC7A/WNT5A/RSAD2 | 18 |
| BP | GO:0030323 | respiratory tube development | 15/706 | 167/18862 | 0.001554236 | 0.02532355 | 0.020333255 | HSD11B1/WNT2B/RBP4/PTN/MYOCD/SPRY2/IGFBP5/ESRP2/SMPD3/EIF4EBP1/ABCA12/ARG1/SLC7A11/WNT5A/MMP12 | 15 |
| BP | GO:1903037 | regulation of leukocyte cell-cell adhesion | 24/706 | 330/18862 | 0.001554864 | 0.02532355 | 0.020333255 | ZBTB16/GATA3/CCL19/PLA2G2F/IL4R/SLC7A1/TNFRSF21/IDO1/CCL2/PYCARD/IL7R/LCK/CD24/PRKCQ/SELE/NFKBIZ/NOD2/CCR7/VNN1/ZDHHC21/ARG1/PNP/CD274/ZC3H12A | 24 |
| BP | GO:0014909 | smooth muscle cell migration | 10/706 | 88/18862 | 0.00164535 | 0.026708233 | 0.02144507 | POSTN/PPARGC1A/MYOCD/ADIPOQ/PDGFD/TPM1/BCL2/IGFBP5/PPARD/PLAT | 10 |
| BP | GO:0051384 | response to glucocorticoid | 13/706 | 135/18862 | 0.001678976 | 0.026893738 | 0.02159402 | ACSBG1/HSD3B1/CLDN1/FIBIN/ADIPOQ/FOS/BCL2/CCND1/FBXO32/FOSL1/TYMS/ARG1/GJB2 | 13 |
| BP | GO:0046890 | regulation of lipid biosynthetic process | 17/706 | 203/18862 | 0.001684729 | 0.026893738 | 0.02159402 | THRSP/SERPINA12/SORBS1/PDK4/PPARGC1A/APOC1/ADIPOQ/ACADL/FASN/APOE/SQLE/ABCG1/RAB38/SMPD3/LDLR/PLA2G3/FABP5 | 17 |
| BP | GO:0002688 | regulation of leukocyte chemotaxis | 12/706 | 119/18862 | 0.001686636 | 0.026893738 | 0.02159402 | CCL27/GREM1/PTN/CCL19/CCL2/NOD2/CCR7/WNT5A/CXCL13/CXCL8/CXCL10/S100A7 | 12 |
| BP | GO:0032615 | interleukin-12 production | 8/706 | 60/18862 | 0.00169698 | 0.026893738 | 0.02159402 | CCL19/LTB/CD36/IRF8/IDO1/NOD2/CCR7/CLEC7A | 8 |
| BP | GO:0032655 | regulation of interleukin-12 production | 8/706 | 60/18862 | 0.00169698 | 0.026893738 | 0.02159402 | CCL19/LTB/CD36/IRF8/IDO1/NOD2/CCR7/CLEC7A | 8 |
| BP | GO:0043567 | regulation of insulin-like growth factor receptor signaling pathway | 5/706 | 24/18862 | 0.001702379 | 0.026893738 | 0.02159402 | CILP/AR/IGFBP6/IGFBP5/CDH3 | 5 |
| BP | GO:0009409 | response to cold | 7/706 | 47/18862 | 0.001705199 | 0.026893738 | 0.02159402 | PPARGC1A/ADRB2/FOS/LPL/ADRB1/PLAC8/CXCL10 | 7 |
| BP | GO:0030857 | negative regulation of epithelial cell differentiation | 7/706 | 47/18862 | 0.001705199 | 0.026893738 | 0.02159402 | SPRY2/FRZB/CCND1/EZH2/ID1/STAT1/MMP9 | 7 |
| BP | GO:0045444 | fat cell differentiation | 18/706 | 221/18862 | 0.001706316 | 0.026893738 | 0.02159402 | WIF1/ZBTB16/PPARGC1A/GATA3/ADRB2/RORA/ADIPOQ/PNPLA3/LPL/FRZB/ADRB1/CCND1/TFAP2B/PPARD/PLAC8/LRG1/WNT5A/ZC3H12A | 18 |
| BP | GO:0046503 | glycerolipid catabolic process | 9/706 | 74/18862 | 0.001723427 | 0.027042048 | 0.021713104 | FABP7/ABHD12B/APOC1/PNPLA3/LPL/LDLR/LIPG/FABP5/GDPD3 | 9 |
| BP | GO:0022409 | positive regulation of cell-cell adhesion | 21/706 | 276/18862 | 0.001726795 | 0.027042048 | 0.021713104 | ZBTB16/GATA3/CCL19/IL4R/SLC7A1/CCL2/PYCARD/IL7R/FUT3/LCK/CD24/PRKCQ/SELE/NFKBIZ/NOD2/CCR7/VNN1/PNP/WNT5A/CD274/CXCL13 | 21 |
| BP | GO:0010038 | response to metal ion | 25/706 | 352/18862 | 0.001738195 | 0.027133617 | 0.021786628 | CHP2/AQP9/PPARGC1A/EEF2K/CLDN1/FIBIN/SYT8/CRIP1/FOS/PLN/BCL2/KCNMB4/CCND1/HMOX1/PLSCR1/ECT2/SMPD3/PPIF/MMP9/GGH/TIGAR/ARG1/WNT5A/CCNB1/S100A8 | 25 |
| BP | GO:1903131 | mononuclear cell differentiation | 28/706 | 411/18862 | 0.001763089 | 0.027434563 | 0.022028269 | ZBTB16/LEPR/SLC46A2/GATA3/RORA/ITM2A/FASN/BCL2/LY6D/IFI16/CCL19/EZH2/BLNK/IRF8/FZD5/IL4R/PRDM1/IL7R/CD2/LCK/GPR68/NFKBIZ/CCR7/VNN1/PNP/IRF7/RSAD2/ZC3H12A | 28 |
| BP | GO:0042058 | regulation of epidermal growth factor receptor signaling pathway | 10/706 | 89/18862 | 0.001792896 | 0.027809815 | 0.022329574 | BTC/SPRY2/BCAR3/TGFA/HBEGF/AREG/AFAP1L2/EREG/MMP9/IFI6 | 10 |
| BP | GO:0001676 | long-chain fatty acid metabolic process | 12/706 | 120/18862 | 0.001811865 | 0.028015097 | 0.022494402 | ELOVL3/ACSBG1/FADS1/FADS2/HPGDS/ACADL/PNPLA3/SLC27A4/PLA2G2F/CYP2C18/CYP2E1/ALOX12B | 12 |
| BP | GO:0071356 | cellular response to tumor necrosis factor | 22/706 | 296/18862 | 0.001826262 | 0.028148639 | 0.022601628 | POSTN/PPARGC1A/GATA3/CLDN1/RORA/ADIPOQ/CCL19/LTB/CCL22/SMPD3/AIM2/PSME2/TNFRSF21/CCL2/PYCARD/TXNDC17/STAT1/GBP1/CXCL8/CCL18/ZC3H12A/CCL20 | 22 |
| BP | GO:0045088 | regulation of innate immune response | 23/706 | 315/18862 | 0.001832262 | 0.028152302 | 0.02260457 | MUC7/APOE/IFI16/RAET1E/SERPINB9/RASGRP1/PLSCR1/AIM2/LRP8/NMI/PSME2/PYCARD/EREG/STAT1/NOD2/SOCS3/POLR3G/PARP9/ARG1/IRF7/WNT5A/MMP12/SERPINB4 | 23 |
| BP | GO:0006066 | alcohol metabolic process | 26/706 | 373/18862 | 0.001846275 | 0.028278687 | 0.022706049 | SERPINA12/LEPR/RBP4/ADH1B/PLCB4/CYP39A1/HMGCS2/ACADL/FASN/APOE/SQLE/PPARD/ABCG1/RDH12/CYP7B1/CH25H/GK/GCH1/LDLR/DHRS9/MOXD1/APOL1/IDH3A/ALDH1A3/GDPD3/AKR1B10 | 26 |
| BP | GO:0010820 | positive regulation of T cell chemotaxis | 4/706 | 15/18862 | 0.001909573 | 0.028971322 | 0.023262192 | CCL27/WNT5A/CXCL13/S100A7 | 4 |
| BP | GO:0051315 | attachment of mitotic spindle microtubules to kinetochore | 4/706 | 15/18862 | 0.001909573 | 0.028971322 | 0.023262192 | NUF2/AURKB/KIF2C/NDC80 | 4 |
| BP | GO:1900006 | positive regulation of dendrite development | 4/706 | 15/18862 | 0.001909573 | 0.028971322 | 0.023262192 | PTN/COBL/EZH2/LRP8 | 4 |
| BP | GO:0060541 | respiratory system development | 16/706 | 188/18862 | 0.00192999 | 0.028971322 | 0.023262192 | HSD11B1/WNT2B/RBP4/PTN/MYOCD/SPRY2/IGFBP5/ESRP2/SMPD3/EIF4EBP1/ALDH1A3/ABCA12/ARG1/SLC7A11/WNT5A/MMP12 | 16 |
| BP | GO:0035850 | epithelial cell differentiation involved in kidney development | 7/706 | 48/18862 | 0.001933003 | 0.028971322 | 0.023262192 | GREM1/GATA3/CD34/ADIPOQ/CD24/STAT1/MMP9 | 7 |
| BP | GO:0043616 | keratinocyte proliferation | 7/706 | 48/18862 | 0.001933003 | 0.028971322 | 0.023262192 | PPARD/FERMT1/AREG/KLK8/EREG/CDH3/TGM1 | 7 |
| BP | GO:0045778 | positive regulation of ossification | 7/706 | 48/18862 | 0.001933003 | 0.028971322 | 0.023262192 | ZBTB16/OSR2/PTN/ADRB2/WNT5A/ISG15/LTF | 7 |
| BP | GO:0003073 | regulation of systemic arterial blood pressure | 10/706 | 90/18862 | 0.001950957 | 0.029150991 | 0.023406455 | AGTR1/POSTN/CORO2B/DDAH1/AR/ADRB2/CAMK2N1/TPM1/ADRB1/KCNK6 | 10 |
| BP | GO:0051302 | regulation of cell division | 15/706 | 171/18862 | 0.001963826 | 0.029253822 | 0.023489022 | BTC/PTN/PDGFD/PDGFC/SUSD2/AURKB/KIF18B/AURKA/TGFA/ECT2/E2F8/PRC1/EREG/KIF20A/C10orf99 | 15 |
| BP | GO:0006213 | pyrimidine nucleoside metabolic process | 6/706 | 36/18862 | 0.00200837 | 0.02964621 | 0.023804086 | UCK2/NT5C3A/TK1/APOBEC3B/UPP1/TYMP | 6 |
| BP | GO:0014912 | negative regulation of smooth muscle cell migration | 6/706 | 36/18862 | 0.00200837 | 0.02964621 | 0.023804086 | PPARGC1A/MYOCD/ADIPOQ/TPM1/IGFBP5/PPARD | 6 |
| BP | GO:0042092 | type 2 immune response | 6/706 | 36/18862 | 0.00200837 | 0.02964621 | 0.023804086 | GATA3/IL4R/IDO1/NOD2/ARG1/RSAD2 | 6 |
| BP | GO:0018958 | phenol-containing compound metabolic process | 11/706 | 106/18862 | 0.002062218 | 0.030182157 | 0.024234419 | GATA3/BCL2/PMEL/GCH1/DUOXA1/MOXD1/CYP2E1/SLC16A10/CDH3/SLC7A11/WNT5A | 11 |
| BP | GO:0001562 | response to protozoan | 5/706 | 25/18862 | 0.002063209 | 0.030182157 | 0.024234419 | IRF8/IL4R/GBP6/ARG1/CLEC7A | 5 |
| BP | GO:0042832 | defense response to protozoan | 5/706 | 25/18862 | 0.002063209 | 0.030182157 | 0.024234419 | IRF8/IL4R/GBP6/ARG1/CLEC7A | 5 |
| BP | GO:0006027 | glycosaminoglycan catabolic process | 8/706 | 62/18862 | 0.002100665 | 0.030456522 | 0.024454716 | OGN/LYVE1/OMD/SDC2/PGLYRP4/HMMR/HYAL4/HPSE | 8 |
| BP | GO:0032613 | interleukin-10 production | 8/706 | 62/18862 | 0.002100665 | 0.030456522 | 0.024454716 | CD34/TNFRSF21/IDO1/PYCARD/NOD2/CLEC7A/ISG15/CD274 | 8 |
| BP | GO:1901570 | fatty acid derivative biosynthetic process | 8/706 | 62/18862 | 0.002100665 | 0.030456522 | 0.024454716 | ELOVL3/ACSBG1/FAR2/SCD5/HMGCS2/FASN/ELOVL7/BDH1 | 8 |
| BP | GO:0043122 | regulation of I-kappaB kinase/NF-kappaB signaling | 19/706 | 244/18862 | 0.002159212 | 0.031106262 | 0.024976418 | RORA/ADIPOQ/ESR1/PELI2/CCL19/CD36/HMOX1/ECT2/TRIM22/TNIP3/PYCARD/STAT1/TRIM14/NOD2/CCR7/WNT5A/ZC3H12A/LTF/S100A12 | 19 |
| BP | GO:0090596 | sensory organ morphogenesis | 19/706 | 244/18862 | 0.002159212 | 0.031106262 | 0.024976418 | WNT2B/OSR2/SLITRK6/RBP4/PTN/GATA3/SPRY2/CDON/FRZB/BCL2/BCAR3/TFAP2B/FZD5/EPHA2/GRHL3/ALDH1A3/GJB6/CHRNA9/WNT5A | 19 |
| BP | GO:0050921 | positive regulation of chemotaxis | 13/706 | 139/18862 | 0.002181251 | 0.031106262 | 0.024976418 | CCL27/F3/PTN/PDGFD/CCL19/TPBG/CCR7/CXCR4/WNT5A/CXCL13/CXCL8/CXCL10/S100A7 | 13 |
| BP | GO:0009395 | phospholipid catabolic process | 7/706 | 49/18862 | 0.002183677 | 0.031106262 | 0.024976418 | ABHD12B/APOC1/SMPD3/LDLR/LIPG/PLBD1/GDPD3 | 7 |
| BP | GO:0043124 | negative regulation of I-kappaB kinase/NF-kappaB signaling | 7/706 | 49/18862 | 0.002183677 | 0.031106262 | 0.024976418 | RORA/ADIPOQ/ESR1/TNIP3/PYCARD/STAT1/ZC3H12A | 7 |
| BP | GO:0070231 | T cell apoptotic process | 7/706 | 49/18862 | 0.002183677 | 0.031106262 | 0.024976418 | PIP/SLC46A2/IDO1/IL7R/PRKCQ/WNT5A/CD274 | 7 |
| BP | GO:0030856 | regulation of epithelial cell differentiation | 14/706 | 156/18862 | 0.002214729 | 0.031456876 | 0.025257939 | GATA3/SPRY2/FRZB/CCND1/TRIM16/EZH2/IL20/ID1/ZBED2/CD24/STAT1/MMP9/ZDHHC21/SERPINB13 | 14 |
| BP | GO:0046632 | alpha-beta T cell differentiation | 11/706 | 107/18862 | 0.002222767 | 0.031479534 | 0.025276132 | ZBTB16/GATA3/RORA/BCL2/CCL19/IL4R/PRDM1/NFKBIZ/PNP/RSAD2/ZC3H12A | 11 |
| BP | GO:0010948 | negative regulation of cell cycle process | 25/706 | 359/18862 | 0.00226647 | 0.032005704 | 0.025698614 | SUSD2/BCL2/CCND1/FHL1/EZH2/AURKB/CENPF/AURKA/BUB1/E2F8/PSME2/CCL2/NEK2/NABP1/ZWINT/BUB1B/CDK1/MAD2L1/NDC80/PTTG1/TTK/CDC20/RRM2/CCNB1/C10orf99 | 25 |
| BP | GO:0000075 | cell cycle checkpoint | 17/706 | 209/18862 | 0.002295165 | 0.032317512 | 0.025948977 | CCND1/AURKB/CENPF/H2AX/AURKA/BUB1/FBXO6/E2F8/NABP1/ZWINT/BUB1B/CDK1/MAD2L1/NDC80/TTK/CDC20/CCNB1 | 17 |
| BP | GO:0036293 | response to decreased oxygen levels | 25/706 | 360/18862 | 0.002352098 | 0.033023994 | 0.026516239 | POSTN/PTN/PPARGC1A/EEF2K/CD34/DDAH1/RORA/MYOCD/ANG/ADIPOQ/CRYAB/SLC29A1/TGFBR3/BCL2/PPARD/ANGPTL4/HMOX1/EIF4EBP1/PSME2/CCNA2/CD24/HK2/TIGAR/CXCR4/CCNB1 | 25 |
| BP | GO:0030217 | T cell differentiation | 19/706 | 246/18862 | 0.002367348 | 0.033142874 | 0.026611692 | ZBTB16/LEPR/SLC46A2/GATA3/RORA/BCL2/CCL19/FZD5/IL4R/PRDM1/IL7R/CD2/LCK/NFKBIZ/CCR7/VNN1/PNP/RSAD2/ZC3H12A | 19 |
| BP | GO:0042552 | myelination | 12/706 | 124/18862 | 0.002391504 | 0.033385398 | 0.026806424 | ACSBG1/TPPP/FA2H/PTN/PLLP/MYOC/CTSC/TNFRSF21/KLK8/CXCR4/TYMP/KLK6 | 12 |
| BP | GO:0043174 | nucleoside salvage | 4/706 | 16/18862 | 0.00247134 | 0.034088909 | 0.0273713 | UCK2/TK1/UPP1/TYMP | 4 |
| BP | GO:0046134 | pyrimidine nucleoside biosynthetic process | 4/706 | 16/18862 | 0.00247134 | 0.034088909 | 0.0273713 | UCK2/TK1/UPP1/TYMP | 4 |
| BP | GO:0051238 | sequestering of metal ion | 4/706 | 16/18862 | 0.00247134 | 0.034088909 | 0.0273713 | S100A8/LCN2/S100A7/S100A9 | 4 |
| BP | GO:0051709 | regulation of killing of cells of other organism | 4/706 | 16/18862 | 0.00247134 | 0.034088909 | 0.0273713 | SERPINB9/ARG1/CLEC7A/KRT6A | 4 |
| BP | GO:0033598 | mammary gland epithelial cell proliferation | 5/706 | 26/18862 | 0.002476783 | 0.034088909 | 0.0273713 | GATA3/ESR1/CCND1/EPHA2/WNT5A | 5 |
| BP | GO:0002367 | cytokine production involved in immune response | 10/706 | 93/18862 | 0.002493662 | 0.034224813 | 0.027480423 | GATA3/CD36/HMOX1/FZD5/NOD2/SLC7A5/ARG1/CLEC7A/WNT5A/RSAD2 | 10 |
| BP | GO:0050731 | positive regulation of peptidyl-tyrosine phosphorylation | 16/706 | 193/18862 | 0.002518433 | 0.034430485 | 0.027645565 | BTC/GREM1/ADIPOQ/CD36/IL20/TGFA/LRP8/HBEGF/AREG/AFAP1L2/EREG/CD24/NOD2/SOCS3/PARP9/CLEC7A | 16 |
| BP | GO:0051091 | positive regulation of DNA-binding transcription factor activity | 20/706 | 266/18862 | 0.002522741 | 0.034430485 | 0.027645565 | GREM1/PPARGC1A/AR/MYOCD/ESR1/CD36/FOSL1/AIM2/LRP8/TRIM22/PYCARD/PRKCQ/IRAK2/TRIM14/NOD2/WNT5A/S100A8/LTF/S100A12/S100A9 | 20 |
| BP | GO:0051310 | metaphase plate congression | 8/706 | 64/18862 | 0.002576237 | 0.035062656 | 0.028153159 | NUF2/CDCA5/AURKB/CENPF/KIF2C/FAM83D/NDC80/CCNB1 | 8 |
| BP | GO:0043406 | positive regulation of MAP kinase activity | 18/706 | 230/18862 | 0.002641196 | 0.035846899 | 0.028782858 | IGFBP6/PDGFD/PDGFC/CCL19/EZH2/MAP3K9/RASGRP1/FZD5/TGFA/FPR1/CD24/IRAK2/CDK1/NOD2/PBK/CXCR4/WNT5A/S100A12 | 18 |
| BP | GO:0050870 | positive regulation of T cell activation | 17/706 | 212/18862 | 0.002663776 | 0.035867063 | 0.028799049 | ZBTB16/GATA3/CCL19/IL4R/SLC7A1/CCL2/PYCARD/IL7R/LCK/CD24/PRKCQ/NFKBIZ/NOD2/CCR7/VNN1/PNP/CD274 | 17 |
| BP | GO:0002683 | negative regulation of immune system process | 27/706 | 403/18862 | 0.002664894 | 0.035867063 | 0.028799049 | GAL/GREM1/LRRC17/APOD/ADIPOQ/IL17D/IFI16/PLA2G2F/SERPINB9/HMOX1/IL4R/NMI/TNFRSF21/IDO1/LDLR/CCL2/IL7R/GPR68/NOD2/GBP1/SAMSN1/ARG1/CD274/ZC3H12A/MMP12/LTF/SERPINB4 | 27 |
| BP | GO:0048286 | lung alveolus development | 6/706 | 38/18862 | 0.002672045 | 0.035867063 | 0.028799049 | MYOCD/IGFBP5/SMPD3/ABCA12/SLC7A11/MMP12 | 6 |
| BP | GO:0071398 | cellular response to fatty acid | 6/706 | 38/18862 | 0.002672045 | 0.035867063 | 0.028799049 | PDK4/PPARGC1A/CLDN1/LPL/CCNB1/ZC3H12A | 6 |
| BP | GO:0051480 | regulation of cytosolic calcium ion concentration | 24/706 | 344/18862 | 0.002681313 | 0.035892867 | 0.028819768 | AGTR1/CACNA2D1/MCOLN3/DMD/ESR1/ACKR4/ATP1A2/PLN/BCL2/CCL19/CXCL11/CD36/FPR1/GNA15/LCK/CD24/CCR7/CXCR4/CHRNA9/CXCR2/WNT5A/CXCL9/CXCL13/CXCL10 | 24 |
| BP | GO:0048145 | regulation of fibroblast proliferation | 9/706 | 79/18862 | 0.002721286 | 0.036229439 | 0.029090014 | BTC/ESR1/PDGFD/PDGFC/IFI30/EREG/CCNA2/WNT5A/CCNB1 | 9 |
| BP | GO:0051303 | establishment of chromosome localization | 9/706 | 79/18862 | 0.002721286 | 0.036229439 | 0.029090014 | NUF2/CDCA5/AURKB/CENPF/KIF2C/FAM83D/NDC80/DLGAP5/CCNB1 | 9 |
| BP | GO:0008217 | regulation of blood pressure | 15/706 | 177/18862 | 0.002743836 | 0.036430384 | 0.029251361 | AGTR1/POSTN/CD34/CORO2B/DDAH1/AR/ADRB2/ADIPOQ/CAMK2N1/ATP1A2/TPM1/ADRB1/KCNK6/HMOX1/GCH1 | 15 |
| BP | GO:0090130 | tissue migration | 25/706 | 365/18862 | 0.002822734 | 0.037248634 | 0.029908365 | GREM1/ACTG2/PTN/GATA3/MEOX2/EFNB2/RHOB/APOE/PPARD/HMOX1/PRSS3P2/ANLN/FERMT1/HBEGF/S100P/ID1/EPHA2/MMP9/S100A2/PRSS3/FGFBP1/WNT5A/CXCL13/ZC3H12A/KRT16 | 25 |
| BP | GO:0019318 | hexose metabolic process | 19/706 | 250/18862 | 0.002834453 | 0.037248634 | 0.029908365 | SERPINA12/LEPR/SORBS1/PDK4/RBP4/PPARGC1A/PGM5/APOD/RORA/ADIPOQ/TFAP2B/PPARD/PHLDA2/PGM2/HK2/FABP5/FUT2/TIGAR/OAS1 | 19 |
| BP | GO:0032729 | positive regulation of interferon-gamma production | 8/706 | 65/18862 | 0.002843582 | 0.037248634 | 0.029908365 | IRF8/RASGRP1/FZD5/PYCARD/CD2/SLC7A5/CLEC7A/WNT5A | 8 |
| BP | GO:0034381 | plasma lipoprotein particle clearance | 8/706 | 65/18862 | 0.002843582 | 0.037248634 | 0.029908365 | APOC1/ADIPOQ/APOE/CD36/HMOX1/LDLR/LIPG/IL19 | 8 |
| BP | GO:0090307 | mitotic spindle assembly | 8/706 | 65/18862 | 0.002843582 | 0.037248634 | 0.029908365 | KIF2A/AURKB/KIF11/PRC1/KIF4A/NEK2/TPX2/CDC20 | 8 |
| BP | GO:0043405 | regulation of MAP kinase activity | 22/706 | 307/18862 | 0.002864137 | 0.037417571 | 0.030044011 | ADIPOQ/IGFBP6/PDGFD/PDGFC/SPRY2/APOE/CCL19/EZH2/MAP3K9/RASGRP1/FZD5/TGFA/FPR1/CD24/IRAK2/CDK1/NOD2/PBK/CXCR4/WNT5A/SERPINB3/S100A12 | 22 |
| BP | GO:0007143 | female meiotic nuclear division | 5/706 | 27/18862 | 0.002947362 | 0.038198442 | 0.030671003 | AURKA/EREG/TTK/TOP2A/CCNB2 | 5 |
| BP | GO:0007263 | nitric oxide mediated signal transduction | 5/706 | 27/18862 | 0.002947362 | 0.038198442 | 0.030671003 | GUCY1A2/DDAH1/APOE/CD36/FPR1 | 5 |
| BP | GO:0060512 | prostate gland morphogenesis | 5/706 | 27/18862 | 0.002947362 | 0.038198442 | 0.030671003 | AR/CRIP1/ESR1/CYP7B1/WNT5A | 5 |
| BP | GO:0048144 | fibroblast proliferation | 9/706 | 80/18862 | 0.00296688 | 0.038280783 | 0.030737117 | BTC/ESR1/PDGFD/PDGFC/IFI30/EREG/CCNA2/WNT5A/CCNB1 | 9 |
| BP | GO:0022612 | gland morphogenesis | 11/706 | 111/18862 | 0.002969385 | 0.038280783 | 0.030737117 | PTN/AR/CRIP1/ESR1/BCL2/IGFBP5/ESRP2/CYP7B1/EPHA2/ARG1/WNT5A | 11 |
| BP | GO:0071827 | plasma lipoprotein particle organization | 7/706 | 52/18862 | 0.00308803 | 0.039605525 | 0.031800804 | AGTR1/APOC1/LPL/APOE/ABCG1/LIPG/PLA2G3 | 7 |
| BP | GO:0001666 | response to hypoxia | 24/706 | 348/18862 | 0.003108533 | 0.039605525 | 0.031800804 | POSTN/PTN/PPARGC1A/CD34/DDAH1/RORA/MYOCD/ANG/ADIPOQ/CRYAB/SLC29A1/TGFBR3/BCL2/PPARD/ANGPTL4/HMOX1/EIF4EBP1/PSME2/CCNA2/CD24/HK2/TIGAR/CXCR4/CCNB1 | 24 |
| BP | GO:0030193 | regulation of blood coagulation | 8/706 | 66/18862 | 0.003132067 | 0.039605525 | 0.031800804 | TSPAN8/F3/CD34/APOE/CD36/F12/PLAT/HPSE | 8 |
| BP | GO:0055088 | lipid homeostasis | 14/706 | 162/18862 | 0.003134348 | 0.039605525 | 0.031800804 | TLCD4/CYP39A1/RORA/ACOX2/PNPLA3/LPL/APOE/ABCG1/ANGPTL4/CYP7B1/LDLR/LIPG/CD24/ABCA12 | 14 |
| BP | GO:0009162 | deoxyribonucleoside monophosphate metabolic process | 4/706 | 17/18862 | 0.003136991 | 0.039605525 | 0.031800804 | TYMS/TK1/UPP1/TYMP | 4 |
| BP | GO:0034374 | low-density lipoprotein particle remodeling | 4/706 | 17/18862 | 0.003136991 | 0.039605525 | 0.031800804 | AGTR1/APOE/ABCG1/PLA2G3 | 4 |
| BP | GO:0051782 | negative regulation of cell division | 4/706 | 17/18862 | 0.003136991 | 0.039605525 | 0.031800804 | SUSD2/AURKB/E2F8/C10orf99 | 4 |
| BP | GO:1904355 | positive regulation of telomere capping | 4/706 | 17/18862 | 0.003136991 | 0.039605525 | 0.031800804 | PKIB/AURKB/NEK2/PRKCQ | 4 |
| BP | GO:1901184 | regulation of ERBB signaling pathway | 10/706 | 96/18862 | 0.00315117 | 0.039682006 | 0.031862214 | BTC/SPRY2/BCAR3/TGFA/HBEGF/AREG/AFAP1L2/EREG/MMP9/IFI6 | 10 |
| BP | GO:1903039 | positive regulation of leukocyte cell-cell adhesion | 18/706 | 234/18862 | 0.003176762 | 0.039901437 | 0.032038403 | ZBTB16/GATA3/CCL19/IL4R/SLC7A1/CCL2/PYCARD/IL7R/LCK/CD24/PRKCQ/SELE/NFKBIZ/NOD2/CCR7/VNN1/PNP/CD274 | 18 |
| BP | GO:0014910 | regulation of smooth muscle cell migration | 9/706 | 81/18862 | 0.003229635 | 0.040358042 | 0.032405029 | POSTN/PPARGC1A/MYOCD/ADIPOQ/PDGFD/TPM1/BCL2/IGFBP5/PPARD | 9 |
| BP | GO:0050000 | chromosome localization | 9/706 | 81/18862 | 0.003229635 | 0.040358042 | 0.032405029 | NUF2/CDCA5/AURKB/CENPF/KIF2C/FAM83D/NDC80/DLGAP5/CCNB1 | 9 |
| BP | GO:0005996 | monosaccharide metabolic process | 20/706 | 272/18862 | 0.003256077 | 0.040584675 | 0.032587001 | SERPINA12/LEPR/SORBS1/PDK4/RBP4/PPARGC1A/PGM5/APOD/RORA/ADIPOQ/TFAP2B/PPARD/SLC23A2/PHLDA2/PGM2/HK2/FABP5/FUT2/TIGAR/OAS1 | 20 |
| BP | GO:0033044 | regulation of chromosome organization | 20/706 | 273/18862 | 0.003394108 | 0.042197485 | 0.033881988 | PPARGC1A/GATA3/PKIB/CDCA5/AURKB/CENPF/BUB1/NEK2/ZWINT/BUB1B/PRKCQ/UBE2C/MAD2L1/NDC80/PTTG1/TTK/TOP2A/CDC20/DLGAP5/CCNB1 | 20 |
| BP | GO:0001909 | leukocyte mediated cytotoxicity | 11/706 | 113/18862 | 0.003411875 | 0.042310718 | 0.033972908 | CTSC/RAET1E/SERPINB9/RASGRP1/IL7R/ULBP2/DNASE1L3/SLAMF7/ARG1/GZMB/SERPINB4 | 11 |
| BP | GO:0046637 | regulation of alpha-beta T cell differentiation | 8/706 | 67/18862 | 0.003442795 | 0.042511279 | 0.034133946 | ZBTB16/GATA3/CCL19/IL4R/PRDM1/NFKBIZ/PNP/ZC3H12A | 8 |
| BP | GO:0010332 | response to gamma radiation | 7/706 | 53/18862 | 0.00344545 | 0.042511279 | 0.034133946 | GATA3/CRYAB/TSPYL5/BCL2/H2AX/TIGAR/CXCL10 | 7 |
| BP | GO:0032607 | interferon-alpha production | 5/706 | 28/18862 | 0.003479222 | 0.042602988 | 0.034207583 | NMI/IFIH1/STAT1/IRF7/MMP12 | 5 |
| BP | GO:0032647 | regulation of interferon-alpha production | 5/706 | 28/18862 | 0.003479222 | 0.042602988 | 0.034207583 | NMI/IFIH1/STAT1/IRF7/MMP12 | 5 |
| BP | GO:0033028 | myeloid cell apoptotic process | 5/706 | 28/18862 | 0.003479222 | 0.042602988 | 0.034207583 | ADIPOQ/BCL2/NOD2/SLC7A11/IRF7 | 5 |
| BP | GO:0060443 | mammary gland morphogenesis | 6/706 | 40/18862 | 0.00348776 | 0.042602988 | 0.034207583 | AR/ESR1/IGFBP5/EPHA2/ARG1/WNT5A | 6 |
| BP | GO:0042445 | hormone metabolic process | 17/706 | 218/18862 | 0.003549701 | 0.043251469 | 0.034728273 | GAL/HSD3B1/RBP4/PPARGC1A/GATA3/ADH1B/ESR1/RDH12/CYP2C18/DUOXA1/HSD17B2/DHRS9/ALDH1A3/CRABP2/SLC16A10/KLK6/AKR1B10 | 17 |
| BP | GO:0050830 | defense response to Gram-positive bacterium | 10/706 | 98/18862 | 0.003661312 | 0.044500424 | 0.035731107 | ANG/PGLYRP4/CD36/IL7R/EPHA2/NOD2/RNASE7/GBP6/KRT6A/C10orf99 | 10 |
| BP | GO:0051701 | biological process involved in interaction with host | 17/706 | 219/18862 | 0.003718674 | 0.045085466 | 0.03620086 | CLDN1/EFNB2/SERPINB9/PLSCR1/TRIM22/LDLR/KPNA2/EPHA2/TYMS/CDK1/TRIM14/IFIT1/TMPRSS4/CXCR4/KRT6A/CXCL8/SERPINB3 | 17 |
| BP | GO:0007292 | female gamete generation | 12/706 | 131/18862 | 0.003764482 | 0.045527873 | 0.036556085 | LGR5/PTN/ANG/BCL2/FOSL1/AURKA/IL4R/EREG/TTK/TOP2A/CCNB2/CCNB1 | 12 |
| BP | GO:0032102 | negative regulation of response to external stimulus | 26/706 | 394/18862 | 0.003867519 | 0.04587417 | 0.036834141 | TSPAN8/GREM1/GATA3/CD34/RORA/ADIPOQ/APOE/IFI16/PPARD/MFHAS1/SERPINB9/NMI/LDLR/CCL2/KLK8/F12/NOD2/SOCS3/PBK/ARG1/PLAT/WNT5A/CXCL13/MMP12/LTF/SERPINB4 | 26 |
| BP | GO:0048469 | cell maturation | 14/706 | 166/18862 | 0.003905624 | 0.04587417 | 0.036834141 | GATA3/ANG/BCL2/GLDN/MYOC/CCL19/TMEM79/FZD5/AURKA/EREG/TYMS/PLA2G3/CLEC7A/CCNB1 | 14 |
| BP | GO:0002523 | leukocyte migration involved in inflammatory response | 4/706 | 18/18862 | 0.003915172 | 0.04587417 | 0.036834141 | PTN/SELE/S100A8/S100A9 | 4 |
| BP | GO:0010885 | regulation of cholesterol storage | 4/706 | 18/18862 | 0.003915172 | 0.04587417 | 0.036834141 | LPL/PPARD/ABCG1/CD36 | 4 |
| BP | GO:0032780 | negative regulation of ATPase activity | 4/706 | 18/18862 | 0.003915172 | 0.04587417 | 0.036834141 | PLN/PPIF/IFIT1/LTF | 4 |
| BP | GO:0033189 | response to vitamin A | 4/706 | 18/18862 | 0.003915172 | 0.04587417 | 0.036834141 | MAP1B/PPARD/TYMS/ARG1 | 4 |
| BP | GO:0034433 | steroid esterification | 4/706 | 18/18862 | 0.003915172 | 0.04587417 | 0.036834141 | AGTR1/APOC1/APOE/ABCG1 | 4 |
| BP | GO:0034434 | sterol esterification | 4/706 | 18/18862 | 0.003915172 | 0.04587417 | 0.036834141 | AGTR1/APOC1/APOE/ABCG1 | 4 |
| BP | GO:0034435 | cholesterol esterification | 4/706 | 18/18862 | 0.003915172 | 0.04587417 | 0.036834141 | AGTR1/APOC1/APOE/ABCG1 | 4 |
| BP | GO:0070269 | pyroptosis | 4/706 | 18/18862 | 0.003915172 | 0.04587417 | 0.036834141 | GSDMC/AIM2/GZMA/GZMB | 4 |
| BP | GO:0071605 | monocyte chemotactic protein-1 production | 4/706 | 18/18862 | 0.003915172 | 0.04587417 | 0.036834141 | APOD/ADIPOQ/NOD2/CLEC7A | 4 |
| BP | GO:0071637 | regulation of monocyte chemotactic protein-1 production | 4/706 | 18/18862 | 0.003915172 | 0.04587417 | 0.036834141 | APOD/ADIPOQ/NOD2/CLEC7A | 4 |
| BP | GO:2000696 | regulation of epithelial cell differentiation involved in kidney development | 4/706 | 18/18862 | 0.003915172 | 0.04587417 | 0.036834141 | GATA3/CD24/STAT1/MMP9 | 4 |
| BP | GO:0002702 | positive regulation of production of molecular mediator of immune response | 10/706 | 99/18862 | 0.003939813 | 0.046052451 | 0.03697729 | RBP4/GATA3/CD36/FZD5/IL4R/NOD2/SLC7A5/CLEC7A/WNT5A/RSAD2 | 10 |
| BP | GO:0030850 | prostate gland development | 6/706 | 41/18862 | 0.003958812 | 0.046054184 | 0.036978681 | AR/PSAPL1/CRIP1/ESR1/CYP7B1/WNT5A | 6 |
| BP | GO:0050691 | regulation of defense response to virus by host | 6/706 | 41/18862 | 0.003958812 | 0.046054184 | 0.036978681 | AIM2/PYCARD/STAT1/PARP9/ZC3H12A/MMP12 | 6 |
| BP | GO:1903706 | regulation of hemopoiesis | 27/706 | 415/18862 | 0.003972784 | 0.046106947 | 0.037021047 | ZBTB16/LRRC17/SLC46A2/PTN/GATA3/ADIPOQ/MTURN/MYL9/FOS/IL17D/CCL19/IL20/IL4R/PRDM1/IL7R/CD2/GPR68/PRKCQ/PLA2G3/STAT1/NFKBIZ/VNN1/PNP/IRF7/ISG15/ZC3H12A/LTF | 27 |
| BP | GO:0046631 | alpha-beta T cell activation | 13/706 | 149/18862 | 0.003993808 | 0.046223297 | 0.037114469 | ZBTB16/GATA3/RORA/BCL2/CCL19/IL4R/PRDM1/PRKCQ/NFKBIZ/PNP/RSAD2/CD274/ZC3H12A | 13 |
| BP | GO:0002833 | positive regulation of response to biotic stimulus | 19/706 | 258/18862 | 0.00400173 | 0.046223297 | 0.037114469 | MUC7/IFI16/RAET1E/RASGRP1/PLSCR1/AIM2/NMI/PSME2/DDX60/PYCARD/EREG/NOD2/POLR3G/PARP9/ARG1/IRF7/WNT5A/CD274/MMP12 | 19 |
| BP | GO:0045940 | positive regulation of steroid metabolic process | 5/706 | 29/18862 | 0.004076629 | 0.046977378 | 0.037719949 | AGTR1/PPARGC1A/APOC1/APOE/ABCG1 | 5 |
| BP | GO:1901987 | regulation of cell cycle phase transition | 30/706 | 478/18862 | 0.004175631 | 0.048005021 | 0.038545083 | SUSD2/BCL2/CCND1/FHL1/CDCA5/EZH2/AURKB/CENPF/AURKA/BUB1/ANLN/E2F8/PSME2/CCL2/NEK2/NABP1/ZWINT/TPX2/BUB1B/HMMR/FAM83D/UBE2C/CDK1/MAD2L1/NDC80/TTK/CDC20/DLGAP5/CCNB1/C10orf99 | 30 |
| BP | GO:0002687 | positive regulation of leukocyte migration | 12/706 | 133/18862 | 0.004255748 | 0.048696923 | 0.039100638 | CCL27/PTN/PDGFD/CCL19/PYCARD/CCR7/WNT5A/CXCL13/CXCL8/CXCL10/CCL20/S100A7 | 12 |
| BP | GO:0050729 | positive regulation of inflammatory response | 12/706 | 133/18862 | 0.004255748 | 0.048696923 | 0.039100638 | AGTR1/LPL/CTSC/NMI/IDO1/PLA2G3/NFKBIZ/CCR7/WNT5A/S100A8/S100A12/S100A9 | 12 |
| BP | GO:0010631 | epithelial cell migration | 24/706 | 357/18862 | 0.004283394 | 0.048898747 | 0.03926269 | GREM1/PTN/GATA3/MEOX2/EFNB2/RHOB/APOE/PPARD/HMOX1/PRSS3P2/ANLN/FERMT1/HBEGF/S100P/ID1/EPHA2/MMP9/S100A2/PRSS3/FGFBP1/WNT5A/CXCL13/ZC3H12A/KRT16 | 24 |
| BP | GO:1902105 | regulation of leukocyte differentiation | 20/706 | 279/18862 | 0.004328763 | 0.049301478 | 0.039586059 | ZBTB16/LRRC17/SLC46A2/GATA3/ADIPOQ/FOS/CCL19/IL20/IL4R/PRDM1/IL7R/CD2/GPR68/PLA2G3/NFKBIZ/VNN1/PNP/IRF7/ZC3H12A/LTF | 20 |
| CC | GO:0001533 | cornified envelope | 14/732 | 45/19520 | 5.45E-10 | 2.44E-07 | 2.07E-07 | CST6/FLG2/SCEL/LORICRIN/IVL/DSG3/DSC2/SPRR1A/SPRR1B/CNFN/TGM1/SPRR3/SPRR2G/PI3 | 14 |
| CC | GO:0062023 | collagen-containing extracellular matrix | 41/732 | 423/19520 | 2.94E-08 | 6.58E-06 | 5.58E-06 | F3/WNT2B/GREM1/CILP/OGN/POSTN/PTN/LAMB4/TNXB/ANG/OMD/ADIPOQ/EDIL3/ELN/FBLN1/CDON/SDC2/APOE/TIMP3/MYOC/SERPINB8/CTSC/SERPINB9/ANGPTL4/C1QB/SLPI/LAD1/SERPINA3/SOST/PLSCR1/LGALS3BP/SERPINA1/COMP/F12/MMP9/SERPINB1/WNT5A/S100A8/ADAMDEC1/S100A7/S100A9 | 41 |
| CC | GO:0000779 | condensed chromosome, centromeric region | 17/732 | 117/19520 | 1.71E-06 | 0.000191343 | 0.000162212 | NUF2/SPC25/AURKB/CENPF/CENPW/BUB1/CENPN/CENPA/NCAPG/NEK2/ZWINT/BUB1B/KIF2C/MAD2L1/NDC80/BIRC5/CCNB1 | 17 |
| CC | GO:0030665 | clathrin-coated vesicle membrane | 17/732 | 117/19520 | 1.71E-06 | 0.000191343 | 0.000162212 | BTC/CD207/ADRB2/HLA-DQB2/NCALD/SLC18A2/APOE/FZD5/FCGR1B/TGFA/HBEGF/AREG/LDLR/IL7R/EREG/EPN3/WNT5A | 17 |
| CC | GO:0000777 | condensed chromosome kinetochore | 15/732 | 106/19520 | 9.61E-06 | 0.000859484 | 0.000728634 | NUF2/SPC25/CENPF/CENPW/BUB1/CENPN/CENPA/NEK2/ZWINT/BUB1B/KIF2C/MAD2L1/NDC80/BIRC5/CCNB1 | 15 |
| CC | GO:0034774 | secretory granule lumen | 29/732 | 322/19520 | 1.30E-05 | 0.000915614 | 0.000776219 | TIMP3/CTSC/SLPI/SERPINA3/AMPD3/PRSS2/S100P/PYCARD/LGALS3BP/SERPINA1/PGM2/FABP5/PLAC8/GM2A/GGH/SERPINB1/ARG1/PNP/LRG1/CXCL1/S100A8/LCN2/HPSE/LTF/S100A7/SERPINB3/S100A12/S100A9/TCN1 | 29 |
| CC | GO:0000776 | kinetochore | 17/732 | 137/19520 | 1.50E-05 | 0.000915614 | 0.000776219 | NUF2/SPC25/AURKB/CENPF/CENPW/BUB1/CENPN/CENPA/NEK2/ZWINT/BUB1B/KIF2C/MAD2L1/NDC80/TTK/BIRC5/CCNB1 | 17 |
| CC | GO:0060205 | cytoplasmic vesicle lumen | 29/732 | 326/19520 | 1.64E-05 | 0.000915614 | 0.000776219 | TIMP3/CTSC/SLPI/SERPINA3/AMPD3/PRSS2/S100P/PYCARD/LGALS3BP/SERPINA1/PGM2/FABP5/PLAC8/GM2A/GGH/SERPINB1/ARG1/PNP/LRG1/CXCL1/S100A8/LCN2/HPSE/LTF/S100A7/SERPINB3/S100A12/S100A9/TCN1 | 29 |
| CC | GO:0031983 | vesicle lumen | 29/732 | 328/19520 | 1.84E-05 | 0.000915614 | 0.000776219 | TIMP3/CTSC/SLPI/SERPINA3/AMPD3/PRSS2/S100P/PYCARD/LGALS3BP/SERPINA1/PGM2/FABP5/PLAC8/GM2A/GGH/SERPINB1/ARG1/PNP/LRG1/CXCL1/S100A8/LCN2/HPSE/LTF/S100A7/SERPINB3/S100A12/S100A9/TCN1 | 29 |
| CC | GO:0000778 | condensed nuclear chromosome kinetochore | 6/732 | 17/19520 | 2.37E-05 | 0.001057251 | 0.000896292 | NUF2/BUB1/CENPA/BUB1B/NDC80/CCNB1 | 6 |
| CC | GO:0016010 | dystrophin-associated glycoprotein complex | 6/732 | 19/19520 | 4.86E-05 | 0.001729395 | 0.001466107 | SNTB1/PGM5/SSPN/DMD/SGCG/KRT19 | 6 |
| CC | GO:0090665 | glycoprotein complex | 6/732 | 19/19520 | 4.86E-05 | 0.001729395 | 0.001466107 | SNTB1/PGM5/SSPN/DMD/SGCG/KRT19 | 6 |
| CC | GO:0000775 | chromosome, centromeric region | 20/732 | 196/19520 | 5.03E-05 | 0.001729395 | 0.001466107 | NUF2/SPC25/CDCA5/AURKB/CENPF/CENPW/BUB1/CENPN/CENPA/NCAPG/NEK2/ZWINT/BUB1B/KIF2C/MAD2L1/NDC80/TTK/TOP2A/BIRC5/CCNB1 | 20 |
| CC | GO:0030669 | clathrin-coated endocytic vesicle membrane | 8/732 | 39/19520 | 8.25E-05 | 0.002634371 | 0.002233306 | CD207/HLA-DQB2/APOE/FZD5/FCGR1B/HBEGF/LDLR/WNT5A | 8 |
| CC | GO:0000940 | condensed chromosome outer kinetochore | 5/732 | 14/19520 | 0.000110427 | 0.00329073 | 0.002789739 | CENPF/BUB1/BUB1B/NDC80/CCNB1 | 5 |
| CC | GO:0000307 | cyclin-dependent protein kinase holoenzyme complex | 8/732 | 43/19520 | 0.000170371 | 0.004759739 | 0.004035102 | CCND1/CCNE2/CCNE1/CCNA2/CDK1/CCNB2/CKS2/CCNB1 | 8 |
| CC | GO:0000793 | condensed chromosome | 20/732 | 217/19520 | 0.000204323 | 0.005372502 | 0.004554576 | NUF2/SPC25/AURKB/CENPF/CENPW/H2AX/BUB1/CENPN/CENPA/NCAPG/NEK2/ZWINT/BUB1B/KIF2C/MAD2L1/NDC80/TOP2A/MKI67/BIRC5/CCNB1 | 20 |
| CC | GO:0005775 | vacuolar lumen | 17/732 | 173/19520 | 0.000283313 | 0.007035616 | 0.005964492 | OGN/OMD/SDC2/CTSC/IFI30/SERPINA3/PRSS2/PYCARD/FABP5/PLAC8/GM2A/GGH/ARG1/SERPINB13/HPSE/S100A7/SERPINB3 | 17 |
| CC | GO:0043034 | costamere | 5/732 | 18/19520 | 0.000417093 | 0.00981267 | 0.00831876 | PGM5/DMD/SYNM/KRT19/AHNAK2 | 5 |
| CC | GO:0035580 | specific granule lumen | 9/732 | 62/19520 | 0.000477585 | 0.010649771 | 0.009028418 | SLPI/GGH/ARG1/LRG1/CXCL1/LCN2/HPSE/LTF/TCN1 | 9 |
| CC | GO:0030662 | coated vesicle membrane | 17/732 | 182/19520 | 0.000512048 | 0.010649771 | 0.009028418 | BTC/CD207/ADRB2/HLA-DQB2/NCALD/SLC18A2/APOE/FZD5/FCGR1B/TGFA/HBEGF/AREG/LDLR/IL7R/EREG/EPN3/WNT5A | 17 |
| CC | GO:0044327 | dendritic spine head | 4/732 | 11/19520 | 0.00052415 | 0.010649771 | 0.009028418 | PPP1R1B/DNM1/MX2/MX1 | 4 |
| CC | GO:0005819 | spindle | 28/732 | 381/19520 | 0.000579811 | 0.011008263 | 0.009332332 | TPPP/KIF2A/AURKB/CENPF/KIF18B/AURKA/SHCBP1/ECT2/KIF11/PRC1/KIF4A/NEK2/TPX2/BUB1B/KIF2C/NUSAP1/FAM83D/CDK1/MAD2L1/TTK/ASPM/FAM110C/KIF20A/BIRC5/CDC20/DLGAP5/CXCR2/CCNB1 | 28 |
| CC | GO:0035578 | azurophil granule lumen | 11/732 | 91/19520 | 0.000591048 | 0.011008263 | 0.009332332 | CTSC/SERPINA3/PRSS2/PYCARD/FABP5/PLAC8/GM2A/GGH/ARG1/S100A7/SERPINB3 | 11 |
| CC | GO:0005766 | primary lysosome | 15/732 | 155/19520 | 0.000742802 | 0.01263139 | 0.010708349 | MGST1/CTSC/SERPINA3/PRSS2/PYCARD/FPR1/FABP5/PLAC8/GM2A/VNN1/GGH/ACP3/ARG1/S100A7/SERPINB3 | 15 |
| CC | GO:0042582 | azurophil granule | 15/732 | 155/19520 | 0.000742802 | 0.01263139 | 0.010708349 | MGST1/CTSC/SERPINA3/PRSS2/PYCARD/FPR1/FABP5/PLAC8/GM2A/VNN1/GGH/ACP3/ARG1/S100A7/SERPINB3 | 15 |
| CC | GO:0045120 | pronucleus | 4/732 | 12/19520 | 0.00076297 | 0.01263139 | 0.010708349 | EZH2/CENPF/AURKA/CCNA2 | 4 |
| CC | GO:0030136 | clathrin-coated vesicle | 17/732 | 192/19520 | 0.000937536 | 0.014850033 | 0.012589219 | BTC/CD207/ADRB2/HLA-DQB2/NCALD/SLC18A2/APOE/FZD5/FCGR1B/TGFA/HBEGF/AREG/LDLR/IL7R/EREG/EPN3/WNT5A | 17 |
| CC | GO:1904724 | tertiary granule lumen | 8/732 | 55/19520 | 0.000963425 | 0.014850033 | 0.012589219 | FLG2/MMP9/PRSS3/GGH/LRG1/CXCL1/LTF/TCN1 | 8 |
| CC | GO:0045334 | clathrin-coated endocytic vesicle | 8/732 | 57/19520 | 0.001224429 | 0.018243996 | 0.015466475 | CD207/HLA-DQB2/APOE/FZD5/FCGR1B/HBEGF/LDLR/WNT5A | 8 |
| CC | GO:0005876 | spindle microtubule | 9/732 | 71/19520 | 0.001300283 | 0.018749246 | 0.015894805 | KIF2A/AURKB/KIF18B/AURKA/KIF11/PRC1/KIF4A/CDK1/BIRC5 | 9 |
| CC | GO:0000922 | spindle pole | 15/732 | 166/19520 | 0.001493746 | 0.020865765 | 0.017689098 | KIF2A/AURKB/CENPF/AURKA/KIF11/PRC1/NEK2/TPX2/FAM83D/MAD2L1/ASPM/FAM110C/CDC20/DLGAP5/CCNB1 | 15 |
| CC | GO:0072686 | mitotic spindle | 14/732 | 157/19520 | 0.002394394 | 0.032433154 | 0.027495433 | TPPP/AURKB/KIF18B/AURKA/ECT2/KIF11/PRC1/TPX2/NUSAP1/FAM83D/CDK1/MAD2L1/ASPM/CXCR2 | 14 |
| CC | GO:0043292 | contractile fiber | 18/732 | 231/19520 | 0.002829039 | 0.036515875 | 0.030956588 | DES/PGM5/MYH11/LMOD1/TPM2/MYL9/DMD/CRYAB/SYNPO2/SYNM/TPM1/KRT19/FBXO32/AHNAK2/CFL2/GLRX3/IDO1/SCO2 | 18 |
| CC | GO:0098687 | chromosomal region | 24/732 | 345/19520 | 0.002859185 | 0.036515875 | 0.030956588 | NUF2/SPC25/CDCA5/EZH2/AURKB/CENPF/CENPW/H2AX/BUB1/CENPN/CENPA/NCAPG/NEK2/NABP1/ZWINT/BUB1B/KIF2C/CDK1/MAD2L1/NDC80/TTK/TOP2A/BIRC5/CCNB1 | 24 |
| MF | GO:0008009 | chemokine activity | 14/705 | 49/18337 | 2.63E-09 | 2.18E-06 | 1.97E-06 | CCL27/CCL19/CXCL11/CCL22/CCL2/CXCL2/CXCL1/CXCL9/CXCL13/CXCL8/CCL18/CXCL10/CCL20/C10orf99 | 14 |
| MF | GO:0005125 | cytokine activity | 29/705 | 235/18337 | 3.13E-08 | 1.30E-05 | 1.17E-05 | CCL27/IL37/WNT2B/GREM1/GREM2/ADIPOQ/IL17D/CCL19/LTB/CXCL11/IL20/CCL22/AREG/CCL2/NAMPT/IL19/CXCL2/IL36A/WNT5A/CXCL1/CXCL9/CXCL13/CXCL8/CCL18/IL36RN/CXCL10/CCL20/C10orf99/IL36G | 29 |
| MF | GO:0042379 | chemokine receptor binding | 14/705 | 69/18337 | 2.95E-07 | 8.15E-05 | 7.37E-05 | CCL27/CCL19/CXCL11/CCL22/CCL2/CXCL2/CXCL1/CXCL9/CXCL13/CXCL8/CCL18/CXCL10/CCL20/C10orf99 | 14 |
| MF | GO:0004252 | serine-type endopeptidase activity | 22/705 | 168/18337 | 5.16E-07 | 0.00010672 | 9.66E-05 | F3/TMPRSS11E/CTSC/PRSS3P2/PRSS2/KLK8/GZMA/F12/PRSS53/MMP9/TMPRSS4/MMP1/PRSS3/PLAT/CFB/KLK10/GZMB/PRSS27/KLK6/KLK13/TMPRSS11D/LTF | 22 |
| MF | GO:0005539 | glycosaminoglycan binding | 26/705 | 228/18337 | 7.62E-07 | 0.000126123 | 0.000114161 | GREM2/POSTN/PTN/LYVE1/TNXB/ANG/CCN5/LPL/DPYSL3/APOE/TGFBR3/PCOLCE2/PGLYRP4/CXCL11/SOST/HBEGF/LIPG/COMP/HMMR/SELL/NOD2/RNASE7/FGFBP1/CXCL13/CXCL10/LTF | 26 |
| MF | GO:0045236 | CXCR chemokine receptor binding | 7/705 | 18/18337 | 2.65E-06 | 0.000308585 | 0.000279319 | CXCL11/CXCL2/CXCL1/CXCL9/CXCL13/CXCL8/CXCL10 | 7 |
| MF | GO:0008236 | serine-type peptidase activity | 22/705 | 186/18337 | 2.92E-06 | 0.000308585 | 0.000279319 | F3/TMPRSS11E/CTSC/PRSS3P2/PRSS2/KLK8/GZMA/F12/PRSS53/MMP9/TMPRSS4/MMP1/PRSS3/PLAT/CFB/KLK10/GZMB/PRSS27/KLK6/KLK13/TMPRSS11D/LTF | 22 |
| MF | GO:0061134 | peptidase regulator activity | 25/705 | 230/18337 | 2.98E-06 | 0.000308585 | 0.000279319 | SERPINA12/CST6/PAPLN/FBLN1/PCOLCE2/TIMP3/SERPINB8/CTSC/SERPINB9/SLPI/SERPINA3/PSME2/PYCARD/SERPINA1/PI15/PTTG1/BIRC5/SERPINB1/WFDC12/SERPINB13/A2ML1/LTF/SERPINB3/PI3/SERPINB4 | 25 |
| MF | GO:0017171 | serine hydrolase activity | 22/705 | 188/18337 | 3.49E-06 | 0.000320882 | 0.00029045 | F3/TMPRSS11E/CTSC/PRSS3P2/PRSS2/KLK8/GZMA/F12/PRSS53/MMP9/TMPRSS4/MMP1/PRSS3/PLAT/CFB/KLK10/GZMB/PRSS27/KLK6/KLK13/TMPRSS11D/LTF | 22 |
| MF | GO:0008201 | heparin binding | 20/705 | 164/18337 | 5.13E-06 | 0.000424425 | 0.000384173 | GREM2/POSTN/PTN/TNXB/ANG/CCN5/LPL/APOE/TGFBR3/PCOLCE2/CXCL11/SOST/HBEGF/LIPG/COMP/SELL/FGFBP1/CXCL13/CXCL10/LTF | 20 |
| MF | GO:0005126 | cytokine receptor binding | 27/705 | 270/18337 | 5.92E-06 | 0.000445881 | 0.000403594 | CCL27/IL37/GREM1/GATA3/PRLR/TGFBR3/CCL19/LTB/CXCL11/IL20/CCL22/CCL2/PYCARD/STAT1/CXCL2/IL36A/LRG1/CXCL1/CXCL9/CXCL13/CXCL8/CCL18/IL36RN/CXCL10/CCL20/C10orf99/IL36G | 27 |
| MF | GO:0048018 | receptor ligand activity | 39/705 | 486/18337 | 1.27E-05 | 0.00087374 | 0.000790876 | BTC/CCL27/GAL/IL37/WNT2B/GREM1/GREM2/OGN/PTN/ADIPOQ/PDGFD/PDGFC/IL17D/CCL19/LTB/CXCL11/IL20/TGFA/CCL22/HBEGF/AREG/CCL2/EREG/NAMPT/IL19/CXCL2/IL36A/WNT5A/CXCL1/CXCL9/CXCL13/TYMP/CXCL8/CCL18/IL36RN/CXCL10/CCL20/C10orf99/IL36G | 39 |
| MF | GO:0030546 | signaling receptor activator activity | 39/705 | 492/18337 | 1.68E-05 | 0.001050941 | 0.000951271 | BTC/CCL27/GAL/IL37/WNT2B/GREM1/GREM2/OGN/PTN/ADIPOQ/PDGFD/PDGFC/IL17D/CCL19/LTB/CXCL11/IL20/TGFA/CCL22/HBEGF/AREG/CCL2/EREG/NAMPT/IL19/CXCL2/IL36A/WNT5A/CXCL1/CXCL9/CXCL13/TYMP/CXCL8/CCL18/IL36RN/CXCL10/CCL20/C10orf99/IL36G | 39 |
| MF | GO:0050786 | RAGE receptor binding | 5/705 | 10/18337 | 1.78E-05 | 0.001050941 | 0.000951271 | FPR1/S100A8/S100A7/S100A12/S100A9 | 5 |
| MF | GO:0004867 | serine-type endopeptidase inhibitor activity | 14/705 | 98/18337 | 2.23E-05 | 0.001228718 | 0.001112189 | SERPINA12/PAPLN/SERPINB8/SERPINB9/SLPI/SERPINA3/SERPINA1/SERPINB1/WFDC12/SERPINB13/A2ML1/SERPINB3/PI3/SERPINB4 | 14 |
| MF | GO:1901681 | sulfur compound binding | 25/705 | 260/18337 | 2.54E-05 | 0.001313682 | 0.001189095 | GREM2/POSTN/PTN/CD34/MGST1/TNXB/ANG/CCN5/ACADL/PNPLA3/LPL/DPYSL3/APOE/TGFBR3/PCOLCE2/CXCL11/SOST/HBEGF/LIPG/COMP/SELL/FGFBP1/CXCL13/CXCL10/LTF | 25 |
| MF | GO:0030414 | peptidase inhibitor activity | 20/705 | 187/18337 | 3.61E-05 | 0.001759319 | 0.001592467 | SERPINA12/CST6/PAPLN/TIMP3/SERPINB8/SERPINB9/SLPI/SERPINA3/SERPINA1/PI15/PTTG1/BIRC5/SERPINB1/WFDC12/SERPINB13/A2ML1/LTF/SERPINB3/PI3/SERPINB4 | 20 |
| MF | GO:0061135 | endopeptidase regulator activity | 20/705 | 192/18337 | 5.26E-05 | 0.002421874 | 0.002192187 | SERPINA12/CST6/PAPLN/TIMP3/SERPINB8/SERPINB9/SLPI/SERPINA3/PSME2/SERPINA1/PTTG1/BIRC5/SERPINB1/WFDC12/SERPINB13/A2ML1/LTF/SERPINB3/PI3/SERPINB4 | 20 |
| MF | GO:0004866 | endopeptidase inhibitor activity | 19/705 | 180/18337 | 6.72E-05 | 0.002928442 | 0.002650713 | SERPINA12/CST6/PAPLN/TIMP3/SERPINB8/SERPINB9/SLPI/SERPINA3/SERPINA1/PTTG1/BIRC5/SERPINB1/WFDC12/SERPINB13/A2ML1/LTF/SERPINB3/PI3/SERPINB4 | 19 |
| MF | GO:0019887 | protein kinase regulator activity | 19/705 | 190/18337 | 0.000138495 | 0.005733704 | 0.005189927 | GREM1/CAB39L/PPP1R1B/PKIB/CAMK2N1/SPRY2/CCND1/CCNE2/AFAP1L2/CCNE1/TPX2/CCNA2/CD24/SOCS3/CCNB2/CKS2/CCNB1/CXCL10/LTF | 19 |
| MF | GO:0004857 | enzyme inhibitor activity | 30/705 | 385/18337 | 0.000208776 | 0.008231752 | 0.007451064 | SERPINA12/CST6/PTN/PAPLN/APOC1/PPP1R1B/PKIB/CAMK2N1/PLN/SPRY2/TIMP3/SERPINB8/SERPINB9/ANGPTL4/SLPI/SERPINA3/SERPINA1/SOCS3/PI15/PTTG1/BIRC5/SERPINB1/PARP9/WFDC12/SERPINB13/A2ML1/LTF/SERPINB3/PI3/SERPINB4 | 30 |
| MF | GO:0019955 | cytokine binding | 15/705 | 136/18337 | 0.000236521 | 0.008901782 | 0.00805755 | LEPR/GREM1/GREM2/PRLR/ACKR4/TGFBR3/TRIM16/CD36/SOST/COMP/GBP1/CCR7/CXCR4/CXCR2/IL36RN | 15 |
| MF | GO:0008017 | microtubule binding | 23/705 | 269/18337 | 0.000305422 | 0.010995193 | 0.009952424 | TPPP/CRYAB/DNM1/SYBU/MAP1B/KIF2A/CENPF/KIF18B/MX2/KIF11/PRC1/KIF4A/TPX2/KIF2C/NUSAP1/FAM83D/KIF20A/BIRC5/GJB6/DLGAP5/MX1/S100A8/S100A9 | 23 |
| MF | GO:0001664 | G protein-coupled receptor binding | 24/705 | 289/18337 | 0.000350677 | 0.011620801 | 0.010518701 | CCL27/GAL/WNT2B/AGTR1/PPP1R1B/ADRB1/MYOC/CCL19/CXCL11/CCL22/CCL2/FPR1/GNA15/RTP4/CXCL2/WNT5A/CXCL1/CXCL9/CXCL13/CXCL8/CCL18/CXCL10/CCL20/C10orf99 | 24 |
| MF | GO:0070851 | growth factor receptor binding | 15/705 | 141/18337 | 0.00035087 | 0.011620801 | 0.010518701 | BTC/IL37/GREM1/GATA3/PDGFD/PDGFC/TGFA/PLSCR1/HBEGF/AREG/PYCARD/EREG/IL36A/IL36RN/IL36G | 15 |
| MF | GO:0002020 | protease binding | 14/705 | 131/18337 | 0.000521421 | 0.016605247 | 0.01503043 | F3/SLC2A13/BCL2/TIMP3/SERPINB9/LDLR/PYCARD/SERPINA1/COMP/SELL/SERPINB13/A2ML1/SERPINB3/SERPINB4 | 14 |
| MF | GO:0005200 | structural constituent of cytoskeleton | 12/705 | 103/18337 | 0.000591551 | 0.018140891 | 0.016420435 | DES/KRT15/DMD/SYNM/TPM1/LORICRIN/KRT19/TUBB6/KRT6B/INA/KRT6A/KRT16 | 12 |
| MF | GO:0019207 | kinase regulator activity | 19/705 | 220/18337 | 0.000877179 | 0.025939442 | 0.023479383 | GREM1/CAB39L/PPP1R1B/PKIB/CAMK2N1/SPRY2/CCND1/CCNE2/AFAP1L2/CCNE1/TPX2/CCNA2/CD24/SOCS3/CCNB2/CKS2/CCNB1/CXCL10/LTF | 19 |
| MF | GO:0008307 | structural constituent of muscle | 7/705 | 42/18337 | 0.001005195 | 0.028700047 | 0.025978176 | MYH11/TPM2/MYL9/DMD/SYNM/TPM1/KRT19 | 7 |
| MF | GO:0015631 | tubulin binding | 27/705 | 368/18337 | 0.001060595 | 0.029272413 | 0.02649626 | TPPP/PPARGC1A/CRYAB/DNM1/SYBU/NCALD/MAP1B/DIXDC1/KIF2A/CENPF/KIF18B/MX2/KIF11/PRC1/KIF4A/TPX2/KIF2C/NUSAP1/FAM83D/FAM110C/KIF20A/BIRC5/GJB6/DLGAP5/MX1/S100A8/S100A9 | 27 |
| MF | GO:0036122 | BMP binding | 4/705 | 13/18337 | 0.001173262 | 0.031337446 | 0.028365449 | GREM1/GREM2/SOST/COMP | 4 |
| MF | GO:0005154 | epidermal growth factor receptor binding | 6/705 | 33/18337 | 0.001440612 | 0.037275848 | 0.033740661 | BTC/TGFA/PLSCR1/HBEGF/AREG/EREG | 6 |
| MF | GO:0048020 | CCR chemokine receptor binding | 7/705 | 46/18337 | 0.001746428 | 0.043819469 | 0.039663694 | CCL27/CCL19/CCL22/CCL2/CXCL13/CCL18/CCL20 | 7 |
