# Supplementary material for: Exploration of Biomarkers of Psoriasis through Combined Multiomics Analysis
Source: Mediators Inflamm. 2022 Sep 23;2022:7731082. doi: 10.1155/2022/7731082 (PMC9525798; doi:10.1155/2022/7731082)
Supplement: Supplementary Materials — Supplementary Figure 1 The PCA of gene expression in psoriasis lesions and healthy controls in GSE13355 database. Supplementary Figure 2 The PCA and methylation distribution density in psoriasis lesions and healthy controls from the GSE73894 dataset. (A) PCA in GSE73894. (B) Methylation distribution density in GSE73894. Supplementary Table 1 Identification of DEGs in the psoriatic lesions and healthy control group in GSE13355. Supplementary Table 2 GO analysis on 767 DEGs in GSE13355. Supplementary Table 3 KEGG analysis on 767 DEGs in GSE13355. Supplementary Table 4 Identification of hyper-MR-genes. Supplementary Table 5 Identification of hypo-MR-genes. Supplementary Table 6 GO analysis of hyper-MR-genes. Supplementary Table 7 GO analysis of hypo-MR-genes. Supplementary Table 8 KEGG analysis of hyper-MR-genes. Supplementary Table 9 KEGG analysis of hypo-MR-genes. Supplementary Table 10 GO analysis through single-gene GSEA of GJB2. Supplementary Table 11 KEGG analysis through single-gene GSEA of GJB2. [file 7731082.f1.zip › Supplementary Table 3 (1).docx]

| KEGG analysis on 767 DEGs in GSE13355 | | | | | | | | |
| --- | --- | --- | --- | --- | --- | --- | --- | --- |
| ID | Description | GeneRatio | BgRatio | pvalue | p.adjust | qvalue | geneID | Count |
| hsa03320 | PPAR signaling pathway | 18/369 | 75/8104 | 4.13E-09 | 1.23E-06 | 1.10E-06 | ACSBG1/FABP7/FADS2/SCD5/SORBS1/ADIPOQ/HMGCS2/ACADL/PLIN1/ACOX2/LPL/SLC27A4/PPARD/CD36/ANGPTL4/GK/FABP5/MMP1 | 18 |
| hsa04061 | Viral protein interaction with cytokine and cytokine receptor | 20/369 | 100/8104 | 1.66E-08 | 2.46E-06 | 2.20E-06 | CCL27/IL37/ACKR4/CCL19/CXCL11/IL20/CCL22/CCL2/CCR7/IL19/CXCL2/CXCR4/CXCR2/CXCL1/CXCL9/CXCL13/CXCL8/CCL18/CXCL10/CCL20 | 20 |
| hsa04657 | IL-17 signaling pathway | 16/369 | 94/8104 | 4.37E-06 | 0.000432341 | 0.000387675 | FOS/IL17D/FOSL1/CCL2/MMP9/MMP1/CXCL2/CXCL1/CXCL8/CXCL10/CCL20/S100A8/LCN2/S100A7/S100A9/S100A7A | 16 |
| hsa04060 | Cytokine-cytokine receptor interaction | 30/369 | 295/8104 | 2.76E-05 | 0.002051756 | 0.001839781 | CCL27/IL37/LEPR/PRLR/ACKR4/IL17D/CCL19/LTB/CXCL11/IL20/IL4R/CCL22/TNFRSF21/CCL2/IL7R/CCR7/IL19/CXCL2/IL36A/CXCR4/CXCR2/CXCL1/CXCL9/CXCL13/CXCL8/CCL18/IL36RN/CXCL10/CCL20/IL36G | 30 |
| hsa00240 | Pyrimidine metabolism | 10/369 | 56/8104 | 0.000184811 | 0.010977746 | 0.009843593 | CTPS1/UCK2/NT5C3A/TYMS/TK1/PNP/CMPK2/UPP1/RRM2/TYMP | 10 |
| hsa05164 | Influenza A | 19/369 | 171/8104 | 0.000272424 | 0.013484978 | 0.012091793 | IRF9/MX2/PRSS2/CCL2/PYCARD/KPNA2/IFIH1/STAT1/TMPRSS4/SOCS3/PRSS3/OAS1/MX1/IRF7/RSAD2/CXCL8/CXCL10/OAS2/TMPRSS11D | 19 |
| hsa05160 | Hepatitis C | 17/369 | 157/8104 | 0.000762556 | 0.03235415 | 0.029011518 | CLDN8/CLDN23/CLDN1/CCND1/IRF9/MX2/CLDN17/LDLR/STAT1/IFIT1/SOCS3/OAS1/MX1/IRF7/RSAD2/CXCL10/OAS2 | 17 |
| hsa04062 | Chemokine signaling pathway | 19/369 | 192/8104 | 0.001152348 | 0.042780936 | 0.038361073 | CCL27/PLCB4/ADCY2/CCL19/CXCL11/CCL22/CCL2/STAT1/CCR7/CXCL2/CXCR4/CXCR2/CXCL1/CXCL9/CXCL13/CXCL8/CCL18/CXCL10/CCL20 | 19 |
| hsa05215 | Prostate cancer | 12/369 | 97/8104 | 0.001429989 | 0.047189651 | 0.042314307 | AR/PDGFD/PDGFC/CREB5/TCF7L1/BCL2/CCND1/TGFA/CCNE2/CCNE1/MMP9/PLAT | 12 |
