# Supplementary material for: Exploration of Biomarkers of Psoriasis through Combined Multiomics Analysis
Source: Mediators Inflamm. 2022 Sep 23;2022:7731082. doi: 10.1155/2022/7731082 (PMC9525798; doi:10.1155/2022/7731082)
Supplement: Supplementary Materials — Supplementary Figure 1 The PCA of gene expression in psoriasis lesions and healthy controls in GSE13355 database. Supplementary Figure 2 The PCA and methylation distribution density in psoriasis lesions and healthy controls from the GSE73894 dataset. (A) PCA in GSE73894. (B) Methylation distribution density in GSE73894. Supplementary Table 1 Identification of DEGs in the psoriatic lesions and healthy control group in GSE13355. Supplementary Table 2 GO analysis on 767 DEGs in GSE13355. Supplementary Table 3 KEGG analysis on 767 DEGs in GSE13355. Supplementary Table 4 Identification of hyper-MR-genes. Supplementary Table 5 Identification of hypo-MR-genes. Supplementary Table 6 GO analysis of hyper-MR-genes. Supplementary Table 7 GO analysis of hypo-MR-genes. Supplementary Table 8 KEGG analysis of hyper-MR-genes. Supplementary Table 9 KEGG analysis of hypo-MR-genes. Supplementary Table 10 GO analysis through single-gene GSEA of GJB2. Supplementary Table 11 KEGG analysis through single-gene GSEA of GJB2. [file 7731082.f1.zip › Supplementary Table 4 (1).docx]

| Identification of hyper-MR-genes |
| --- |

| x |
| --- |
| HOXA5 |
| HOXA6 |
| SKIV2L |
| STK19 |
| RNF39 |
| DDAH2 |
| ZBTB22 |
| TAPBP |
| HOXA4 |
| PPT2 |
| PRRT1 |
| PPP2R3A |
| TSPAN4 |
| ZNF577 |
| C10orf26 |
| TRIM2 |
| EGFL8 |
| HLA-DPB1 |
| PLEC1 |
| HOXA3 |
| CSNK1E |
| MIR199A1 |
| DNM2 |
| SLC27A3 |
| C22orf9 |
| C6orf27 |
| NOTCH4 |
| GPSM3 |
| PHKG1 |
| RWDD3 |
| WFIKKN2 |
| CMYA5 |
| MPZL1 |
| SPG20 |
| PHACTR2 |
| GPR37L1 |
| HOXB3 |
| RUFY1 |
| TNXB |
| SYN3 |
| LRRC2 |
| LOXL1 |
| DNMT3A |
| CECR1 |
| HSPB1 |
| LDHA |
| CALCA |
| RXRB |
| ELF5 |
| SLC39A7 |
| PWWP2B |
| LAMA4 |
| ATP6V0E2 |
| MTSS1 |
| AGTRAP |
| CIT |
| FXYD6 |
| SOD3 |
| C1QTNF7 |
| CD81 |
| COL11A2 |
| 8-Sep |
| SLC12A4 |
| FXYD1 |
| VARS2 |
| GTF2H4 |
| LGALS1 |
| CARD14 |
| TGFB1I1 |
| BAZ2B |
| ESR1 |
| TRIM65 |
| CFL2 |
| VENTX |
| PXDNL |
| THBS1 |
| ALOX12 |
| UCN |
| MCF2L |
| LAMB2 |
| LOC728392 |
| ITGBL1 |
| LOC404266 |
| ZNF454 |
| TBCD |
| LOC399959 |
| SLC44A2 |
| PRKCZ |
| CD59 |
| ABR |
| ZNF280D |
| FEZ1 |
| ANKMY1 |
| BAT2 |
| NSUN7 |
| DLX5 |
| ZNF532 |
| TAGLN3 |
| CLDN6 |
| AQP1 |
| ZNF135 |
| ATP11A |
| SHISA4 |
| CNDP1 |
| ISLR |
| LOC400931 |
| LOC100130872-SPON2 |
| SPON2 |
| SYNE1 |
| C3orf24 |
| NRN1L |
| HIVEP3 |
| WWTR1 |
| SLC22A18AS |
| PDZRN3 |
| GPR81 |
| MBP |
| BRD4 |
| SEC31B |
| CAP2 |
| GRB7 |
| MUM1 |
| KCP |
| HPCAL1 |
| ATF6B |
| ASCL2 |
| RIN1 |
| CCDC105 |
| CCND1 |
| RUSC1 |
| MB |
| HOXB1 |
| NDUFS2 |
| HOXB2 |
| DIXDC1 |
| HLA-DQB2 |
| SLC4A11 |
| HOXC4 |
| GNL1 |
| RILP |
| S100A13 |
| NET1 |
| ZNF662 |
| MAGI2 |
| LFNG |
| PSTPIP2 |
| GNPNAT1 |
| ST7OT4 |
| NFU1 |
| DLEU7 |
| HLA-DOA |
| KRTAP17-1 |
| PDE6B |
| CS |
| PGM3 |
| PPM1G |
| POU2F2 |
| GABBR1 |
| RBM24 |
| ME3 |
| PMEPA1 |
| WBSCR27 |
| CPT1B |
| HLA-DRB1 |
| TRIOBP |
| HLA-DPB2 |
| LOC134466 |
| EMID2 |
| FLOT1 |
| ZNF267 |
| LRBA |
| MAB21L2 |
| EXPH5 |
| PAM |
| KLHL33 |
| LZTS1 |
| COLEC11 |
| MDFI |
| C6orf25 |
| ABAT |
| FES |
| TCF23 |
| FHL2 |
| SLC38A4 |
| ZNF502 |
| RAPSN |
| F10 |
| GRIK2 |
| GEFT |
| KIAA1949 |
| C10orf107 |
| TSSC4 |
| TBC1D16 |
| VWA5B2 |
| ZSCAN1 |
| DNHD1 |
| HAGHL |
| CCDC78 |
| SLC38A7 |
| RGS12 |
| AMOTL1 |
| CDH1 |
| LGALS8 |
| EID3 |
| TXNRD1 |
| FOXA2 |
| AGBL4 |
| DDR1 |
| KDM2B |
| MON1A |
| CDKN1C |
| ZNF790 |
| FOXK1 |
| TRIM26 |
| CLDN11 |
| GLRB |
| GRAMD1B |
| EGFLAM |
| NME5 |
| RPL22 |
| NEUROG1 |
| MIR574 |
| FAM114A1 |
| TMEM88 |
| KDM6B |
| CYR61 |
| CD47 |
| SEMA3B |
| LRRC32 |
| TACC2 |
| GSTT1 |
| C5orf38 |
| ALS2CR11 |
| GOLT1A |
| MIR25 |
| MCM7 |
| ZNF418 |
| CNTN1 |
| IL17RE |
| MOV10L1 |
| MRVI1 |
| FBLN2 |
| CALD1 |
| TP73 |
| WDR8 |
| PKD2L1 |
| FLJ45983 |
| PPP1R9A |
| C6orf47 |
| LOC285370 |
| TEF |
| ADPRHL1 |
| FLJ42875 |
| C20orf166 |
| TMEM159 |
| DNAH3 |
| PRLR |
| FAM180A |
| CORT |
| C10orf25 |
| TNNI2 |
| SYNGAP1 |
| ZBTB47 |
| SMPD3 |
| PLOD2 |
| SACS |
| C11orf90 |
| NEU1 |
| TRIM40 |
| GPT |
| RNASE1 |
| C1QTNF2 |
| HCG9 |
| LOC100302652 |
| GPR75 |
| B4GALNT4 |
| TFAP2E |
| ATP10A |
| MACROD2 |
| RGL2 |
| AGPAT1 |
| CLDN9 |
| TTLL10 |
| BIK |
| FOXG1 |
| SUOX |
| RNF135 |
| FOXI2 |
| SLC44A4 |
| MUPCDH |
| CLDN5 |
| EDNRA |
| LDHD |
| CDK2AP1 |
| MOSC2 |
| PLEKHB1 |
| CYP1A1 |
| C10orf116 |
| ERN2 |
| ALDH3B1 |
| PCDHB3 |
| PRLHR |
| IFFO1 |
| ABHD10 |
| CADPS |
| GGT1 |
| OTOP3 |
| DNALI1 |
| DNAH9 |
| IL34 |
| C1orf51 |
| KCNQ1OT1 |
| KAZALD1 |
| ECE2 |
| GRK1 |
| ZNF167 |
| RING1 |
| ERBB2 |
| ZNF471 |
| CLSTN3 |
| RBP5 |
| WNT2 |
| MYH14 |
| C7orf53 |
| KIFC1 |
| ZNF542 |
| BCL9L |
| BDKRB2 |
| PF4 |
| NAPRT1 |
| TRIM54 |
| ACADM |
| RORA |
| CA3 |
| CASZ1 |
| MRGPRE |
| C6orf154 |
| GPR25 |
| WNK4 |
| HORMAD2 |
| SLFN13 |
| COL1A1 |
| PRDM16 |
| HCCA2 |
| GPD2 |
| MUC4 |
| SLC27A6 |
| TRPC6 |
| TCEA2 |
| C2orf39 |
| TF |
| ELFN1 |
| GAL3ST3 |
| GULP1 |
| MPZL2 |
| C10orf71 |
| THSD7A |
| NFYA |
| SFRP5 |
| LIMS2 |
| ACCN3 |
| MIR589 |
| PRSS16 |
| NR2F1 |
| SLC35C1 |
| SLITRK3 |
| SLC12A7 |
| TULP1 |
| PACS2 |
| HOXD1 |
| FAM84A |
| FAM47E |
| GRB10 |
| EDNRB |
| TBX10 |
| ESM1 |
| VSTM2A |
| NEK3 |
| KCNQ1 |
| LUZP2 |
| PTRF |
| IGFALS |
| FASTK |
| PTGDR |
| PPP1R2P1 |
| ZSCAN18 |
| RPTOR |
| LYPD5 |
| AIFM2 |
| SHANK2 |
| TBPL2 |
| SFRP1 |
| C17orf101 |
| HLA-DRA |
| AIRE |
| TMEM22 |
| HNF1A |
| DSCAM |
| C8orf31 |
| ZNF385D |
| SLIT3 |
| AKAP12 |
| GPR78 |
| C13orf26 |
| IQGAP3 |
| KCNN3 |
| TAP2 |
| PTGFR |
| A4GALT |
| MESTIT1 |
| REC8 |
| FLRT1 |
| MACROD1 |
| C3orf25 |
| MYLK2 |
| NRM |
| TDRD5 |
| BTBD18 |
| PRAP1 |
| KRT72 |
| WDR88 |
| FGF12 |
| PSKH2 |
| C6orf155 |
| GADL1 |
| RNF180 |
| MIR124-2 |
| SCGN |
| WDR46 |
| COX4I2 |
| TRAM1L1 |
| KCNA4 |
| TET1 |
| BRD2 |
| SLC5A5 |
| NCOA4 |
| SLC25A2 |
| LOC154449 |
| NPR3 |
| SYT9 |
| PCDHGA4 |
| KCNAB3 |
| GNG4 |
| MYO15B |
| STAG3 |
| H19 |
| SGEF |
| C3orf27 |
| ADRB3 |
| DFNA5 |
| ITPRIP |
| OR2L13 |
| ZNF560 |
| ZNF214 |
| NLRP14 |
| MOCS1 |
| DBX1 |
| TACSTD2 |
| C10orf11 |
| LOC650226 |
| ZNF578 |
| TBX15 |
| PNMAL1 |
| LOC494141 |
| CTPS |
| CTSD |
| ZNF264 |
| ZNF709 |
| PCSK1 |
| GRB2 |
| PFDN6 |
| GLRA1 |
| BRD1 |
| LOC90834 |
| ASB16 |
| RSPH9 |
| SDK1 |
| ZSCAN12L1 |
| VARS |
| JAKMIP1 |
