# Supplementary material for: Exploration of Biomarkers of Psoriasis through Combined Multiomics Analysis
Source: Mediators Inflamm. 2022 Sep 23;2022:7731082. doi: 10.1155/2022/7731082 (PMC9525798; doi:10.1155/2022/7731082)
Supplement: Supplementary Materials — Supplementary Figure 1 The PCA of gene expression in psoriasis lesions and healthy controls in GSE13355 database. Supplementary Figure 2 The PCA and methylation distribution density in psoriasis lesions and healthy controls from the GSE73894 dataset. (A) PCA in GSE73894. (B) Methylation distribution density in GSE73894. Supplementary Table 1 Identification of DEGs in the psoriatic lesions and healthy control group in GSE13355. Supplementary Table 2 GO analysis on 767 DEGs in GSE13355. Supplementary Table 3 KEGG analysis on 767 DEGs in GSE13355. Supplementary Table 4 Identification of hyper-MR-genes. Supplementary Table 5 Identification of hypo-MR-genes. Supplementary Table 6 GO analysis of hyper-MR-genes. Supplementary Table 7 GO analysis of hypo-MR-genes. Supplementary Table 8 KEGG analysis of hyper-MR-genes. Supplementary Table 9 KEGG analysis of hypo-MR-genes. Supplementary Table 10 GO analysis through single-gene GSEA of GJB2. Supplementary Table 11 KEGG analysis through single-gene GSEA of GJB2. [file 7731082.f1.zip › Supplementary Table 5 (1).docx]

| Identification of hypo-MR-genes |
| --- |

| x |
| --- |
| KIAA1949 |
| C11orf21 |
| TSPAN32 |
| TNXB |
| FLJ45983 |
| GATA3 |
| IER3 |
| FLOT1 |
| PRRT1 |
| ARHGDIB |
| PSMB8 |
| TAP1 |
| TRIM27 |
| BNC1 |
| LTA |
| BLCAP |
| IGF2AS |
| GFI1 |
| TSTD1 |
| FXYD1 |
| FXYD7 |
| HOXA7 |
| TCF7 |
| SLC26A10 |
| MAP1LC3B2 |
| C1orf133 |
| HRH1 |
| TNF |
| CTSZ |
| CCND2 |
| MIR196B |
| C1orf150 |
| PSMB9 |
| VARS |
| HCP5 |
| RBP7 |
| TNFRSF10A |
| CPT1A |
| TM4SF19 |
| MIR596 |
| ZC3H12D |
| RAB36 |
| DEF6 |
| LIME1 |
| HLA-E |
| S100A9 |
| EPSTI1 |
| HSD17B8 |
| THRB |
| RAP1GAP2 |
| HLA-DMA |
| GJB2 |
| MGAT1 |
| ALX4 |
| MSX1 |
| RIPK3 |
| HPN |
| TMC8 |
| SLC22A18AS |
| EYA4 |
| ADM |
| MIR1182 |
| FAM89A |
| TCP11 |
| BIN2 |
| LXN |
| GFM1 |
| DALRD3 |
| NDUFAF3 |
| MIR425 |
| DIABLO |
| TWIST1 |
| ZNF385A |
| PRF1 |
| SHANK2 |
| EIF4E |
| SGPP2 |
| PITX2 |
| LYPD1 |
| WDR66 |
| GRHL3 |
| TTC22 |
| DENND1B |
| SOX7 |
| TNFRSF18 |
| FOXF1 |
| PCGF3 |
| ADORA2A |
| HLX |
| NR2F2 |
| ZEB1 |
| NR4A2 |
| SHISA3 |
| TRIM39 |
| EN1 |
| HLA-DMB |
| GSC |
| C2orf70 |
| HOXD3 |
| WNT5A |
| EPHA1 |
| PRDM6 |
| TBX1 |
| LOC100132215 |
| OTX1 |
| RAPGEF4 |
| XAF1 |
| PTK6 |
| HTR2A |
| ACP5 |
| DGKZ |
| KIF15 |
| KIAA1143 |
| LOC401463 |
| BHLHE22 |
| HCG27 |
| APBB2 |
| BCL2 |
| FLJ32063 |
| PHACTR3 |
| UPF2 |
| PAX3 |
| ARAP1 |
| KCNAB2 |
| LRRC56 |
| PHF11 |
| TNFAIP8 |
| TMEM154 |
| SPTBN1 |
| HMGA1 |
| DCTPP1 |
| B3GALT4 |
| FAM110A |
| FGFR2 |
| TAP2 |
| HOPX |
| SLC25A33 |
| TNFAIP8L1 |
| C6orf27 |
| HOXA9 |
| BCAT1 |
| SOX2OT |
| ARFRP1 |
| ZGPAT |
| RASA3 |
| CMTM2 |
| CMTM1 |
| C20orf85 |
| MEST |
| C6orf227 |
| GSDMD |
| SIM2 |
| GJB6 |
| CDKL2 |
| BGLAP |
| RECQL5 |
| LOC100130933 |
| VEPH1 |
| FASTKD5 |
| UBOX5 |
| VWCE |
| RAPGEFL1 |
| C10orf58 |
| ASAM |
| AGPAT1 |
| RNF5 |
| MIR330 |
| EML2 |
| LOC100130987 |
| CLCF1 |
| CHRNB1 |
| LAD1 |
| RASSF1 |
| LAT |
| SPNS1 |
| HOXD4 |
| MIR10B |
| SPINT2 |
| POLR3G |
| MBLAC2 |
| PAX6 |
| NANP |
| LOC283731 |
| ISLR2 |
| LMBRD2 |
| SKP2 |
| MPZL3 |
| CD74 |
| HSPE1 |
| HSPD1 |
| SYN3 |
| PAX9 |
| RGMA |
| KLHDC9 |
| SEMA3B |
| RGS22 |
| GDF7 |
| REPIN1 |
| ICA1L |
| SIM1 |
| ITGA6 |
| BRP44L |
| ZNF416 |
| WDFY4 |
| TOMM20L |
| BAI1 |
| KPNA2 |
| ZNF346 |
| ECHDC3 |
| DUSP1 |
| HMGCR |
| AK3L1 |
| LOC440925 |
| SYNGAP1 |
| HDAC1 |
| TMEM168 |
| CITED4 |
| MRP63 |
| SKA3 |
| CHAD |
| ACSF2 |
| CSRP1 |
| LRRC61 |
| C22orf24 |
| ZMYND8 |
| RARG |
| DLX2 |
| NDUFA6 |
| ZAR1 |
| RUNX3 |
| CMTM7 |
| ARPC1B |
| UNC84B |
| CTTN |
| FRMD4A |
| SORBS2 |
| ACSL1 |
| EBPL |
| E2F8 |
| HELZ |
| HLA-DRA |
| TBR1 |
| RPN2 |
| C20orf132 |
| ASH2L |
| AFF1 |
| SNORD59A |
| ATP5B |
| PLEKHH2 |
| LYN |
| C12orf34 |
| ANKRD32 |
| C5orf36 |
| HGS |
| ARL16 |
| FBXO5 |
| CASP8 |
| FAM59A |
| TNFSF13B |
| TXNRD1 |
| ARPC5L |
| NAP1L4 |
| RTN2 |
| C19orf40 |
| CCDC123 |
| NAA20 |
| MGAT2 |
| RPL36AL |
| CDKN1C |
| ZNF200 |
| TTC12 |
| CYFIP1 |
| EPB41 |
| C6orf150 |
| DIP2C |
| RAD17 |
| TAF9 |
| MX1 |
| FAR1 |
| KCNQ1DN |
| C18orf10 |
| KIAA1328 |
| IL6R |
| CD320 |
| HNRNPH1 |
| CCDC18 |
| TMED5 |
| LTF |
| ABLIM1 |
| LOC92659 |
| KCTD21 |
| AP3M2 |
| INPP5D |
| SNORD27 |
| STAT4 |
| PTPN6 |
| POFUT1 |
| PLAGL2 |
| CPLX1 |
| LRRC27 |
| HLA-DPA1 |
| THUMPD3 |
| MIF |
| UBXN11 |
| ZBTB25 |
| ZBTB1 |
| CHEK2 |
| HSCB |
| CTSA |
| NEURL2 |
| CAMK2D |
| MAPK1 |
| TMEM146 |
| MTHFD2 |
| ATIC |
| HIST1H2BG |
| HIST1H2AE |
| EPHX3 |
| KLHL14 |
| EIF4G3 |
| CN5H6.4 |
| GTSE1 |
| XKR6 |
| SFXN1 |
| RASSF6 |
| NDUFA4L2 |
| ALG12 |
| APBB1IP |
| NUFIP2 |
| VDAC2 |
| YWHAZ |
| UBFD1 |
| EARS2 |
| LAMC2 |
| MRS2 |
| RPS6KA5 |
| FAM63B |
| PNRC2 |
| BAZ1B |
| ERBB2IP |
| ADORA2B |
| ATP6V0E2 |
| LOC401431 |
| SNHG3-RCC1 |
| SNHG3 |
| C1orf74 |
| ATP13A1 |
| RASSF5 |
| COL4A1 |
| C1orf87 |
| SERPINB5 |
| FOXJ2 |
| FLJ35776 |
| DLGAP1 |
| HSPH1 |
| ZFYVE16 |
| ANKH |
| FLCN |
| CCDC63 |
| SDCBP |
| PLEKHJ1 |
| HK1 |
| TAF12 |
| PEX10 |
| GRK4 |
| THAP5 |
| EBF3 |
| TNFRSF10B |
| DDX43 |
| MTMR7 |
| FAM105A |
| HSD11B2 |
| HLA-F |
| LOC100133991 |
| C17orf46 |
| LPL |
| SREBF1 |
| STXBP2 |
| TRIM36 |
| SUB1 |
| C4orf34 |
| CUL2 |
| NODAL |
| PEBP1 |
| CDC73 |
| C14orf50 |
| LOC388965 |
| TMEM93 |
| C14orf80 |
| CALM3 |
| KIF21B |
| SNX10 |
| UBAC2 |
| ZNHIT1 |
| PLOD3 |
| ZNF141 |
| GPC5 |
| SDAD1 |
| SKIL |
| SMARCC1 |
| MYC |
| LGALS3BP |
| TTC13 |
| ARV1 |
| AGAP1 |
| TRIM59 |
| RCE1 |
| TMEM41B |
| MYBL1 |
| UBE2V2 |
| TGIF2 |
| DDHD2 |
| ARVCF |
| NUSAP1 |
| KLF10 |
| IDI1 |
| VAPA |
| ESRP2 |
| KLF6 |
| NPM1 |
| FDFT1 |
| FLOT2 |
| YTHDF1 |
| RPTOR |
| PTTG1IP |
| DHODH |
| RBM5 |
| PTPLAD1 |
| KDELC2 |
| NADSYN1 |
| PIK3R3 |
| C7orf55 |
| FOXK2 |
| TMEM57 |
| CCDC84 |
| FYN |
