# Supplementary material for: Exploration of Biomarkers of Psoriasis through Combined Multiomics Analysis
Source: Mediators Inflamm. 2022 Sep 23;2022:7731082. doi: 10.1155/2022/7731082 (PMC9525798; doi:10.1155/2022/7731082)
Supplement: Supplementary Materials — Supplementary Figure 1 The PCA of gene expression in psoriasis lesions and healthy controls in GSE13355 database. Supplementary Figure 2 The PCA and methylation distribution density in psoriasis lesions and healthy controls from the GSE73894 dataset. (A) PCA in GSE73894. (B) Methylation distribution density in GSE73894. Supplementary Table 1 Identification of DEGs in the psoriatic lesions and healthy control group in GSE13355. Supplementary Table 2 GO analysis on 767 DEGs in GSE13355. Supplementary Table 3 KEGG analysis on 767 DEGs in GSE13355. Supplementary Table 4 Identification of hyper-MR-genes. Supplementary Table 5 Identification of hypo-MR-genes. Supplementary Table 6 GO analysis of hyper-MR-genes. Supplementary Table 7 GO analysis of hypo-MR-genes. Supplementary Table 8 KEGG analysis of hyper-MR-genes. Supplementary Table 9 KEGG analysis of hypo-MR-genes. Supplementary Table 10 GO analysis through single-gene GSEA of GJB2. Supplementary Table 11 KEGG analysis through single-gene GSEA of GJB2. [file 7731082.f1.zip › Supplementary Table 6 (1).docx]

| GO analysis of hyper-MR-genes | | | | | | | | | | |
| --- | --- | --- | --- | --- | --- | --- | --- | --- | --- | --- |
| ONTOLOGY | ID | Description | GeneRatio | BgRatio | pvalue | p.adjust | qvalue | geneID | Count |  |
| GO:0009952 | BP | GO:0009952 | anterior/posterior pattern specification | 16/384 | 203/18862 | 4.34E-06 | 0.00882948 | 0.008358972 | HOXA5/HOXA6/HOXA4/HOXA3/HOXB3/HOXB1/HOXB2/HOXC4/LFNG/FOXA2/KDM2B/NEUROG1/RING1/WNT2/SFRP1/TDRD5 | 16 |
| GO:0048706 | BP | GO:0048706 | embryonic skeletal system development | 12/384 | 117/18862 | 4.79E-06 | 0.00882948 | 0.008358972 | HOXA5/HOXA6/HOXA4/HOXA3/HOXB3/HOXB1/HOXB2/HOXC4/MDFI/COL1A1/HOXD1/TBX15 | 12 |
| GO:0048704 | BP | GO:0048704 | embryonic skeletal system morphogenesis | 10/384 | 90/18862 | 1.46E-05 | 0.017978498 | 0.017020455 | HOXA5/HOXA6/HOXA4/HOXA3/HOXB3/HOXB1/HOXB2/HOXC4/MDFI/TBX15 | 10 |
| GO:0048705 | BP | GO:0048705 | skeletal system morphogenesis | 15/384 | 213/18862 | 3.28E-05 | 0.030238586 | 0.028627225 | HOXA5/HOXA6/HOXA4/HOXA3/HOXB3/DLX5/HOXB1/HOXB2/HOXC4/MDFI/NEUROG1/SMPD3/COL1A1/SFRP1/TBX15 | 15 |
| GO:0003002 | BP | GO:0003002 | regionalization | 19/384 | 326/18862 | 4.30E-05 | 0.030621811 | 0.028990029 | HOXA5/HOXA6/HOXA4/HOXA3/HOXB3/HOXB1/HOXB2/HOXC4/LFNG/MDFI/FOXA2/KDM2B/NEUROG1/FOXG1/RING1/WNT2/SFRP1/TDRD5/DBX1 | 19 |
| GO:0030157 | BP | GO:0030157 | pancreatic juice secretion | 4/384 | 11/18862 | 4.98E-05 | 0.030621811 | 0.028990029 | UCN/AQP1/WNK4/NPR3 | 4 |
| GO:0007589 | BP | GO:0007589 | body fluid secretion | 9/384 | 86/18862 | 6.32E-05 | 0.033274791 | 0.031501636 | UCN/AQP1/CCND1/PAM/DDR1/NEUROG1/PRLR/WNK4/NPR3 | 9 |
| GO:0042613 | CC | GO:0042613 | MHC class II protein complex | 5/394 | 16/19520 | 1.19E-05 | 0.004714873 | 0.004225446 | HLA-DPB1/HLA-DQB2/HLA-DOA/HLA-DRB1/HLA-DRA | 5 |
| GO:0043296 | CC | GO:0043296 | apical junction complex | 11/394 | 137/19520 | 0.000107478 | 0.013021305 | 0.01166963 | TBCD/PRKCZ/CLDN6/CCND1/MAGI2/AMOTL1/CDH1/CLDN11/CLDN9/CLDN5/WNK4 | 11 |
| GO:0042611 | CC | GO:0042611 | MHC protein complex | 5/394 | 25/19520 | 0.000124369 | 0.013021305 | 0.01166963 | HLA-DPB1/HLA-DQB2/HLA-DOA/HLA-DRB1/HLA-DRA | 5 |
| GO:0005923 | CC | GO:0005923 | bicellular tight junction | 10/394 | 117/19520 | 0.000131197 | 0.013021305 | 0.01166963 | TBCD/PRKCZ/CLDN6/CCND1/MAGI2/AMOTL1/CLDN11/CLDN9/CLDN5/WNK4 | 10 |
| GO:0070160 | CC | GO:0070160 | tight junction | 10/394 | 124/19520 | 0.000211907 | 0.014753847 | 0.013222326 | TBCD/PRKCZ/CLDN6/CCND1/MAGI2/AMOTL1/CLDN11/CLDN9/CLDN5/WNK4 | 10 |
| GO:0071556 | CC | GO:0071556 | integral component of lumenal side of endoplasmic reticulum membrane | 5/394 | 29/19520 | 0.000260143 | 0.014753847 | 0.013222326 | TAPBP/HLA-DPB1/HLA-DQB2/HLA-DRB1/HLA-DRA | 5 |
| GO:0098553 | CC | GO:0098553 | lumenal side of endoplasmic reticulum membrane | 5/394 | 29/19520 | 0.000260143 | 0.014753847 | 0.013222326 | TAPBP/HLA-DPB1/HLA-DQB2/HLA-DRB1/HLA-DRA | 5 |
| GO:0098576 | CC | GO:0098576 | lumenal side of membrane | 5/394 | 36/19520 | 0.000735547 | 0.036501532 | 0.032712496 | TAPBP/HLA-DPB1/HLA-DQB2/HLA-DRB1/HLA-DRA | 5 |
| GO:0030658 | CC | GO:0030658 | transport vesicle membrane | 12/394 | 206/19520 | 0.001018414 | 0.044923385 | 0.04026012 | PRRT1/HLA-DPB1/SYN3/CD59/SEC31B/HLA-DQB2/HLA-DRB1/PAM/ECE2/HLA-DRA/SCGN/SYT9 | 12 |
| GO:0032395 | MF | GO:0032395 | MHC class II receptor activity | 4/390 | 10/18337 | 3.82E-05 | 0.024735417 | 0.023381237 | HLA-DQB2/HLA-DOA/HLA-DRB1/HLA-DRA | 4 |
