# Supplementary material for: Exploration of Biomarkers of Psoriasis through Combined Multiomics Analysis
Source: Mediators Inflamm. 2022 Sep 23;2022:7731082. doi: 10.1155/2022/7731082 (PMC9525798; doi:10.1155/2022/7731082)
Supplement: Supplementary Materials — Supplementary Figure 1 The PCA of gene expression in psoriasis lesions and healthy controls in GSE13355 database. Supplementary Figure 2 The PCA and methylation distribution density in psoriasis lesions and healthy controls from the GSE73894 dataset. (A) PCA in GSE73894. (B) Methylation distribution density in GSE73894. Supplementary Table 1 Identification of DEGs in the psoriatic lesions and healthy control group in GSE13355. Supplementary Table 2 GO analysis on 767 DEGs in GSE13355. Supplementary Table 3 KEGG analysis on 767 DEGs in GSE13355. Supplementary Table 4 Identification of hyper-MR-genes. Supplementary Table 5 Identification of hypo-MR-genes. Supplementary Table 6 GO analysis of hyper-MR-genes. Supplementary Table 7 GO analysis of hypo-MR-genes. Supplementary Table 8 KEGG analysis of hyper-MR-genes. Supplementary Table 9 KEGG analysis of hypo-MR-genes. Supplementary Table 10 GO analysis through single-gene GSEA of GJB2. Supplementary Table 11 KEGG analysis through single-gene GSEA of GJB2. [file 7731082.f1.zip › Supplementary Table 7 (1).docx]

| GO analysis of hypo-MR-genes | | | | | | | | | | |
| --- | --- | --- | --- | --- | --- | --- | --- | --- | --- | --- |
| ONTOLOGY | ID | Description | GeneRatio | BgRatio | pvalue | p.adjust | qvalue | geneID | Count |  |
| GO:0048568 | BP | GO:0048568 | embryonic organ development | 31/344 | 412/18862 | 2.73E-11 | 1.13E-07 | 9.25E-08 | GATA3/HOXA7/TNF/ALX4/MSX1/HPN/ADM/TWIST1/PITX2/GRHL3/FOXF1/HLX/NR2F2/EN1/GSC/HOXD3/WNT5A/TBX1/OTX1/FGFR2/HOXA9/GJB6/HOXD4/SPINT2/RARG/DLX2/E2F8/TBR1/CASP8/MAPK1/NODAL | 31 |
| GO:0048562 | BP | GO:0048562 | embryonic organ morphogenesis | 23/344 | 277/18862 | 1.65E-09 | 3.42E-06 | 2.80E-06 | GATA3/HOXA7/ALX4/MSX1/HPN/TWIST1/GRHL3/FOXF1/HLX/GSC/HOXD3/WNT5A/TBX1/OTX1/FGFR2/HOXA9/GJB6/HOXD4/RARG/DLX2/TBR1/MAPK1/NODAL | 23 |
| GO:2001233 | BP | GO:2001233 | regulation of apoptotic signaling pathway | 24/344 | 348/18862 | 2.78E-08 | 3.85E-05 | 3.14E-05 | TNF/TNFRSF10A/S100A9/MSX1/RIPK3/TMC8/EYA4/ZNF385A/NR4A2/TRIM39/BCL2/CD74/ITGA6/HDAC1/CTTN/CASP8/TAF9/PLAGL2/MIF/VDAC2/TNFRSF10B/SKIL/PTTG1IP/FYN | 24 |
| GO:0048732 | BP | GO:0048732 | gland development | 26/344 | 413/18862 | 4.61E-08 | 4.78E-05 | 3.91E-05 | GATA3/TNF/CPT1A/MSX1/RIPK3/HPN/PITX2/FOXF1/HLX/HOXD3/WNT5A/TBX1/BCL2/UPF2/FGFR2/HOXA9/GDF7/RARG/E2F8/ZBTB1/MAPK1/SERPINB5/NODAL/SMARCC1/ESRP2/DHODH | 26 |
| GO:0045785 | BP | GO:0045785 | positive regulation of cell adhesion | 26/344 | 425/18862 | 8.15E-08 | 6.76E-05 | 5.52E-05 | GATA3/FLOT1/TNF/HLA-E/TNFRSF18/FOXF1/HLX/HLA-DMB/WNT5A/EPHA1/CD74/HSPD1/ITGA6/RUNX3/HLA-DRA/LYN/TNFSF13B/PTPN6/HLA-DPA1/ZBTB1/APBB1IP/HSPH1/FLCN/NODAL/FLOT2/FYN | 26 |
| GO:0022409 | BP | GO:0022409 | positive regulation of cell-cell adhesion | 20/344 | 276/18862 | 1.85E-07 | 0.000127941 | 0.00010453 | GATA3/FLOT1/TNF/HLA-E/HLX/HLA-DMB/WNT5A/CD74/HSPD1/RUNX3/HLA-DRA/LYN/TNFSF13B/PTPN6/HLA-DPA1/ZBTB1/HSPH1/NODAL/FLOT2/FYN | 20 |
| GO:1902253 | BP | GO:1902253 | regulation of intrinsic apoptotic signaling pathway by p53 class mediator | 7/344 | 28/18862 | 5.38E-07 | 0.000261441 | 0.000213603 | MSX1/ZNF385A/BCL2/CD74/TAF9/MIF/PTTG1IP | 7 |
| GO:0090596 | BP | GO:0090596 | sensory organ morphogenesis | 18/344 | 244/18862 | 5.90E-07 | 0.000261441 | 0.000213603 | GATA3/THRB/MSX1/HPN/TWIST1/PITX2/GRHL3/GSC/WNT5A/TBX1/OTX1/BCL2/FGFR2/GJB6/PAX6/HDAC1/RARG/MAPK1 | 18 |
| GO:0002699 | BP | GO:0002699 | positive regulation of immune effector process | 17/344 | 219/18862 | 5.98E-07 | 0.000261441 | 0.000213603 | GATA3/LTA/TNF/HLA-E/FOXF1/HLX/HLA-DMB/WNT5A/CLCF1/CD74/HSPD1/HLA-DRA/LYN/ZBTB1/HK1/HLA-F/STXBP2 | 17 |
| GO:0051251 | BP | GO:0051251 | positive regulation of lymphocyte activation | 22/344 | 356/18862 | 7.01E-07 | 0.000261441 | 0.000213603 | GATA3/HLA-E/HLX/HLA-DMB/BCL2/CLCF1/CD74/HSPD1/RUNX3/HLA-DRA/LYN/TNFSF13B/CD320/INPP5D/PTPN6/HLA-DPA1/MIF/ZBTB1/HSPH1/HLA-F/FLOT2/FYN | 22 |
| GO:0048704 | BP | GO:0048704 | embryonic skeletal system morphogenesis | 11/344 | 90/18862 | 7.29E-07 | 0.000261441 | 0.000213603 | HOXA7/ALX4/TWIST1/GSC/HOXD3/TBX1/FGFR2/HOXA9/HOXD4/DLX2/NODAL | 11 |
| GO:0042471 | BP | GO:0042471 | ear morphogenesis | 12/344 | 110/18862 | 7.93E-07 | 0.000261441 | 0.000213603 | GATA3/MSX1/HPN/TWIST1/GRHL3/GSC/WNT5A/TBX1/OTX1/FGFR2/GJB6/MAPK1 | 12 |
| GO:2001234 | BP | GO:2001234 | negative regulation of apoptotic signaling pathway | 17/344 | 224/18862 | 8.20E-07 | 0.000261441 | 0.000213603 | TNF/TNFRSF10A/EYA4/ZNF385A/NR4A2/BCL2/CD74/ITGA6/HDAC1/CTTN/CASP8/TAF9/MIF/VDAC2/TNFRSF10B/PTTG1IP/FYN | 17 |
| GO:1901797 | BP | GO:1901797 | negative regulation of signal transduction by p53 class mediator | 7/344 | 31/18862 | 1.14E-06 | 0.000337358 | 0.000275628 | TWIST1/ZNF385A/BCL2/CD74/TAF9/MIF/PTTG1IP | 7 |
| GO:0002696 | BP | GO:0002696 | positive regulation of leukocyte activation | 23/344 | 401/18862 | 1.42E-06 | 0.00035522 | 0.000290222 | GATA3/HLA-E/HLX/HLA-DMB/WNT5A/BCL2/CLCF1/CD74/HSPD1/RUNX3/HLA-DRA/LYN/TNFSF13B/CD320/INPP5D/PTPN6/HLA-DPA1/MIF/ZBTB1/HSPH1/HLA-F/FLOT2/FYN | 23 |
| GO:1903039 | BP | GO:1903039 | positive regulation of leukocyte cell-cell adhesion | 17/344 | 234/18862 | 1.50E-06 | 0.00035522 | 0.000290222 | GATA3/TNF/HLA-E/HLX/HLA-DMB/CD74/HSPD1/RUNX3/HLA-DRA/LYN/TNFSF13B/PTPN6/HLA-DPA1/ZBTB1/HSPH1/FLOT2/FYN | 17 |
| GO:1902254 | BP | GO:1902254 | negative regulation of intrinsic apoptotic signaling pathway by p53 class mediator | 6/344 | 21/18862 | 1.52E-06 | 0.00035522 | 0.000290222 | ZNF385A/BCL2/CD74/TAF9/MIF/PTTG1IP | 6 |
| GO:0048706 | BP | GO:0048706 | embryonic skeletal system development | 12/344 | 117/18862 | 1.54E-06 | 0.00035522 | 0.000290222 | HOXA7/ALX4/TWIST1/GSC/HOXD3/WNT5A/TBX1/FGFR2/HOXA9/HOXD4/DLX2/NODAL | 12 |
| GO:0050870 | BP | GO:0050870 | positive regulation of T cell activation | 16/344 | 212/18862 | 1.86E-06 | 0.000405811 | 0.000331556 | GATA3/HLA-E/HLX/HLA-DMB/CD74/HSPD1/RUNX3/HLA-DRA/LYN/TNFSF13B/PTPN6/HLA-DPA1/ZBTB1/HSPH1/FLOT2/FYN | 16 |
| GO:0050867 | BP | GO:0050867 | positive regulation of cell activation | 23/344 | 412/18862 | 2.24E-06 | 0.000436803 | 0.000356877 | GATA3/HLA-E/HLX/HLA-DMB/WNT5A/BCL2/CLCF1/CD74/HSPD1/RUNX3/HLA-DRA/LYN/TNFSF13B/CD320/INPP5D/PTPN6/HLA-DPA1/MIF/ZBTB1/HSPH1/HLA-F/FLOT2/FYN | 23 |
| GO:0042110 | BP | GO:0042110 | T cell activation | 25/344 | 474/18862 | 2.24E-06 | 0.000436803 | 0.000356877 | GATA3/TCF7/HLA-E/RIPK3/TNFRSF18/HLX/HLA-DMB/BCL2/LAT/CD74/HSPD1/WDFY4/RUNX3/HLA-DRA/LYN/CASP8/TNFSF13B/IL6R/PTPN6/HLA-DPA1/ZBTB1/APBB1IP/HSPH1/FLOT2/FYN | 25 |
| GO:2001021 | BP | GO:2001021 | negative regulation of response to DNA damage stimulus | 10/344 | 82/18862 | 2.39E-06 | 0.000436803 | 0.000356877 | TWIST1/ZNF385A/BCL2/RECQL5/CD74/FBXO5/TAF9/MIF/CHEK2/PTTG1IP | 10 |
| GO:0003002 | BP | GO:0003002 | regionalization | 20/344 | 326/18862 | 2.53E-06 | 0.000436803 | 0.000356877 | HOXA7/ALX4/MSX1/PITX2/FOXF1/NR2F2/EN1/GSC/HOXD3/WNT5A/TBX1/OTX1/FGFR2/HOXA9/HOXD4/RARG/DLX2/TBR1/POFUT1/NODAL | 20 |
| GO:1903706 | BP | GO:1903706 | regulation of hemopoiesis | 23/344 | 415/18862 | 2.53E-06 | 0.000436803 | 0.000356877 | GATA3/HOXA7/TCF7/TNF/TNFRSF18/HLX/HOXA9/BGLAP/CD74/HDAC1/RARG/RUNX3/HLA-DRA/ASH2L/LYN/CASP8/LTF/INPP5D/PTPN6/ZBTB1/CDC73/MYC/KLF10 | 23 |
| GO:0050863 | BP | GO:0050863 | regulation of T cell activation | 20/344 | 327/18862 | 2.65E-06 | 0.000439285 | 0.000358905 | GATA3/TCF7/HLA-E/RIPK3/TNFRSF18/HLX/HLA-DMB/LAT/CD74/HSPD1/RUNX3/HLA-DRA/LYN/TNFSF13B/PTPN6/HLA-DPA1/ZBTB1/HSPH1/FLOT2/FYN | 20 |
| GO:0002819 | BP | GO:0002819 | regulation of adaptive immune response | 14/344 | 170/18862 | 2.97E-06 | 0.00047373 | 0.000387048 | GATA3/TRIM27/LTA/TNF/HLA-E/RIPK3/HLX/CLCF1/HSPD1/HLA-DRA/TNFSF13B/PTPN6/ZBTB1/HLA-F | 14 |
| GO:2001020 | BP | GO:2001020 | regulation of response to DNA damage stimulus | 16/344 | 221/18862 | 3.20E-06 | 0.000490729 | 0.000400936 | IER3/MSX1/EYA4/TWIST1/ZNF385A/BCL2/RECQL5/CD74/FBXO5/TAF9/MIF/CHEK2/SKIL/MYC/UBE2V2/PTTG1IP | 16 |
| GO:0007389 | BP | GO:0007389 | pattern specification process | 23/344 | 426/18862 | 3.91E-06 | 0.00055462 | 0.000453136 | HOXA7/ALX4/MSX1/PITX2/GRHL3/FOXF1/NR2F2/EN1/GSC/HOXD3/WNT5A/TBX1/OTX1/FGFR2/HOXA9/SIM2/HOXD4/SYNGAP1/RARG/DLX2/TBR1/POFUT1/NODAL | 23 |
| GO:0052548 | BP | GO:0052548 | regulation of endopeptidase activity | 23/344 | 426/18862 | 3.91E-06 | 0.00055462 | 0.000453136 | PSMB8/TNF/PSMB9/TNFRSF10A/S100A9/LXN/DIABLO/SOX7/ADORA2A/TNFAIP8/SPINT2/HSPE1/HSPD1/HDAC1/LYN/CASP8/LTF/SERPINB5/TNFRSF10B/NODAL/PEBP1/MYC/FYN | 23 |
| GO:1902105 | BP | GO:1902105 | regulation of leukocyte differentiation | 18/344 | 279/18862 | 4.01E-06 | 0.00055462 | 0.000453136 | GATA3/HOXA7/TCF7/TNF/TNFRSF18/HLX/BGLAP/CD74/RUNX3/HLA-DRA/LYN/CASP8/LTF/INPP5D/PTPN6/ZBTB1/MYC/KLF10 | 18 |
| GO:0097193 | BP | GO:0097193 | intrinsic apoptotic signaling pathway | 18/344 | 283/18862 | 4.89E-06 | 0.000636263 | 0.00051984 | TNF/S100A9/MSX1/RIPK3/DIABLO/ZNF385A/BCL2/CD74/HDAC1/TAF9/PLAGL2/MIF/CHEK2/VDAC2/TNFRSF10B/CUL2/SKIL/PTTG1IP | 18 |
| GO:0009952 | BP | GO:0009952 | anterior/posterior pattern specification | 15/344 | 203/18862 | 5.06E-06 | 0.000636263 | 0.00051984 | HOXA7/ALX4/MSX1/FOXF1/NR2F2/EN1/HOXD3/WNT5A/TBX1/OTX1/HOXA9/HOXD4/RARG/POFUT1/NODAL | 15 |
| GO:1902166 | BP | GO:1902166 | negative regulation of intrinsic apoptotic signaling pathway in response to DNA damage by p53 class mediator | 5/344 | 15/18862 | 5.06E-06 | 0.000636263 | 0.00051984 | ZNF385A/BCL2/CD74/TAF9/MIF | 5 |
| GO:2000116 | BP | GO:2000116 | regulation of cysteine-type endopeptidase activity | 16/344 | 230/18862 | 5.34E-06 | 0.000650111 | 0.000531154 | TNF/PSMB9/TNFRSF10A/S100A9/DIABLO/SOX7/ADORA2A/TNFAIP8/HSPE1/HSPD1/CASP8/LTF/TNFRSF10B/NODAL/MYC/FYN | 16 |
| GO:0002822 | BP | GO:0002822 | regulation of adaptive immune response based on somatic recombination of immune receptors built from immunoglobulin superfamily domains | 13/344 | 155/18862 | 5.49E-06 | 0.000650111 | 0.000531154 | GATA3/LTA/TNF/HLA-E/RIPK3/HLX/CLCF1/HSPD1/HLA-DRA/TNFSF13B/PTPN6/ZBTB1/HLA-F | 13 |
| GO:0019883 | BP | GO:0019883 | antigen processing and presentation of endogenous antigen | 6/344 | 26/18862 | 5.96E-06 | 0.000686271 | 0.000560697 | TAP1/HLA-E/TAP2/CD74/HLA-DRA/HLA-F | 6 |
| GO:2001242 | BP | GO:2001242 | regulation of intrinsic apoptotic signaling pathway | 13/344 | 160/18862 | 7.77E-06 | 0.000870714 | 0.000711392 | S100A9/MSX1/RIPK3/ZNF385A/BCL2/CD74/HDAC1/TAF9/PLAGL2/MIF/VDAC2/SKIL/PTTG1IP | 13 |
| GO:0048705 | BP | GO:0048705 | skeletal system morphogenesis | 15/344 | 213/18862 | 9.06E-06 | 0.000988595 | 0.000807703 | HOXA7/ALX4/MSX1/TWIST1/GSC/HOXD3/TBX1/FGFR2/HOXA9/HOXD4/CHAD/RARG/DLX2/LTF/NODAL | 15 |
| GO:1902165 | BP | GO:1902165 | regulation of intrinsic apoptotic signaling pathway in response to DNA damage by p53 class mediator | 5/344 | 17/18862 | 1.01E-05 | 0.001076526 | 0.000879544 | ZNF385A/BCL2/CD74/TAF9/MIF | 5 |
| GO:0001501 | BP | GO:0001501 | skeletal system development | 24/344 | 486/18862 | 1.09E-05 | 0.00112688 | 0.000920684 | HOXA7/ALX4/MSX1/TWIST1/ZNF385A/PITX2/EN1/GSC/HOXD3/WNT5A/TBX1/FGFR2/HOXA9/BGLAP/HOXD4/CHAD/RARG/DLX2/RUNX3/LTF/PTPN6/ANKH/NODAL/SNX10 | 24 |
| GO:0052547 | BP | GO:0052547 | regulation of peptidase activity | 23/344 | 455/18862 | 1.14E-05 | 0.001157832 | 0.000945973 | PSMB8/TNF/PSMB9/TNFRSF10A/S100A9/LXN/DIABLO/SOX7/ADORA2A/TNFAIP8/SPINT2/HSPE1/HSPD1/HDAC1/LYN/CASP8/LTF/SERPINB5/TNFRSF10B/NODAL/PEBP1/MYC/FYN | 23 |
| GO:0048736 | BP | GO:0048736 | appendage development | 13/344 | 167/18862 | 1.24E-05 | 0.00119158 | 0.000973546 | ALX4/MSX1/TWIST1/PITX2/NR2F2/EN1/WNT5A/FGFR2/HOXA9/ITGA6/HDAC1/RARG/ZNF141 | 13 |
| GO:0060173 | BP | GO:0060173 | limb development | 13/344 | 167/18862 | 1.24E-05 | 0.00119158 | 0.000973546 | ALX4/MSX1/TWIST1/PITX2/NR2F2/EN1/WNT5A/FGFR2/HOXA9/ITGA6/HDAC1/RARG/ZNF141 | 13 |
| GO:0002824 | BP | GO:0002824 | positive regulation of adaptive immune response based on somatic recombination of immune receptors built from immunoglobulin superfamily domains | 10/344 | 99/18862 | 1.32E-05 | 0.001245237 | 0.001017385 | GATA3/LTA/TNF/HLA-E/CLCF1/HSPD1/HLA-DRA/TNFSF13B/ZBTB1/HLA-F | 10 |
| GO:0030888 | BP | GO:0030888 | regulation of B cell proliferation | 8/344 | 61/18862 | 1.43E-05 | 0.001319202 | 0.001077815 | BCL2/CLCF1/CD74/LYN/TNFSF13B/CD320/INPP5D/MIF | 8 |
| GO:0042476 | BP | GO:0042476 | odontogenesis | 11/344 | 123/18862 | 1.58E-05 | 0.001425065 | 0.001164308 | MSX1/ADM/TWIST1/PITX2/TBX1/FGFR2/BGLAP/PAX9/HDAC1/DLX2/SNX10 | 11 |
| GO:0002483 | BP | GO:0002483 | antigen processing and presentation of endogenous peptide antigen | 5/344 | 19/18862 | 1.85E-05 | 0.001628924 | 0.001330865 | TAP1/HLA-E/TAP2/HLA-DRA/HLA-F | 5 |
| GO:0002821 | BP | GO:0002821 | positive regulation of adaptive immune response | 10/344 | 104/18862 | 2.04E-05 | 0.001764625 | 0.001441735 | GATA3/LTA/TNF/HLA-E/CLCF1/HSPD1/HLA-DRA/TNFSF13B/ZBTB1/HLA-F | 10 |
| GO:0043583 | BP | GO:0043583 | ear development | 14/344 | 202/18862 | 2.15E-05 | 0.001797859 | 0.001468888 | GATA3/GJB2/MSX1/HPN/TWIST1/GRHL3/GSC/WNT5A/TBX1/OTX1/BCL2/FGFR2/GJB6/MAPK1 | 14 |
| GO:2001056 | BP | GO:2001056 | positive regulation of cysteine-type endopeptidase activity | 12/344 | 151/18862 | 2.17E-05 | 0.001797859 | 0.001468888 | TNF/TNFRSF10A/S100A9/DIABLO/SOX7/HSPE1/HSPD1/CASP8/TNFRSF10B/NODAL/MYC/FYN | 12 |
| GO:0060425 | BP | GO:0060425 | lung morphogenesis | 7/344 | 48/18862 | 2.44E-05 | 0.001987248 | 0.001623623 | TNF/FOXF1/FGFR2/MAPK1/NODAL/PLOD3/ESRP2 | 7 |
| GO:0043516 | BP | GO:0043516 | regulation of DNA damage response, signal transduction by p53 class mediator | 6/344 | 34/18862 | 3.08E-05 | 0.002368019 | 0.001934721 | MSX1/TWIST1/ZNF385A/CD74/MIF/PTTG1IP | 6 |
| GO:0010950 | BP | GO:0010950 | positive regulation of endopeptidase activity | 13/344 | 182/18862 | 3.08E-05 | 0.002368019 | 0.001934721 | TNF/TNFRSF10A/S100A9/DIABLO/SOX7/HSPE1/HSPD1/LYN/CASP8/TNFRSF10B/NODAL/MYC/FYN | 13 |
| GO:0002053 | BP | GO:0002053 | positive regulation of mesenchymal cell proliferation | 5/344 | 21/18862 | 3.14E-05 | 0.002368019 | 0.001934721 | FOXF1/WNT5A/TBX1/FGFR2/MYC | 5 |
| GO:0002703 | BP | GO:0002703 | regulation of leukocyte mediated immunity | 14/344 | 209/18862 | 3.14E-05 | 0.002368019 | 0.001934721 | GATA3/LTA/TNF/HLA-E/RIPK3/FOXF1/CLCF1/HSPD1/HLA-DRA/LYN/PTPN6/ZBTB1/HLA-F/STXBP2 | 14 |
| GO:0044843 | BP | GO:0044843 | cell cycle G1/S phase transition | 17/344 | 298/18862 | 3.63E-05 | 0.002691175 | 0.002198746 | GFI1/CCND2/ZNF385A/EIF4E/TRIM39/BCL2/BCAT1/SKP2/E2F8/FBXO5/PTPN6/CHEK2/GTSE1/CUL2/CDC73/MYC/RPTOR | 17 |
| GO:0043280 | BP | GO:0043280 | positive regulation of cysteine-type endopeptidase activity involved in apoptotic process | 11/344 | 135/18862 | 3.78E-05 | 0.002750815 | 0.002247474 | TNF/TNFRSF10A/S100A9/DIABLO/SOX7/HSPE1/HSPD1/CASP8/TNFRSF10B/NODAL/MYC | 11 |
| GO:0060541 | BP | GO:0060541 | respiratory system development | 13/344 | 188/18862 | 4.32E-05 | 0.003090674 | 0.002525145 | TNF/THRB/EIF4E/FOXF1/WNT5A/FGFR2/SIM2/RARG/MAPK1/SREBF1/NODAL/PLOD3/ESRP2 | 13 |
| GO:0030324 | BP | GO:0030324 | lung development | 12/344 | 163/18862 | 4.62E-05 | 0.003247229 | 0.002653053 | TNF/THRB/EIF4E/FOXF1/WNT5A/FGFR2/SIM2/MAPK1/SREBF1/NODAL/PLOD3/ESRP2 | 12 |
| GO:0000082 | BP | GO:0000082 | G1/S transition of mitotic cell cycle | 16/344 | 275/18862 | 4.86E-05 | 0.00335883 | 0.002744234 | GFI1/CCND2/ZNF385A/EIF4E/BCL2/BCAT1/SKP2/E2F8/FBXO5/PTPN6/CHEK2/GTSE1/CUL2/CDC73/MYC/RPTOR | 16 |
| GO:1902229 | BP | GO:1902229 | regulation of intrinsic apoptotic signaling pathway in response to DNA damage | 6/344 | 37/18862 | 5.08E-05 | 0.00345352 | 0.002821598 | ZNF385A/BCL2/CD74/TAF9/MIF/SKIL | 6 |
| GO:0060562 | BP | GO:0060562 | epithelial tube morphogenesis | 17/344 | 309/18862 | 5.71E-05 | 0.003717996 | 0.00303768 | GATA3/TNF/ADM/TWIST1/GRHL3/FOXF1/WNT5A/BCL2/FGFR2/SPINT2/GDF7/RARG/TBR1/COL4A1/NODAL/MYC/ESRP2 | 17 |
| GO:0001819 | BP | GO:0001819 | positive regulation of cytokine production | 21/344 | 437/18862 | 5.74E-05 | 0.003717996 | 0.00303768 | GATA3/FLOT1/LTA/TNF/HLA-E/TWIST1/WNT5A/SPTBN1/GSDMD/AGPAT1/POLR3G/CD74/HSPD1/CASP8/IL6R/HLA-DPA1/MIF/HK1/HLA-F/LPL/NODAL | 21 |
| GO:0022407 | BP | GO:0022407 | regulation of cell-cell adhesion | 21/344 | 437/18862 | 5.74E-05 | 0.003717996 | 0.00303768 | GATA3/FLOT1/TNF/HLA-E/HLX/HLA-DMB/WNT5A/SPINT2/CD74/HSPD1/RUNX3/HLA-DRA/LYN/TNFSF13B/PTPN6/HLA-DPA1/ZBTB1/HSPH1/NODAL/FLOT2/FYN | 21 |
| GO:0030323 | BP | GO:0030323 | respiratory tube development | 12/344 | 167/18862 | 5.85E-05 | 0.003732663 | 0.003049664 | TNF/THRB/EIF4E/FOXF1/WNT5A/FGFR2/SIM2/MAPK1/SREBF1/NODAL/PLOD3/ESRP2 | 12 |
| GO:0010463 | BP | GO:0010463 | mesenchymal cell proliferation | 6/344 | 38/18862 | 5.94E-05 | 0.003732663 | 0.003049664 | MSX1/FOXF1/WNT5A/TBX1/FGFR2/MYC | 6 |
| GO:0002761 | BP | GO:0002761 | regulation of myeloid leukocyte differentiation | 10/344 | 118/18862 | 6.09E-05 | 0.003769702 | 0.003079925 | HOXA7/TNF/BGLAP/CD74/LYN/CASP8/LTF/INPP5D/MYC/KLF10 | 10 |
| GO:0042100 | BP | GO:0042100 | B cell proliferation | 9/344 | 96/18862 | 6.47E-05 | 0.003943926 | 0.003222269 | BCL2/CLCF1/CD74/HSPD1/LYN/TNFSF13B/CD320/INPP5D/MIF | 9 |
| GO:0072332 | BP | GO:0072332 | intrinsic apoptotic signaling pathway by p53 class mediator | 8/344 | 75/18862 | 6.57E-05 | 0.003950635 | 0.003227751 | MSX1/ZNF385A/BCL2/CD74/TAF9/MIF/CHEK2/PTTG1IP | 8 |
| GO:0021915 | BP | GO:0021915 | neural tube development | 11/344 | 145/18862 | 7.27E-05 | 0.004308772 | 0.003520357 | ADM/TWIST1/GRHL3/EN1/GSC/WNT5A/SPINT2/GDF7/RARG/NODAL/PLOD3 | 11 |
| GO:0030890 | BP | GO:0030890 | positive regulation of B cell proliferation | 6/344 | 40/18862 | 8.01E-05 | 0.00463414 | 0.003786189 | BCL2/CLCF1/CD74/TNFSF13B/CD320/MIF | 6 |
| GO:0060485 | BP | GO:0060485 | mesenchyme development | 16/344 | 287/18862 | 8.06E-05 | 0.00463414 | 0.003786189 | GATA3/MSX1/HPN/TWIST1/PITX2/FOXF1/GSC/WNT5A/TBX1/BCL2/FGFR2/SEMA3B/MAPK1/SDCBP/NODAL/MYC | 16 |
| GO:0010952 | BP | GO:0010952 | positive regulation of peptidase activity | 13/344 | 200/18862 | 8.16E-05 | 0.00463414 | 0.003786189 | TNF/TNFRSF10A/S100A9/DIABLO/SOX7/HSPE1/HSPD1/LYN/CASP8/TNFRSF10B/NODAL/MYC/FYN | 13 |
| GO:0045637 | BP | GO:0045637 | regulation of myeloid cell differentiation | 15/344 | 258/18862 | 8.41E-05 | 0.004709359 | 0.003847644 | HOXA7/TNF/HOXA9/BGLAP/CD74/HDAC1/RARG/ASH2L/LYN/CASP8/LTF/INPP5D/CDC73/MYC/KLF10 | 15 |
| GO:0070665 | BP | GO:0070665 | positive regulation of leukocyte proliferation | 11/344 | 148/18862 | 8.75E-05 | 0.004838501 | 0.003953156 | HLA-E/HLA-DMB/BCL2/CLCF1/CD74/LYN/TNFSF13B/CD320/HLA-DPA1/MIF/MAPK1 | 11 |
| GO:0010464 | BP | GO:0010464 | regulation of mesenchymal cell proliferation | 5/344 | 26/18862 | 9.41E-05 | 0.005070599 | 0.004142785 | FOXF1/WNT5A/TBX1/FGFR2/MYC | 5 |
| GO:1901722 | BP | GO:1901722 | regulation of cell proliferation involved in kidney development | 4/344 | 14/18862 | 9.42E-05 | 0.005070599 | 0.004142785 | GATA3/IL6R/FLCN/MYC | 4 |
| GO:0001892 | BP | GO:0001892 | embryonic placenta development | 8/344 | 79/18862 | 9.55E-05 | 0.005073726 | 0.00414534 | ADM/NR2F2/FGFR2/SPINT2/E2F8/CASP8/MAPK1/NODAL | 8 |
| GO:0072175 | BP | GO:0072175 | epithelial tube formation | 10/344 | 125/18862 | 9.91E-05 | 0.005166679 | 0.004221284 | GATA3/ADM/TWIST1/GRHL3/WNT5A/FGFR2/SPINT2/GDF7/RARG/NODAL | 10 |
| GO:0050852 | BP | GO:0050852 | T cell receptor signaling pathway | 13/344 | 204/18862 | 9.97E-05 | 0.005166679 | 0.004221284 | GATA3/PSMB8/PSMB9/LIME1/DENND1B/DGKZ/LAT/HLA-DRA/INPP5D/PTPN6/HLA-DPA1/MAPK1/FYN | 13 |
| GO:0002478 | BP | GO:0002478 | antigen processing and presentation of exogenous peptide antigen | 12/344 | 177/18862 | 0.000102369 | 0.005175873 | 0.004228796 | PSMB8/TAP1/PSMB9/HLA-E/HLA-DMA/HLA-DMB/KIF15/TAP2/CD74/HLA-DRA/HLA-DPA1/HLA-F | 12 |
| GO:1901796 | BP | GO:1901796 | regulation of signal transduction by p53 class mediator | 12/344 | 177/18862 | 0.000102369 | 0.005175873 | 0.004228796 | MSX1/TWIST1/ZNF385A/BCL2/CD74/HDAC1/RAD17/TAF9/MIF/CHEK2/TAF12/PTTG1IP | 12 |
| GO:0043281 | BP | GO:0043281 | regulation of cysteine-type endopeptidase activity involved in apoptotic process | 13/344 | 205/18862 | 0.000104728 | 0.005231341 | 0.004274115 | TNF/TNFRSF10A/S100A9/DIABLO/SOX7/ADORA2A/TNFAIP8/HSPE1/HSPD1/CASP8/TNFRSF10B/NODAL/MYC | 13 |
| GO:0019882 | BP | GO:0019882 | antigen processing and presentation | 14/344 | 234/18862 | 0.000106631 | 0.005262992 | 0.004299974 | PSMB8/TAP1/PSMB9/HLA-E/HLA-DMA/HLA-DMB/KIF15/TAP2/CD74/WDFY4/HLA-DRA/HLA-DPA1/HLA-F/YTHDF1 | 14 |
| GO:0032526 | BP | GO:0032526 | response to retinoic acid | 9/344 | 103/18862 | 0.000112007 | 0.005399773 | 0.004411727 | GJB2/WNT5A/TBX1/PTK6/FGFR2/DUSP1/RARG/LYN/SREBF1 | 9 |
| GO:2001237 | BP | GO:2001237 | negative regulation of extrinsic apoptotic signaling pathway | 9/344 | 103/18862 | 0.000112007 | 0.005399773 | 0.004411727 | TNF/TNFRSF10A/EYA4/BCL2/ITGA6/CTTN/CASP8/TNFRSF10B/FYN | 9 |
| GO:0042493 | BP | GO:0042493 | response to drug | 18/344 | 359/18862 | 0.00011408 | 0.005436489 | 0.004441725 | GATA3/LTA/CPT1A/ADORA2A/HTR2A/BCL2/BGLAP/RECQL5/ACSL1/RPN2/LYN/CHEK2/HSD11B2/LPL/SREBF1/MYC/DHODH/FYN | 18 |
| GO:0007409 | BP | GO:0007409 | axonogenesis | 21/344 | 460/18862 | 0.000118177 | 0.005567763 | 0.004548978 | GATA3/FLOT1/NR4A2/WNT5A/EPHA1/BCL2/SPTBN1/FGFR2/ISLR2/SEMA3B/GDF7/SYNGAP1/CTTN/TBR1/CYFIP1/MAPK1/LAMC2/RPS6KA5/SKIL/YTHDF1/FYN | 21 |
| GO:0002456 | BP | GO:0002456 | T cell mediated immunity | 9/344 | 104/18862 | 0.000120683 | 0.00562191 | 0.004593218 | GATA3/HLA-E/RIPK3/PRF1/DENND1B/HSPD1/HLA-DRA/ZBTB1/HLA-F | 9 |
| GO:2001236 | BP | GO:2001236 | regulation of extrinsic apoptotic signaling pathway | 11/344 | 154/18862 | 0.000124903 | 0.0057407 | 0.004690272 | TNF/TNFRSF10A/TMC8/EYA4/BCL2/ITGA6/CTTN/CASP8/TNFRSF10B/SKIL/FYN | 11 |
| GO:0043518 | BP | GO:0043518 | negative regulation of DNA damage response, signal transduction by p53 class mediator | 4/344 | 15/18862 | 0.000126574 | 0.0057407 | 0.004690272 | TWIST1/CD74/MIF/PTTG1IP | 4 |
| GO:1903037 | BP | GO:1903037 | regulation of leukocyte cell-cell adhesion | 17/344 | 330/18862 | 0.000127387 | 0.0057407 | 0.004690272 | GATA3/TNF/HLA-E/HLX/HLA-DMB/CD74/HSPD1/RUNX3/HLA-DRA/LYN/TNFSF13B/PTPN6/HLA-DPA1/ZBTB1/HSPH1/FLOT2/FYN | 17 |
| GO:0042771 | BP | GO:0042771 | intrinsic apoptotic signaling pathway in response to DNA damage by p53 class mediator | 6/344 | 44/18862 | 0.00013859 | 0.006034465 | 0.004930284 | ZNF385A/BCL2/CD74/TAF9/MIF/CHEK2 | 6 |
| GO:0048546 | BP | GO:0048546 | digestive tract morphogenesis | 6/344 | 44/18862 | 0.00013859 | 0.006034465 | 0.004930284 | FOXF1/HLX/WNT5A/BCL2/FGFR2/NODAL | 6 |
| GO:0060324 | BP | GO:0060324 | face development | 6/344 | 44/18862 | 0.00013859 | 0.006034465 | 0.004930284 | MSX1/WNT5A/TBX1/PAX9/RARG/MAPK1 | 6 |
| GO:0030330 | BP | GO:0030330 | DNA damage response, signal transduction by p53 class mediator | 9/344 | 106/18862 | 0.000139727 | 0.006034465 | 0.004930284 | MSX1/TWIST1/ZNF385A/CD74/E2F8/MIF/CHEK2/GTSE1/PTTG1IP | 9 |
| GO:0007159 | BP | GO:0007159 | leukocyte cell-cell adhesion | 18/344 | 366/18862 | 0.000145153 | 0.006144386 | 0.005020091 | GATA3/TNF/HLA-E/S100A9/HLX/HLA-DMB/CD74/HSPD1/RUNX3/HLA-DRA/LYN/TNFSF13B/PTPN6/HLA-DPA1/ZBTB1/HSPH1/FLOT2/FYN | 18 |
| GO:0070663 | BP | GO:0070663 | regulation of leukocyte proliferation | 14/344 | 241/18862 | 0.000145417 | 0.006144386 | 0.005020091 | HLA-E/RIPK3/HLA-DMB/BCL2/CLCF1/CD74/LYN/TNFSF13B/CD320/INPP5D/PTPN6/HLA-DPA1/MIF/MAPK1 | 14 |
| GO:0008625 | BP | GO:0008625 | extrinsic apoptotic signaling pathway via death domain receptors | 8/344 | 84/18862 | 0.000147418 | 0.006144386 | 0.005020091 | TNF/TNFRSF10A/TMC8/DIABLO/BCL2/CASP8/TNFRSF10B/SKIL | 8 |
| GO:0002706 | BP | GO:0002706 | regulation of lymphocyte mediated immunity | 11/344 | 157/18862 | 0.0001482 | 0.006144386 | 0.005020091 | GATA3/LTA/TNF/HLA-E/RIPK3/CLCF1/HSPD1/HLA-DRA/PTPN6/ZBTB1/HLA-F | 11 |
| GO:0002460 | BP | GO:0002460 | adaptive immune response based on somatic recombination of immune receptors built from immunoglobulin superfamily domains | 18/344 | 367/18862 | 0.00015015 | 0.006163594 | 0.005035785 | GATA3/LTA/TNF/HLA-E/RIPK3/PRF1/DENND1B/HLX/CLCF1/CD74/HSPD1/HLA-DRA/TNFSF13B/IL6R/INPP5D/PTPN6/ZBTB1/HLA-F | 18 |
| GO:0001701 | BP | GO:0001701 | in utero embryonic development | 17/344 | 335/18862 | 0.000152496 | 0.006198509 | 0.005064311 | GATA3/MGAT1/MSX1/ADM/TWIST1/FOXF1/NR2F2/FGFR2/HOPX/SPINT2/E2F8/CASP8/MAPK1/FLCN/NODAL/PLOD3/SKIL | 17 |
| GO:0019884 | BP | GO:0019884 | antigen processing and presentation of exogenous antigen | 12/344 | 185/18862 | 0.000155453 | 0.006218309 | 0.005080488 | PSMB8/TAP1/PSMB9/HLA-E/HLA-DMA/HLA-DMB/KIF15/TAP2/CD74/HLA-DRA/HLA-DPA1/HLA-F | 12 |
| GO:0007568 | BP | GO:0007568 | aging | 16/344 | 304/18862 | 0.000156892 | 0.006218309 | 0.005080488 | GJB2/ADM/TWIST1/HTR2A/BCL2/HMGA1/GJB6/BGLAP/RPN2/FBXO5/INPP5D/MIF/CHEK2/MAPK1/SREBF1/NPM1 | 16 |
| GO:0048538 | BP | GO:0048538 | thymus development | 6/344 | 45/18862 | 0.000157482 | 0.006218309 | 0.005080488 | GATA3/RIPK3/TBX1/BCL2/ZBTB1/MAPK1 | 6 |
| GO:0002708 | BP | GO:0002708 | positive regulation of lymphocyte mediated immunity | 9/344 | 108/18862 | 0.000161219 | 0.006276816 | 0.00512829 | GATA3/LTA/TNF/HLA-E/CLCF1/HSPD1/HLA-DRA/ZBTB1/HLA-F | 9 |
| GO:0035115 | BP | GO:0035115 | embryonic forelimb morphogenesis | 5/344 | 29/18862 | 0.00016235 | 0.006276816 | 0.00512829 | ALX4/MSX1/TWIST1/EN1/HOXA9 | 5 |
| GO:0001763 | BP | GO:0001763 | morphogenesis of a branching structure | 12/344 | 186/18862 | 0.000163506 | 0.006276816 | 0.00512829 | TNF/ADM/FOXF1/WNT5A/BCL2/FGFR2/SPINT2/GDF7/DLX2/COL4A1/MYC/ESRP2 | 12 |
| GO:0030901 | BP | GO:0030901 | midbrain development | 8/344 | 86/18862 | 0.000173861 | 0.006494954 | 0.005306513 | MSX1/NR4A2/EN1/WNT5A/OTX1/FGFR2/GDF7/CALM3 | 8 |
| GO:0050804 | BP | GO:0050804 | modulation of chemical synaptic transmission | 19/344 | 405/18862 | 0.000175554 | 0.006494954 | 0.005306513 | FLOT1/PRRT1/HRH1/TNF/SHANK2/EIF4E/ADORA2A/HTR2A/DGKZ/SYN3/SYNGAP1/ZMYND8/CYFIP1/CPLX1/MAPK1/DLGAP1/CALM3/YTHDF1/FYN | 19 |
| GO:0035107 | BP | GO:0035107 | appendage morphogenesis | 10/344 | 134/18862 | 0.000176371 | 0.006494954 | 0.005306513 | ALX4/MSX1/TWIST1/EN1/WNT5A/FGFR2/HOXA9/HDAC1/RARG/ZNF141 | 10 |
| GO:0035108 | BP | GO:0035108 | limb morphogenesis | 10/344 | 134/18862 | 0.000176371 | 0.006494954 | 0.005306513 | ALX4/MSX1/TWIST1/EN1/WNT5A/FGFR2/HOXA9/HDAC1/RARG/ZNF141 | 10 |
| GO:0050890 | BP | GO:0050890 | cognition | 15/344 | 276/18862 | 0.000177021 | 0.006494954 | 0.005306513 | PRRT1/HRH1/TNF/ZNF385A/SHANK2/EN1/HTR2A/SYNGAP1/HLA-DRA/TBR1/CYFIP1/MAPK1/DDHD2/YTHDF1/FYN | 15 |
| GO:0099177 | BP | GO:0099177 | regulation of trans-synaptic signaling | 19/344 | 406/18862 | 0.000181166 | 0.006588717 | 0.005383119 | FLOT1/PRRT1/HRH1/TNF/SHANK2/EIF4E/ADORA2A/HTR2A/DGKZ/SYN3/SYNGAP1/ZMYND8/CYFIP1/CPLX1/MAPK1/DLGAP1/CALM3/YTHDF1/FYN | 19 |
| GO:0097191 | BP | GO:0097191 | extrinsic apoptotic signaling pathway | 13/344 | 217/18862 | 0.000184521 | 0.006652369 | 0.005435124 | TNF/TNFRSF10A/TMC8/EYA4/DIABLO/BCL2/ITGA6/CTTN/CASP8/IL6R/TNFRSF10B/SKIL/FYN | 13 |
| GO:0006919 | BP | GO:0006919 | activation of cysteine-type endopeptidase activity involved in apoptotic process | 8/344 | 87/18862 | 0.000188476 | 0.006717149 | 0.005488051 | TNF/TNFRSF10A/S100A9/DIABLO/HSPE1/HSPD1/CASP8/TNFRSF10B | 8 |
| GO:0050678 | BP | GO:0050678 | regulation of epithelial cell proliferation | 18/344 | 374/18862 | 0.000189558 | 0.006717149 | 0.005488051 | GATA3/TNF/HPN/TWIST1/SGPP2/NR2F2/WNT5A/TBX1/FGFR2/MIR10B/RUNX3/CDKN1C/SERPINB5/NODAL/CDC73/MYC/ESRP2/RPTOR | 18 |
| GO:1902230 | BP | GO:1902230 | negative regulation of intrinsic apoptotic signaling pathway in response to DNA damage | 5/344 | 30/18862 | 0.00019193 | 0.006743577 | 0.005509643 | ZNF385A/BCL2/CD74/TAF9/MIF | 5 |
| GO:0022612 | BP | GO:0022612 | gland morphogenesis | 9/344 | 111/18862 | 0.000198576 | 0.00691847 | 0.005652534 | TNF/HPN/WNT5A/BCL2/FGFR2/GDF7/RARG/SERPINB5/ESRP2 | 9 |
| GO:0070661 | BP | GO:0070661 | leukocyte proliferation | 16/344 | 312/18862 | 0.000210733 | 0.007252186 | 0.005925188 | HLA-E/RIPK3/HLA-DMB/BCL2/CLCF1/CD74/HSPD1/LYN/TNFSF13B/CD320/INPP5D/PTPN6/HLA-DPA1/MIF/MAPK1/FYN | 16 |
| GO:1903131 | BP | GO:1903131 | mononuclear cell differentiation | 19/344 | 411/18862 | 0.000211653 | 0.007252186 | 0.005925188 | GATA3/HOXA7/TCF7/RIPK3/TNFRSF18/HLX/BCL2/CLCF1/CD74/RUNX3/CMTM7/HLA-DRA/LYN/IL6R/INPP5D/PTPN6/ZBTB1/MYC/KLF6 | 19 |
| GO:0019885 | BP | GO:0019885 | antigen processing and presentation of endogenous peptide antigen via MHC class I | 4/344 | 17/18862 | 0.000214415 | 0.007286597 | 0.005953302 | TAP1/HLA-E/TAP2/HLA-F | 4 |
| GO:0050670 | BP | GO:0050670 | regulation of lymphocyte proliferation | 13/344 | 221/18862 | 0.000220744 | 0.007411474 | 0.006055329 | HLA-E/RIPK3/HLA-DMB/BCL2/CLCF1/CD74/LYN/TNFSF13B/CD320/INPP5D/PTPN6/HLA-DPA1/MIF | 13 |
| GO:0045165 | BP | GO:0045165 | cell fate commitment | 14/344 | 251/18862 | 0.000221719 | 0.007411474 | 0.006055329 | GATA3/NR2F2/GSC/WNT5A/TBX1/BCL2/FGFR2/PAX6/GDF7/DLX2/TBR1/IL6R/NODAL/CDC73 | 14 |
| GO:0046651 | BP | GO:0046651 | lymphocyte proliferation | 15/344 | 282/18862 | 0.000223453 | 0.007411474 | 0.006055329 | HLA-E/RIPK3/HLA-DMB/BCL2/CLCF1/CD74/HSPD1/LYN/TNFSF13B/CD320/INPP5D/PTPN6/HLA-DPA1/MIF/FYN | 15 |
| GO:1904837 | BP | GO:1904837 | beta-catenin-TCF complex assembly | 5/344 | 31/18862 | 0.000225446 | 0.007418256 | 0.006060869 | TCF7/HDAC1/ASH2L/CDC73/MYC | 5 |
| GO:0032944 | BP | GO:0032944 | regulation of mononuclear cell proliferation | 13/344 | 223/18862 | 0.000241033 | 0.007805684 | 0.006377406 | HLA-E/RIPK3/HLA-DMB/BCL2/CLCF1/CD74/LYN/TNFSF13B/CD320/INPP5D/PTPN6/HLA-DPA1/MIF | 13 |
| GO:0048002 | BP | GO:0048002 | antigen processing and presentation of peptide antigen | 12/344 | 194/18862 | 0.000241774 | 0.007805684 | 0.006377406 | PSMB8/TAP1/PSMB9/HLA-E/HLA-DMA/HLA-DMB/KIF15/TAP2/CD74/HLA-DRA/HLA-DPA1/HLA-F | 12 |
| GO:0001838 | BP | GO:0001838 | embryonic epithelial tube formation | 9/344 | 114/18862 | 0.000242869 | 0.007805684 | 0.006377406 | GATA3/ADM/TWIST1/GRHL3/WNT5A/SPINT2/GDF7/RARG/NODAL | 9 |
| GO:0032943 | BP | GO:0032943 | mononuclear cell proliferation | 15/344 | 285/18862 | 0.00025038 | 0.007917069 | 0.00646841 | HLA-E/RIPK3/HLA-DMB/BCL2/CLCF1/CD74/HSPD1/LYN/TNFSF13B/CD320/INPP5D/PTPN6/HLA-DPA1/MIF/FYN | 15 |
| GO:0016331 | BP | GO:0016331 | morphogenesis of embryonic epithelium | 10/344 | 140/18862 | 0.000252063 | 0.007917069 | 0.00646841 | GATA3/ADM/TWIST1/GRHL3/WNT5A/FGFR2/SPINT2/GDF7/RARG/NODAL | 10 |
| GO:0035148 | BP | GO:0035148 | tube formation | 10/344 | 140/18862 | 0.000252063 | 0.007917069 | 0.00646841 | GATA3/ADM/TWIST1/GRHL3/WNT5A/FGFR2/SPINT2/GDF7/RARG/NODAL | 10 |
| GO:0042531 | BP | GO:0042531 | positive regulation of tyrosine phosphorylation of STAT protein | 7/344 | 69/18862 | 0.000258243 | 0.008050184 | 0.006577168 | TNF/TNFRSF18/PTK6/CLCF1/LYN/IL6R/FYN | 7 |
| GO:0030099 | BP | GO:0030099 | myeloid cell differentiation | 19/344 | 419/18862 | 0.000269819 | 0.008348268 | 0.006820709 | GATA3/HOXA7/TNF/ZNF385A/HOXA9/BGLAP/CD74/HDAC1/RARG/ASH2L/LYN/CASP8/LTF/INPP5D/PTPN6/CDC73/SNX10/MYC/KLF10 | 19 |
| GO:0002367 | BP | GO:0002367 | cytokine production involved in immune response | 8/344 | 93/18862 | 0.000298895 | 0.009179385 | 0.007499749 | GATA3/TNF/HLA-E/DENND1B/WNT5A/CD74/HK1/HLA-F | 8 |
| GO:0048762 | BP | GO:0048762 | mesenchymal cell differentiation | 13/344 | 231/18862 | 0.00033895 | 0.010333004 | 0.00844228 | GATA3/MSX1/HPN/TWIST1/PITX2/GSC/WNT5A/TBX1/BCL2/FGFR2/SEMA3B/MAPK1/SDCBP | 13 |
| GO:0061138 | BP | GO:0061138 | morphogenesis of a branching epithelium | 11/344 | 173/18862 | 0.000344592 | 0.010385969 | 0.008485553 | TNF/ADM/FOXF1/WNT5A/BCL2/FGFR2/SPINT2/GDF7/COL4A1/MYC/ESRP2 | 11 |
| GO:2001243 | BP | GO:2001243 | negative regulation of intrinsic apoptotic signaling pathway | 8/344 | 95/18862 | 0.000345698 | 0.010385969 | 0.008485553 | ZNF385A/BCL2/CD74/HDAC1/TAF9/MIF/VDAC2/PTTG1IP | 8 |
| GO:0050673 | BP | GO:0050673 | epithelial cell proliferation | 19/344 | 428/18862 | 0.000351534 | 0.010473993 | 0.00855747 | GATA3/TNF/HPN/TWIST1/SGPP2/NR2F2/WNT5A/TBX1/FGFR2/MIR10B/RUNX3/CDKN1C/MAPK1/SERPINB5/NODAL/CDC73/MYC/ESRP2/RPTOR | 19 |
| GO:0002711 | BP | GO:0002711 | positive regulation of T cell mediated immunity | 6/344 | 52/18862 | 0.00035368 | 0.010473993 | 0.00855747 | GATA3/HLA-E/HSPD1/HLA-DRA/ZBTB1/HLA-F | 6 |
| GO:0072331 | BP | GO:0072331 | signal transduction by p53 class mediator | 14/344 | 263/18862 | 0.000356716 | 0.010488959 | 0.008569698 | MSX1/TWIST1/ZNF385A/BCL2/CD74/HDAC1/E2F8/RAD17/TAF9/MIF/CHEK2/GTSE1/TAF12/PTTG1IP | 14 |
| GO:0001841 | BP | GO:0001841 | neural tube formation | 8/344 | 96/18862 | 0.000371245 | 0.010768957 | 0.008798462 | ADM/TWIST1/GRHL3/WNT5A/SPINT2/GDF7/RARG/NODAL | 8 |
| GO:0002697 | BP | GO:0002697 | regulation of immune effector process | 20/344 | 465/18862 | 0.000371433 | 0.010768957 | 0.008798462 | TSPAN32/GATA3/LTA/TNF/HLA-E/RIPK3/FOXF1/HLX/HLA-DMB/WNT5A/CLCF1/CD74/HSPD1/HLA-DRA/LYN/PTPN6/ZBTB1/HK1/HLA-F/STXBP2 | 20 |
| GO:0002573 | BP | GO:0002573 | myeloid leukocyte differentiation | 12/344 | 204/18862 | 0.000382546 | 0.011014137 | 0.008998779 | GATA3/HOXA7/TNF/BGLAP/CD74/LYN/CASP8/LTF/INPP5D/SNX10/MYC/KLF10 | 12 |
| GO:0002720 | BP | GO:0002720 | positive regulation of cytokine production involved in immune response | 6/344 | 53/18862 | 0.000392789 | 0.011231043 | 0.009175996 | GATA3/HLA-E/WNT5A/CD74/HK1/HLA-F | 6 |
| GO:0007050 | BP | GO:0007050 | cell cycle arrest | 13/344 | 235/18862 | 0.000399457 | 0.01134348 | 0.009267859 | ZNF385A/APBB2/SLC25A33/RASSF1/DUSP1/E2F8/CDKN1C/MIF/CHEK2/GTSE1/SKIL/MYC/RPTOR | 13 |
| GO:0035270 | BP | GO:0035270 | endocrine system development | 9/344 | 122/18862 | 0.000402508 | 0.011352381 | 0.009275132 | GATA3/MSX1/PITX2/HOXD3/WNT5A/TBX1/PAX6/IL6R/MAPK1 | 9 |
| GO:0035821 | BP | GO:0035821 | modulation of process of other organism | 9/344 | 123/18862 | 0.000427453 | 0.011974452 | 0.009783377 | S100A9/HPN/PRF1/GSDMD/KPNA2/HDAC1/CASP8/LTF/VAPA | 9 |
| GO:0007369 | BP | GO:0007369 | gastrulation | 11/344 | 179/18862 | 0.000460378 | 0.012777823 | 0.010439748 | SOX7/FOXF1/GSC/WNT5A/FGFR2/ARFRP1/DUSP1/TXNRD1/NODAL/CDC73/TGIF2 | 11 |
| GO:0035136 | BP | GO:0035136 | forelimb morphogenesis | 5/344 | 36/18862 | 0.000464253 | 0.012777823 | 0.010439748 | ALX4/MSX1/TWIST1/EN1/HOXA9 | 5 |
| GO:0002683 | BP | GO:0002683 | negative regulation of immune system process | 18/344 | 403/18862 | 0.000465377 | 0.012777823 | 0.010439748 | TSPAN32/TRIM27/HOXA7/TNF/HLA-E/FOXF1/HLX/DGKZ/CD74/DUSP1/RUNX3/LYN/LTF/INPP5D/PTPN6/MIF/HLA-F/MYC | 18 |
| GO:0071496 | BP | GO:0071496 | cellular response to external stimulus | 15/344 | 303/18862 | 0.000478382 | 0.012953937 | 0.010583636 | MAP1LC3B2/TNFRSF10A/NR4A2/BCL2/DCTPP1/BGLAP/LYN/CASP8/MAPK1/FLCN/TNFRSF10B/LPL/SREBF1/KLF10/RPTOR | 15 |
| GO:0002705 | BP | GO:0002705 | positive regulation of leukocyte mediated immunity | 9/344 | 125/18862 | 0.000481164 | 0.012953937 | 0.010583636 | GATA3/LTA/TNF/HLA-E/CLCF1/HSPD1/HLA-DRA/ZBTB1/HLA-F | 9 |
| GO:0048565 | BP | GO:0048565 | digestive tract development | 9/344 | 125/18862 | 0.000481164 | 0.012953937 | 0.010583636 | TNF/ALX4/FOXF1/HLX/WNT5A/PTK6/BCL2/FGFR2/NODAL | 9 |
| GO:0008630 | BP | GO:0008630 | intrinsic apoptotic signaling pathway in response to DNA damage | 8/344 | 100/18862 | 0.000489266 | 0.013087068 | 0.010692407 | TNF/ZNF385A/BCL2/CD74/TAF9/MIF/CHEK2/SKIL | 8 |
| GO:0048608 | BP | GO:0048608 | reproductive structure development | 18/344 | 405/18862 | 0.000493267 | 0.013109511 | 0.010710744 | GATA3/GJB2/ADM/NR2F2/WNT5A/BCL2/FGFR2/HOXA9/SPINT2/GDF7/RARG/E2F8/CASP8/PTPN6/MAPK1/SERPINB5/NODAL/SMARCC1 | 18 |
| GO:0002320 | BP | GO:0002320 | lymphoid progenitor cell differentiation | 4/344 | 21/18862 | 0.000509 | 0.013272403 | 0.01084383 | GATA3/BCL2/ZBTB1/FLCN | 4 |
| GO:0045655 | BP | GO:0045655 | regulation of monocyte differentiation | 4/344 | 21/18862 | 0.000509 | 0.013272403 | 0.01084383 | HOXA7/CD74/INPP5D/MYC | 4 |
| GO:0072111 | BP | GO:0072111 | cell proliferation involved in kidney development | 4/344 | 21/18862 | 0.000509 | 0.013272403 | 0.01084383 | GATA3/IL6R/FLCN/MYC | 4 |
| GO:1902106 | BP | GO:1902106 | negative regulation of leukocyte differentiation | 8/344 | 101/18862 | 0.000523077 | 0.013460192 | 0.010997258 | HOXA7/HLX/CD74/RUNX3/LYN/LTF/INPP5D/MYC | 8 |
| GO:0010883 | BP | GO:0010883 | regulation of lipid storage | 6/344 | 56/18862 | 0.000530663 | 0.013460192 | 0.010997258 | TNF/CPT1A/MEST/MIR10B/LPL/SREBF1 | 6 |
| GO:0032731 | BP | GO:0032731 | positive regulation of interleukin-1 beta production | 6/344 | 56/18862 | 0.000530663 | 0.013460192 | 0.010997258 | TNF/WNT5A/GSDMD/CASP8/HK1/LPL | 6 |
| GO:0061458 | BP | GO:0061458 | reproductive system development | 18/344 | 408/18862 | 0.000537809 | 0.013460192 | 0.010997258 | GATA3/GJB2/ADM/NR2F2/WNT5A/BCL2/FGFR2/HOXA9/SPINT2/GDF7/RARG/E2F8/CASP8/PTPN6/MAPK1/SERPINB5/NODAL/SMARCC1 | 18 |
| GO:0014065 | BP | GO:0014065 | phosphatidylinositol 3-kinase signaling | 10/344 | 154/18862 | 0.000538927 | 0.013460192 | 0.010997258 | GATA3/IER3/TNF/LIME1/TWIST1/HTR2A/LYN/PTPN6/MAPK1/FYN | 10 |
| GO:1902107 | BP | GO:1902107 | positive regulation of leukocyte differentiation | 10/344 | 154/18862 | 0.000538927 | 0.013460192 | 0.010997258 | GATA3/TNF/HLX/CD74/RUNX3/HLA-DRA/CASP8/INPP5D/ZBTB1/KLF10 | 10 |
| GO:1903708 | BP | GO:1903708 | positive regulation of hemopoiesis | 10/344 | 154/18862 | 0.000538927 | 0.013460192 | 0.010997258 | GATA3/TNF/HLX/CD74/RUNX3/HLA-DRA/CASP8/INPP5D/ZBTB1/KLF10 | 10 |
| GO:0002709 | BP | GO:0002709 | regulation of T cell mediated immunity | 7/344 | 78/18862 | 0.000549922 | 0.013652548 | 0.011154416 | GATA3/HLA-E/RIPK3/HSPD1/HLA-DRA/ZBTB1/HLA-F | 7 |
| GO:0043122 | BP | GO:0043122 | regulation of I-kappaB kinase/NF-kappaB signaling | 13/344 | 244/18862 | 0.000569894 | 0.014064161 | 0.011490713 | TRIM27/TNF/LIME1/TRIM39/WNT5A/CD74/HDAC1/CASP8/LTF/TNFRSF10B/TRIM59/VAPA/FYN | 13 |
| GO:0070997 | BP | GO:0070997 | neuron death | 16/344 | 342/18862 | 0.00058024 | 0.014234752 | 0.011630089 | GATA3/TNF/CTSZ/THRB/DIABLO/ADORA2A/NR4A2/EN1/WNT5A/BCL2/CLCF1/SYNGAP1/CASP8/HLA-F/UBE2V2/FYN | 16 |
| GO:0002260 | BP | GO:0002260 | lymphocyte homeostasis | 6/344 | 57/18862 | 0.00058412 | 0.014245658 | 0.011638999 | RIPK3/BCL2/CD74/LYN/TNFSF13B/SKIL | 6 |
| GO:0040013 | BP | GO:0040013 | negative regulation of locomotion | 17/344 | 377/18862 | 0.000597616 | 0.014489563 | 0.011838275 | GATA3/ARHGDIB/HOXA7/TNF/ADORA2A/NR2F2/WNT5A/EPHA1/BCL2/SPINT2/CD74/SEMA3B/DUSP1/ZMYND8/MIF/FLCN/NODAL | 17 |
| GO:0090103 | BP | GO:0090103 | cochlea morphogenesis | 4/344 | 22/18862 | 0.000613226 | 0.014781606 | 0.01207688 | HPN/GRHL3/WNT5A/TBX1 | 4 |
| GO:0048853 | BP | GO:0048853 | forebrain morphogenesis | 3/344 | 10/18862 | 0.000656096 | 0.015633175 | 0.01277263 | WNT5A/OTX1/GDF7 | 3 |
| GO:2000343 | BP | GO:2000343 | positive regulation of chemokine (C-X-C motif) ligand 2 production | 3/344 | 10/18862 | 0.000656096 | 0.015633175 | 0.01277263 | TNF/CD74/LPL | 3 |
| GO:0042770 | BP | GO:0042770 | signal transduction in response to DNA damage | 9/344 | 131/18862 | 0.000676485 | 0.015965797 | 0.013044389 | MSX1/TWIST1/ZNF385A/CD74/E2F8/MIF/CHEK2/GTSE1/PTTG1IP | 9 |
| GO:1903707 | BP | GO:1903707 | negative regulation of hemopoiesis | 8/344 | 105/18862 | 0.000677757 | 0.015965797 | 0.013044389 | HOXA7/HLX/CD74/RUNX3/LYN/LTF/INPP5D/MYC | 8 |
| GO:0051092 | BP | GO:0051092 | positive regulation of NF-kappaB transcription factor activity | 10/344 | 159/18862 | 0.000691511 | 0.016197769 | 0.013233915 | FLOT1/TRIM27/TNF/S100A9/RIPK3/WNT5A/LTF/RPS6KA5/NPM1/FLOT2 | 10 |
| GO:0007249 | BP | GO:0007249 | I-kappaB kinase/NF-kappaB signaling | 14/344 | 282/18862 | 0.000712327 | 0.016591605 | 0.013555687 | TRIM27/TNF/LIME1/RIPK3/TRIM39/WNT5A/CD74/HDAC1/CASP8/LTF/TNFRSF10B/TRIM59/VAPA/FYN | 14 |
| GO:0060571 | BP | GO:0060571 | morphogenesis of an epithelial fold | 4/344 | 23/18862 | 0.000731732 | 0.016854226 | 0.013770254 | WNT5A/FGFR2/GDF7/NODAL | 4 |
| GO:0060706 | BP | GO:0060706 | cell differentiation involved in embryonic placenta development | 4/344 | 23/18862 | 0.000731732 | 0.016854226 | 0.013770254 | NR2F2/SPINT2/E2F8/CASP8 | 4 |
| GO:0032722 | BP | GO:0032722 | positive regulation of chemokine production | 6/344 | 60/18862 | 0.000769857 | 0.017537517 | 0.014328517 | TNF/TWIST1/WNT5A/CD74/IL6R/LPL | 6 |
| GO:2001244 | BP | GO:2001244 | positive regulation of intrinsic apoptotic signaling pathway | 6/344 | 60/18862 | 0.000769857 | 0.017537517 | 0.014328517 | S100A9/MSX1/RIPK3/BCL2/PLAGL2/SKIL | 6 |
| GO:0055123 | BP | GO:0055123 | digestive system development | 9/344 | 134/18862 | 0.000796026 | 0.018034567 | 0.014734617 | TNF/ALX4/FOXF1/HLX/WNT5A/PTK6/BCL2/FGFR2/NODAL | 9 |
| GO:0051235 | BP | GO:0051235 | maintenance of location | 15/344 | 319/18862 | 0.00081186 | 0.018293317 | 0.014946022 | TNF/CPT1A/LIME1/S100A9/HTR2A/RASA3/MEST/MIR10B/LYN/PTPN6/CAMK2D/HK1/LPL/SREBF1/CALM3 | 15 |
| GO:0001890 | BP | GO:0001890 | placenta development | 9/344 | 135/18862 | 0.000839496 | 0.018612566 | 0.015206854 | GJB2/ADM/NR2F2/FGFR2/SPINT2/E2F8/CASP8/MAPK1/NODAL | 9 |
| GO:0050671 | BP | GO:0050671 | positive regulation of lymphocyte proliferation | 9/344 | 135/18862 | 0.000839496 | 0.018612566 | 0.015206854 | HLA-E/HLA-DMB/BCL2/CLCF1/CD74/TNFSF13B/CD320/HLA-DPA1/MIF | 9 |
| GO:0051384 | BP | GO:0051384 | response to glucocorticoid | 9/344 | 135/18862 | 0.000839496 | 0.018612566 | 0.015206854 | TNF/GJB2/ADM/EIF4E/BCL2/BGLAP/DUSP1/HSD11B2/CALM3 | 9 |
| GO:0030850 | BP | GO:0030850 | prostate gland development | 5/344 | 41/18862 | 0.000856657 | 0.018801121 | 0.015360907 | WNT5A/FGFR2/RARG/SERPINB5/SMARCC1 | 5 |
| GO:0002718 | BP | GO:0002718 | regulation of cytokine production involved in immune response | 7/344 | 84/18862 | 0.000859133 | 0.018801121 | 0.015360907 | GATA3/TNF/HLA-E/WNT5A/CD74/HK1/HLA-F | 7 |
| GO:0060348 | BP | GO:0060348 | bone development | 11/344 | 193/18862 | 0.000861605 | 0.018801121 | 0.015360907 | MSX1/TWIST1/ZNF385A/PITX2/FGFR2/BGLAP/CHAD/RARG/LTF/PTPN6/SNX10 | 11 |
| GO:0032946 | BP | GO:0032946 | positive regulation of mononuclear cell proliferation | 9/344 | 136/18862 | 0.000884877 | 0.019019736 | 0.015539521 | HLA-E/HLA-DMB/BCL2/CLCF1/CD74/TNFSF13B/CD320/HLA-DPA1/MIF | 9 |
| GO:0061029 | BP | GO:0061029 | eyelid development in camera-type eye | 3/344 | 11/18862 | 0.000889973 | 0.019019736 | 0.015539521 | TWIST1/GRHL3/HDAC1 | 3 |
| GO:0061307 | BP | GO:0061307 | cardiac neural crest cell differentiation involved in heart development | 3/344 | 11/18862 | 0.000889973 | 0.019019736 | 0.015539521 | TWIST1/PITX2/MAPK1 | 3 |
| GO:0061308 | BP | GO:0061308 | cardiac neural crest cell development involved in heart development | 3/344 | 11/18862 | 0.000889973 | 0.019019736 | 0.015539521 | TWIST1/PITX2/MAPK1 | 3 |
| GO:0050851 | BP | GO:0050851 | antigen receptor-mediated signaling pathway | 15/344 | 323/18862 | 0.000920827 | 0.019500129 | 0.015932012 | GATA3/PSMB8/PSMB9/LIME1/DENND1B/DGKZ/BCL2/LAT/HLA-DRA/LYN/INPP5D/PTPN6/HLA-DPA1/MAPK1/FYN | 15 |
| GO:0042509 | BP | GO:0042509 | regulation of tyrosine phosphorylation of STAT protein | 7/344 | 85/18862 | 0.000921858 | 0.019500129 | 0.015932012 | TNF/TNFRSF18/PTK6/CLCF1/LYN/IL6R/FYN | 7 |
| GO:0030098 | BP | GO:0030098 | lymphocyte differentiation | 16/344 | 358/18862 | 0.000943601 | 0.019858737 | 0.016225002 | GATA3/TCF7/RIPK3/TNFRSF18/HLX/BCL2/CLCF1/CD74/RUNX3/CMTM7/HLA-DRA/IL6R/INPP5D/PTPN6/ZBTB1/KLF6 | 16 |
| GO:0032755 | BP | GO:0032755 | positive regulation of interleukin-6 production | 7/344 | 86/18862 | 0.000988137 | 0.020690985 | 0.016904966 | TNF/TWIST1/WNT5A/CD74/HSPD1/IL6R/LPL | 7 |
| GO:0061180 | BP | GO:0061180 | mammary gland epithelium development | 6/344 | 63/18862 | 0.000998199 | 0.020796644 | 0.016991292 | GATA3/MSX1/FOXF1/WNT5A/FGFR2/MAPK1 | 6 |
| GO:0050954 | BP | GO:0050954 | sensory perception of mechanical stimulus | 10/344 | 167/18862 | 0.001008622 | 0.020908726 | 0.017082865 | THRB/GJB2/HPN/EYA4/TBX1/HTR2A/PAX3/GJB6/EML2/FYN | 10 |
| GO:0017157 | BP | GO:0017157 | regulation of exocytosis | 11/344 | 197/18862 | 0.001018724 | 0.021013085 | 0.017168128 | FOXF1/ADORA2A/RAPGEF4/HTR2A/LYN/HGS/CPLX1/SDCBP/HLA-F/STXBP2/CALM3 | 11 |
| GO:0030326 | BP | GO:0030326 | embryonic limb morphogenesis | 8/344 | 112/18862 | 0.001035745 | 0.021050002 | 0.017198291 | ALX4/MSX1/TWIST1/EN1/WNT5A/HOXA9/HDAC1/RARG | 8 |
| GO:0032609 | BP | GO:0032609 | interferon-gamma production | 8/344 | 112/18862 | 0.001035745 | 0.021050002 | 0.017198291 | GATA3/TRIM27/LTA/TNF/RIPK3/WNT5A/HSPD1/HLA-DPA1 | 8 |
| GO:0035113 | BP | GO:0035113 | embryonic appendage morphogenesis | 8/344 | 112/18862 | 0.001035745 | 0.021050002 | 0.017198291 | ALX4/MSX1/TWIST1/EN1/WNT5A/HOXA9/HDAC1/RARG | 8 |
| GO:0035094 | BP | GO:0035094 | response to nicotine | 5/344 | 43/18862 | 0.001068141 | 0.021602505 | 0.017649697 | TNF/MSX1/BCL2/CHRNB1/MAPK1 | 5 |
| GO:0016049 | BP | GO:0016049 | cell growth | 19/344 | 470/18862 | 0.001082761 | 0.021623684 | 0.017667001 | S100A9/MSX1/HPN/TMC8/WNT5A/APBB2/BCL2/SLC25A33/ISLR2/SEMA3B/RARG/CTTN/SORBS2/CYFIP1/CAMK2D/FLCN/SDCBP/CDC73/RPTOR | 19 |
| GO:0032732 | BP | GO:0032732 | positive regulation of interleukin-1 production | 6/344 | 64/18862 | 0.001084835 | 0.021623684 | 0.017667001 | TNF/WNT5A/GSDMD/CASP8/HK1/LPL | 6 |
| GO:0071300 | BP | GO:0071300 | cellular response to retinoic acid | 6/344 | 64/18862 | 0.001084835 | 0.021623684 | 0.017667001 | WNT5A/TBX1/PTK6/FGFR2/RARG/LYN | 6 |
| GO:0031647 | BP | GO:0031647 | regulation of protein stability | 14/344 | 295/18862 | 0.001099923 | 0.021755565 | 0.01777475 | FLOT1/MSX1/TRIM39/BCL2/RNF5/HSPD1/TAF9/CHEK2/CTSA/MAPK1/GTSE1/SREBF1/CDC73/FLOT2 | 14 |
| GO:0051402 | BP | GO:0051402 | neuron apoptotic process | 12/344 | 230/18862 | 0.001101946 | 0.021755565 | 0.01777475 | GATA3/TNF/THRB/DIABLO/ADORA2A/NR4A2/EN1/BCL2/CLCF1/SYNGAP1/UBE2V2/FYN | 12 |
| GO:0007260 | BP | GO:0007260 | tyrosine phosphorylation of STAT protein | 7/344 | 88/18862 | 0.001131911 | 0.022136344 | 0.018085854 | TNF/TNFRSF18/PTK6/CLCF1/LYN/IL6R/FYN | 7 |
| GO:0014020 | BP | GO:0014020 | primary neural tube formation | 7/344 | 88/18862 | 0.001131911 | 0.022136344 | 0.018085854 | ADM/TWIST1/GRHL3/WNT5A/SPINT2/RARG/NODAL | 7 |
| GO:0002863 | BP | GO:0002863 | positive regulation of inflammatory response to antigenic stimulus | 3/344 | 12/18862 | 0.001170653 | 0.022470028 | 0.018358481 | LTA/TNF/HLA-E | 3 |
| GO:0032352 | BP | GO:0032352 | positive regulation of hormone metabolic process | 3/344 | 12/18862 | 0.001170653 | 0.022470028 | 0.018358481 | GATA3/HPN/ADM | 3 |
| GO:0036462 | BP | GO:0036462 | TRAIL-activated apoptotic signaling pathway | 3/344 | 12/18862 | 0.001170653 | 0.022470028 | 0.018358481 | TNFRSF10A/CASP8/TNFRSF10B | 3 |
| GO:0060670 | BP | GO:0060670 | branching involved in labyrinthine layer morphogenesis | 3/344 | 12/18862 | 0.001170653 | 0.022470028 | 0.018358481 | ADM/FGFR2/SPINT2 | 3 |
| GO:0060711 | BP | GO:0060711 | labyrinthine layer development | 5/344 | 44/18862 | 0.001187314 | 0.022684819 | 0.01853397 | ADM/FGFR2/SPINT2/CASP8/MAPK1 | 5 |
| GO:0001704 | BP | GO:0001704 | formation of primary germ layer | 8/344 | 115/18862 | 0.001229308 | 0.02327265 | 0.01901424 | SOX7/FOXF1/WNT5A/FGFR2/DUSP1/TXNRD1/NODAL/CDC73 | 8 |
| GO:0007569 | BP | GO:0007569 | cell aging | 8/344 | 115/18862 | 0.001229308 | 0.02327265 | 0.01901424 | TWIST1/BCL2/HMGA1/BGLAP/FBXO5/MIF/CHEK2/NPM1 | 8 |
| GO:0048754 | BP | GO:0048754 | branching morphogenesis of an epithelial tube | 9/344 | 143/18862 | 0.001261433 | 0.023772272 | 0.019422441 | TNF/FOXF1/WNT5A/BCL2/FGFR2/GDF7/COL4A1/MYC/ESRP2 | 9 |
| GO:0009991 | BP | GO:0009991 | response to extracellular stimulus | 19/344 | 477/18862 | 0.001285251 | 0.024111537 | 0.019699629 | LTA/MAP1LC3B2/CPT1A/ADM/NR4A2/BCL2/DCTPP1/BGLAP/ACSL1/LYN/MAPK1/FLCN/HSD11B2/LPL/SREBF1/KLF10/RPTOR/DHODH/FOXK2 | 19 |
| GO:0042472 | BP | GO:0042472 | inner ear morphogenesis | 7/344 | 90/18862 | 0.001291603 | 0.024121558 | 0.019707816 | GATA3/HPN/GRHL3/WNT5A/TBX1/OTX1/FGFR2 | 7 |
| GO:0048701 | BP | GO:0048701 | embryonic cranial skeleton morphogenesis | 5/344 | 45/18862 | 0.001316043 | 0.024295979 | 0.019850322 | TWIST1/TBX1/FGFR2/DLX2/NODAL | 5 |
| GO:0090102 | BP | GO:0090102 | cochlea development | 5/344 | 45/18862 | 0.001316043 | 0.024295979 | 0.019850322 | GATA3/HPN/GRHL3/WNT5A/TBX1 | 5 |
| GO:0007611 | BP | GO:0007611 | learning or memory | 12/344 | 235/18862 | 0.001324383 | 0.024295979 | 0.019850322 | PRRT1/HRH1/ZNF385A/SHANK2/EN1/HTR2A/SYNGAP1/TBR1/MAPK1/DDHD2/YTHDF1/FYN | 12 |
| GO:0031668 | BP | GO:0031668 | cellular response to extracellular stimulus | 12/344 | 235/18862 | 0.001324383 | 0.024295979 | 0.019850322 | MAP1LC3B2/NR4A2/BCL2/DCTPP1/BGLAP/LYN/MAPK1/FLCN/LPL/SREBF1/KLF10/RPTOR | 12 |
| GO:0001558 | BP | GO:0001558 | regulation of cell growth | 17/344 | 406/18862 | 0.001345588 | 0.024516324 | 0.020030347 | S100A9/MSX1/HPN/TMC8/WNT5A/APBB2/BCL2/SLC25A33/ISLR2/SEMA3B/CTTN/CYFIP1/CAMK2D/FLCN/SDCBP/CDC73/RPTOR | 17 |
| GO:0035116 | BP | GO:0035116 | embryonic hindlimb morphogenesis | 4/344 | 27/18862 | 0.001369327 | 0.024516324 | 0.020030347 | ALX4/MSX1/TWIST1/RARG | 4 |
| GO:0036037 | BP | GO:0036037 | CD8-positive, alpha-beta T cell activation | 4/344 | 27/18862 | 0.001369327 | 0.024516324 | 0.020030347 | HLA-E/BCL2/WDFY4/RUNX3 | 4 |
| GO:0060441 | BP | GO:0060441 | epithelial tube branching involved in lung morphogenesis | 4/344 | 27/18862 | 0.001369327 | 0.024516324 | 0.020030347 | TNF/FOXF1/FGFR2/ESRP2 | 4 |
| GO:0060512 | BP | GO:0060512 | prostate gland morphogenesis | 4/344 | 27/18862 | 0.001369327 | 0.024516324 | 0.020030347 | WNT5A/FGFR2/RARG/SERPINB5 | 4 |
| GO:1901214 | BP | GO:1901214 | regulation of neuron death | 14/344 | 302/18862 | 0.00137345 | 0.024516324 | 0.020030347 | GATA3/TNF/CTSZ/ADORA2A/NR4A2/EN1/WNT5A/BCL2/CLCF1/SYNGAP1/CASP8/HLA-F/UBE2V2/FYN | 14 |
| GO:0050810 | BP | GO:0050810 | regulation of steroid biosynthetic process | 7/344 | 91/18862 | 0.001377787 | 0.024516324 | 0.020030347 | GFI1/TNF/ADM/HMGCR/SREBF1/IDI1/FDFT1 | 7 |
| GO:0002685 | BP | GO:0002685 | regulation of leukocyte migration | 11/344 | 205/18862 | 0.001404289 | 0.024881115 | 0.02032839 | HOXA7/TNF/RIPK3/TNFRSF18/WNT5A/CD74/DUSP1/LYN/IL6R/MIF/MAPK1 | 11 |
| GO:0045619 | BP | GO:0045619 | regulation of lymphocyte differentiation | 10/344 | 175/18862 | 0.001436082 | 0.025336159 | 0.02070017 | GATA3/TCF7/TNFRSF18/HLX/CD74/RUNX3/HLA-DRA/INPP5D/PTPN6/ZBTB1 | 10 |
| GO:0045638 | BP | GO:0045638 | negative regulation of myeloid cell differentiation | 7/344 | 92/18862 | 0.001468397 | 0.025796507 | 0.021076284 | HOXA7/HOXA9/LYN/LTF/INPP5D/CDC73/MYC | 7 |
| GO:0002923 | BP | GO:0002923 | regulation of humoral immune response mediated by circulating immunoglobulin | 3/344 | 13/18862 | 0.001501375 | 0.026264561 | 0.021458694 | LTA/TNF/PTPN6 | 3 |
| GO:0060284 | BP | GO:0060284 | regulation of cell development | 19/344 | 485/18862 | 0.001555397 | 0.027095273 | 0.022137403 | BNC1/LTA/TNF/THRB/TCP11/WNT5A/BCL2/CLCF1/PAX6/ISLR2/SEMA3B/SYNGAP1/HDAC1/DLX2/LYN/CYFIP1/LTF/NODAL/SKIL | 19 |
| GO:0001782 | BP | GO:0001782 | B cell homeostasis | 4/344 | 28/18862 | 0.001574818 | 0.027204973 | 0.02222703 | BCL2/CD74/LYN/TNFSF13B | 4 |
| GO:0001893 | BP | GO:0001893 | maternal placenta development | 4/344 | 28/18862 | 0.001574818 | 0.027204973 | 0.02222703 | GJB2/NR2F2/MAPK1/NODAL | 4 |
| GO:0046631 | BP | GO:0046631 | alpha-beta T cell activation | 9/344 | 149/18862 | 0.001678628 | 0.028877977 | 0.023593909 | GATA3/HLA-E/HLX/BCL2/WDFY4/RUNX3/HLA-DRA/IL6R/HSPH1 | 9 |
| GO:0006469 | BP | GO:0006469 | negative regulation of protein kinase activity | 12/344 | 243/18862 | 0.001756775 | 0.029562755 | 0.024153387 | TRIM27/ADORA2A/NR2F2/EPHA1/PTK6/ZGPAT/DUSP1/LYN/CDKN1C/PTPN6/NPM1/RPTOR | 12 |
| GO:0045834 | BP | GO:0045834 | positive regulation of lipid metabolic process | 9/344 | 150/18862 | 0.001757819 | 0.029562755 | 0.024153387 | TNF/CPT1A/ADM/TWIST1/HTR2A/DGKZ/CD74/LYN/SREBF1 | 9 |
| GO:0001913 | BP | GO:0001913 | T cell mediated cytotoxicity | 5/344 | 48/18862 | 0.001764165 | 0.029562755 | 0.024153387 | HLA-E/RIPK3/PRF1/HLA-DRA/HLA-F | 5 |
| GO:0002762 | BP | GO:0002762 | negative regulation of myeloid leukocyte differentiation | 5/344 | 48/18862 | 0.001764165 | 0.029562755 | 0.024153387 | HOXA7/LYN/LTF/INPP5D/MYC | 5 |
| GO:0010522 | BP | GO:0010522 | regulation of calcium ion transport into cytosol | 7/344 | 95/18862 | 0.001768346 | 0.029562755 | 0.024153387 | LIME1/BCL2/LYN/PTPN6/CAMK2D/CALM3/FYN | 7 |
| GO:0032602 | BP | GO:0032602 | chemokine production | 7/344 | 95/18862 | 0.001768346 | 0.029562755 | 0.024153387 | TNF/S100A9/TWIST1/WNT5A/CD74/IL6R/LPL | 7 |
| GO:0060337 | BP | GO:0060337 | type I interferon signaling pathway | 7/344 | 95/18862 | 0.001768346 | 0.029562755 | 0.024153387 | PSMB8/HLA-E/WNT5A/XAF1/MX1/PTPN6/HLA-F | 7 |
| GO:0019218 | BP | GO:0019218 | regulation of steroid metabolic process | 8/344 | 122/18862 | 0.001793743 | 0.02986691 | 0.024401888 | GFI1/TNF/ADM/HMGCR/SREBF1/ARV1/IDI1/FDFT1 | 8 |
| GO:0007411 | BP | GO:0007411 | axon guidance | 13/344 | 277/18862 | 0.001807889 | 0.029982034 | 0.024495946 | GATA3/WNT5A/EPHA1/SPTBN1/SEMA3B/GDF7/TBR1/CYFIP1/MAPK1/LAMC2/RPS6KA5/YTHDF1/FYN | 13 |
| GO:0043627 | BP | GO:0043627 | response to estrogen | 6/344 | 71/18862 | 0.001863287 | 0.030703304 | 0.025085239 | GATA3/BGLAP/CITED4/ARPC1B/ASH2L/MAPK1 | 6 |
| GO:0097485 | BP | GO:0097485 | neuron projection guidance | 13/344 | 278/18862 | 0.001866192 | 0.030703304 | 0.025085239 | GATA3/WNT5A/EPHA1/SPTBN1/SEMA3B/GDF7/TBR1/CYFIP1/MAPK1/LAMC2/RPS6KA5/YTHDF1/FYN | 13 |
| GO:0071357 | BP | GO:0071357 | cellular response to type I interferon | 7/344 | 96/18862 | 0.001878231 | 0.030771118 | 0.025140645 | PSMB8/HLA-E/WNT5A/XAF1/MX1/PTPN6/HLA-F | 7 |
| GO:0002475 | BP | GO:0002475 | antigen processing and presentation via MHC class Ib | 3/344 | 14/18862 | 0.001885158 | 0.030771118 | 0.025140645 | HLA-E/TAP2/HLA-F | 3 |
| GO:0031960 | BP | GO:0031960 | response to corticosteroid | 9/344 | 152/18862 | 0.001925171 | 0.03117875 | 0.025473688 | TNF/GJB2/ADM/EIF4E/BCL2/BGLAP/DUSP1/HSD11B2/CALM3 | 9 |
| GO:0060402 | BP | GO:0060402 | calcium ion transport into cytosol | 9/344 | 152/18862 | 0.001925171 | 0.03117875 | 0.025473688 | LIME1/HTR2A/BCL2/RASA3/LYN/PTPN6/CAMK2D/CALM3/FYN | 9 |
| GO:0072132 | BP | GO:0072132 | mesenchyme morphogenesis | 5/344 | 49/18862 | 0.001935737 | 0.031227886 | 0.025513834 | MSX1/TWIST1/FOXF1/WNT5A/MYC | 5 |
| GO:0010800 | BP | GO:0010800 | positive regulation of peptidyl-threonine phosphorylation | 4/344 | 30/18862 | 0.002048333 | 0.032789139 | 0.02678941 | WNT5A/MAPK1/CALM3/RPTOR | 4 |
| GO:0071711 | BP | GO:0071711 | basement membrane organization | 4/344 | 30/18862 | 0.002048333 | 0.032789139 | 0.02678941 | HPN/SPINT2/COL4A1/PLOD3 | 4 |
| GO:2001235 | BP | GO:2001235 | positive regulation of apoptotic signaling pathway | 8/344 | 126/18862 | 0.00219795 | 0.035048843 | 0.028635635 | TNF/S100A9/MSX1/RIPK3/TRIM39/BCL2/PLAGL2/SKIL | 8 |
| GO:1901991 | BP | GO:1901991 | negative regulation of mitotic cell cycle phase transition | 12/344 | 250/18862 | 0.002224623 | 0.035338263 | 0.028872098 | PSMB8/PSMB9/ZNF385A/TRIM39/BCL2/DUSP1/E2F8/FBXO5/RAD17/CHEK2/GTSE1/CDC73 | 12 |
| GO:0002702 | BP | GO:0002702 | positive regulation of production of molecular mediator of immune response | 7/344 | 99/18862 | 0.002239871 | 0.035444674 | 0.028959037 | GATA3/HLA-E/WNT5A/CLCF1/CD74/HK1/HLA-F | 7 |
| GO:0048863 | BP | GO:0048863 | stem cell differentiation | 12/344 | 251/18862 | 0.002299078 | 0.036000166 | 0.029412886 | GATA3/PSMB8/HOXA7/PSMB9/MSX1/TWIST1/PITX2/GSC/TBX1/FGFR2/SEMA3B/MAPK1 | 12 |
| GO:0014066 | BP | GO:0014066 | regulation of phosphatidylinositol 3-kinase signaling | 8/344 | 127/18862 | 0.002309388 | 0.036000166 | 0.029412886 | IER3/TNF/LIME1/TWIST1/LYN/PTPN6/MAPK1/FYN | 8 |
| GO:0042733 | BP | GO:0042733 | embryonic digit morphogenesis | 5/344 | 51/18862 | 0.00231501 | 0.036000166 | 0.029412886 | ALX4/MSX1/TWIST1/WNT5A/HDAC1 | 5 |
| GO:0010644 | BP | GO:0010644 | cell communication by electrical coupling | 4/344 | 31/18862 | 0.00231839 | 0.036000166 | 0.029412886 | GJB2/GJB6/CAMK2D/CALM3 | 4 |
| GO:0048566 | BP | GO:0048566 | embryonic digestive tract development | 4/344 | 31/18862 | 0.00231839 | 0.036000166 | 0.029412886 | TNF/FOXF1/HLX/FGFR2 | 4 |
| GO:0045930 | BP | GO:0045930 | negative regulation of mitotic cell cycle | 14/344 | 321/18862 | 0.002415457 | 0.037367482 | 0.030530012 | PSMB8/TNF/PSMB9/ZNF385A/TRIM39/BCL2/DUSP1/E2F8/FBXO5/CDKN1C/RAD17/CHEK2/GTSE1/CDC73 | 14 |
| GO:0002479 | BP | GO:0002479 | antigen processing and presentation of exogenous peptide antigen via MHC class I, TAP-dependent | 6/344 | 75/18862 | 0.002465582 | 0.037860381 | 0.030932721 | PSMB8/TAP1/PSMB9/HLA-E/TAP2/HLA-F | 6 |
| GO:1902930 | BP | GO:1902930 | regulation of alcohol biosynthetic process | 6/344 | 75/18862 | 0.002465582 | 0.037860381 | 0.030932721 | GFI1/HRH1/HMGCR/SREBF1/IDI1/FDFT1 | 6 |
| GO:0032675 | BP | GO:0032675 | regulation of interleukin-6 production | 9/344 | 158/18862 | 0.002504816 | 0.038108203 | 0.031135196 | TNF/TWIST1/WNT5A/CD74/HSPD1/IL6R/INPP5D/PTPN6/LPL | 9 |
| GO:0034340 | BP | GO:0034340 | response to type I interferon | 7/344 | 101/18862 | 0.002509296 | 0.038108203 | 0.031135196 | PSMB8/HLA-E/WNT5A/XAF1/MX1/PTPN6/HLA-F | 7 |
| GO:0045621 | BP | GO:0045621 | positive regulation of lymphocyte differentiation | 7/344 | 101/18862 | 0.002509296 | 0.038108203 | 0.031135196 | GATA3/HLX/CD74/RUNX3/HLA-DRA/INPP5D/ZBTB1 | 7 |
| GO:0002285 | BP | GO:0002285 | lymphocyte activation involved in immune response | 10/344 | 189/18862 | 0.002531352 | 0.038302867 | 0.031294241 | GATA3/HLX/HLA-DMB/CLCF1/CD74/HSPD1/HLA-DRA/IL6R/APBB1IP/HLA-F | 10 |
| GO:0045787 | BP | GO:0045787 | positive regulation of cell cycle | 16/344 | 395/18862 | 0.002571169 | 0.038763877 | 0.031670895 | CCND2/MSX1/ZNF385A/EIF4E/WNT5A/PTK6/FGFR2/E2F8/FBXO5/MIF/CHEK2/GTSE1/CDC73/NUSAP1/NPM1/RPTOR | 16 |
| GO:0009954 | BP | GO:0009954 | proximal/distal pattern formation | 4/344 | 32/18862 | 0.002611983 | 0.038902812 | 0.031784408 | EN1/HOXA9/DLX2/NODAL | 4 |
| GO:1901099 | BP | GO:1901099 | negative regulation of signal transduction in absence of ligand | 4/344 | 32/18862 | 0.002611983 | 0.038902812 | 0.031784408 | TNF/EYA4/BCL2/FYN | 4 |
| GO:2001240 | BP | GO:2001240 | negative regulation of extrinsic apoptotic signaling pathway in absence of ligand | 4/344 | 32/18862 | 0.002611983 | 0.038902812 | 0.031784408 | TNF/EYA4/BCL2/FYN | 4 |
| GO:0006694 | BP | GO:0006694 | steroid biosynthetic process | 10/344 | 190/18862 | 0.002629903 | 0.038902812 | 0.031784408 | GFI1/TNF/HSD17B8/ADM/HMGCR/HSD11B2/SREBF1/ARV1/IDI1/FDFT1 | 10 |
| GO:0007492 | BP | GO:0007492 | endoderm development | 6/344 | 76/18862 | 0.002636684 | 0.038902812 | 0.031784408 | SOX7/PAX9/DUSP1/HDAC1/NODAL/CDC73 | 6 |
| GO:0009791 | BP | GO:0009791 | post-embryonic development | 6/344 | 76/18862 | 0.002636684 | 0.038902812 | 0.031784408 | GATA3/ALX4/NR4A2/BCL2/FGFR2/PLAGL2 | 6 |
| GO:0002449 | BP | GO:0002449 | lymphocyte mediated immunity | 15/344 | 360/18862 | 0.00265588 | 0.039047091 | 0.031902287 | GATA3/LTA/TNF/HLA-E/RIPK3/PRF1/DENND1B/CLCF1/CD74/HSPD1/HLA-DRA/INPP5D/PTPN6/ZBTB1/HLA-F | 15 |
| GO:0007612 | BP | GO:0007612 | learning | 8/344 | 130/18862 | 0.002670489 | 0.039123134 | 0.031964416 | HRH1/SHANK2/EN1/SYNGAP1/TBR1/DDHD2/YTHDF1/FYN | 8 |
| GO:0031214 | BP | GO:0031214 | biomineral tissue development | 9/344 | 160/18862 | 0.002726124 | 0.039657934 | 0.032401359 | TWIST1/TBX1/FGFR2/BGLAP/FBXO5/LTF/ANKH/SNX10/KLF10 | 9 |
| GO:0050680 | BP | GO:0050680 | negative regulation of epithelial cell proliferation | 9/344 | 160/18862 | 0.002726124 | 0.039657934 | 0.032401359 | GATA3/TNF/HPN/NR2F2/WNT5A/FGFR2/RUNX3/CDKN1C/CDC73 | 9 |
| GO:0032496 | BP | GO:0032496 | response to lipopolysaccharide | 14/344 | 326/18862 | 0.00277816 | 0.040133279 | 0.032789726 | LTA/GFI1/TNF/S100A9/GJB2/ADM/WNT5A/FGFR2/GJB6/LYN/CASP8/LTF/MAPK1/CDC73 | 14 |
| GO:0042113 | BP | GO:0042113 | B cell activation | 14/344 | 326/18862 | 0.00277816 | 0.040133279 | 0.032789726 | BCL2/CLCF1/CD74/HSPD1/CMTM7/LYN/CASP8/TNFSF13B/CD320/INPP5D/PTPN6/MIF/ZBTB1/KLF6 | 14 |
| GO:0048557 | BP | GO:0048557 | embryonic digestive tract morphogenesis | 3/344 | 16/18862 | 0.002822909 | 0.040638126 | 0.033202196 | FOXF1/HLX/FGFR2 | 3 |
| GO:0048015 | BP | GO:0048015 | phosphatidylinositol-mediated signaling | 10/344 | 192/18862 | 0.002836223 | 0.04068851 | 0.033243361 | GATA3/IER3/TNF/LIME1/TWIST1/HTR2A/LYN/PTPN6/MAPK1/FYN | 10 |
| GO:0062012 | BP | GO:0062012 | regulation of small molecule metabolic process | 17/344 | 437/18862 | 0.002902501 | 0.041484932 | 0.033894054 | PSMB8/GFI1/HRH1/TNF/PSMB9/CPT1A/ADM/TWIST1/HTR2A/CD74/HMGCR/FLCN/SREBF1/ARV1/IDI1/FDFT1/FOXK2 | 17 |
| GO:0032350 | BP | GO:0032350 | regulation of hormone metabolic process | 4/344 | 33/18862 | 0.002930099 | 0.041484932 | 0.033894054 | GATA3/GFI1/HPN/ADM | 4 |
| GO:0030879 | BP | GO:0030879 | mammary gland development | 8/344 | 132/18862 | 0.002934735 | 0.041484932 | 0.033894054 | GATA3/MSX1/FOXF1/WNT5A/FGFR2/HOXA9/MAPK1/DHODH | 8 |
| GO:0050731 | BP | GO:0050731 | positive regulation of peptidyl-tyrosine phosphorylation | 10/344 | 193/18862 | 0.002944125 | 0.041484932 | 0.033894054 | TNF/TNFRSF18/PTK6/HTR2A/CLCF1/CD74/LYN/IL6R/MIF/FYN | 10 |
| GO:0032635 | BP | GO:0032635 | interleukin-6 production | 9/344 | 162/18862 | 0.002962635 | 0.041484932 | 0.033894054 | TNF/TWIST1/WNT5A/CD74/HSPD1/IL6R/INPP5D/PTPN6/LPL | 9 |
| GO:0110148 | BP | GO:0110148 | biomineralization | 9/344 | 162/18862 | 0.002962635 | 0.041484932 | 0.033894054 | TWIST1/TBX1/FGFR2/BGLAP/FBXO5/LTF/ANKH/SNX10/KLF10 | 9 |
| GO:0002712 | BP | GO:0002712 | regulation of B cell mediated immunity | 5/344 | 54/18862 | 0.002981387 | 0.041484932 | 0.033894054 | LTA/TNF/HLA-E/CLCF1/PTPN6 | 5 |
| GO:0002889 | BP | GO:0002889 | regulation of immunoglobulin mediated immune response | 5/344 | 54/18862 | 0.002981387 | 0.041484932 | 0.033894054 | LTA/TNF/HLA-E/CLCF1/PTPN6 | 5 |
| GO:0001503 | BP | GO:0001503 | ossification | 16/344 | 401/18862 | 0.002981792 | 0.041484932 | 0.033894054 | TNF/TWIST1/WNT5A/ACP5/BCL2/FGFR2/BGLAP/HSPE1/RUNX3/FBXO5/IL6R/LTF/MAPK1/ANKH/SNX10/KLF10 | 16 |
| GO:0019216 | BP | GO:0019216 | regulation of lipid metabolic process | 16/344 | 402/18862 | 0.003055239 | 0.042364624 | 0.034612781 | GFI1/TNF/CPT1A/ADM/TWIST1/HTR2A/DGKZ/CD74/HMGCR/ACSL1/LYN/TXNRD1/SREBF1/ARV1/IDI1/FDFT1 | 16 |
| GO:0042303 | BP | GO:0042303 | molting cycle | 7/344 | 105/18862 | 0.003122334 | 0.0430073 | 0.03513786 | TNF/ALX4/WNT5A/BCL2/FGFR2/MPZL3/HDAC1 | 7 |
| GO:0042633 | BP | GO:0042633 | hair cycle | 7/344 | 105/18862 | 0.003122334 | 0.0430073 | 0.03513786 | TNF/ALX4/WNT5A/BCL2/FGFR2/MPZL3/HDAC1 | 7 |
| GO:1902806 | BP | GO:1902806 | regulation of cell cycle G1/S phase transition | 10/344 | 195/18862 | 0.003169759 | 0.043349521 | 0.035417463 | CCND2/ZNF385A/TRIM39/BCL2/E2F8/PTPN6/CHEK2/GTSE1/CDC73/RPTOR | 10 |
| GO:0045862 | BP | GO:0045862 | positive regulation of proteolysis | 15/344 | 367/18862 | 0.003182587 | 0.043349521 | 0.035417463 | TNF/TNFRSF10A/S100A9/HPN/DIABLO/SOX7/HSPE1/HSPD1/LYN/CASP8/TNFRSF10B/NODAL/MYC/UBE2V2/FYN | 15 |
| GO:0001776 | BP | GO:0001776 | leukocyte homeostasis | 6/344 | 79/18862 | 0.003203615 | 0.043349521 | 0.035417463 | RIPK3/BCL2/CD74/LYN/TNFSF13B/SKIL | 6 |
| GO:0048145 | BP | GO:0048145 | regulation of fibroblast proliferation | 6/344 | 79/18862 | 0.003203615 | 0.043349521 | 0.035417463 | LTA/WNT5A/CD74/MIF/CDC73/MYC | 6 |
| GO:0051348 | BP | GO:0051348 | negative regulation of transferase activity | 13/344 | 296/18862 | 0.003211237 | 0.043349521 | 0.035417463 | TRIM27/ADORA2A/NR2F2/EPHA1/PTK6/ZGPAT/DUSP1/LYN/FBXO5/CDKN1C/PTPN6/NPM1/RPTOR | 13 |
| GO:0097553 | BP | GO:0097553 | calcium ion transmembrane import into cytosol | 8/344 | 134/18862 | 0.003218927 | 0.043349521 | 0.035417463 | LIME1/HTR2A/RASA3/LYN/PTPN6/CAMK2D/CALM3/FYN | 8 |
| GO:0009266 | BP | GO:0009266 | response to temperature stimulus | 11/344 | 228/18862 | 0.00322037 | 0.043349521 | 0.035417463 | ADM/LXN/HTR2A/HSPD1/LYN/CASP8/CAMK2D/MAPK1/HSPH1/LPL/RPTOR | 11 |
| GO:0050730 | BP | GO:0050730 | regulation of peptidyl-tyrosine phosphorylation | 12/344 | 262/18862 | 0.003260794 | 0.043653144 | 0.035665528 | TNF/TNFRSF18/PTK6/HTR2A/ZGPAT/CLCF1/CD74/LYN/IL6R/PTPN6/MIF/FYN | 12 |
| GO:0035137 | BP | GO:0035137 | hindlimb morphogenesis | 4/344 | 34/18862 | 0.003273708 | 0.043653144 | 0.035665528 | ALX4/MSX1/TWIST1/RARG | 4 |
| GO:0042594 | BP | GO:0042594 | response to starvation | 10/344 | 196/18862 | 0.003287628 | 0.043653144 | 0.035665528 | MAP1LC3B2/ADM/BCL2/MAPK1/FLCN/SREBF1/KLF10/RPTOR/DHODH/FOXK2 | 10 |
| GO:0048017 | BP | GO:0048017 | inositol lipid-mediated signaling | 10/344 | 196/18862 | 0.003287628 | 0.043653144 | 0.035665528 | GATA3/IER3/TNF/LIME1/TWIST1/HTR2A/LYN/PTPN6/MAPK1/FYN | 10 |
| GO:0002429 | BP | GO:0002429 | immune response-activating cell surface receptor signaling pathway | 18/344 | 481/18862 | 0.003336551 | 0.043653144 | 0.035665528 | GATA3/PSMB8/PSMB9/LIME1/DENND1B/DGKZ/BCL2/LAT/ARPC1B/HLA-DRA/LYN/CYFIP1/INPP5D/PTPN6/HLA-DPA1/MAPK1/RPS6KA5/FYN | 18 |
| GO:0002757 | BP | GO:0002757 | immune response-activating signal transduction | 18/344 | 481/18862 | 0.003336551 | 0.043653144 | 0.035665528 | GATA3/PSMB8/PSMB9/LIME1/DENND1B/DGKZ/BCL2/LAT/ARPC1B/HLA-DRA/LYN/CYFIP1/INPP5D/PTPN6/HLA-DPA1/MAPK1/RPS6KA5/FYN | 18 |
| GO:0018108 | BP | GO:0018108 | peptidyl-tyrosine phosphorylation | 15/344 | 369/18862 | 0.003347944 | 0.043653144 | 0.035665528 | TNF/TNFRSF18/EPHA1/PTK6/HTR2A/FGFR2/ZGPAT/CLCF1/CD74/LYN/IL6R/PTPN6/MIF/BAZ1B/FYN | 15 |
| GO:0043524 | BP | GO:0043524 | negative regulation of neuron apoptotic process | 8/344 | 135/18862 | 0.003368812 | 0.043653144 | 0.035665528 | ADORA2A/NR4A2/EN1/BCL2/CLCF1/SYNGAP1/UBE2V2/FYN | 8 |
| GO:0042474 | BP | GO:0042474 | middle ear morphogenesis | 3/344 | 17/18862 | 0.003381872 | 0.043653144 | 0.035665528 | MSX1/GSC/TBX1 | 3 |
| GO:0060438 | BP | GO:0060438 | trachea development | 3/344 | 17/18862 | 0.003381872 | 0.043653144 | 0.035665528 | FOXF1/RARG/MAPK1 | 3 |
| GO:0072567 | BP | GO:0072567 | chemokine (C-X-C motif) ligand 2 production | 3/344 | 17/18862 | 0.003381872 | 0.043653144 | 0.035665528 | TNF/CD74/LPL | 3 |
| GO:2000341 | BP | GO:2000341 | regulation of chemokine (C-X-C motif) ligand 2 production | 3/344 | 17/18862 | 0.003381872 | 0.043653144 | 0.035665528 | TNF/CD74/LPL | 3 |
| GO:0043523 | BP | GO:0043523 | regulation of neuron apoptotic process | 10/344 | 197/18862 | 0.00340896 | 0.043653144 | 0.035665528 | GATA3/TNF/ADORA2A/NR4A2/EN1/BCL2/CLCF1/SYNGAP1/UBE2V2/FYN | 10 |
| GO:0001942 | BP | GO:0001942 | hair follicle development | 6/344 | 80/18862 | 0.003411389 | 0.043653144 | 0.035665528 | TNF/ALX4/WNT5A/BCL2/FGFR2/HDAC1 | 6 |
| GO:0042590 | BP | GO:0042590 | antigen processing and presentation of exogenous peptide antigen via MHC class I | 6/344 | 80/18862 | 0.003411389 | 0.043653144 | 0.035665528 | PSMB8/TAP1/PSMB9/HLA-E/TAP2/HLA-F | 6 |
| GO:0048144 | BP | GO:0048144 | fibroblast proliferation | 6/344 | 80/18862 | 0.003411389 | 0.043653144 | 0.035665528 | LTA/WNT5A/CD74/MIF/CDC73/MYC | 6 |
| GO:0032649 | BP | GO:0032649 | regulation of interferon-gamma production | 7/344 | 107/18862 | 0.003468773 | 0.044250865 | 0.036153879 | GATA3/LTA/TNF/RIPK3/WNT5A/HSPD1/HLA-DPA1 | 7 |
| GO:0007259 | BP | GO:0007259 | receptor signaling pathway via JAK-STAT | 9/344 | 166/18862 | 0.003484135 | 0.044310499 | 0.036202601 | TNF/TNFRSF18/PTK6/CLCF1/LYN/HGS/IL6R/STAT4/FYN | 9 |
| GO:0060760 | BP | GO:0060760 | positive regulation of response to cytokine stimulus | 5/344 | 56/18862 | 0.003496197 | 0.044327923 | 0.036216837 | GFI1/WNT5A/AGPAT1/CD74/TAF9 | 5 |
| GO:0018212 | BP | GO:0018212 | peptidyl-tyrosine modification | 15/344 | 372/18862 | 0.00360917 | 0.045545003 | 0.037211217 | TNF/TNFRSF18/EPHA1/PTK6/HTR2A/FGFR2/ZGPAT/CLCF1/CD74/LYN/IL6R/PTPN6/MIF/BAZ1B/FYN | 15 |
| GO:0071772 | BP | GO:0071772 | response to BMP | 9/344 | 167/18862 | 0.003625145 | 0.045545003 | 0.037211217 | GATA3/MSX1/WNT5A/SPINT2/RGMA/GDF7/ZFYVE16/NODAL/SKIL | 9 |
| GO:0071773 | BP | GO:0071773 | cellular response to BMP stimulus | 9/344 | 167/18862 | 0.003625145 | 0.045545003 | 0.037211217 | GATA3/MSX1/WNT5A/SPINT2/RGMA/GDF7/ZFYVE16/NODAL/SKIL | 9 |
| GO:0048854 | BP | GO:0048854 | brain morphogenesis | 4/344 | 35/18862 | 0.003643763 | 0.04564061 | 0.03728933 | SHANK2/WNT5A/OTX1/GDF7 | 4 |
| GO:0051091 | BP | GO:0051091 | positive regulation of DNA-binding transcription factor activity | 12/344 | 266/18862 | 0.003682282 | 0.045984157 | 0.037570015 | FLOT1/TRIM27/TNF/S100A9/RIPK3/WNT5A/LTF/RPS6KA5/TAF12/NODAL/NPM1/FLOT2 | 12 |
| GO:2000242 | BP | GO:2000242 | negative regulation of reproductive process | 5/344 | 57/18862 | 0.003776204 | 0.047015439 | 0.038412594 | ARHGDIB/WNT5A/DUSP1/FBXO5/NODAL | 5 |
| GO:0033673 | BP | GO:0033673 | negative regulation of kinase activity | 12/344 | 267/18862 | 0.003794234 | 0.04709849 | 0.038480448 | TRIM27/ADORA2A/NR2F2/EPHA1/PTK6/ZGPAT/DUSP1/LYN/CDKN1C/PTPN6/NPM1/RPTOR | 12 |
| GO:0001843 | BP | GO:0001843 | neural tube closure | 6/344 | 82/18862 | 0.003856808 | 0.047590255 | 0.03888223 | ADM/TWIST1/GRHL3/WNT5A/SPINT2/RARG | 6 |
| GO:0002065 | BP | GO:0002065 | columnar/cuboidal epithelial cell differentiation | 6/344 | 82/18862 | 0.003856808 | 0.047590255 | 0.03888223 | WNT5A/PTK6/FGFR2/PAX6/RARG/NODAL | 6 |
| GO:1901988 | BP | GO:1901988 | negative regulation of cell cycle phase transition | 12/344 | 268/18862 | 0.003908913 | 0.048090067 | 0.039290587 | PSMB8/PSMB9/ZNF385A/TRIM39/BCL2/DUSP1/E2F8/FBXO5/RAD17/CHEK2/GTSE1/CDC73 | 12 |
| GO:0016050 | BP | GO:0016050 | vesicle organization | 14/344 | 339/18862 | 0.00393505 | 0.048268399 | 0.039436288 | TAP1/CTSZ/ZNF385A/BCL2/TAP2/HGS/FBXO5/MX1/CPLX1/ZFYVE16/SDCBP/PLEKHJ1/SNX10/VAPA | 14 |
| GO:0060065 | BP | GO:0060065 | uterus development | 3/344 | 18/18862 | 0.0040039 | 0.04882403 | 0.03989025 | GATA3/WNT5A/HOXA9 | 3 |
| GO:0060252 | BP | GO:0060252 | positive regulation of glial cell proliferation | 3/344 | 18/18862 | 0.0040039 | 0.04882403 | 0.03989025 | LTA/TNF/LYN | 3 |
| GO:0030224 | BP | GO:0030224 | monocyte differentiation | 4/344 | 36/18862 | 0.004041202 | 0.048847881 | 0.039909737 | HOXA7/CD74/INPP5D/MYC | 4 |
| GO:0032435 | BP | GO:0032435 | negative regulation of proteasomal ubiquitin-dependent protein catabolic process | 4/344 | 36/18862 | 0.004041202 | 0.048847881 | 0.039909737 | TRIM39/TAF9/SDCBP/SMARCC1 | 4 |
| GO:0042092 | BP | GO:0042092 | type 2 immune response | 4/344 | 36/18862 | 0.004041202 | 0.048847881 | 0.039909737 | GATA3/DENND1B/HLX/CD74 | 4 |
| GO:0022404 | BP | GO:0022404 | molting cycle process | 6/344 | 83/18862 | 0.004095015 | 0.049069162 | 0.040090529 | TNF/ALX4/WNT5A/BCL2/FGFR2/HDAC1 | 6 |
| GO:0022405 | BP | GO:0022405 | hair cycle process | 6/344 | 83/18862 | 0.004095015 | 0.049069162 | 0.040090529 | TNF/ALX4/WNT5A/BCL2/FGFR2/HDAC1 | 6 |
| GO:0060606 | BP | GO:0060606 | tube closure | 6/344 | 83/18862 | 0.004095015 | 0.049069162 | 0.040090529 | ADM/TWIST1/GRHL3/WNT5A/SPINT2/RARG | 6 |
| GO:0042611 | CC | GO:0042611 | MHC protein complex | 7/352 | 25/19520 | 2.12E-07 | 9.13E-05 | 7.87E-05 | HLA-E/HLA-DMA/HLA-DMB/CD74/HLA-DRA/HLA-DPA1/HLA-F | 7 |
| GO:0042613 | CC | GO:0042613 | MHC class II protein complex | 5/352 | 16/19520 | 6.87E-06 | 0.001478053 | 0.001273622 | HLA-DMA/HLA-DMB/CD74/HLA-DRA/HLA-DPA1 | 5 |
| GO:0098576 | CC | GO:0098576 | lumenal side of membrane | 6/352 | 36/19520 | 4.06E-05 | 0.005410928 | 0.004662537 | HLA-E/CD74/HLA-DRA/HLA-DPA1/CTSA/HLA-F | 6 |
| GO:0030134 | CC | GO:0030134 | COPII-coated ER to Golgi transport vesicle | 9/352 | 94/19520 | 5.03E-05 | 0.005410928 | 0.004662537 | CTSZ/HLA-E/CD74/HLA-DRA/TMED5/HLA-DPA1/HLA-F/SREBF1/DDHD2 | 9 |
| GO:0071556 | CC | GO:0071556 | integral component of lumenal side of endoplasmic reticulum membrane | 5/352 | 29/19520 | 0.000154138 | 0.009890728 | 0.008522732 | HLA-E/CD74/HLA-DRA/HLA-DPA1/HLA-F | 5 |
| GO:0098553 | CC | GO:0098553 | lumenal side of endoplasmic reticulum membrane | 5/352 | 29/19520 | 0.000154138 | 0.009890728 | 0.008522732 | HLA-E/CD74/HLA-DRA/HLA-DPA1/HLA-F | 5 |
| GO:0098978 | CC | GO:0098978 | glutamatergic synapse | 16/352 | 308/19520 | 0.000161012 | 0.009890728 | 0.008522732 | FLOT1/PRRT1/EIF4E/ADORA2A/WNT5A/HTR2A/DGKZ/SYN3/KPNA2/SYNGAP1/LYN/ARPC5L/CPLX1/YWHAZ/DLGAP1/FYN | 16 |
| GO:0030666 | CC | GO:0030666 | endocytic vesicle membrane | 10/352 | 163/19520 | 0.000770426 | 0.041363584 | 0.03564255 | TAP1/HLA-E/WNT5A/TAP2/CD74/HLA-DRA/HLA-DPA1/CAMK2D/ATP6V0E2/HLA-F | 10 |
| GO:0012507 | CC | GO:0012507 | ER to Golgi transport vesicle membrane | 6/352 | 62/19520 | 0.000865749 | 0.041363584 | 0.03564255 | HLA-E/CD74/HLA-DRA/HLA-DPA1/HLA-F/SREBF1 | 6 |
| GO:0042605 | MF | GO:0042605 | peptide antigen binding | 6/350 | 32/18337 | 2.76E-05 | 0.016554882 | 0.01598784 | TAP1/HLA-E/TAP2/HLA-DRA/HLA-DPA1/HLA-F | 6 |
| GO:0001216 | MF | GO:0001216 | DNA-binding transcription activator activity | 22/350 | 447/18337 | 5.26E-05 | 0.016554882 | 0.01598784 | GATA3/BNC1/HOXA7/ALX4/MSX1/GRHL3/FOXF1/NR4A2/HOXD3/OTX1/HOXA9/HOXD4/PAX9/DLX2/PLAGL2/FOXJ2/SREBF1/MYC/MYBL1/KLF10/KLF6/FOXK2 | 22 |
| GO:0001228 | MF | GO:0001228 | DNA-binding transcription activator activity, RNA polymerase II-specific | 21/350 | 443/18337 | 0.000131232 | 0.027558782 | 0.02661483 | GATA3/HOXA7/ALX4/MSX1/GRHL3/FOXF1/NR4A2/HOXD3/OTX1/HOXA9/HOXD4/PAX9/DLX2/PLAGL2/FOXJ2/SREBF1/MYC/MYBL1/KLF10/KLF6/FOXK2 | 21 |
| GO:0023026 | MF | GO:0023026 | MHC class II protein complex binding | 4/350 | 17/18337 | 0.000255051 | 0.040170548 | 0.038794615 | HLA-DMA/HLA-DMB/CD74/HLA-DRA | 4 |
