# Supplementary material for: Exploration of Biomarkers of Psoriasis through Combined Multiomics Analysis
Source: Mediators Inflamm. 2022 Sep 23;2022:7731082. doi: 10.1155/2022/7731082 (PMC9525798; doi:10.1155/2022/7731082)
Supplement: Supplementary Materials — Supplementary Figure 1 The PCA of gene expression in psoriasis lesions and healthy controls in GSE13355 database. Supplementary Figure 2 The PCA and methylation distribution density in psoriasis lesions and healthy controls from the GSE73894 dataset. (A) PCA in GSE73894. (B) Methylation distribution density in GSE73894. Supplementary Table 1 Identification of DEGs in the psoriatic lesions and healthy control group in GSE13355. Supplementary Table 2 GO analysis on 767 DEGs in GSE13355. Supplementary Table 3 KEGG analysis on 767 DEGs in GSE13355. Supplementary Table 4 Identification of hyper-MR-genes. Supplementary Table 5 Identification of hypo-MR-genes. Supplementary Table 6 GO analysis of hyper-MR-genes. Supplementary Table 7 GO analysis of hypo-MR-genes. Supplementary Table 8 KEGG analysis of hyper-MR-genes. Supplementary Table 9 KEGG analysis of hypo-MR-genes. Supplementary Table 10 GO analysis through single-gene GSEA of GJB2. Supplementary Table 11 KEGG analysis through single-gene GSEA of GJB2. [file 7731082.f1.zip › Supplementary Table 9 (1).docx]

| KEGG analysis of hypo-MR-genes | | | | | | | | |
| --- | --- | --- | --- | --- | --- | --- | --- | --- |
| ID | Description | GeneRatio | BgRatio | pvalue | p.adjust | qvalue | geneID | Count |
| hsa04940 | Type I diabetes mellitus | 10/180 | 43/8101 | 2.32E-08 | 6.08E-06 | 4.59E-06 | LTA/TNF/HLA-E/HLA-DMA/PRF1/HLA-DMB/HSPD1/HLA-DRA/HLA-DPA1/HLA-F | 10 |
| hsa05169 | Epstein-Barr virus infection | 18/180 | 202/8101 | 4.77E-07 | 5.75E-05 | 4.34E-05 | TAP1/TNF/CCND2/HLA-E/HLA-DMA/HLA-DMB/BCL2/TAP2/SKP2/HDAC1/RUNX3/HLA-DRA/LYN/CASP8/HLA-DPA1/HLA-F/MYC/PIK3R3 | 18 |
| hsa05416 | Viral myocarditis | 10/180 | 60/8101 | 6.58E-07 | 5.75E-05 | 4.34E-05 | HLA-E/HLA-DMA/PRF1/HLA-DMB/HLA-DRA/CASP8/HLA-DPA1/EIF4G3/HLA-F/FYN | 10 |
| hsa05330 | Allograft rejection | 8/180 | 38/8101 | 1.41E-06 | 9.21E-05 | 6.96E-05 | TNF/HLA-E/HLA-DMA/PRF1/HLA-DMB/HLA-DRA/HLA-DPA1/HLA-F | 8 |
| hsa05332 | Graft-versus-host disease | 8/180 | 42/8101 | 3.15E-06 | 0.000164897 | 0.000124551 | TNF/HLA-E/HLA-DMA/PRF1/HLA-DMB/HLA-DRA/HLA-DPA1/HLA-F | 8 |
| hsa04612 | Antigen processing and presentation | 10/180 | 78/8101 | 7.77E-06 | 0.000339461 | 0.000256403 | TAP1/TNF/HLA-E/HLA-DMA/HLA-DMB/TAP2/CD74/HLA-DRA/HLA-DPA1/HLA-F | 10 |
| hsa05166 | Human T-cell leukemia virus 1 infection | 15/180 | 222/8101 | 0.000118575 | 0.004438109 | 0.003352208 | LTA/TNF/CCND2/HLA-E/HLA-DMA/MSX1/HLA-DMB/HLA-DRA/HLA-DPA1/CHEK2/MAPK1/VDAC2/HLA-F/MYC/PIK3R3 | 15 |
| hsa05320 | Autoimmune thyroid disease | 7/180 | 53/8101 | 0.000154718 | 0.004747134 | 0.003585621 | HLA-E/HLA-DMA/PRF1/HLA-DMB/HLA-DRA/HLA-DPA1/HLA-F | 7 |
| hsa05145 | Toxoplasmosis | 10/180 | 112/8101 | 0.000182236 | 0.004747134 | 0.003585621 | TNF/HLA-DMA/HLA-DMB/BCL2/ITGA6/HLA-DRA/CASP8/HLA-DPA1/MAPK1/LAMC2 | 10 |
| hsa04658 | Th1 and Th2 cell differentiation | 9/180 | 92/8101 | 0.000192292 | 0.004747134 | 0.003585621 | GATA3/HLA-DMA/HLA-DMB/LAT/RUNX3/HLA-DRA/STAT4/HLA-DPA1/MAPK1 | 9 |
| hsa04115 | p53 signaling pathway | 8/180 | 73/8101 | 0.000199307 | 0.004747134 | 0.003585621 | CCND2/TNFRSF10A/BCL2/CASP8/CHEK2/GTSE1/SERPINB5/TNFRSF10B | 8 |
| hsa04350 | TGF-beta signaling pathway | 9/180 | 94/8101 | 0.00022665 | 0.004948519 | 0.003737732 | TNF/PITX2/RGMA/GDF7/MAPK1/ZFYVE16/NODAL/MYC/TGIF2 | 9 |
| hsa04550 | Signaling pathways regulating pluripotency of stem cells | 11/180 | 143/8101 | 0.000325566 | 0.006561406 | 0.004955984 | TCF7/PCGF3/WNT5A/OTX1/FGFR2/PAX6/MAPK1/NODAL/SKIL/MYC/PIK3R3 | 11 |
| hsa05164 | Influenza A | 12/180 | 171/8101 | 0.00040862 | 0.00764703 | 0.005775981 | TNF/TNFRSF10A/HLA-DMA/HLA-DMB/KPNA2/HLA-DRA/CASP8/MX1/HLA-DPA1/MAPK1/TNFRSF10B/PIK3R3 | 12 |
| hsa05310 | Asthma | 5/180 | 31/8101 | 0.000544471 | 0.009147069 | 0.006908996 | TNF/HLA-DMA/HLA-DMB/HLA-DRA/HLA-DPA1 | 5 |
| hsa05321 | Inflammatory bowel disease | 7/180 | 65/8101 | 0.0005586 | 0.009147069 | 0.006908996 | GATA3/TNF/HLA-DMA/HLA-DMB/HLA-DRA/STAT4/HLA-DPA1 | 7 |
| hsa04650 | Natural killer cell mediated cytotoxicity | 10/180 | 131/8101 | 0.000645965 | 0.009444894 | 0.00713395 | TNF/TNFRSF10A/HLA-E/PRF1/LAT/PTPN6/MAPK1/TNFRSF10B/PIK3R3/FYN | 10 |
| hsa05152 | Tuberculosis | 12/180 | 180/8101 | 0.000648886 | 0.009444894 | 0.00713395 | TNF/HLA-DMA/HLA-DMB/BCL2/CD74/HSPD1/HLA-DRA/CASP8/HLA-DPA1/CAMK2D/MAPK1/CALM3 | 12 |
| hsa04664 | Fc epsilon RI signaling pathway | 7/180 | 68/8101 | 0.000735585 | 0.010143329 | 0.007661494 | TNF/LAT/LYN/INPP5D/MAPK1/PIK3R3/FYN | 7 |
| hsa04210 | Apoptosis | 10/180 | 136/8101 | 0.000865393 | 0.011336653 | 0.00856284 | TNF/CTSZ/TNFRSF10A/DIABLO/PRF1/BCL2/CASP8/MAPK1/TNFRSF10B/PIK3R3 | 10 |
| hsa05323 | Rheumatoid arthritis | 8/180 | 93/8101 | 0.001036031 | 0.01273738 | 0.009620841 | TNF/HLA-DMA/HLA-DMB/ACP5/HLA-DRA/TNFSF13B/HLA-DPA1/ATP6V0E2 | 8 |
| hsa05165 | Human papillomavirus infection | 17/180 | 331/8101 | 0.001069551 | 0.01273738 | 0.009620841 | TNXB/TCF7/TNF/CCND2/HLA-E/WNT5A/ITGA6/HDAC1/CHAD/CASP8/MX1/MAPK1/LAMC2/ATP6V0E2/COL4A1/HLA-F/PIK3R3 | 17 |
| hsa05140 | Leishmaniasis | 7/180 | 77/8101 | 0.001543567 | 0.017583236 | 0.01328103 | TNF/HLA-DMA/HLA-DMB/HLA-DRA/PTPN6/HLA-DPA1/MAPK1 | 7 |
| hsa04145 | Phagosome | 10/180 | 152/8101 | 0.002012333 | 0.021967964 | 0.016592917 | TAP1/HLA-E/HLA-DMA/HLA-DMB/TAP2/HLA-DRA/HGS/HLA-DPA1/ATP6V0E2/HLA-F | 10 |
| hsa04218 | Cellular senescence | 10/180 | 156/8101 | 0.002437246 | 0.025542334 | 0.019292723 | CCND2/HLA-E/CHEK2/MAPK1/VDAC2/RASSF5/HLA-F/CALM3/MYC/PIK3R3 | 10 |
| hsa04659 | Th17 cell differentiation | 8/180 | 108/8101 | 0.002702617 | 0.027234062 | 0.020570525 | GATA3/HLA-DMA/HLA-DMB/LAT/HLA-DRA/IL6R/HLA-DPA1/MAPK1 | 8 |
| hsa04066 | HIF-1 signaling pathway | 8/180 | 109/8101 | 0.002862567 | 0.027777498 | 0.020980995 | EIF4E/BCL2/IL6R/CAMK2D/MAPK1/HK1/CUL2/PIK3R3 | 8 |
| hsa04910 | Insulin signaling pathway | 9/180 | 137/8101 | 0.003379739 | 0.031624697 | 0.023886875 | FLOT1/EIF4E/MAPK1/HK1/SREBF1/CALM3/FLOT2/RPTOR/PIK3R3 | 9 |
| hsa05132 | Salmonella infection | 13/180 | 249/8101 | 0.003591819 | 0.032450222 | 0.024510413 | TCF7/TNF/TNFRSF10A/RIPK3/BCL2/GSDMD/ARPC1B/CASP8/ARPC5L/CYFIP1/MAPK1/TNFRSF10B/MYC | 13 |
| hsa05222 | Small cell lung cancer | 7/180 | 92/8101 | 0.004258865 | 0.036257311 | 0.027385996 | BCL2/SKP2/ITGA6/LAMC2/COL4A1/MYC/PIK3R3 | 7 |
| hsa05130 | Pathogenic Escherichia coli infection | 11/180 | 197/8101 | 0.004385181 | 0.036257311 | 0.027385996 | TNF/TNFRSF10A/ARPC1B/CTTN/CASP8/ARPC5L/CYFIP1/PTPN6/MAPK1/TNFRSF10B/FYN | 11 |
| hsa04672 | Intestinal immune network for IgA production | 5/180 | 49/8101 | 0.004428374 | 0.036257311 | 0.027385996 | HLA-DMA/HLA-DMB/HLA-DRA/TNFSF13B/HLA-DPA1 | 5 |
| hsa05203 | Viral carcinogenesis | 11/180 | 204/8101 | 0.005690188 | 0.04387909 | 0.033142904 | CCND2/HLA-E/HPN/SKP2/HDAC1/LYN/CASP8/MAPK1/YWHAZ/HLA-F/PIK3R3 | 11 |
| hsa04666 | Fc gamma R-mediated phagocytosis | 7/180 | 97/8101 | 0.005694233 | 0.04387909 | 0.033142904 | LAT/ARPC1B/LYN/ARPC5L/INPP5D/MAPK1/PIK3R3 | 7 |
| hsa04640 | Hematopoietic cell lineage | 7/180 | 99/8101 | 0.00635981 | 0.047607718 | 0.035959225 | TNF/HLA-DMA/HLA-DMB/ITGA6/HLA-DRA/IL6R/HLA-DPA1 | 7 |
